# A Platform for the Development of Highly Red‐Shifted Azobenzene‐Based Optical Tools

**DOI:** 10.1002/anie.202501779

**Published:** 2025-06-23

**Authors:** Kyra Lützel, Henryk Laqua, Manjima B. Sathian, Benedikt Nißl, Judit Katalin Szántó, Christina‐Anna Senser, Gökcen Savasci, Lars Allmendinger, Bilal Kicin, Vincent Ruf, Dominik Kammerer, Theobald Lohmüller, Konstantin Karaghiosoff, Ahmed M. Ali, Ursula Storch, Michael Mederos y Schnitzler, Christian Ochsenfeld, David B. Konrad

**Affiliations:** ^1^ Department of Pharmacy Ludwig‐Maximilians‐Universität München Butenandtstr. 5–13 Munich 81377 Germany; ^2^ Department of Chemistry Ludwig‐Maximilians‐Universität München Butenandtstr. 5–13 Munich 81377 Germany; ^3^ Walther Straub Institute of Pharmacology and Toxicology Ludwig‐Maximilians‐Universität München Goethestr. 33 Munich 80336 Germany; ^4^ College of Chemistry University of California Berkeley California 94720 USA; ^5^ Max Planck Institute for Solid State Research Heisenbergstr. 1 Stuttgart 70569 Germany; ^6^ Chair for Photonics and Optoelectronics Nano‐Institute Munich Department of Physics Ludwig‐Maximilians‐Universität München Königinstraße 10 Munich 80539 Germany; ^7^ Department of Medicinal Chemistry Faculty of Pharmacy Assiut University Assiut 71515 Egypt; ^8^ Institute of Pharmacy University of Regensburg Universitätsstr. 31 Regensburg 93040 Germany; ^9^ Department of Pharmaceutical Sciences University of Vienna Josef‐Holaubek‐Platz 2 Vienna 1090 Austria

**Keywords:** Chemical probe development, In silico design, Photophysical properties, Red‐shifted azobenzenes

## Abstract

Azobenzenes are versatile photoswitches that can be used to generate elaborate optical tools, including photopharmaceuticals. However, the targeted application‐guided design of new photoswitches with specific properties remains challenging. We have developed synthetic protocols for derivatives of the **dfdc** (di‐*ortho‐*fluoro‐di‐*ortho‐*chloro) azobenzene scaffold with chemical alterations in the *para*‐/*ortho*‐positions and performed an in‐depth study into the effects of their structures on their photophysical properties with an emphasis on the n → π* absorption band using NMR, UV–vis, and X‐ray analysis. The data was used to establish and validate a computational approach that allows to compute realistic UV–vis spectra by combining TD‐DFT excited‐state calculations from 6000 thermally accessible structures generated through MD simulations while considering the high structural flexibility of *ortho*‐substituted azobenzenes. We added 15 new visible light‐operated photoswitches to the toolbox for the development of optical devices with relaxation rates across multiple orders of magnitude and identified several examples with stronger bathochromic shifts than the **dfdc** azobenzene lead structure. Our combined experimental and computational study forms the foundation for the advanced in silico design and synthesis of new highly red‐shifted photoswitches. To showcase the potential of **dfdc** azobenzenes for the development of chemical tools, we synthesized **dfdc**‐OptoBI‐1 and demonstrated its biological activity as a red light‐operated activator of TRPC6 channels in HEK293 cells.

## Graphical abstract

Our platform is built on a combination of chemical synthesis, NMR, UV–vis, and quantum chemical studies. The **dfdc** azobenzene scaffold provides efficient photoswitching with red light and the ability to fine‐tune relaxation times. A TD‐DFT‐based workflow allows the in silico design of photoswitches with properties tailored to desired applications. The strong potential is demonstrated by the development of a red light‐operated TRPC6 activator.

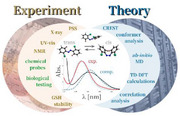

## Introduction

Azobenzenes are versatile photoswitches that can be cycled between their *cis*‐ and *trans*‐configurations with light.^[^ ^1^, ^2^ ^]^ Due to their small size, robust photoswitching, synthetic accessibility, and low rate of photobleaching, they serve as excellent building blocks for the generation of elaborate optical tools.^[^ ^3^, ^4^, ^5^, ^6^ ^]^ Photopharmaceuticals, for example, contain azobenzene fragments as ON and OFF switches which allow for the control of biological functions with the spatiotemporal precision of light.^[^ ^6^, ^7^, ^8^, ^9^ ^]^ To elicit the full potential of these optical tools and to apply them to complex animal tissues, it is crucial to use tissue‐penetrating, non‐hazardous red or near‐infrared (NIR) light within the bio‐optical window (650–950 nm).^[^ ^10^, ^11^ ^]^ It is highly desirable to have access to an arsenal of photoswitches with candidates that are subject to thermal relaxation rates spanning multiple orders of magnitude for the less stable isomers. Slow‐relaxing photopharmaceuticals allow photoswitching between the two isomers through light pulses whereby the established photostationary state (PSS), i.e., the established *cis*:*trans* ratios, is retained over prolonged time periods. Fast‐relaxing azobenzenes require constant illumination with high intensity light for photoswitching to outcompete the thermal relaxation, which becomes difficult in deeper tissue layers.^[^ ^12^ ^]^ However, they allow for bidirectional switching by alternating between irradiation to access the metastable state and relaxation in the dark. This holds a key advantage in case the photoswitching wavelength to access the less stable isomer falls within the biooptical window while the back‐switching requires higher energy light. In addition, the availability of a toolbox of red‐shifted azobenzene substitution patterns with relaxation half‐lives at varying time scales is beneficial to optimize photoresponsive tools to tailored applications.^[^ ^13^ ^]^

The thermal stability of the metastable isomer and the illumination range of azobenzenes are strongly influenced by their substitution pattern.^[^ ^12^ ^]^ With the exception of the diazocine substitution pattern,^[^ ^14^, ^15^ ^]^ the *trans‐*isomer is slightly more stable than the *cis* ^[^ ^16^, ^17^ ^]^ isomer and can be reached through thermal relaxation in the dark. A strategy to shift the isomerization to longer wavelengths entails the implementation of electron‐active structural modifications or substituting one phenyl ring for a heterocycle.^[^ ^18^, ^19^ ^]^ For example, the modification of an azobenzene group with an electron‐rich substituent shifts the *trans*‐to‐*cis* isomerization (π → π* excitation band) into the blue range of the visible spectrum and due to the rapid relaxation rate of this substitution pattern, the back‐isomerization proceeds in the dark.^[^ ^18^ ^]^ By using a “push–pull” system, the effects that were found for electron‐rich azobenzenes are increased, which is highlighted by the *cis*‐isomerization with green light and a faster relaxation rate in the dark.^[^ ^20^ ^]^ A predominant approach to allow for photoswitching with visible light is the implementation of a tetra‐*ortho*‐substitution pattern with electron‐active substituents.^[^ ^21^, ^22^, ^23^, ^24^ ^]^ This causes the n → π* transition bands between the two isomers to separate in the visible range. The underlying mechanisms can be divided into two categories: 1) A decrease in the electron density around the diazene unit, which stabilizes the n molecular orbital and leads to a hypsochromic shift by increasing the gap to the π* molecular orbital.^[^ ^25^, ^26^ ^]^ For the tetra‐*ortho*‐fluoro (**tof**) substitution pattern, for instance, this effect has a stronger impact on the *cis*‐state^[^ ^26^ ^]^ which leads to a significantly increased thermal stability and enables addressing the n → π* transition to effect a *cis*‐ to *trans‐*isomerization with blue light, while allowing to selectively address the absorption band in the *trans*‐state to effect a *cis*‐isomerization with green light.^[^ ^23^, ^26^ ^]^ 2) With an increasing size of the *ortho*‐substituents, their repulsive interaction with the nitrogen lone pairs destabilizes the n molecular orbital, which is accompanied by a bathochromic shift through a decreased gap to the π* molecular orbital.^[^ ^21^, ^22^, ^25^, ^27^ ^]^ For tetra‐*ortho*‐chloro (**toc**) azobenzenes, the latter effect is stronger pronounced for the *trans*‐isomer, which enables the use of lower energy light to address the n → π* excitation band.^[^ ^25^ ^]^ The resultant advantage of the **toc** azobenzene over the **tof** substitution pattern is the ability to induce a *trans*‐to‐*cis* isomerization with deep red light, which is, however, accompanied by a twisted conformation of the *trans*‐isomer out of the plane and a decrease in the stability of the *cis* isomer. Both **tof** and **toc** azobenzenes allow for near‐quantitative, bidirectional photoswitching by irradiation with visible light.^[^ ^22^, ^25^, ^26^, ^27^ ^]^ For **tof** azobenzenes, additional substitution with electron‐withdrawing groups lower n orbital energies, which results in a shift of the n → π* band toward longer wavelengths for the *trans‐*isomer relative to the *cis‐*isomer, while electron‐donating substituents have the opposite effect.^[^ ^23^, ^26^ ^]^ A study that established general principles for the design of **toc** azobenzenes reported a red‐shift for electron‐withdrawing and electron‐donating groups.^[^ ^27^ ^]^

The hybrid di‐*ortho‐*fluoro di‐*ortho‐*chloro (**dfdc**) azobenzene combines the advantageous properties of both the **toc** and **tof** pattern, including *trans*‐to‐*cis* isomerization with deep red light. Compared to **toc** substitution, it exhibits an increased separation of the n → π* excitation band between the *trans‐* and *cis‐*isomer, which enables generating near‐quantitative PSS with high levels of *trans*‐ and *cis*‐isomers with a broader range of wavelengths. The reduced bulk of fluorine compared to chlorine leads to a near‐planar structure of the *trans*‐isomer and thereby establishes a closer resemblance to azobenzene (**1**) compared to the twisted conformation that is present with the **toc** substitution.^[^ ^25^ ^]^ It was hypothesized that this feature is important to consider when red‐shifting preexisting photopharmaceuticals by exchanging azobenzene (**1**) for a substituted photoswitch.^[^ ^25^, ^28^ ^]^ The near‐bistable nature of both isomers is retained from the **tof** pattern.^[^ ^25^ ^]^

Due to their excellent photophysical properties, we were intrigued by the potential of the **dfdc** pattern as a foundation to build a toolbox of modified azobenzenes suitable for in vivo photopharmacology with excitation wavelengths within the bio‐optical window and examples with varying *cis*‐isomer stability levels. This study is important to assess substitution‐dependent changes in the photophysical properties that occur with modifications necessary to incorporate the photoswitches into light‐responsive tools. Our approach focused on the development of synthetic protocols for derivatives of the **dfdc** azobenene scaffold with various chemical alterations in the *para*‐/*ortho*‐positions and examining the effect of these substitutions on extent as well as shift of the n → π absorption band, the stability of this *cis*‐isomer and the resistance of both isomers toward reduction via glutathione (GSH), which is a potential deactivation mechanism of azobenzene photoswitches in vivo.^[^ ^29^ ^]^

**Figure 1:**
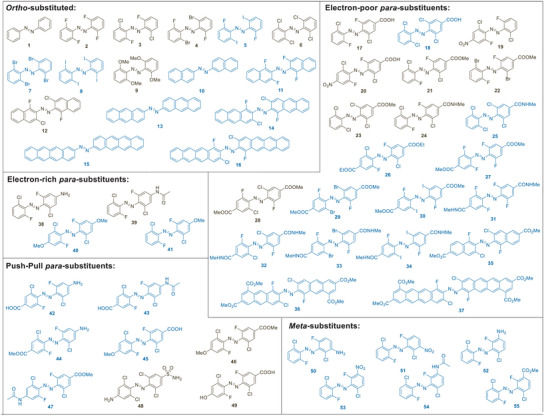
Studied azobenzene substitution patterns: computed (blue) and computed as well as synthesized (camel).

In order to rationalize the experimental results and to allow for the design of new highly red‐shifted azobenzenes by predicting the impact of alternate substitutions on the photophysical properties, we used our data to establish and validate a computational platform that consists of a combination of quantum‐chemical methodologies. Remarkably, this platform allows us to calculate the UV–vis traces of the n → π* transitions, including the separation between the isomers and the extent of the excitation band tails. The term “extent of the excitation band tails” refers to the point that the absorption of the compound nears zero and is thus too low to allow productive photoswitching with the respective wavelength of light. We computed 55 distinct photoswitches and selected 20 candidates, which were synthesized as well as characterized (Figure 1). Our overall library was categorized into purely *ortho*‐substituted (**1**–**16**) and combinations between *ortho*‐ and *para‐*modifications (**17**–**55**). The latter was split into four categories: *ortho*‐halogenated azobenzenes that contain electron‐poor *para*‐substituents (**17**–**37**), electron‐rich *para*‐substituents (**38**–**41**), push–pull *para*‐substituents (**42**–**49**) and diverse *meta*‐substituents (**50**–**55**). The azobenzene cores were synthesized via the formation of diazonium salts followed by a nucleophilic addition (**9**, **46**, and **49**), oxidative aniline dimerization (**2**, **3**, **4**, and **12**), or through a Bayer–Mills reaction (**17**, **18**, **19**, **20**, **21**, **22**, **23**, **24**, **28**, **38**, **39**, and **48**) (cf. Supporting Information I for details). We found that with an increasing density of *ortho*‐substituents on the aniline and/or nitrosobenzene reaction components, the Bayer–Mills reaction becomes less likely to give the desired product. As such, a di‐*ortho*‐fluoro azobenzene core was established in the synthesis of **3**, **4**, **12**, **17**, **19**, **20**, **21**, **22**, **24**, **28**, **38**, and **39** followed by late‐stage chlorination or bromination. Except for the compounds **1**, **2**, **4**, **9**, and **22**, the chlorine atoms were introduced through a palladium‐catalyzed C–H di‐*ortho*‐chlorination or tetra‐*ortho*‐chlorination procedure that we have previously developed (cf. Supporting Information I for synthetic details).^[^ ^25^, ^28^ ^]^ As such, our study has significantly extended the substrate scope of this procedure and we found that electron‐rich azobenzenes are not well tolerated. This feature necessitated to synthesize amine‐substituted derivatives, such as **38**, **39**, and **48**, by chlorinating the corresponding nitro derivatives **19** and **64**, respectively, and performing a reduction. The reduction of a nitro group in the presence of a diazene unit, however, proved challenging and the use of Na_2_S was found to be uniquely suited to this task, albeit each substrate required the optimization of the temperature to avoid the cleavage of the N═N bond. For the synthesis of the **dfdb** azobenzene derivatives **4** and **22**, we identified that two C–H bromination methods were suitable^[^ ^30^, ^31^ ^]^ but, although excess of the bromination reagent was used, a mono‐bromination occurred, which required to iteratively resubject the substrate to suitable conditions. An electron‐rich phenol ring is present in the push–pull azobenzene **48**, which is a precursor to **46**. This feature excluded the use of the C–H chlorination procedure while employing starting materials with two *ortho*‐substituents was not tolerated by the Bayer–Mills reaction. To circumvent these difficulties, we demonstrated that the formation of diazonium salts followed by a nucleophilic addition is excellently suited to synthesize the push–pull scaffold **48**. To support the characterization of the chemical structures, X‐ray analyses were performed with the *cis*‐configuration of **tof** (**2**, CCDC 2387092), **toc** (**6**, CCDC 2387095) as well as carboxymethyl **dfdfc** (**21**, CCDC 2387093) and the *trans*‐configuration of nitro **dfdc** (**19**, CCDC 2387091) as well as the symmetrical carboxymethyl **dfdc** (**28**, CCDC 2387094) azobenzene (cf. Supporting Information II, Chapter 8).^[^ ^32^ ^]^ Next, we experimentally determined the photophysical properties of the azobenzene derivatives and compared our data to the computational results (cf. Supporting Information III).

## Results and Discussion

### Photophysical Study

In general, the extent and localization of the n → π* excitation bands on the azobenzene derivatives were visualized through UV–vis analyses and verified through photoswitching by illuminating the dark‐adapted compounds with light. The exact *cis*/*trans* ratios obtained with specific wavelengths were determined through NMR analysis. To ensure that the UV–vis results are comparable to the NMR data, we performed both experiments using a 500 µM concentration of the azobenzenes in a 9:1 DMSO/H_2_O or DMSO‐*d*_6_/D_2_O mixture. Depending on the thermal stability, the relaxation half‐lives of the *cis*‐isomer were determined through UV–vis or NMR analysis. To assess their potential utility for in vivo applications, we investigated the stability of the azobenzene derivatives in presence of GSH in an 8:2 DMSO/PBS pH 7.4 mixture by UV–vis analysis at 37 °C.

#### Ortho‐Substituted Azobenzenes

To enable a direct comparison between new and literature‐known azobenzenes, we examined the **tof** (**2**), **toc** (**6**), and tetra‐*ortho*‐methoxy (**tom**) (**9**) substitutions as well as **dfdc** (**3**), our lead modification, using our setup.^[^ ^21^, ^22^, ^25^, ^26^ ^]^ We performed irradiation experiments with affordable, commercially available UV (365 nm, 0.11 mW mm^−2^), blue (450 nm, 0.53 mW mm^−2^), green (525 nm, 0.52 mW mm^−2^), red (650 nm, 1.12 mW mm^−2^), and near‐infrared (near‐IR) (740 nm, 1.18 mW mm^−2^) light‐emitting diodes (LEDs) to ensure the reproducibility of our results beyond our laboratory and the photoswitch community. Due to the broad beam angle of the LEDs, however, the irradiation interfered with the simultaneous measurement of an UV–vis spectra. Considering the high thermal stability of most azobenzene derivatives within our library, pausing the irradiation for the duration of a measurement (∼10 s) did not lead to a deviation in the UV–vis spectrum. However, for compounds with very short thermal half‐lives, such as the push–pull azobenzenes **48** and **49** as well as the nitro‐*para*‐substituted azobenzene **19**, we used a more elaborate irradiation set‐up, namely the pE‐4000, with a focused optical beam of its built‐in LEDs to yield intensities of 50.1 mW mm^−2^ for 660 nm, 9.95 mW mm^−2^ for 525 nm, 50.3 mW mm^−2^ for 460 nm, and 50.4 mW mm^−2^ for 365 nm.^[^ ^33^ ^]^

Except for azobenzene (**1**) and **tof** azobenzene (**2**), which do not show photoswitching with deep red light,^[^ ^25^ ^]^ the highest photoconversion to the *cis*‐isomer for the literature‐known *ortho*‐substituted derivatives was obtained by irradiation with 650 nm (**3**: *trans*:*cis* = 8:92 after 120 min; **6**: *trans*:*cis* = 18:82 after 30 min; **9**: *trans*:*cis* = 10:90 after 60 min). With increasing wavelengths, the absorption of the azobenzenes is decreasing, which leads to higher irradiation times and impacts the overall *trans*:*cis* ratio. To assess the extent of the n → π* band tail, the 740 nm near‐IR LED was applied to the dark‐adapted compound, which produced a *trans*:*cis* ratio of 72:28 after 1 d and 42:58 after 4 d for **3**, 71:29 after 1 d, and 15:85 after 4 d for **tom** azobenzene (**9**) as well as 79:21 after 1 d and 22:78 after 4 d for **toc** azobenzene (**6**). Azobenzene (**1**) demonstrated the lowest molar absorption coefficient ε at the respective *λ*_max_ (n → π) for the *trans* isomer and PSS with the highest *cis*‐content followed by **dfdb** (**4**), **toc** (**6**), **dfdc** (**3**), and **tof** (**2**) with **tom** (**9**) showing the highest absorbance (cf. Table 1). In contrast to azobenzene (**1**: *t*_1/2_ = 3.16 h at 55 °C) and **toc** azobenzene (**6**: *t*_1/2_ = 9 h at 55 °C), the thermal relaxation of the *cis‐*isomer is slowed to 3.97 h at 90 °C for tetra‐*ortho*‐fluoro (**2**), 3.7 h at 90 °C for **dfdc** azobenzene (**3**), and 3.27 h at 90 °C for **tom** azobenzene (**9**).

**Table 1:** *λ*_max_ (n → π*), the molar absorption coefficient *ε* at *λ*_max_ (n → π*) and half‐life *t*_1/2_ of the *cis*‐isomer from experimental and computational data.

	Experimental						Computational		
	*λ*_max_ (nm)		*ε* [$\frac{1}{\frac{mmol\cdot m}{L}}$]		*t*_1/2_ (dark) *cis* → *trans*		*λ*_max_ (nm)		*t*_1/2_ *cis* → *trans*
No.	*trans* (100%)	*cis* (PSS)	*trans* (100%)	*cis* (PSS)	Measured	Extrapolated (rt)	*trans* (100%)	*cis* (100%)	(rt)
1	446	435 (64%)	0.05	0.09 (64%)	3.16 h (55 °C)	211 h	455	457	267 d
2	449	417 (92%)	0.11	0.15 (92%)	3.97 h (90 °C)	7.65 y	465	435	3760 y
3	455	426 (92%)	0.08	0.14 (92%)	3.7 h (90 °C)	5.16 y	481	439	598 y
4	461	429 (83%)	0.06	0.10 (83%)	1.82 h (90 °C)	2.16 y	485	448	241 y
6	461	442 (82%)	0.07	0.12 (82%)	9.03 h (55 °C)	31.3 d	493	461	1.41 y
9	462	429 (92%)	0.15	0.16 (92%)	3.27 h (90 °C)	12.3 y	485	444	167 000 y
12	–	434 (90%)	–	0.09 (90%)	60.1 min (70 °C)	44.4 d	485	444	1065 y
17	460	428 (95%)	0.01	0.02 (95%)	69.6 min (90 °C)	131 d	490	444	43.4 d
19	467	451^a)^	0.09	0.10^a)^	12.6 s (rt)	12.6 s	488	444	8.43 h
20	461	–	0.15	–	25.7 min (rt)	25.7 min	510	450	27.1 h
21	464	429 (100%)	0.09	0.17 (100%)	44.0 min (90 °C)	104 h	490	444	156 d
22	467	432 (89%)	0.08	0.14 (89%)	27.9 min (90 °C)	52.2 d	485	455	40.8 d
23	463	447 (87%)	0.06	0.11 (87%)	1.95 h (45 °C)	23.6 h	508	467	10.5 h
24	465	428 (99%)	0.09	0.16 (99%)	79.0 min (90 °C)	317 d	488	444	8.51 y
28	465	429 (77%)	0.04	0.06 (77%)	38.8 min (55 °C)	40.3 h	495	452	122 d
38	380^b)^, ^c)^	493 (73%)^b)^, ^d)^	1.98	0.39 (50%)	52.2 min (60 °C)	6.16 h	463	446	229 d
39	448	434 (90%)	0.12	0.18 (90%)	46.2 min (90 °C)	246 d	478	441	43.1 y
46	467	439 (93%)	0.07	0.10 (93%)	4.31 h (45 °C)	59.6 h	481	446	90.1 h
48	–	–	–	–	33.0 s (rt)	33 s	493	476	14.9 s
49	397^b)^, ^c)^	429^b)^, ^d)^	1.40	0.53^d)^	0.12 s (rt)	0.12 s	481	446	42.5 h

*λ*_max_ (n → π*) was determined from a 500 µM solution for 100% *trans* and the highest *cis*‐content, including the PSS states that were measured using NMR analysis.

^a)^ Irradiation with 525 nm light.

^b)^ *λ*_max_ was determined from a 50 µM solution.

^c)^ Irradiation with 365 nm light.

^d)^ Due to the strong overlap between the π → π* and n → π* bands in the UV–vis spectrum, these values cannot be unambiguously assigned to the n → π* band.

**Figure 2:**
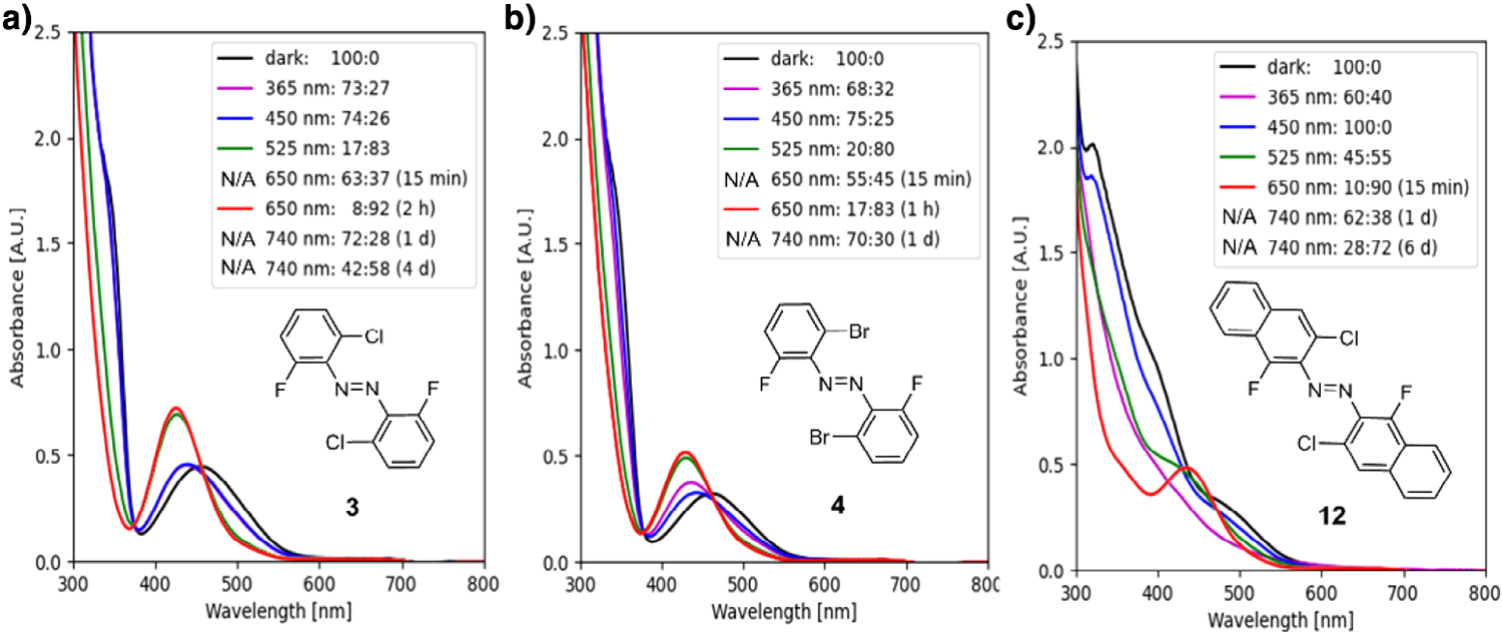
a) UV–vis spectra and NMR analysis‐derived PSS (*trans*:*cis* ratios) of a) **dfdc** azobenzene **3**, b) **dfdb** azobenzene **4**, and c) **dfdc** azonaphthalene **12** (500 µM in DMSO‐*d*_6_/D_2_O (9:1)).

First, two design strategies were investigated to further increase the red‐shifted absorption spectrum of the **dfdc** azobenzene: **1)** Substituting the two chlorine atoms for the larger bromine to generate the di‐*ortho*‐fluoro di‐*ortho*‐bromo (**dfdb**) azobenzene (**4**), which potentially increases the repulsive overlap with the nitrogen lone pairs; and **2)** increasing the extent of the π system by using naphthyl groups (**12**) in place of the phenyl moieties within the azobenzene core. UV–vis analysis shows a bathochromic shift of the absorption maximum of the n → π* band from 426 nm for the PSS with the highest *cis*‐isomer content of the **dfdc** version (**3**) to 429 nm for the *cis*‐isomer of the **dfdb** azobenzene (**4**) and 434 nm for the *cis*‐isomer of the naphthalene‐derived **12** (cf. Table 1). Both photoswitches show faster photoswitching with 650 nm red light (cf. Figure 2). Naphthyl‐derived **12** is the most promising *ortho*‐substituted photoswitch, reaching its PSS with the highest *cis*‐content after merely 15 min (*trans*:*cis* = 10:90), while the **dfdb 4** required 1 h (*trans*:*cis* = 17:83) and the **dfdc 3** required 2 h (*trans*:*cis* = 8:92). Remarkably, at the same time, the **dfdc** azonaphthalene (**12**) afforded quantitative photoswitching to the *trans*‐isomer with 450 nm blue light. To assess whether the n → π band tail was extended into the near‐IR region, 740 nm light was subjected to the dark‐adapted compound, which produced a *trans*:*cis* ratio of 62:38 after 1 d and 28:72 after 6 d for **dfdc** azonaphthalene (**12**) as well as 70:30 after 1 d for **dfdb** azobenzene (**4**). The thermal half‐life of the *cis‐*isomer of **dfdb 4** is 1.82 h at 90 °C, which is similar to **dfdc** azobenzene (**3**), while it is accelerated to *t*_1/2_ = 60.1 min at 70 °C for **dfdc** azonaphthalene (**12**).

It was discovered that the highly red‐shifted n → π absorption band tails of *ortho*‐substituted azobenzenes are a result of the molecular motion‐induced adaptation of near‐planar geometries of *trans‐*isomers that are twisted out of the plane. These transient conformational changes increase the overlap between the bulky *ortho* substituents and the n molecular orbitals, which concomitantly destabilizes the n molecular orbital and decreases the gap of the n to the π* orbitals.^[^ ^25^ ^]^ As such, we reasoned that an increase in the temperature, which increases the rate of the molecular motions, could lead to faster photoswitching. Please note that, for this experiment, the irradiation was performed in a water bath, which decreased the overall light intensity that reached the sample and thus led to longer irradiation times. Remarkably, our hypothesis was valid for the **dfdb** azobenzene (**4**), whereby an increase in the temperature shortens the irradiation time with 650 nm light needed to reach the highest *cis*‐content from 4 h at 25 °C (*trans*:*cis* = 18:82) to 2 h at 37 °C (*trans*:*cis* = 21:79) and further to 1 h at 50 °C (*trans*:*cis* = 23:77). This difference can already be observed after 15 min, where the observed *cis*‐content is 9% at 25 °C, 35% at 37 °C, and 45% at 50 °C (cf. Supporting Information II, Chapter 1). **Dfdc** azobenzene (**3**) shows no significant temperature‐dependent changes in photoswitching. After 15 min, the *cis*‐content is 25% at 25 °C, 36% at 37 °C, and 32% at 50 °C, while the irradiation time necessary to reach the PSS with the highest *cis*‐isomer level is 2 h at 25 °C (*trans*:*cis* = 13:87), 1.5 h at 37 °C (*trans*:*cis* = 20:80), and 2.5 h at 50 °C (*trans*:*cis* = 11:89). It is worth noting that this experiment could not provide a rational outcome with **toc** azobenzene (**6**), since the thermal relaxation of the *cis*‐isomer competes with photoswitching at elevated temperatures.

#### Electron‐Poor Para‐Substituents

With the exception of nitro groups, the introduction of an electron‐deficient *para*‐substituent leads to a red‐shift of the n → π* band tail. Irradiating with 650 nm, therefore, leads to a faster photoconversion to the *cis‐*isomer and using 740 nm additionally allows to reach PSS with a higher *cis‐*content than for the corresponding non‐*para‐*substituted **dfdc**, **dfdb**, and **toc** azobenzenes. In most cases, the *cis‐*isomers have a high thermal stability (cf. Supporting Information II, Chapter 5). A substitution with a nitro group, however, increases the relaxation rates (**19**: *t*_1/2_ = 12.6 s at rt). Complementing nitro **dfdc** azobenzene **19** with a carboxylic acid moiety on the opposite *para‐*position (**20**) slows the relaxation to *t*_1/2_ = 25.7 min at rt while the same compound without the nitro group, i.e., azobenzene carboxylic acid has a long‐lived *cis*‐isomer (**17**: *t*_1/2_ = 69.6 min at 90 °C). While the increased relaxation times did not allow us to analyze the exact PSS by NMR, UV–vis analysis shows that the highest *cis*‐content was reached with green and purple light. This finding highlights the ability to design **dfdc**‐based azobenzenes with relaxation rates across a broad time frame that spans multiple orders of magnitude, allowing versatile and highly tunable control for diverse applications.

**Figure 3:**
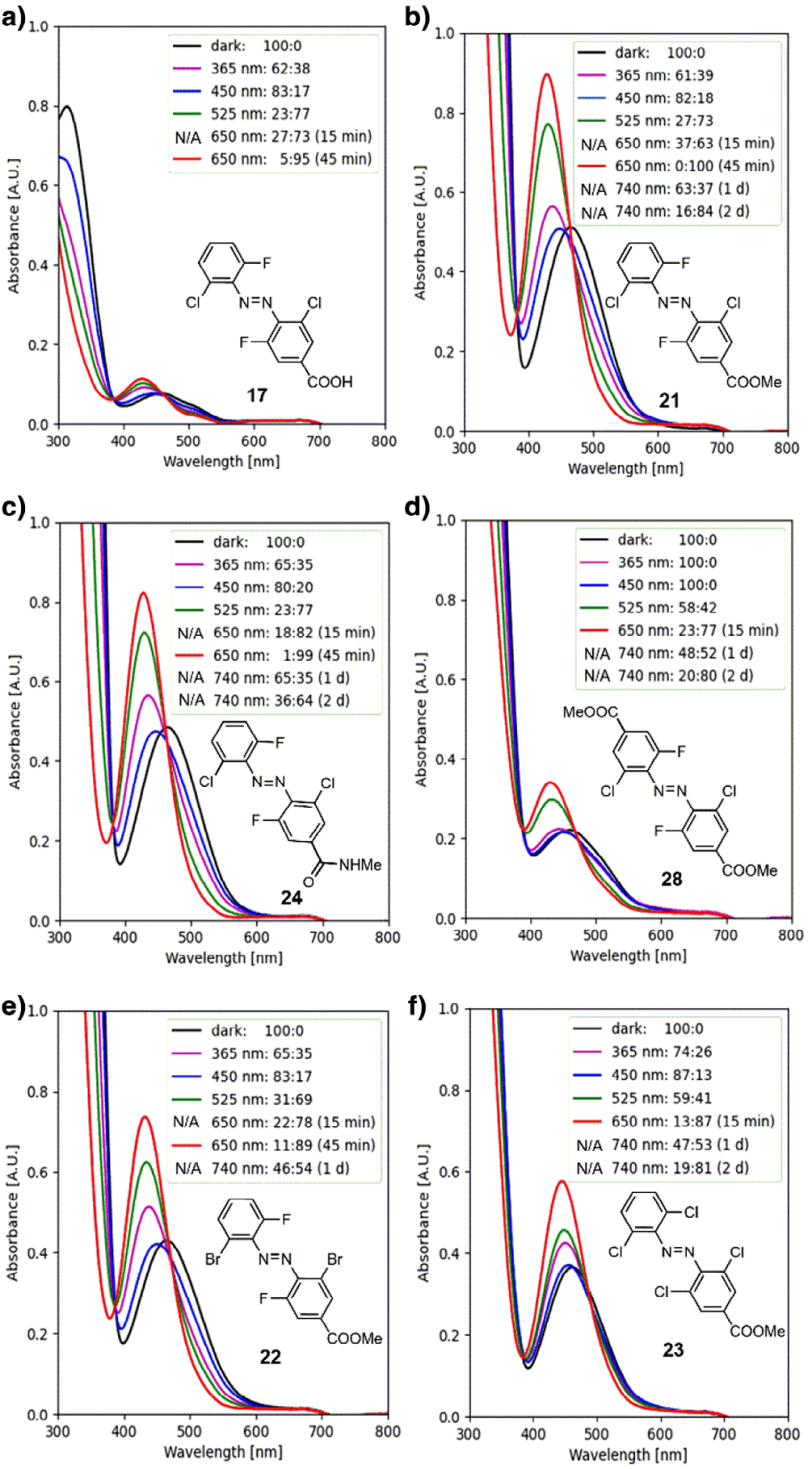
UV–vis spectra and NMR analysis‐derived PSS (*trans*:*cis* ratios) of a) **dfdc** carboxylic acid **17**, b) **dfdc** methyl ester **21**, c) **dfdc** methyl amide **24**, d) **dfdc** di‐methyl ester **28**, e) **dfdb** methyl ester **22**, and f) **toc** methyl ester **23** (500 µM in DMSO‐*d*_6_/D_2_O (9:1)).

Small changes in the electronic nature of the *para*‐substituent on **dfdc** azobenzene, such as moving from a carboxylic acid (**17**) to a methyl ester (**21**) or a methyl amide (**24**), result in a slight change in the photochemical behavior (cf. Figure 3) but are associated with a clear shift in the molar absorption coefficient *ε* (cf. Table 1). While all three derivatives show high photoconversion to the *cis*‐state after irradiation with 650 nm (*trans*:*cis* ratios for **17**: 5:95 after 45 min; **21**: 0:100 after 45 min, and **24**; 1:99 after 45 min), the photoconversion with 740 nm differs from **21** (84% *cis* after 2 d) to **24** (64% *cis* after 2 d) based on the relative electron‐withdrawing properties of the substituent. While the methyl ester (**21**) or a methyl amide (**24**) substituent compared to the nonsubstituted **dfdc** azobenzene (**3**) leads to a slight increase, the carboxylic acid substituent (**17**) is associated with a strong decrease in the absorption (cf. Table 1). In addition, **17** suffered from a low solubility in DMSO‐*d*_6_/D_2_O (9:1), which led to a precipitation out of the solution during extended measurements, hindering the determination of PSS through irradiation with 740 nm light over 2 days. All three isomers (**17**, **21**, and **24**) have long‐lived *cis*‐isomers whereby an increase of the relaxation half‐life can be observed from methyl ester (**21**, *t*_1/2_ = 44.0 min at 90 °C) to carboxylic acid (**17**, *t*_1/2_ = 69.6 min at 90 °C) and methyl amide (**24**, *t*_1/2_ = 79 min at 90 °C).

The addition of a second methyl ester at the opposite *para*‐position (**28**) leads to a faster conversion to the *cis*‐isomer (77% *cis* after 15 min) in comparison to the mono *para*‐substituted methyl ester **21** (63% *cis* after 15 min), even though the molar absorption coefficient *ε* at the respective *λ*_max_ (n → π) for the *trans* isomer and PSS with the highest *cis*‐content is strongly decreased (cf. Table 1). While illuminating with 525 nm (*trans*:*cis* = 58:42) and 650 nm (*trans*:*cis* = 23:77) shows a lower maximal fraction of the *cis*‐isomer than **21**, **28** enables quantitative photoswitching to *trans* with 365 nm and 450 nm (*trans*:*cis* = 100:0) (cf. Figure 3). To assess whether the n → π* band tail was extended into a more red‐shifted region, 740 nm light was subjected to the dark‐adapted **28**, which produced a *trans*:*cis* ratio of 48:52 after 1 d and 20:80 after 2 d. In comparison to the mono‐substituted methyl carboxylate **21**, the relaxation of the disubstituted methyl ester **28** is slightly accelerated from *t*_1/2_ = 44.0 min at 90 °C to *t*_1/2_ = 38.8 min at 55 °C.

To assess slight changes in the *ortho*‐substitution pattern toward the photophysical behavior and thereby complement the results gained from the analysis of the unsubstituted **dfdc** (**3**), **dfdb** (**4**), and **toc** (**6**) azobenzenes, we synthesized derivatives with an electron‐active methyl carboxylate substituent, namely **dfdb 22** and **toc** azobenzene **23**, to compare to the **dfdc** version **21**. All methyl ester derivatives show a faster photoconversion and lead to a higher *cis*‐content with 650 and 740 nm light than their nonsubstituted derivatives. At 650 nm, the highest *cis*‐content reached for **21**: 100% after 45 min, **22**: 89% after 45 min, and **23**: 87% after 15 min, while at 740 nm, the highest *cis*‐content reached for **21**: 81% after 2 d, **22**: 54% after 1 d, and **23**: 81% after 2 d. Even though the **dfdb** substitution pattern has the faster photoconversion, the extent of the *cis*‐isomer that can be reached is significantly lower compared to the **dfdc** substitution pattern. The carboxylate **dfdc 21** and **dfdb 22** have a slightly increased absorption compared to the purely *ortho*‐substituted derivatives **3** and **4**, respectively, whereas **toc 23** retains the molar absorptivity of **6** (cf. Table 1).

While the half‐lives of the **dfdb** methyl carboxylate **22** and corresponding **dfdc** analogue **21** lie within the same order of magnitude with *t*_1/2_ = 27.9 min at 90 °C and 44.0 min at 90 °C, respectively, the **toc** substitution pattern (**23**) has a significantly higher relaxation rate of the *cis*‐isomer with *t*_1/2_ = 1.95 h at 45 °C. These trends in the changes of the thermal half‐lives between **21**, **22**, and **23** are reflected in the *ortho*‐substituted azobenzenes **3**, **4**, and **6** with hydrogen atoms at both the *para*‐positions.

#### Electron‐Rich Para‐Substituents

**Figure 4:**
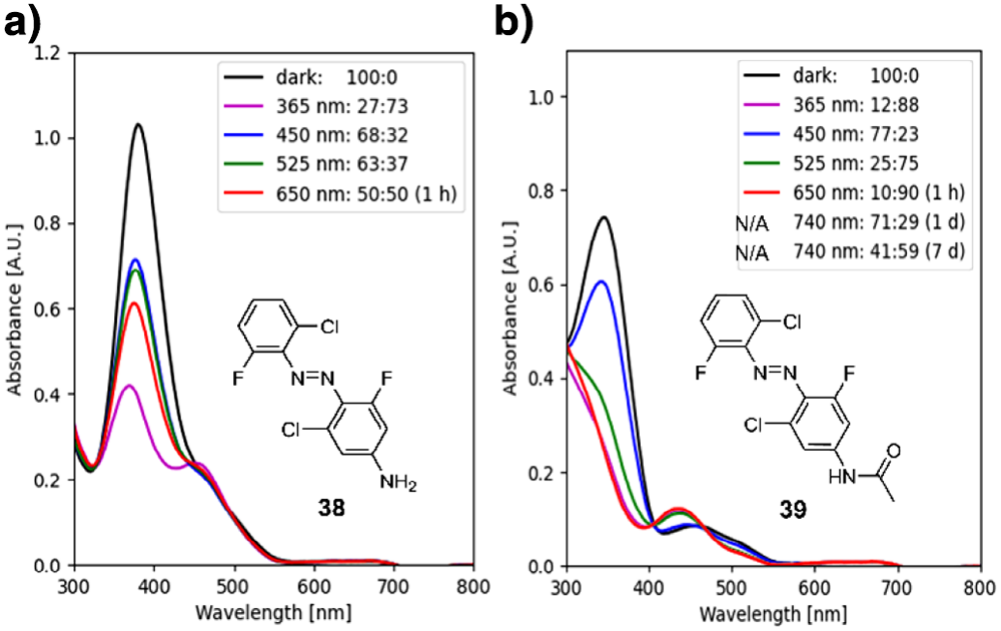
UV–vis spectra (50 µM in DMSO/H_2_O (9:1)) and NMR analysis‐derived (500 µM in DMSO‐*d*_6_/D_2_O (9:1)) PSS (*trans*:*cis* ratios) of a) **dfdc** amine **38** and b) **dfdc** amide **39**.

In general, the introduction of an amine or acetamide substituent impedes the photoconversion with near‐IR light at 740 nm. While irradiating amide **39** (highest *cis*‐content is 90% after 1 h) with 650 nm produces similar results to the standard **dfdc** azobenzene (**3**), it requires a prolonged illumination of 7 d to reach the highest level of the *cis*‐isomer of 59% with 740 nm as opposed to **dfdc** azobenzene **3** of 52% after 4 d. These results are in line with the fact that, albeit we have added the acetamide substituent (**39**) to the electron‐donating category, its Hammett substituent constant *σ*_P_ is 0.00, which establishes a close relationship to the hydrogen‐substituted **3**.^[^ ^34^ ^]^ The amine **38** shows no photoconversion with 740 nm and reaches a PSS with a maximal *cis* content of 49% with 650 nm, which requires an illumination time of 60 min (cf. Figure 4). Irradiation with 365 nm light produces the PSS with the highest *cis*‐isomer content of 73% for the electron‐rich **38**. The molar absorption coefficient *ε* of amine **38** is significantly higher than that of amide **39**. The absorbance of the latter (**39**) is marginally increased in comparison to **dfdc** azobenzene **3** (cf. Table 1). The half‐life of *cis*‐**38** is decreased to *t*_1/2_ = 52.2 min at 60 °C compared to *cis*‐**39**, which is near‐bistable with *t*_1/2_ = 46.2 min at 90 °C.

#### Push–Pull Para‐Substituents

The introduction of an electron‐donating hydroxy and an electron‐withdrawing carboxylic acid substituent at opposite *para*‐positions established the **dfdc** azobenzene **49** and a di‐methylation thereof yielded **46** with a strong and moderate push–pull effect, respectively. Compared to the electron‐poor **dfdc** methyl ester **21**, the installation of an additional methoxy group in *para*‐position impedes its photoconversion with 740 nm and accelerates the photoconversion with 650 nm to a *trans:cis* ratio of 7:93 after 15 min. At the same time, the thermal stability of the *cis*‐isomer is decreased to a half‐life of *t*_1/2_ = 4.31 h at 45 °C.

**Figure 5:**
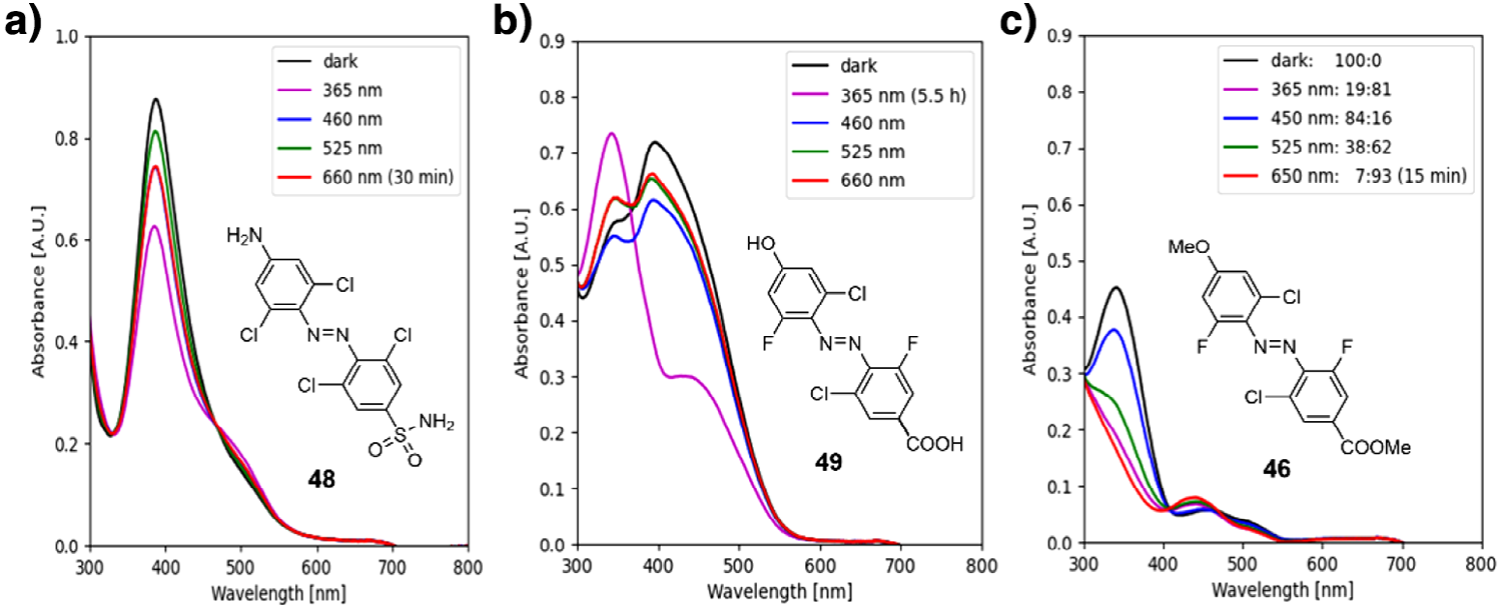
UV–vis spectra (50 µM in DMSO/H_2_O (9:1)) and NMR analysis‐derived (500 µM in DMSO‐*d*_6_/H_2_O (9:1)) PSS (*trans*:*cis* ratios) of a) **toc 48**, b) **dfdc 49**, and c) **dfdc 46** (50 µM in DMSO/H_2_O (9:1)).

Since no **toc**‐derived azobenzene that hosts an electron‐donating and an electron‐withdrawing substituent on opposite *para‐*positions has been included in the key study that established general principles for the design of **toc** azobenzenes,^[^ ^27^ ^]^ we decided to additionally prepare **48**. The strong push–pull characteristics of **toc**‐derived **48** and **dfdc**‐derived **49** lead to very low thermal stabilities with *t*_1/2_ = 33 s for *cis*‐**48** and *t*_1/2_ = 0.12 s for *cis*‐**49** at room temperature. These fast relaxation rates did not allow to determine a PSS through NMR analysis. UV–vis analysis of **toc**‐azobenzene **48** did not show significant photoconversion, while **dfdc** azobenzene **49** showed significant photoconversion with 365 nm after an extended irradiation time of 5.5 h (cf. Figure 5). Due to a strong overlap between the n → π* and π → π* absorption bands, the transitions could not be clearly distinguished from each other for the azobenzene derivative **49** and for the *trans*‐isomer of **48** (cf. Figure 5). While the molar absorption coefficient *ε*, compared to **dfdc** azobenzene **3** is decreased for **46** with a moderate push–pull effect, the stronger electron‐donating and ‐withdrawing groups on **48** lead to a strongly increased absorbance (cf. Table 1).

#### Quantum Yields

We further investigated the isomerization process by determining the quantum yields (*Φ*_E→Z_, 650 nm), which are derived from UV–vis and NMR measurements (cf. Supporting Information II, Chapter 7).^[^ ^23^ ^]^ In short, the *trans* → *cis* isomerization is induced by irradiation at 650 nm, a wavelength characterized by generally low absorbance for azobenzenes, and the absorption is measured at the *λ*_max_ (n → π*) of the PSS with the highest possible *cis*‐content (see Table 2 for the specific *λ*_max_ values) at regular intervals along with the corresponding *trans*:*cis* ratios via NMR analysis. The quantum yields were found to be in the range of 0.001–0.011 (cf. Table 2). For the four investigated *ortho*‐substitution patterns **dfdc** (**3**), **dfdb** (**4**), **toc** (**6**), and **tom** (**9**), we observe the highest value for **toc** (**6**) with *Φ*_E→Z_, 650 nm = 0.00446 followed by **dfdb** (**4**) with *Φ*_E→Z_, 650 nm = 0.00299. The introduction of a *para*‐substituent increases the quantum yield with the electron‐poor methyl ester **21** (*Φ*_E→Z_, 650 nm = 0.00395) having a stronger influence than the electron‐rich amide **39** (*Φ*_E→Z_, 650 nm = 0.00215). The greatest quantum yield was found for the push–pull azobenzene **46** (*Φ*_E→Z_, 650 nm = 0.0114), which is reflected in the short irradiation time required to generate a *trans:cis* ratio of 7:93.

**Table 2:** Quantum yields of the *trans* → *cis* isomerization (*Φ*_E→Z_) measured in DMSO/H_2_O (9:1) while irradiating with 650 nm and measuring the absorbance at *λ*_max_ (*cis*).

Molecule	*λ*_max_ (nm)	Quantum yield (*Φ*_E→Z_ at 650 nm)
3	426 (92% cis)	0.00126
4	429 (83% cis)	0.00299
6	442 (82% cis)	0.00446
9	429 (92% cis)	0.00222
21	429 (100% cis)	0.00395
39	434 (90% cis)	0.00215
46	439 (93% cis)	0.0114

#### Stability Toward Glutathione

The ability of GSH to reduce and thereby inactivate the photoswitching ability of azobenzenes impedes their use in vivo.^[^ ^29^ ^]^ As such, we investigated the stability of our azobenzene library in the presence of GSH. To a solution of 50 µM azobenzene in an 8:2 mixture of DMSO and phosphate‐buffered saline (PBS) at pH 7.4 was subjected to 10 mM GSH. All samples were studied in their dark‐adapted (*trans*) and illuminated (PSS were selected with a high *cis* content) state to assess differences in their stability toward GSH. We discovered that illumination with 365 nm leads to a decomposition of GSH itself with *t*_1/2_ = 2.75 h at 37 °C (cf. Supporting Information II, Chapter 4).

Sulfasazaline is an azobenzene‐based small molecule prodrug that is not metabolically stable within the body. In fact, its mechanism of action relies on the reduction of the diazene group in vivo, which generates the active drug.^[^ ^35^, ^36^ ^]^ Prior studies described *para*‐substituted derivatives of the red‐shifted **tom** azobenzene (**9**) with, e.g., amine and amide groups, that are unstable in the presence of GSH.^[^ ^21^, ^22^, ^37^ ^]^ Since **tom** azobenzene (**9**) itself is stable in our assay, we reason that *para‐*substituents can impede the stability of certain azobenzene scaffolds. As such, we have collectively assessed the stability of all synthesized compounds toward GSH.

**Figure 6:**
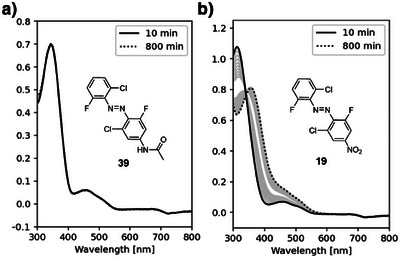
Stability against GSH of a) the stable **39** and b) the unstable **19** (50 µM in 8:2 DMSO/PBS pH 7.4 with 10 mM GSH).

Apart from the derivatives that hosted a nitro substituent, all investigated tetra‐*ortho‐*halogenated azobenzenes with varying functional groups in *para*‐position remained stable. The nitro‐substituted *trans‐* **19** (cf. Figure 6) had a half‐life of 112 min in the presence of GSH. For the *cis*‐isomer, a two‐step process was observed with half‐lives of $t_{1/2}^{a}$ = 20 min and $t_{1/2}^{b}$ = 160 min. The addition of an acid group on the opposite *para*‐position yielded azobenzene **20** with a shortened half‐life of *t*_1/2_ = 69 min for both isomers.

### Computational Study

In order to augment and understand the experimental findings, a comprehensive quantum‐mechanical investigation was carried out for each compound in both their *cis‐* and *trans‐*configuration. We decided to use the PBEh‐3c^[^ ^38^ ^]^ composite scheme, which provides a consistent description of bonding and nonbonding interactions, molecular geometries, and excitation energies for a reasonable computational cost. This relatively modest computational cost, coupled with our recent advancements regarding highly efficient evaluation of hybrid‐density functional theory implemented in the FermiONs++ program,^[^ ^39^, ^40^, ^41^, ^42^, ^43^, ^44^ ^]^ allows us to go beyond minimum energy geometries. We optimized the geometry of all conformers and all possible transition structures regarding the thermal *cis*→*trans* relaxation, providing reaction energies and barriers using the most stable conformers (cf. Table 3). These computed barriers correspond to the available experimental free energy barriers with a relative error (trends between different compounds) of approximately 2 kcal mol^−1^ (cf. Supporting Information III).

**Table 3:** Computed (Comp.) reaction energies and barriers (energy differences of most stable conformers corrected for zero‐point vibrational energies; TS – *cis* for barriers; *trans – cis* for energies) as well as experimental (Exp.) free energy barriers for thermal *cis*→*trans* back‐relaxation.

	*cis*→*trans* reaction energy (kcal mol^−1^)	*cis*→*trans* reaction barrier (kcal mol^−1^)	
Molecule	Comp.	Exp.	Comp.
1	−11.5	25.6	27.2
2	−6.3	28.6	32.2
3	−5.2	28.5	31.1
4	−5.2	28.0	30.6
6	−5.4	26.3	27.6
9	−2.9	28.4	34.4
12	−5.1	26.0	31.5
17	−5.0	27.7	26.2
19	−5.1	19.2	24.4
20	−5.6	22.0	24.1
21	−5.0	27.4	26.9
22	−5.1	27.0	26.1
23	−5.4	24.5	23.5
24	−5.0	27.8	28.7
28	−5.7	24.6	27.9
38	−7.3	23.3	27.2
39	−6.0	27.4	29.6
46	−6.1	25.0	24.8
48	−7.4	19.7	18.9
49	−6.2	16.4	24.3

Our previous results highlighted that the excitation wavelengths of *ortho*‐substituted azobenzenes are dependent on molecular motion‐dependent conformational flexibility.^[^ ^25^ ^]^ As such, we performed ab initio MD simulations^[^ ^45^ ^]^ for each of the 55 azobenzene derivatives, which then allowed us to compute more realistic UV–vis spectra using the whole ensemble of accessible structures obtained by the MD stimulation. By assembling a full spectrum out of 6000 individual TD‐DFT excited‐state^[^ ^46^ ^]^ calculations per compound, we take the high structural flexibility of the investigated *ortho*‐substituted azobenzenes into account, particularly the nearly free rotation around both C─N bonds caused by the bulky *ortho*‐substituents. Finally, in order to quantify the effect of this structural flexibility on the UV–vis spectra, we computed Pearson correlation coefficients between structural features and photo‐properties. More technical details and the comprehensive data for each molecule are provided in Supporting Information III.

**Figure 7:**
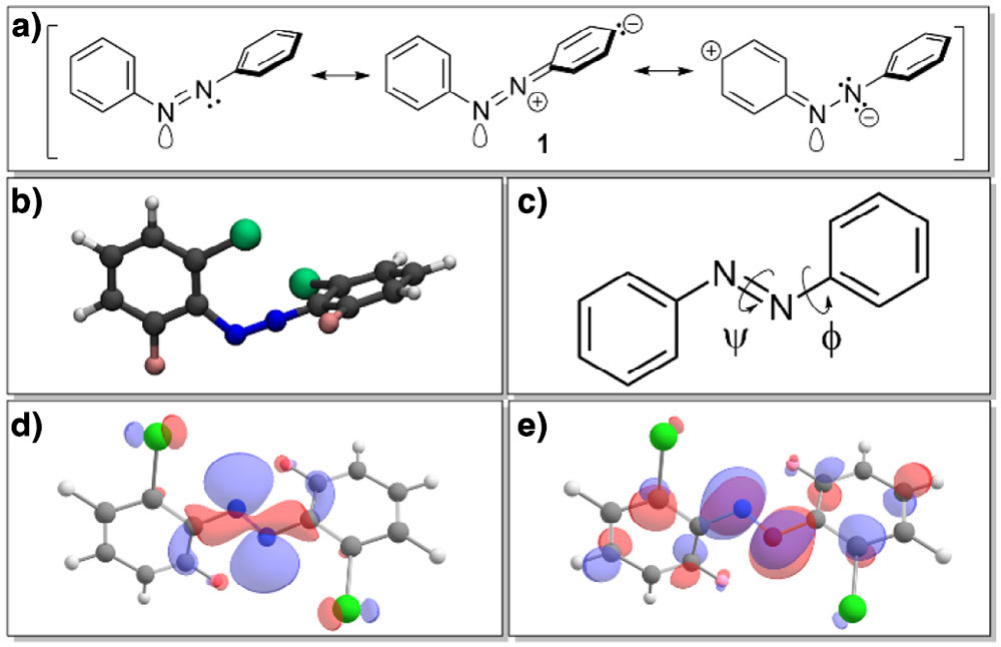
a) Resonance structures for the transition state of the thermal relaxation of **1**. b) Optimized structure for the transition state of the thermal relaxation of **3**. c) Definition of dihedral angles *ψ* and *ϕ*. d) Occupied NTO for n → π* excitation of **dfdc** azobenzene (**3**). e) virtual NTO for n → π* excitation of **dfdc** azobenzene (**3**).

**Figure 8:**
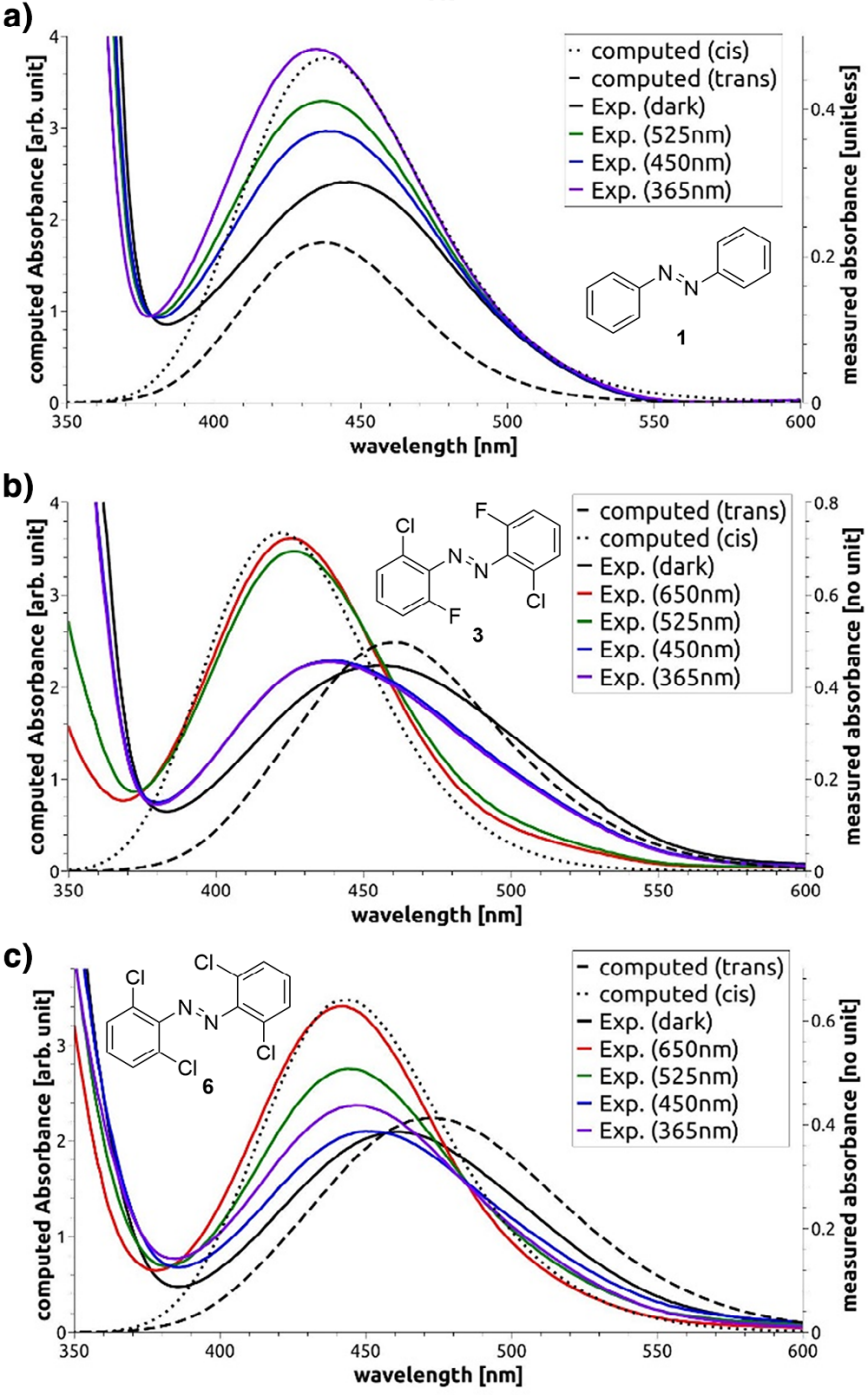
Comparison of computational and experimental UV–vis spectra of a) azobenzene (**1**), b) **dfdc** azobenzene (**2**), and c) **toc** azobenzene (**6**).

**Figure 9:**
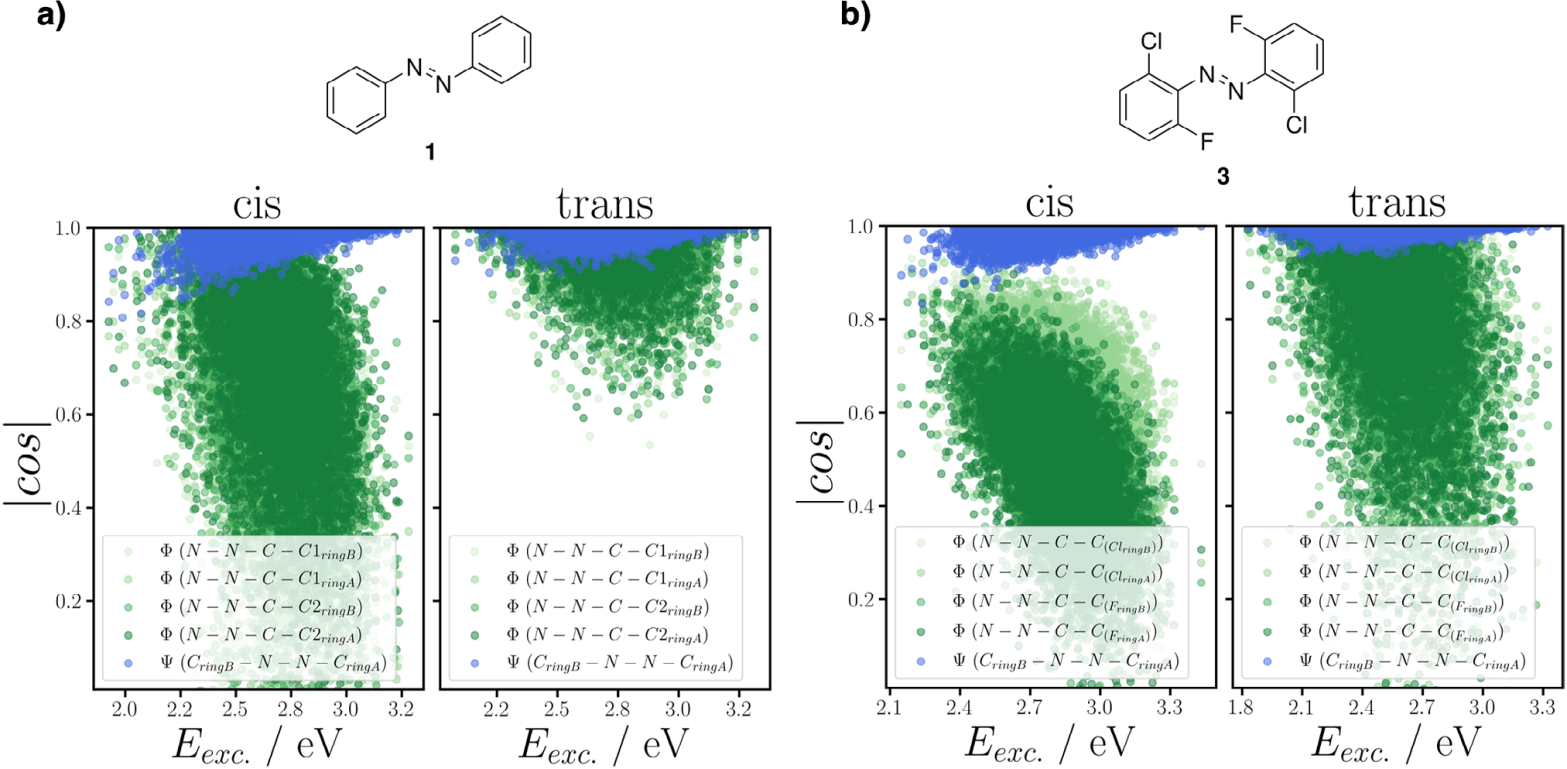
Correlation plots for *cis*‐ (left) and *trans*‐conformers (right) of a) azobenzene (**1**) and b) **dfdc** azobenzene (**3**) showing the relationship between excitation energy (*X*‐axis) and the cosine‐transformed dihedral angles (|cos| = 1 represents planar geometries; |cos| = 0 represent perpendicular geometries) in azobenzenes (*Y*‐axis). Green points represents the dihedral angles *ϕ*, measuring the rotation around the C─N bond, while blue points correspond to the central dihedral angle *ψ*, measuring the rotation around the N═N bond (see Figure 7c for angle definitions).

**Figure 10:**
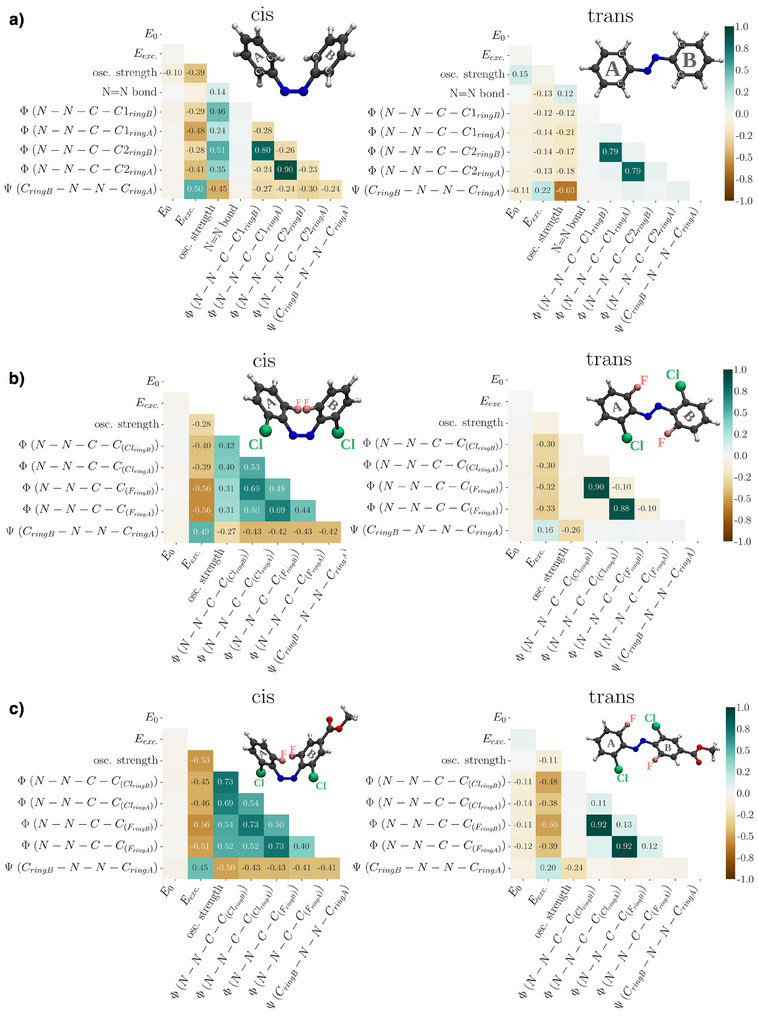
Heatmaps for *cis*‐ (left) and *trans*‐conformers (right) of a) azobenzene (**1**), b) **dfdc** azobenzene (**3**), and c) **dfdc** azobenzene with a methyl ester substitution in *para*‐position (**21**). The heatmaps show Pearson correlation coefficients between pairs of variables in the dataset. The color scale represents the strength and direction of the correlations: values close to +1 (darker brown tones) indicate strong positive correlations, values close to −1 (darker turquoise tones) indicate strong negative correlations, and values near 0 (light cream tones to white) reflect little to no correlation. Each cell corresponds to the correlation coefficient computed from 6000 thermally accessible structures that were generated through MD simulations.

While significant effort has been dedicated to the development of machine learning potentials and their application to azobenzenes, we opted against a blind, large‐scale exploration.^[^ ^47^ ^]^ Instead, it is our goal to build a “human”—not “machine”—understanding of azobenzene photochemistry and illuminate the systematic effects of various substitution patterns. This understanding provides guidance for the targeted design of biologically active photoswitches. As such, the computational study reveals a number of noteworthy trends and insights:

1. The mechanism for the thermal back‐relaxation can best be understood by considering the computed structures depicted in Figure 7a,b whereby the transition state features a linear C─N─N bond, and the two aryl rings are configured perpendicular to each other. This transition state is stabilized by negative charges on one side and positive charges on the opposite side. This rationalizes the shorter half‐life times of *para*‐substituted derivatives and, particularly, the push–pull variants **42**–**49** (cf. Table 1). Since this stabilization is only possible with *para*‐substituents, the *meta*‐substitution in compounds **50**–**55** has only little impact on thermal stability.
2. Likewise, the excitation energy of the n → π* band is only marginally impacted by *meta*‐substitution, instead being much stronger influenced by *ortho‐* and *para*‐substituents. In particular, bulky electron‐active *ortho*‐substituents destabilize the occupied n orbital, mostly comprised of the lone pairs of the diazene nitrogens, via Pauli repulsion (cf. Figure 7d,e) This effect, however, only applies to the *trans*‐isomer, where the lone pairs reside close to the *ortho*‐substituents. This explains the observed ∼50 nm *cis*‐*trans* split in the maximum of the n → π* band for all investigated *ortho*‐substituted azobenzenes. Considering the bathochromic shift of the n → π* bands with electron‐poor *para*‐substituents, it is best explained by their stabilizing effect on the π^*^ orbital through a delocalization in the π system. This delocalization is not possible with *meta*‐substituents due to its low probability density caused by the node in *meta*‐position, rationalizing the generally small impact of *meta*‐substitution.
3. Overlaying the experimental UV–vis spectra with computed spectra (cf. Figure 8), systematically shifted to account for systematic errors, shows remarkably good matching (for more details, see Supporting Information III). Please note in Figure 8 that the highest level of the *cis*‐isomer is reached with 365 nm for standard azobenzene (**1**) and 650 nm for **dfdc** (**3**) as well as **toc** azobenzene (**6**) while the highest level of the *trans*‐isomer is reached through thermal relaxation in the dark. In particular, the *cis*/*trans* separation matches well between computed and experimental spectra. Since our methodology takes structural flexibility into account, we were able to predict realistic line widths. Especially the broadened peak of **toc** azobenzene (**6**) matches quite accurately between theory and experiments.
4. In addition to naturally broad spectra, our sampling‐based approach provides detailed insights into the impact of specific structural parameters on the UV–vis spectra. The dihedral angles *ψ* (rotation around N─N bond) and *ϕ* (rotation around C─N bond, Figure 7c) are of particular interest due to their influence on the photoswitching behavior.^[^ ^25^ ^]^ The correlations between those parameters and the excitation energy of the n → π* transition are provided in Figure 9. The high flexibility with respect to the C─N‐bond rotation (*ϕ*) due to the introduction of bulky *ortho* substituents that twist the planar conformation of *trans*‐azobenzene (*trans*‐**1**) (cf. Figure 9a) is clearly visible when comparing with *trans*‐**3** (cf. Figure 9b), where the latter spans a much larger space of accessible structures.
5. Pearson correlation plots (cf. Figure 10) reveal a strong correlation between *ϕ* and the excitation energy. In particular, in‐plane configurations (cos(*ϕ*) = 0) of aryl rings with respect to the C─N─N plane lead to a reduction of the excitation energy (red‐shift), which may be explained by the delocalization of the π^*^ orbital into the aryl rings, which requires a planar configuration. In addition, this effect is significantly stronger (higher correlation coefficient in Figure 10c) where the aryl ring is substituted with an electron‐poor group in *para*‐position, as compared to azobenzene (**1**) (cf. Figure 10a) and dfdc (**3**) (cf. Figure 10b). This is due to the resonance effect of the electron‐pulling substituent in **21** requiring conjugation of the π systems to be effective, which is only realized in planar geometries.

### Biological Study

**Figure 11:**
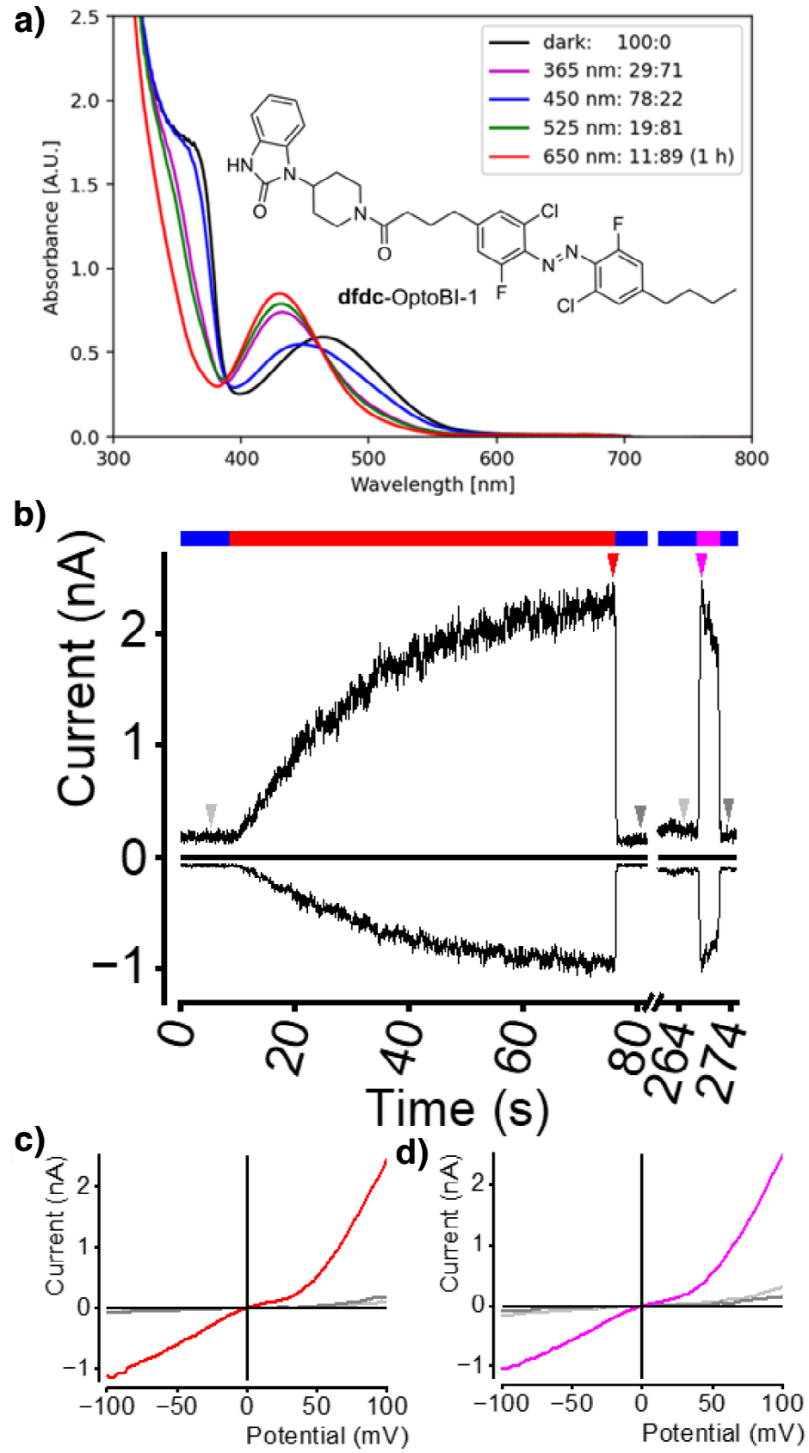
PSS of **dfdc**‐OptoBI‐1 and electrophysiological whole‐cell measurement in HEK293T cells overexpressing TRPC6. a) UV–vis spectra (500 µM in DMSO/H_2_O (9:1)) and NMR analysis‐derived (500 µM in DMSO‐*d*_6_/D_2_O (9:1)) structure and PSS (*trans*:*cis* ratios) of **dfdc**‐OptoBI‐1. b) Representative current‐time course at holding potentials of ±100 mV in the presence of 10 µM **dfdc**‐OptoBI‐1. Light stimulation was performed by illumination with LEDs. Blue bars represent illumination with 440 nm, red bars with 623 nm, and magenta bars with 365 nm. The half‐life time constants for the activation and deactivation kinetics are *τ*_1/2_ = 1354 ms for activation with red light, *τ*_1/2_ = 58 ms for activation with UV light and *τ*_1/2_ = 55 ms for first and *τ*_1/2_ = 88 ms for the second deactivation with blue light at +100 mV. At −100 mV, the half‐life time constants are *τ*_1/2_ = 1381 ms for activation with red light, *τ*_1/2_ = 56 ms for activation with UV light and *τ*_1/2_ = 74 ms for first and *τ*_1/2_ = 85 ms for second deactivation with blue light. Arrows show time points of selected current–voltage relationships. c) and d) Corresponding current–voltage relationships.

To assess whether **dfdc** azobenzenes are suitable for the development of optical tools, such as photopharmaceuticals, we synthesized **dfdc**‐OptoBI‐1—a photoswitchable derivative of OptoBI‐1, a known activator of the transient receptor potential canonical (TRPC) channels TRPC3 and TRPC6.^[^ ^48^, ^49^ ^]^ Using our standard methods to determine the PSS showed that **dfdc**‐OptoBI‐1 behaves similarly to nonsubstituted **dfdc** azobenzene **3**. The highest fraction of the *cis*‐isomer is reached by irradiation with 650 nm (*trans*:*cis* = 11:89) followed by green (*trans*:*cis* = 19:81) and UV light (*trans*:*cis* = 29:71) while blue light produces high levels of the *trans*‐isomer (*trans*:*cis* = 78:22) (cf. Figure 11a). Thermal relaxation of *cis*‐**dfdc**‐OptoBI‐1 proceeds with a half‐life of 409.7 min at 60 °C. To study the biological activity of **dfdc**‐OptoBI‐1, we performed whole‐cell patch‐clamp recordings on HEK293T cells overexpressing TRPC6 (cf. Figure 11b–d). Photoswitching was induced using LED illumination. In the presence of 10 µM **dfdc**‐OptoBI‐1, switching from blue light (440 nm) to red light (623 nm) resulted in a gradual increase or decrease in TRPC6‐mediated currents at +100 and −100 mV, respectively, indicating that red light indeed activates the TRPC6 channel (cf. Figure 11b). Switching back to blue light led to a rapid return to baseline current levels, demonstrating that the photoswitching process is reversible. Additionally, illumination with UV light (365 nm) caused a rapid activation of TRPC6 currents. Subsequent switching from UV to blue light again resulted in fast deactivation of the currents. Both red and UV light induced comparable current amplitudes and current–voltage relationships (cf. Figure 11c,d), suggesting that both wavelengths activate a similar fraction of channels and result in a comparable channel open probability. Kinetic analysis revealed that red light‐induced TRPC6 activation proceeds with a half‐life time constant of *τ*_1/2 _= 1381 ms while UV light‐induced activation proceeds with *τ*_1/2_ = 56 ms. Please note that the photophysical characterization of the azobenzene derivatives was performed with affordable, commercially available LEDs to ensure the reproducibility of our results beyond our laboratory and the photoswitch community, which showed that full isomerization of **dfdc**‐OptoBI‐1 with 650 nm was achieved after 1 h. In contrast, using high‐powered red light LEDs in biological experiments, remarkably, enables full activation of the TRPC6 channel within ∼70 s. This suggests that red light stimulation could be used together with in vivo models to study channel activity in medical contexts.

## Conclusion

To harness *ortho*‐substituted azobenzenes for the development of red light‐responsive optical devices, we have established synthetic protocols for derivatives of the **dfdc** azobenzene scaffold with various chemical alterations in the *para*‐/*ortho*‐positions. These scaffolds were subjected to an in‐depth analysis using a combination of quantum‐mechanical, NMR, and UV–vis studies to investigate the influence of the azobenzene substitution patterns toward their photophysical properties. To eliminate one of the key obstacles for the use of the synthesized photoswitches in vivo, we have assessed their stability toward GSH, which is known to chemically reduce and thereby inactivate the photoswitching ability of azobenzenes. Remarkably, we found that, apart from nitro‐substituted derivatives, all synthesized compounds were stable to this reducing agent.

To account for the conformational flexibility of the *ortho*‐substituted azobenzenes, we used ab initio MD simulations, which allowed us to predict entire UV–vis spectra with realistic line width. This approach was validated using our experimental data and provides a key advancement to the field by forming the foundation for the advanced in silico design of new red‐shifted azobenzene‐based photoswitches. With this method, we deduced that the photochemical behavior of azobenzenes depends strongly on the substitution pattern in the *ortho*‐ and *para*‐positions, while *meta*‐substituents have only a minor influence.

The combined experimental and computational results showed that the substitution of **dfdc** azobenzenes in the *para*‐position with electron‐poor substituents leads to a red shift by providing an accelerated photoconversion to the *cis*‐conformation through light irradiation within the bio‐optical window (650 nm). As key factors, we identified stabilizing effects on the π^*^ orbital and a decrease of the n → π* excitation energy, while electron‐rich *para*‐substituents cause a blue shift. By exploring the conformational flexibility and visualizing Pearson correlation matrices, we pinpoint important structural characteristics that influence the photoconversion. Our analysis shows that in‐plane *trans*‐configurations display the highest redshift. Remarkably, we discovered that accelerating the molecular motions by raising the temperature in **dfdb** azobenzene (**4**) samples, which concomitantly increases the occurrence of near‐planar conformations leads to enhanced photoswitching using red‐shifted wavelengths.

Examples that stood out among the experimentally investigated photoswitches were **12** and **28** for the ability to quantitatively switch to the *trans‐*state with 450 nm blue light and fast photoswitching to high levels of the *cis‐*isomer with 650 nm (90% for **12** and 77% for **28** after 15 min). Fast photoswitching with 650 nm light was also observed for the push–pull azobenzene **46** (93% after 15 min). **Dfdc** azobenzenes with one electron‐withdrawing group, particularly **21** and **24** (≥99% after 45 min) enabled quantitative photoswitching with deep red light (650 nm).

In general, the *cis*‐isomers of the **dfdc** and **dfdb** azobenzenes have a higher thermal stability than the standard and **toc** azobenzenes. The introduction of a *para*‐substituent results in a shortened half‐life due to the destabilization of the transition state, which is stabilized by negative charges on one aryl ring while stabilizing positive charges on the opposing aryl ring. This destabilization effect is particularly evident for the push–pull azobenzenes. All of this was rationalized by computations and confirmed through experiments.

Understanding the effect of the substitution pattern toward the photoabsorption, the thermal relaxation, the stability toward GSH in biological samples, and the influence of the structural flexibility allows us to predict photophysical properties of azobenzenes ahead of their synthesis. Our study serves as a foundational platform to guide the design, preparation, and optimization of photoswitches based on the desired application‐dependent photophysical properties and, particularly, enable the generation of optical tools that can be operated with red light.

Remarkably, since our first report on the **dfdc** azobenzene substitution pattern, their application for the development of optical devices has been investigated. A computational study has assessed the use of this substitution pattern to generate photoswitchable anion receptors with enhanced optical properties,^[^ ^50^ ^]^ while an experimental study has described phase change materials for the controlled heat storage and triggered release.^[^ ^51^ ^]^ In the latter, the **dfdc** azobenzene allowed the spontaneous energy storage with filtered sunlight while achieving a high thermal stability of Z isomers.

To showcase that the described azobenzene derivatives can be employed to generate red light‐operated photopharmaceuticals, which allow isomerization in biologically relevant time frame, we have incorporated the **dfdc** substitution pattern into OptoBI‐1 to generate **dfdc**‐OptoBI‐1. OptoBI‐1 is a photoswitchable chemical probe that has been used to control TRPC3 and TRPC6 channels with the spatiotemporal precision of light. By using whole‐cell patch‐clamp recordings on HEK293T cells overexpressing TRPC6, we demonstrated that, remarkably, the installation of the **dfdc** substitution pattern retains the biological activity of OptoBI‐1 and allows full activation of the TRPC6 channel with red light within ∼70 s. Altogether, our experiments validate **dfdc**‐OptoBI‐1 as a novel chemical tool for precise spatiotemporal control of TRPC6 channel activity that constitutes a major advancement that holds the potential for future applications in organs, tissues, and living organisms.

## Supporting Information

Full details about the experimental procedures and the structural analysis (Supporting Information I, PDF) as well as photostationary states, reversible photoswitching, stability against GSH, and thermal relaxation measurements (Supporting Information II, PDF) are available. X‐ray crystallographic data of *cis*‐**tof** (**2**, CCDC 2387092), *cis*‐**toc** (**6**, CCDC 2387095), *cis*‐carboxymethyl **dfdc** (**21**, CCDC 2387093), *trans*‐**dfdc** (**19**, CCDC 2387091), and *trans*‐symmetrical carboxymethyl **dfdc** (**28**, CCDC 2387094) azobenzene were uploaded to the CCDC database.^[^ ^23^, ^25^, ^26^, ^32^, ^52^, ^53^, ^54^, ^55^, ^56^, ^57^, ^58^, ^59^, ^60^ ^]^ Full details about the used quantum‐chemical methods, ab initio MD simulations and TD‐DFT calculations. Summaries of the computed spectroscopical and thermochemical properties for the *cis‐* and *trans‐*isomers of all compounds are available. Details about the correlation analysis and Pearson correlation matrices for selected compounds with different substitution patterns (**1**, **3**, **4**, **6**—no *meta*/*para*‐substituents; **21**, **28**—electron‐poor *para* substituents, **39**—electron‐rich *para*‐substituents, and **46**—push–pull substituents). Computed absorption spectra and natural transition orbitals for all molecules in the *trans‐* and *cis‐*configurations are included. Benchmarks of computed reaction barriers and excitation energies employing different density functionals are available (cf. Supporting Information III, PDF).^[^ ^38^, ^39^, ^46^, ^61^, ^62^, ^63^, ^64^, ^65^, ^66^, ^67^, ^68^, ^69^, ^70^, ^71^, ^72^, ^73^, ^74^, ^75^, ^76^, ^77^, ^78^, ^79^, ^80^, ^81^, ^82^, ^83^ ^]^ The .xyz files for optimized structures and MD trajectories have been deposited to Zenodo (https://doi.org/10.5281/zenodo.15441450). Full details on the biological investigation of **dfdc**‐OptoBI‐1 are available (Supporting Information IV, PDF).

## Conflict of Interests

The authors declare no conflict of interest.

## Supporting information



Supporting Information

Supporting Information

Supporting Information

Supporting Information

## Data Availability

The data that support the findings of this study are available in the Supporting Information of this article.
