# Supplementary material for: A Platform for the Development of Highly Red‐Shifted Azobenzene‐Based Optical Tools
Source: Angew Chem Int Ed Engl. 2025 Jun 23;64(32):e202501779. doi: 10.1002/anie.202501779 (PMC12322654; doi:10.1002/anie.202501779)
Supplement: Supplementary file 1 — Supporting Information [file ANIE-64-e202501779-s001.pdf]

# A platform for the development of Highly Red-Shifted Azobenzene-based Optical Tools

Kyra Lützel[#], Henryk Laqua[#], Manjima B. Sathian[#], Benedikt Nißl[#], Judit Katalin Szántó, Christina-Anna Senser, Gökcen Savasci, Lars Allmendinger, Bilal Kicin, Vincent Ruf, Dominik Kammerer, Theobald Lohmüller, Konstantin Karaghiosoff, Ahmed M. Ali, Ursula Storch*, Michael Mederos y Schnitzler*, Christian Ochsenfeld* and David B. Konrad*

[#] these authors contributed equally to this work

[*] corresponding authors

# Supporting Information I: Chemical Synthesis

# Table of Content

**ABBREVIATIONS** .......... **3**

**EXPERIMENTAL SECTION** .......... **5**

MATERIAL AND METHODS .......... 5

*Equipment and Instruments* .......... 5

*Methods* .......... 6

*Chemicals* .......... 7

COMPOUND LIST .......... 8

EXPERIMENTAL PROCEDURES .......... 8

*Final Compounds* .......... 8

*Precursor* .......... 21

NMR DATA – STRUCTURAL ANALYSIS .......... 28

*Final compounds* .......... 28

*Precursor* .......... 55

**LITERATURE REFERENCES** .......... **68**

# Abbreviations

| | |
|---|---|
| Ac | acetyl |
| AcOH | acetic acid |
| aq | aqueous |
| DBDMH | 1,3-dibromo-5,5-dimethylhydantoin |
| DCE | dichloroethan |
| dfdb | di-fluoro-di-bromo |
| dfdc | di-fluoro-di-bromo |
| DMF | dimethylformamide |
| DMSO | dimethylsulfoxide |
| EI | electron ionization |
| ESI | electrospray ionization |
| EtOAc | ethyl acetate |
| EtOH | ethanol |
| eq | equivalent |
| GSH | glutathione |
| HATU | O-(7-Azabenzotriazol-1-yl)-*N*,*N*,*N′*,*N′*-tetramethyluronium-hexafluorphosphat |
| HPLC | High Perfomance Liquid Chromatography |
| IR | Infrared spectroscopy |
| IUPAC | International Union of Pure and Applied Chemistry |
| LED | light-emitting diode |
| NBS | *N*-bromosuccinimide |
| NCS | *N*-chlorosuccinimide |
| NMR | Nuclear magnetic resonance spectroscopy |
| Me | methyl |
| MeCN | acetonitrile |
| MS | mass spectrometry |
| PBS | phosphate-buffered saline |

| | |
|---|---|
| Ph | phenyl |
| ppm | parts per million |
| PSS | photostationary states |
| *p*TsOH | ***p***-toluenesulfonic acid |
| rt | room temperature |
| THF | tetrahydrofuran |
| toc | tetra-*ortho*-chloro |
| UV-vis | ultraviolet- visible |

# Experimental section

## Material and Methods

### Equipment and Instruments

**Nuclear magnetic resonance (NMR) spectroscopy:**

NMR spectra were acquired with the following spectrometers: Avance III HD 400 MHz Bruker BioSpin with a broadband probehead ($^{19}$F NMR with $^{1}$H decoupling), a 500 MHz Avance III HD Bruker BioSpin equipped with a CryoProbe™ Prodigy ($^{19}$F NMR without $^{1}$H decoupling) and a 800 MHz Bruker Avance III HD equipped with a CryoProbe™ Prodigy. For spectra measured in $CDCl_3$, $(CD_3)_2CO$, $(CD_3)_2SO$, $CD_3OD$ and $D_2O$ the residual solvent signal was used as internal references. Signal multiplicities are described as follows: s (singlet), d (doublet), t (triplet), or a combination thereof and m (multiple). Structural analysis was conducted with $^{1}$H, $^{19}$F and $^{13}$C NMR spectra with the aid of additional 2D spectra (COSY, HMBC, HMQC, NOESY). Spectra analysis was conducted with the software MestReNova v.10.0.1-14719.

**Mass spectrometry (MS):**

High resolution MS spectra were recorded either on Finnigan MAT 95Q (EI: electron ionization), Thermo Finnigan LTQ FT Ultra Fourier Transform Ionen Cyclotron Resonanz or Thermo Finnigan LTQ Orbitrap XL mass spectrometer (ESI: electrospray ionization).

**Infrared spectroscopy (IR):**

IR spectra were recorded on a PerkinElmer Spectrum BX II FT-IR device equipped with an attenuated total reflection (ATR) measuring unit. For measurements, the neat substances were directly applied as a thin film on the ATR unit. The measured wavenumbers are reported with their relative intensities which were classified as: vs (very strong), s (strong), m (medium), w (weak) or vw (very weak).

**UV-vis spectroscopy:**

UV-vis spectra were recorded on a Cary 60 UV-Vis spectrophotometer (Agilent) and as cuvette a Hellma Analytics High Precision Cell (quartz glass, 10.00 mm light path) was used. Analysis was conducted with the Cary win UV/Scan.exe software. Illumination was provided by high-power LEDs purchased from EvoluChem™.
EvoluChem™: 365 nm (HCK1012-01-011), 450-455 nm (HCK1012-01-002), 525 nm (HCK1012-01-004), 650 nm (HCK1012-01-014), 740 nm (HCK1012-01-015).
The optical power output of the LEDs was measured using a ThorLabs (https://www.thorlabs.de) Power and Energy Meter Interface PM100 USB equipped with the Microscope Slide Power Sensor S170C. The optical power output of the LEDs used for the measurement of the photostationary states and the thermal relaxation is 1.18 mW/mm$^2$ for 740 nm, 1.12 mW/mm$^2$ for 650 nm, 0.52 mW/mm$^2$ for 525 nm, 0.53 mW/mm$^2$ for 450 nm and 0.11 mW/mm$^2$ for 365 nm.
Illumination for reversible photostationary states was provided by the illumination system pE-4000 from CoolLED. The optical power output of the LEDs according to CoolLED is 50.1 mW/mm$^2$ for 660 nm, 9.95 mW/mm$^2$ for 525 nm, 50.3 mW/mm$^2$ for 460 nm, and 50.4 mW/mm$^2$ for 365 nm.

The experiments were conducted using a 50 µM and 500 µM solution of the compound in DMSO:$H_2O$ (9:1).

## Single Crystal X-Ray Diffraction

Single crystals suitable for X-ray diffraction, were obtained by slow evaporation of **ethanol** solution. The crystals were introduced into perfluorinated oil and a suitable single crystal was carefully mounted on the top of a thin glass wire. The X-ray intensity data were measured on a 'D8 Venture' system equipped with a 'Bruker D8 Venture TXS' 'rotating-anode X-ray tube' ('Mo Kα', λ = 0.71073 Å) and a 'multilayer mirror optics' monochromator.
Data collection[1], data reduction[2] and cell refinement[3] were performed with the Bruker specific software. Absorption correction using the multiscan method was applied. The structures were solved with SHELXS-97[4], refined with SHELXL-97[5] and finally checked using PLATON[6]. Details for data collection and structure refinement are summarized in the corresponding Table.
CCDC2387091, CCDC2387092, CCDC2387093, CCDC2387094 and CCDC2387095 contains supplementary crystallographic data for this compound. These data can be obtained free of charge from The Cambridge Crystallographic Data Centre *via* www.ccdc.cam.ac.uk/data_request/cif.

## High Performance Liquid Chromatography (HPLC):

A Varian Prep Star HPLC System Model SD-1 was used equipped with Varian Dynamax reverse phase columns (semi-preparative: Microsorb 60 C18, 250 x 21.4 mm, particle size 8 µm; preparative: Microsorb 60 C18, 250 x 41.4 mm, particle size 8 µm). Prior to injection, samples were filtered through a syringe filter (Chromafil Xtra GF100/25, pore size 1 µm).

## Methods

Unless otherwise noted, all reactions were stirred under inert gas ($N_2$) atmosphere using standard Schlenk techniques. Glassware was evacuated and dried by heating with a heat-gun (set to 550 °C). Drying over $Na_2SO_4$ or $MgSO_4$ implies stirring with an appropriate amount of anhydrous salt for several minutes followed by filtration through a glass frit and rinsing of the filter cake with additional solvent. Electric heating plates and oil baths were used for reactions at elevated temperature. Stated reaction temperatures refer to the external bath temperature. Cannulas and syringes were used for the transfer of reagents and solvents, which were flooded with inert gas (3×) before use. Purification by column chromatography was performed under elevated pressure (flash column chromatography) on silica gel ($SiO_2$, 60–20 µm, 60 Å) from Acros Organics. After flash column chromatography, the concentrated fractions were filtered once through a glass frit. Silica gel 60 $F_{254}$ TLC plates from Merck KGaA were used for monitoring reactions, analyzing fractions of column chromatography and measuring $R_f$ values. To visualize the analytes, TLC plates were irradiated with UV light and/or treated with appropriate staining solutions followed by subsequent heating. Freeze-drying refers to freezing of the respective sample in liquid nitrogen followed by evacuating the containing flask with high vacuum (< 1 mbar) and slow thawing to rt. Reaction yields refer to NMR spectroscopically pure (purity of >95%) isolated amounts of compounds.

## Chemicals

All chemicals were purchased from *Sigma Aldrich*, *Fisher Scientific*, *TCI Europe*, *Alfa Aesar, BLDPharm* or *Acros Organics*. Solvents purchased in technical grade quality and were distilled under reduced pressure and used for purification procedures. Purchased solvents in HPLC- and analytical-grade quality were used without further purification. Unless otherwise noted, reactions were performed using dry solvents which were purchased from commercial sources (*Sigma Aldrich, TCI Europe, BLDPharm* or *Fisher Scientific*). All other reagents with a purity of >95% purchased from commercial sources were used without further purification.

# Compound List

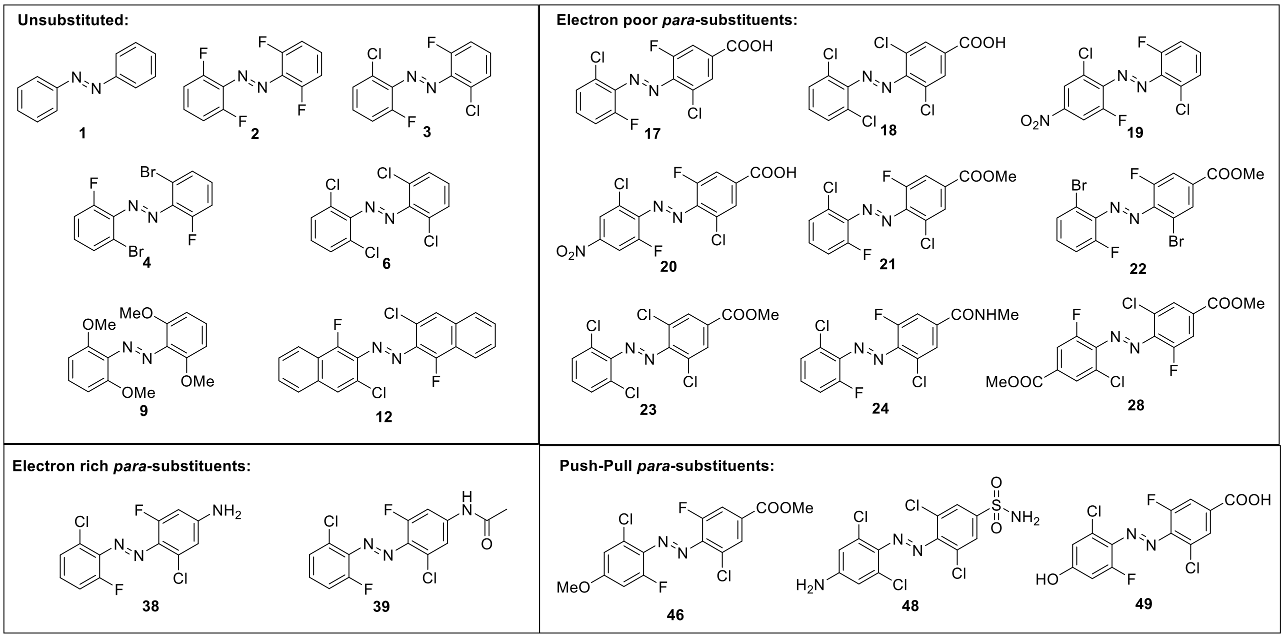


# Experimental Procedures

## Final Compounds

The compounds presented here are mainly mixtures of (*Z*)- and (*E*)-isomers. For the $^{13}$C NMR only the major isomer was described, as the minor isomer is not fully visible due to the signal-to-noise ratio.

### Literature-known azobenzene derivatives

Azobenzene (**1**), 1,2-bis(2,6-difluorophenyl)diazene[7] (**2**), 1,2-bis(2-chloro-6-fluorophenyl)diazene[8] (**3**), 1,2-bis(2,6-dichlorophenyl)diazene[8] (**6**) and 1,2-bis(2,6-dimethoxyphenyl)diazene[9] (**9**) were prepared according to a literature procedure.

### (*E*/*Z*)-1,2-Bis(2-bromo-6-fluorophenyl)diazene (4)

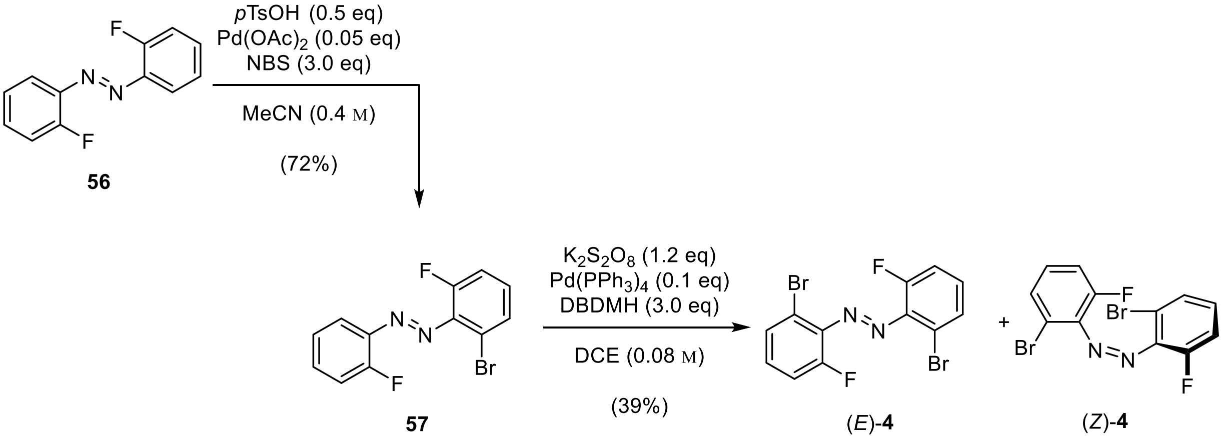


1,2-Bis(2-fluorophenyl)diazene[8,10] (**56**, 50 mg, 0.23 mmol, 1.0 eq. ), *p*TsOH (19.7 mg, 0.11 mmol, 1.2 eq),

$Pd(OAc)_2$ (3 mg, 0.01 mmol, 0.1 eq) and NBS (123 mg, 0.69 mmol, 3.0 eq) were dissolved in MeCN (0.58 mL) and stirred at 84 °C for 24 h. After cooling to rt, the solvent was removed *in vacuo*. Purification by flash column chromatography (*n*-hexane 100) afforded 1-(2-bromo-6-fluorophenyl)-2-(2-fluorophenyl)diazene (**57**, 49.2 mg, 0.17 mmol, 72%) as a red solid (***R*f** (*n*-hexane/$CH_2Cl_2$ 90:10) = 0.49. (visible)).

1-(2-Bromo-6-fluorophenyl)-2-(2-fluorophenyl)diazene (**57**, 49.2 mg, 0.17 mmol, 1.0 eq), $K_2S_2O_8$ (53.7 mg, 0.20 mmol, 1.2 eq), $Pd(PPh_3)_4$ (19.6 mg, 0.02 mmol, 0.1 eq) and DBDMH (146 mg, 0.51 mmol, 3.0 eq) were dissolved in DCE (2.13 mL) and stirred at 110 °C for 24 h. After cooling to rt, the solvent was removed *in vacuo*. Purification by flash column chromatography (*n*-hexane 100) afforded (*E*/*Z*)-1,2-bis(2-bromo-6-fluorophenyl)diazene ((*E*/*Z*)-**4**, 25.2 mg, 0.07 mmol, 39%) as an isomeric mixture (*E*/*Z* = 35:65 ($^1$H NMR)) red solid.

***R*f** (*n*-hexane/EtOAc 9:1) = 0.64. (visible)

**$^1$H NMR (800 MHz, $(CD_3)_2CO$) *E*-Isomer: δ (ppm)** 7.72 (d, *J* = 8.2 Hz, 2H), 7.50 (td, *J* = 8.2, 5.3 Hz, 2H), 7.47–7.41 (m, 2H). ***Z*-Isomer: δ (ppm)** 7.65 (d, *J* = 8.2 Hz, 2H), 7.33 (td, *J* = 8.2, 4.7 Hz, 2H), 7.07 (t, *J* = 9.0 Hz, 2H).

**$^{13}$C{$^1$H} NMR (201 MHz, $(CD_3)_2CO$) *Z*-Isomer: δ (ppm)** 152.7 (d, *J* = 262.2 Hz), 141.0 (d, *J* = 17.2 Hz), 133.0 (d, *J* = 9.5 Hz), 130.6, 121.5, 116.5 (d, *J* = 20.6 Hz).

**$^{19}$F{$^1$H} NMR (376 MHz, $(CD_3)_2CO$) *E*-Isomer: δ (ppm)** -117.7. ***Z*-Isomer: δ (ppm)** -124.9.

**HRMS:** (EI) calculated for $C_{12}H_6Br_2F_2N_2$ [M]: 373.8876 found: 373.8876.

**IR (Diamond-ATR, neat) $\tilde{\nu}$ (cm$^{-1}$) =** 3084 (w), 2922 (w), 2852 (w), 1936 (w), 1588 (m), 1568 (m), 1512 (w), 1452 (s), 1400 (w), 1284 (w), 1250 (s), 1220 (m), 1178 (w), 1152 (w), 1138 (w), 1076 (w), 1058 (w), 894 (m), 878 (vs), 844 (m), 812 (m) 778 (vs), 756 (m), 736 (s), 704 (w), 676 (w).

### 1,2-Bis(3-chloro-1-fluoronaphthalen-2-yl)diazene (12)

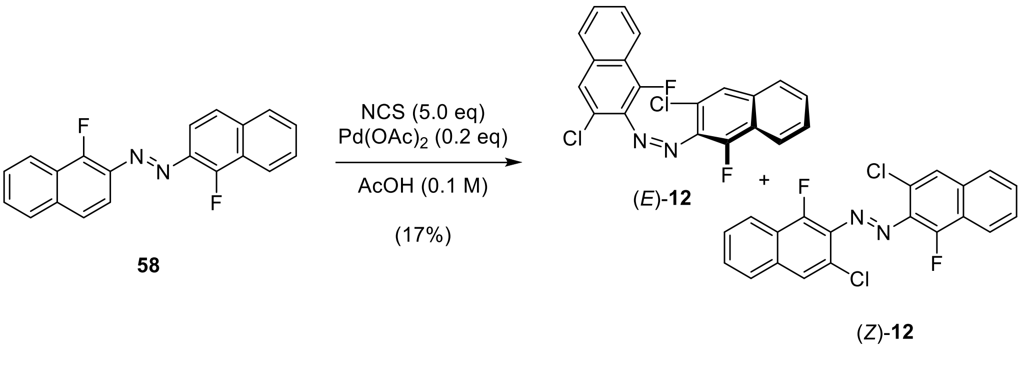


1,2-Bis(1-fluoronaphthalen-2-yl)diazene[11] (**58**, 19.9 mg, 0.06 mmol, 1.0 eq.), NCS (41.7 mg, 0.31 mmol, 5.0 eq.) and $Pd(OAc)_2$ (3 mg, 0.01 mmol, 0.2 eq) were dissolved in AcOH (0.6 mL) and stirred at 120 °C for 6 h. The reaction mixture was cooled to rt and concentrated *in vacuo.* Purification by flash column chromatography (*n*-hexane/EtOAc 95:5) afforded 1,2-bis(3-chloro-1-fluoronaphthalen-2-yl)diazene (**12**, 4.0 mg, 0.01 mmol, 17%) as an isomeric mixture (*E*/*Z* = 56:44 ($^{19}$F{$^1$H} NMR)) red solid.

***R*f** (*n*-hexane/EtOAc 9:1) = 0.56. (visible)

**$^{1}$H NMR (400 MHz, $(CD_3)_2CO$) *E*-Isomer: δ (ppm)** 8.32–8.24 (m, 2H), 8.15 (m, 2H), 8.08 (m, 2H), 7.83–7.73 (m, 4H).

**$^{13}$C{$^{1}$H} NMR (201 MHz, $(CD_3)_2CO$) *E*-Isomer: δ (ppm)** 148.0 (d, *J* = 269.8 Hz), 135.1, 130.6, 129.9, 128.8, 128.1 (d, *J* = 21.1 Hz), 126.0, 125.6 (d, *J* = 3.6 Hz), 122.9 (d, *J* = 6.3 Hz), 122.2.

**$^{19}$F{$^{1}$H} NMR (376 MHz, $(CD_3)_2CO$) *E*-Isomer: δ (ppm)** -135.0. ***Z*-Isomer: δ (ppm)** -129.5.

**HRMS:** (EI) calculated for $C_{20}H_{10}Cl_2F_2N_2$ [M]: 386.0189 found: 386.0187.

**IR (Diamond-ATR, neat) $\tilde{\nu}$ (cm$^{-1}$) =** 3328 (m), 3072 (w),2974 (m), 2922 (w), 1620 (w), 1572 (m), 1494 (m), 1444 (m), 1428 (m), 1376 (m), 1332 (m), 1266 (m), 1088 (s), 1046 (vs), 982 (m), 902 (m), 876 (m), 838 (m), 828 (m), 794 (w), 772 (w), 742 (s), 712 (w), 668 (w).

## (*E*/*Z*)-3-Chloro-4-((2-chloro-6-fluorophenyl)diazenyl)-5-fluorobenzoic acid (17)

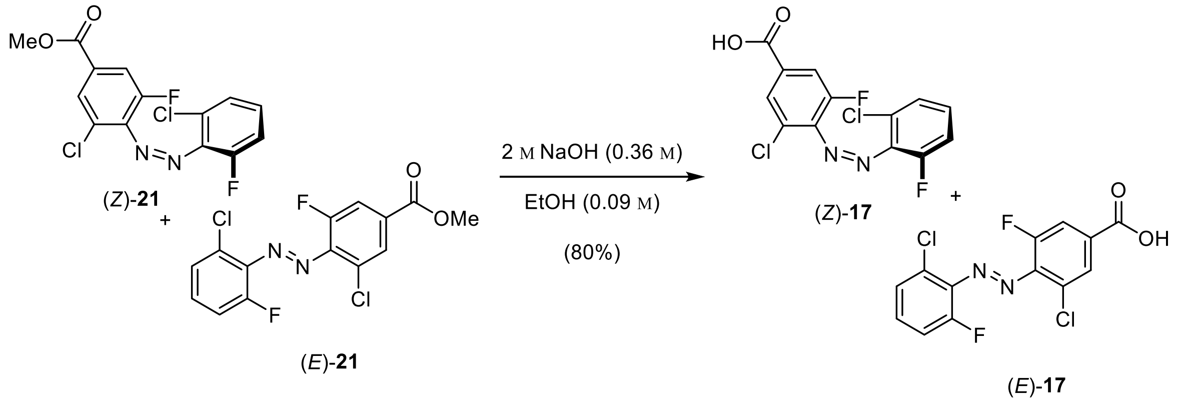


2 M aq. NaOH (1.88 mL) was added to (E/Z)-methyl-3-chloro-4-((2-chloro-6-fluorophenyl)diazenyl)-5-fluorobenzoate (**21**, 233 mg, 0.68 mmol, 1.0 eq.) in EtOH (7.56 mL) and stirred at rt for 18 h. After concentrating *in vacuo,* the residue was redissolved in $H_2O$ (10 mL), transferred into a separatory funnel and washed with $CH_2Cl_2$ (2 × 20 mL). The aqueous phase was acidified with 2 M aq. HCl (5 mL, pH3) and extracted with $CH_2Cl_2$ (3 × 50 mL). The combined organic phases were dried over $Na_2SO_4$ and concentrated under reduced pressure. Purification by flash column chromatography (*n*-hexane/EtOAc/AcOH 89.9:10:0.1) afforded (*E*/*Z*)-3-chloro-4-((2-chloro-6-fluorophenyl)diazenyl)-5-fluorobenzoic acid ((*E*/*Z*)-**17**, 180 mg, 0.54 mmol, 80%) as an isomeric mixture (*E*/*Z* = 67:33 ($^{19}$F{$^{1}$H} NMR)) red solid.

***R*f** (*n*-hexane/EtOAc/AcOH 49.9:50:0.1) = 0.13. (visible)

**$^{1}$H NMR (400 MHz, $(CD_3)_2CO$) *E*-Isomer: δ (ppm)** 8.07 (s, 1H), 7.91 (d, *J* = 10.7 Hz, 1H), 7.66–7.55 (m, 2H), 7.42 (ddd, *J* = 10.7, 8.0, 1.7 Hz, 1H). ***Z*-Isomer: δ (ppm)** 7.97 (s, 1H), 7.64 (d, *J* = 5.2 Hz, 1H), 7.50 – 7.45 (m, 2H), 7.12 – 7.04 (m, 1H).

**$^{13}$C{$^{1}$H} NMR (101 MHz, $(CD_3)_2CO$) *E*-Isomer: δ (ppm)** 164.9, 153.0 (d, *J* = 298.9 Hz), 153.0 (d, *J* = 223.1 Hz), 150.2, 147.7, 143.0 (d, *J* = 10.7 Hz), 139.8 (d, *J* = 9.6 Hz), 133.4 (d, *J* = 9.6 Hz), 132.7, 128.1 (d, *J* = 3.7 Hz), 127.5 (d, *J* = 3.7 Hz), 118.0 (d, *J* = 22.0 Hz), 117.2 (d, *J* = 20.4 Hz).

**$^{19}$F{$^{1}$H} NMR (376 MHz, $(CD_3)_2CO$) *E*-Isomer: δ (ppm)** -124.6, -124.7. ***Z*-Isomer: δ (ppm) -**118.2 (d, *J* = 8.9 Hz), -119.3 (d, *J* = 8.9 Hz).

**HRMS:** (EI) calculated for $C_{13}H_5Cl_2F_2N_2O_2$ [M-H]: 328.97012 found: 328.96994.

**IR (Diamond-ATR, neat) $\tilde{\nu}$ (cm$^{-1}$)** = 3078 (m), 2630 (w), 1922 (w), 1780 (w), 1700 (vs), 1592 (m), 1572 (m), 1496 (w), 1452 (s), 1414 (s), 1402 (s), 1288 (s), 1260 (m), 1242 (s), 1180 (m), 1146 (w), 1090 (w), 1062 (w), 978 (m), 928 (s), 882 (vs), 772 (s), 742 (s), 722 (m), 700 (m), 658 (w).

## (*E*)-1-(2-Chloro-6-fluoro-4-nitrophenyl)-2-(2-chloro-6-fluorophenyl)diazene (19)

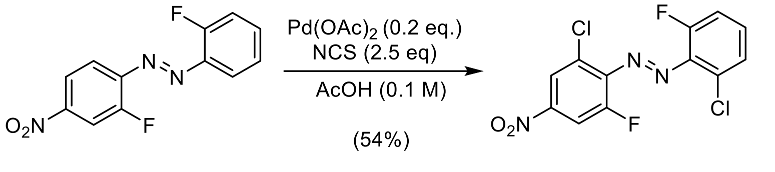


**59** **19**

A pressure tube was charged with (*E*)-1-(2-fluoro-4-nitrophenyl)-2-(2-fluorophenyl)diazene (**59**, 1.00 g, 3.79 mmol, 1.0 eq.), NCS (1.27 g, 9.50 mmol, 2.5 eq.) and $Pd(OAc)_2$ (170 mg, 0.76 mmol, 0.2 eq.). AcOH (38.0 mL) was added and the tube was sealed, warmed to 120 °C and stirred for 16 h. After cooling to rt, the solvent was removed under reduced pressure and the mixture was transferred to a separatory funnel with $CH_2Cl_2$ (50 mL). The organic layer was washed with sat. aq. $NaHCO_3$ (2 × 50 mL), dried over $Na_2SO_4$ and concentrated under reduced pressure. Purification by flash column chromatography afforded (*E*)-1-(2-chloro-6-fluoro-4-nitrophenyl)-2-(2-chloro-6-fluorophenyl)diazene (**19**, 681 mg, 2.05 mmol, 54%) as a red solid.

***R*f** (cyclohexane/$CHCl_3$ 1:1) = 0.4. (visible)

**$^{1}$H NMR (500 MHz, $(CD_3)_2CO$) δ (ppm)** = 8.46–8.41 (m, 1H), 8.32 (dd, *J* = 10.4, 2.3 Hz, 1H), 7.67 (td, *J* = 8.3, 5.3 Hz, 1H), 7.62 (dt, *J* = 8.3, 1.3 Hz, 1H), 7.46 (ddd, *J* = 10.4, 8.2, 1.3 Hz, 1H).

**$^{13}$C{$^{1}$H} NMR (126 MHz, $(CD_3)_2CO$) δ (ppm)** = 154.0 (d, *J* = 70.7 Hz), 151.9 (d, *J* = 69.8 Hz), 148.9 (d, *J* = 9.4 Hz), 144.5 (d, *J* = 10.4 Hz), 139.6 (d, *J* = 9.4 Hz), 134.1 (d, *J* = 10.4 Hz), 133.3 (d, *J* = 2.8 Hz), 131.7 (d, *J* = 2.8 Hz), 127.7 (d, *J* = 3.8 Hz), 122.6 (d, *J* = 4.5 Hz), 117.3 (d, *J* = 20.7 Hz), 113.3 (d, *J* = 25.5 Hz).

**$^{19}$F{$^{1}$H} NMR (376 MHz, $(CD_3)_2CO$) δ (ppm)** = -121.8, -124.1.

**HRMS:** (EI) calculated for $C_{12}H_5Cl_2F_2N_3O_2$ •+ [M − e$^{-}$]: 330.9721 found: 330.9719.

**IR (Diamond-ATR, neat) $\tilde{\nu}$ (cm$^{-1}$)** = 3093 (w), 1590 (w), 1578 (w), 1536 (m), 1527 (s), 1494 (w), 1455 (m), 1422 (m), 1344 (s), 1305 (m), 1258 (m), 1216 (w), 1174 (w), 1074 (w), 978 (m), 932 (s) 894 (S), 885 (s), 809 (w), 792 (vs), 758 (m), 740 (vs), 703 (w), 663 (m).

## (*E*)-3-Chloro-4-((2-chloro-6-fluoro-4-nitrophenyl)diazenyl)-5-fluorobenzoic acid (20)

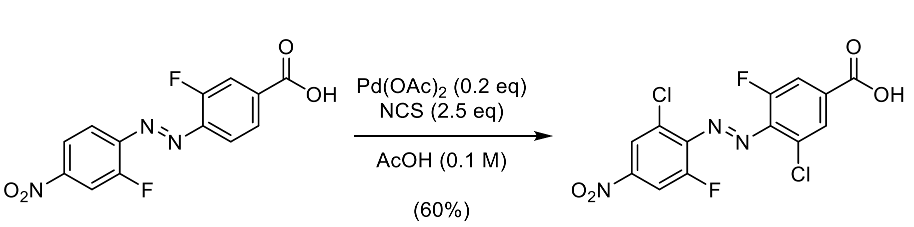


**60** **20**

($E$)-3-Fluoro-4-((2-fluoro-4-nitrophenyl)diazenyl)benzoic aciddiazene (**60**, 500 mg, 1.60 mmol, 1.0 eq.), Pd(OAc)$_2$ (73.1 mg, 0.30 mmol, 0.2 eq.) and NCS (534 mg, 4.00 mmol, 2.5 eq.) were dissolved in AcOH (16 mL) and stirred at 120 °C for 4 h. After cooling to rt the precipitated residue was filtered off. Purification by recrystallization from EtOH afforded ($E$)-3-chloro-4-((2-chloro-6-fluoro-4-nitrophenyl)diazenyl)-5-fluorobenzoic acid (**20**, 360 mg, 0.96 mmol, 60%) as a red solid.

***R*f** ($CH_2Cl_2$/MeOH 97.5:2.5) = 0.1. (visible)
**$^1$H NMR (400 MHz, $(CD_3)_2CO$) δ (ppm)** = 8.43 (t, $J$ = 2.1 Hz, 1H), 8.32 (dd, $J$ = 10.4, 2.1 Hz, 1H), 8.11 (d, $J$ = 1.6 Hz, 1H), 7.94 (dd, $J$ = 10.4, 1.6 Hz, 1H), 1.96 (s, 1H).
**$^{13}$C{$^1$H} NMR (126 MHz, $(CD_3)_2CO$) δ (ppm)** = 172.0, 164.7 (d, $J$ = 2.4 Hz), 153.7 (d, $J$ = 19.7 Hz), 151.6 (d, $J$ = 20.4 Hz), 149.2 (d, $J$ = 9.4 Hz), 144.1 (d, $J$ = 10.8 Hz), 142.4 (d, $J$ = 10.0 Hz), 135.2 (d, $J$ = 8.5 Hz), 132.5 (d, $J$ = 2.3 Hz), 132.2 (d, $J$ = 2.9 Hz), 128.3 (d, $J$ = 3.7 Hz), 122.7 (d, $J$ = 4.1 Hz), 118.2 (d, $J$ = 22.2 Hz), 113.4 (d, $J$ = 25.9 Hz).
**$^{19}$F{$^1$H} NMR (376 MHz, $(CD_3)_2SO$) δ (ppm)** = -121.3, -123.5.
**HRMS:** (ESI) calculated for $C_{13}H_4Cl_2F_2N_3O_4$ (M-H): 373.9552 found: 373.95548.
**IR (Diamond-ATR, neat) $\tilde{\nu}$ (cm$^{-1}$)** = 3090 (vw), 1703 (vs), 1567 (w), 1527 (s), 1459 (w), 1420 (s), 1350 (s), 1291 (s), 1246 (m), 1177 (w), 973 (s), 894 (vs), 797 (m), 767 (m), 742 (vs), 696 (m).

## (*E*/*Z*)-Methyl-3-chloro-4-((2-chloro-6-fluorophenyl)diazenyl)-5-fluorobenzoate (21)

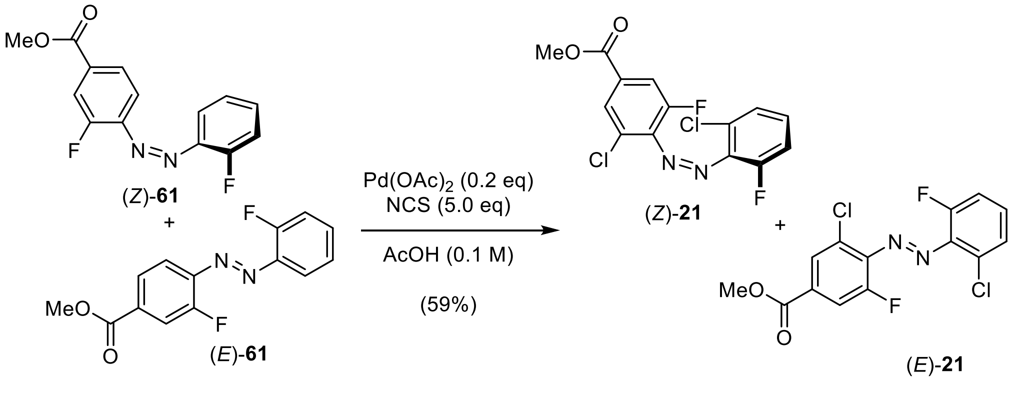


($E$/$Z$)-Methyl-3-fluoro-4-((2-fluorophenyl)diazenyl)benzoate (**61,** 317 mg, 1.15 mmol, 1.0 eq.), Pd(OAc)$_2$ (51.6 mg, 0.23 mmol, 0.2 eq.) and NCS (766 mg, 5.73 mmol, 5.0 eq.) were dissolved in AcOH (11.5 mL) and stirred at 120 °C for 6.5 h. After cooling to rt the reaction mixture was concentrated *in vacuo*. Purification by flash column chromatography ($n$-hexane/EtOAc 90:10) afforded ($E$/$Z$)-methyl-3-chloro-4-((2-chloro-6-fluorophenyl)diazenyl)-5-fluorobenzoate (($E$/$Z$)-**21**, 233 mg, 0.68 mmol, 59%) as an isomeric mixture ($E$/$Z$ = 88:12 ($^{19}$F{$^1$H} NMR)) red solid.

***R*f** ($n$-hexane/EtOAc 90:10) = 0.56. (visible)

**$^{1}$H NMR (400 MHz, CDCl$_3$)** ***E*-Isomer: δ (ppm)** 8.04 (t, *J* = 1.6 Hz, 1H), 7.81 (d, *J* = 1.6 Hz, 1H), 7.42–7.32 (m, 2H), 7.22–7.13 (m, 1H), 3.97 (s, 3H). ***Z*-Isomer: δ (ppm)** 7.93 (t, *J* = 1.5 Hz, 1H), 7.48 (dd, *J* = 9.8, 1.5 Hz, 1H), 7.29 (dt, *J* = 8.3, 1.2 Hz, 2H), 6.81 (ddd, *J* = 8.3, 1.2 Hz, 1H), 3.91 (s, 3H).

**$^{13}$C{$^{1}$H} NMR (101 MHz, CDCl$_3$)** ***E*-Isomer: δ (ppm)** 164.5 (d, *J* = 3.2 Hz), 153.5, 152.7 (d, *J* = 263.6 Hz), 150.9, 142.7 (d, *J* = 10.4 Hz), 139.4, 132.9, 132.1 (d, *J* = 8.6 Hz), 131.6 (d, *J* = 9.7 Hz), 127.4, 126.5, 117.1 (d, *J* = 22.5 Hz), 116.1 (d, *J* = 20.4 Hz), 53.1.

**$^{19}$F{$^{1}$H} NMR (376 MHz, CDCl$_3$)** ***E*-Isomer: δ (ppm)** -123.0, -123.0. ***Z*-Isomer: δ (ppm)** -116.6 (d, *J* = 9.3 Hz), -117.6 (d, *J* = 8.4 Hz).

**HRMS:** (EI) calculated for $C_{14}H_8Cl_2F_2N_2O_2$ [M]: 343.9931 found: 343.9920.

**IR (Diamond-ATR, neat) $\tilde{\nu}$ (cm$^{-1}$)** = 3088 (w), 2956 (w), 2924 (w), 2850 (vw), 1722 (s), 1592 (m), 1576 (m), 1490 (w), 1452 (s), 1410 (s), 1286 (s), 1254 (m), 1226 (s), 1196 (m), 1180 (m), 1154 (w), 1094 (m), 1032 (w), 1004 (m), 948 (w), 922 (vs), 890 (m), 874 (m), 806 (w), 778 (s), 764 (s), 742 (s), 704 (w), 688 (w), 662 (w).

### (*E*/*Z*)-Methyl-3-bromo-4-((2-bromo-6-fluorophenyl)diazenyl)-5-fluorobenzoate (22)

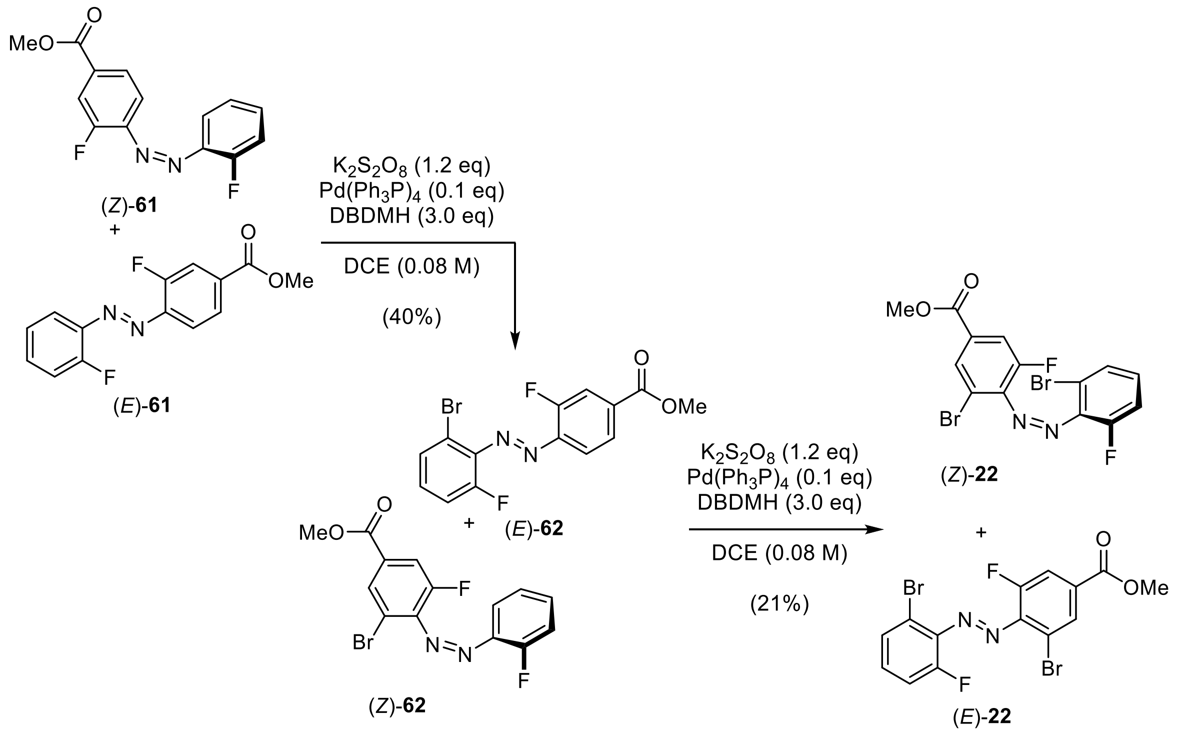


(*E*/*Z*)-Methyl-3-fluoro-4-((2-fluorophenyl)diazenyl)benzoate (**61**, 100 mg, 0.36 mmol, 1.0 eq.), $K_2S_2O_8$ (117 mg, 0.43 mmol, 1.2 eq), $Pd(PPh_3)_4$ (41.6 mg, 0.04 mmol, 0.1 eq) and DBDMH (309 mg, 1.08 mmol, 3.0 eq) were dissolved in DCE (4.5 mL) and stirred at 110 °C for 23 h. The reaction mixture was concentrated *in vacuo* and purified by flash column chromatography (*n*-hexane/EtOAc 90:10), which yielded (*E*/*Z*)-methyl-4-((2-bromo-6-fluorophenyl)diazenyl)-3-fluorobenzoate ((*E*/*Z*)-**62**, 51.0 mg, 0.14 mmol, 40%) as a red solid (***R*f** (*n*-hexane/EtOAc 90:10) = 0.56. (visible)).

(*E*/*Z*)-Methyl-4-((2-bromo-6-fluorophenyl)diazenyl)-3-fluorobenzoate (**62**, 51.0 mg, 0.14 mmol, 1.0 eq), $K_2S_2O_8$ (45.4 mg, 0.17 mmol, 1.2 eq), $Pd(PPh_3)_4$ (16.2 mg, 0.01 mmol, 0.1 eq) and DBDMH (120 mg, 0.42 mmol, 3.0 eq) were dissolved in DCE (1.8 mL) and stirred at 110 °C for 16 h. The reaction mixture was concentrated *in vacuo* and purified by flash column chromatography (*n*-hexane/EtOAc 90:10), which yielded (*E*/*Z*)-methyl-3-bromo-4-

((2-bromo-6-fluorophenyl)diazenyl)-5-fluorobenzoate ((*E*/*Z*)-**22**, 12.8 mg, 0.03 mmol, 21%) as an isomeric mixture (*E*/*Z* = 62:38 ($^{19}$F{$^{1}$H} NMR)) dark red solid.

***R*f** (*n*-hexane/EtOAc 90:10) = 0.58. (visible)

**$^{1}$H NMR (800 MHz, $CDCl_3$) *E*-Isomer: δ (ppm)** 8.22 (s, 1H), 7.85 (dd, *J* = 10.4, 1.6 Hz, 1H), 7.59 (d, *J* = 8.1 Hz, 1H), 7.29 (td, *J* = 8.1, 5.1 Hz, 1H), 7.22 (dd, *J* = 10.4, 8.1 Hz, 1H), 3.97 (s, 3H). ***Z*-Isomer: δ (ppm)** 8.15 (s, 1H), 7.52 – 7.47 (m, 2H), 7.13 (td, *J* = 8.2, 5.4 Hz, 1H), 6.83 (t, *J* = 9.0 Hz, 1H), 3.91 (s, 3H).

**$^{13}$C{$^{1}$H} NMR (201 MHz, $CDCl_3$) *E*-Isomer: δ (ppm)** 164.4, 152.8, 151.0, 143.6, 143.5, 140.2, 131.9 (d, *J* = 9.4 Hz), 130.4, 129.5, 122.7, 120.7, 117.9 (d, *J* = 22.3 Hz), 116.8 (d, *J* = 20.6 Hz), 53.1.

**$^{19}$F{$^{1}$H} NMR (376 MHz, $CDCl_3$) *E*-Isomer: δ (ppm)** -122.4, -122.5. ***Z*-Isomer: δ (ppm)** -115.4 (d, *J* = 6.9 Hz), -116.1 (d, *J* = 6.9 Hz).

**HRMS:** (EI) calculated for $C_{14}H_8Br_2F_2N_2O_2$ [M]: 431.8921 found: 431.8911.

## (*E*/*Z*)-Methyl-3,5-dichloro-4-((2,6-dichlorophenyl)diazenyl)benzoate (23)

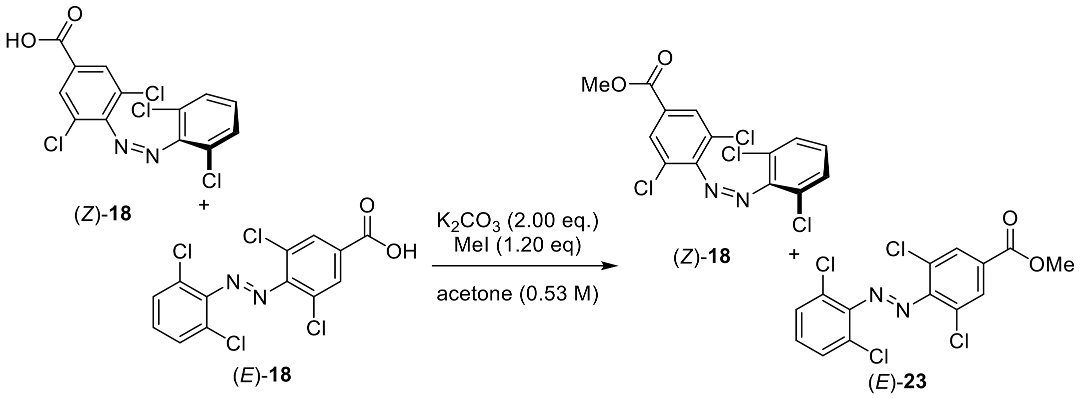


(*E*/*Z*)-3,5-Dichloro-4-((2,6-dichlorophenyl)diazenyl)benzoic acid (**18**, 100 mg, 0.27 mmol, 1.0 eq), $K_2CO_3$ (75.9 mg, 0.55 mmol, 2.0 eq.) and MeI (0.02 mL, 0.32 mmol, 1.2 eq) were dissolved in acetone (0.51 mL) and stirred at 40 °C for 5.5 h. After cooling to rt the reaction mixture was concentrated *in vacuo* and redissolved in EtOAc (80 mL). The organic phase was washed with sat. aq. $NaHCO_3$ (2 × 100 mL) and sat. aq. NaCl (2 × 100 mL), dried over $MgSO_4$, filtered and concentrated *in vacuo.* Purification by flash column chromatography (*n*-hexane/$CH_2Cl_2$ 90:10) afforded (*E*/*Z*)-methyl-3,5-dichloro-4-((2,6-dichlorophenyl)diazenyl)-benzoate ((*E*/*Z*)-**23**, 41.2 mg, 0.11 mmol, 40%) as an isomeric mixture (*E*/*Z* = 96:4 ($^{1}$H NMR)) red solid.

.

***R*f** (*n*-hexane/EtOAc 50:50) = 0.81. (visible)

**$^{1}$H NMR (500 MHz, $(CD_3)_2CO$) *E*-Isomer: δ (ppm)** 8.15 (s, 2H), 7.68 (d, *J* = 8.1 Hz, 2H), 7.55 (d, *J* = 8.1 Hz, 1H), 3.98 (s, 3H). ***Z*-Isomer: δ (ppm)** 7.93 (s, 1H), 7.47 (d, *J* = 8.6 Hz, 1H), 7.42 (d, *J* = 8.6 Hz, 1H), 3.92 (s, 2H).

**$^{13}$C{$^{1}$H} NMR (126 MHz, $(CD_3)_2CO$) *E*-Isomer: δ (ppm)** 164.6, 151.3, 147.8, 132.9, 132.2, 131.2, 130.9, 128.0, 127.4, 53.3.

**HRMS:** (EI) calculated for $C_{14}H_8Cl_4N_2O_2$ [M]: 375.9340 found: 375.9335.

**IR (Diamond-ATR, neat) $\tilde{\nu}$ ($cm^{-1}$)** = 3092 (w), 3074 (w), 2952 (w), 1730 (s), 1562 (m), 1504 (w), 1430 (s), 1382 (m), 1270 (s), 1212 (m), 1200 (m), 1190 (m), 1134 (m), 986 (m), 918 (w), 898 (m), 880 (w), 810 (s), 778 (vs), 760

(vs), 744 (m), 732 (m), 690 (w).

## (*E*/*Z*)-3-Chloro-4-((2-chloro-6-fluorophenyl)diazenyl)-5-fluoro-N-methylbenzamide (24)

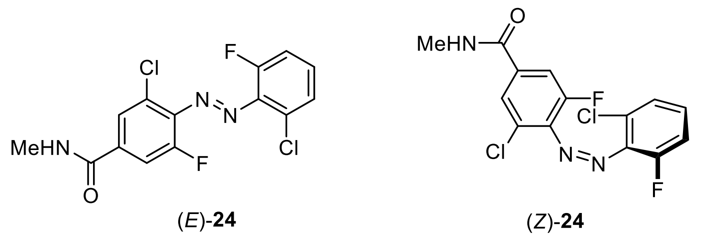


The synthesis to this compound will be published elsewhere.

***R*f** (*n*-hexane/EtOAc 8:2) = 0.10. (visible)

**$^1$H NMR (400 MHz, $(CD_3)_2CO$) *E*-Isomer: δ (ppm)** 8.01 (s, 1H), 7.98 (t, *J* = 1.6 Hz, 1H), 7.82 (dd, *J* = 11.2, 1.6 Hz, 1H), 7.64–7.53 (m, 2H), 7.51–7.43 (m, 1H), 2.95 (d, *J* = 4.6 Hz, 3H). ***Z*-Isomer: δ (ppm)** 7.88 (dd, *J* = 1.6, 1.1 Hz, 1H), 7.64 – 7.58 (m, 1H), 7.50 – 7.45 (m, 1H), 7.07 (ddd, *J* = 10.1, 8.1, 1.6 Hz, 0H), 2.88 (d, *J* = 4.7 Hz, 3H).

**$^{13}$C{$^1$H} NMR (101 MHz, $(CD_3)_2CO$) *E*-Isomer: δ (ppm)** 164.5, 152.9 (d, *J* = 260.4 Hz), 141.4, 139.9, 138.6 (d, *J* = 7.6 Hz), 133.2, 132.4 (d, *J* = 3.1 Hz), 132.0, 127.5 (d, *J* = 3.7 Hz), 125.8 (d, *J* = 3.7 Hz), 117.2, 115.9 (d, *J* = 22.4 Hz), 26.9.

**$^{19}$F{$^1$H} NMR (376 MHz, $(CD_3)_2CO$) *E*-Isomer: δ (ppm)** -125.5, -125.9. ***Z*-Isomer: δ (ppm)** -119.3 (d, *J* = 8.7 Hz), -120.3 (d, *J* = 8.7 Hz).

**HRMS:** (EI) calculated for $C_{14}H_9Cl_2F_2N_3O$ [M]: 343.0091 found: 343.0082.

**IR (Diamond-ATR, neat) $\tilde{\nu}$ (cm$^{-1}$)** = 3284 (m), 3074 (w), 2946 (vw), 1638 (s), 1594 (m), 1556 (s), 1490 (w), 1450 (m), 1416 (m), 1408 (m), 1318 (m), 1256 (m), 1224 (w), 1184 (w), 1160 (w), 1118 (w), 964 (w), 928 (s), 908 (m), 878 (vs), 780 (s), 742 (m), 702 (s), 656 (w).

## (*E*)-Dimethyl 4,4'-(diazene-1,2-diyl)-bis(3-chloro-5-fluorobenzoate) (28)

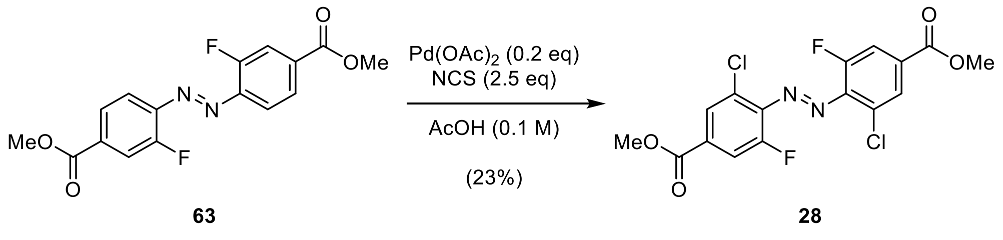


Methyl-3-chloro-4-((2-chloro-6-fluorophenyl)diazenyl)-5-fluorobenzoate (**63,** 163 mg, 0.49 mmol, 1.0 eq.), $Pd(OAc)_2$ (21.8 mg, 0.10 mmol, 0.2 eq.) and NCS (164 mg, 1.23 mmol, 2.5 eq.) were dissolved in AcOH (4.9 mL) and stirred at 120 °C for 7 h. After cooling to rt the reaction mixture was concentrated *in vacuo*. Purification by flash column chromatography (hexane/EtOAc 85:15) afforded (*E*)-dimethyl-4,4'-(diazene-1,2-diyl)-bis(3-chloro-

5-fluorobenzoate) (**28**, 46.0 mg, 0.11 mmol, 23%) as a red solid.

***R*f** (*n*-hexane/EtOAc 75:25) = 0.55. (visible)
**$^1$H NMR (400 MHz, $(CD_3)_2CO$) δ (ppm)** = 8.08 (t, *J* = 1.6 Hz, 2H), 7.92 (dd, *J* = 10.7, 1.6 Hz, 2H), 3.98 (d, *J* = 0.9 Hz, 6H).
**$^{13}C\{^1H\}$ NMR (151 MHz, $(CD_3)_2CO$) δ (ppm)** = 164.5, 152.7 (d, *J* = 261.8 Hz), 142.7 (d, *J* = 10.3 Hz), 134.2 (d, *J* = 8.6 Hz), 132.1 (d, *J* = 2.6 Hz), 128.0 (d, *J* = 3.7 Hz), 117.9 (d, *J* = 22.5 Hz), 110.9, 53.4.
**$^{19}F\{^1H\}$ NMR (376 MHz, $(CD_3)_2CO$) δ** = -124.8.
**HRMS (EI):** calc. for $C_{16}H_{10}O_4N_2Cl_2F_2$ [M]: 401.9986, found: 401.9979.
**IR (Diamond-ATR, neat) $\tilde{\nu}$ (cm$^{-1}$)** = 3442 (vw), 3084 (m), 3002 (w), 2952 (w), 2924 (w), 2848 (w), 1724 (vs), 1570 (m), 1456 (m), 1434 (m), 1414 (s), 1286 (vs), 1224 (vs), 1196 (m), 1180 (s), 1092 (s), 1006 (s), 930 (vs), 906 (s), 892 (m), 878 (m), 800 (m), 762 (vs), 750 (s), 702 (m).

## (*E*/*Z*)-3-Chloro-4-((2-chloro-6-fluorophenyl)diazenyl)-5-fluoroaniline (38)

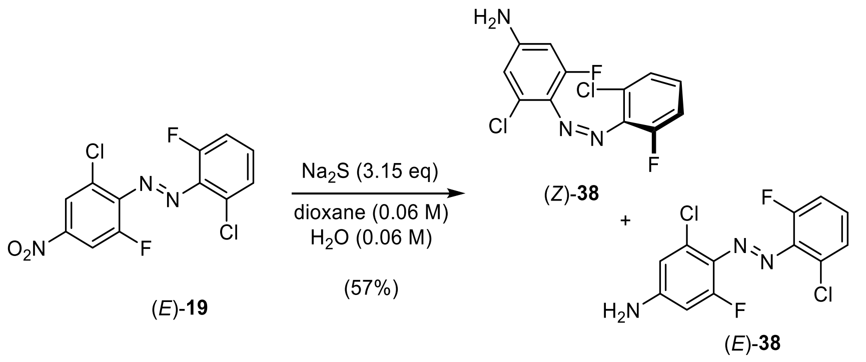


(*E*)-1-(2-Chloro-6-fluoro-4-nitrophenyl)-2-(2-chloro-6-fluorophenyl)diazene (**19**, 320 mg, 0.96 mmol, 1.0 eq.) was dissolved in 1,4-dioxane (16.0 mL) and cooled to 0 °C. A solution of $Na_2S$ (729 mg, 3.03 mmol, 3.15 eq.) in $H_2O$ (16.0 mL) was added at 0 °C. The mixture was warmed to 65 °C and stirred for 2 h. The reaction was allowed to cool to rt, quenched with sat. aq. $NaHCO_3$ (30 mL) and extracted with EtOAc (2 × 150 mL). The combined organic layers were dried over $Na_2SO_4$ and concentrated under reduce pressure. Purification by flash column chromatography afforded (*E*/*Z*)-3-chloro-4-((2-chloro-6-fluorophenyl)diazenyl)-5-fluoroaniline ((*E*/*Z*)-**38**, 167 mg, 0.551 mmol, 57%) as an isomeric mixture (*E*/*Z* = 80:20 ($^{19}F\{^1H\}$ NMR)) red solid.

***R*f** = (cyclohexane/$CH_2Cl_2$ 4:6) = 0.5. (visible light)
**$^1$H NMR (400 MHz, $CD_3OD$) *E*-Isomer: δ (ppm)** 7.44–7.24 (m, 2H), 7.18 (ddd, *J* = 10.7, 8.2, 1.4 Hz, 1H), 6.68 (dd, *J* = 2.4, 1.1 Hz, 1H), 6.37 (dd, *J* = 14.2, 2.4 Hz, 1H). ***Z*-Isomer: δ (ppm)** 7.34–7.22 (m, 2H), 6.99 (ddd, *J* = 9.6, 8.2, 1.1 Hz, 1H), 6.62 (dd, *J* = 2.2, 1.1 Hz, 1H), 6.00 (dd, *J* = 13.0, 2.2 Hz, 1H).
**$^{13}C\{^1H\}$ NMR (126 MHz, $CD_3OD$) *E*-Isomer: δ (ppm)** 157.7, 155.6, 155.1 (d, *J* = 72.5 Hz), 155.0, 153.4, 141.8 (d, *J* = 10.4 Hz), 139.1 (d, *J* = 6.9 Hz), 130.0 (d, *J* = 9.4 Hz), 127.0 (d, *J* = 3.8 Hz), 116.6 (d, *J* = 20.7 Hz), 111.8, 100.7 (d, *J* = 23.6 Hz).

**$^{19}$F{$^{1}$H} NMR (376 MHz, $CD_3OD$) *E*-Isomer: δ (ppm)** -120.4, -127.8. ***Z*-Isomer: δ (ppm)** -117.7 (d, *J* = 7.7 Hz), -120.4(d, *J* = 7.7 Hz).

**HRMS:** calculated for [$C_{12}H_6Cl_2F_2N_3$]−(M−H$^+$): 299.9912 found: 299.9912.

**IR (Diamond-ATR, neat) $\tilde{\nu}$ (cm$^{-1}$) =** 2922 (s), 2853 (m), 2611 (vw), 2451 (w), 1606 (s), 1570 (m), 1540 (m), 1492 (w), 1452 (s), 1415 (w), 1352 (s), 1237 (m), 1198 (m), 1175 (m), 1152 (w), 1126 (m), 994 (m), 937 (w), 892 (s), 874 (m), 830 (s), 780 (vs), 733 (vs).

## (*E*/*Z*)-*N*-(3-Chloro-4-((2-chloro-6-fluorophenyl)diazenyl)-5-fluorophenyl)acetamide (39)

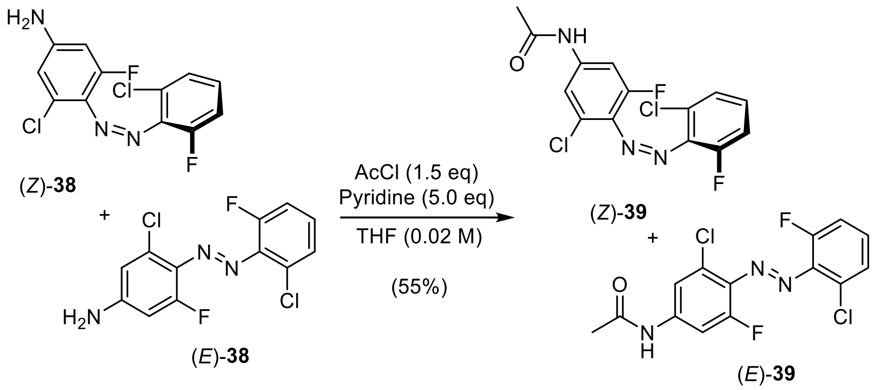


(*E*/*Z*)-3-Chloro-4-((2-chloro-6-fluorophenyl)diazenyl)-5-fluoroaniline ((*E*/*Z*)-**38**, 50.0 mg, 0.17 mmol, 1.0 eq.) and pyridine (65.2 mg, 0.83 mmol, 5.0 eq.) were dissolved in THF (11.0 mL) and cooled to 0 °C. A stock solution of AcCl (18.0 µL, 0.25 mmol, 1.5 eq.) in THF (0.1 mL) was added to the mixture at 0 °C. The reaction was allowed to warm to rt and was stirred for 2 h. After addition of sat. aq. $NH_4Cl$ (50 mL), the mixture was extracted with EtOAc (2 × 150 mL). The combined organic layers were dried over $Na_2SO_4$ and concentrated under reduce pressure. Purification by flash column chromatography afforded (*E*/*Z*)-*N*-(3-chloro-4-((2-chloro-6-fluorophenyl)diazenyl)-5-fluorophenyl)acetamide ((*E*/*Z*)-**39**, 31.2 mg, 0.09 mmol, 55%) as a isomeric mixture (*E*/*Z* = 93:7 ($^{19}$F{$^{1}$H} NMR)) red solid.

***R*f** = (MeOH/$CH_2Cl_2$ 5:95) = 0.5. (visible light)

**$^{1}$H NMR (400 MHz, $(CD_3)_2CO$) *E*-Isomer: δ (ppm)** 7.81 (t, *J* = 2.1 Hz, 1H), 7.70 (dd, *J* = 13.6, 2.1 Hz, 1H), 7.54–7.48 (m, 2H), 7.38–7.30 (m, 1H), 2.16 (s, 3H).

**$^{13}$C{$^{1}$H} NMR (101 MHz, $(CD_3)_2CO$) *E*-Isomer: δ (ppm)** 170.0, 155.3, 153.4 (d, *J* = 259.2 Hz), 152.7, 143.8 (d, *J* = 13.0 Hz), 140.5 (d, *J* = 9.3 Hz), 135.1 (d, *J* = 5.3 Hz), 131.9 (d, *J* = 9.3 Hz), 131.6 (d, *J* = 3.0 Hz), 127.2 (d, *J* = 3.8 Hz), 116.9 (d, *J* = 20.4 Hz), 106.8 (d, *J* = 7.2 Hz), 106.6 (d, *J* = 7.2 Hz), 24.5.

**$^{19}$F{$^{1}$H} NMR (376 MHz, $(CD_3)_2CO$) *E*-Isomer: δ (ppm)** -121.3 (d, *J* = 6.2 Hz), -125.7. ***Z*-Isomer: δ (ppm)** -117.1 (d, *J* = 8.1 Hz), -119.4 (d, *J* = 8.1 Hz).

**HRMS:** (EI) calculated for $C_8H_6ClFN_3O$: 214.0183 found: 214.0175.

**IR (Diamond-ATR, neat) $\tilde{\nu}$ (cm$^{-1}$) =** 2922 (m), 2852 (w), 2427 (vw), 2326 (vw), 1669 (m), 1647 (m), 1594 (vs), 1568 (m), 1537 (w), 1469 (s), 1448 (vs), 1382 (vs), 1306 (m), 1269 (m), 1256 (s), 1224 (m), 1187 (w), 1140 (m), 1084 (w), 1031 (w), 1000 (w), 982 (m), 936 (m), 921 (w), 899 (s), 886 (vs), 854 (vs), 781 (vs), 740 (s), 708 (m), 660 (w).

**(*E*/*Z*)-Methyl-3-chloro-4-((2-chloro-6-fluoro-4-methoxyphenyl)diazenyl)-5-fluorobenzoate (46)**

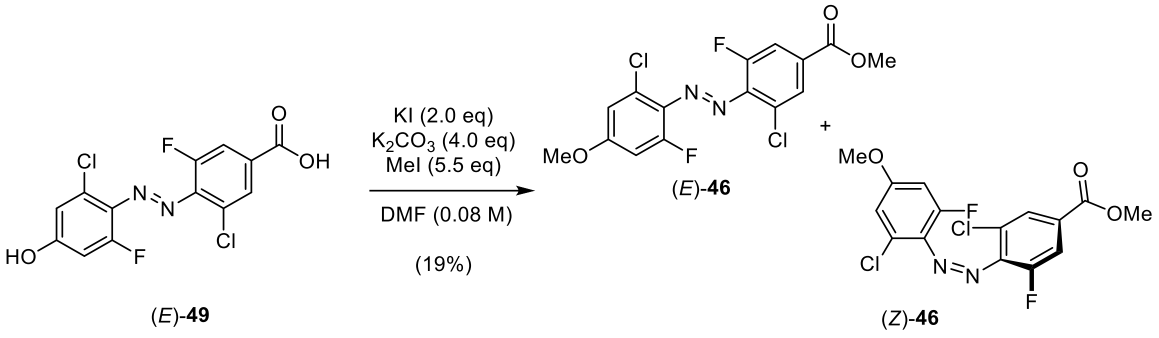


(*E*)-3-Chloro-4-((2-chloro-6-fluoro-4-hydroxyphenyl)diazenyl)-5-fluorobenzoic acid (**49**, 23.5 mg, 0.07 mmol, 1.0 eq), KI (22.5 mg, 0.14 mmol, 2.0 eq) and $K_2CO_3$ (38.7 mg, 0.28 mmol, 4.0 eq) were dissolved in DMF (0.88 mL) and stirred at 50 °C for 15 min. MeI (0.02 mL, 0.39 mmol, 5.5 eq) in DMF (0.18 mL) was added over 5 min and the reaction mixture was stirred at 90 °C for 40 h. After cooling to rt the reaction mixture was poured into $H_2O$ (50 mL), acidified with 2 M aq. HCl (20 mL, pH4) and extracted with $CH_2Cl_2$ (3 × 200 mL). The combined organic phases were dried over $Na_2SO_4$ and the solvent was removed *in vacuo.* Purification via flash column chromatography (*n*-hexane/EtOAc 9:1) afforded (*E*/*Z*)-methyl-3-chloro-4-((2-chloro-6-fluoro-4-methoxyphenyl)diazenyl)-5-fluorobenzoate ((*E*/*Z*)-**46**, 3.8 mg, 0.01 mmol, 19%) as an isomeric mixture (*E*/*Z* = 92:8 ($^{19}F\{^1H\}$ NMR)) orange solid.

***R*f** (*n*-hexane/EtOAc 9:1) = 0.37. (visible)

**$^1$H NMR (400 MHz, (CD3)2CO) *E*-Isomer: δ (ppm)** 8.02 (t, *J* = 1.6 Hz, 1H), 7.86 (dd, *J* = 10.7, 1.6 Hz, 1H), 7.17 (dd, *J* = 2.7, 1.6 Hz, 1H), 6.99 (dd, *J* = 13.3, 2.7 Hz, 1H), 4.01 (s, 3H), 3.96 (s, 3H). ***Z*-Isomer: δ (ppm)** 7.95–7.93 (m, 1H), 7.61 (dd, *J* = 10.1, 1.5 Hz, 1H), 7.09–7.06 (m, 1H), 6.64 (dd, *J* = 12.3, 2.5 Hz, 1H), 3.91 (s, 3H), 3.86 (s, 3H).

**$^{13}C\{^1H\}$ NMR (101 MHz, (CD3)2CO) *E*-Isomer: δ (ppm)** 164.7 (d, *J* = 2.7 Hz), 163.9, 155.0 (d, *J* = 265.0 Hz), 137.1, 132.7, 131.0, 127.8 (d, *J* = 3.6 Hz), 117.7 (d, *J* = 22.6 Hz), 116.9 (d, *J* = 23.0 Hz), 113.6 (d, *J* = 2.7 Hz), 113.4 (d, *J* = 3.0 Hz), 103.4 (d, *J* = 24.1 Hz), 102.0, 57.2, 53.3.

**$^{19}F\{^1H\}$ NMR (376 MHz, (CD3)2CO) *E*-Isomer: δ (ppm)** -120.0, -125.8. ***Z*-Isomer: δ (ppm)** -117.4 (d, *J* = 8.3 Hz), -119.3 (d, *J* = 8.3 Hz).

**HRMS:** (EI) calculated for $C_{15}H_{10}Cl_2F_2N_2O_3$ [M]: 373.0037 found: 373.0029.

**IR (Diamond-ATR, neat) $\tilde{\nu}$ (cm$^{-1}$)** = 3088 (w), 2952 (w), 2846 (w), 1732 (vs), 1606 (s), 1568 (s), 1478 (m), 1452 (m), 1426 (s), 1408 (s), 1322 (s), 1282 (vs), 1224 (vs), 1192 (m), 1170 (m), 1136 (s), 1094 (s), 1036 (m), 1006 (m), 980 (s), 942 (vs), 908 (m), 886 (s), 850 (s), 824 (s), 796 (w), 762 (s), 744 (m), 728 (m).

### ($E$)-4-((4-Amino-2,6-dichlorophenyl)diazenyl)-3,5-dichlorobenzenesulfonamide (48)

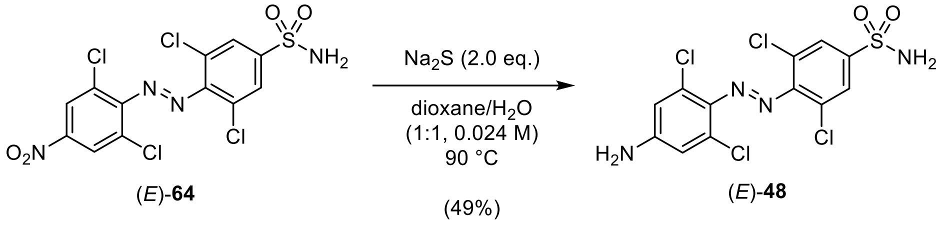


($E$)-3,5-Dichloro-4-((2,6-dichloro-4-nitrophenyl)diazenyl)benzenesulfonamide (**64**, 50.0 mg, 0.133 mmol, 1.0 eq) was dissolved in dioxane/$H_2O$ (1:1, 5.5 mmol) and $Na_2S$ hydrate (≥60%, 34.1 mg, 262 mmol, 2.0 eq) was added before heating to 90 °C for 2 h 30 min. After cooling to rt, the mixture was diluted with EtOAc (75 mL) and washed with sat. aq. NaCl (20 mL). The organic layer was dried over $Na_2SO_4$ and the solvent was removed *in vacuo*. Purification *via* flash column chromatography (petane/acetone 7:3) afforded ($E$)-4-((4-amino-2,6-dichlorophenyl)diazenyl)-3,5-dichlorobenzenesulfonamide (**48**, 23.0 mg, 55.5 µmol, 49%) as a bright brown gum.

***R*f** (pentane/acetone 7:3) = 0.22 (visible)

**tR** (reverse-phase semi-preparative HPLC, MeCN:$H_2O$:HCOOH = 35:65:1% → 65:35:1% over 30 min) = 21.93 min.

**$^1$H NMR (400 MHz, $(CD_3)_2CO$) δ (ppm)** = 7.98 (s, 2H), 6.92 (s, 2H), 6.91 (s, 2H), 6.21 (s, 2H).

**$^{13}$C{$^1$H} NMR (101 MHz, $(CD_3)_2CO$) δ (ppm)** = 153.5, 151.9, 144.8, 135.6, 133.7, 127.7, 127.6, 115.2.

**HRMS (ESI):** calc. for $C_{12}H_9Cl_4N_4O_2S^+$ [M+H$^+$]$^+$: 412.9195, found: 412.9200.

**IR (Diamond-ATR, neat) $\tilde{\nu}$ (cm$^{-1}$)** = 3420 (vw), 3335 (w), 3255 (vw), 3088 (vw), 1637 (w), 1598 (m), 1550 (w), 1476 (w), 1427 (w), 1413 (w), 1375 (w), 1330 (s), 1283 (m), 1210 (w), 1162 (vs), 1125 (m), 1073 (m), 920 (m), 901 (m), 877 (m), 853 (m), 845 (m), 813 (s), 793 (m), 749 (m), 735 (m).

### ($E$)-3-Chloro-4-((2-chloro-6-fluoro-4-hydroxyphenyl)diazenyl)-5-fluorobenzoic acid (49)

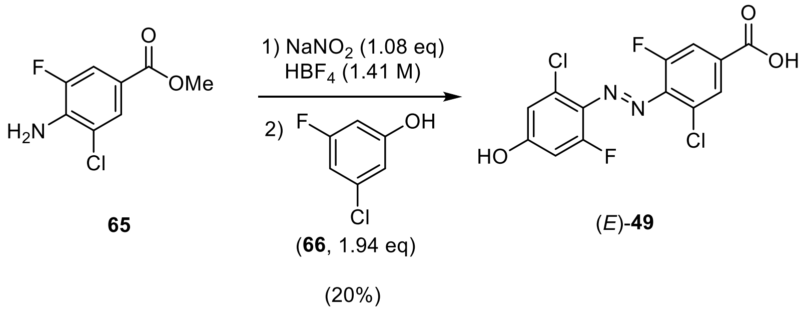


Methyl 4-amino-3-chloro-5-fluorobenzoate (**65**, 103 mg, 0.50 mmol, 1.0 eq) was dissolved in $HBF_4$ (0.35 mL) and cooled to -15 °C. $NaNO_2$ (37.3 mg, 0.54 mmol, 1.08 eq) was added and the reaction stirred at -15 °C for 10 min. After addition of 3-chloro-5-fluorophenol (**66**, 142 mg, 0.97 mmol, 1.94 eq) the reaction was stirred at rt for 4 h. 2 M aq. NaOH (20 mL) is added and the aqueous phase is washed with EtOAc (2 × 50 mL) and acidified with 2 M aq. HCl (30 mL, pH1). The precipitate is filtered off and purified by automated reversed phase column

chromatography ($H_2O$/MeCN), which afforded (*E*)-3-chloro-4-((2-chloro-6-fluoro-4-hydroxyphenyl)diazenyl)-5-fluorobenzoic acid (**49**, 34.8 mg, 0.10 mmol, 20%) as a red solid.

***R*f** ($CH_2Cl_2$/MeOH 8:2) = 0.51. (visible)
**$^1$H NMR (400 MHz, $(CD_3)_2CO$) δ (ppm)** = 10.15 (s, 1H), 8.03 (t, *J* = 1.6 Hz, 1H), 7.86 (dd, *J* = 10.7, 1.6 Hz, 1H), 7.05 (dd, *J* = 2.6, 1.6 Hz, 1H), 6.81 (dd, *J* = 13.1, 2.6 Hz, 1H).
**$^{13}$C{$^1$H} NMR (126 MHz, $(CD_3)_2CO$) δ (ppm)** = 165.0, 162.4, 156.3, 154.1, 152.1, 143.6, 137.4, 132.8, 130.8, 128.0, 117.9 (d, *J* = 22.4 Hz), 114.7, 104.5 (d, *J* = 23.2 Hz).
**$^{19}$F{$^1$H} NMR (376 MHz, ($CDCl_3$)) δ (ppm)** = -119.9, -126.1.
**HRMS (ESI):** calc. for $C_{13}H_5O_3Cl_2N_2F_2^+$ [M-H]$^+$: 344.9651, found: 344.9646.
**IR (Diamond-ATR, neat) $\tilde{\nu}$ (cm$^{-1}$)** = 3316 (vw), 1704 (m), 1636 (m), 1606 (m), 1576 (m), 1526 (s), 1486 (s), 1442 (s), 1398 (vs), 1294 (s), 1224 (vs), 1208 (s), 1186 (s), 1166 (s), 1132 (s), 1092 (s), 1004 (m), 980 (m), 948 (s), 878 (vs), 866 (s), 848 (s), 836 (s), 824 (s), 802 (s), 768 (vs), 744 (s), 706 (s), 680 (s).

## (*E*/*Z*)-1-(1-(4-(4-((4-Butyl-2-chloro-6-fluorophenyl)diazenyl)-3-chloro-5-fluorophenyl)butanoyl)-piperidin-4-yl)-1,3-dihydro-2*H*-benzo[*d*]imidazol-2-one (dfdc-OptoBI-1)

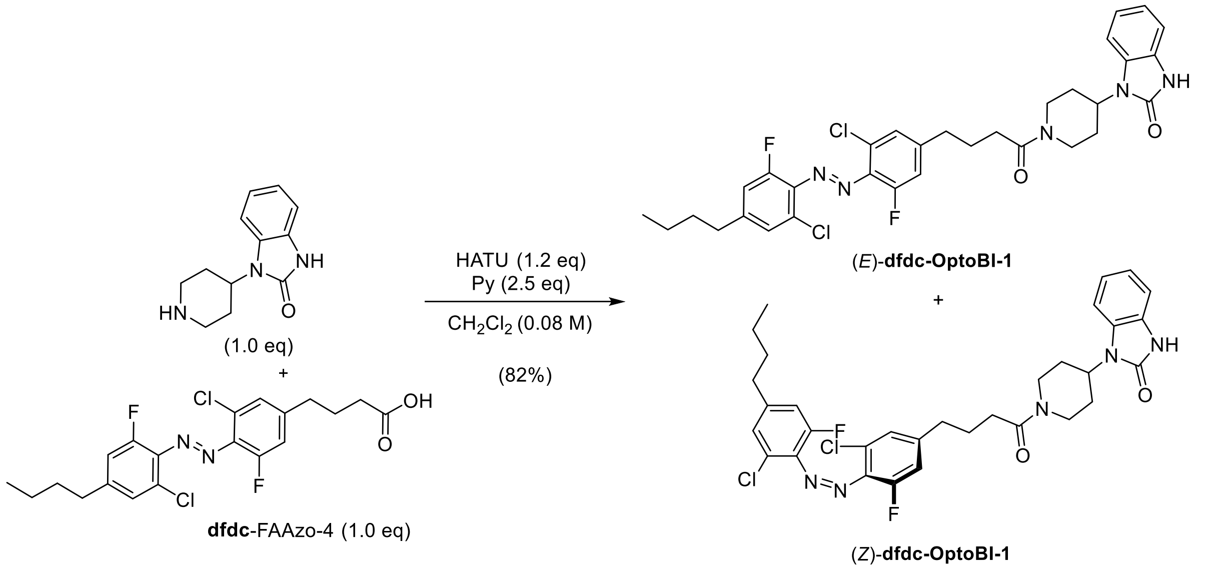


**Dfdc**-FAAzo-4 (198 mg, 0.46 mmol, 1.0 eq) and HATU (210 mg, 0.55 mmol, 1.2 eq) were dissolved in anh. $CH_2Cl_2$ (6.0 ml), followed by dropwise addition of anh. pyridine (92.7 µl, 1.2 mmol, 2.5 eq). The reaction mixture was stirred for 10 min at rt, followed by addition of 1-(piperidin-4-yl)-1,3-dihydro-2*H*-benzo[*d*]imidazol-2-one (100 mg, 0.46 mmol, 1.0 eq) and stirred for 22 h. The reaction mixture was washed with saturated $NaHCO_3$ solution (1 × 30 mL), 3% $KHSO_4$ solution (1 × 30 mL) and extracted with $CH_2Cl_2$ (3 × 30 mL). The combined organic phases were washed with sat. aq. NaCl (2 × 30 mL), dried over $Na_2SO_4$ and the solvent was removed *in vacuo*. Purification via column chromatography ($CH_2Cl_2$/MeOH 97:3) afforded (*E/Z*)-1-(1-(4-(4-((4-butyl-2-chloro-6-fluorophenyl)diazenyl)-3-chloro-5-fluorophenyl)butanoyl)piperidin-4-yl)-1,3-dihydro-2H-benzo[*d*]imidazol-2-one (**dfdc**-OptoBI-1, 238 mg, 0.38 mmol, 82%) as an isomeric mixture (*E/Z* = 3:1 from $^{19}$F NMR) orange solid.

***R*f** (*n*-hexane/EtOAc 20:80) = 0.21 (visible)

**$^1$H NMR (400 MHz, CDCl$_3$) *E*-Isomer: δ (ppm)** 7.21 (d, *J* = 20.6 Hz, 2H), 7.09 – 7.05 (m, 4H), 6.98 (ddd, *J* = 23.3, 11.5, 1.7 Hz, 2H), 4.89 (s, 1H), 4.56 – 4.48 (m, 2H), 3.99 (s, 1H), 3.19 (s, 1H), 2.83 – 2.72 (m, 2H), 2.69 – 2.59 (m, 2H), 2.47 – 2.29 (m, 3H), 2.12 – 1.95 (m, 2H), 1.69 – 1.51 (m, 2H), 1.45 – 1.23 (m, 2H), 1.01 – 0.87 (m, 3H). ***Z*-Isomer: δ (ppm)** 6.65 (dd, J = 25.1, 9.5 Hz, 0.5H). All remaining *Z*-isomer $^1$H peaks overlap with *E*-isomer signals and are not listed separately.

**$^{13}$C NMR (126 MHz, CDCl$_3$) δ (ppm)** 170.6, 154.3, 153.8, 151.7, 147.5 (d, *J* = 8.9 Hz), 146.0 (d, *J* = 8.5 Hz), 129.2, 127.7, 126.3 (d, *J* =37.8 Hz), 126.2 (d, *J* = 25.2 Hz), 121.6 (d, *J* = 3.4 Hz), 115.9 (d, *J* = 8.9 Hz), 115.8, 109.5 (d, *J* = 42.1 Hz), 50.9, 45.2, 41.6, 35.4, 35.0, 33.0, 32.1, 29.9, 29.1, 22.3, 14.0 (d, *J* = 1.8 Hz).

**$^{19}$F NMR (376 MHz, CDCl$_3$) *E*-Isomer: δ (ppm)** -123.5, -123.7. ***Z*-Isomer: δ (ppm)** 117.5 (d, *J* = 7.7 Hz), -118.0 (d, *J* = 8.1 Hz)

**HRMS (EI):** calc. for $C_{32}H_{33}Cl_2F_2N_5O_2$ $[M]^+$: 627.1979, found: 627.1972.

## Precursor

### (*E*)-1,2-Bis(1-fluoronaphthalen-2-yl)diazene (58)

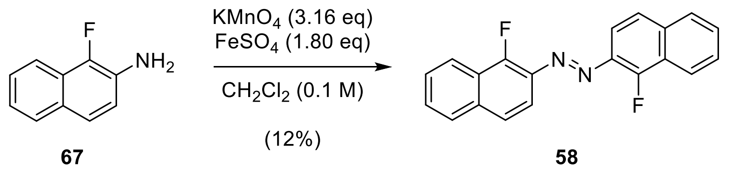


1-Fluoronaphthalen-2-amine[11] (**67**, 160 mg, 0.99 mmol, 1.0 eq.), $KMnO_4$ (496 mg, 3.14 mmol, 3.16 eq.) and $FeSO_4$·7 $H_2O$ (495 mg, 1.78 mmol, 1.8 eq.) were dissolved in $CH_2Cl_2$ (9.9 mL) and stirred at 43 °C for 17 h. The forming precipitate was filtered off through celite and the filtrate concentrated *in vacuo*. Purification by flash column chromatography yielded (*E*)-1,2-bis(1-fluoronaphthalen-2-yl)diazene (**58**, 19.9 mg, 0.06 mmol, 12%) as an orange solid.

***R*f** (*n*-hexane/$CH_2Cl_2$ 9:1) = 0.63. (visible)

**$^1$H NMR (400 MHz, CDCl$_3$) δ (ppm) =** 8.33 (dt, *J* = 7.1, 3.6 Hz, 1H), 8.10 (dd, *J* = 9.0, 7.1 Hz, 1H), 7.95–7.85 (m, 1H), 7.67 (d, *J* = 9.0 Hz, 1H), 7.66–7.60 (m, 2H).

**$^{13}$C{$^1$H} NMR (101 MHz, (CDCl$_3$)) δ (ppm)** = 159.1, 156.4, 136.3 (d, *J* = 5.1 Hz), 128.8, 128.0 (d, *J* = 3.3 Hz), 127.1, 124.6 (d, *J* = 15.0 Hz), 124.3 (d, *J* = 4.0 Hz), 122.2 (d, *J* = 6.0 Hz), 115.4.

**$^{19}$F{$^1$H} NMR (376 MHz, (CDCl$_3$)) δ (ppm)** = -131.9.

**HRMS:** (EI) calculated for $C_{20}H_{12}N_2F_2$ 318.0969 found: 318.0971.

### (*E*)-1 (2-Fluoro-4-nitrophenyl)-2-(2-fluorophenyl)diazene (59)

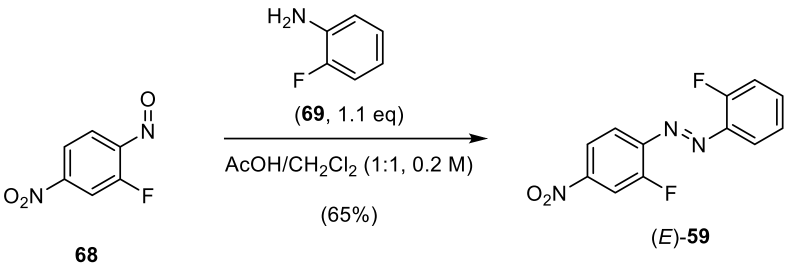


2-Fluoro-4-nitro-1-nitrosobenzene (**68**, 2.30 g, 13.5 mmol, 1.00 eq.) and 2-fluoroaniline (**69**, 1.08 g, 14.9 mmol, 1.10 eq.) were dissolved in AcOH/$CH_2Cl_2$ (68.0 mL, 1:1) at rt. The mixture was warmed to 40 °C and stirred vigorously for 20 h. The solvent mixture was removed under reduced pressure and, the residue was redissolved in $CH_2Cl_2$ (150 mL), followed by subsequent washing with $H_2O$ (100 mL) and sat. aq. $NaHCO_3$ (100 mL). The organic layer was dried over $Na_2SO_4$ and concentrated under reduced pressure. Purification by flash column chromatography afforded (*E*)-1 (2-fluoro-4-nitrophenyl)-2-(2-fluorophenyl)diazene (**59**, 2.31 g, 8.78 mmol, 65%) as a red solid.

***R*f** (*n*-hexane/$CH_2Cl_2$ 1:1) = 0.24.
**$^1$H NMR (400 MHz, $(CD_3)_2CO$) δ (ppm) =** 8.31 (dd, *J* = 10.2, 2.4 Hz, 1H), 8.24 (ddd, *J* = 9.09, 2.4, 1.2 Hz, 1H), 7.97 (dd, *J* = 9.09, 7.7 Hz, 1H), 7.83 (td, *J* = 7.7, 1.8 Hz, 1H), 7.73 (dddd, *J* = 9.0, 6.9, 5.1, 1.8 Hz, 1H), 7.48 (ddd, *J* = 10.9, 8.4, 1.3 Hz, 1H), 7.40 (tdd, *J* = 7.4, 1.3, 0.7 Hz, 1H).
**$^{13}$C{$^1$H} NMR (101 MHz, $(CD_3)_2CO$ ) δ =** 161.7 (d, *J* = 259.0 Hz), 159.9 (d, *J* = 260.5 Hz), 150.8 (d, *J* = 8.6 Hz), 145.0 (d, *J* = 7.3 Hz), 141.5 (d, *J* = 6.7 Hz), 136.2 (d, *J* = 8.6 Hz), 125.9 (d, *J* = 3.7 Hz), 121.1 (d, *J* = 4.5 Hz), 119.9 (d, *J* = 5.5 Hz), 118.4 (d, *J* = 4.5 Hz), 118.3, 114.3 (d, *J* = 16.3 Hz).
**$^{19}$F{$^1$H} NMR (376 MHz, $(CD_3)_2CO$) δ (ppm) = -**121.6, -124.2.
**HRMS:** (EI) calculated for $C_{12}H_7O_2N_3F_2$ 263.0506 found: 263.0511.

### (*E*)-3-Fluoro-4-((2-fluoro-4-nitrophenyl)diazenyl)benzoic aciddiazene (60)

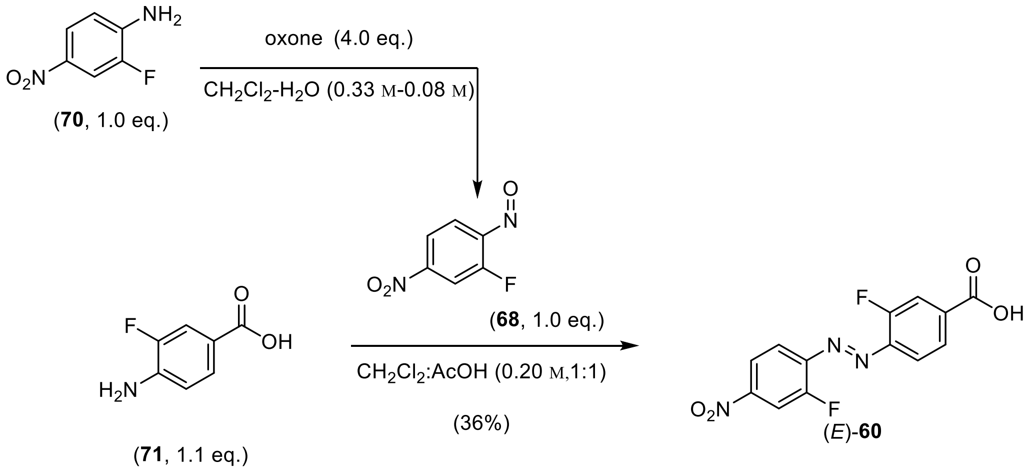

To a solution of 2-fluoro-4-nitroaniline (**70,** 8.00 g, 51.2 mmol, 1.0 eq.) in $CH_2Cl_2$ (155 mL) oxone (63.0 g, 205 mmol, 4.0 eq.) in $H_2O$ (617 mL) was added. After stirring at rt for 16 h the reaction mixture $CH_2Cl_2$ and $H_2O$ were separated and the aqueous layer was extracted with $CH_2Cl_2$ (2 × 250 mL). The combined organic layers were washed with 2 M aq. HCl (2 × 300 mL), sat. aq. $NaHCO_3$ (2 × 300 mL) and sat. aq. NaCl (2 × 300 mL), dried over $Na_2SO_4$, filtered and concentrated *in vacuo.* The obtained 2-fluoro-4-nitro-1-nitrosobenzene (**68**) was directly used without further purification.

2-Fluoro-4-nitro-1-nitrosobenzene (**68**, 7.70 g, 45.3 mmol, 1.0 eq.) and 3-amino-4-fluorobenzoic acid (**71,** 7.73 g, 49.8 mmol, 1.1 eq.) were dissolved in $CH_2Cl_2$ (113 mL) and AcOH (113 mL). After stirring at 40 °C for 21 h the reaction mixture was concentrated *in vacuo*. Purification by recrystallization from EtOH afforded (*E*)-3-fluoro-4-((2-fluoro-4-nitrophenyl)diazenyl)benzoic aciddiazene (**60**, 5.00 g, 16.3 mmol, 36%) as a red solid.

***R*f** (*n*-hexane/EtOAc/AcOH 49.9:50:0.1) = 0.21. (visible)
**$^1$H NMR (400 MHz, $(CD_3)_2CO$) δ (ppm) =** 8.36 (dd, *J* = 10.2, 2.4 Hz, 1H), 8.28 (ddd, *J* = 8.8, 2.4, 1.3 Hz, 1H), 8.08–7.99 (m, 3H), 7.92 (t, *J* = 8.8, 7.2 Hz, 1H).
**$^{13}$C{$^1$H} NMR (101 MHz, $(CD_3)_2CO$) δ (ppm) =** 165.7 (d, *J* = 2.4 Hz), 162.2, 160.6 (d, *J* = 190.1 Hz), 158.9, 144.9 (d, *J* = 7.6 Hz), 143.9 (d, *J* = 7.6 Hz), 137.0 (d, *J* = 7.6 Hz), 127.0 (d, *J* = 3.9 Hz), 121.2 (d, *J* = 3.9 Hz), 120.0, 119.4 (d, *J* = 21.5 Hz), 119.0, 114.3 (d, *J* = 25.1 Hz)
**$^{19}$F{$^1$H} NMR (376 MHz, $(CD_3)_2CO$) δ (ppm) =** -122.0, -124.6.
**HRMS (ESI):** calcd. for $C_{13}H_6O_4N_3F_2$ [M-H]: 306.03324, found: 306.03283.

### (*E*/*Z*)-Methyl-3-fluoro-4-((2-fluorophenyl)diazenyl)benzoate (61)

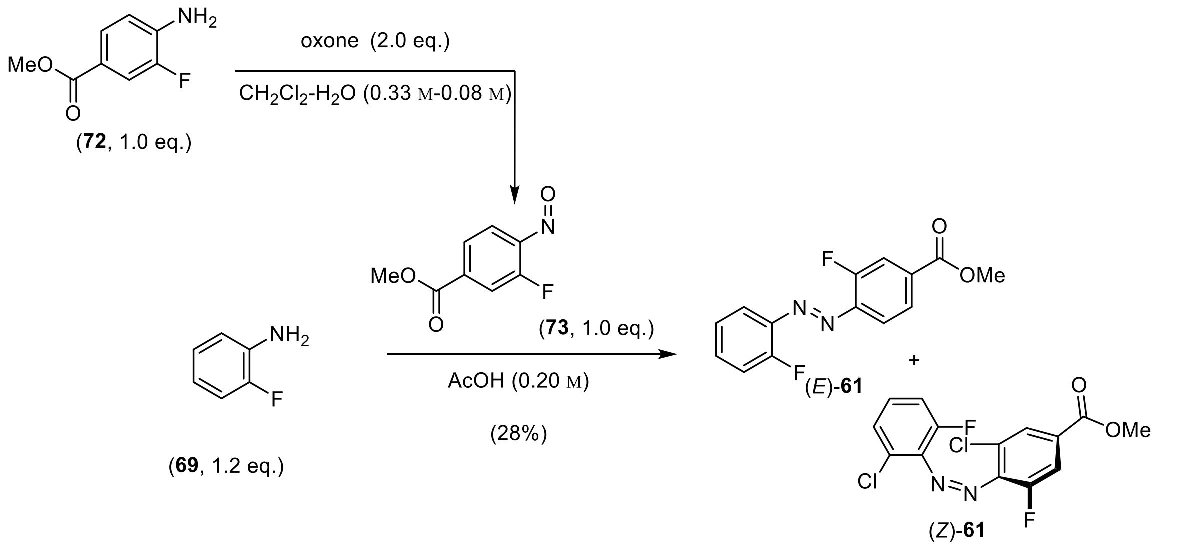


To a solution of methyl 4-amino-3-fluorobenzoate (**72**, 1 g, 5.90 mmol, 1.0 eq.) in $CH_2Cl_2$ (18 mL) oxone (7.27 g, 11.8 mmol, 4.0 eq.) in $H_2O$ (74 mL) was added. After stirring at rt for 2 h the reaction mixture was partitioned between $CH_2Cl_2$ and $H_2O$ and the aqueous layer was extracted with $CH_2Cl_2$ (2 × 60 mL). The combined organic layers were washed with 1 M aq. HCl (2 × 100 mL), sat. aq. $NaHCO_3$ (2 × 100 mL) and sat. aq. NaCl (2 × 100 mL), dried over $MgSO_4$, filtered and concentrated *in vacuo.* The obtained 2- methyl 3-fluoro-4-nitrosobenzoate (**73**) was directly used without further purification.

Methyl 3-fluoro-4-nitrosobenzoate (**73**, 972 mg, 5.31 mmol, 1.0 eq.) and 2-fluoroaniline (**69,** 708 mg, 6.37 mmol, 1.1 eq.) were dissolved in AcOH (27 mL). After stirring at rt for 18 h the reaction mixture was diluted with hexane (300 mL) and washed with $H_2O$ (2 × 300 mL). The organic layer was dried over $MgSO_4$, filtered and concentrated *in vacuo*. Purification by flash column chromatography (*n*-hexane/$CH_2Cl_2$ 75:25) afforded (*E*/*Z*)-methyl-3-fluoro-4-((2-fluorophenyl)diazenyl)benzoate ((*E*/*Z*)-**61**, 405 mg, 1.47 mmol, 28%) as an isomeric mixture (*E*/*Z* = 97:3 ($^{19}F\{^1H\}$ NMR)) orange solid.

***R*f** (*n*-hexane/EtOAc 75:25) = 0.44. (visible)

**$^1$H NMR (400 MHz, $CDCl_3$) *E*-Isomer: δ (ppm)** 7.98–7.87 (m, 2H), 7.85–7.77 (m, 2H), 7.55–7.47 (m, 1H), 7.32–7.20 (m, 2H), 3.96 (s, 3H). ***Z*-Isomer: δ (ppm)** 7.77–7.70 (m, 1H), 7.62 (dd, *J* = 10.2, 1.6 Hz, 1H), 7.20–7.14 (m, 1H), 7.10–7.03 (m, 1H), 7.01–6.90 (m, 3H), 3.88 (d, *J* = 0.8 Hz, 3H).

**$^{13}C\{^1H\}$ NMR (101 MHz, $CDCl_3$) *E*-Isomer: δ (ppm)** 165.6 (d, *J* = 2.8 Hz), 162.2, 160.3 (d, *J* = 150.5 Hz), 158.5, 143.5 (d, *J* = 7.3 Hz), 141.0 (d, *J* = 6.6 Hz), 134.1, 134.0 (d, *J* = 8.0 Hz), 125.8 (d, *J* = 3.8 Hz), 124.6 (d, *J* = 3.8 Hz), 118.6 (d, *J* = 21.8 Hz), 118.0 (d, *J* = 23.7 Hz), 117.4 (d, *J* = 19.6 Hz), 52.8.

**$^{19}F\{^1H\}$ NMR (376 MHz, $CDCl_3$) *E*-Isomer: δ (ppm)** -123.2, -123.3. ***Z*-Isomer: δ (ppm)** -120.9, -121.3.

**HRMS (EI):** calc. for $C_{14}H_{10}O_2N_2F_2$ [M]: 276.0710, found: 276.0707.

## (*E*/*Z*)-3,5-Dichloro-4-((2,6-dichlorophenyl)diazenyl)benzoic acid (18)

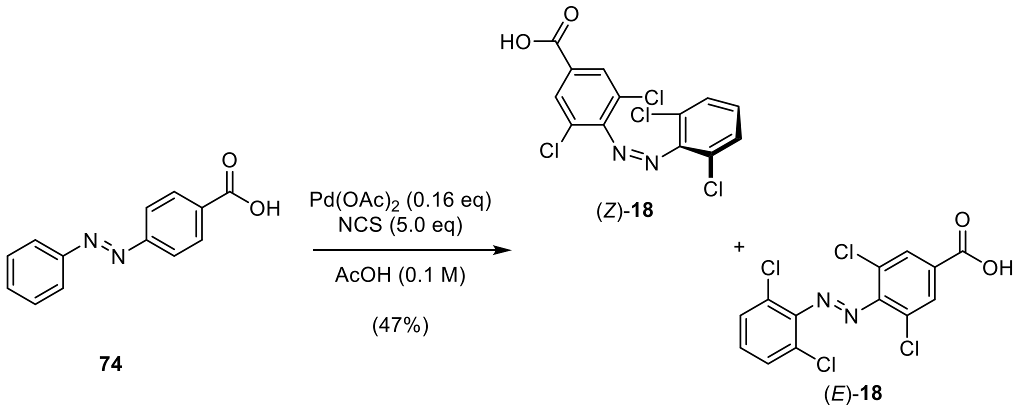


4-(Phenyldiazenyl)benzoic acid (**74**, 500 mg, 2.21 mmol, 1.0 eq.), $Pd(OAc)_2$ (74.4 mg, 0.33 mmol, 0.16 eq.) and NCS (1.48 g, 11.1 mmol, 5.0 eq.) were dissolved in AcOH (22.1 mL) and stirred at 120 °C for 4 h. After cooling to rt the residue was concentrated *in vacuo*. Purification by flash column chromatography ($CH_2Cl_2$ 100) afforded (*E*/*Z*)-3,5-dichloro-4-((2,6-dichlorophenyl)diazenyl)benzoic acid (**18**, 377 mg, 1.04 mmol, 47%) as an isomeric mixture (*E*/*Z* = 68:32 ($^1$H NMR)) orange-brown solid.

***R*f** (*n*-hexane/EtOAc 75:25) = 0.21. (visible)

**$^1$H NMR (400 MHz, $(CD_3)_2CO$) *E*-Isomer: δ (ppm)**8.17 (s, 2H), 7.73–7.65 (m, 2H), 7.60–7.51 (m, 1H). ***Z*-Isomer: δ (ppm)** 8.06 (dd, *J* = 16.2, 8.7 Hz, 2H), 7.55 – 7.52 (m, 2H), 7.27 (d, *J* = 8.7 Hz, 1H).

**$^{13}C\{^1H\}$ NMR (101 MHz, $(CD_3)_2CO$) *E*-Isomer: δ (ppm)** 164.7, 151.3, 147.8, 133.2, 132.2, 131.4, 130.9, 127.9, 127.4, 122.8.

**HRMS (ESI):** calc. for $C_{13}H_5O_2N_2Cl_4$ [M-H]: 360.911024, found: 360.91069.

**(*E*/*Z*)-Dimethyl 4,4'-(diazene-1,2-diyl)-bis(3-fluorobenzoate) (63)**

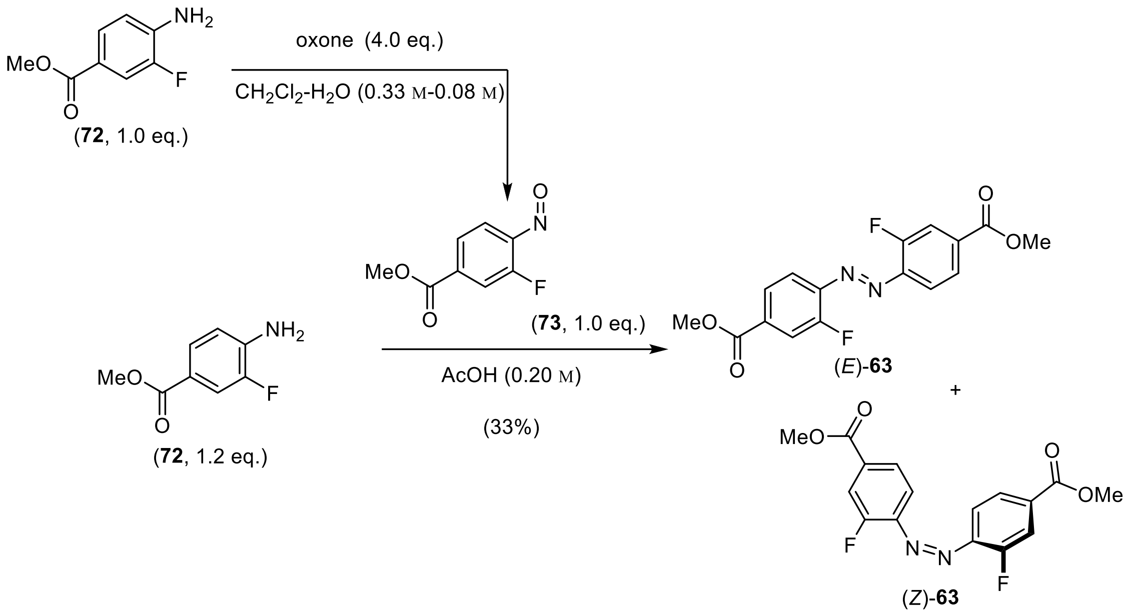


To a solution of methyl 4-amino-3-fluorobenzoate (**72,** 500 mg, 2.96 mmol, 1.0 eq.) in $CH_2Cl_2$ (37 mL) was oxone (3.64 g, 5.91 mmol, 4.0 eq.) in $H_2O$ (9 mL) added. After stirring at rt for 4 h the reaction mixture was partitioned between $CH_2Cl_2$ and $H_2O$ and the aqueous layer was extracted with $CH_2Cl_2$ (2 × 50 mL). The combined organic layers were washed with 1 M aq. HCl (2 × 100 mL), sat. aq. $NaHCO_3$ (2 × 100 mL), $H_2O$ (2 × 100 mL) and sat. aq. NaCl (2 × 100 mL), dried over $MgSO_4$, filtered and concentrated *in vacuo.* The obtained 2- methyl 3-fluoro-4-nitrosobenzoate (**73**) was directly used without further purification.

Methyl 3-fluoro-4-nitrosobenzoate (**73**, 542 mg, 2.96 mmol, 2.0 eq.) and methyl-4-amino-3-fluorobenzoate (**72**, 250 mg, 1.48 mmol, 1.0 eq.) were dissolved in AcOH (7.4 mL). After stirring at rt for 2.5 d the reaction mixture was diluted with hexane (300 mL) and washed with $H_2O$ (2 × 300 mL). The organic layer was dried over $MgSO_4$, filtered and concentrated *in vacuo*. Purification by flash column chromatography (hexane/EtOAc 75:25) afforded (*E*/*Z*)-dimethyl 4,4'-(diazene-1,2-diyl)-bis(3-fluorobenzoate) ((*E*/*Z*)-**63**, 163 mg, 0.49 mmol, 33%) as an isomeric mixture (*E*/*Z* = 97:3 ($^{19}F\{^1H\}$ NMR)) orange solid.

***R*f** (*n*-hexane/EtOAc 75:25) = 0.45. (visible)

**$^1$H NMR (500 MHz, CDCl3) *E*-Isomer: δ (ppm)** 7.96 (dd, *J* = 10.8, 1.7 Hz, 1H), 7.91 (dd, *J* = 8.4, 1.7 Hz, 1H), 7.83 (dd, *J* = 8.4, 7.1 Hz, 1H), 3.97 (s, 6H).

**$^{13}C\{^1H\}$ NMR (126 MHz, CDCl3) *E*-Isomer: δ (ppm)** 165.5 (d, *J* = 3.2 Hz), 161.2, 159.1, 143.4 (d, *J* = 6.7 Hz), 134.8 (d, *J* = 8.2 Hz), 125.8 (d, *J* = 3.7 Hz), 118.9, 118.7, 118.1, 52.8.

**$^{19}F\{^1H\}$ NMR (471 MHz, CDCl3) *E*-Isomer: δ (ppm)** -122.4 (d, *J* = 7.2 Hz), -122.41 (d, *J* = 7.2 Hz). ***Z*-Isomer:**-114.5 (dd, *J* = 10.8, 7.1 Hz), -118.7 (dd, *J* = 10.8, 6.6 Hz).

**HRMS (EI):** calc. for $C_{16}H_{12}O_4N_2F_2$ [M]: 334.0765, found: 334.0758.

## ($E$)-3,5-Dichloro-4-((2,6-dichloro-4-nitrophenyl)diazenyl)benzenesulfonamide (64)

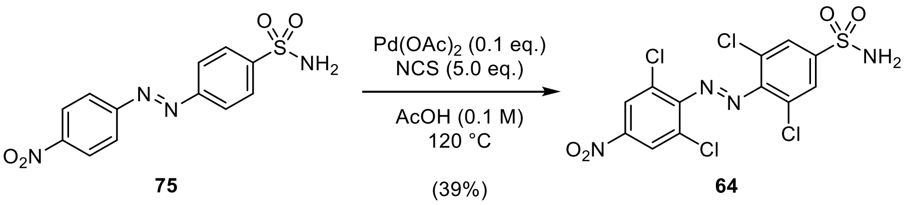

($E$)-4-((4-Nitrophenyl)diazenyl)benzenesulfonamide[12] (**75**, 1.00 g, 3.26 mmol, 1.0 eq.) $Pd(OAc)_2$ (73.3 mg, 0.326 mmol, 0.1 eq) and NCS (2.18 g, 16.3 mmol, 5.0 eq) were dissolved in AcOH (32.6 mL) and heated to 120 °C for 13 h. It was observed during heating process, that the orange suspension turned into a dark red solution. After cooling to rt, the solvent was removed *in vacuo* and the brown residue was dissolved in EtOAc (150 mL). The organic layer was washed with sat. aq. $NaHCO_3$ (50 mL), sat. aq. NaCl (50 mL) and dried over $Na_2SO_4$. The residue was purified via flash column chromatography (dry loaded, pentane/acetone 17:3 → 4:1) to give ($E$)-3,5-dichloro-4-((2,6-dichloro-4-nitrophenyl)diazenyl)benzenesulfonamide (**64**, 560 mg, 1,26 mmol, 39%) as an orange-brown solid.

$\boldsymbol{R_f}$ (pentane/acetone 7:3) = 0.56. (visible)

$\mathbf{t_R}$ (reverse-phase semi-preparative HPLC, MeCN:$H_2O$:HCOOH = 60:40:1% → 100:0:1% over 40 min) = 14.08 min.

**$^1$H NMR (400 MHz, $(CD_3)_2CO$) δ (ppm)** = 8.53 (s, 2H), 8.10 (s, 2H), 7.07 (s, 2H).

**$^{13}C\{^1H\}$ NMR (101 MHz, $(CD_3)_2CO$) δ (ppm)** = 152.2, 149.7, 148.6, 147.2, 128.3, 128.0, 125.9.

**HRMS (EI):** calc. for $C_{12}H_5Cl_4N_4O_4S$ $[M]^+$: 440.8791, found: 440.8784.

**IR (Diamond-ATR, neat) $\upsilon_{max}$ ($cm^{-1}$)** = 3340 (m), 3263 (m), 3092 (w), 2922 (w), 1532 (s), 1387 (m), 1371 (w), 1349 (vs), 1331 (vs), 1281 (w), 1168 (vs), 1147 (m), 1131 (m), 916 (s), 896 (m), 882 (m), 870 (s), 817 (vs), 778 (s), 740 (s), 709 (s), 667 (s).

## Methyl 4-amino-3-chloro-5-fluorobenzoate (65)

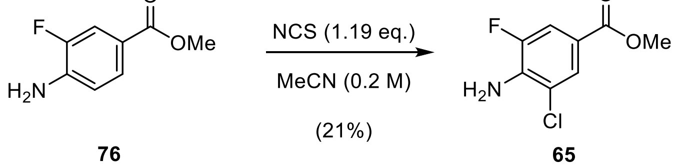

Methyl 4-amino-3-fluorobenzoate (**76**, 400 mg, 2.36 mmol, 1.00 eq) and NCS (375 mg, 2.81 mmol, 1.19 eq) are dissolved in MeCN (11.8 mL) and stirred at 85 °C for 4.5 h. The reaction mixture was concentrated *in vacuo,* redissolved in $CH_2Cl_2$ (50 mL) and washed with 0.5 M aq. HCl (2 × 50 mL). The aqueous phase was alkalized with 0.5 M aq. NaOH (120 mL, pH10) and extracted with $CH_2Cl_2$ (3 × 200 mL). The combined organic phases

were dried over $Na_2SO_4$, filtered and concentrated *in vacuo.* Methyl 4-amino-3-chloro-5-fluorobenzoate (**65**, 103 mg, 0.50 mmol, 21%) was used without further purification.

***R*f** (*n*-hexane/EtOAc 8:2) = 0.75. (254 nm)

**$^{1}$H NMR (400 MHz, $(CD_3)_2CO$) δ =** 7.71 (t, *J* = 1.7 Hz, 1H), 7.54 (dd, *J* = 11.4, 1.7 Hz, 1H), 3.83 (s, 3H).

**$^{13}$C{$^{1}$H} NMR (101 MHz, $(CD_3)_2CO$) δ** = 178.9, 165.7 (d, *J* = 3.2 Hz), 152.0, 149.6, 139.3, 127.4 (d, *J* = 2.4 Hz), 115.3 (d, *J* = 20.3 Hz), 52.3.

**$^{19}$F{$^{1}$H} NMR (376 MHz, $(CD_3)_2CO$) δ (ppm) =** -133.7.

NMR Data – Structural Analysis

## Final compounds

### (*E*/*Z*)-1,2-Bis(2,6-difluorophenyl)diazene (2)

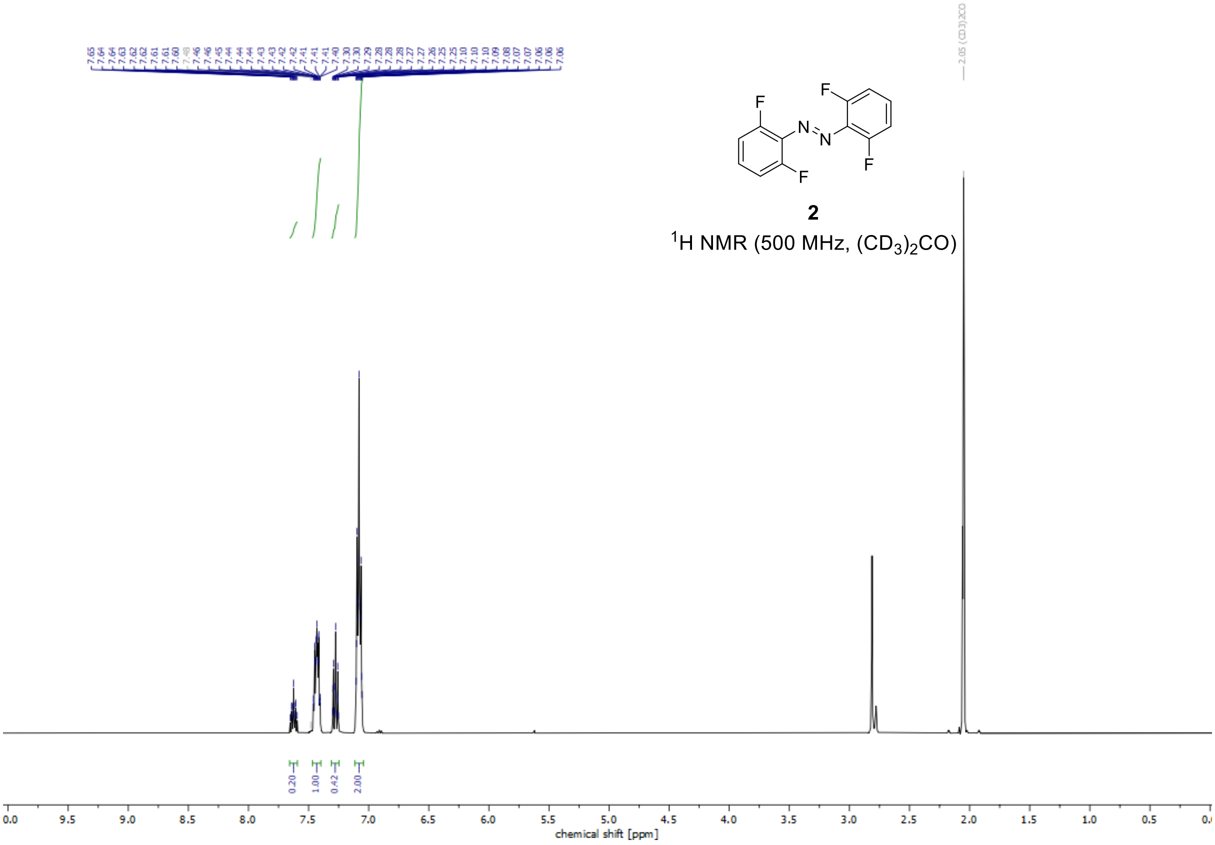

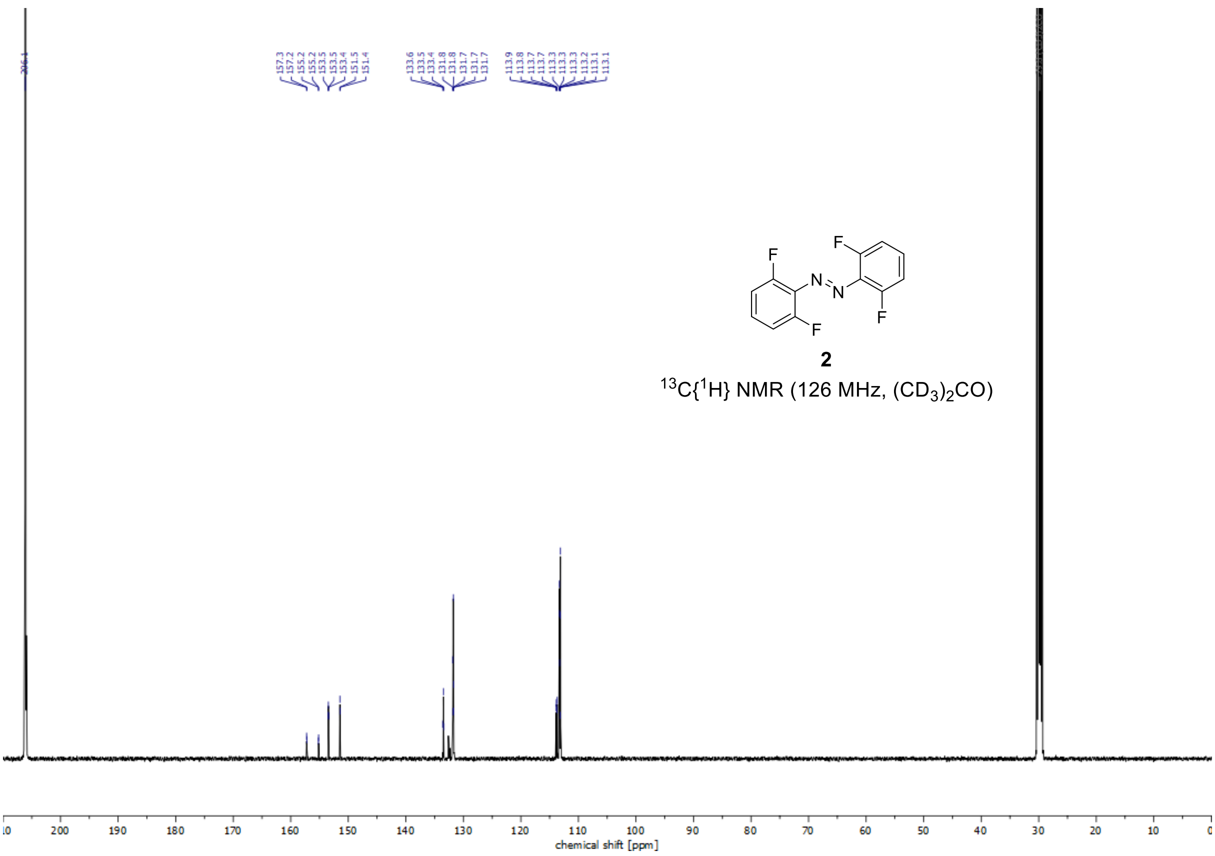

F
N
N
F
F
F
2
$^{13}C\{^{1}H\}$ NMR (126 MHz, $(CD_3)_2CO$)
chemical shift [ppm]


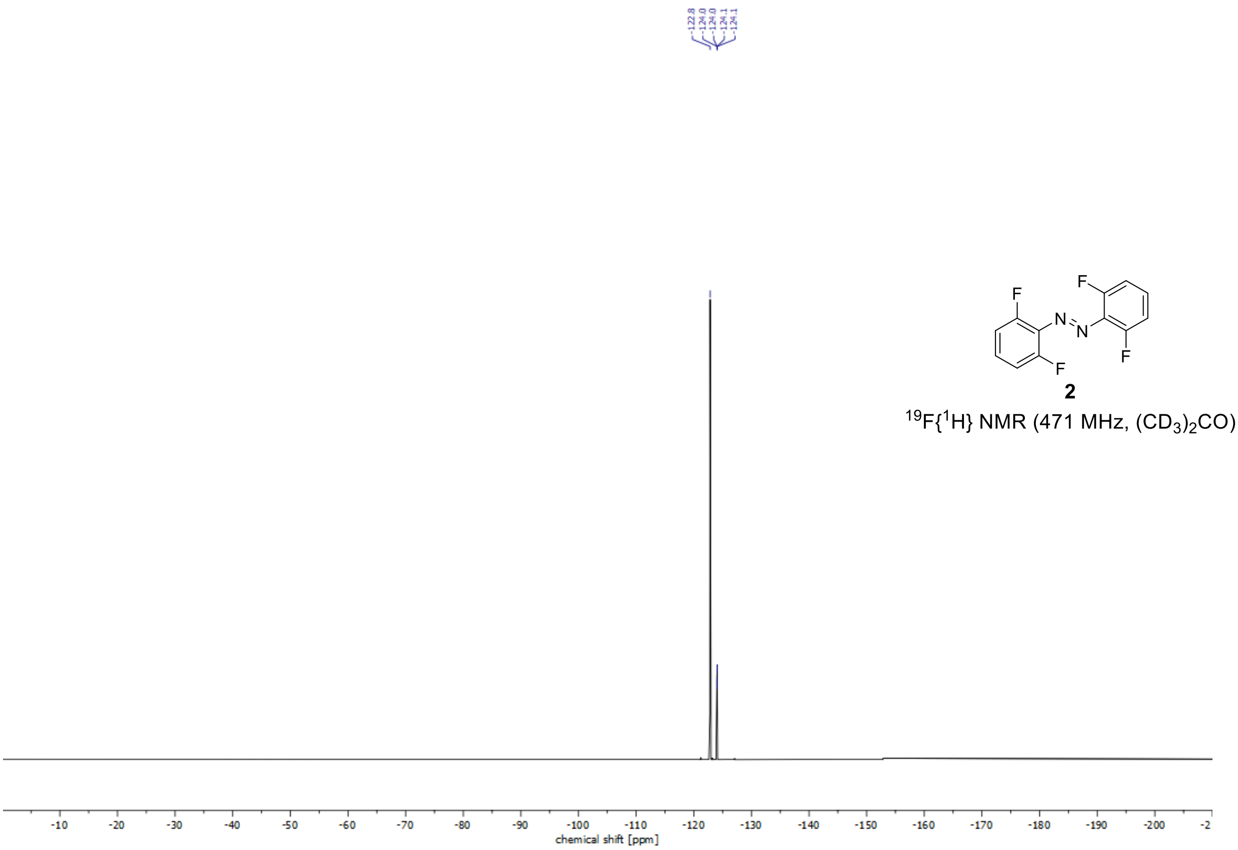

F
N
N
F
F
F
2
$^{19}F\{^{1}H\}$ NMR (471 MHz, $(CD_3)_2CO$)
chemical shift [ppm]

**(*E*/*Z*)-1,2-Bis(2-chloro-6-fluorophenyl)diazene (3)**

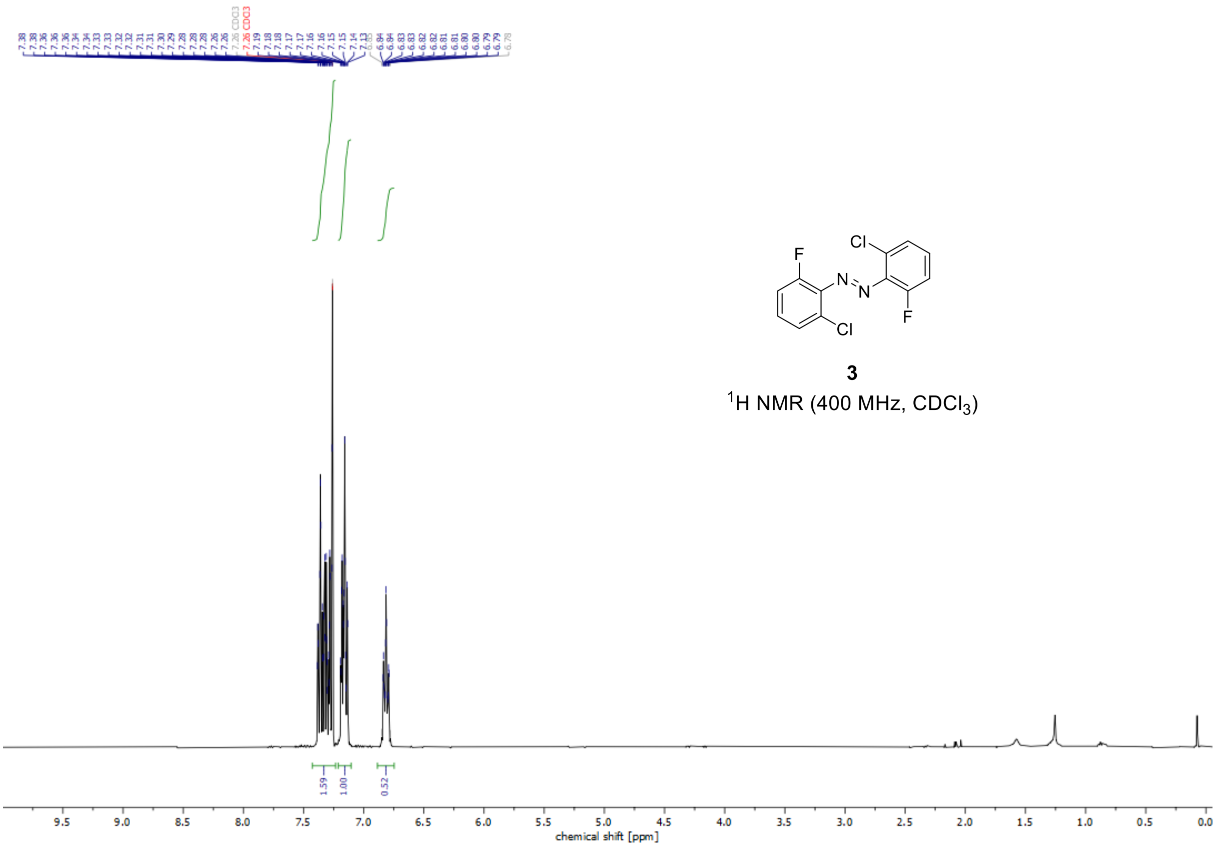


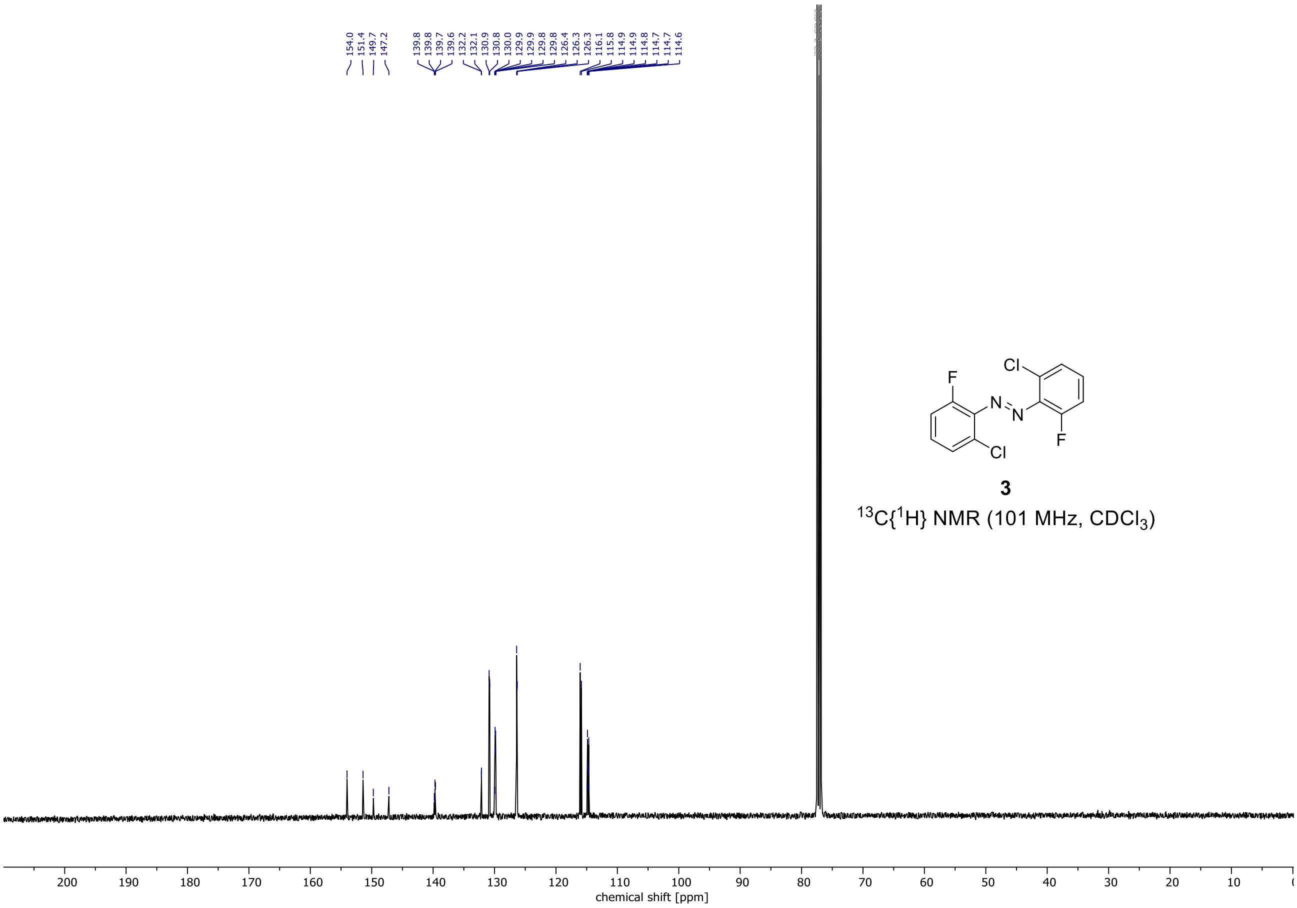

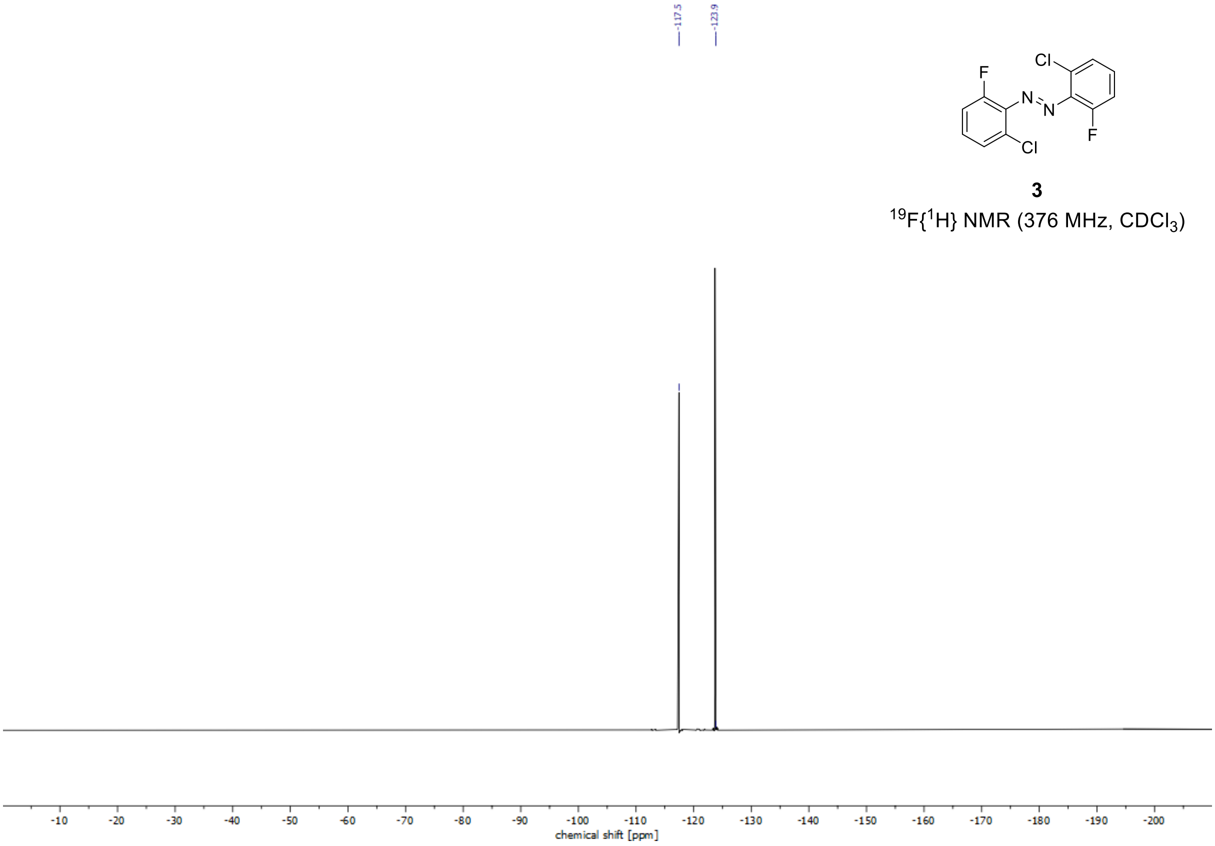


**3**

$^{19}F\{^{1}H\}$ NMR (376 MHz, $CDCl_3$)

## (*E*/*Z*)-1,2-Bis(2-bromo-6-fluorophenyl)diazene (4)

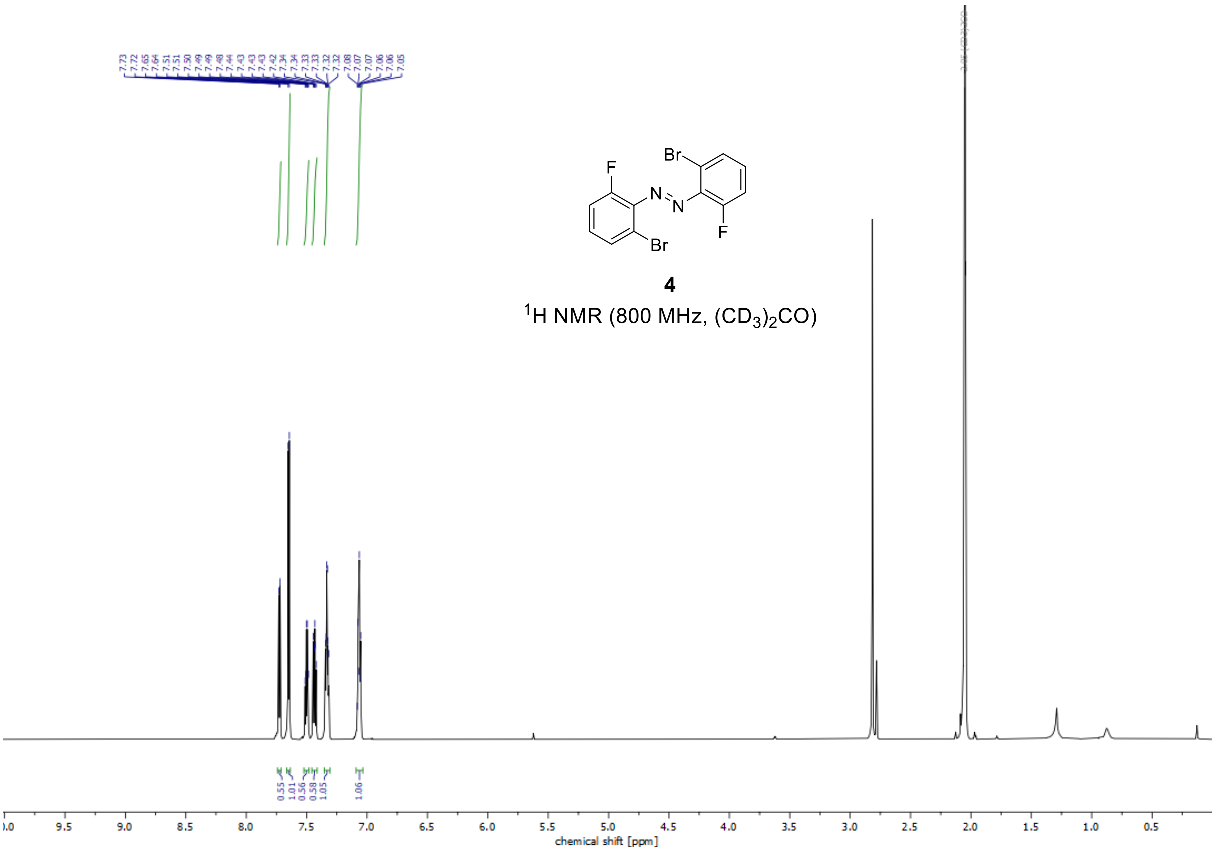


**4**

$^{1}H$ NMR (800 MHz, $(CD_3)_2CO$)

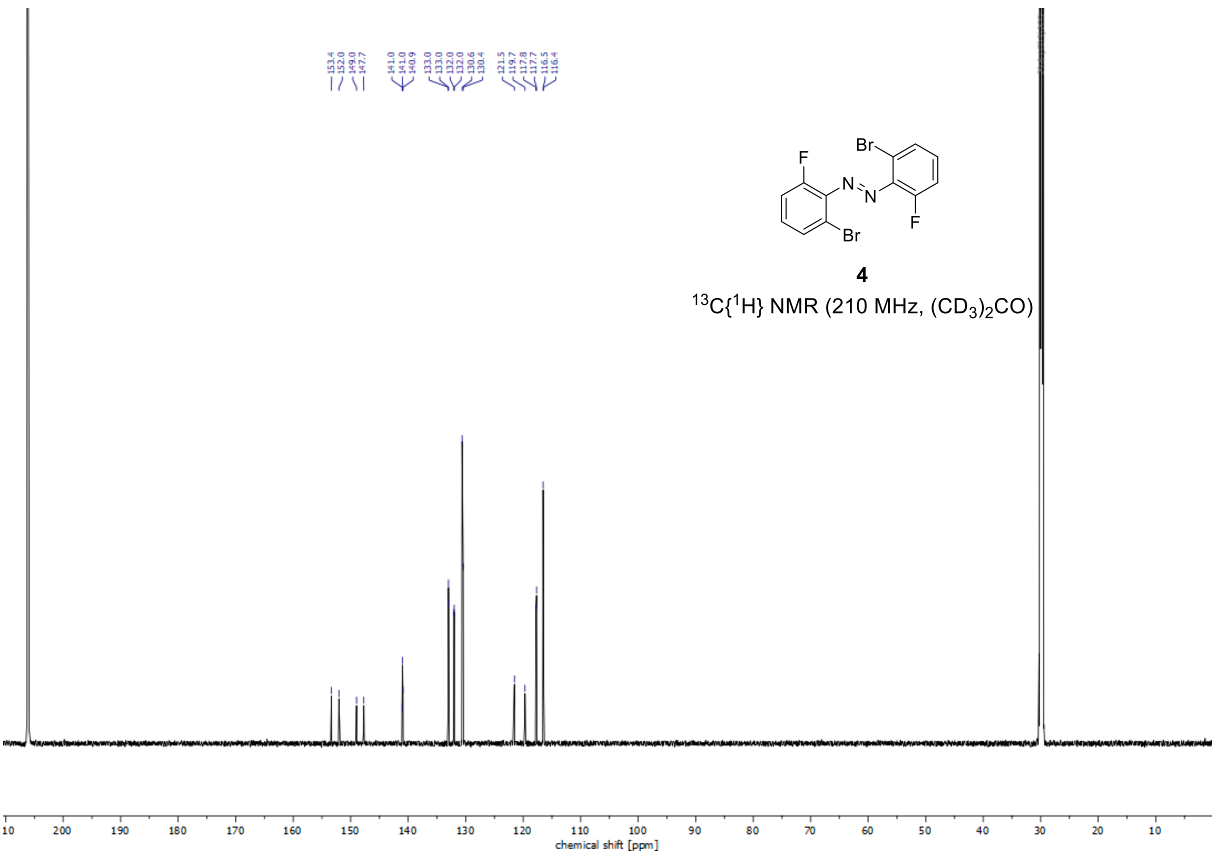


**4**

$^{13}C\{^{1}H\}$ NMR (210 MHz, $(CD_3)_2CO$)

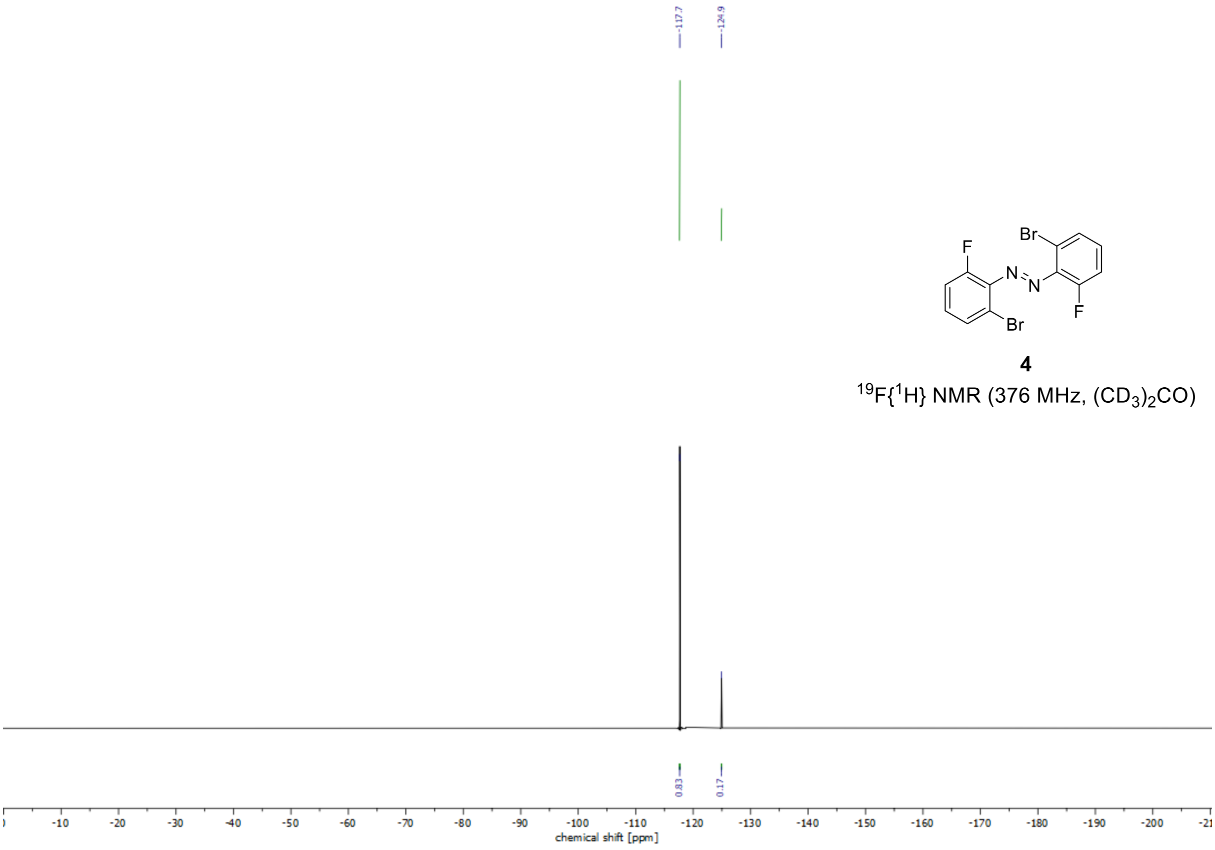


**4**

$^{19}F\{^{1}H\}$ NMR (376 MHz, $(CD_3)_2CO$)

**(*E*/*Z*)-1,2-Bis(2,6-dichlorophenyl)diazene (6)**

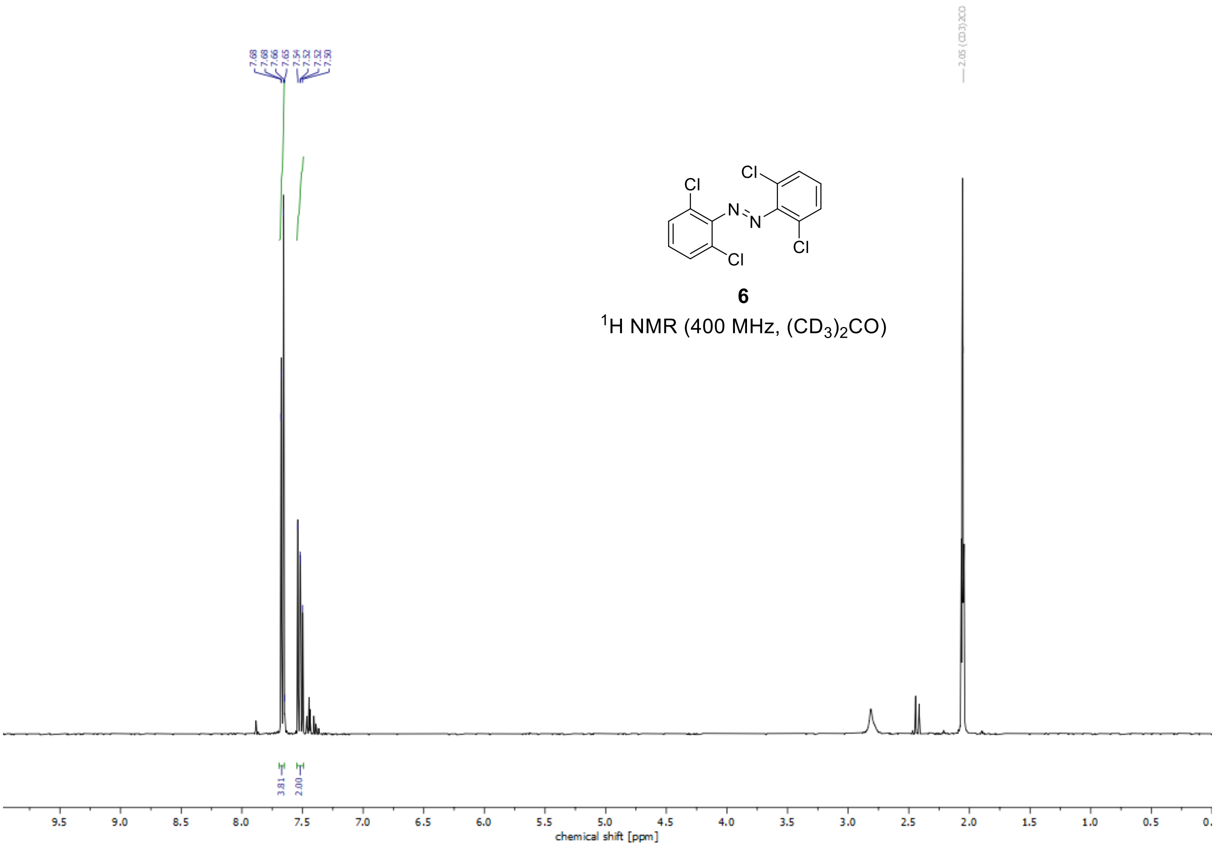


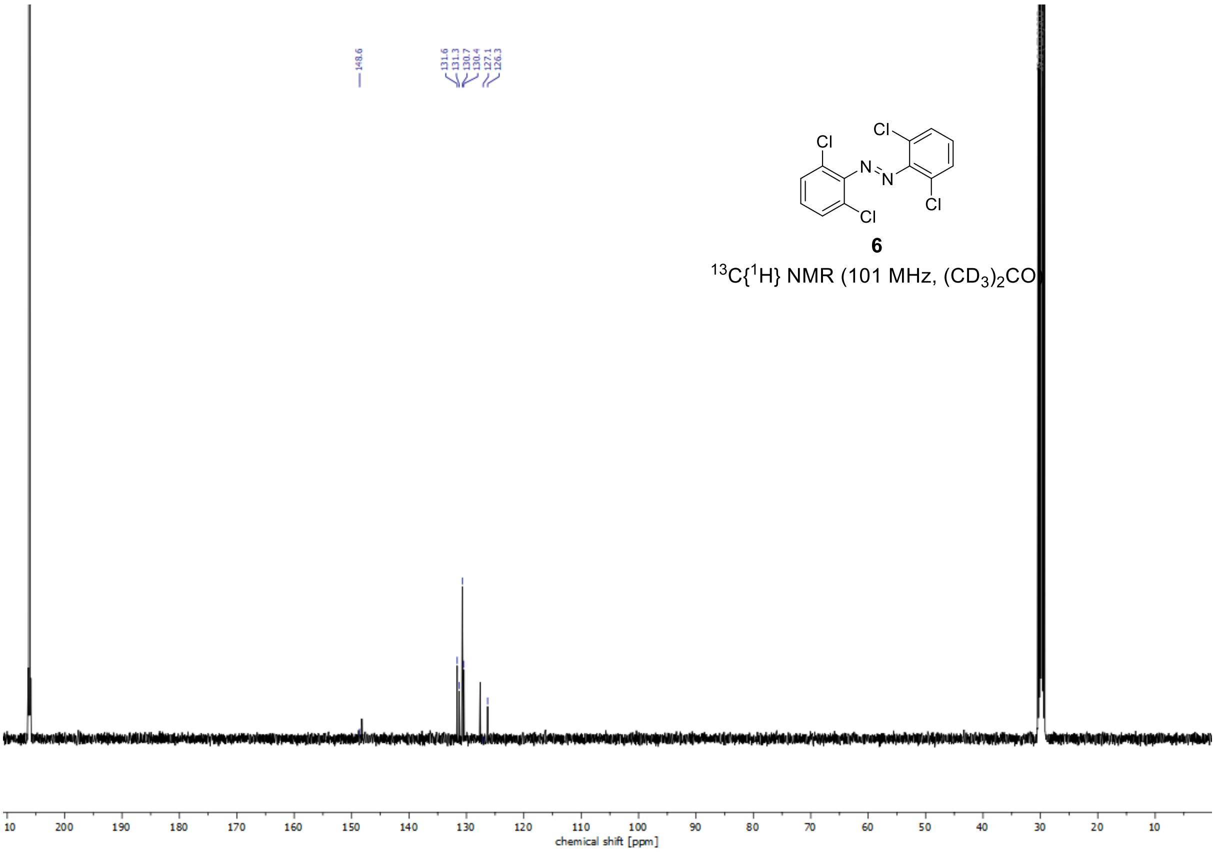

## (*E*/*Z*)-1,2-Bis(2,6-dimethoxyphenyl)diazene (9)

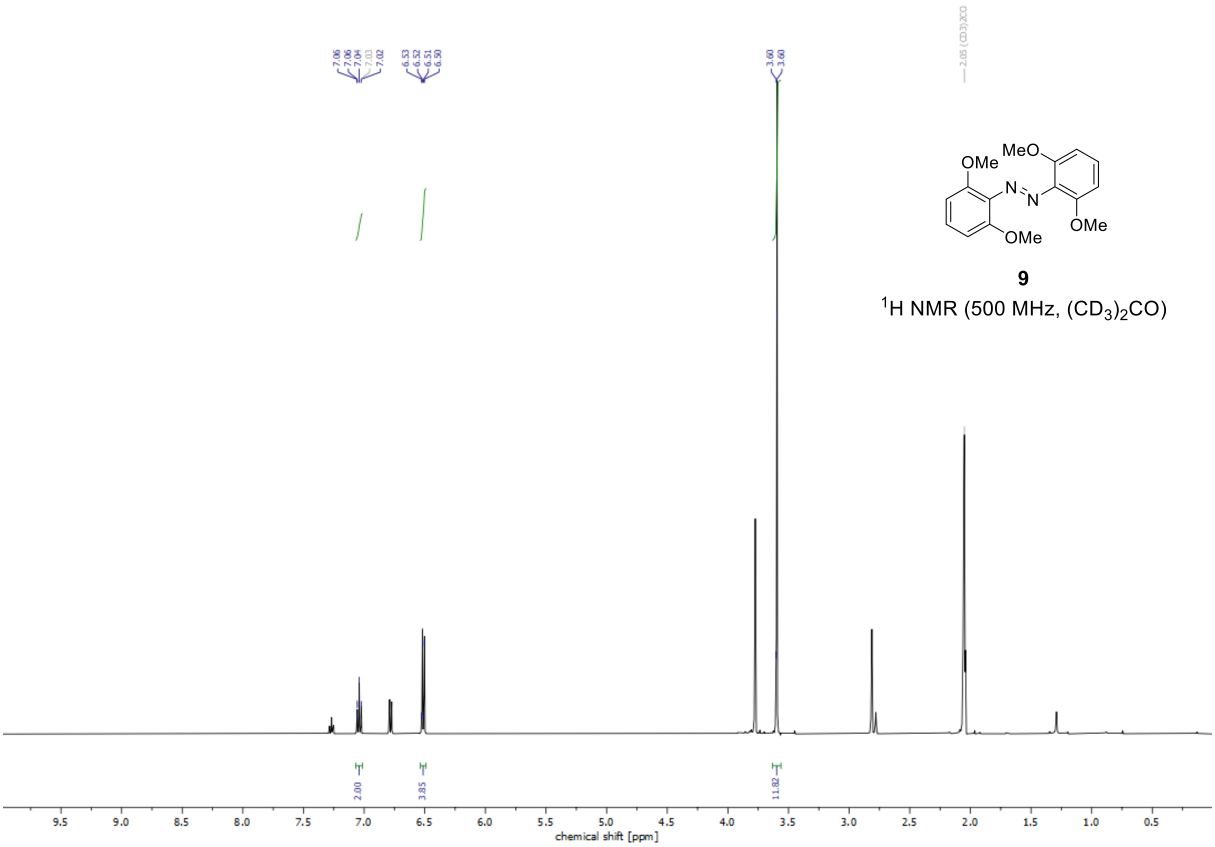


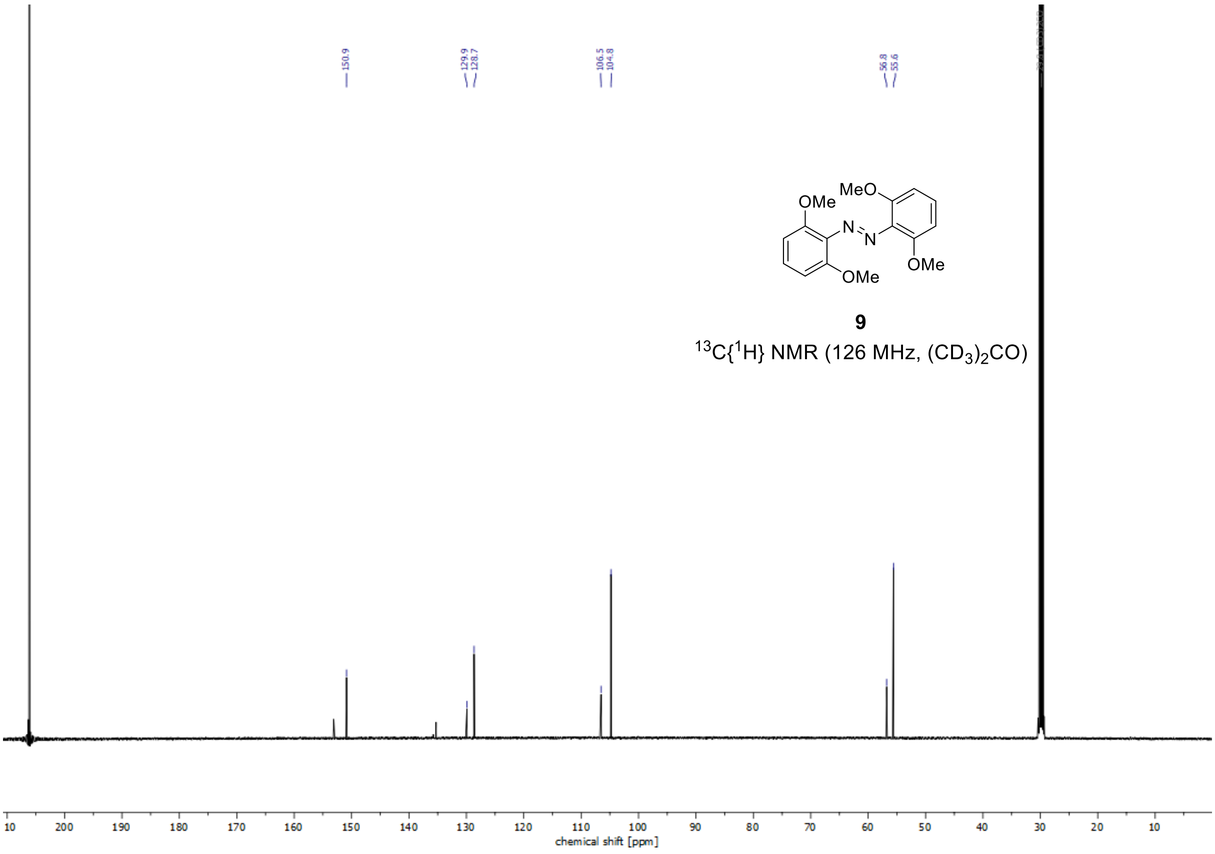

**(*E*/*Z*)-1,2-Bis(3-chloro-1-fluoronaphthalen-2-yl)diazene (12)**

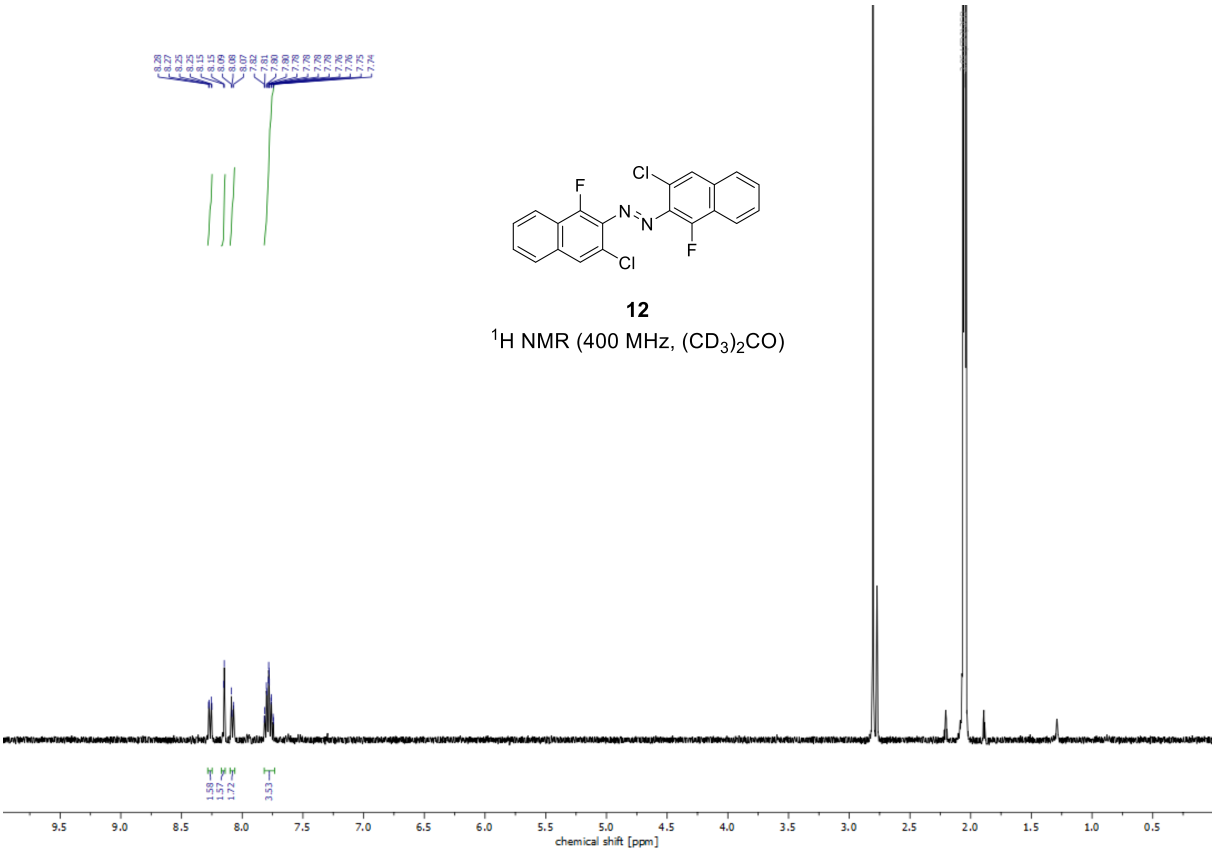


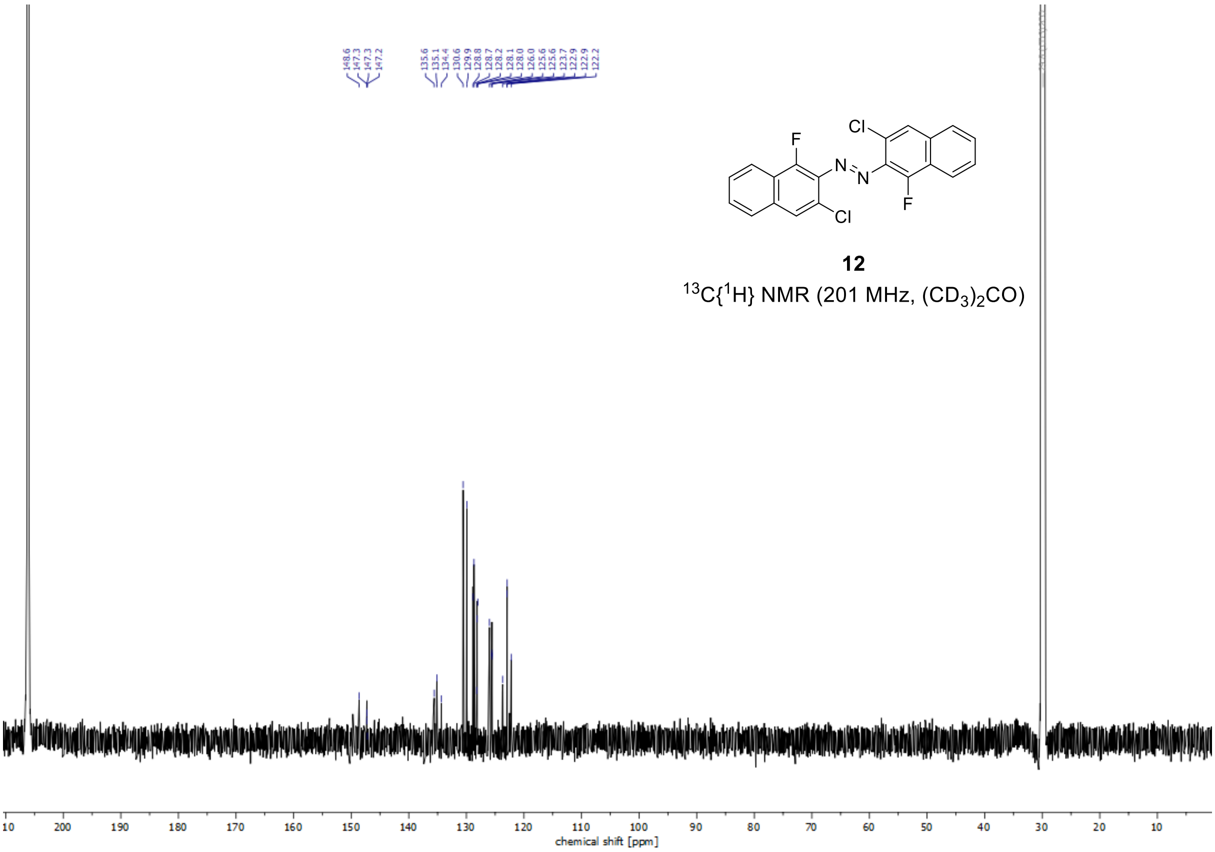

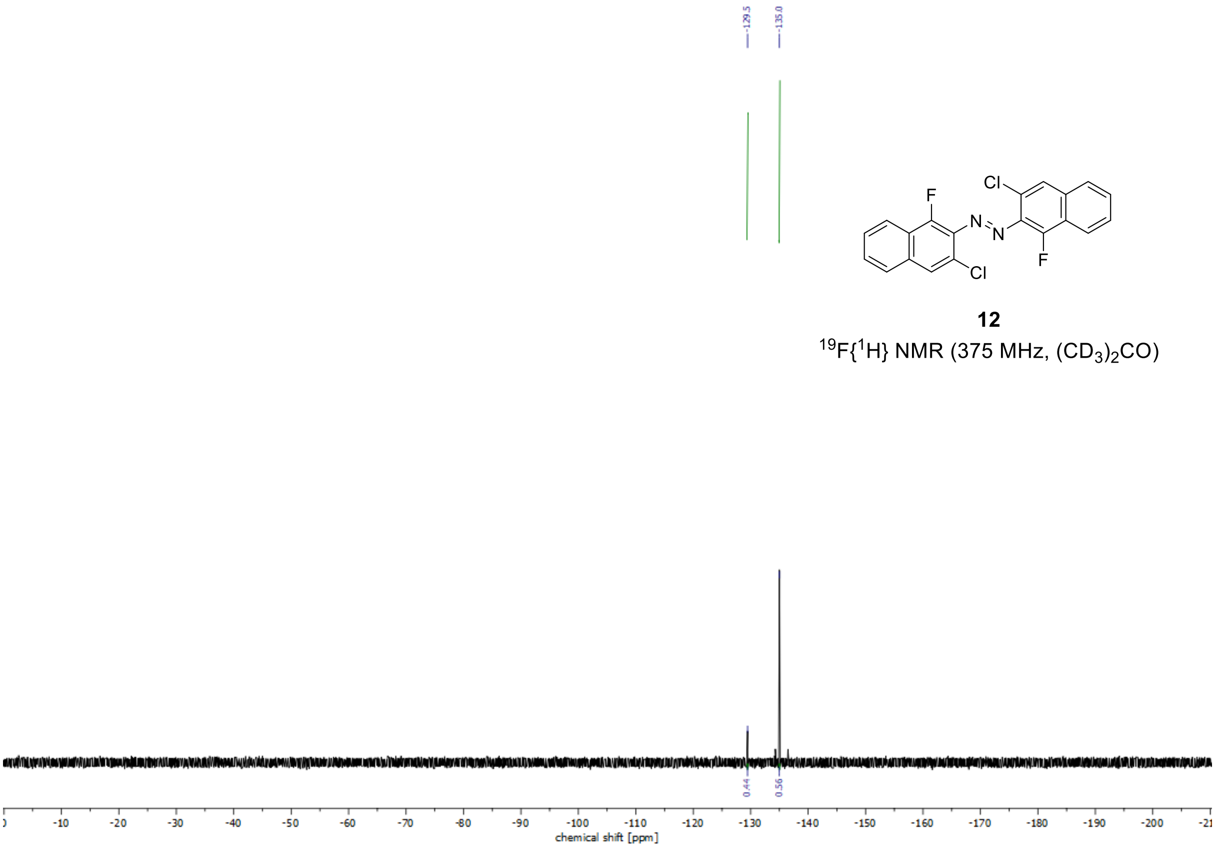


**(*E*/*Z*)-3-Chloro-4-((2-chloro-6-fluorophenyl)diazenyl)-5-fluorobenzoic acid (17)**

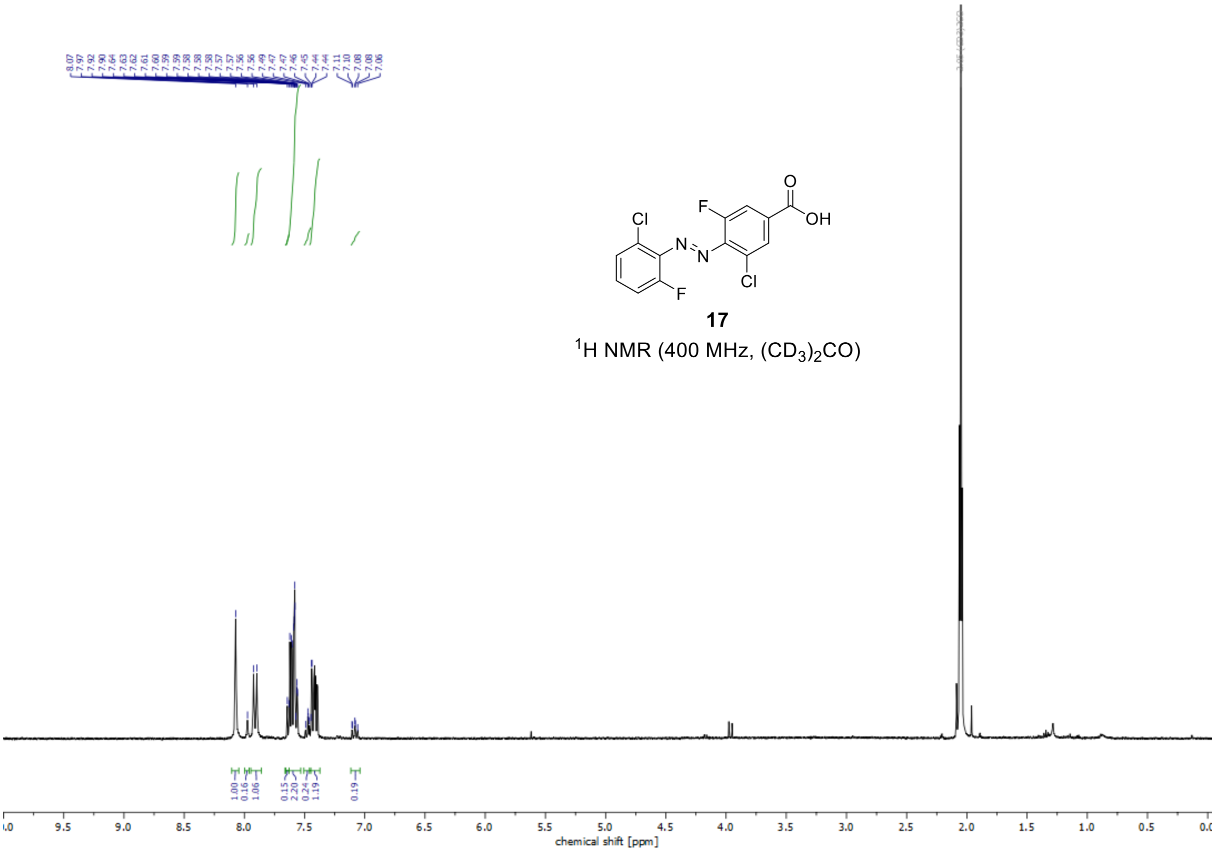

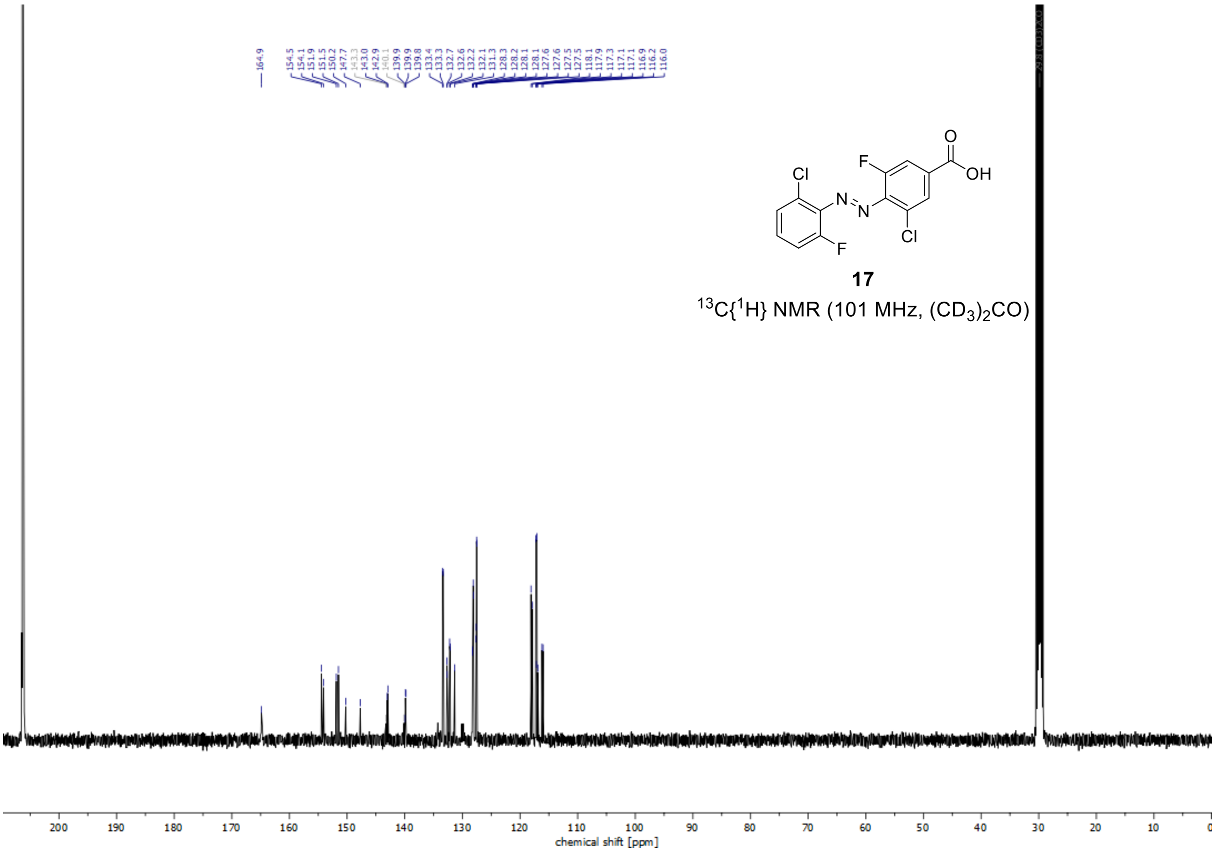


**17**

$^{13}C\{^1H\}$ NMR (101 MHz, $(CD_3)_2CO$)

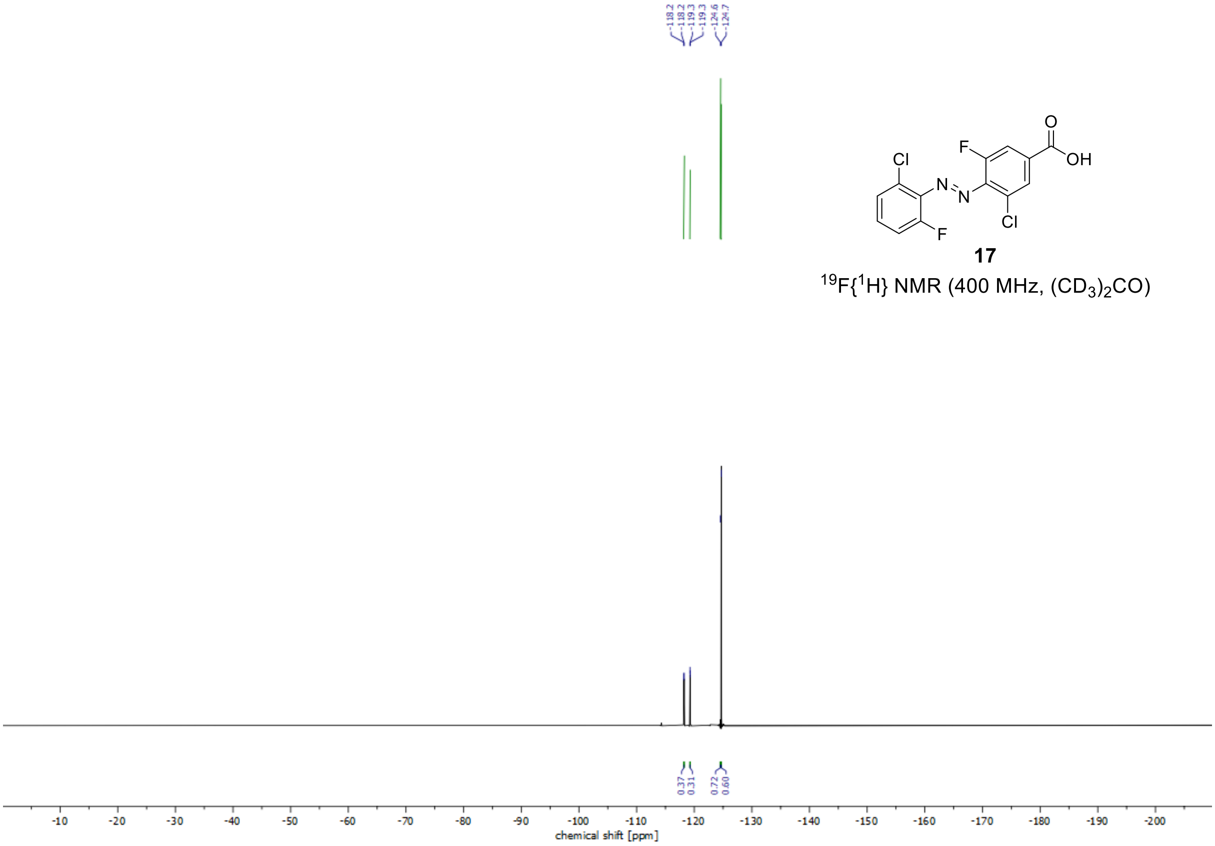


**17**

$^{19}F\{^1H\}$ NMR (400 MHz, $(CD_3)_2CO$)

**(*E*)-1-(2-Chloro-6-fluoro-4-nitrophenyl)-2-(2-chloro-6-fluorophenyl)diazene (19)**

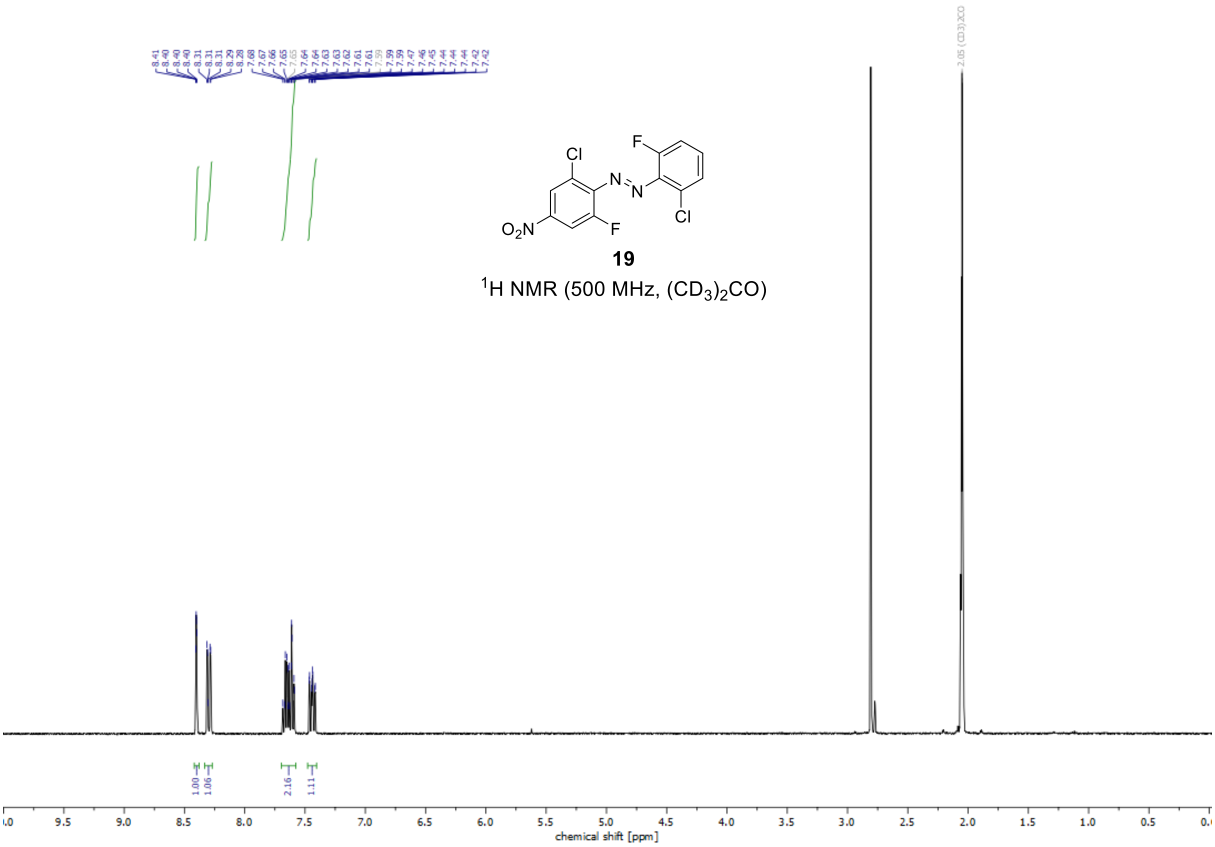


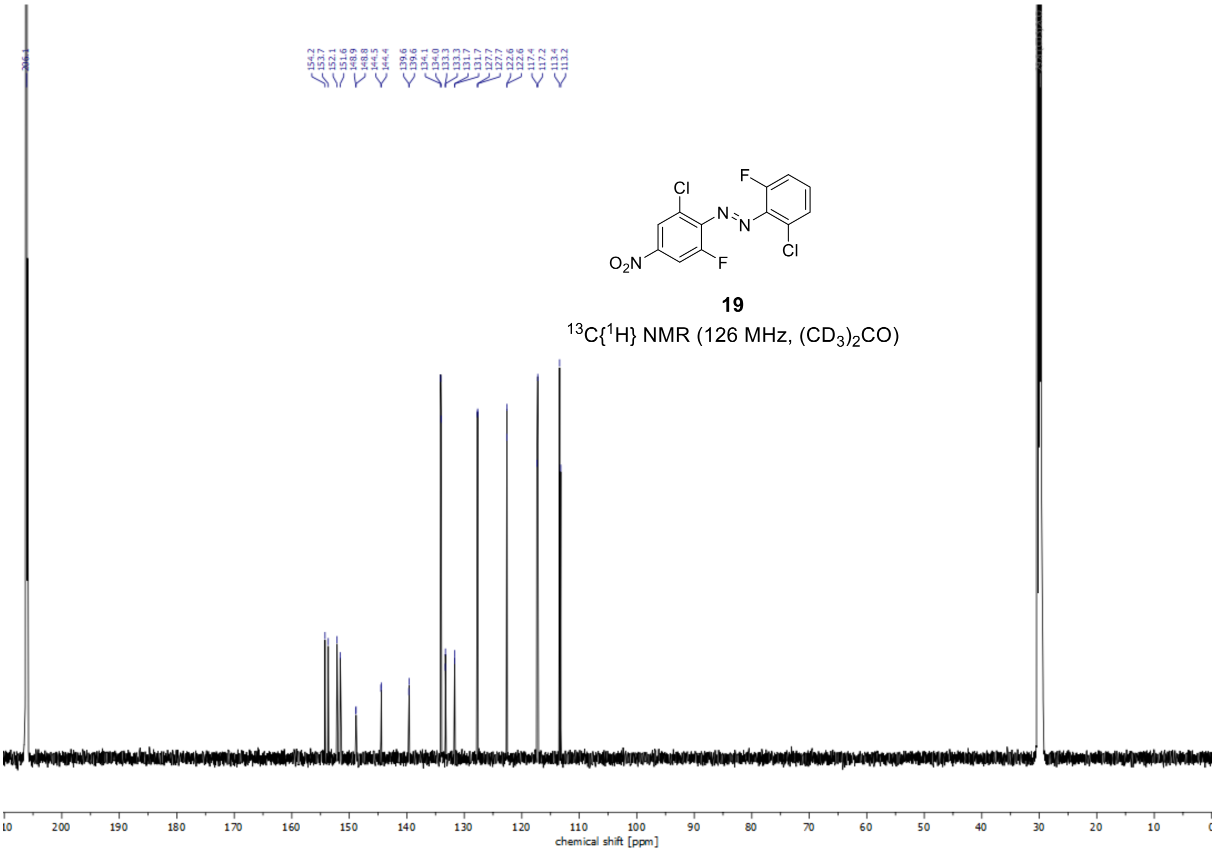

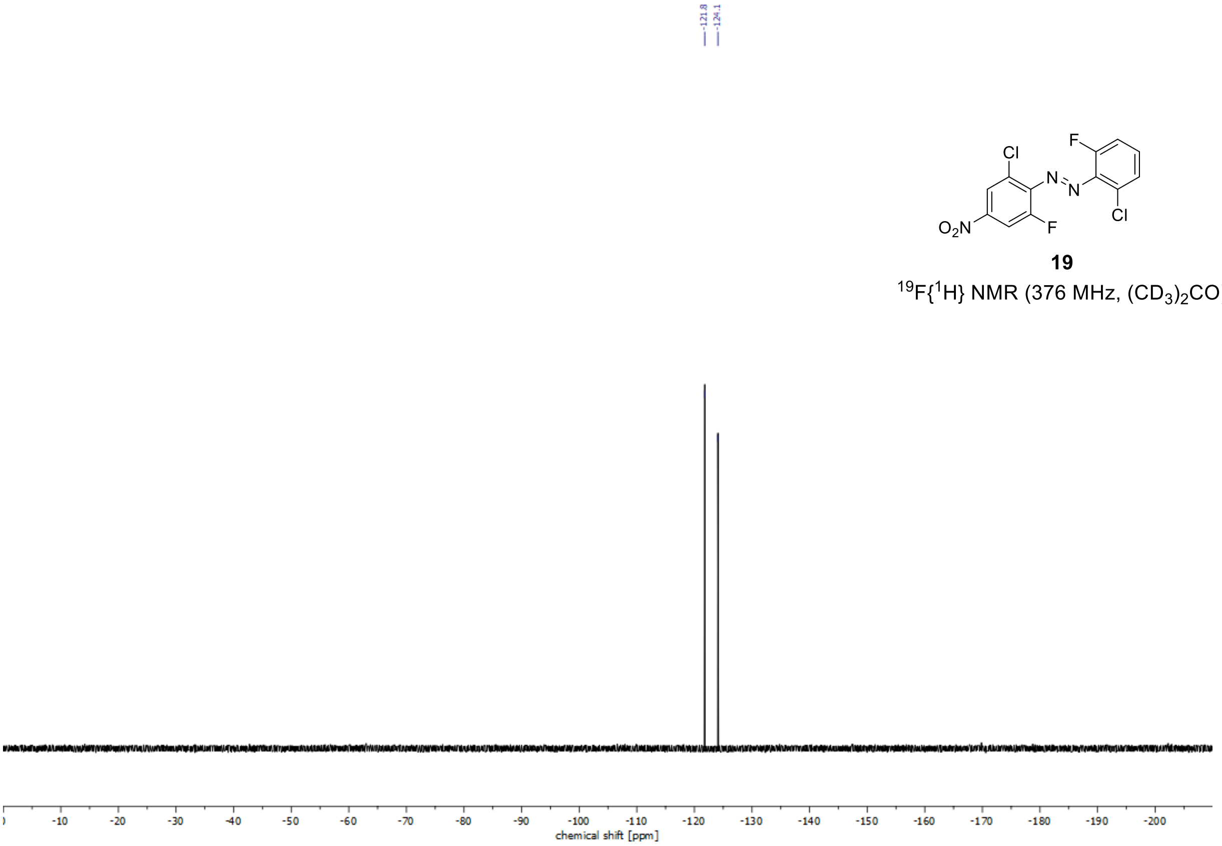


**(*E*)-3-Chloro-4-((2-chloro-6-fluoro-4-nitrophenyl)diazenyl)-5-fluorobenzoic acid (20)**

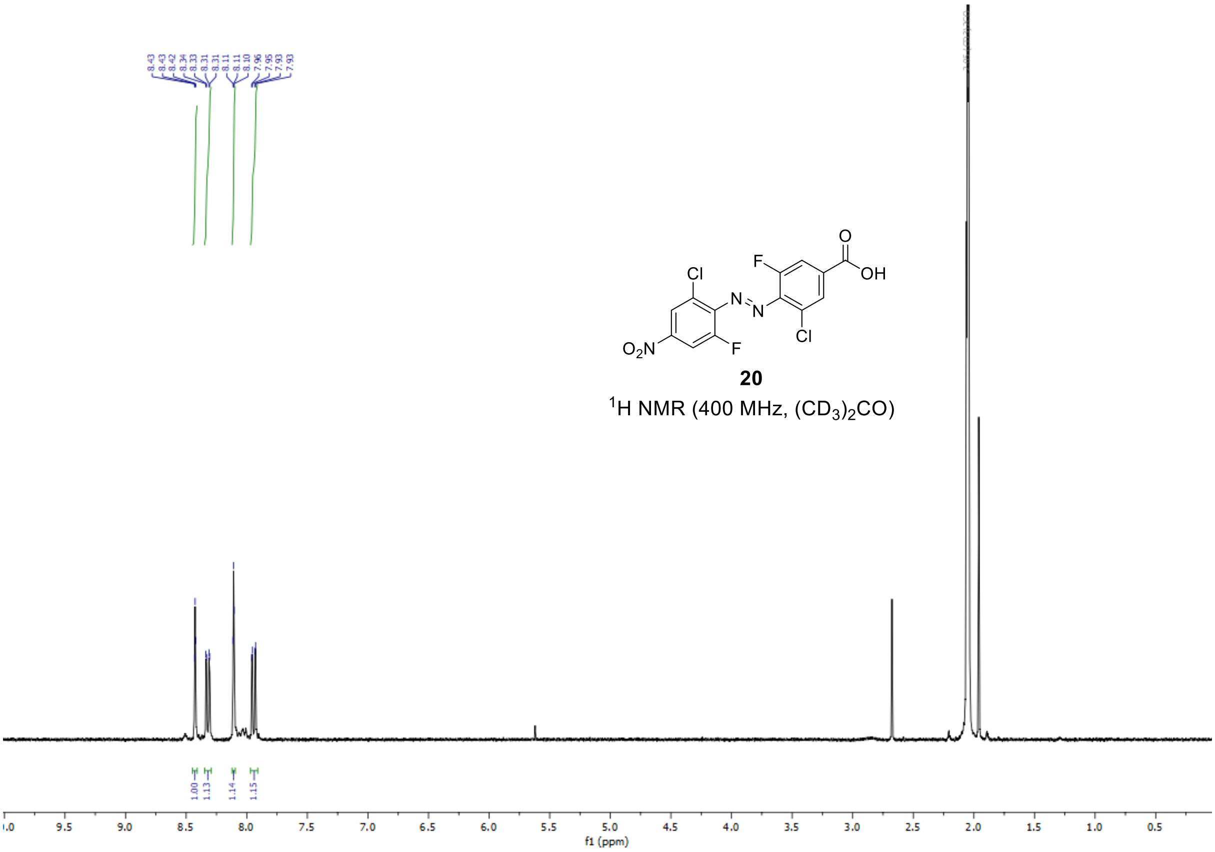

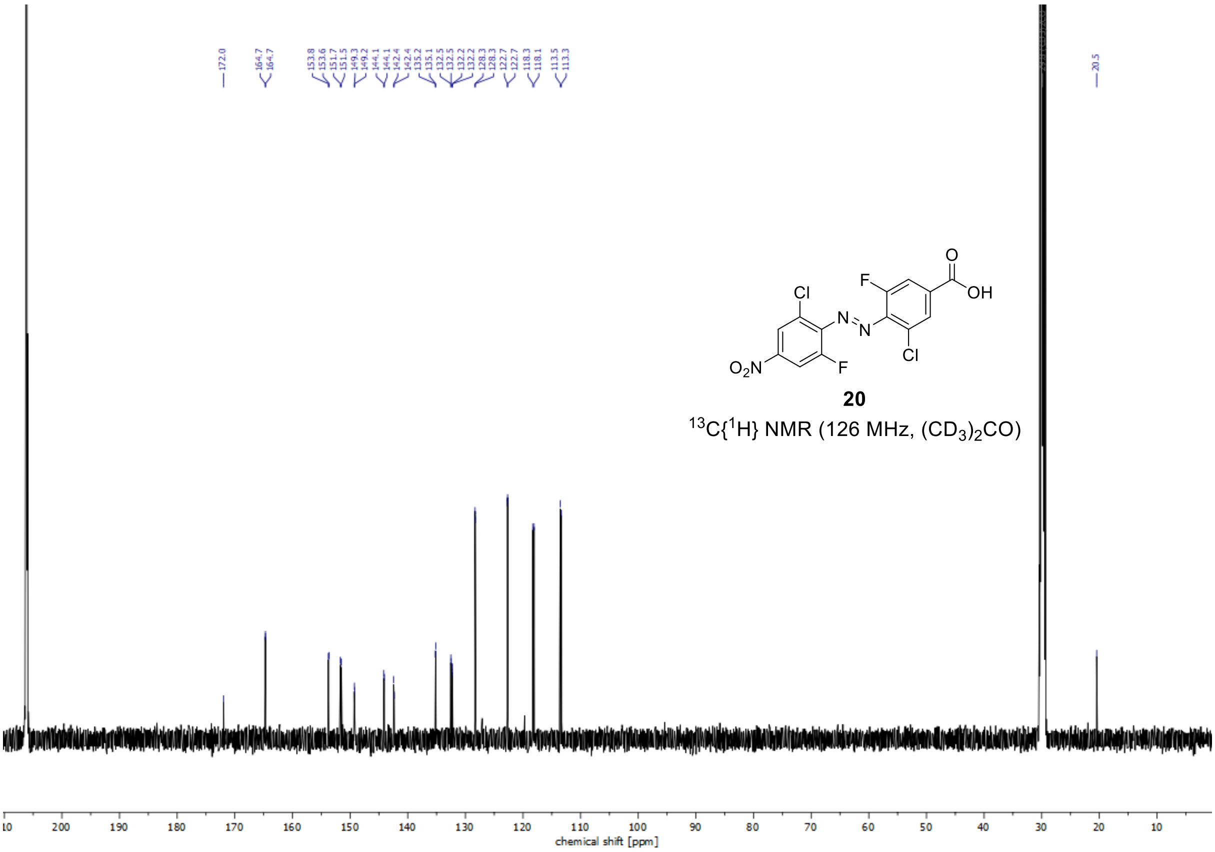
172.0
164.7 164.7
153.8 153.6 151.7 151.5 149.3 149.2 144.1 144.1 142.4 142.4 135.2 135.1 132.5 132.5 132.2 132.2 128.3 128.3 122.7 122.7 118.3 118.1 113.5 113.3
20.5
O
F
OH
Cl
N
N
$O_2N$
F
Cl
**20**
$^{13}C\{^{1}H\}$ NMR (126 MHz, $(CD_3)_2CO$)
chemical shift [ppm]

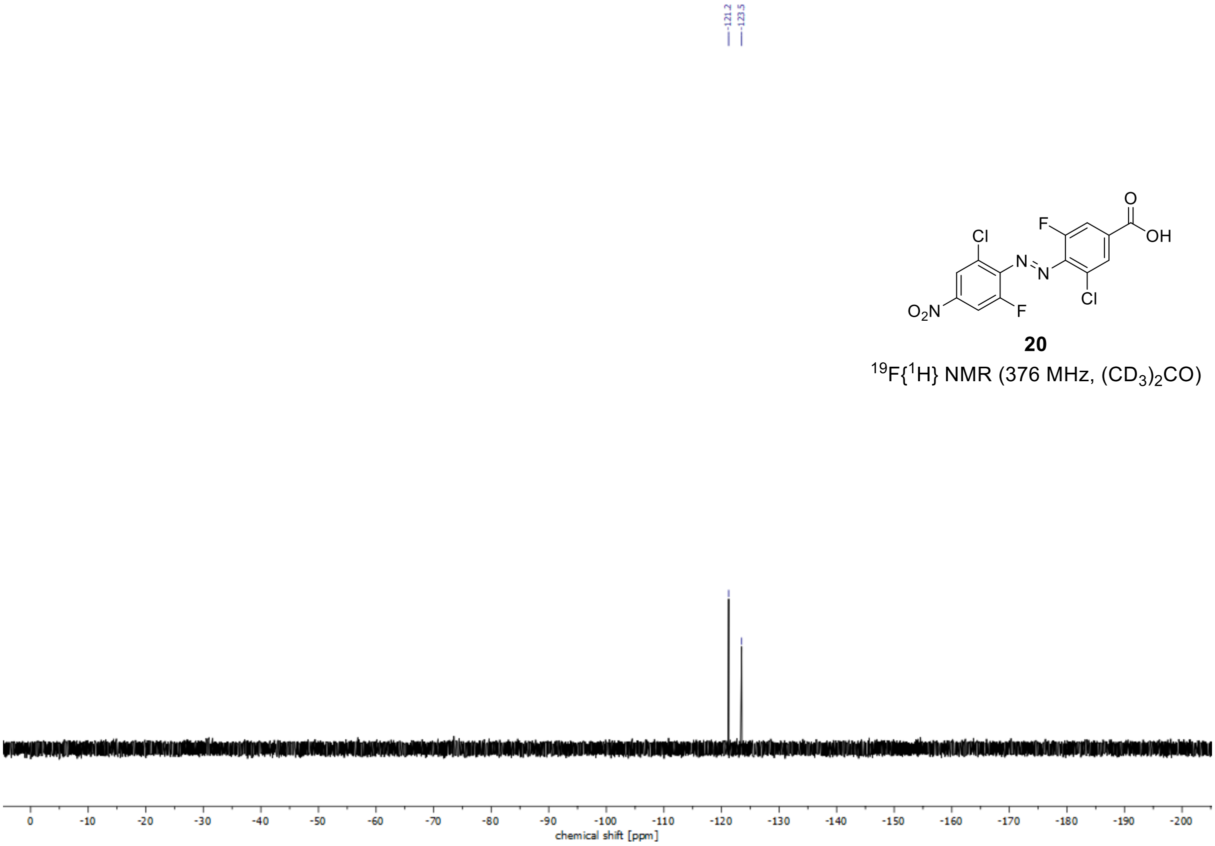
-121.2
-123.5
O
F
OH
Cl
N
N
$O_2N$
F
Cl
**20**
$^{19}F\{^{1}H\}$ NMR (376 MHz, $(CD_3)_2CO$)
chemical shift [ppm]

**(*E*/*Z*)-Methyl-3-chloro-4-((2-chloro-6-fluorophenyl)diazenyl)-5-fluorobenzoate (21)**

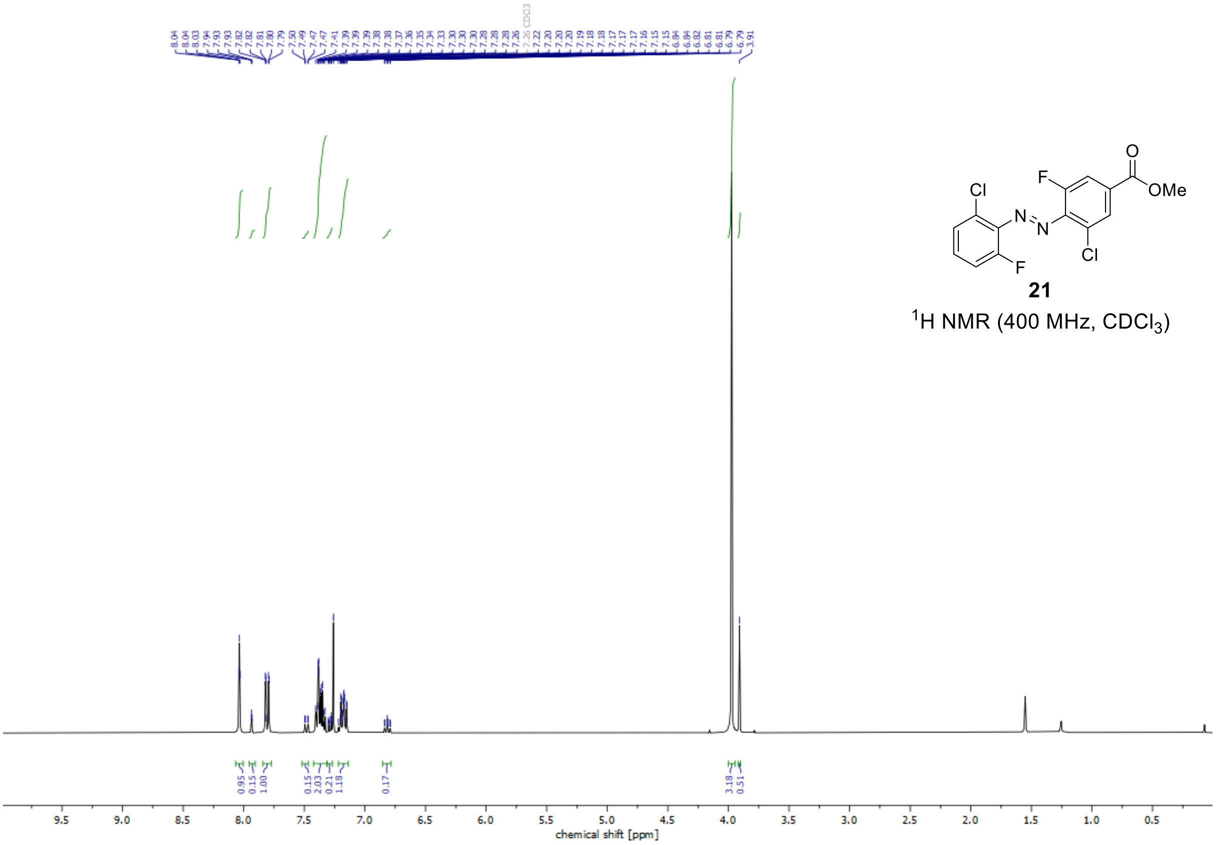


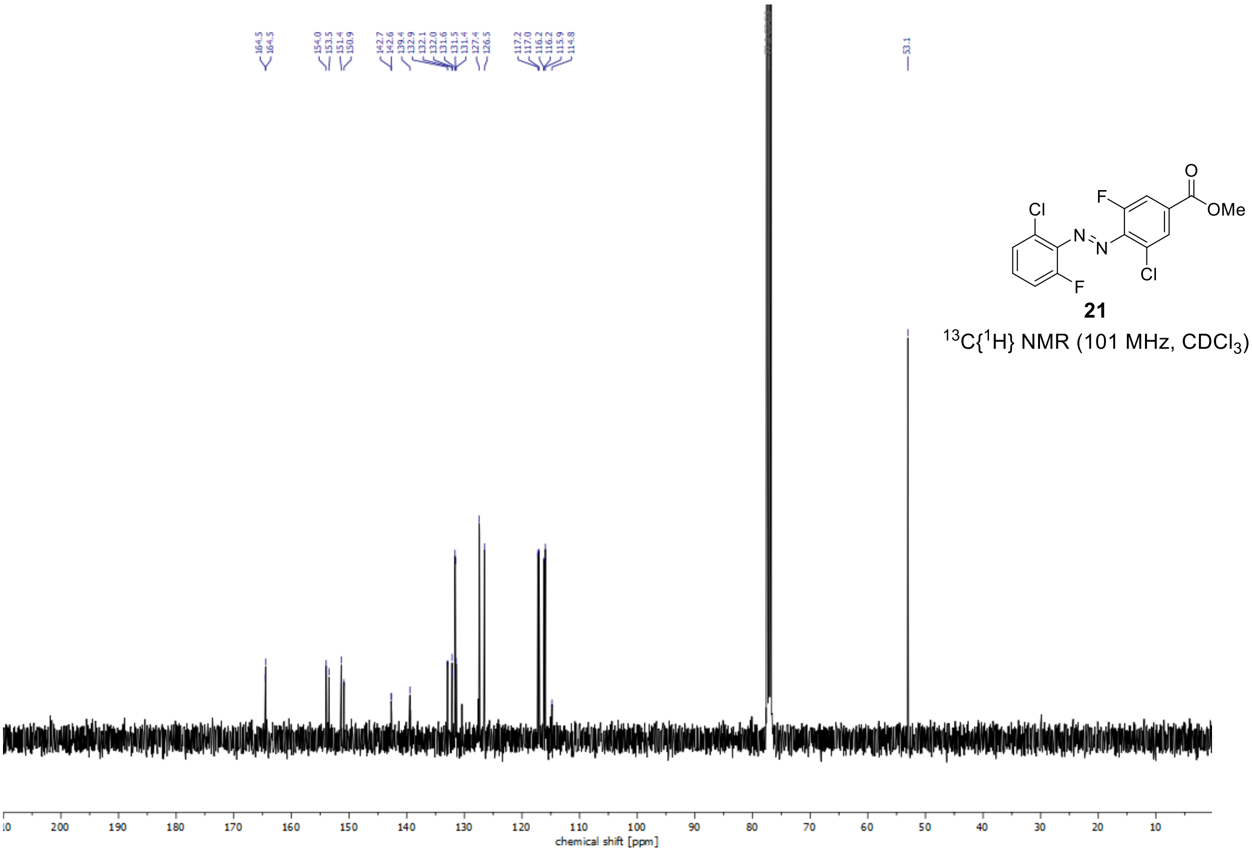

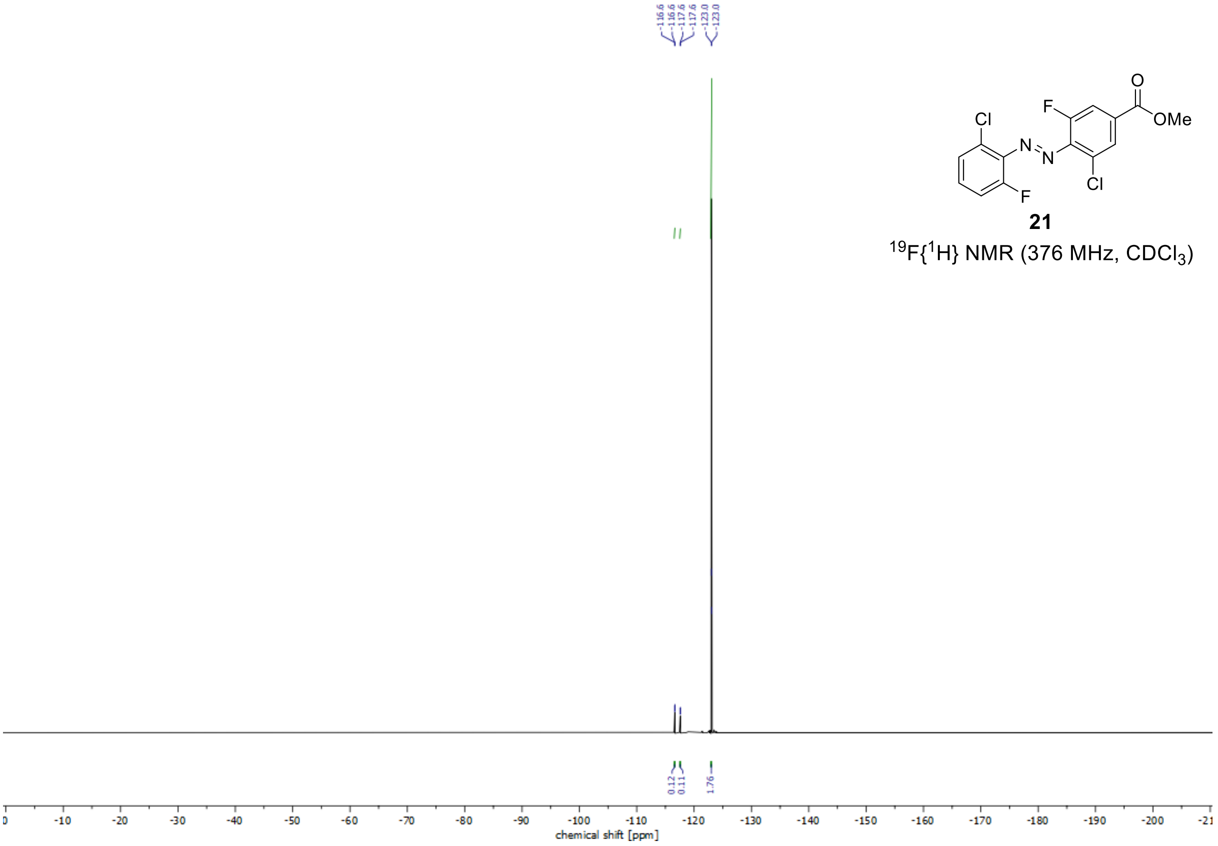


**(*E*/*Z*)-Methyl-3-bromo-4-((2-bromo-6-fluorophenyl)diazenyl)-5-fluorobenzoate (22)**

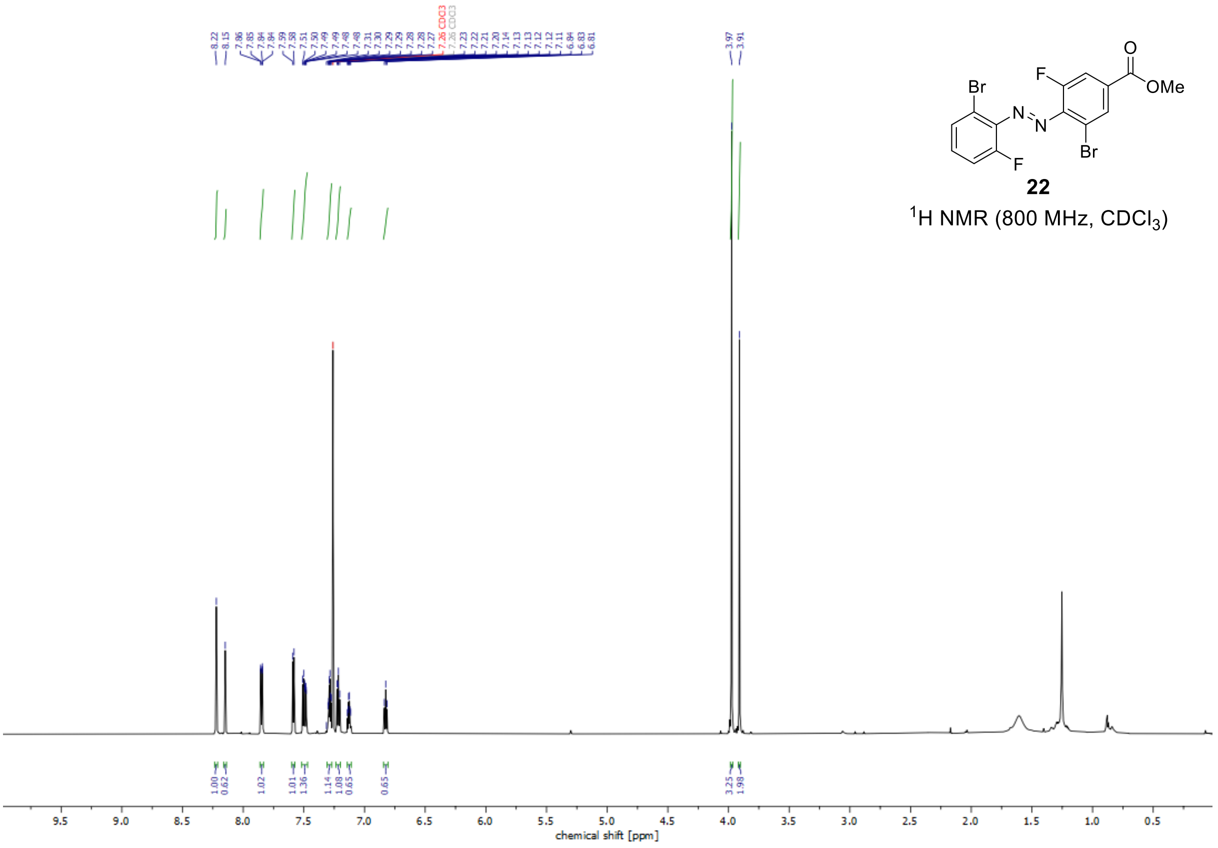

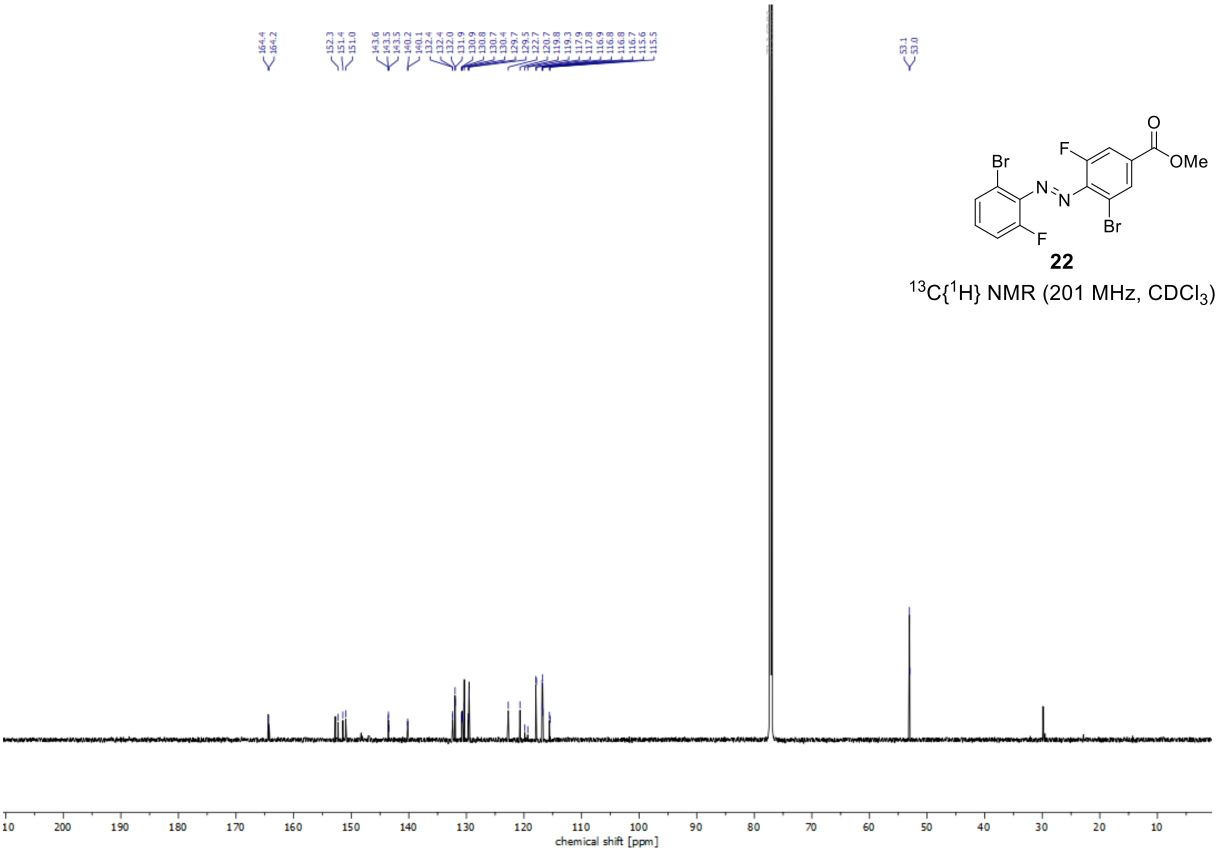


**22**

$^{13}C\{^{1}H\}$ NMR (201 MHz, $CDCl_3$)

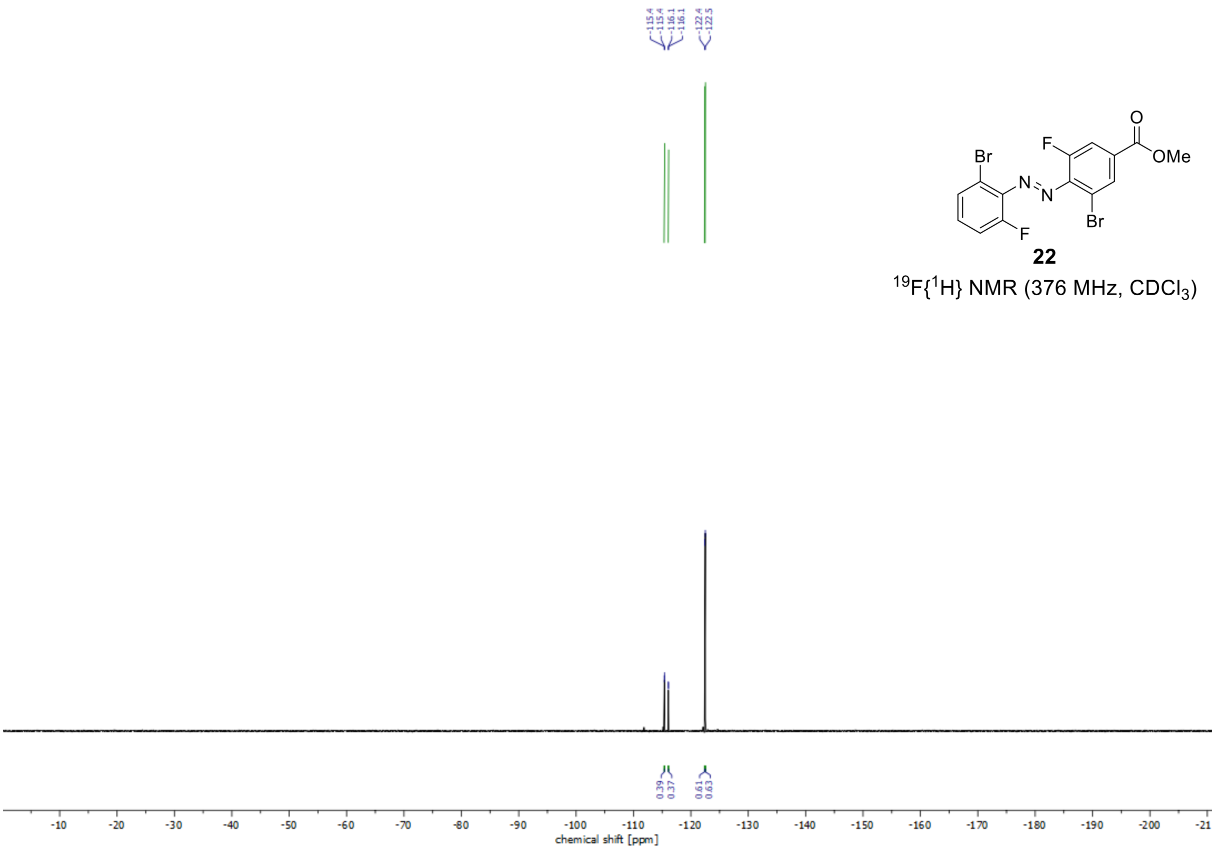


**22**

$^{19}F\{^{1}H\}$ NMR (376 MHz, $CDCl_3$)

**(*E*/*Z*)-Methyl-3,5-dichloro-4-((2,6-dichlorophenyl)diazenyl)benzoate (23)**

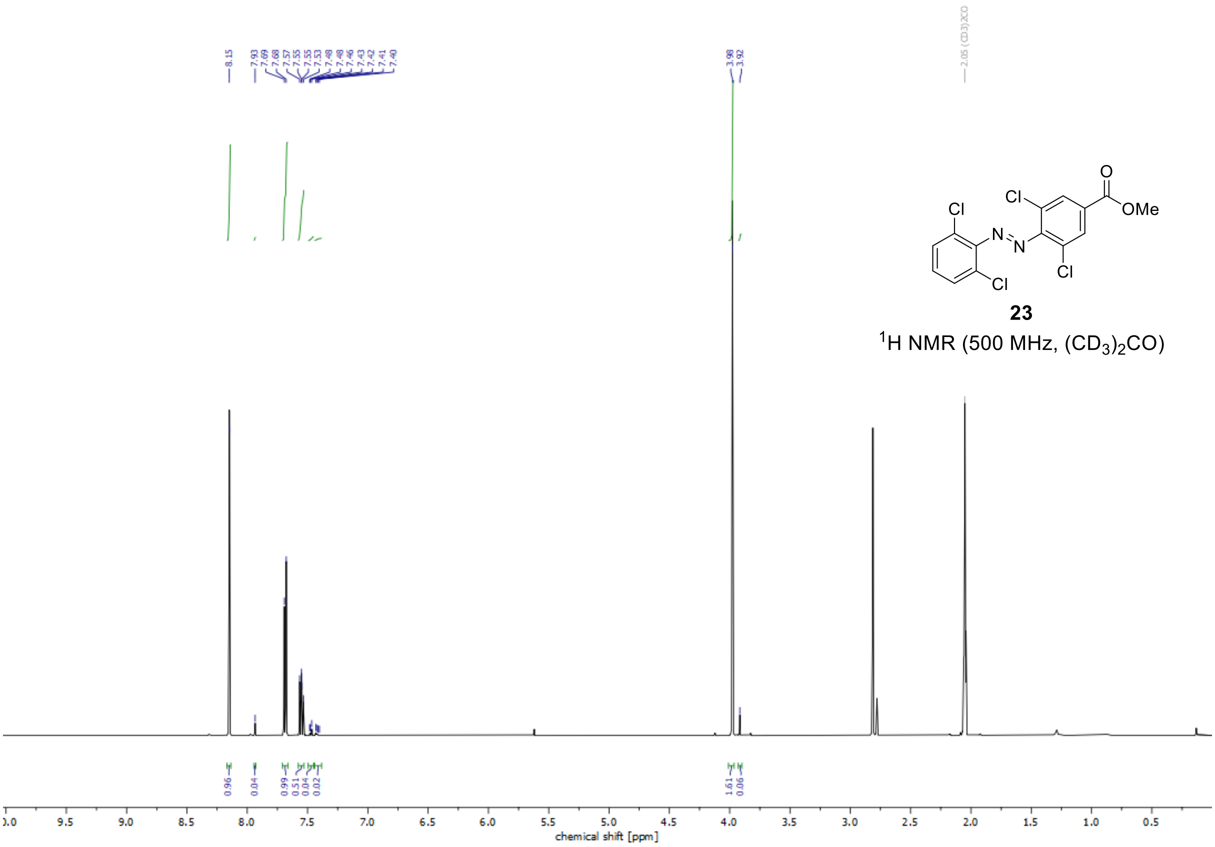


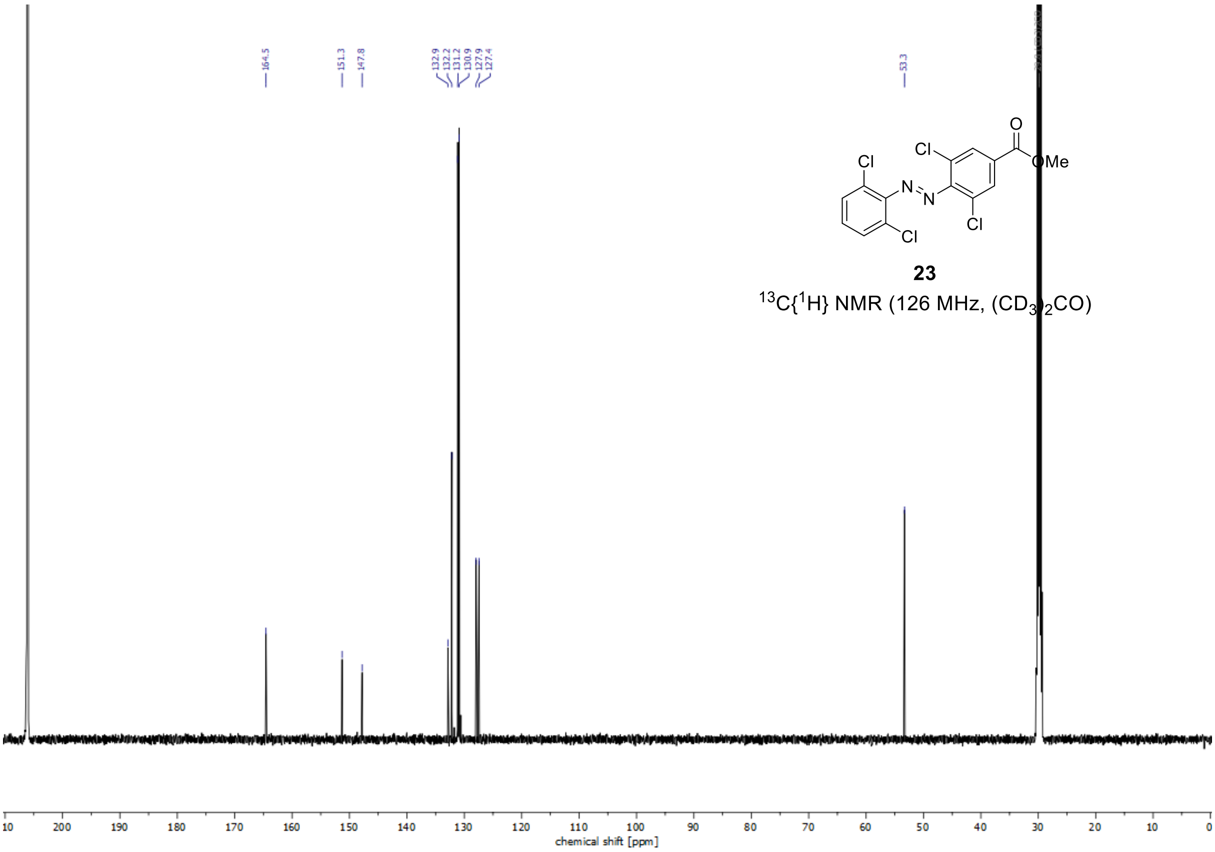

**(*E*/*Z*)-3-Chloro-4-((2-chloro-6-fluorophenyl)diazenyl)-5-fluoro-N-methylbenzamide (24)**

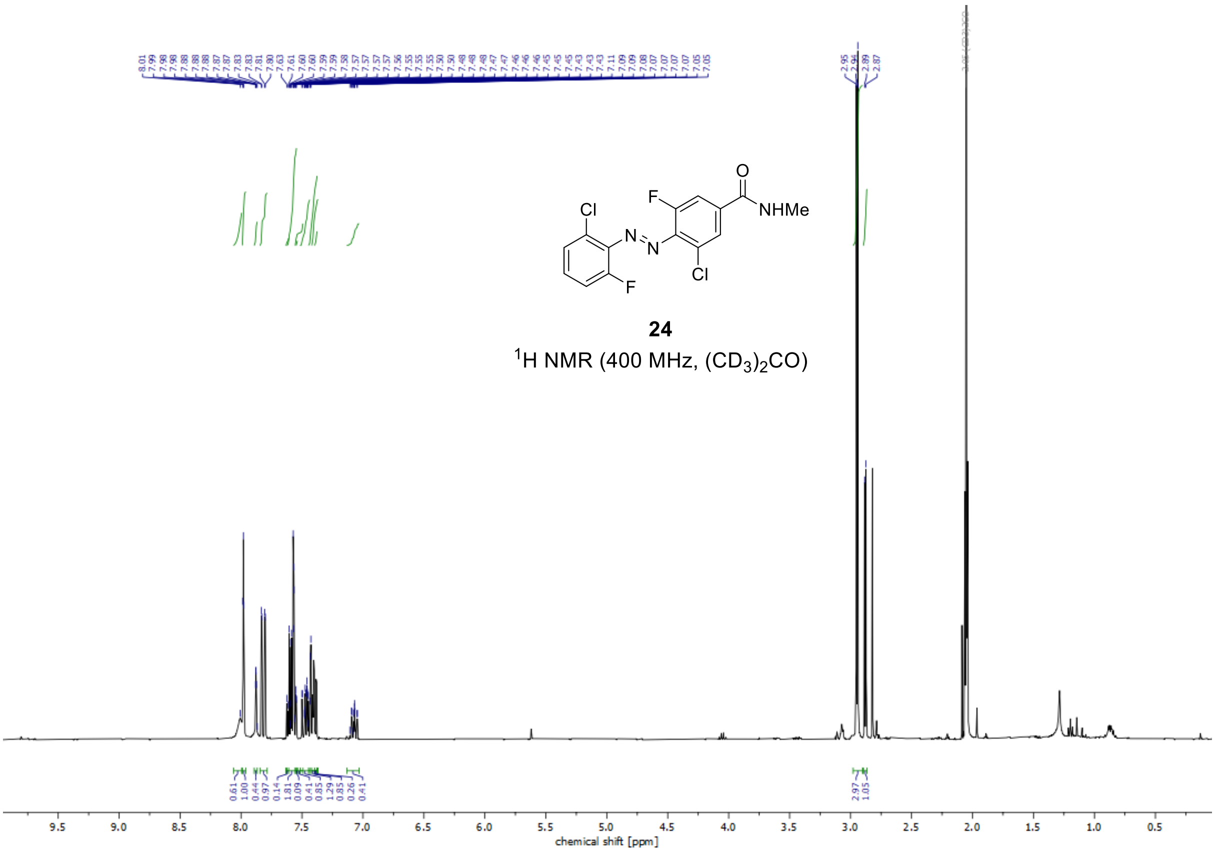


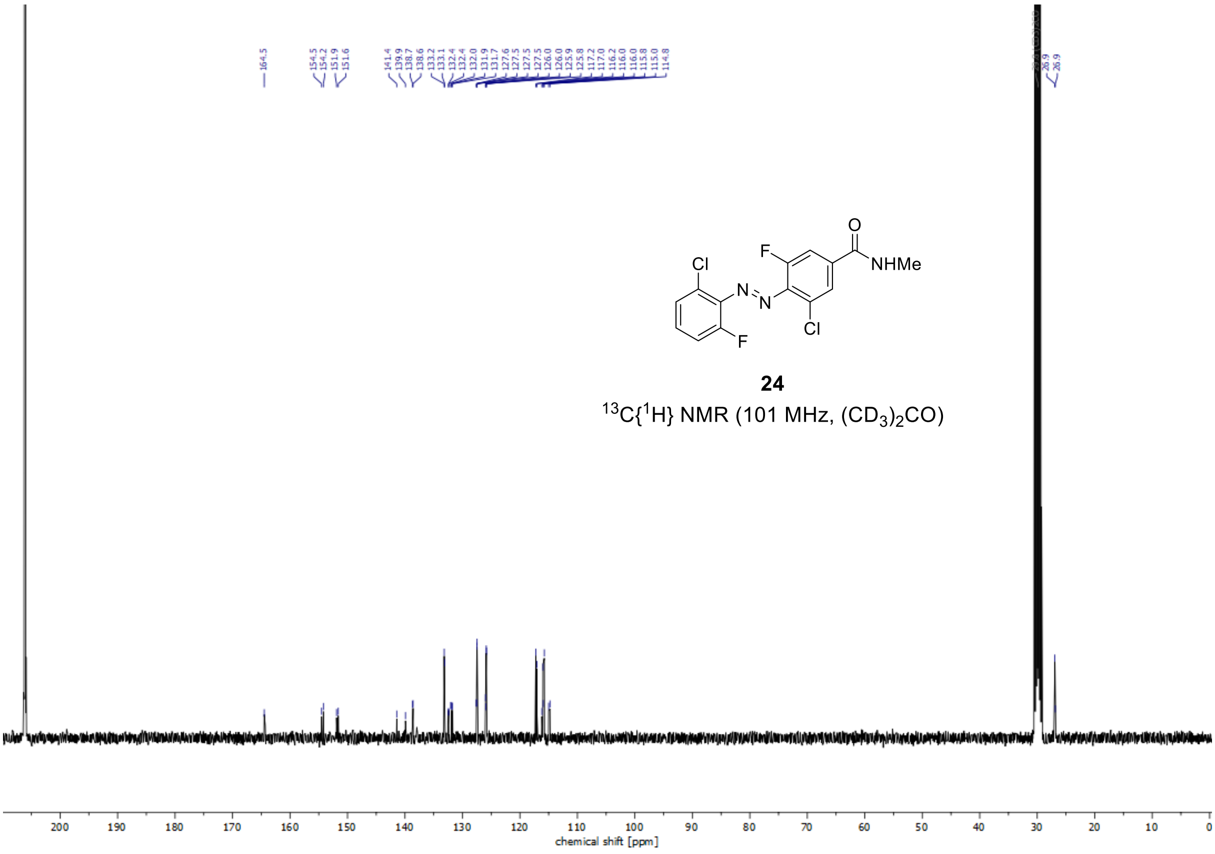

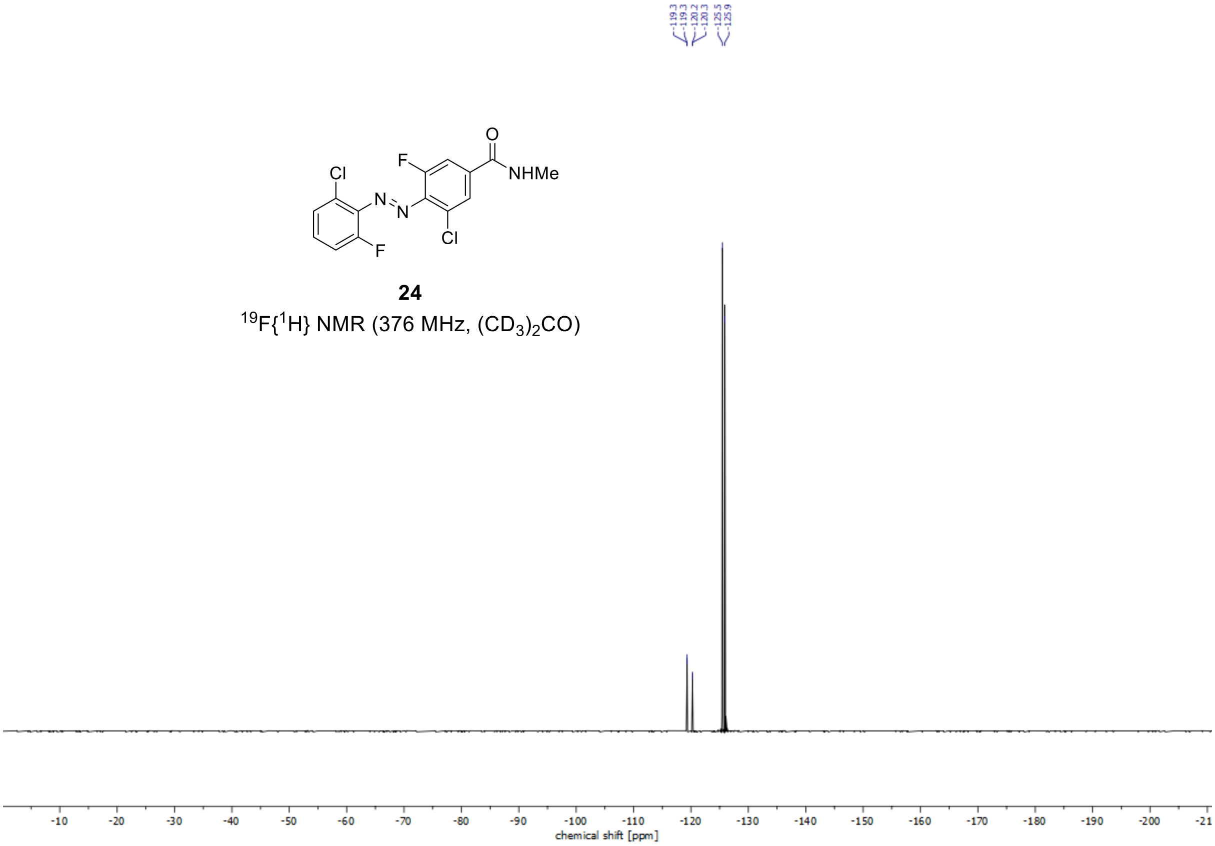


## (*E*)-Dimethyl 4,4'-(diazene-1,2-diyl)(E)-bis(3-chloro-5-fluorobenzoate) (28)

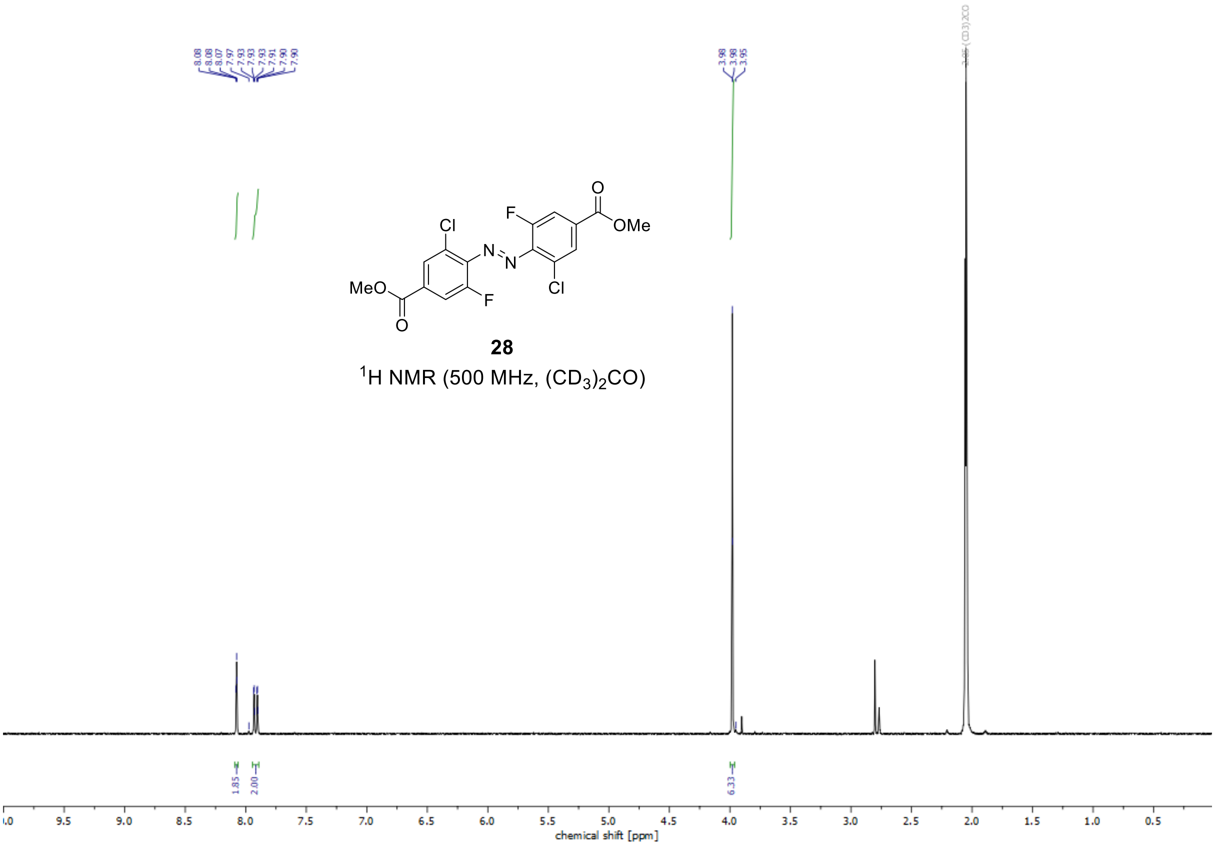

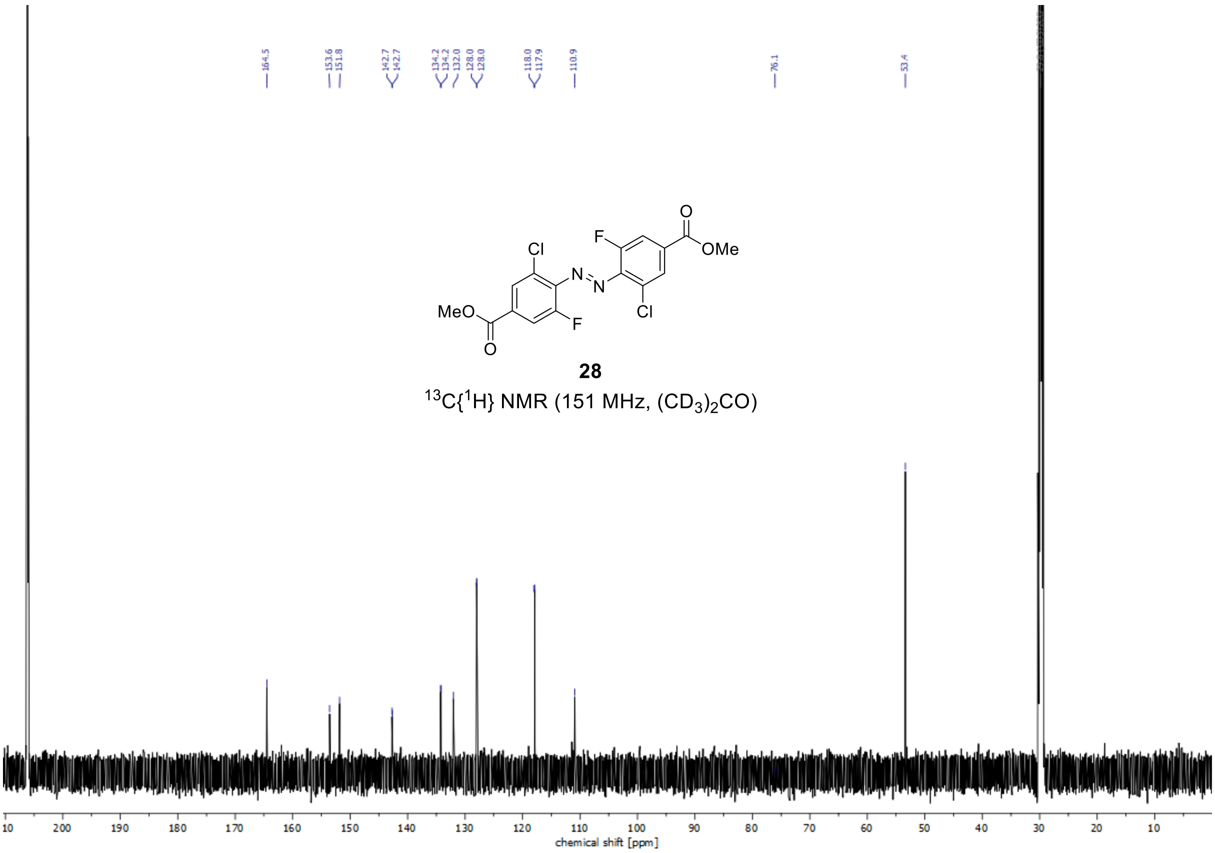


**28**

$^{13}C\{^{1}H\}$ NMR (151 MHz, $(CD_3)_2CO$)

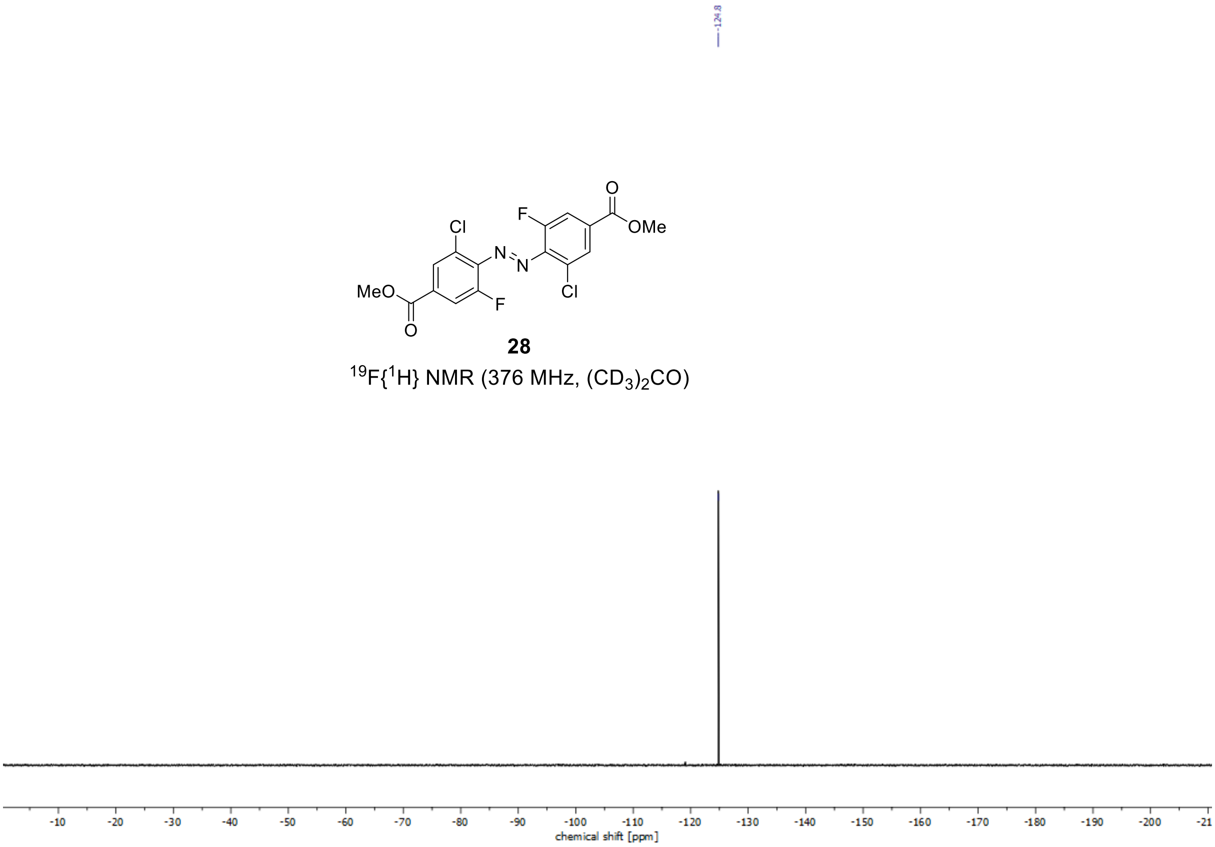


**28**

$^{19}F\{^{1}H\}$ NMR (376 MHz, $(CD_3)_2CO$)

## (*E*/*Z*)-3-Chloro-4-((2-chloro-6-fluorophenyl)diazenyl)-5-fluoroaniline (38)

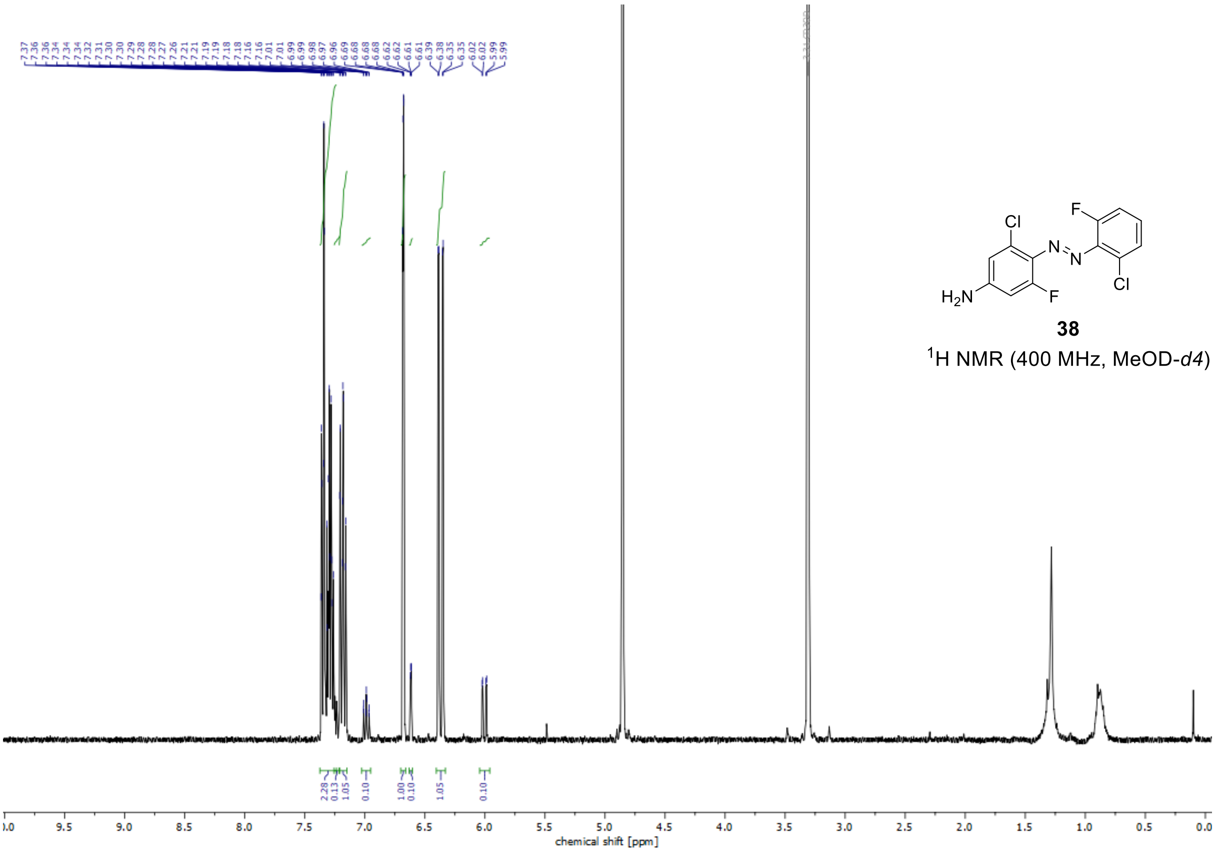


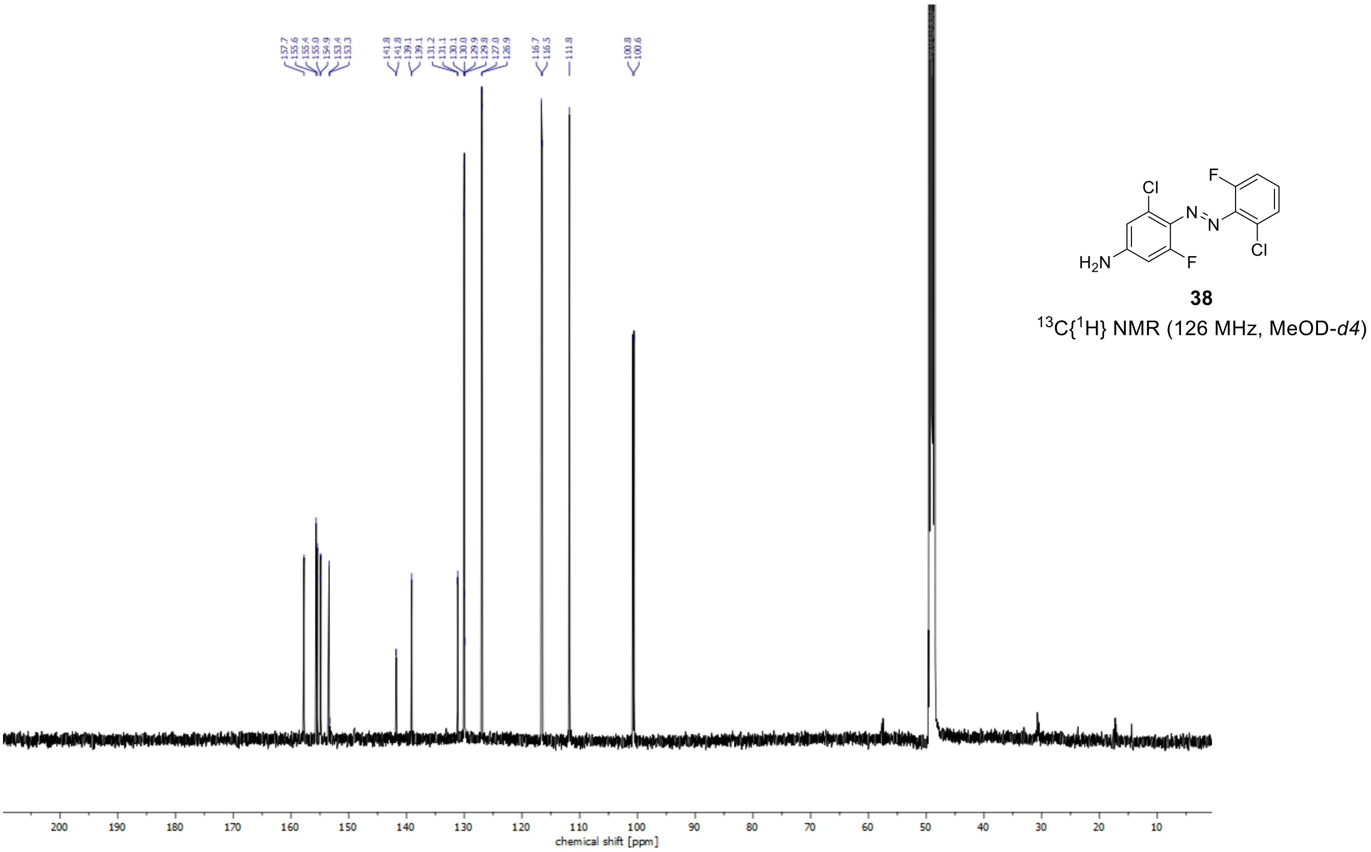

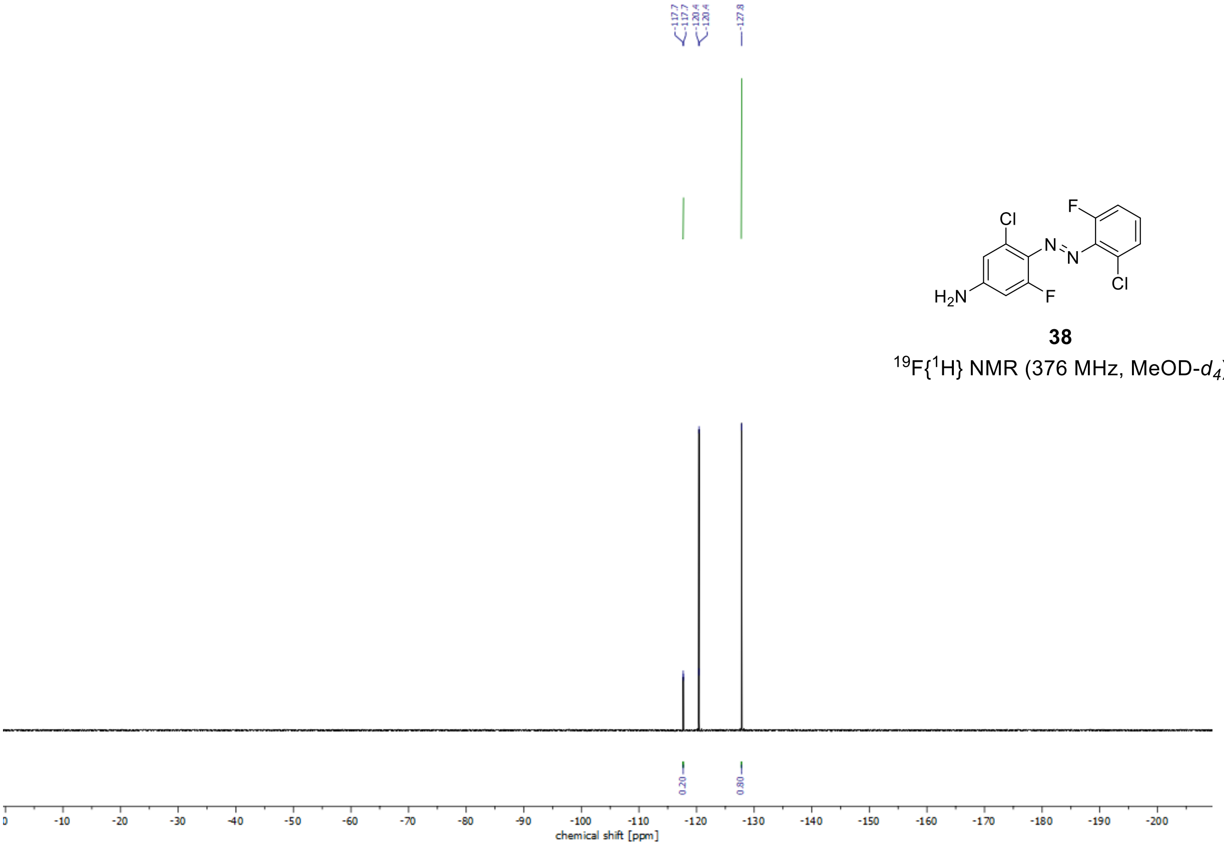


**(*E*/*Z*)-*N*-(3-chloro-4-((2-chloro-6-fluorophenyl)diazenyl)-5-fluorophenyl)acetamide (39)**

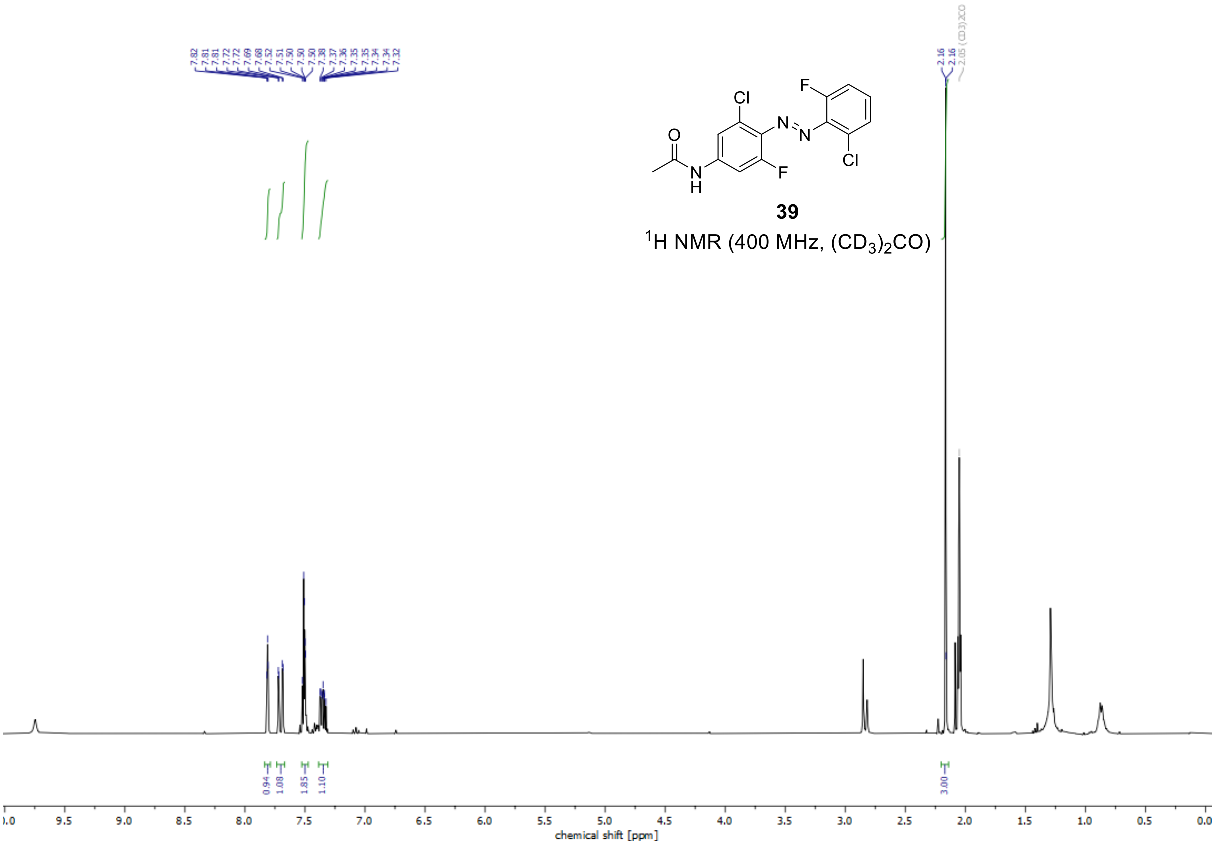

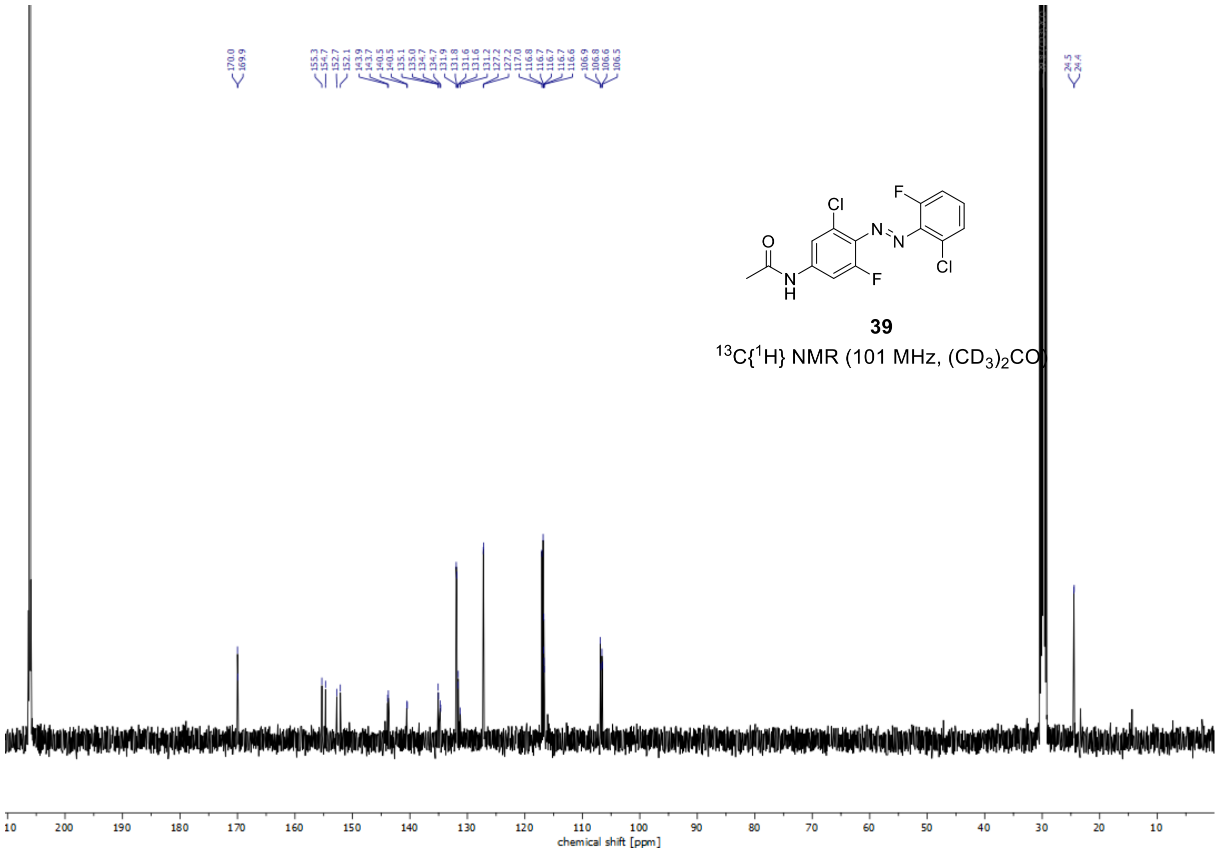
Cl
F
N
N
O
N
H
F
Cl
39
$^{13}C\{^1H\}$ NMR (101 MHz, $(CD_3)_2CO$)
chemical shift [ppm]

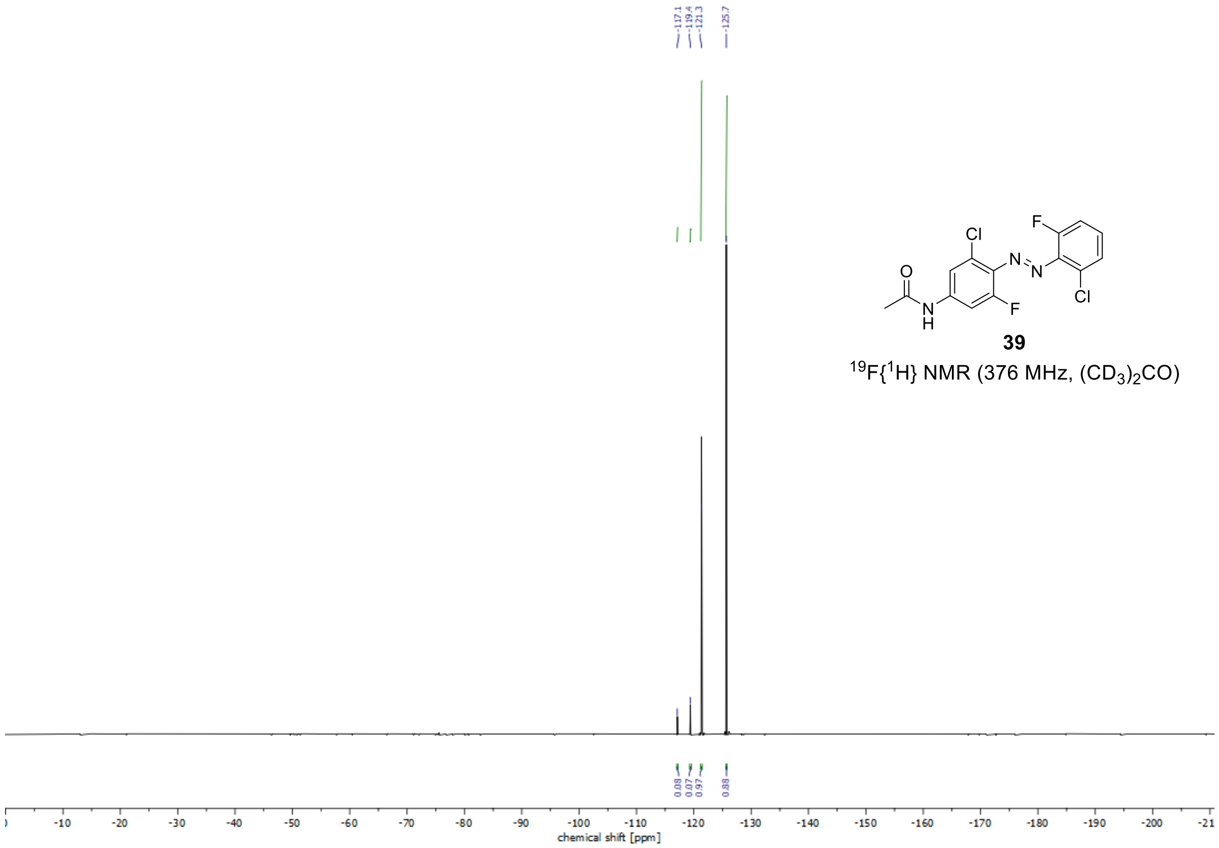
Cl
F
N
N
O
N
H
F
Cl
39
$^{19}F\{^1H\}$ NMR (376 MHz, $(CD_3)_2CO$)
chemical shift [ppm]

**(*E*/*Z*)-Methyl-3-chloro-4-((2-chloro-6-fluoro-4-methoxyphenyl)diazenyl)-5-fluorobenzoate (46)**

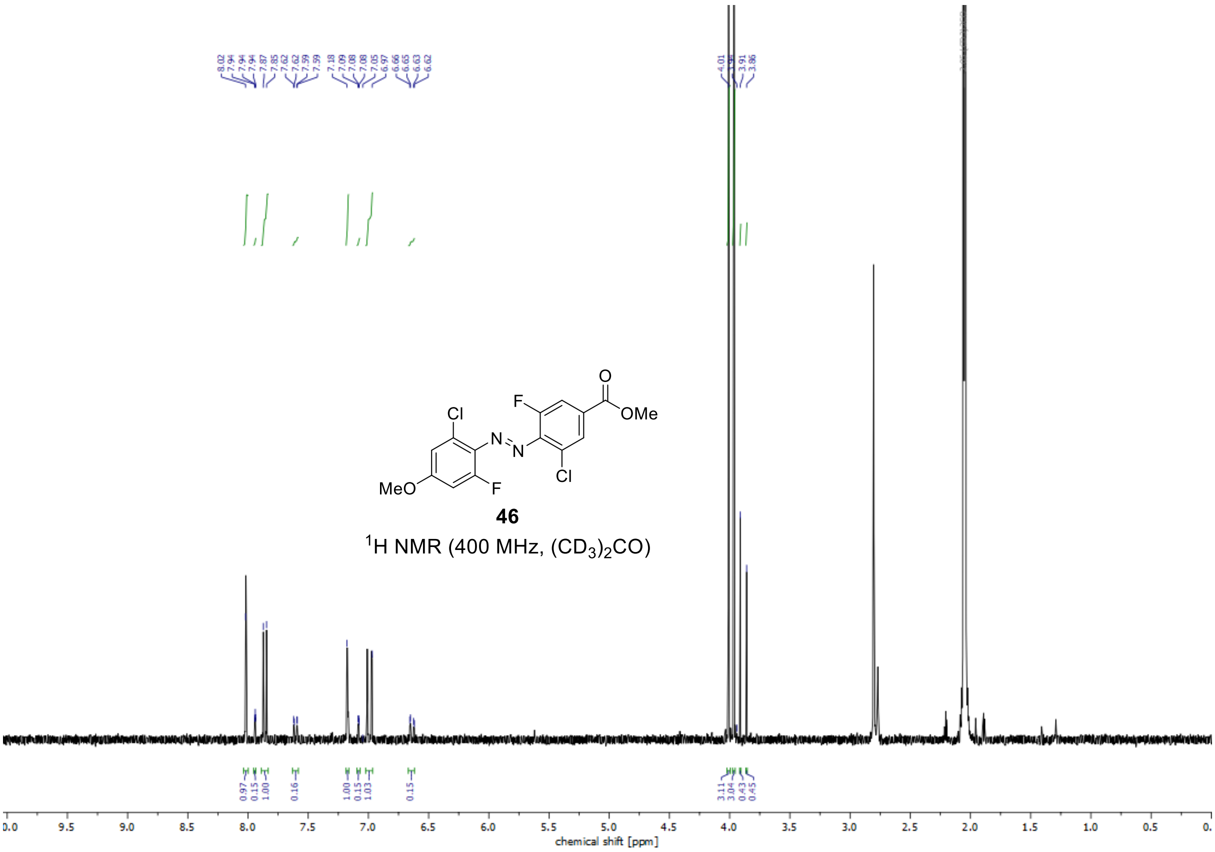


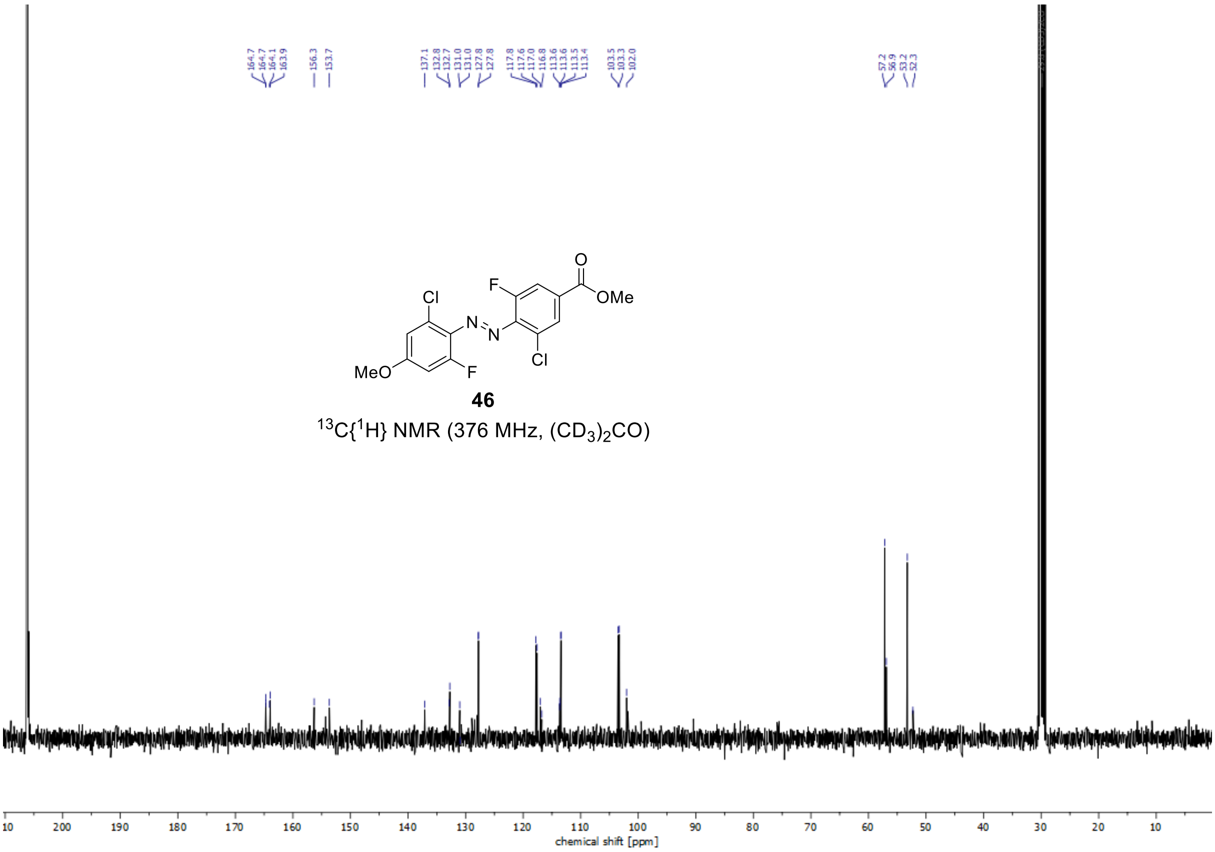

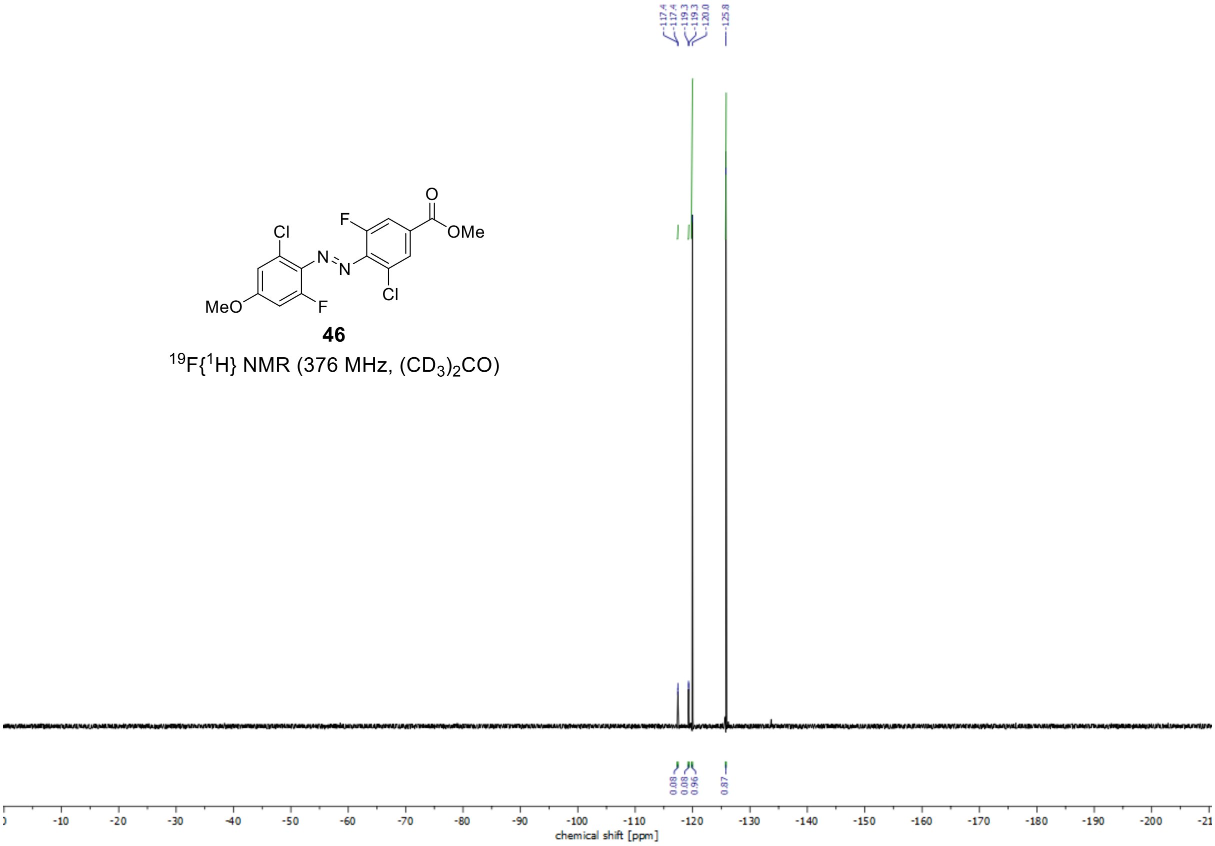


**(*E*)-4-((4-Amino-2,6-dichlorophenyl)diazenyl)-3,5-dichlorobenzenesulfonamide (48)**

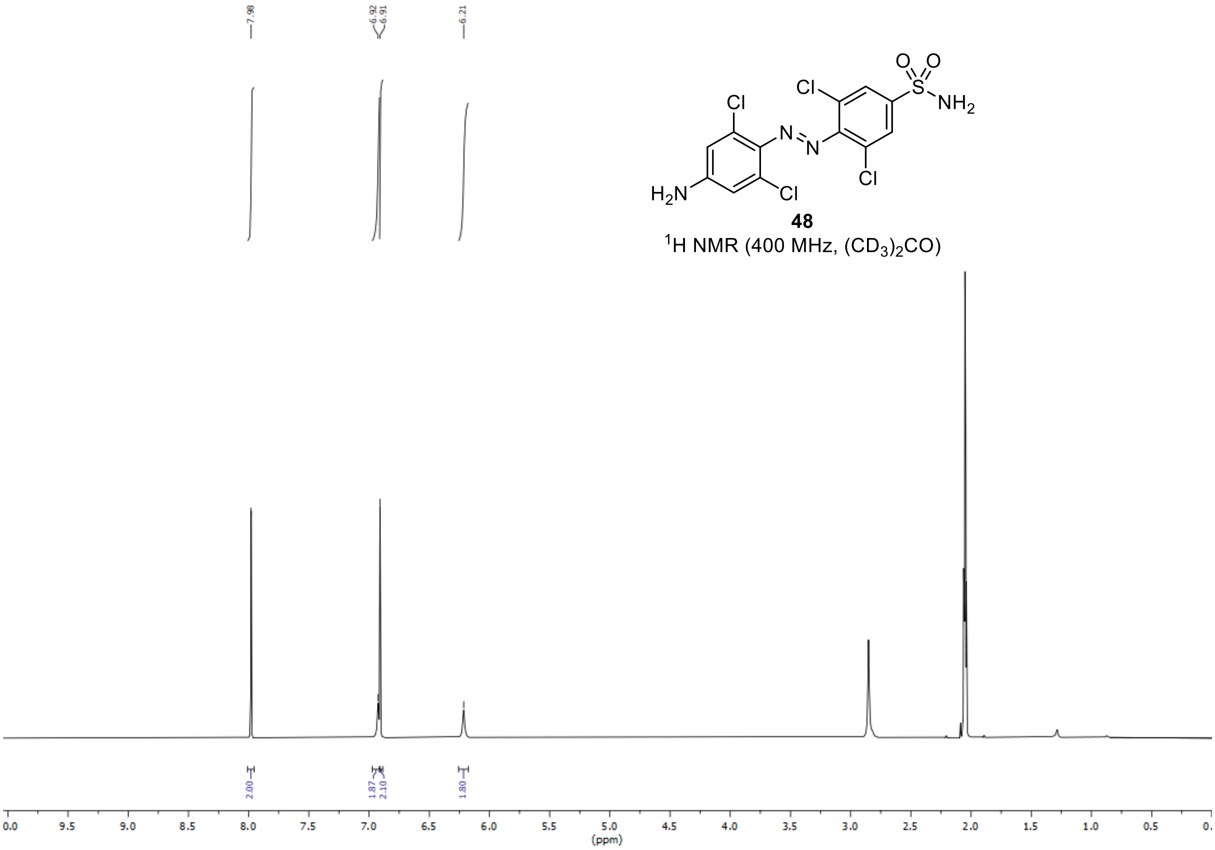

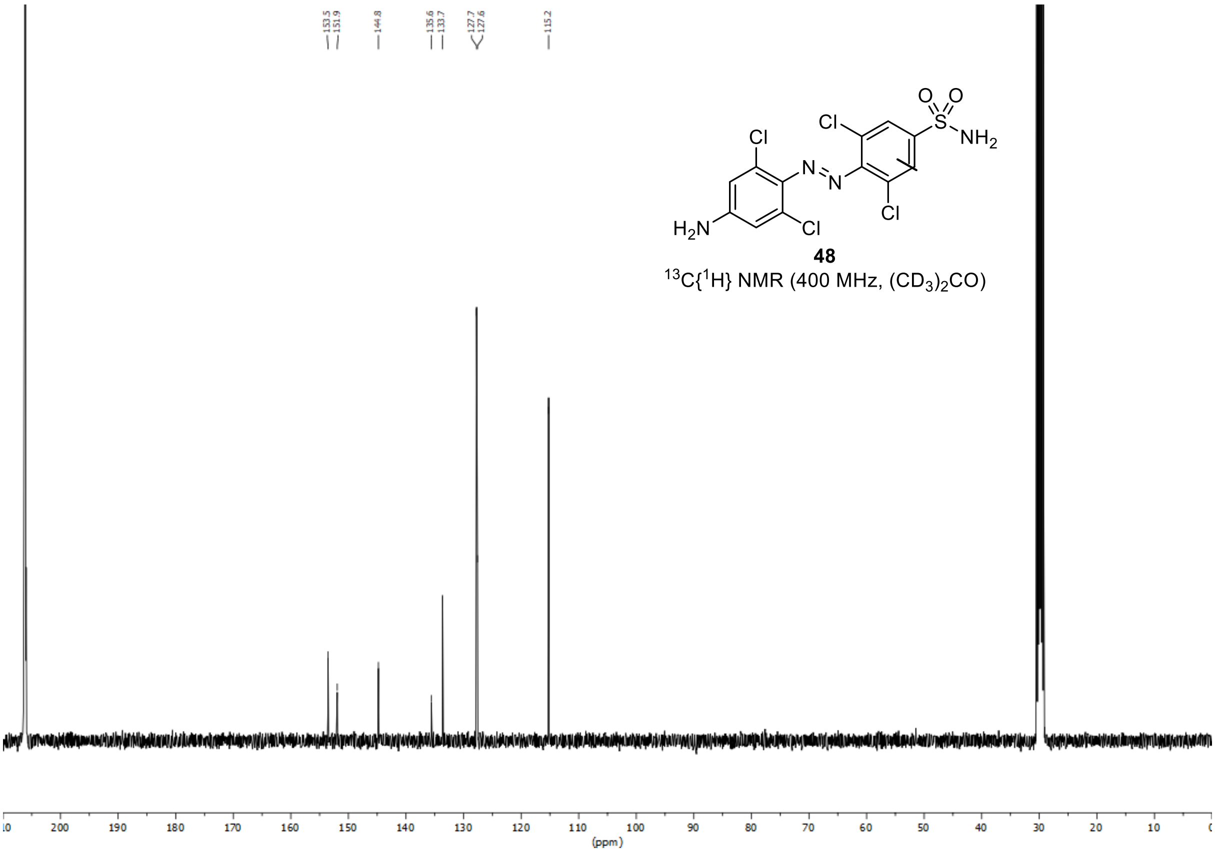


**(*E*)-3-Chloro-4-((2-chloro-6-fluoro-4-hydroxyphenyl)diazenyl)-5-fluorobenzoic acid (49)**

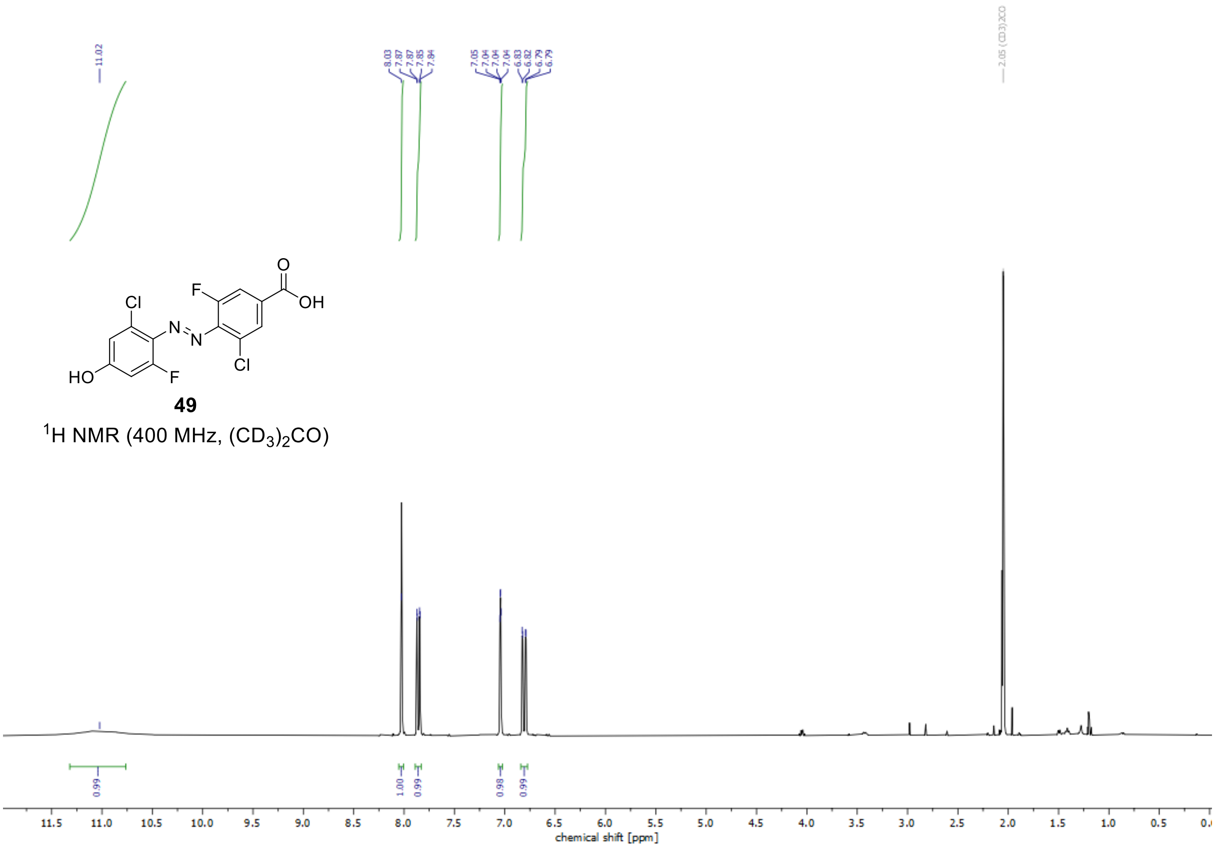

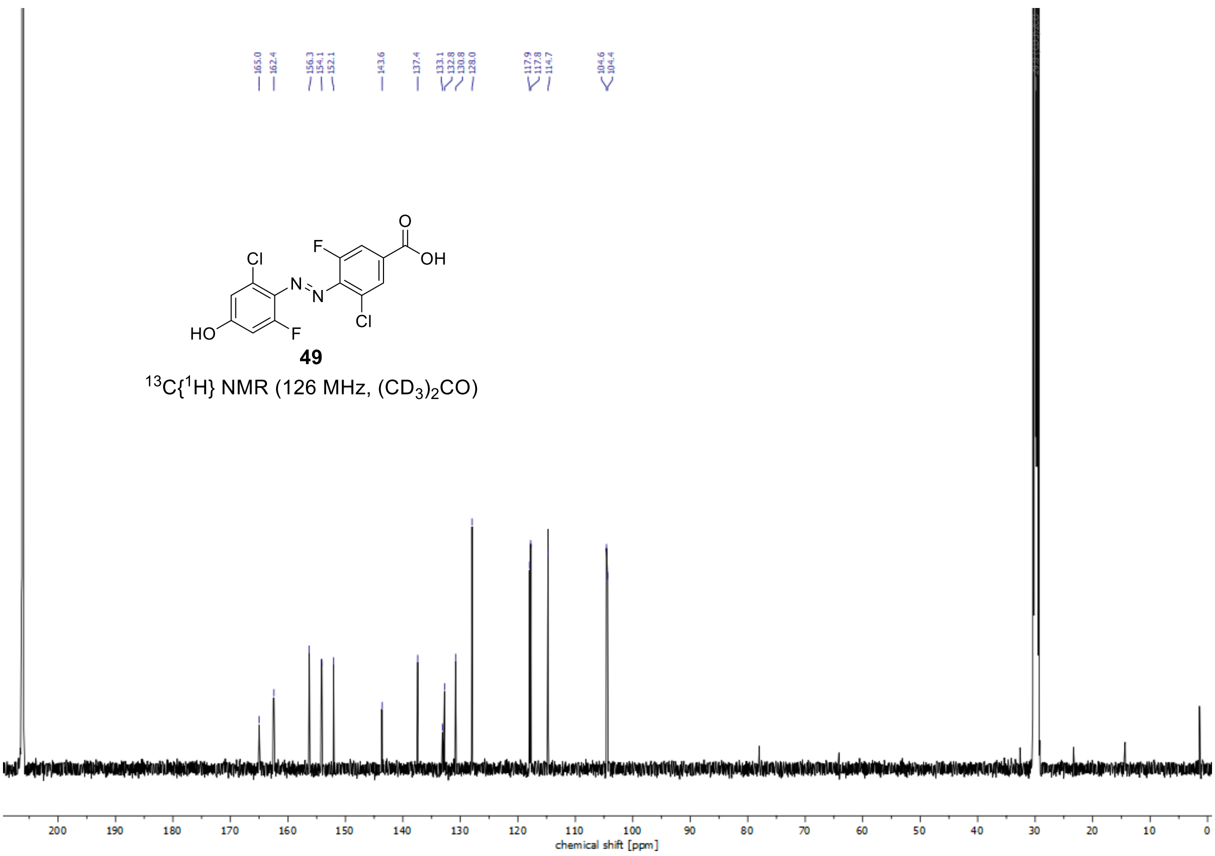
165.0
162.4
156.3
154.1
152.1
143.6
137.4
133.1
132.8
130.8
128.0
117.9
117.8
114.7
104.6
104.4
O
F
OH
Cl
N
N
Cl
HO
F
49
$^{13}C\{^{1}H\}$ NMR (126 MHz, $(CD_3)_2CO$)
200 190 180 170 160 150 140 130 120 110 100 90 80 70 60 50 40 30 20 10 0
chemical shift [ppm]

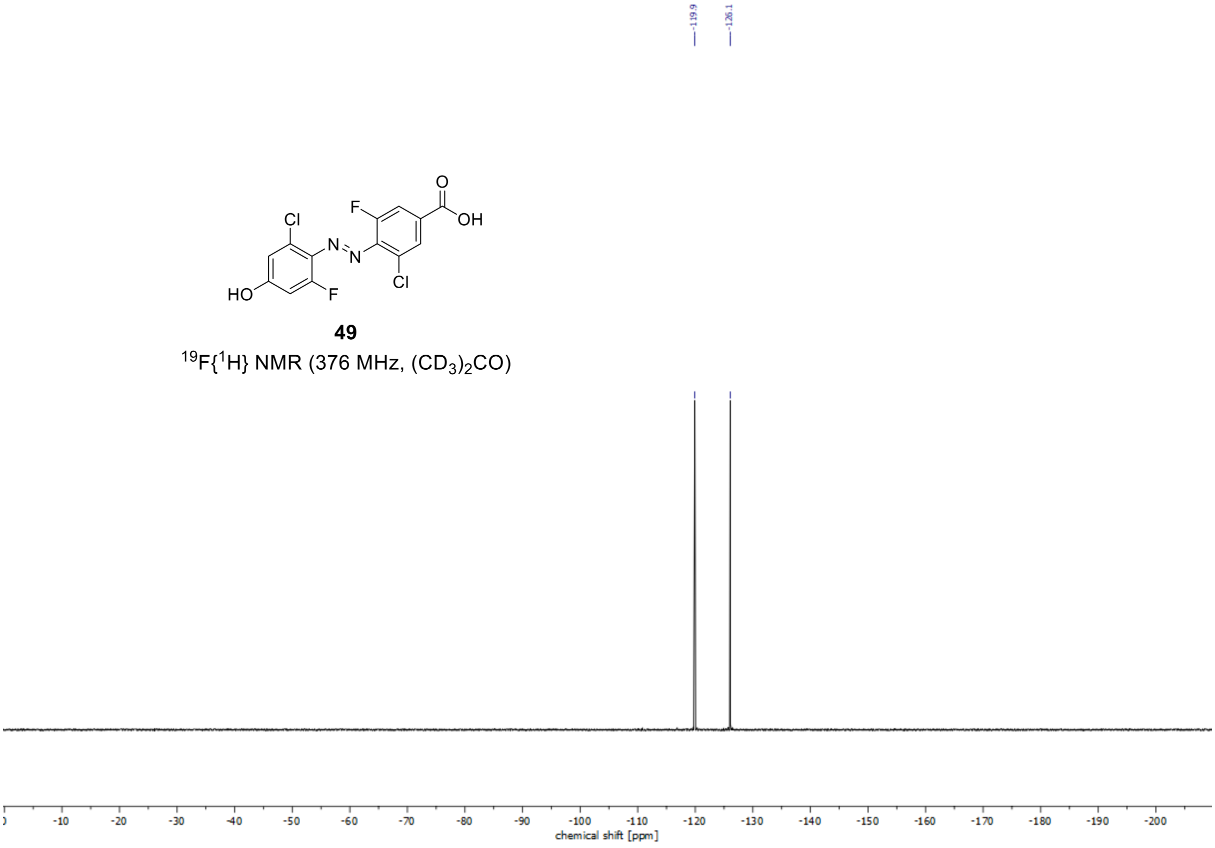
-119.9
-126.1
O
F
OH
Cl
N
N
Cl
HO
F
49
$^{19}F\{^{1}H\}$ NMR (376 MHz, $(CD_3)_2CO$)
-10 -20 -30 -40 -50 -60 -70 -80 -90 -100 -110 -120 -130 -140 -150 -160 -170 -180 -190 -200
chemical shift [ppm]

**(*E*/*Z*)-1-(1-(4-(4-((4-Butyl-2-chloro-6-fluorophenyl)diazenyl)-3-chloro-5-fluorophenyl)butanoyl)-piperidin-4-yl)-1,3-dihydro-2*H*-benzo[*d*]imidazol-2-one (dfdc-OptoBI-1)**

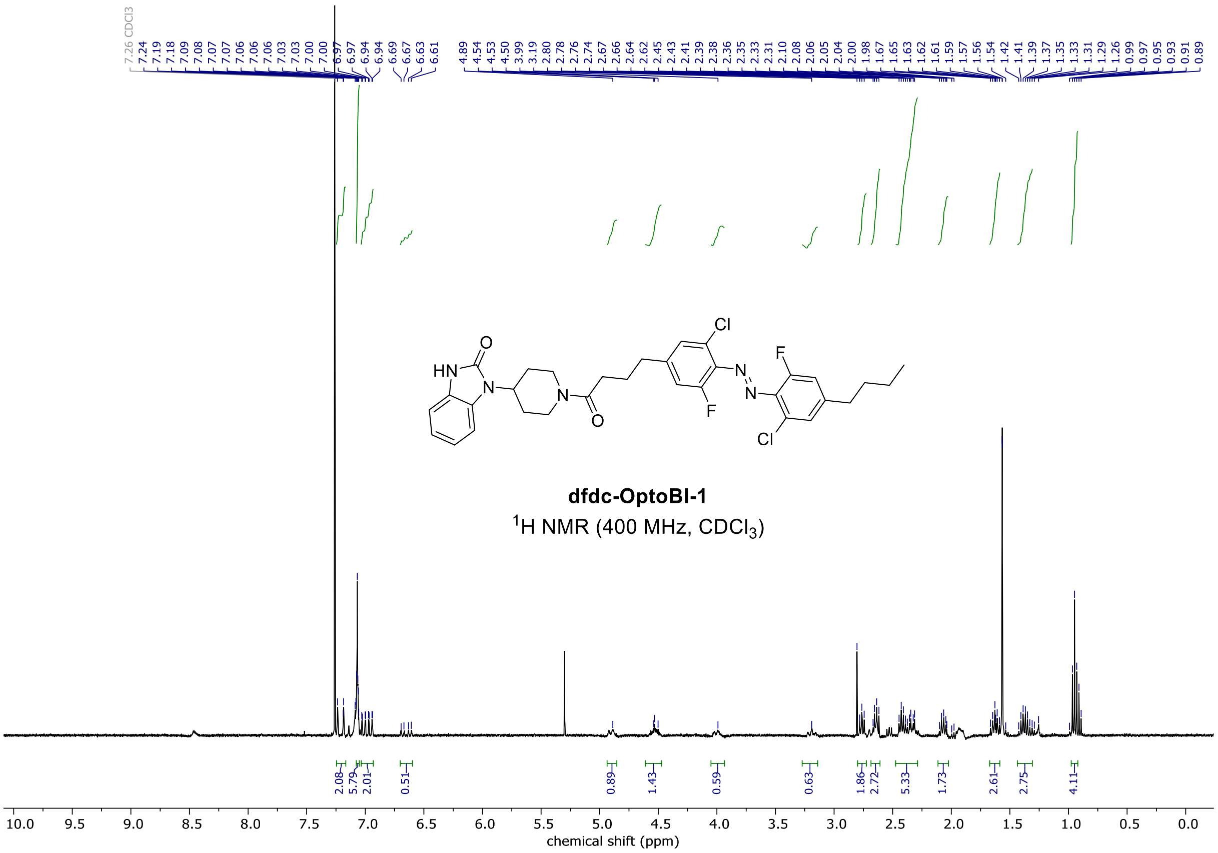


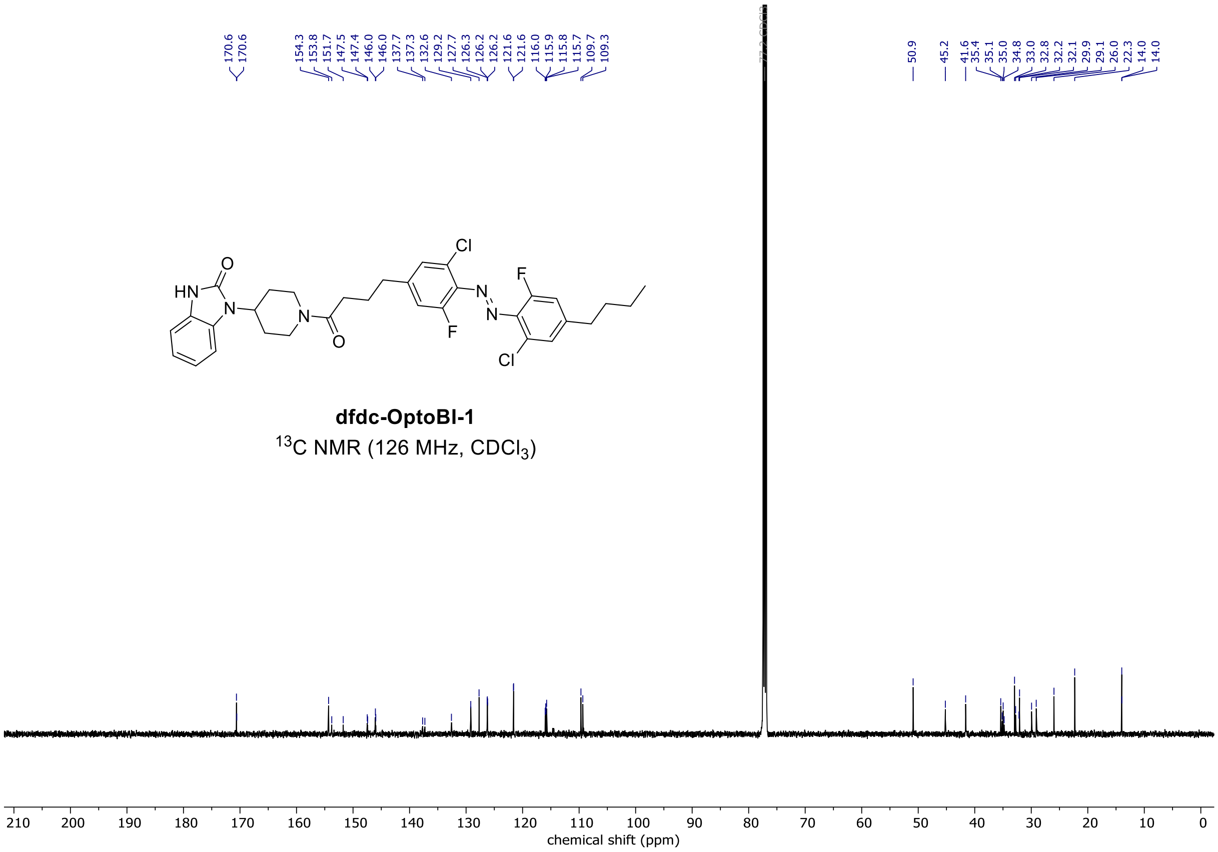

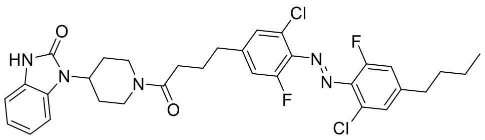


**dfdc-OptoBI-1**

$^{19}$F NMR (376 MHz, $CDCl_3$)

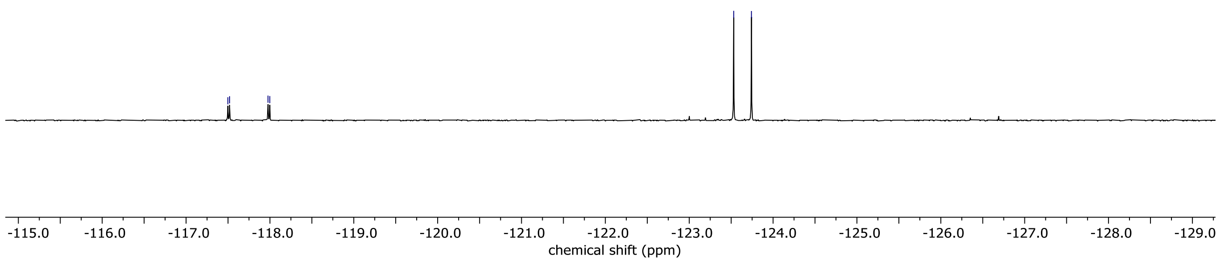

## Precursor

## (*E*)-1,2-Bis(1-fluoronaphthalen-2-yl)diazene (58)

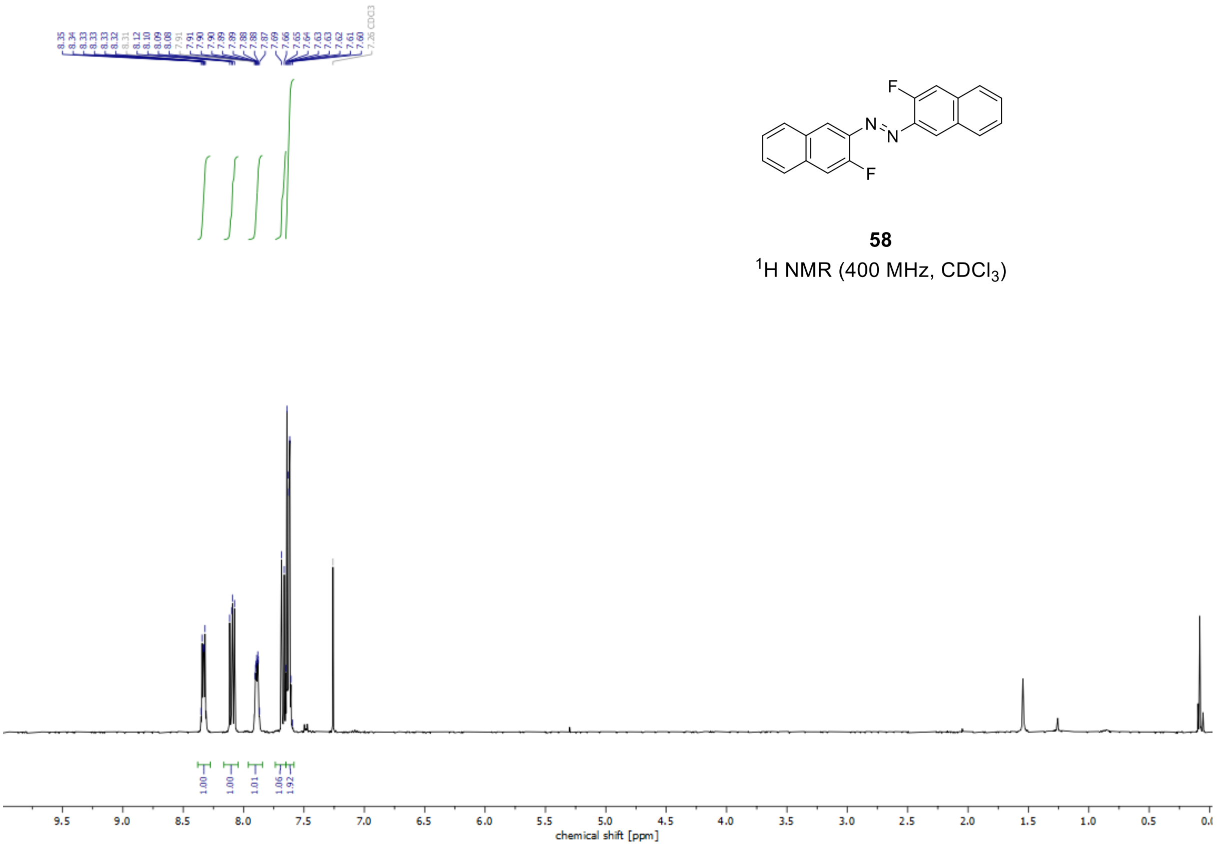


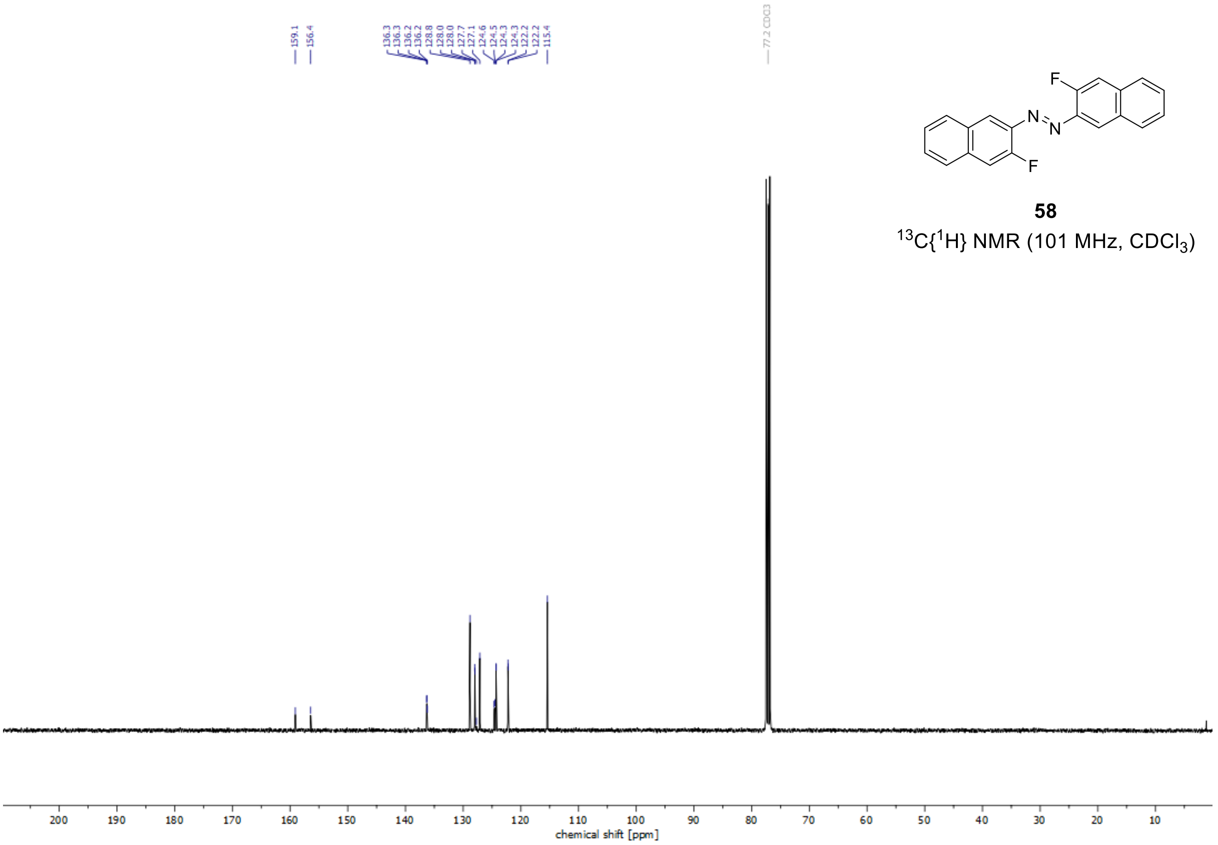

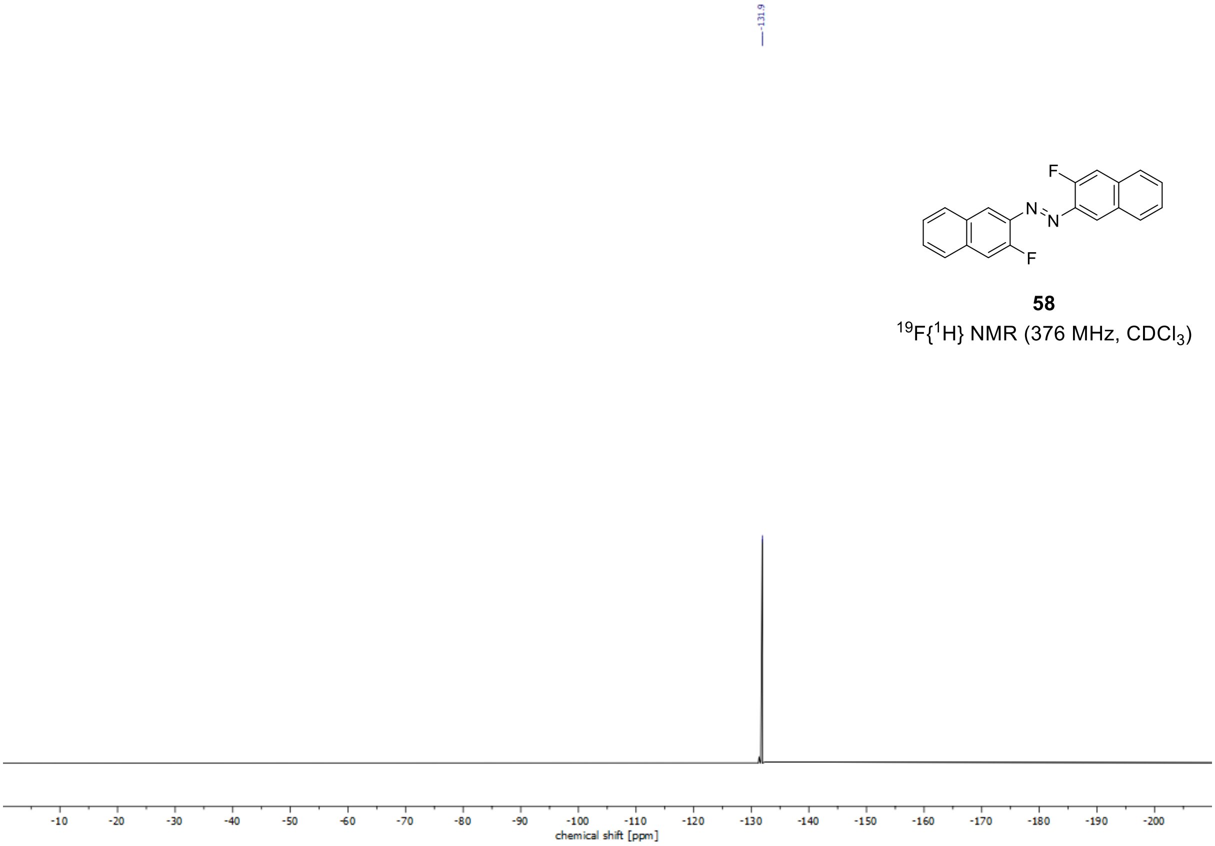


## (*E*)-1-(2-Fluoro-4-nitrophenyl)-2-(2-fluorophenyl)diazene (59)

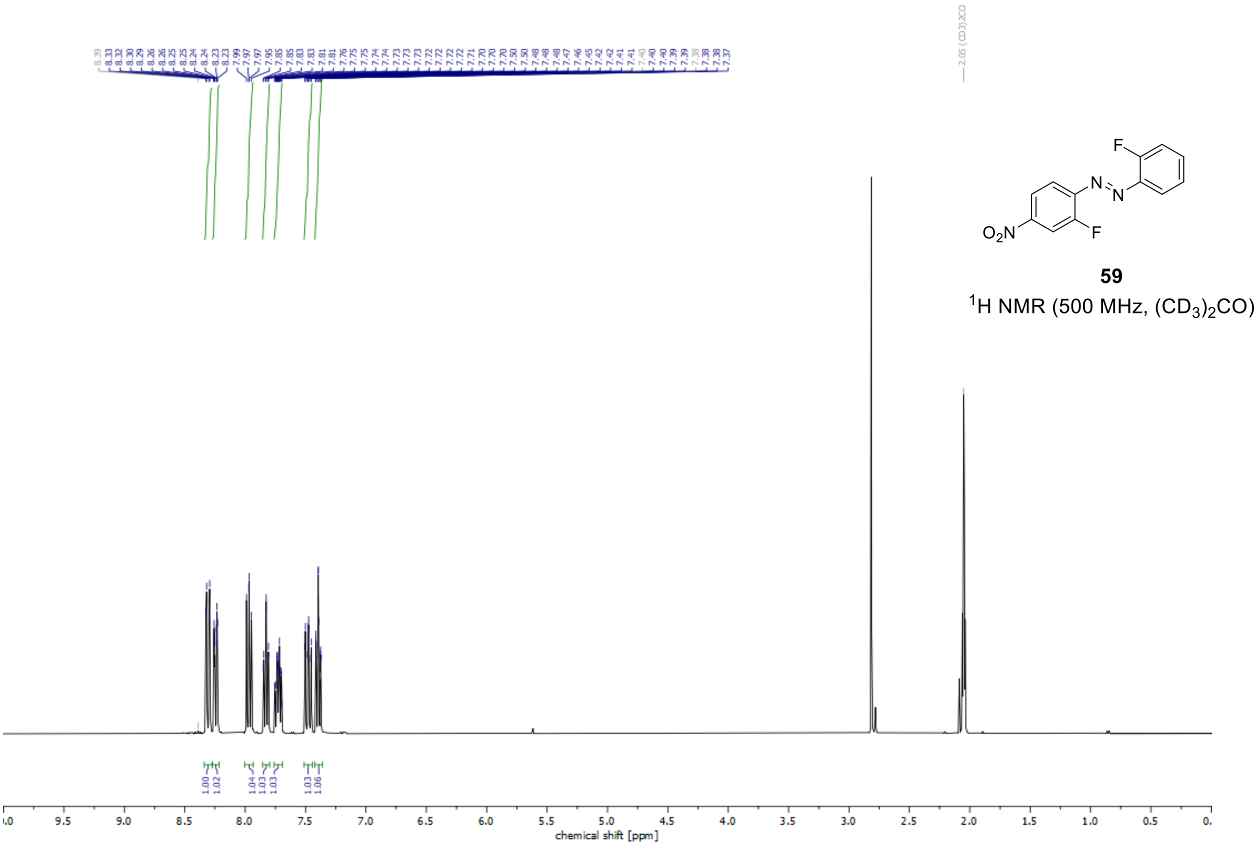

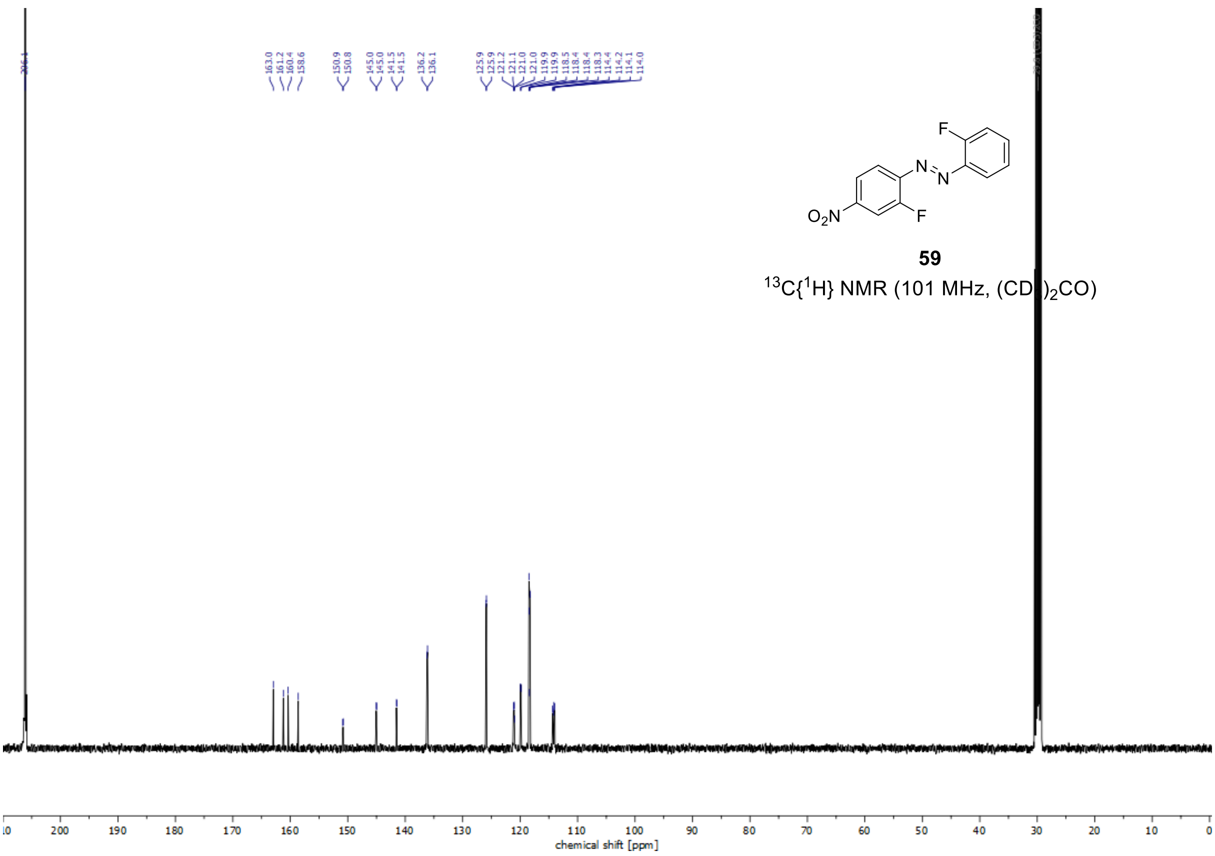


**59**

$^{13}C\{^{1}H\}$ NMR (101 MHz, $(CD_3)_2CO$)

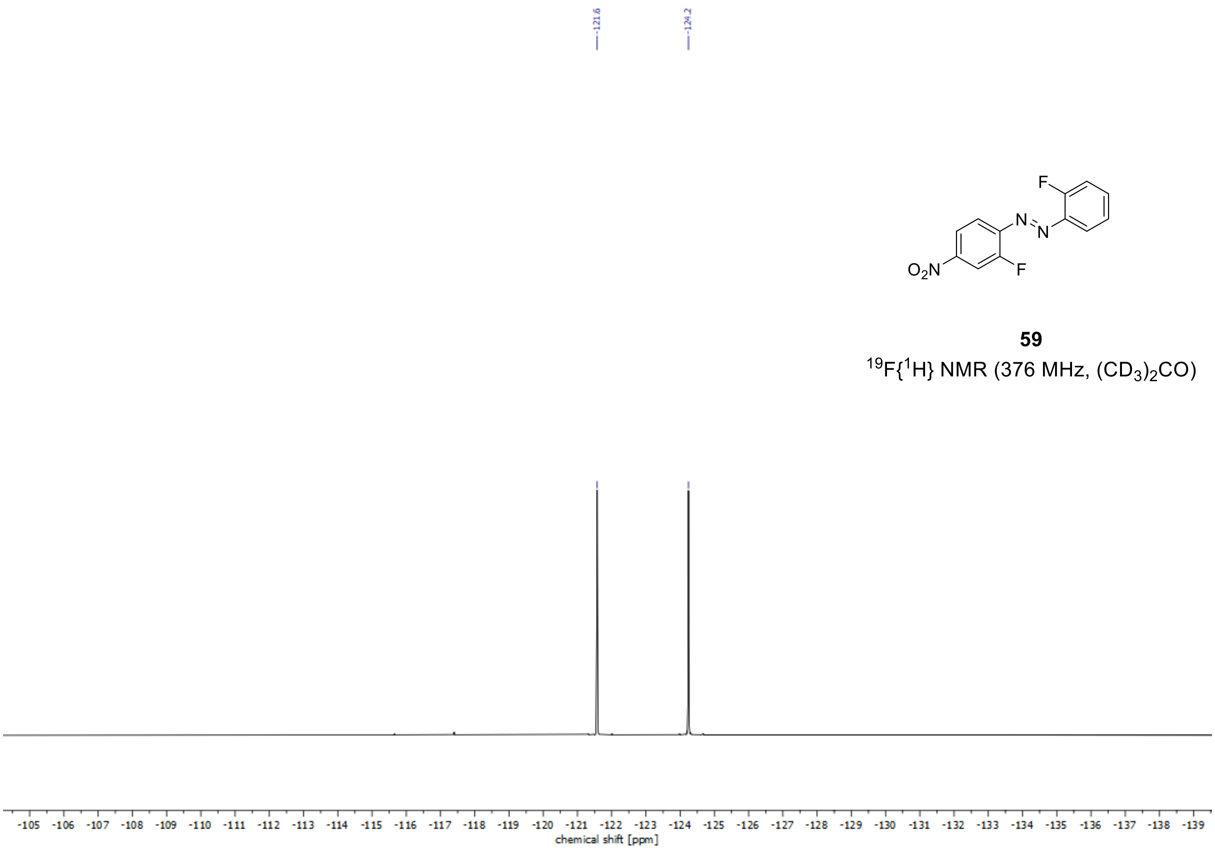


**59**

$^{19}F\{^{1}H\}$ NMR (376 MHz, $(CD_3)_2CO$)

**(*E*)-3-Fluoro-4-((2-fluoro-4-nitrophenyl)diazenyl)benzoic aciddiazene (60)**

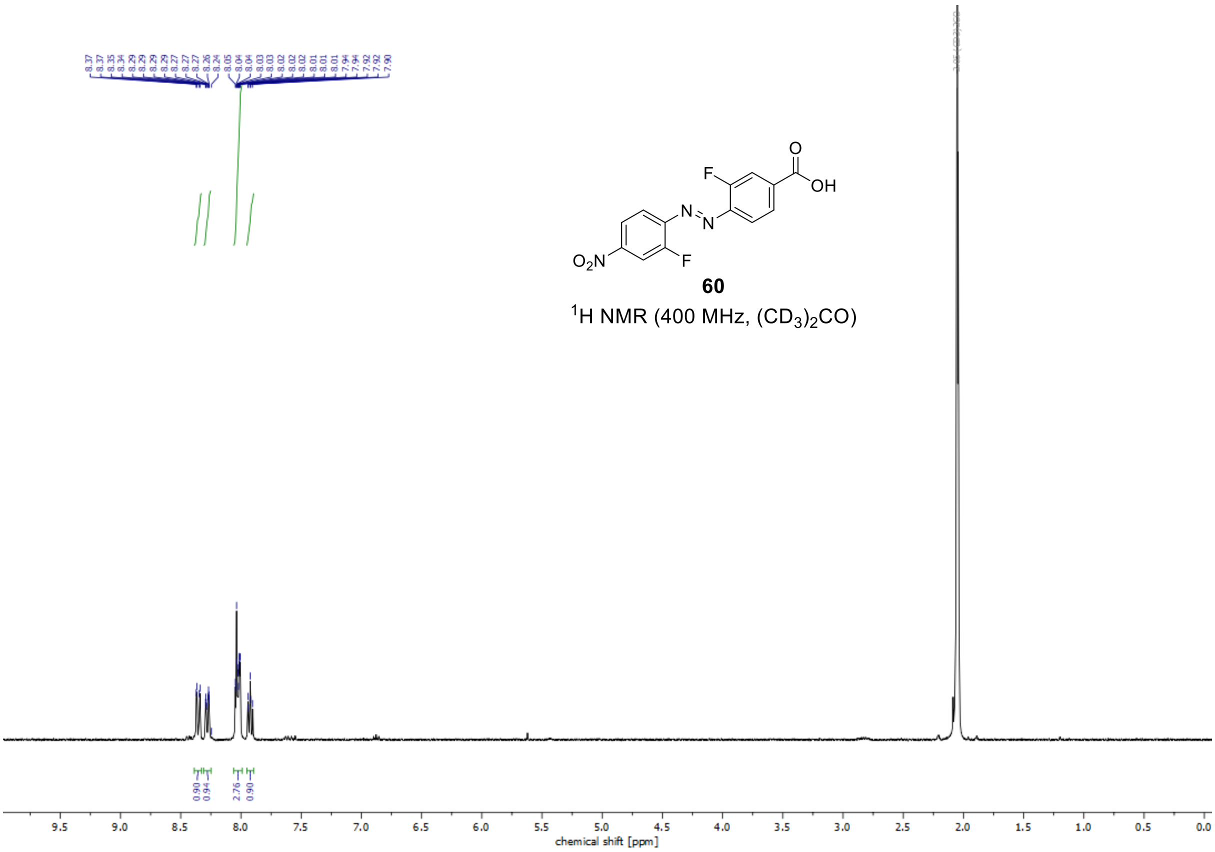


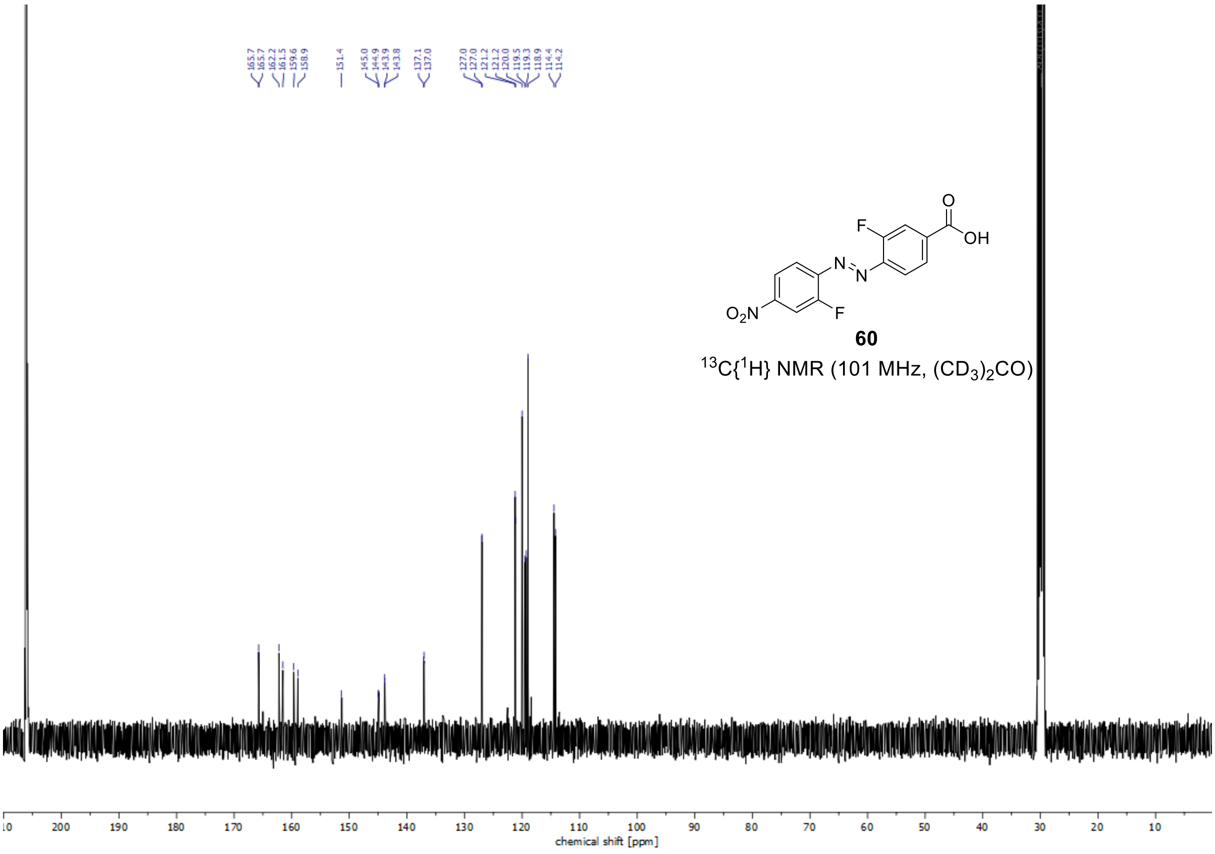

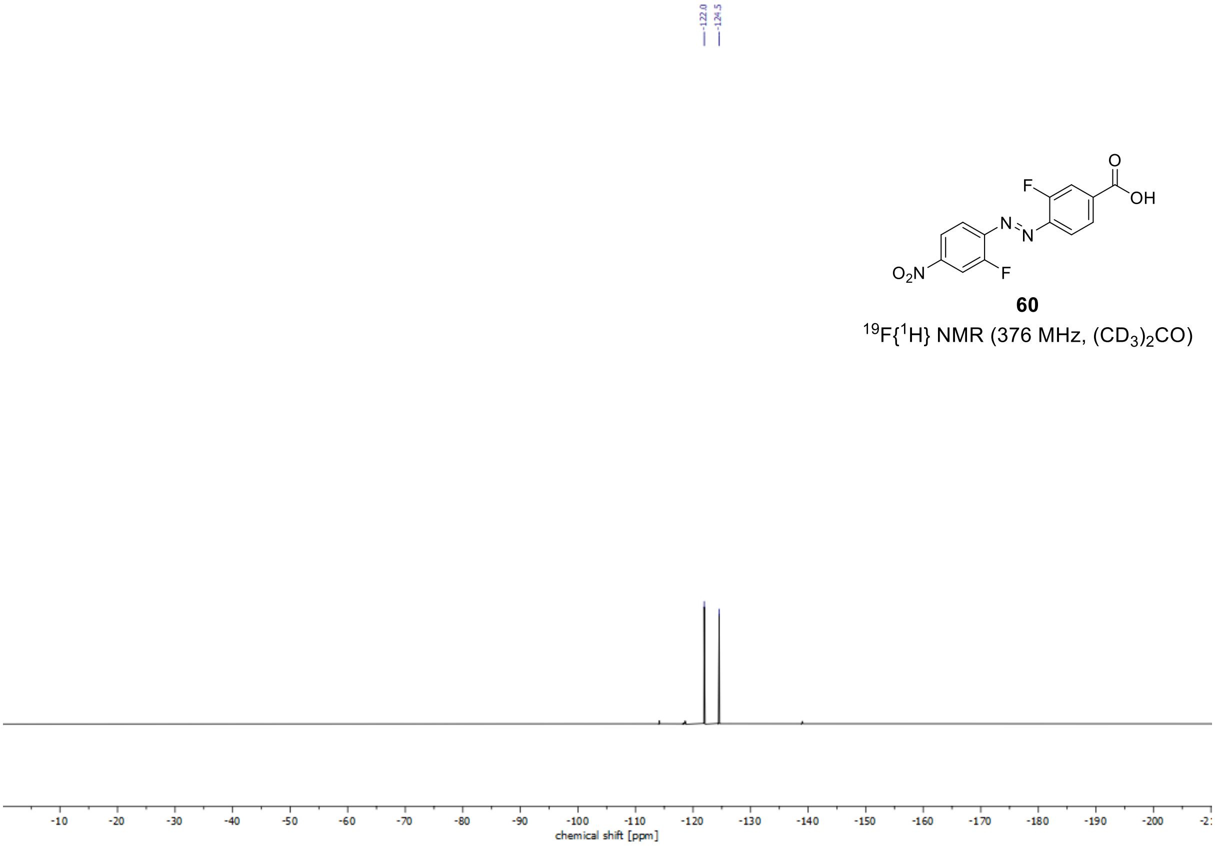


## (*E*/*Z*)-Methyl-3-fluoro-4-((2-fluorophenyl)diazenyl)benzoate (61)

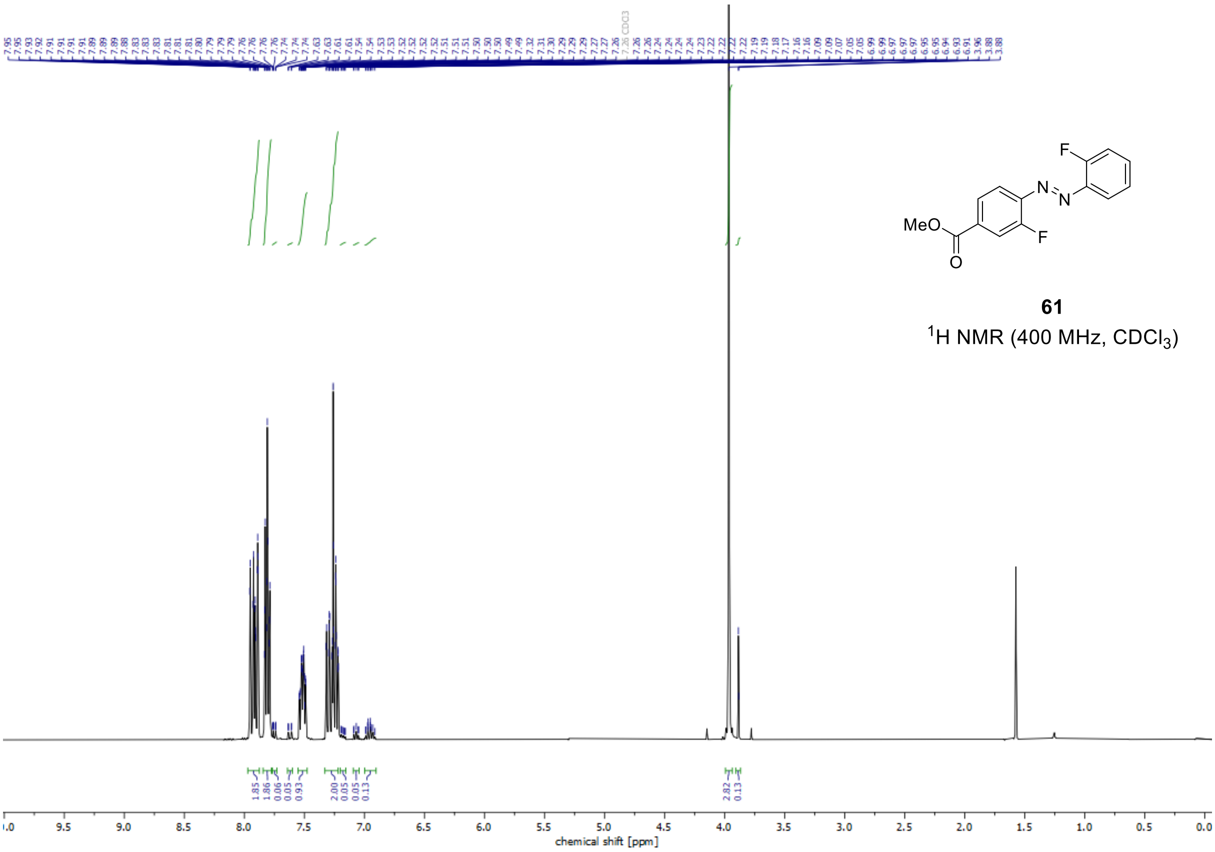

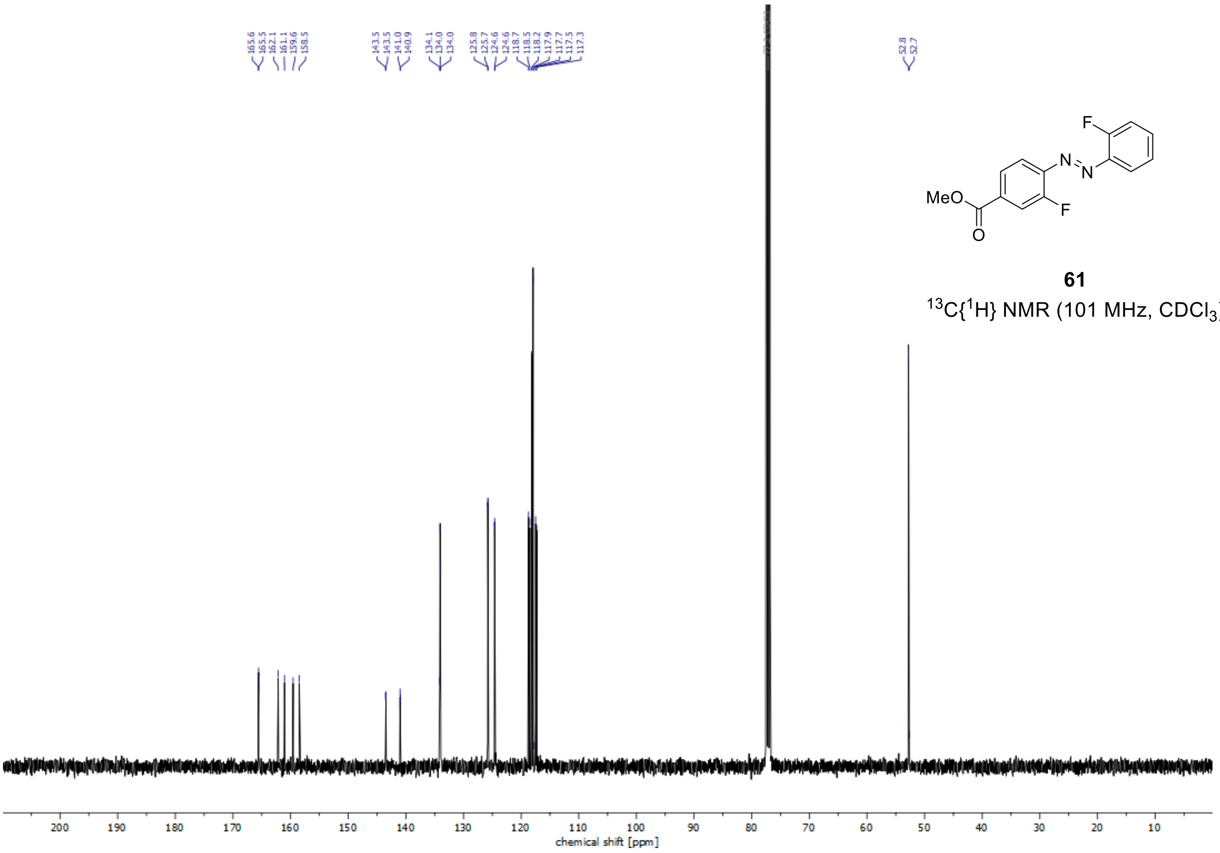


**61**

$^{13}C\{^1H\}$ NMR (101 MHz, $CDCl_3$)

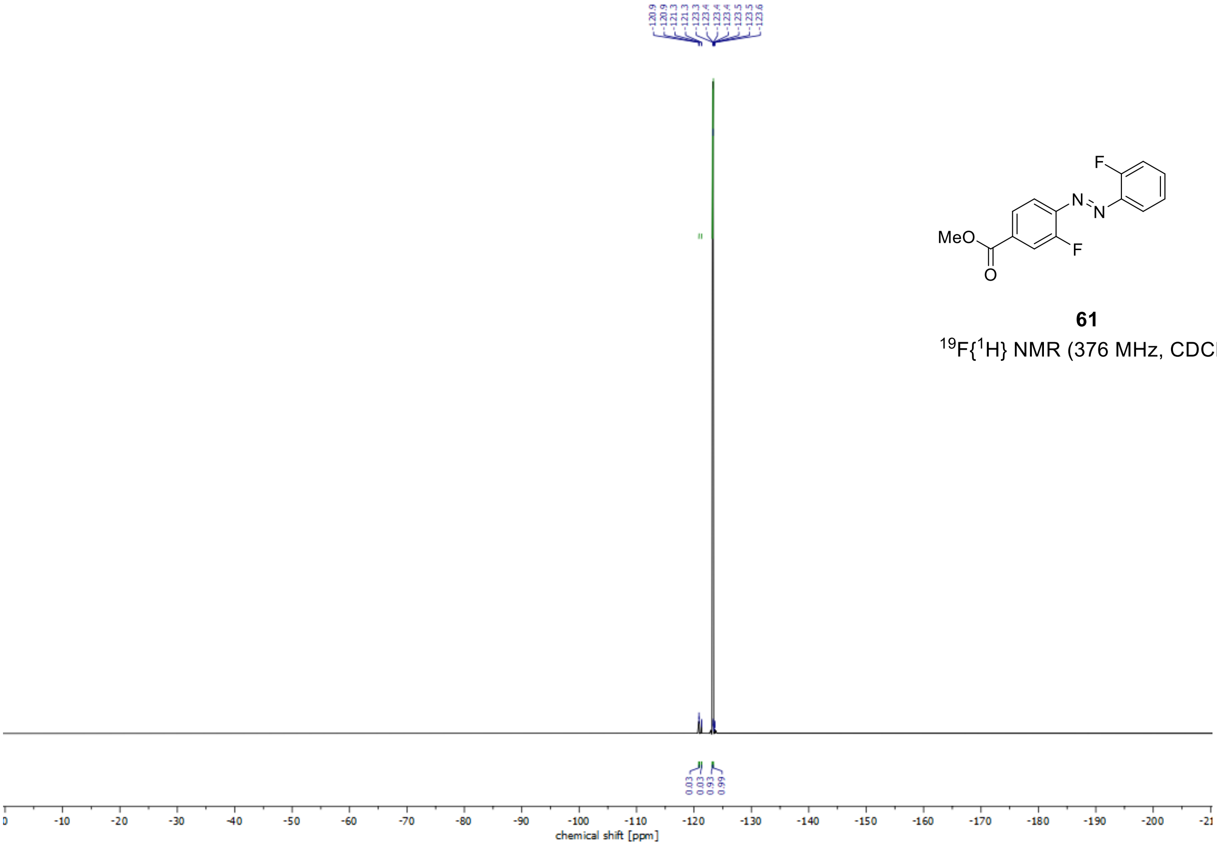


**61**

$^{19}F\{^1H\}$ NMR (376 MHz, $CDCl_3$)

**(*E*/*Z*)-3,5-Dichloro-4-((2,6-dichlorophenyl)diazenyl)benzoic acid (18)**

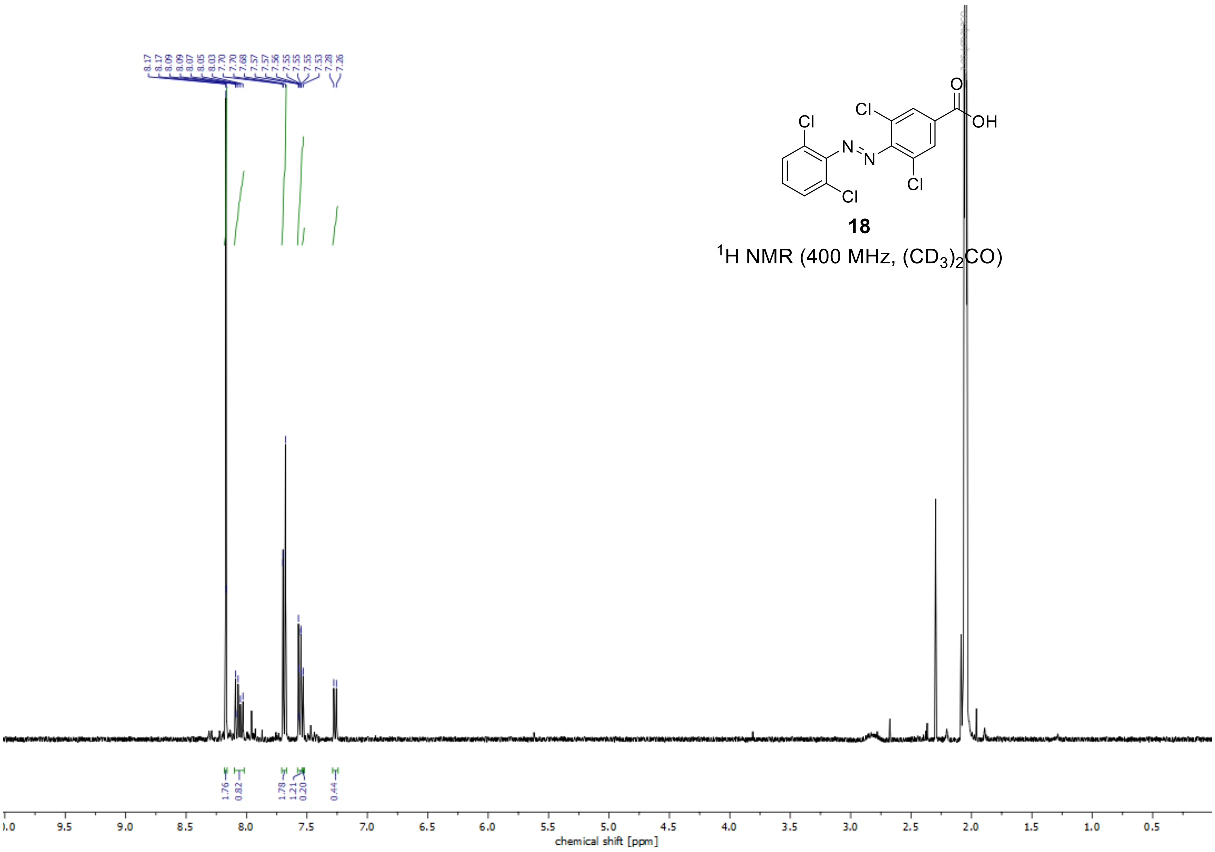


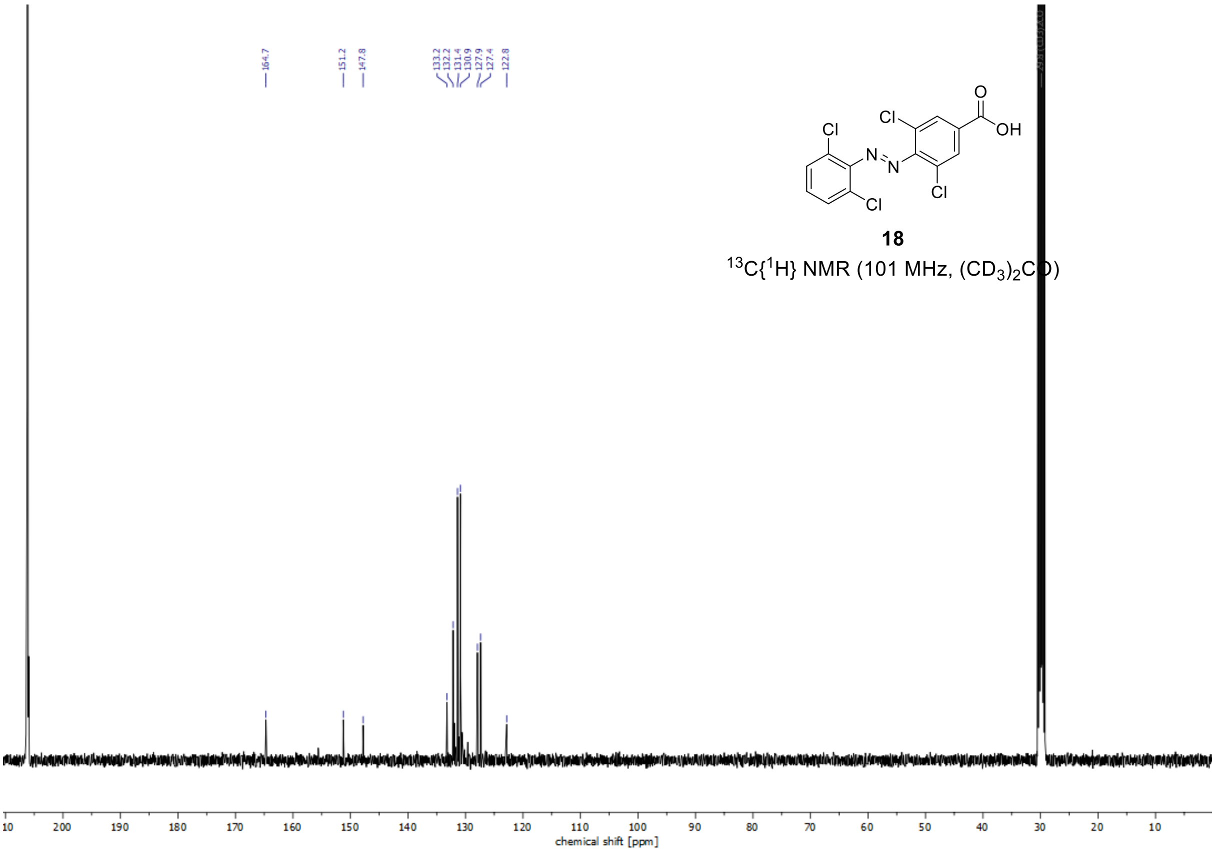

**(*E*/*Z*)-Dimethyl 4,4'-(diazene-1,2-diyl)-bis(3-fluorobenzoate) (63)**

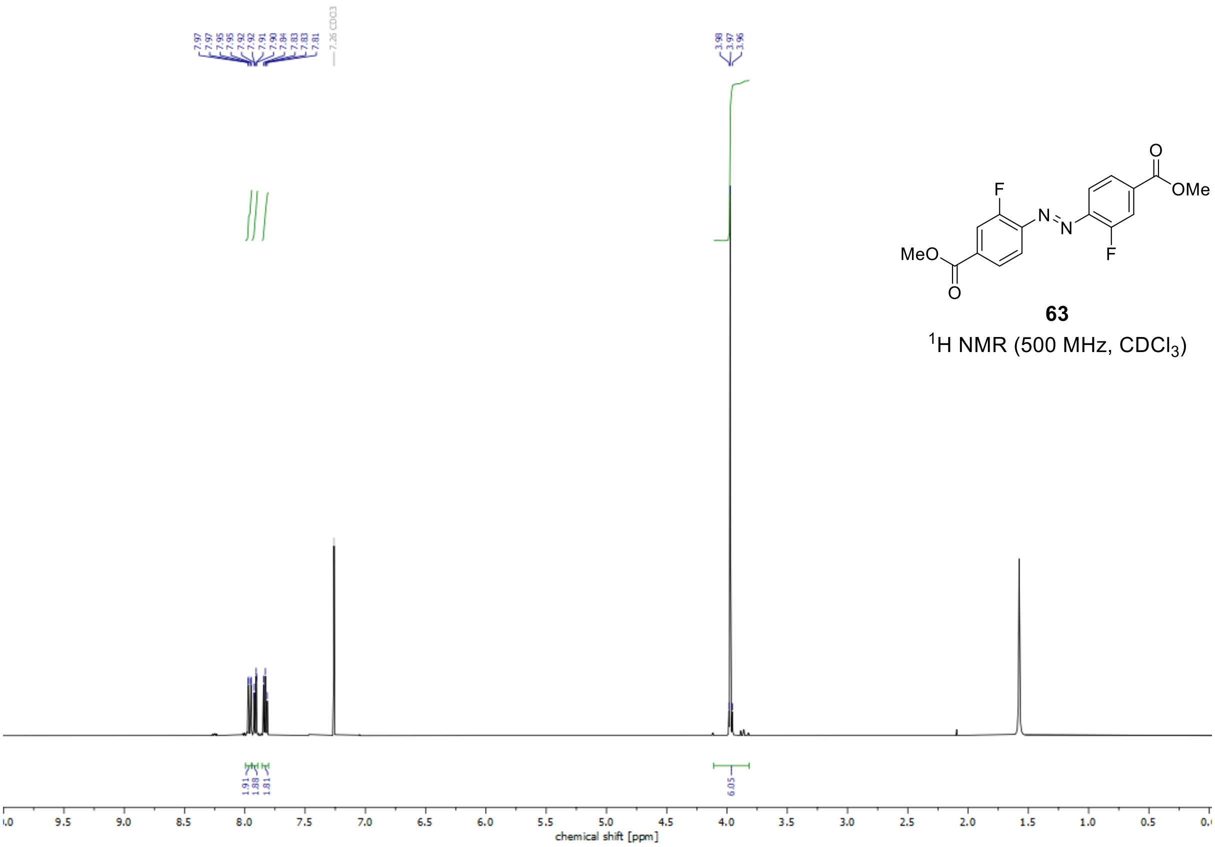


**63**

$^{1}$H NMR (500 MHz, $CDCl_3$)

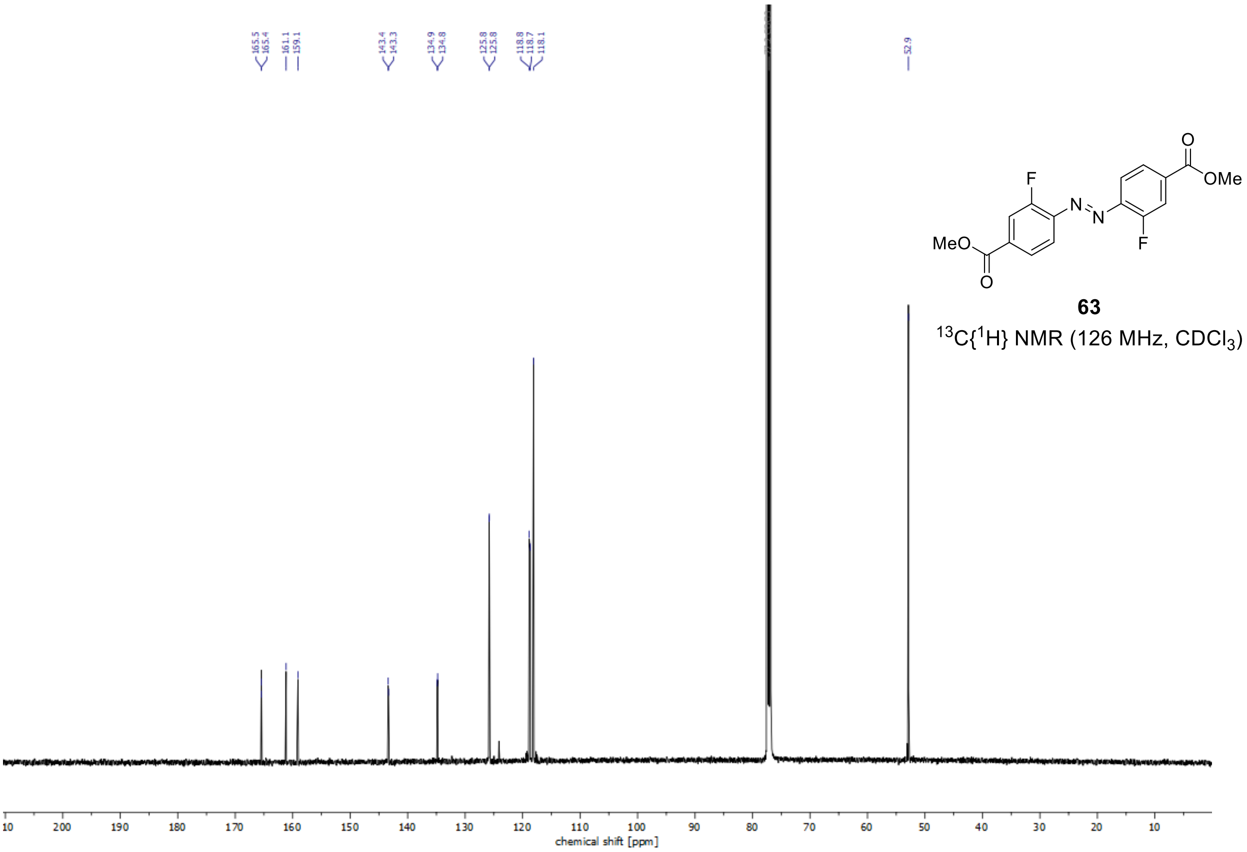


**63**

$^{13}$C{$^{1}$H} NMR (126 MHz, $CDCl_3$)

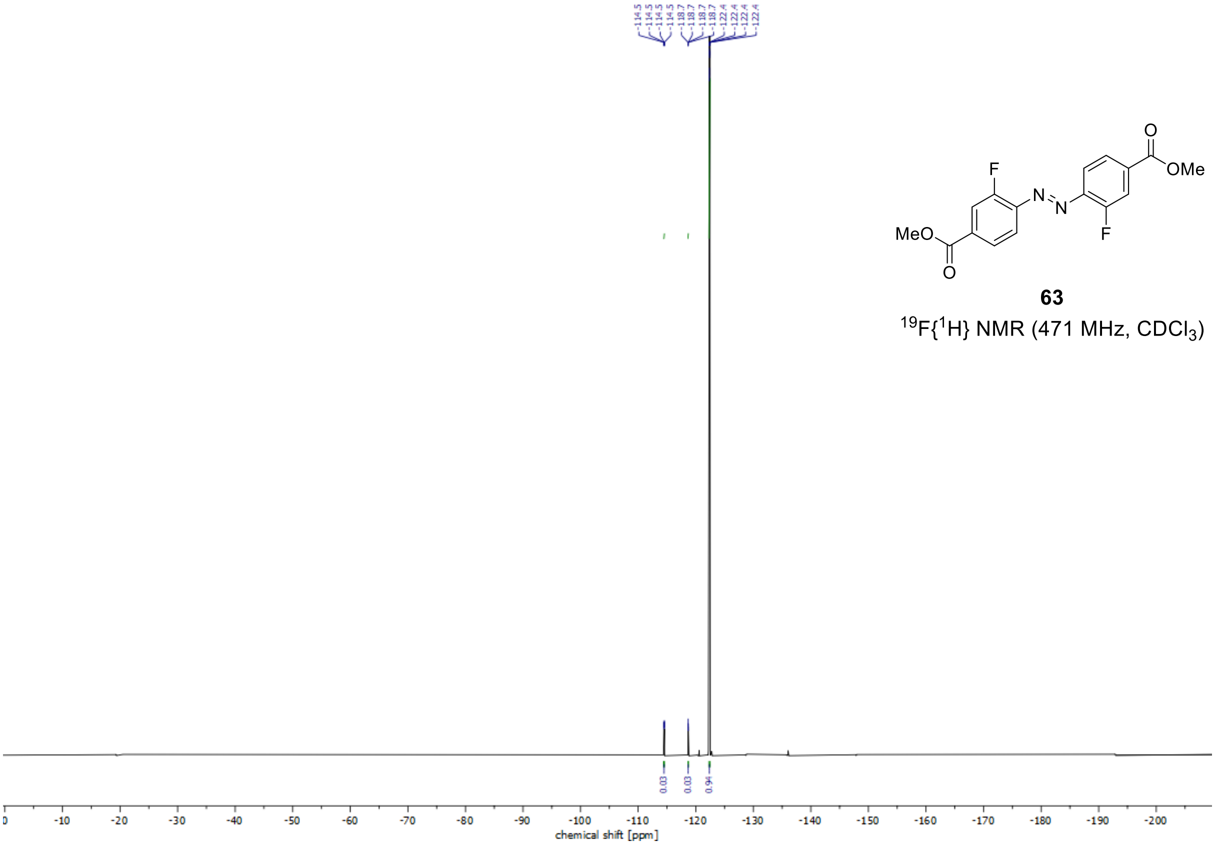


## (*E*)-3,5-Dichloro-4-((2,6-dichloro-4-nitrophenyl)diazenyl)benzenesulfonamide (64)

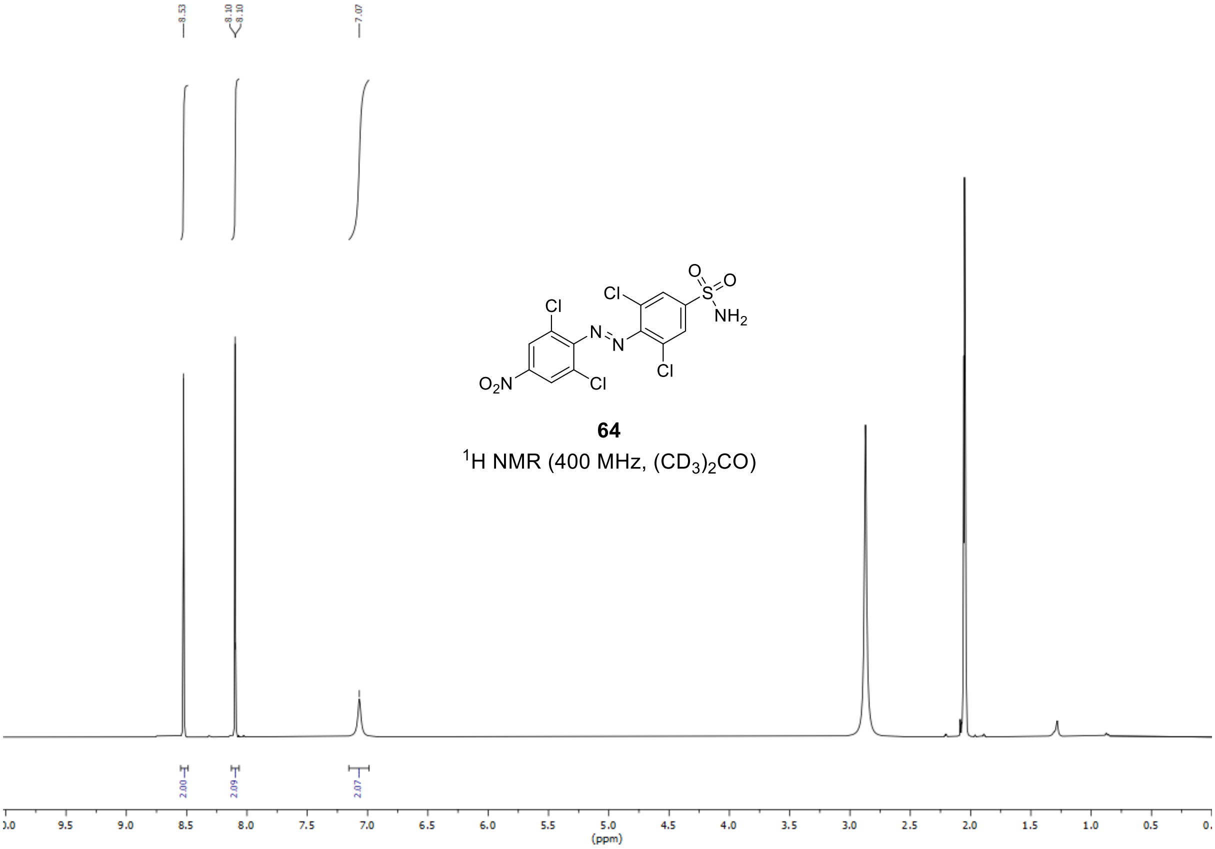

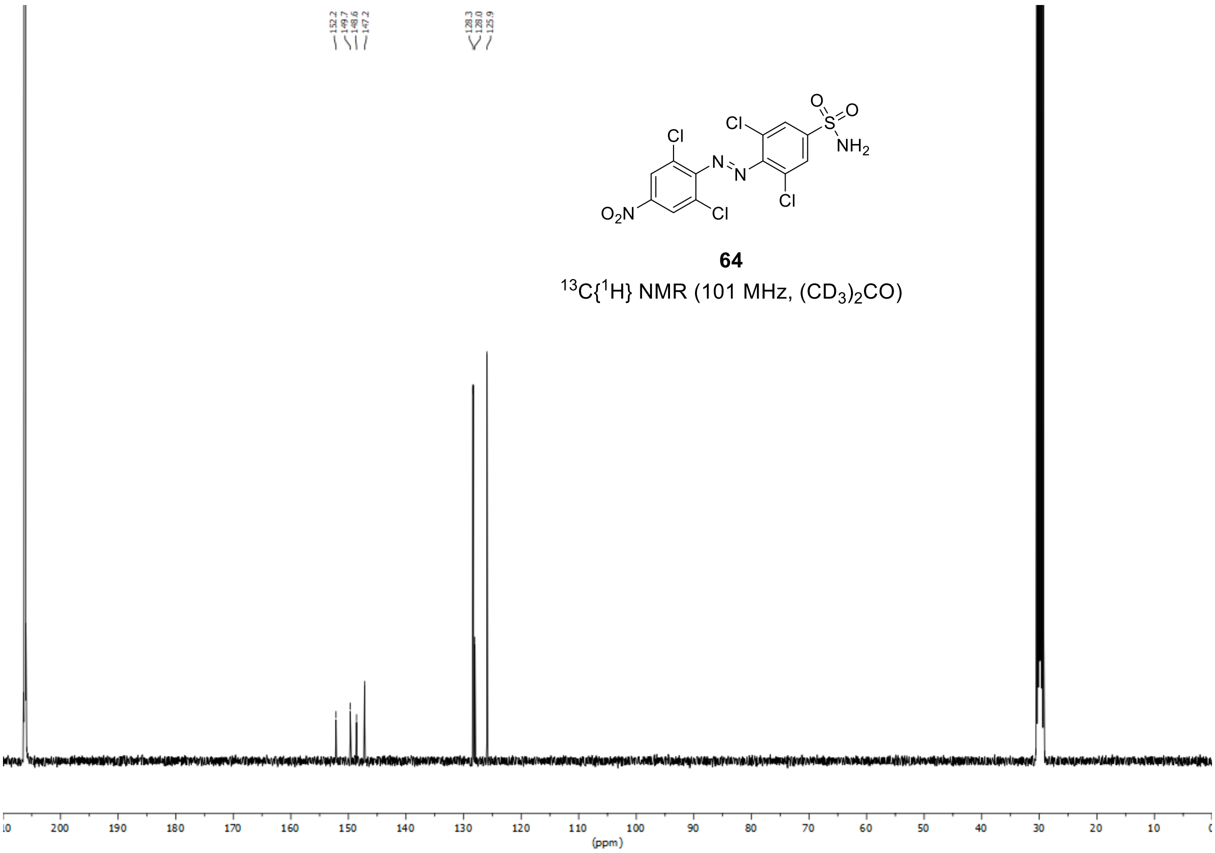


## Methyl 4-amino-3-chloro-5-fluorobenzoate (65)

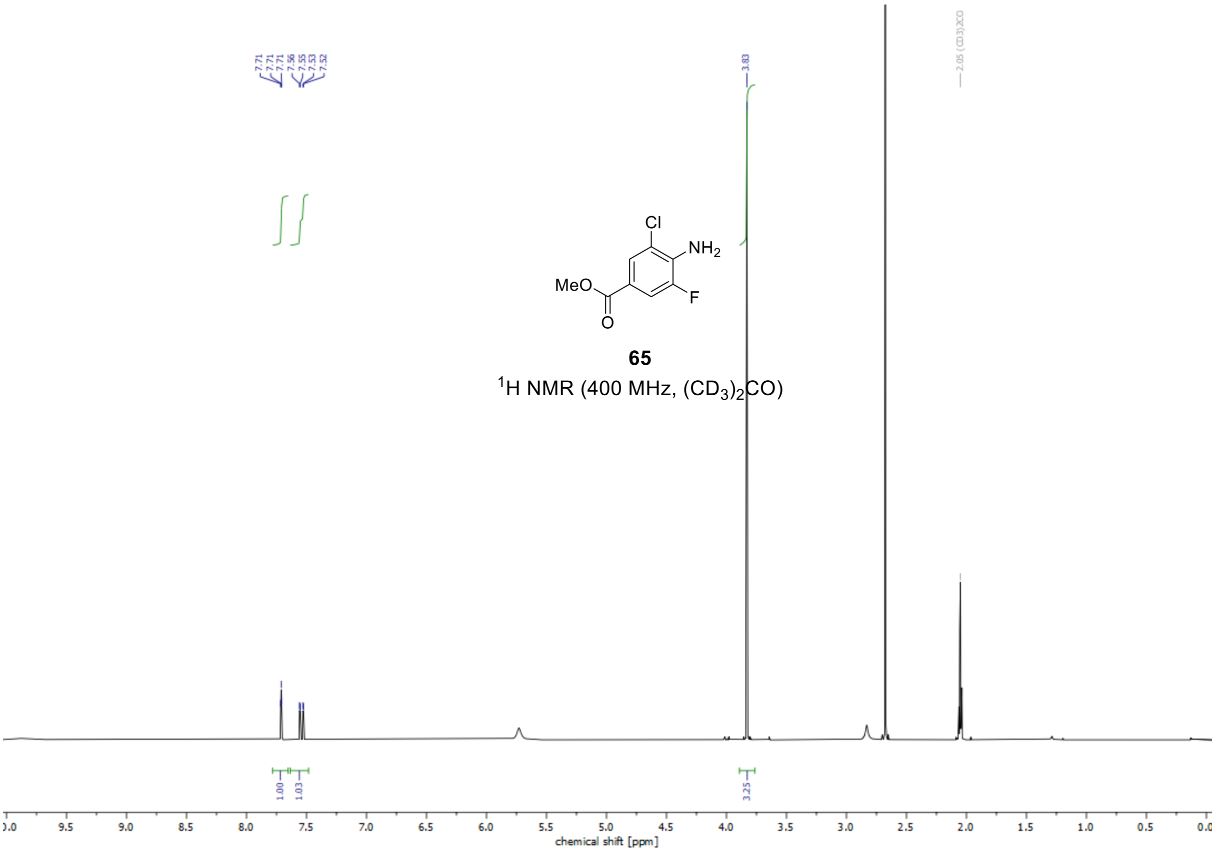

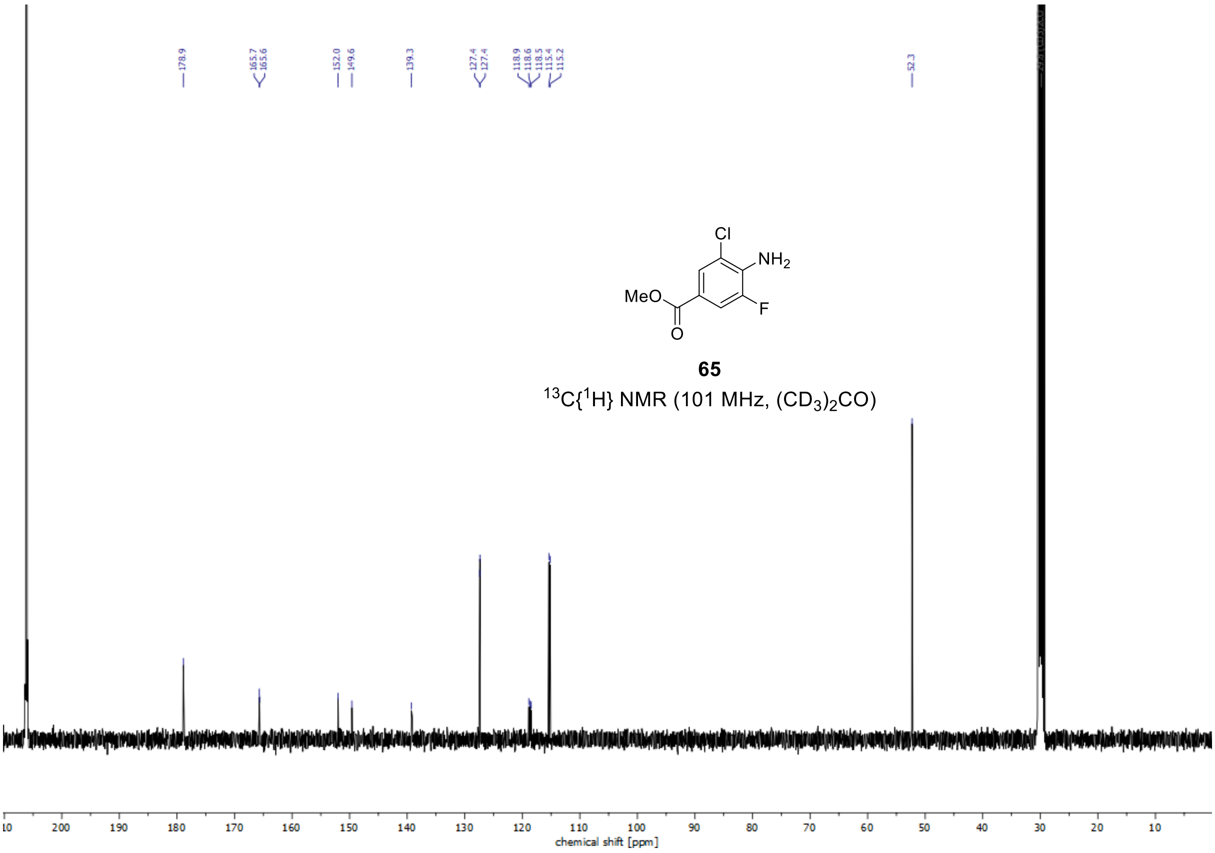
178.9
165.7
165.6
152.0
149.6
139.3
127.4
127.4
118.9
118.6
118.5
115.4
115.2
52.3
Cl
NH2
MeO
F
O
65
13C{1H} NMR (101 MHz, (CD3)2CO)
chemical shift [ppm]

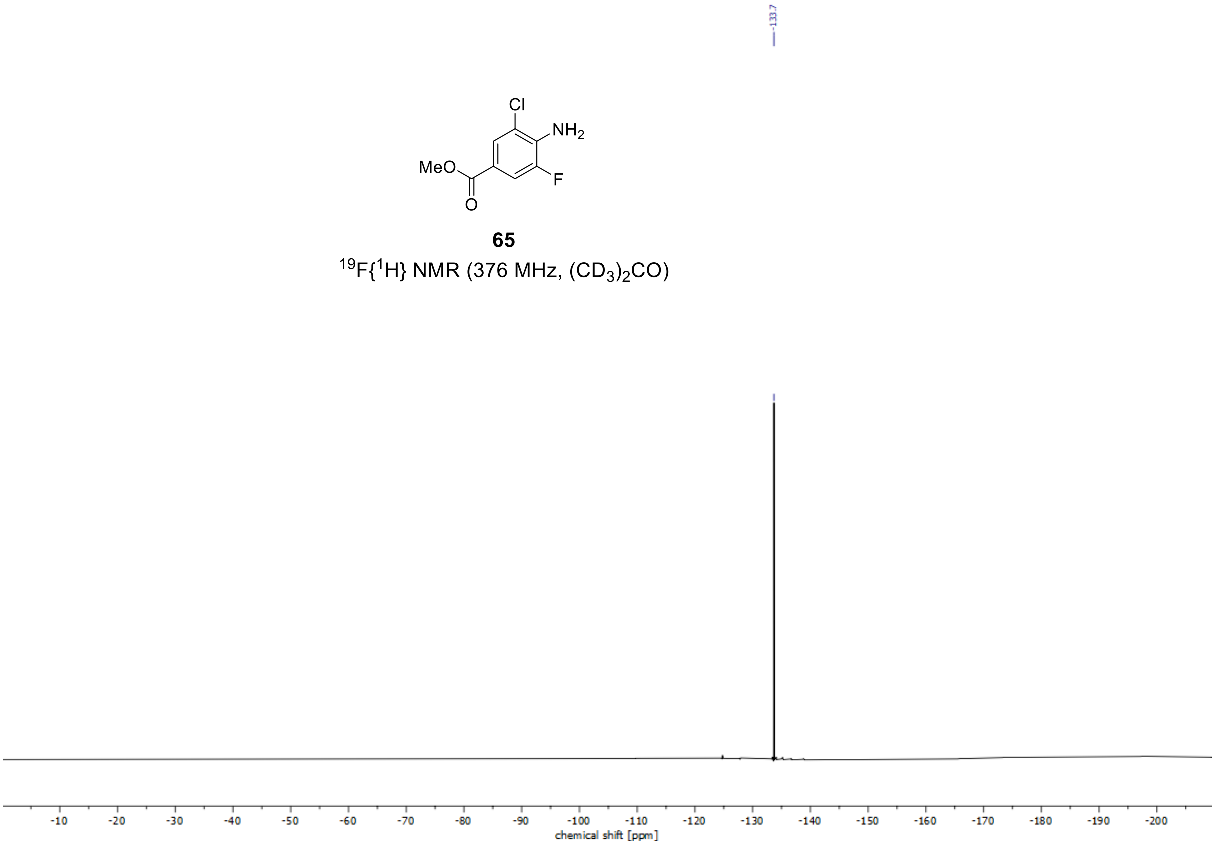
-133.7
Cl
NH2
MeO
F
O
65
19F{1H} NMR (376 MHz, (CD3)2CO)
chemical shift [ppm]

## Literature References


[1] *Bruker Instrument Service v3.0.21*.

[2] *SAINT V8.18C*, **2011**.

[3] *APEX2 V2012.4-3*.

[4] *SHELXS-97: Program for Crystal Structure Solution*, **1997**.

[5] *SHELXL-97: Program for the Refinement of Crystal Structures*, **1997**.

[6] *Platon: A Multipurpose Crystallographic Tool*, **1999**.

[7] D. Bléger, J. Schwarz, A. M. Brouwer, S. Hecht, *J. Am. Chem. Soc.* **2012**, *134*, 20597–20600.

[8] D. B. Konrad, G. Savasci, L. Allmendinger, D. Trauner, C. Ochsenfeld, A. M. Ali, *J. Am. Chem. Soc.* **2020**, *142*, 6538–6547.

[9] M. J. Hansen, M. M. Lerch, W. Szymanski, B. L. Feringa, *Angew. Chem. Int. Ed.* **2016**, *55*, 13514–13518.

[10] C. Knie, M. Utecht, F. Zhao, H. Kulla, S. Kovalenko, A. M. Brouwer, P. Saalfrank, S. Hecht, D. Bléger, *Chem. – Eur. J.* **2014**, *20*, 16492–16501.

[11] R. A. Fujimoto, L. W. Mcquire, L. G. Monovich, B. B. Mugrage, D. T. Parker, J. H. V. Duzer, S. Wattanasin, *Substituted Amino Phenylacetic Acids, Derivatives Thereof, Their Preparation and Their Use as Cyclooxygenase 2 (Cox-2) Inhibitors*, **2004**, WO2004048314A1.

[12] L. S. Runtsch, D. M. Barber, P. Mayer, M. Groll, D. Trauner, J. Broichhagen, *Beilstein J. Org. Chem.* **2015**, *11*, 1129–1135.
