# Supplementary material for: A Platform for the Development of Highly Red‐Shifted Azobenzene‐Based Optical Tools
Source: Angew Chem Int Ed Engl. 2025 Jun 23;64(32):e202501779. doi: 10.1002/anie.202501779 (PMC12322654; doi:10.1002/anie.202501779)
Supplement: Supplementary file 2 — Supporting Information [file ANIE-64-e202501779-s003.pdf]

A platform for the development of Highly Red-Shifted Azobenzene-based Optical Tools

Kyra Lützel[#], Henryk Laqua[#], Manjima B. Sathian[#], Benedikt Nißl[#], Judit Katalin Szántó, Christina-Anna Senser, Gökcen Savasci, Lars Allmendinger, Bilal Kicin, Vincent Ruf, Dominik Kammerer, Theobald Lohmüller, Konstantin Karaghiosoff, Ahmed M. Ali, Ursula Storch*, Michael Mederos y Schnitzler*, Christian Ochsenfeld* and David B. Konrad*

[#] these authors contributed equally to this work

[*] corresponding authors

Supporting Information II:
Photophysical Characterization

Table of Content

1. NMR Data – Photostationary State (PSS) Analysis 3
2. UV-vis Data – Photostationary State (PSS) Analysis 100
3. UV-vis Data - Reversible Photoswitching 121
4. UV-vis Data – Stability against GSH 132
5. Thermal Relaxation 158
6. Molecular Absorption Coefficient 182
7. Quantum Yield 183
8. X-Ray Data 188

1. NMR Data – Photostationary State (PSS) Analysis

To determine the photostationary states (PSS), the dark adapted azobenzenes were first irradiated with 740 nm, 650 nm, 525 nm, 450-455 nm and 365 nm and then measured at a 500 MHz Avance III HD Bruker BioSpin equipped with a CryoProbe™ Prodigy (^{19}F NMR without ^{1}H decoupling) until the final PSS is reached. The ratio of the (*E*)- and (*Z*)-isomers to each other results from the integral ratio in the ^{1}H or ^{19}F NMR. In case of the minor species being 0% it means as far as detectable by the applied NMR method. Due to short half-lives, the determination *via* NMR could not be carried out for all compounds.

(*E*/*Z*)-Azobenzene (1, 500 µM, DMSO-*d*$_6$//D$_2$O 9:1)

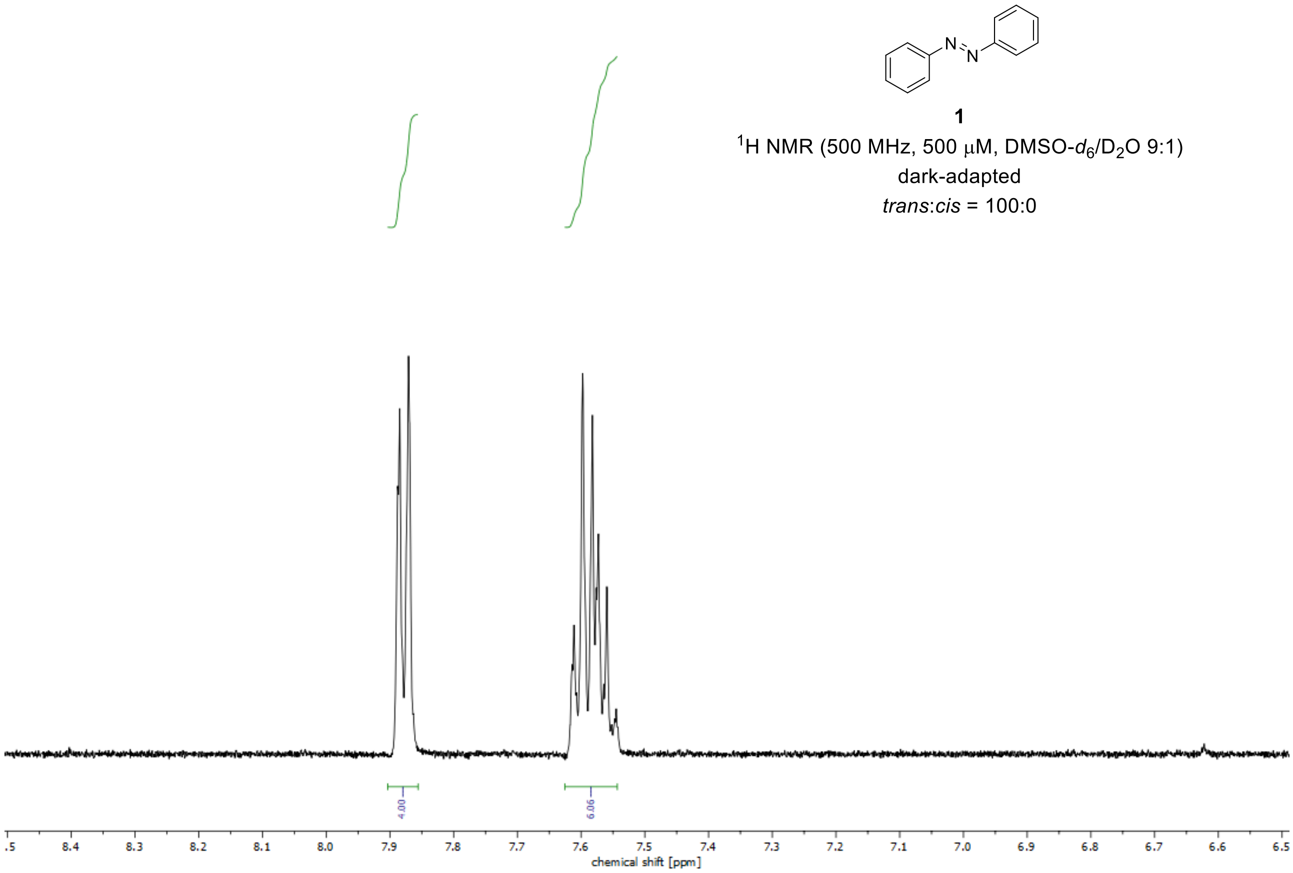

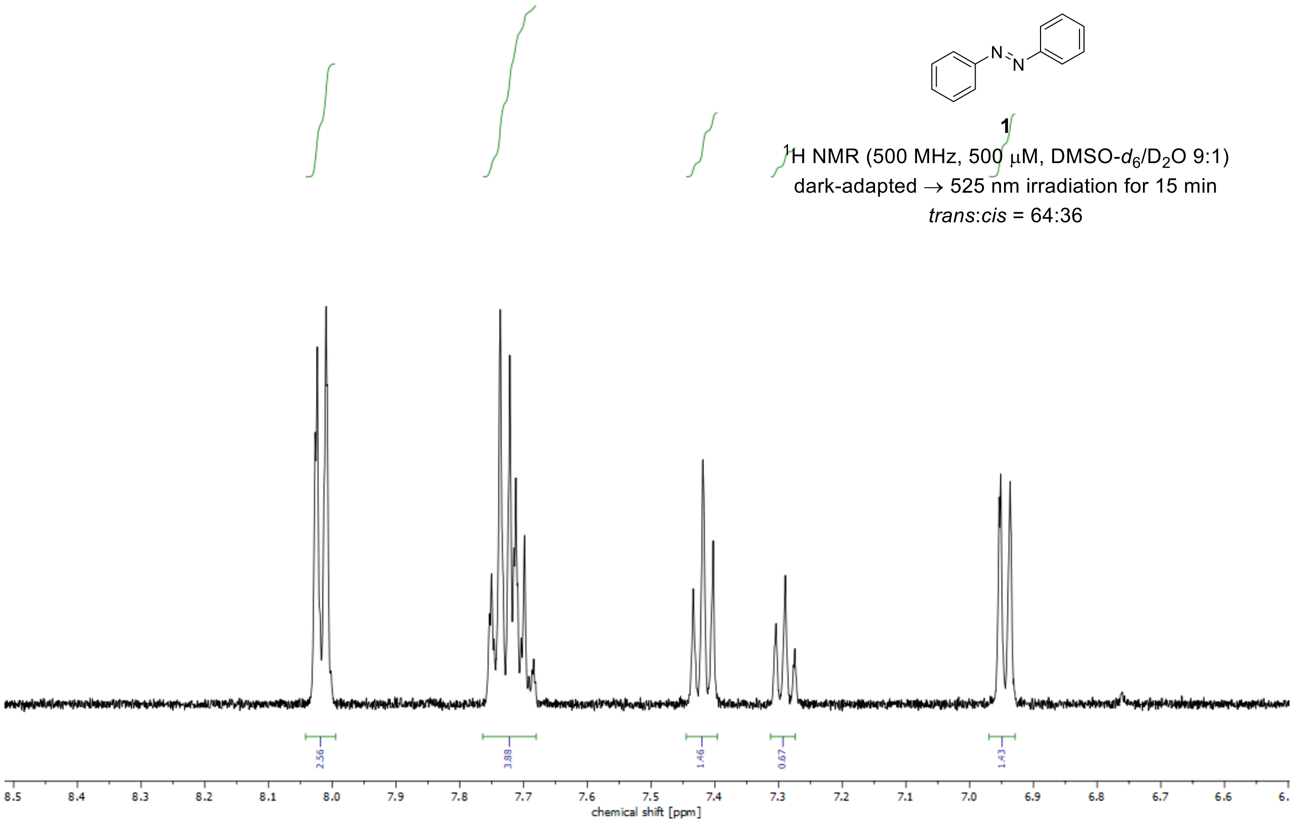

1

^{1}H NMR (500 MHz, 500 μM, DMSO-d_6/D_2O 9:1)

dark-adapted → 525 nm irradiation for 15 min

trans:*cis* = 64:36

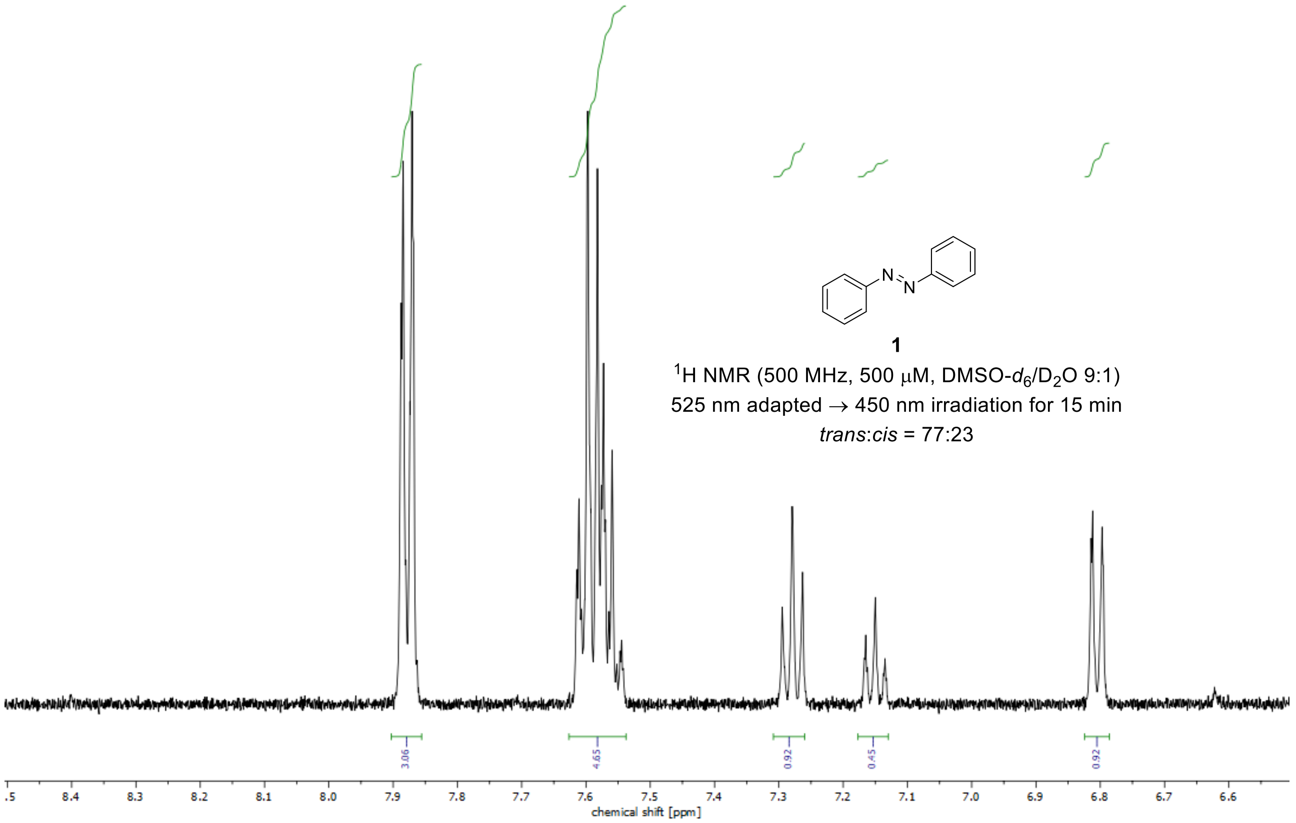

1

^{1}H NMR (500 MHz, 500 μM, DMSO-d_6/D_2O 9:1)

525 nm adapted → 450 nm irradiation for 15 min

trans:*cis* = 77:23

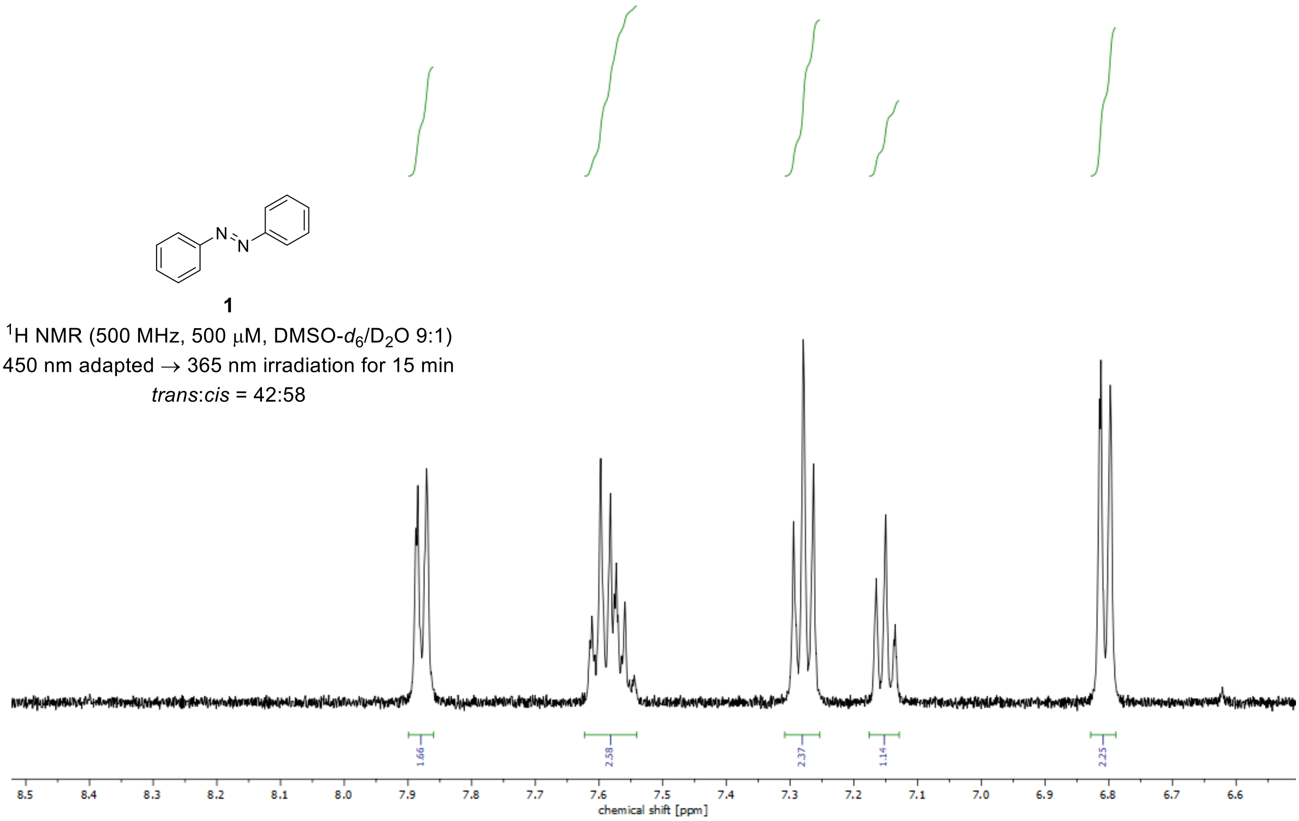

(*E*/*Z*)-1,2-Bis(2,6-difluorophenyl)diazene (2, 500 µM, DMSO-d_6/D_2O 9:1)

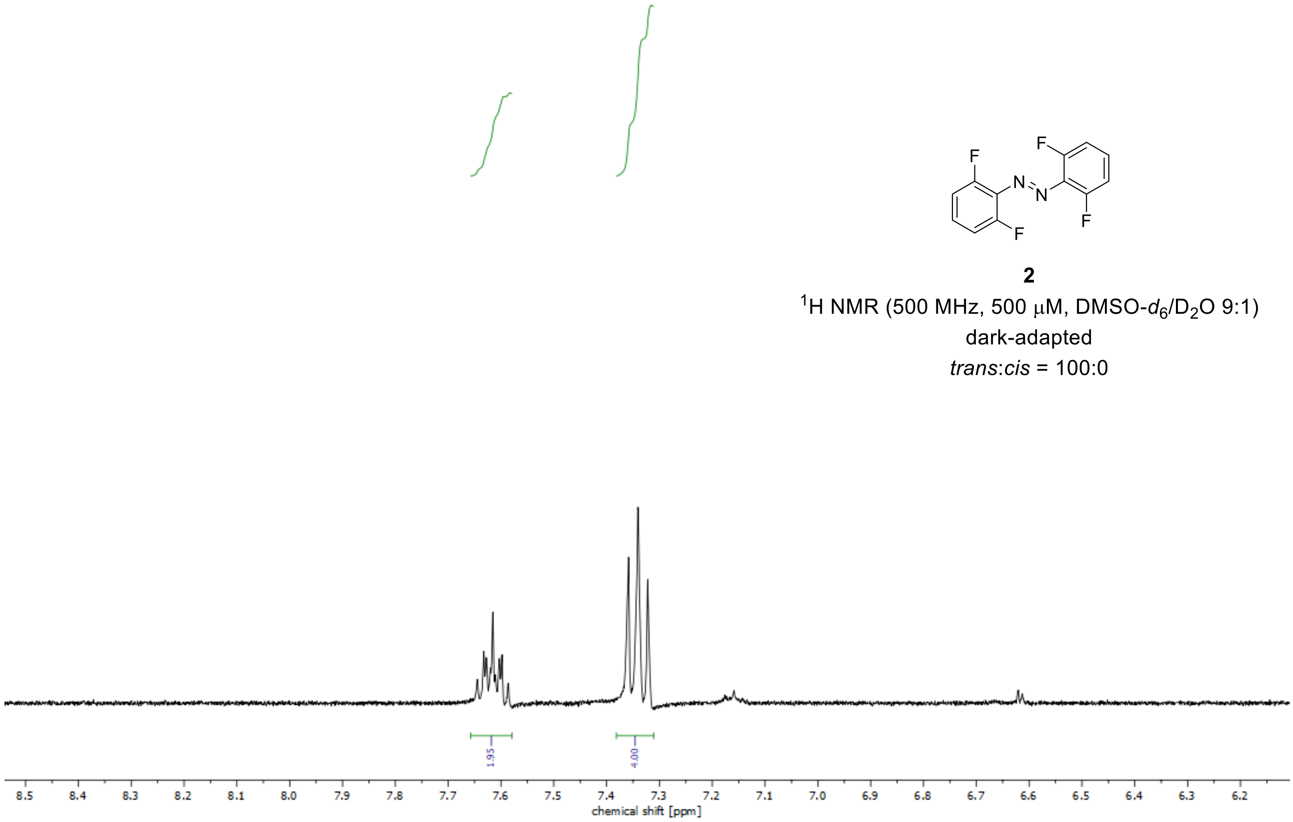

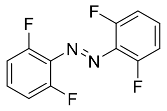

2

^{19}F NMR (471 MHz, 500 μM, DMSO-d_6/D_2O 9:1)

dark-adapted

trans:*cis* = 100:0

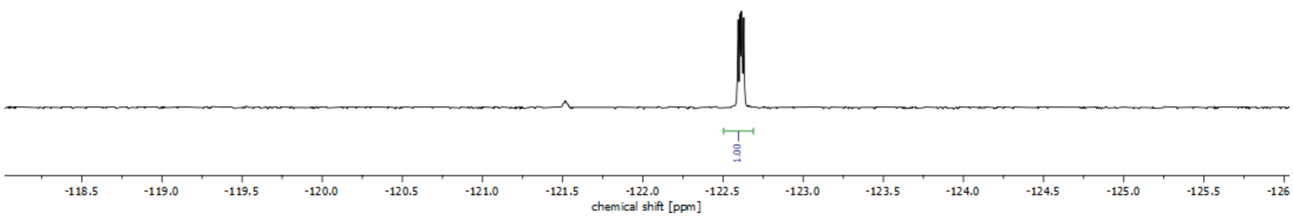

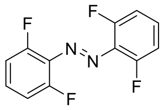

2

^{19}F NMR (471 MHz, 500 μM, DMSO-d_6/D_2O 9:1)

dark-adapted → 525 nm irradiation for 15 min

trans:*cis* = 8:92

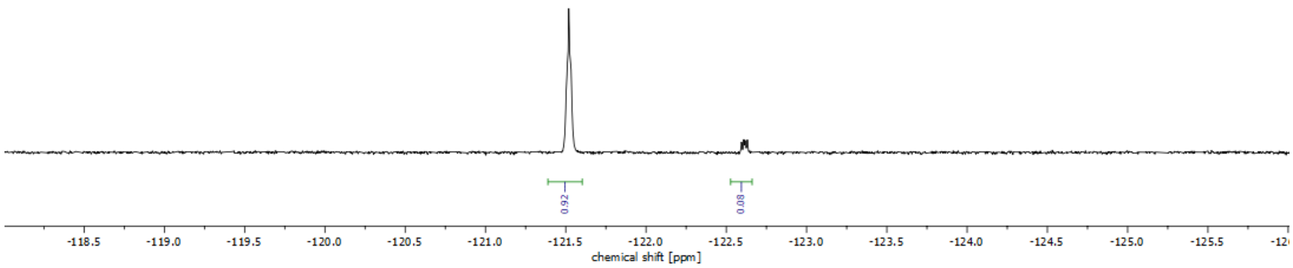

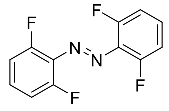

2

^{19}F NMR (471 MHz, 500 μM, DMSO-d_6/D_2O 9:1)
525nm-adapted → 450 nm irradiation for 15 min
trans:*cis* = 58:42

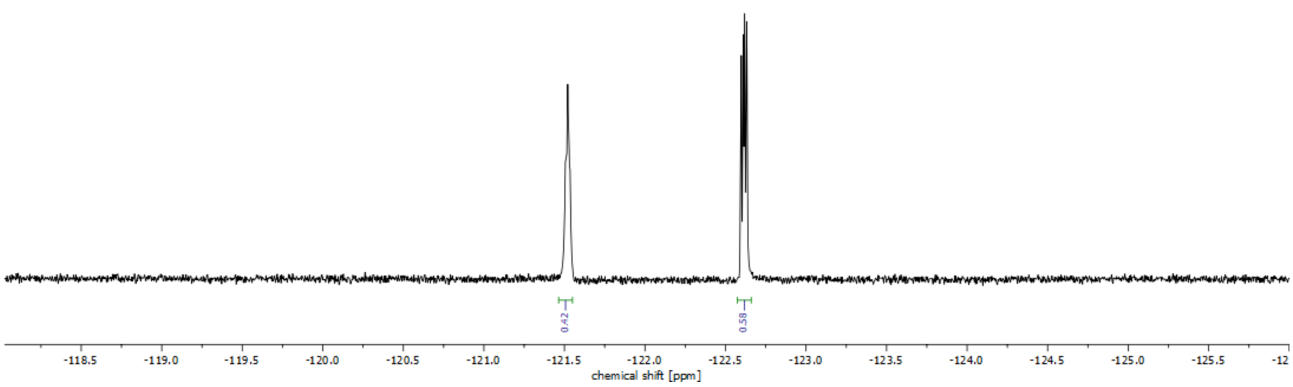

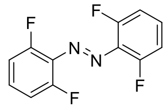

2

^{19}F NMR (471 MHz, 500 μM, DMSO-d_6/D_2O 9:1)
450nm-adapted → 365 nm irradiation for 15 min
trans:*cis* = 84:16

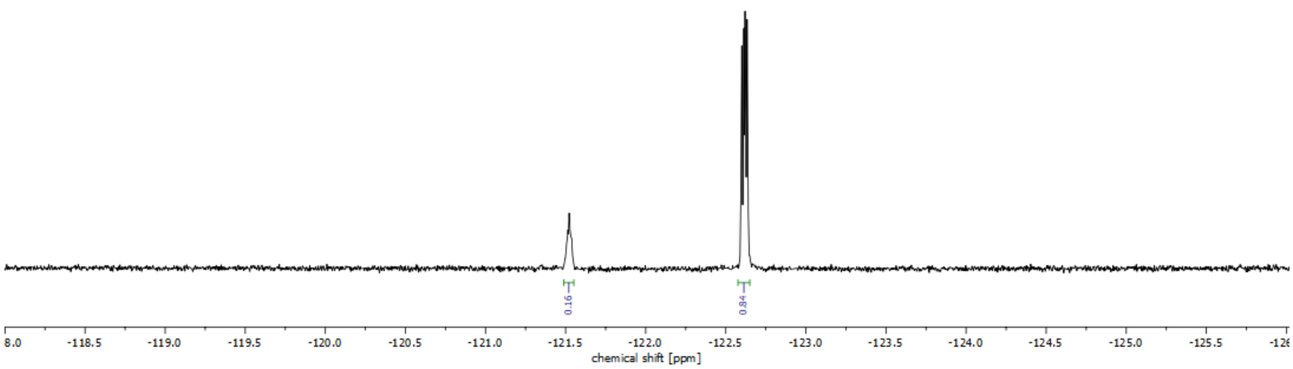

(*E*/*Z*)-1,2-Bis(2-chloro-6-fluorophenyl)diazene (3, 500 µM, DMSO-d_6/D_2O 9:1)

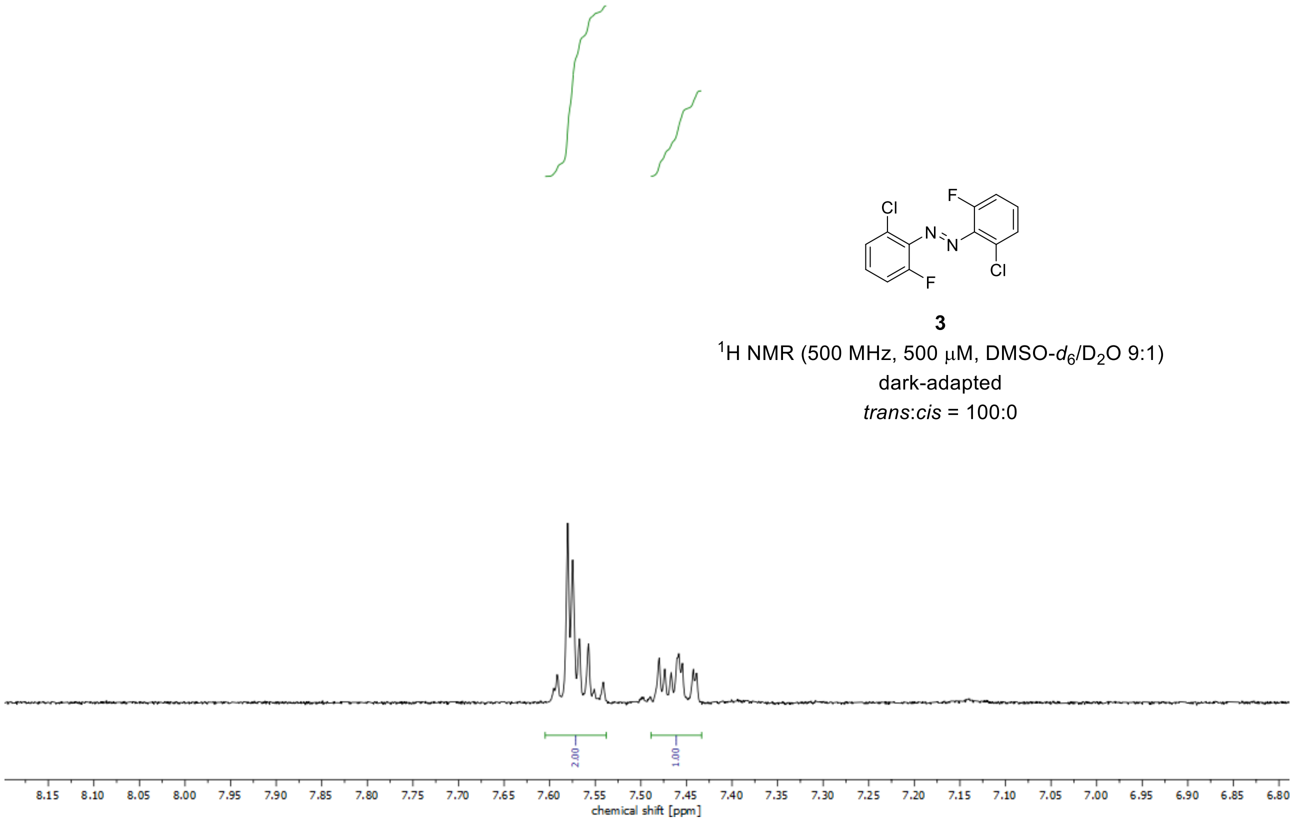

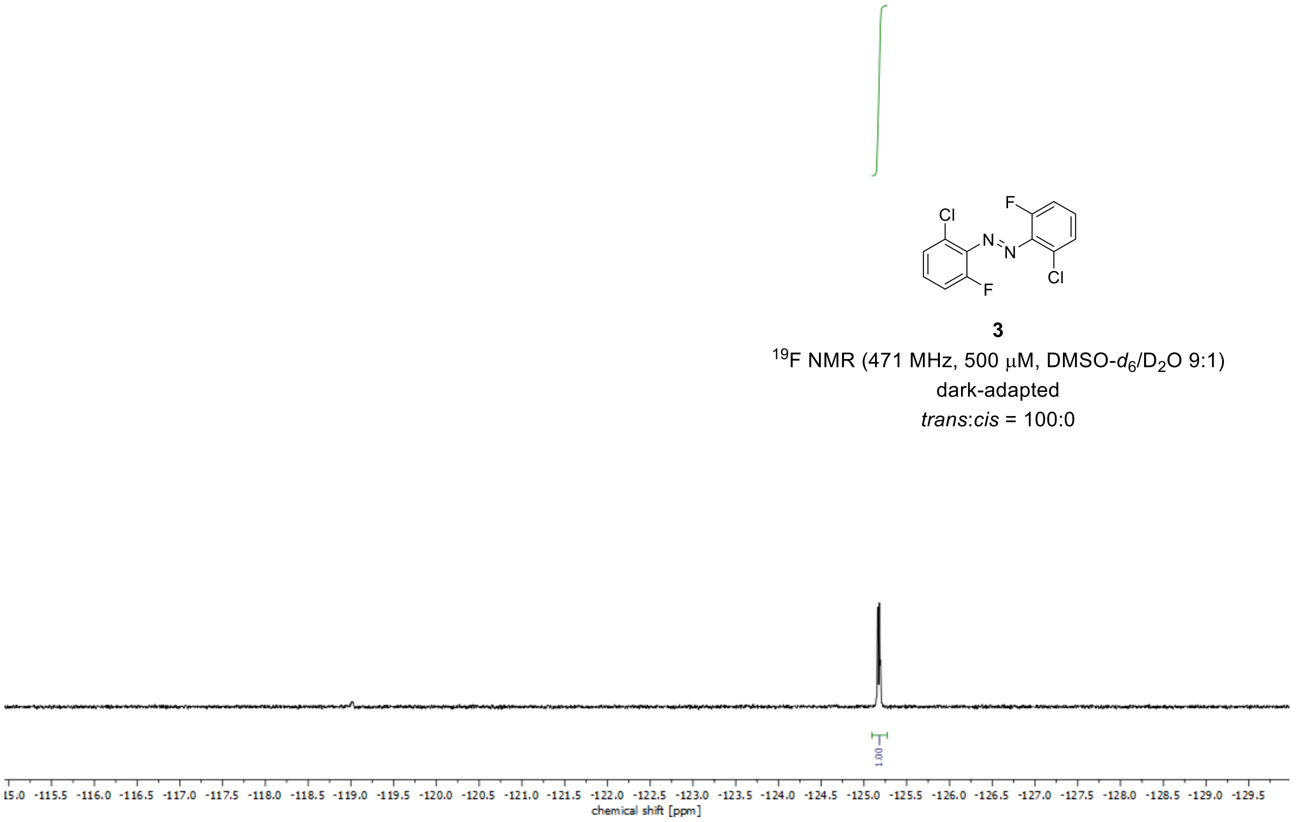

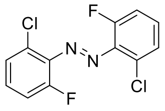

3

^{19}F NMR (471 MHz, 500 µM, DMSO-d_6/D_2O 9:1)
dark-adapted → 650 nm irradiation for 15 min
trans:*cis* = 63:37

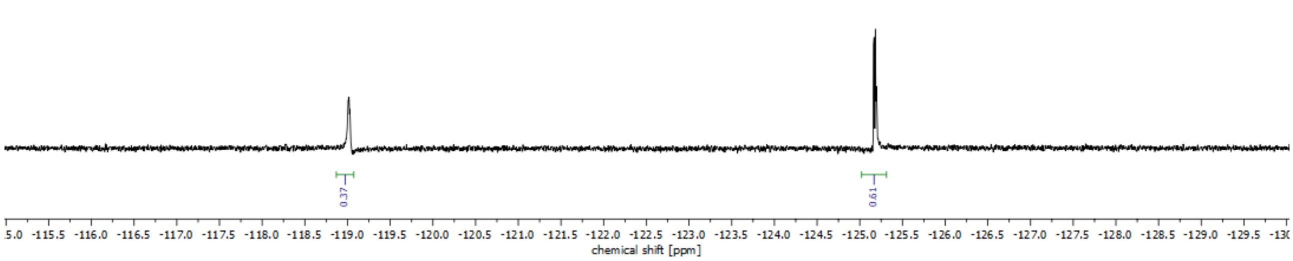

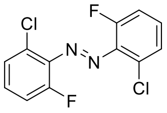

3

^{19}F NMR (471 MHz, 500 µM, DMSO-d_6/D_2O 9:1)
dark-adapted → 650 nm irradiation for 30 min
trans:*cis* = 43:57

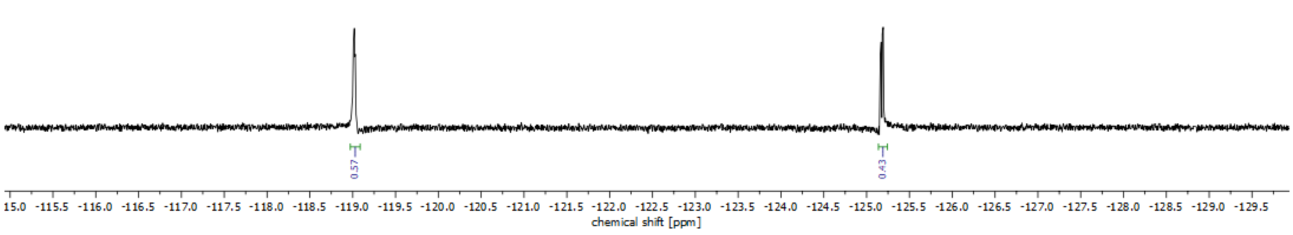

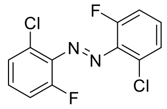

3

^{19}F NMR (471 MHz, 500 μM, DMSO-d_6/D_2O 9:1)
dark-adapted → 650 nm irradiation for 45 min
trans:*cis* = 29:71

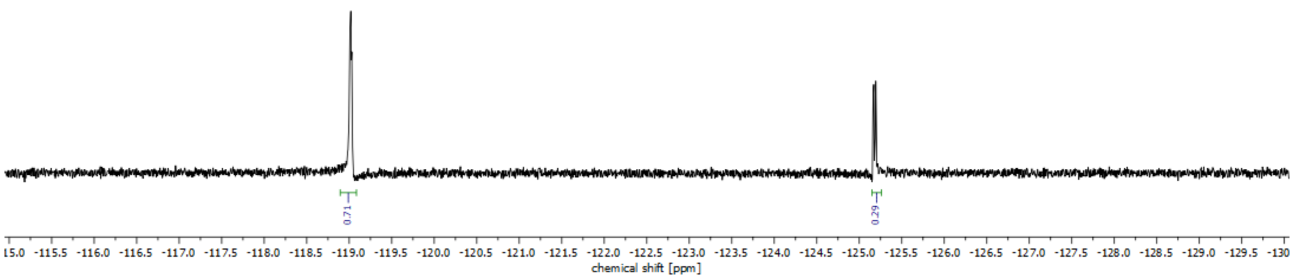

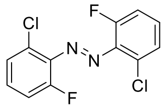

3

^{19}F NMR (471 MHz, 500 μM, DMSO-d_6/D_2O 9:1)
dark-adapted → 650 nm irradiation for 60 min
trans:*cis* = 22:78

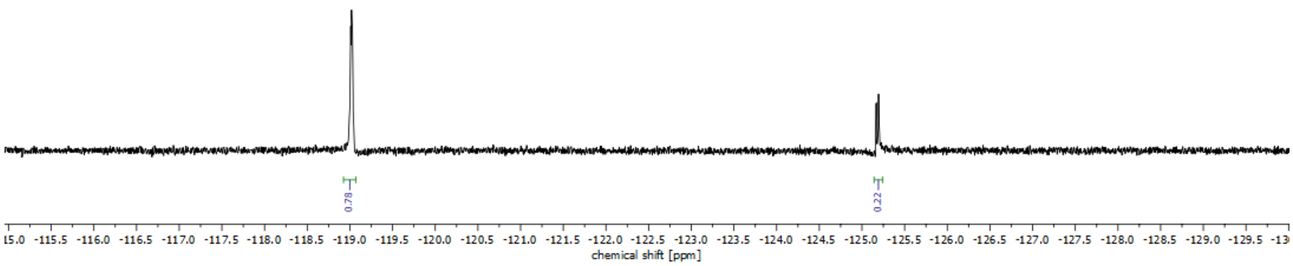

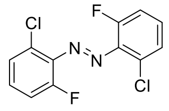

3

^{19}F NMR (471 MHz, 500 μM, DMSO-d_6/D_2O 9:1)
dark-adapted → 650 nm irradiation for 90 min
trans:*cis* = 12:88

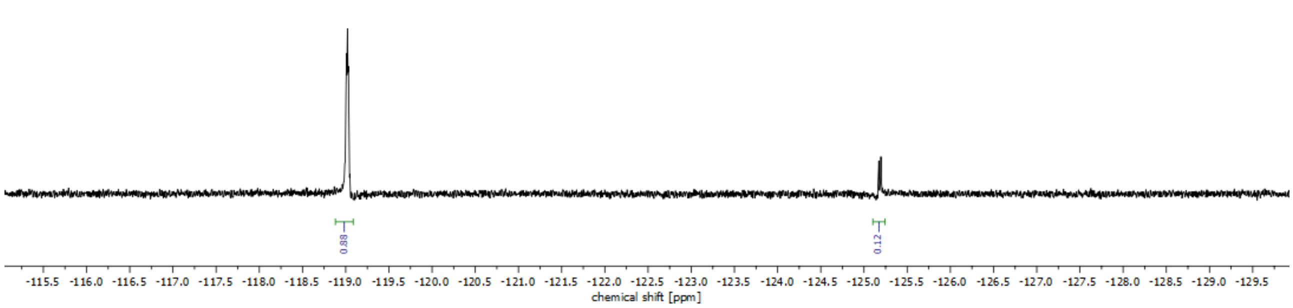

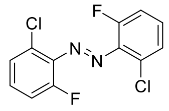

3

^{19}F NMR (471 MHz, 500 μM, DMSO-d_6/D_2O 9:1)
dark-adapted → 650 nm irradiation for 120 min
trans:*cis* = 8:92

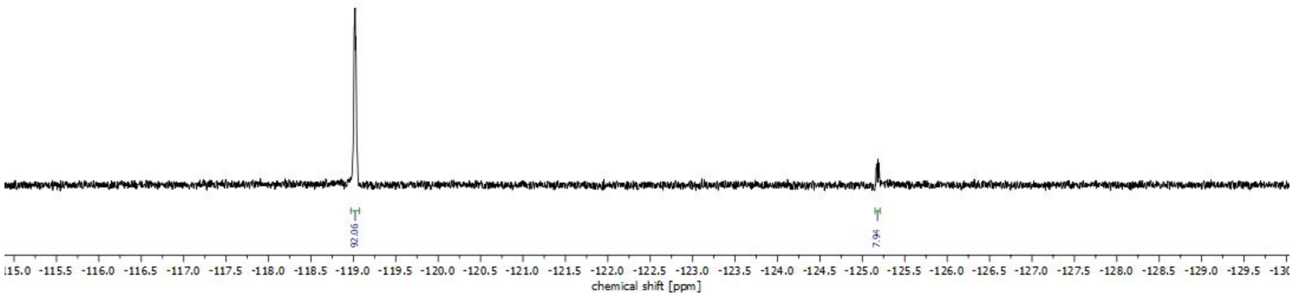

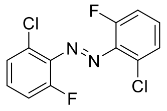

3

^{19}F NMR (471 MHz, 500 μM, DMSO-d_6/D_2O 9:1)
650nm-adapted → 525 nm irradiation for 15 min
trans:*cis* = 17:83

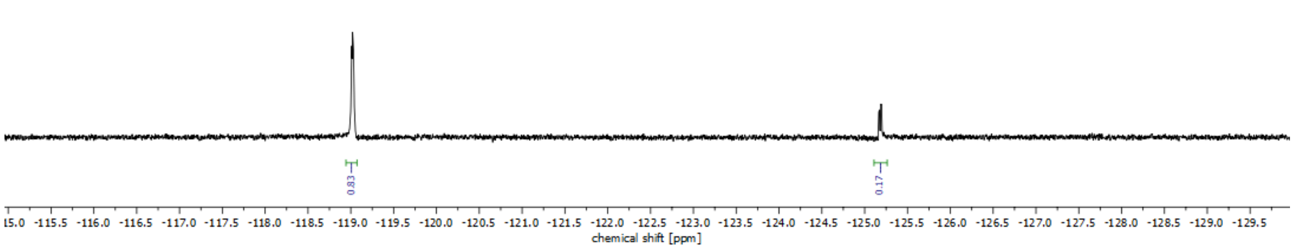

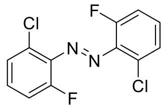

3

^{19}F NMR (471 MHz, 500 μM, DMSO-d_6/D_2O 9:1)
525 nm-adapted → 450 nm irradiation for 15 min
trans:*cis* = 74:26

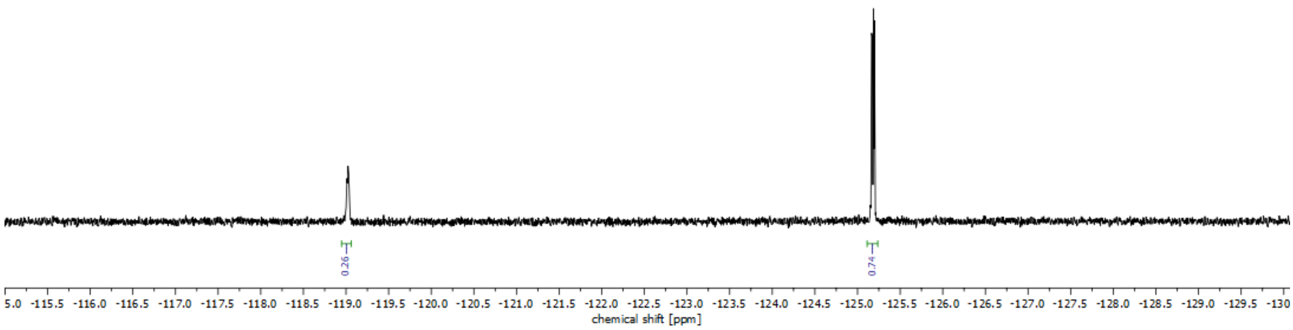

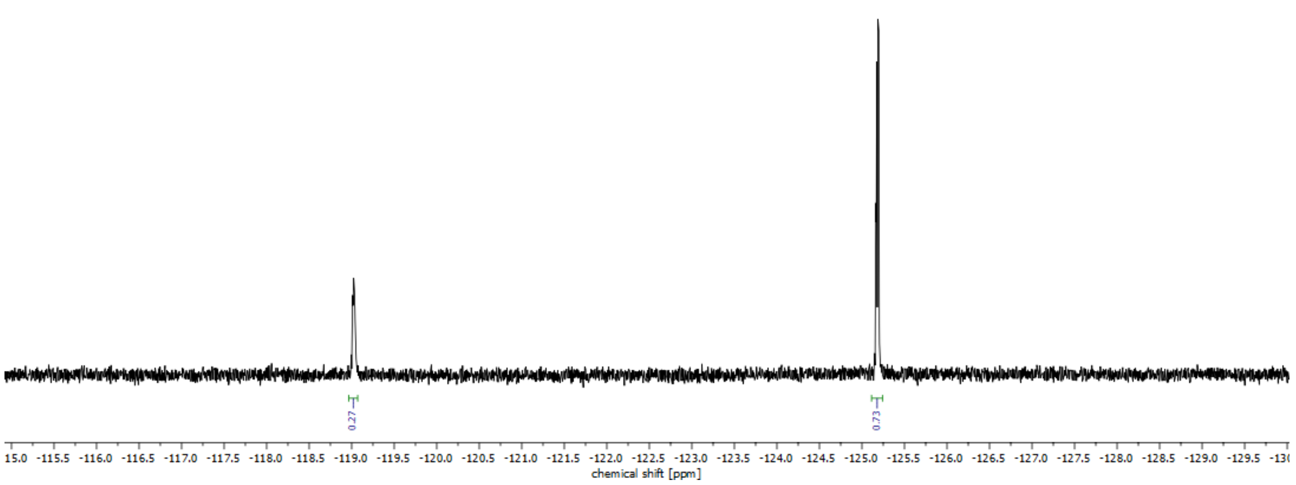

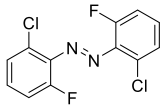

3

^{19}F NMR (471 MHz, 500 μM, DMSO-d_6/D_2O 9:1)
450 nm-adapted → 365 nm irradiation for 15 min
trans:*cis* = 73:27

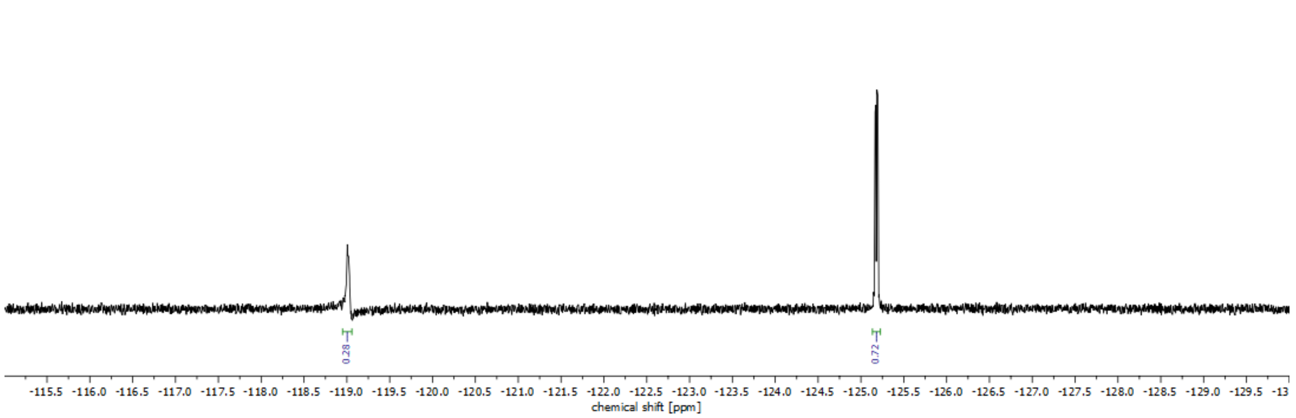

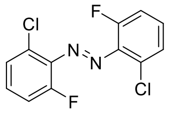

3

^{19}F NMR (471 MHz, 500 μM, DMSO-d_6/D_2O 9:1)
dark-adapted → 740 nm irradiation for 1 d
trans:*cis* = 72:28

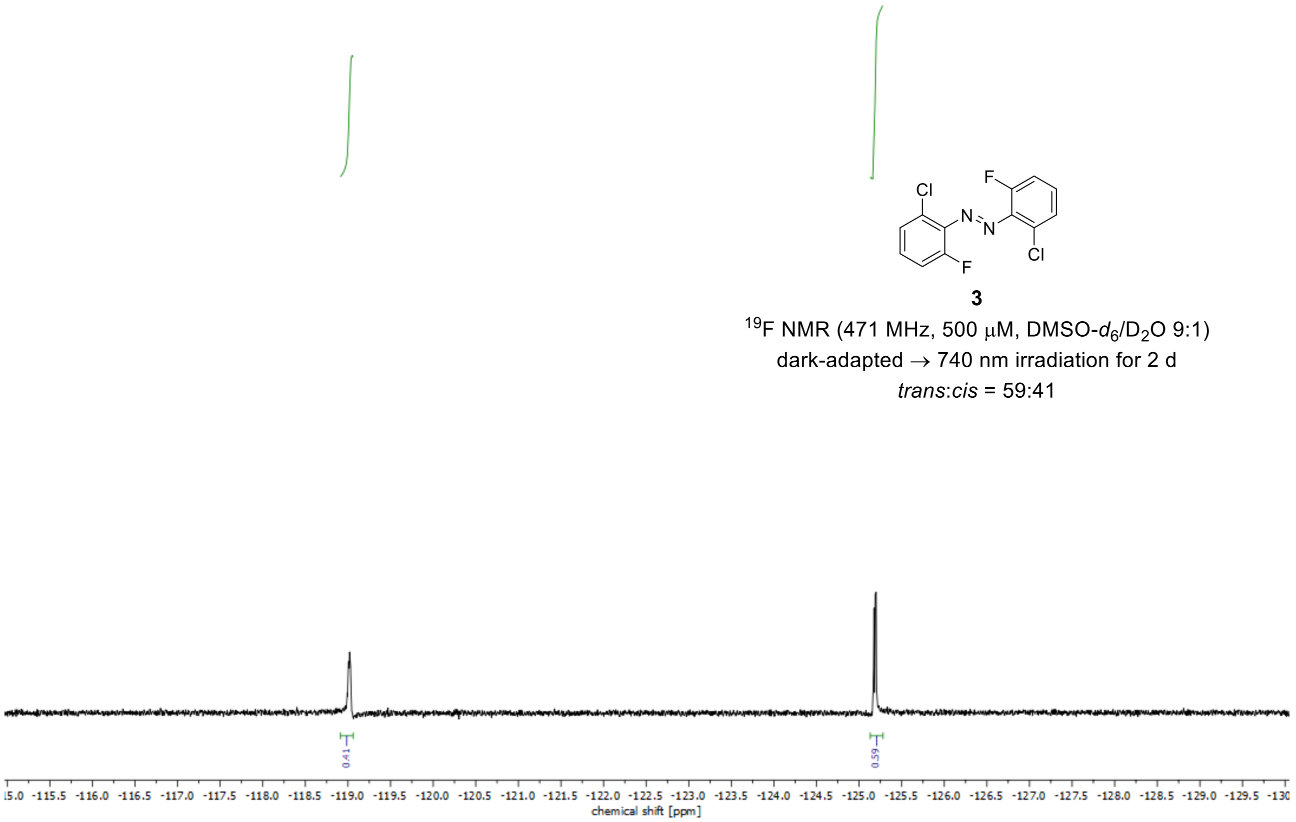

3

^{19}F NMR (471 MHz, 500 μM, DMSO-d_6/D_2O 9:1)
dark-adapted → 740 nm irradiation for 2 d
trans:*cis* = 59:41

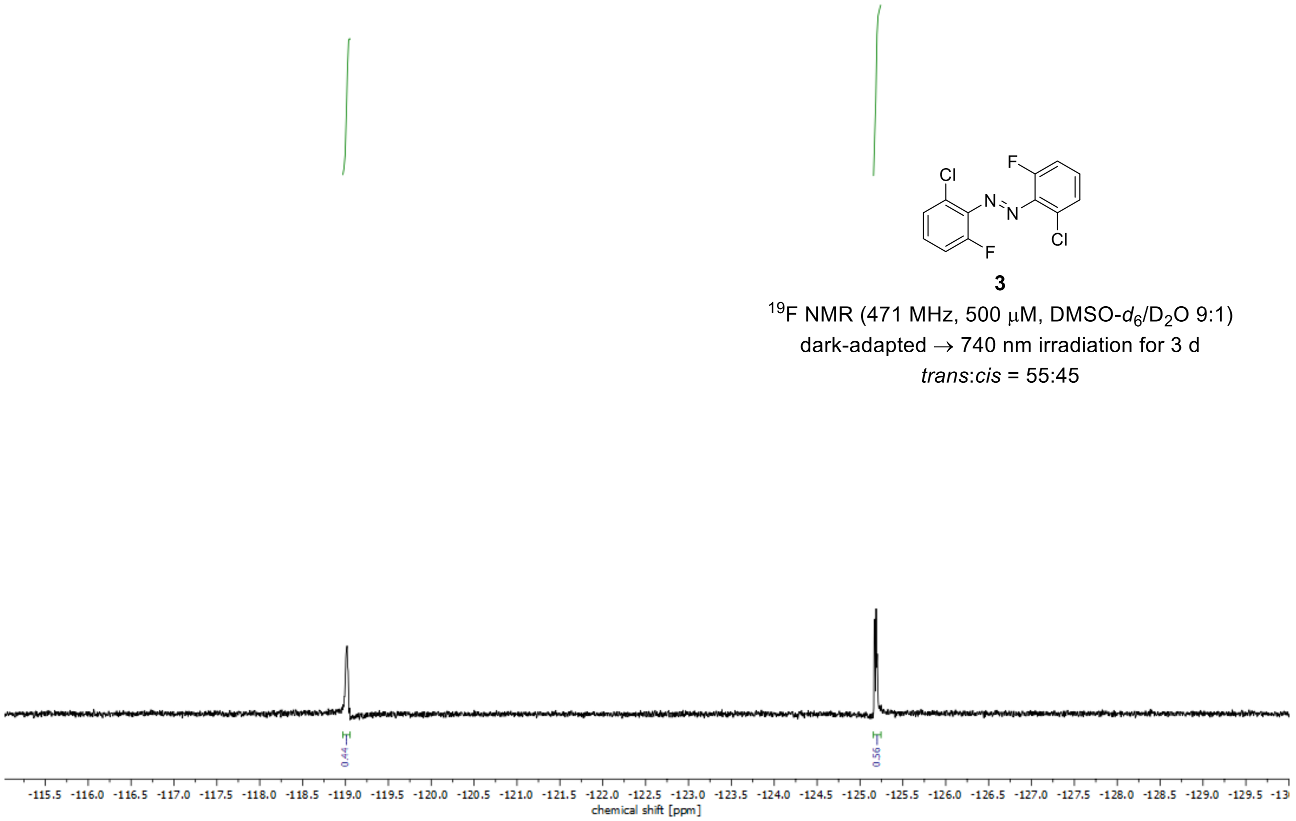

3

^{19}F NMR (471 MHz, 500 μM, DMSO-d_6/D_2O 9:1)
dark-adapted → 740 nm irradiation for 3 d
trans:*cis* = 55:45

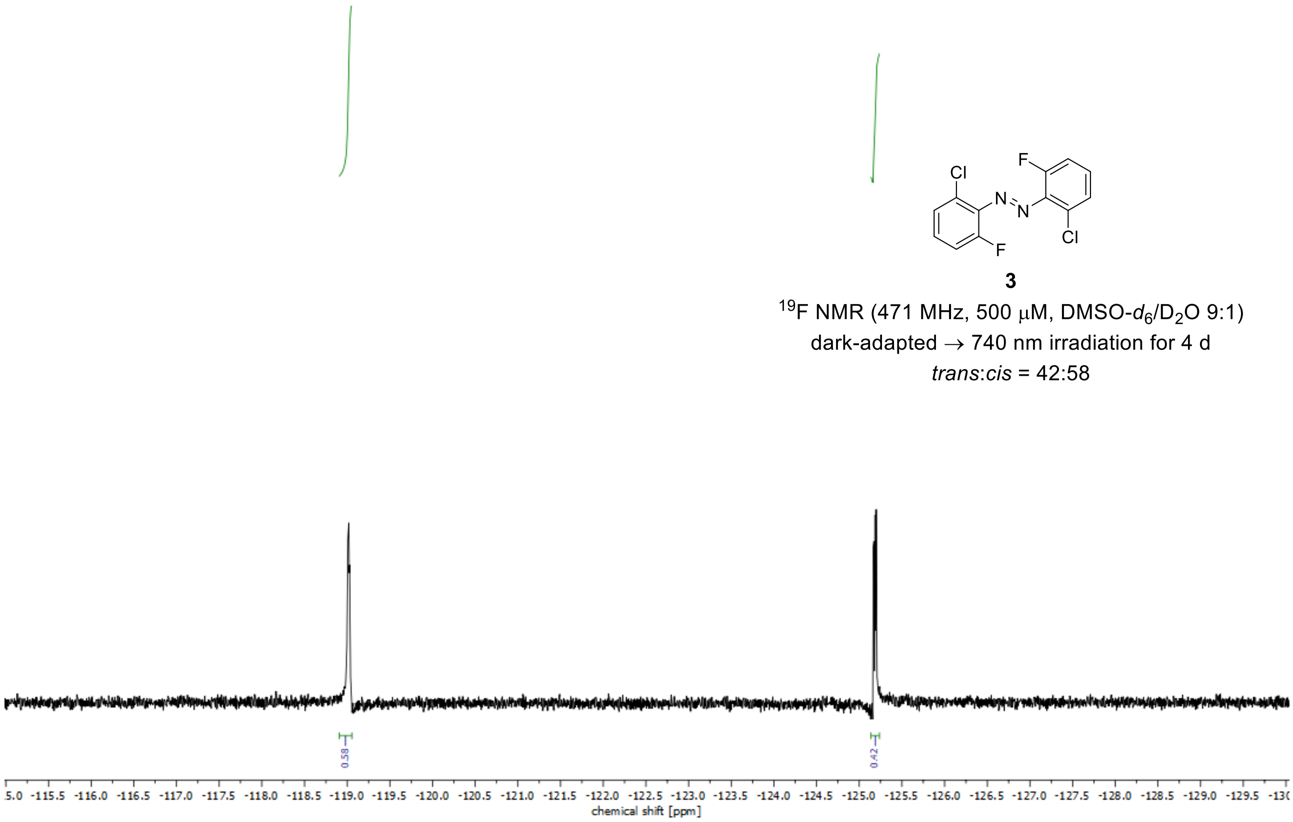

(*E*/*Z*)-1,2-Bis(2-chloro-6-fluorophenyl)diazene (3, 500 µM, DMSO-d_6/D_2O at 25 °C)

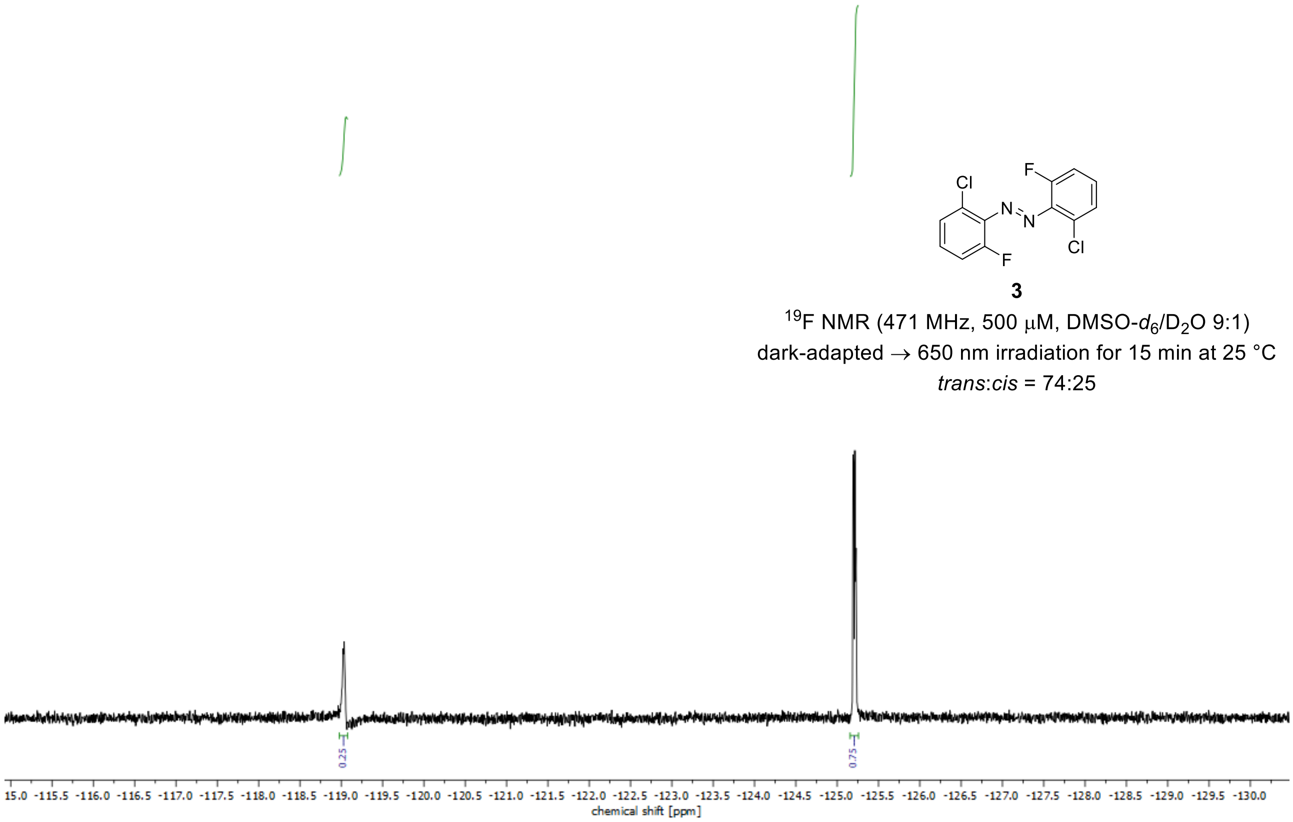

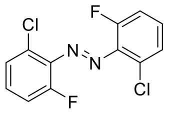

3

^{19}F NMR (471 MHz, 500 μM, DMSO-d_6/D_2O 9:1)
dark-adapted → 650 nm irradiation for 30 min at 25 °C
trans:*cis* = 50:50

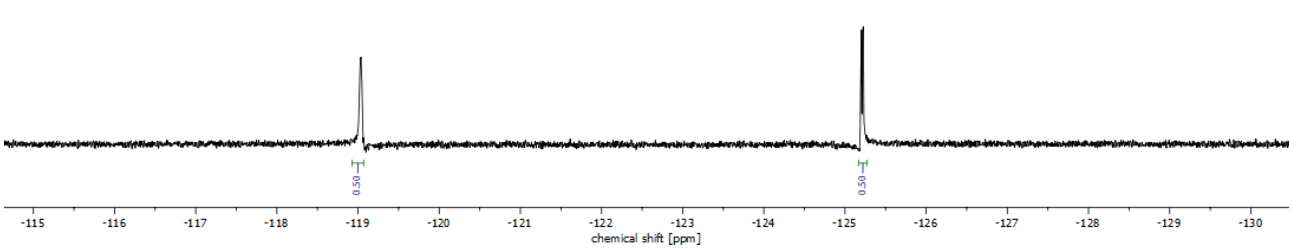

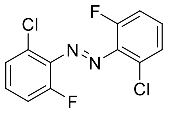

3

^{19}F NMR (471 MHz, 500 μM, DMSO-d_6/D_2O 9:1)
dark-adapted → 650 nm irradiation for 45 min at 25 °C
trans:*cis* = 38:62

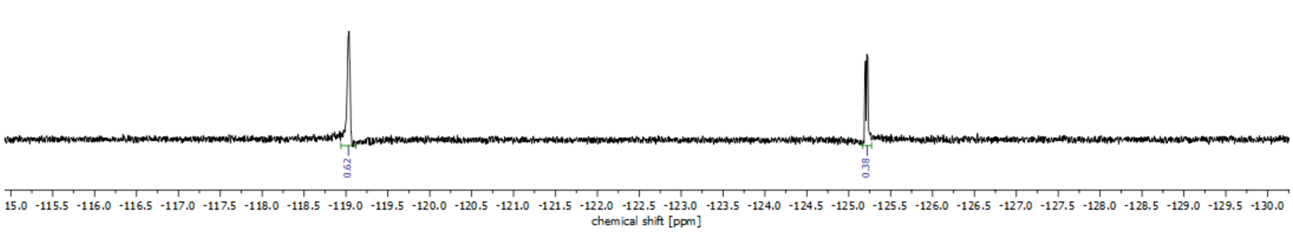

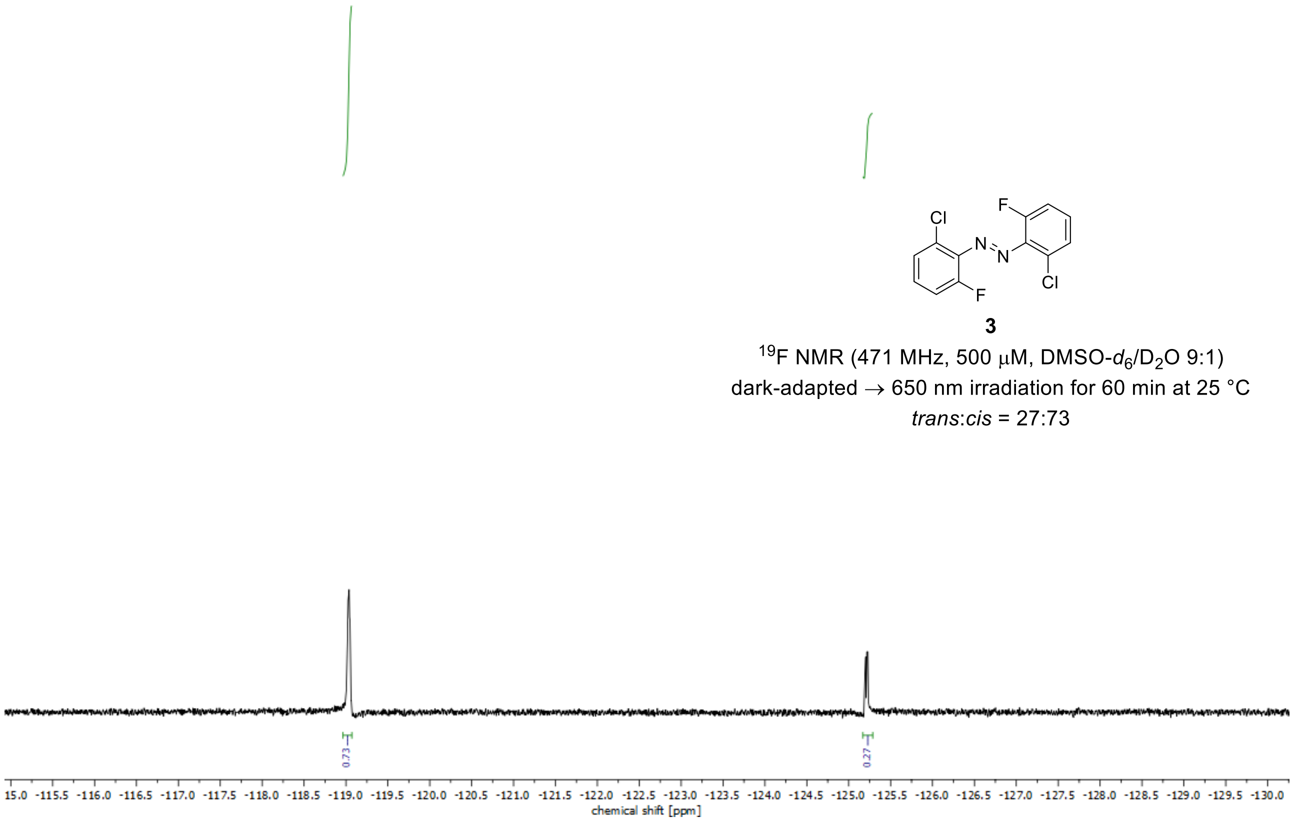

3

^{19}F NMR (471 MHz, 500 μM, DMSO-d_6/D_2O 9:1)
dark-adapted → 650 nm irradiation for 60 min at 25 °C
trans:*cis* = 27:73

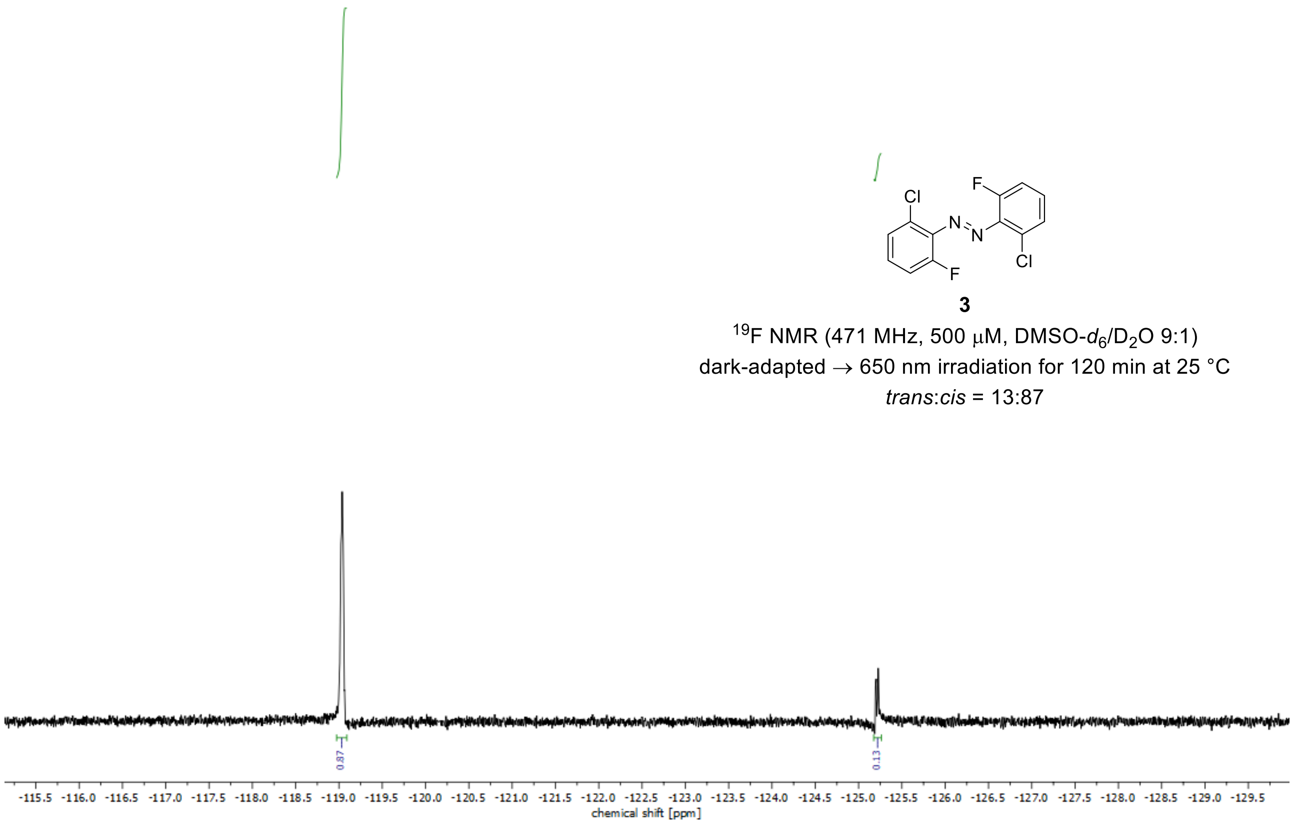

3

^{19}F NMR (471 MHz, 500 μM, DMSO-d_6/D_2O 9:1)
dark-adapted → 650 nm irradiation for 120 min at 25 °C
trans:*cis* = 13:87

(*E*/*Z*)-1,2-Bis(2-chloro-6-fluorophenyl)diazene (3, 500 µM, DMSO-d_6/D_2O at 37 °C)

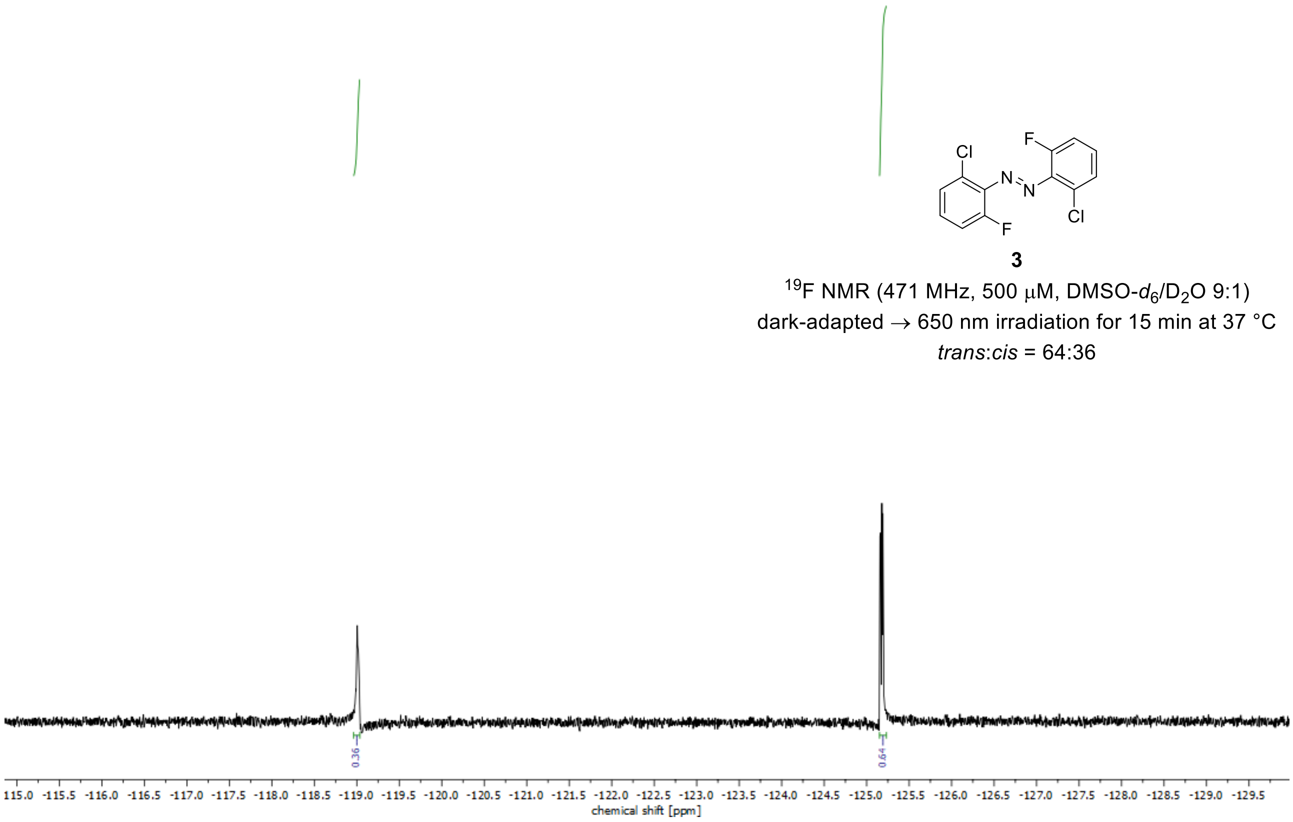

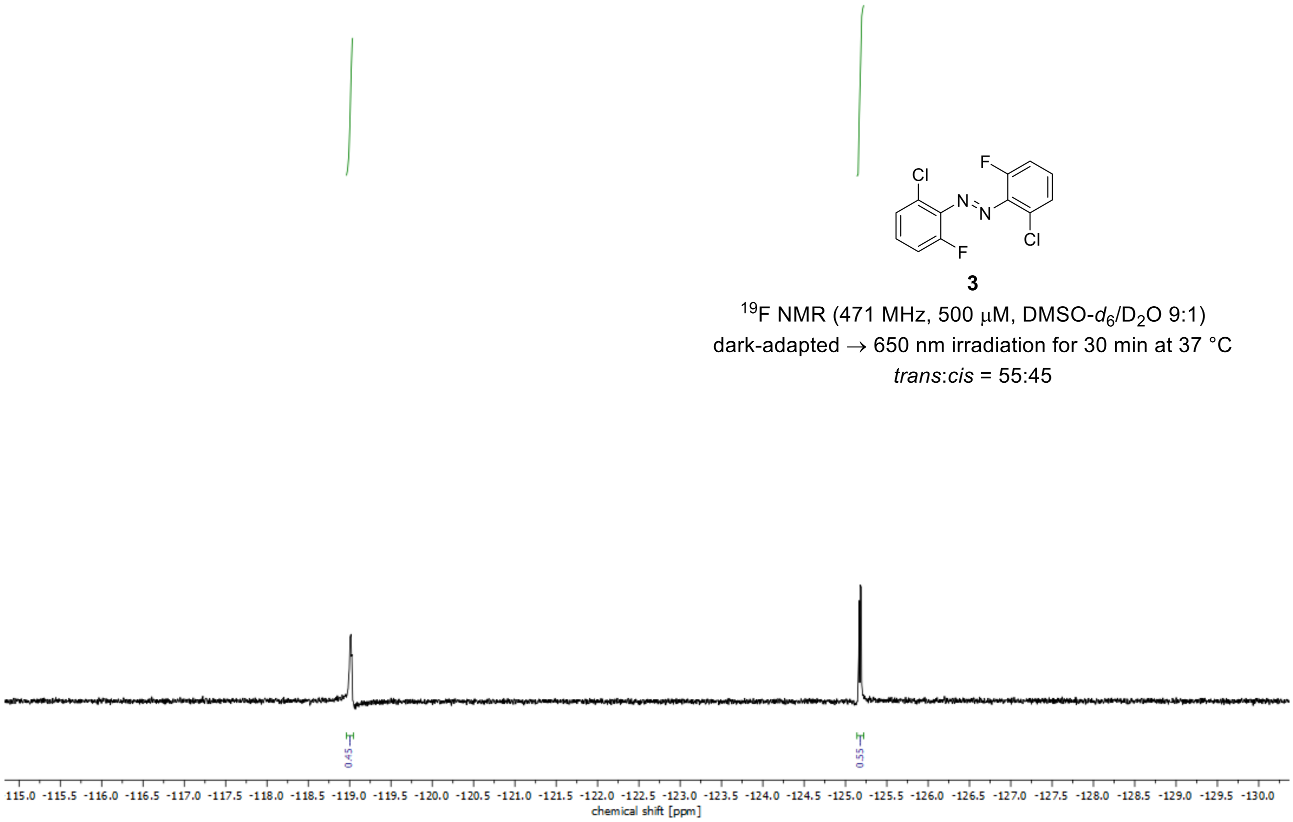

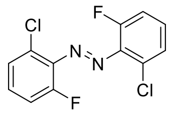

3

^{19}F NMR (471 MHz, 500 μM, DMSO-d_6/D_2O 9:1)
dark-adapted → 650 nm irradiation for 45 min at 37 °C
trans:*cis* = 37:63

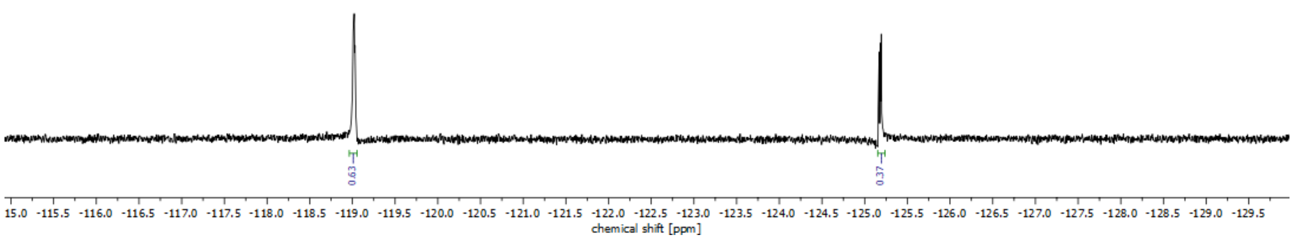

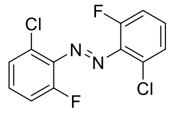

3

^{19}F NMR (471 MHz, 500 μM, DMSO-d_6/D_2O 9:1)
dark-adapted → 650 nm irradiation for 60 min at 37 °C
trans:*cis* = 30:70

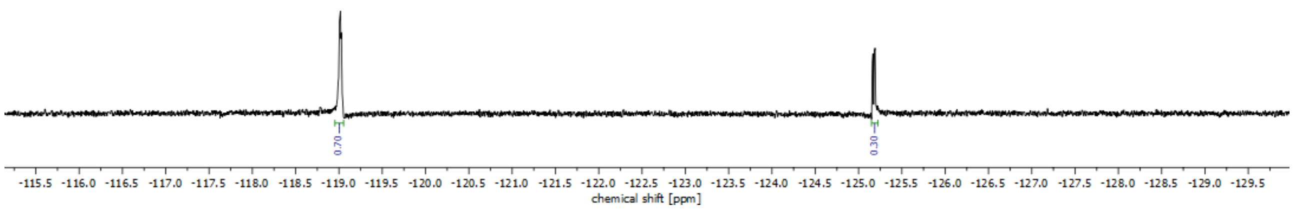

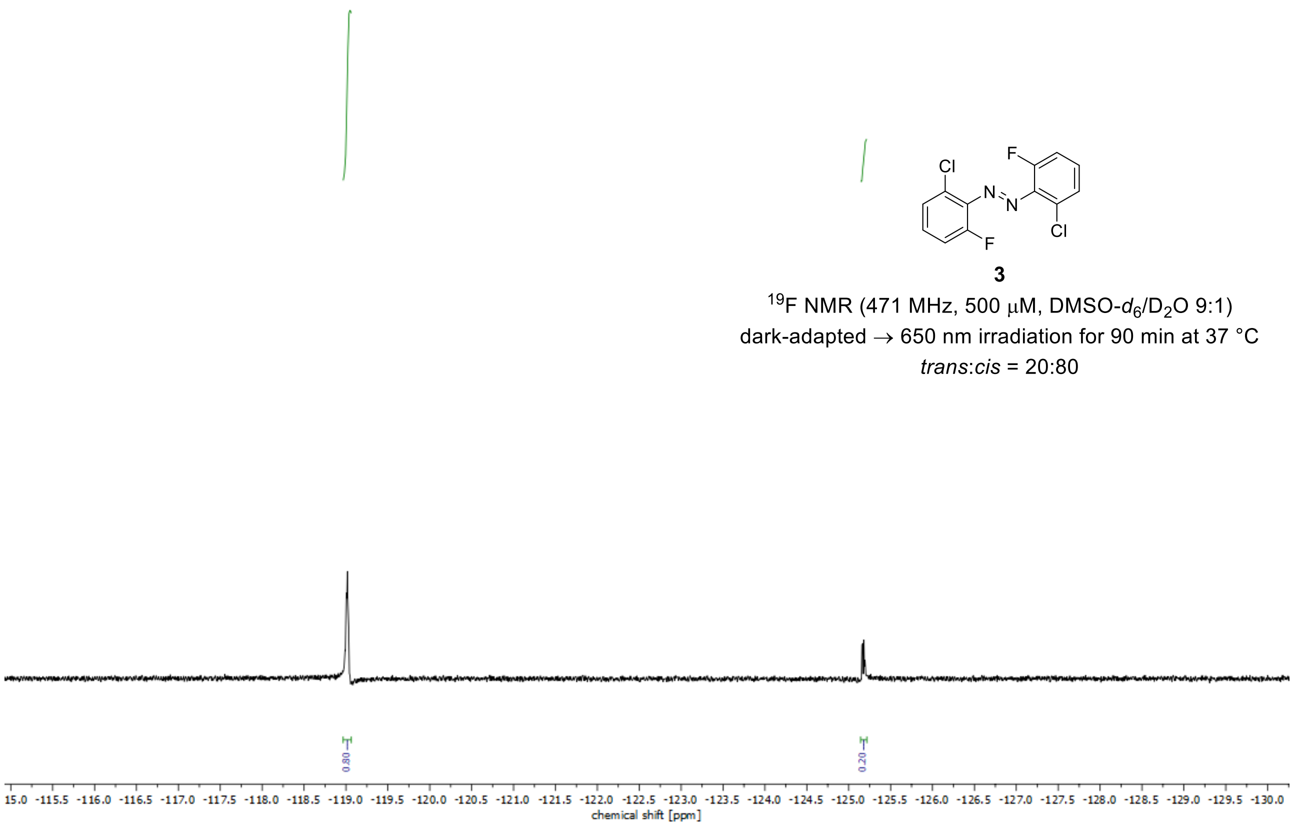

(*E*/*Z*)-1,2-Bis(2-chloro-6-fluorophenyl)diazene (3, 500 μM, DMSO-d_6/D_2O at 50 °C)

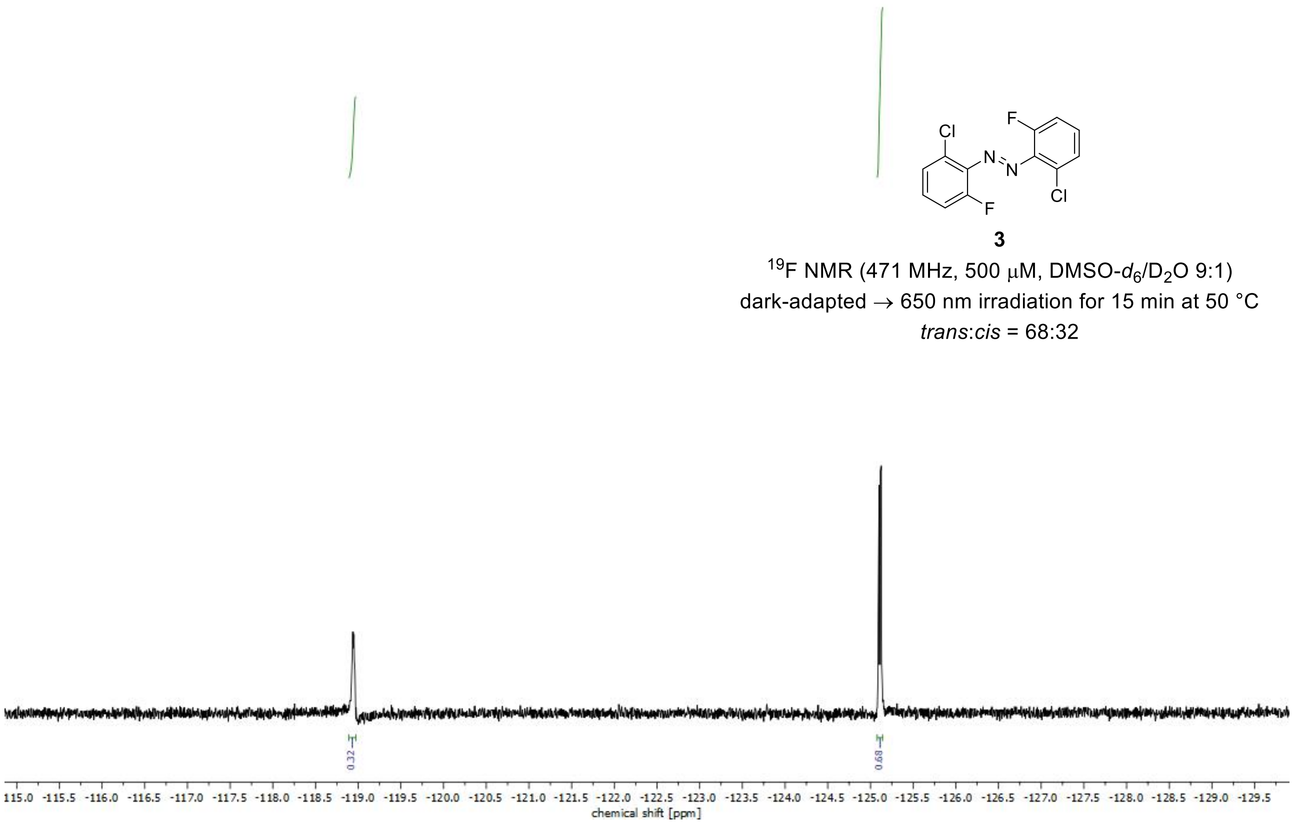

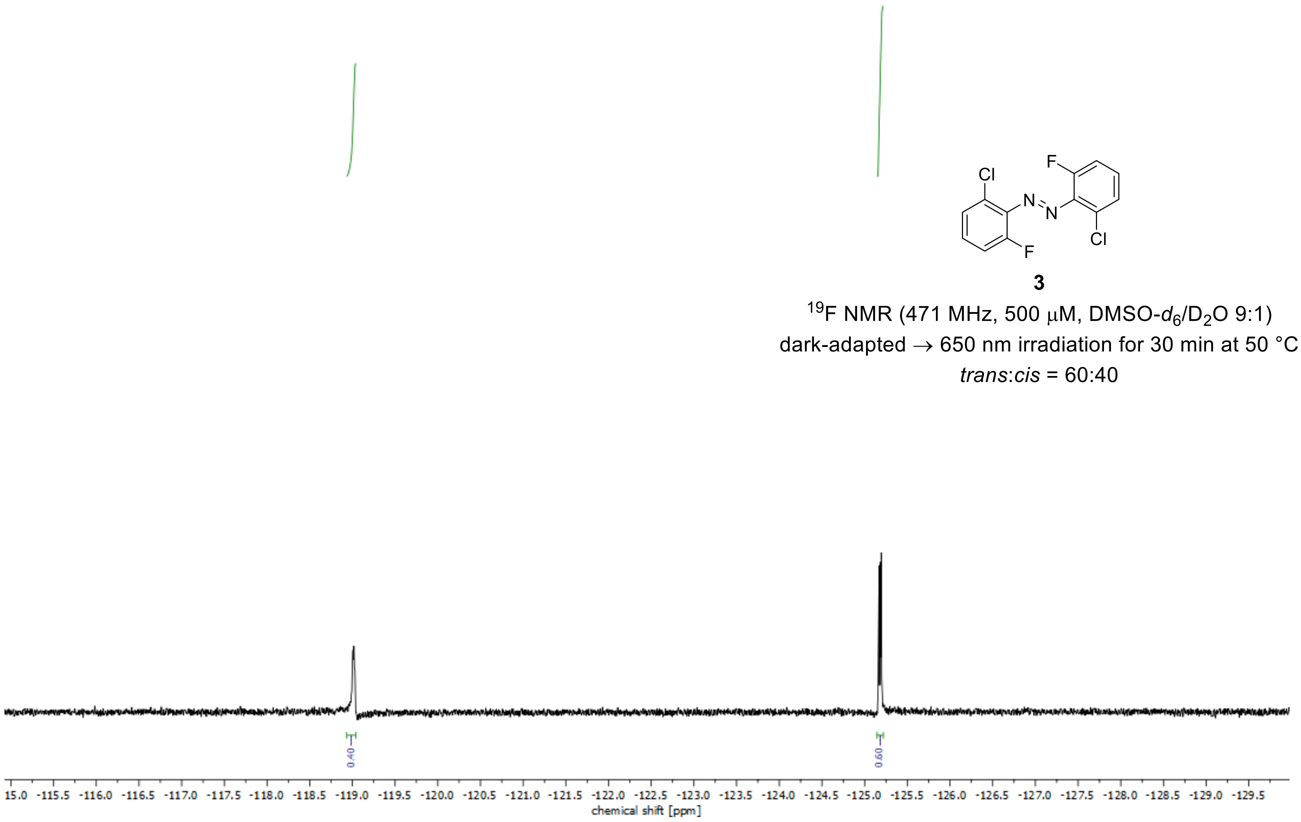

3

^{19}F NMR (471 MHz, 500 μM, DMSO-d_6/D_2O 9:1)
dark-adapted → 650 nm irradiation for 30 min at 50 °C
trans:*cis* = 60:40

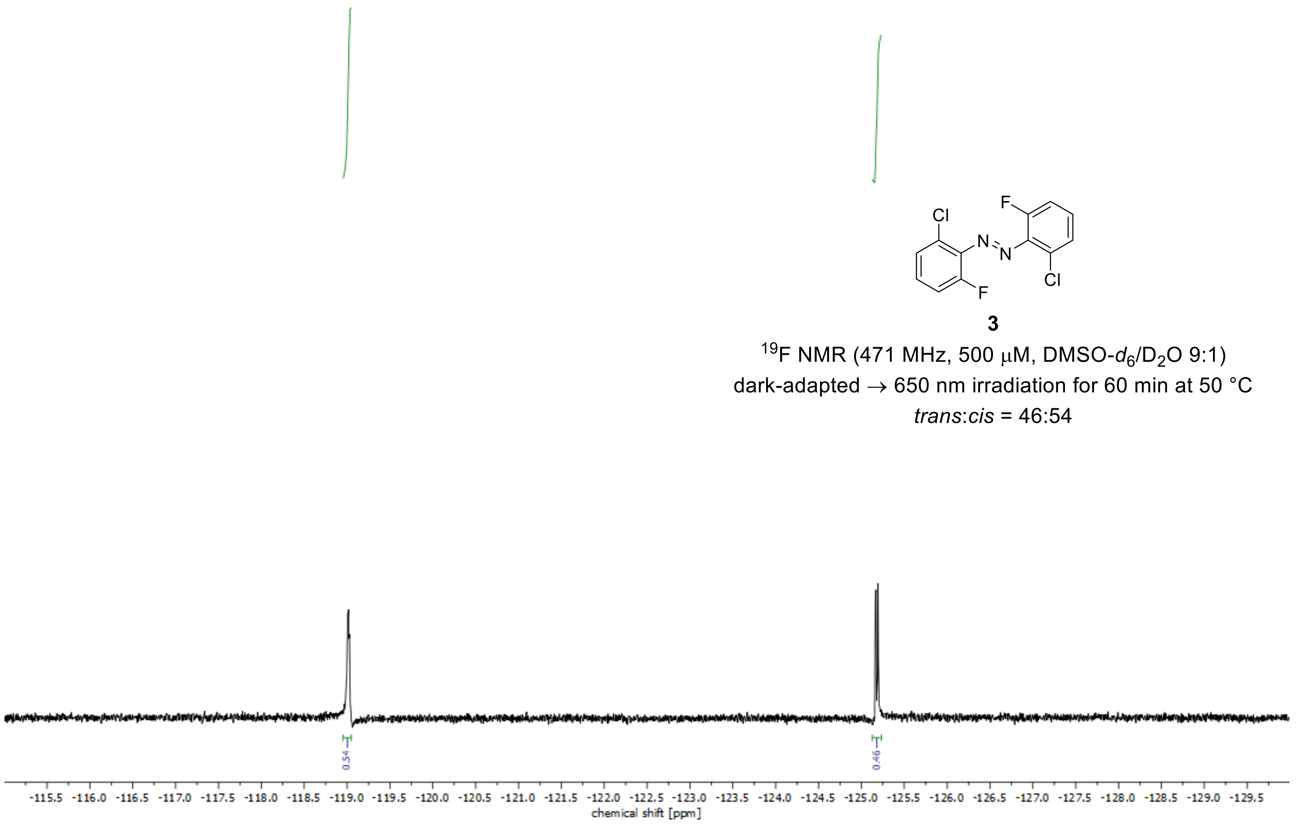

3

^{19}F NMR (471 MHz, 500 μM, DMSO-d_6/D_2O 9:1)
dark-adapted → 650 nm irradiation for 60 min at 50 °C
trans:*cis* = 46:54

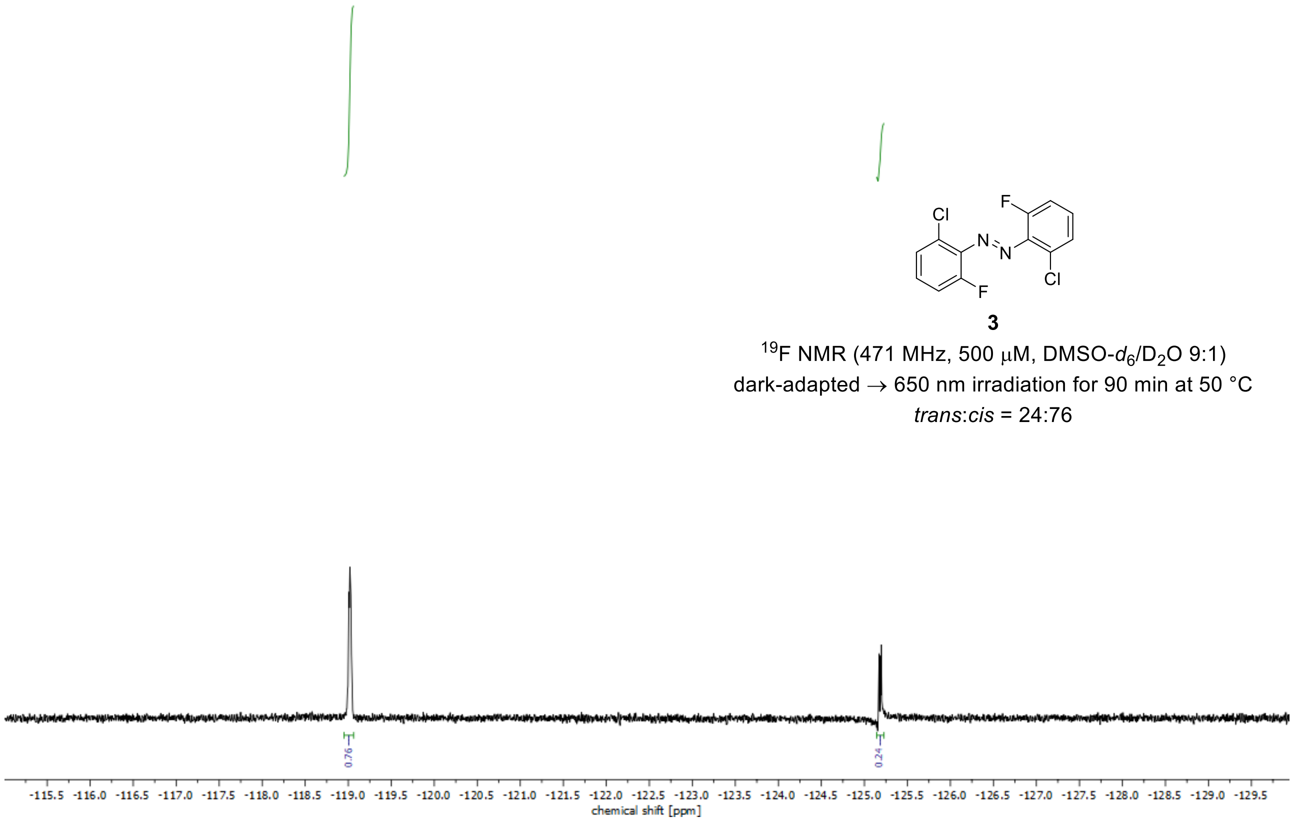

3

^{19}F NMR (471 MHz, 500 μM, DMSO-d_6/D_2O 9:1)
dark-adapted → 650 nm irradiation for 90 min at 50 °C
trans:*cis* = 24:76

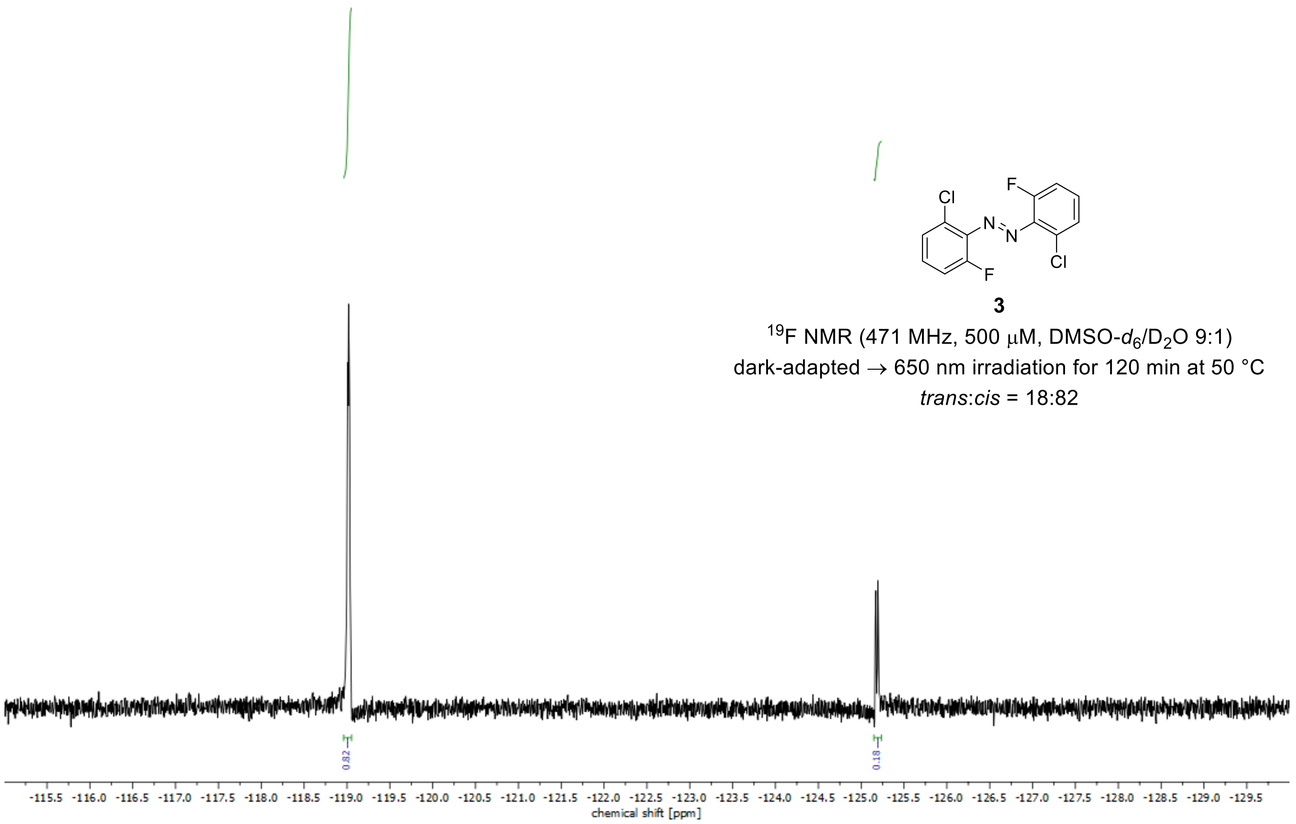

3

^{19}F NMR (471 MHz, 500 μM, DMSO-d_6/D_2O 9:1)
dark-adapted → 650 nm irradiation for 120 min at 50 °C
trans:*cis* = 18:82

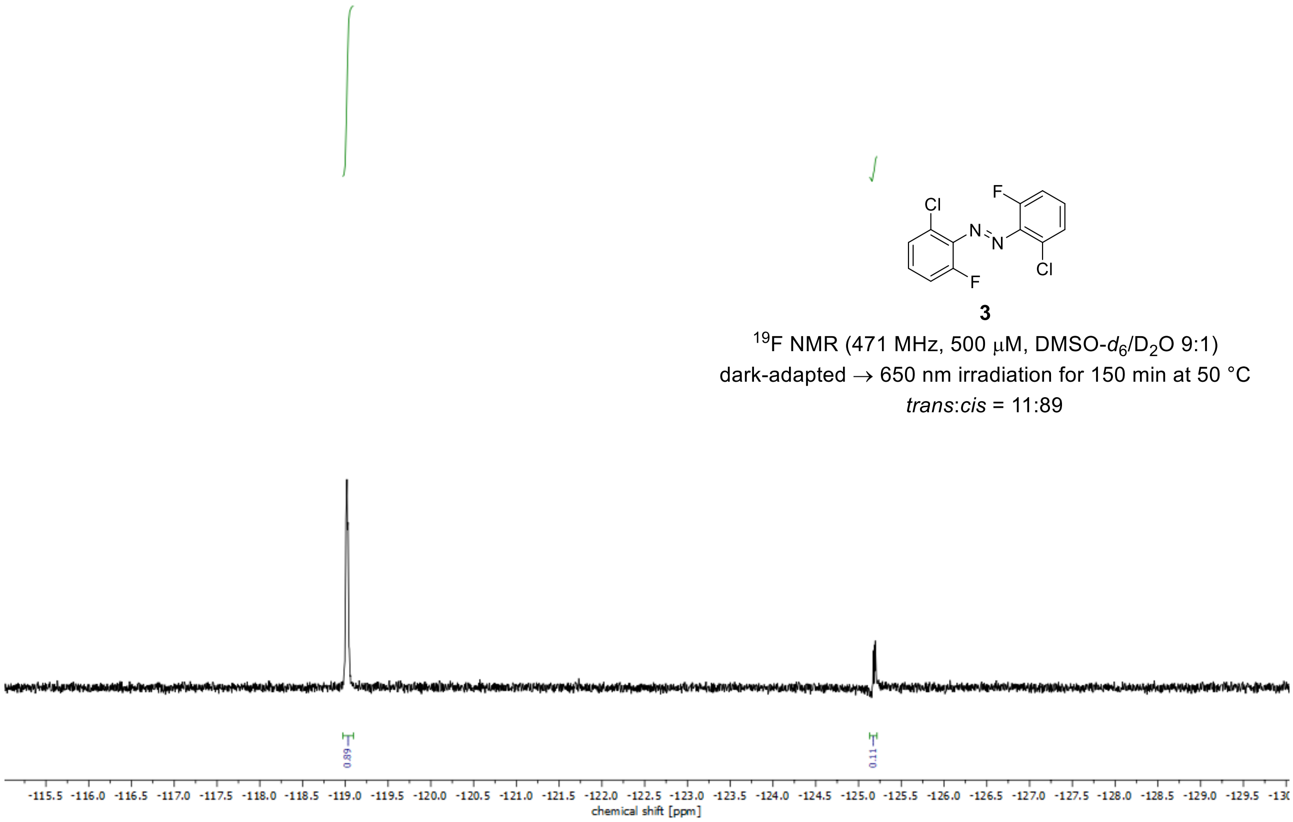

(*E*/*Z*)-1,2-Bis(2-bromo-6-fluorophenyl)diazene (4, 500 μM, DMSO-d_6/D_2O 9:1)

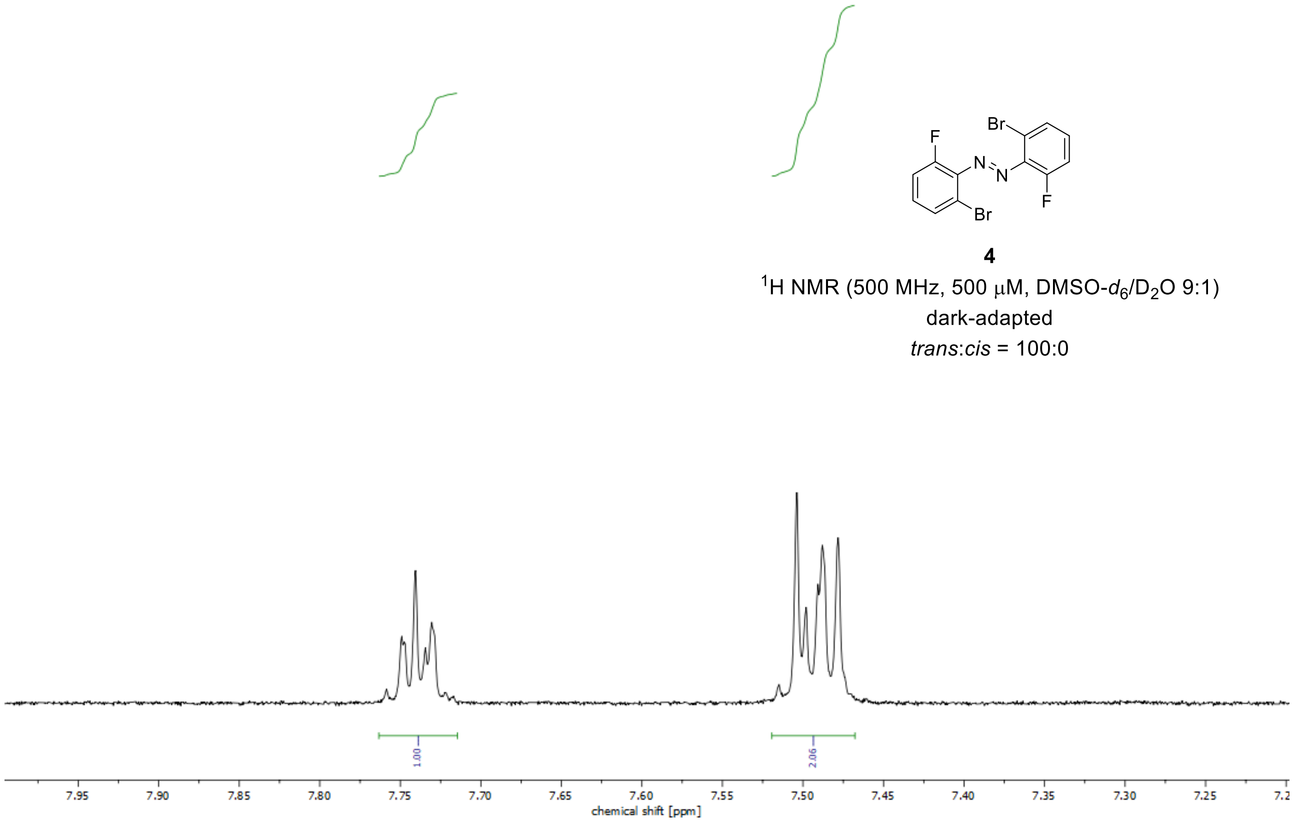

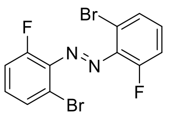

4

^{19}F NMR (471 MHz, 500 μM, DMSO-d_6/D_2O 9:1)
dark-adapted
trans:*cis* = 100:0

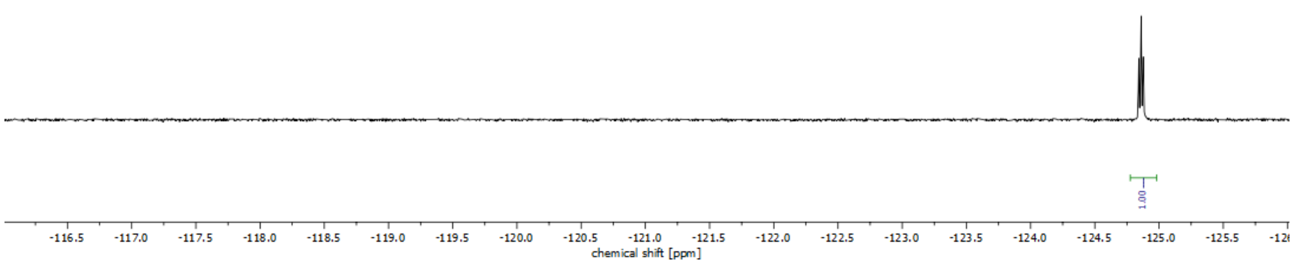

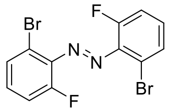

4

^{19}F NMR (471 MHz, 500 μM, DMSO-d_6/D_2O 9:1)
dark-adapted → 650 nm irradiation for 15 min
trans:*cis* = 55:45

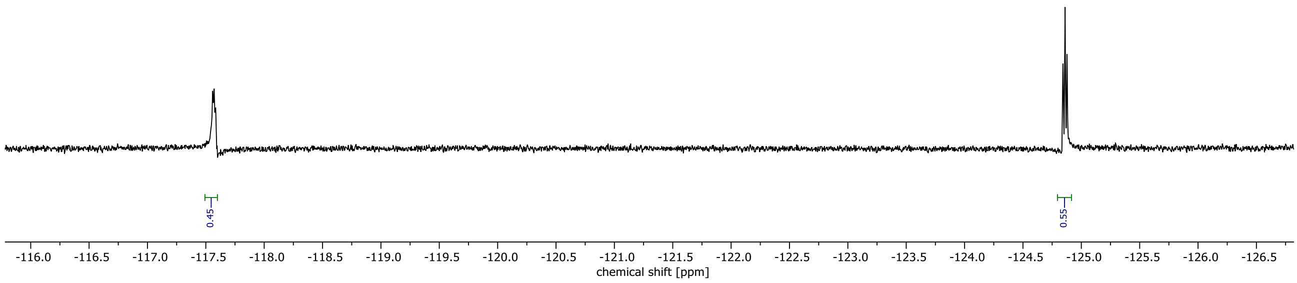

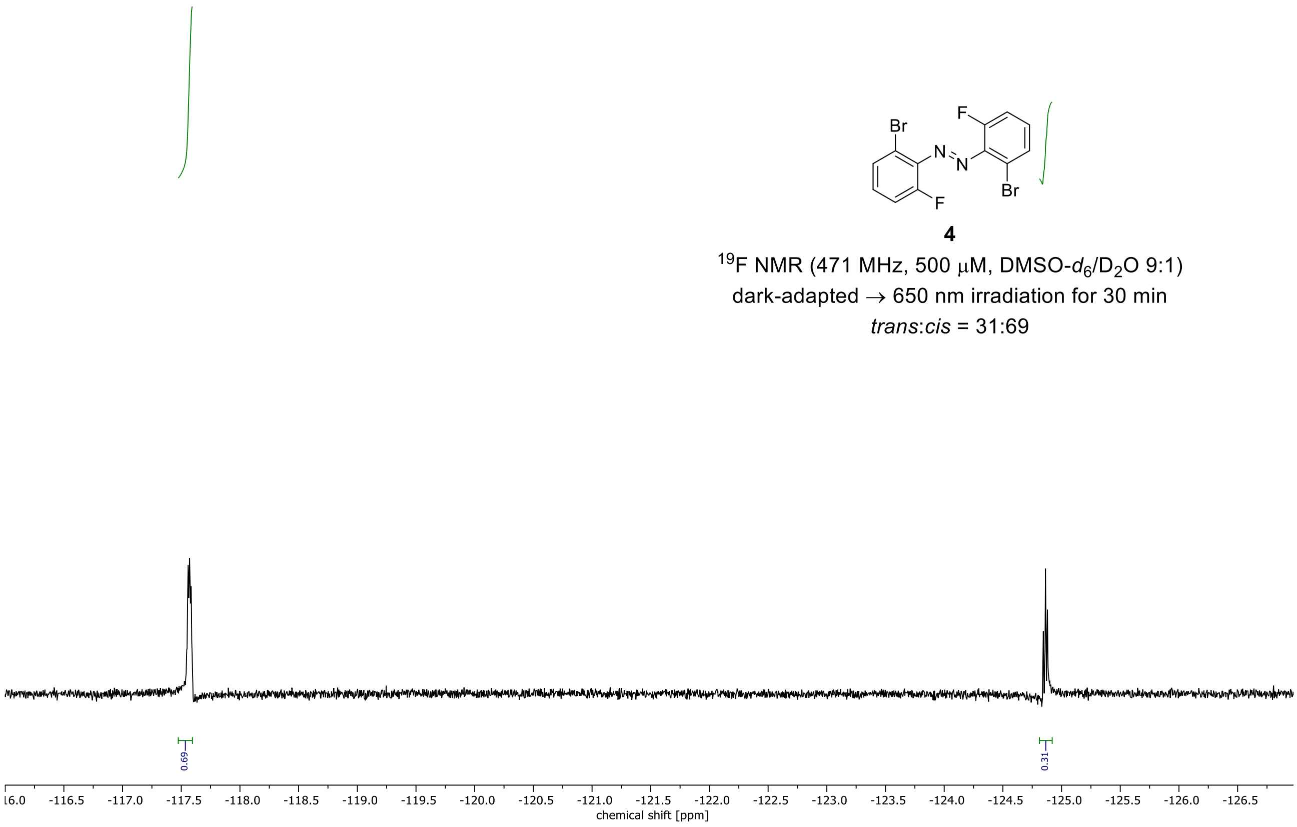

4

^{19}F NMR (471 MHz, 500 μM, DMSO-d_6/D_2O 9:1)
dark-adapted → 650 nm irradiation for 30 min
trans:*cis* = 31:69

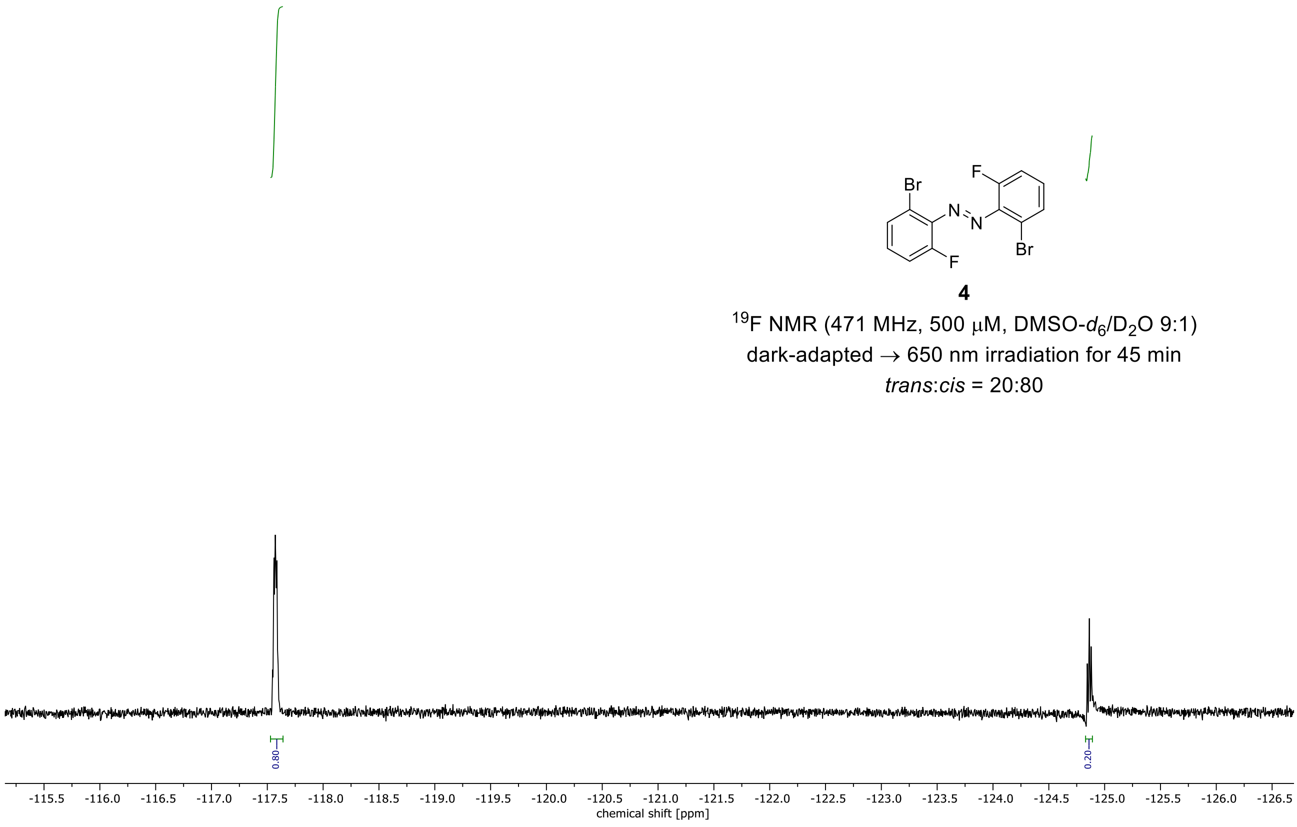

4

^{19}F NMR (471 MHz, 500 μM, DMSO-d_6/D_2O 9:1)
dark-adapted → 650 nm irradiation for 45 min
trans:*cis* = 20:80

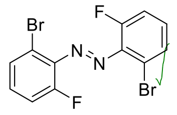

4

^{19}F NMR (471 MHz, 500 μM, DMSO-d_6/D_2O 9:1)
dark-adapted → 650 nm irradiation for 60 min
trans:*cis* = 17:83

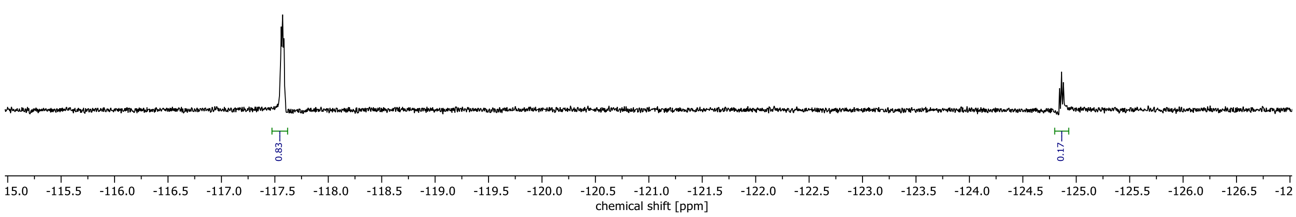

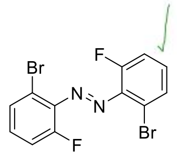

4

^{19}F NMR (471 MHz, 500 μM, DMSO-d_6/D_2O 9:1)
dark-adapted → 525 nm irradiation for 15 min
trans:*cis* = 20:80

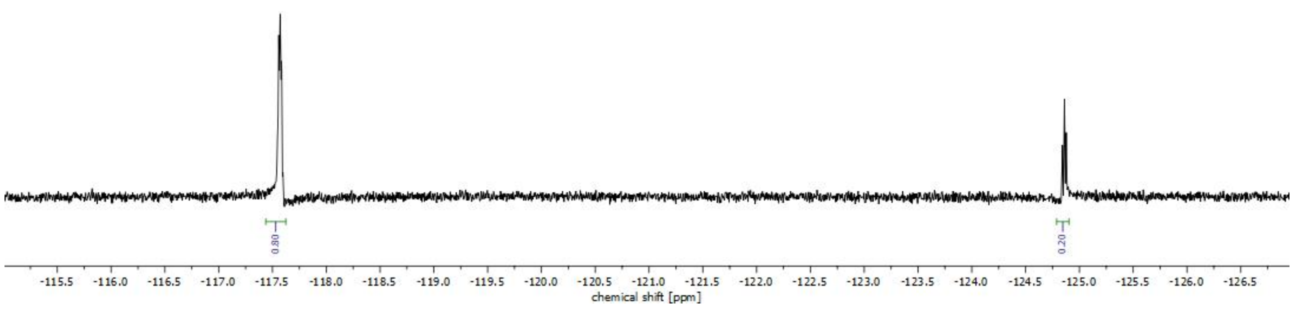

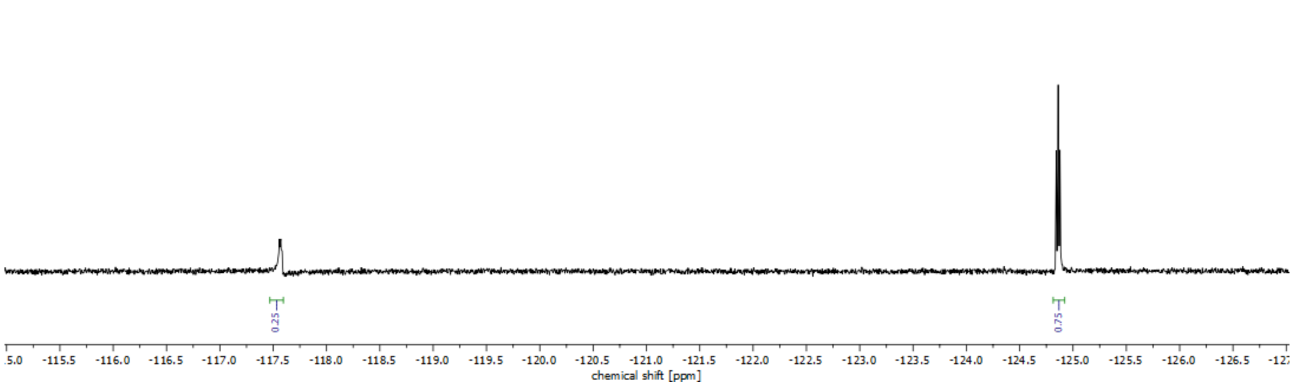

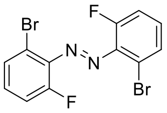

4

^{19}F NMR (471 MHz, 500 μM, DMSO-d_6/D_2O 9:1)

525 nm adapted → 450 nm irradiation for 15 min

trans:*cis* = 75:25

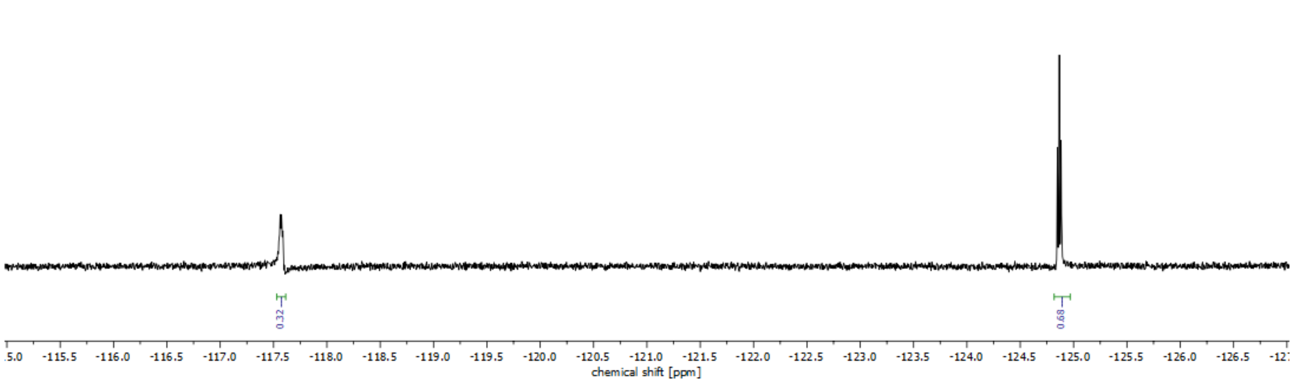

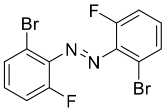

4

^{19}F NMR (471 MHz, 500 μM, DMSO-d_6/D_2O 9:1)

450 nm adapted → 365 nm irradiation for 15 min

trans:*cis* = 68:32

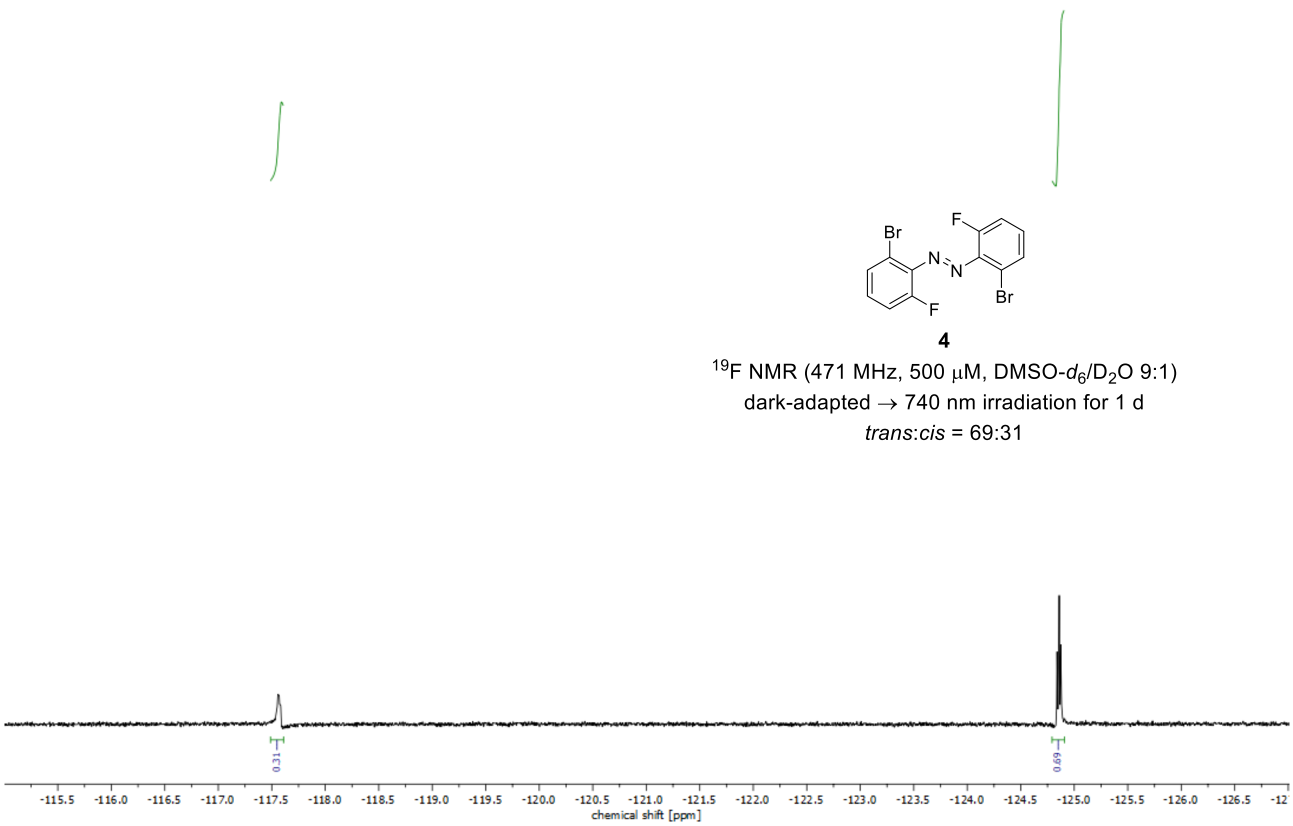

(*E*/*Z*)-1,2-Bis(2-bromo-6-fluorophenyl)diazene (4, 500 μM, DMSO-d_6/D_2O at 25 °C)

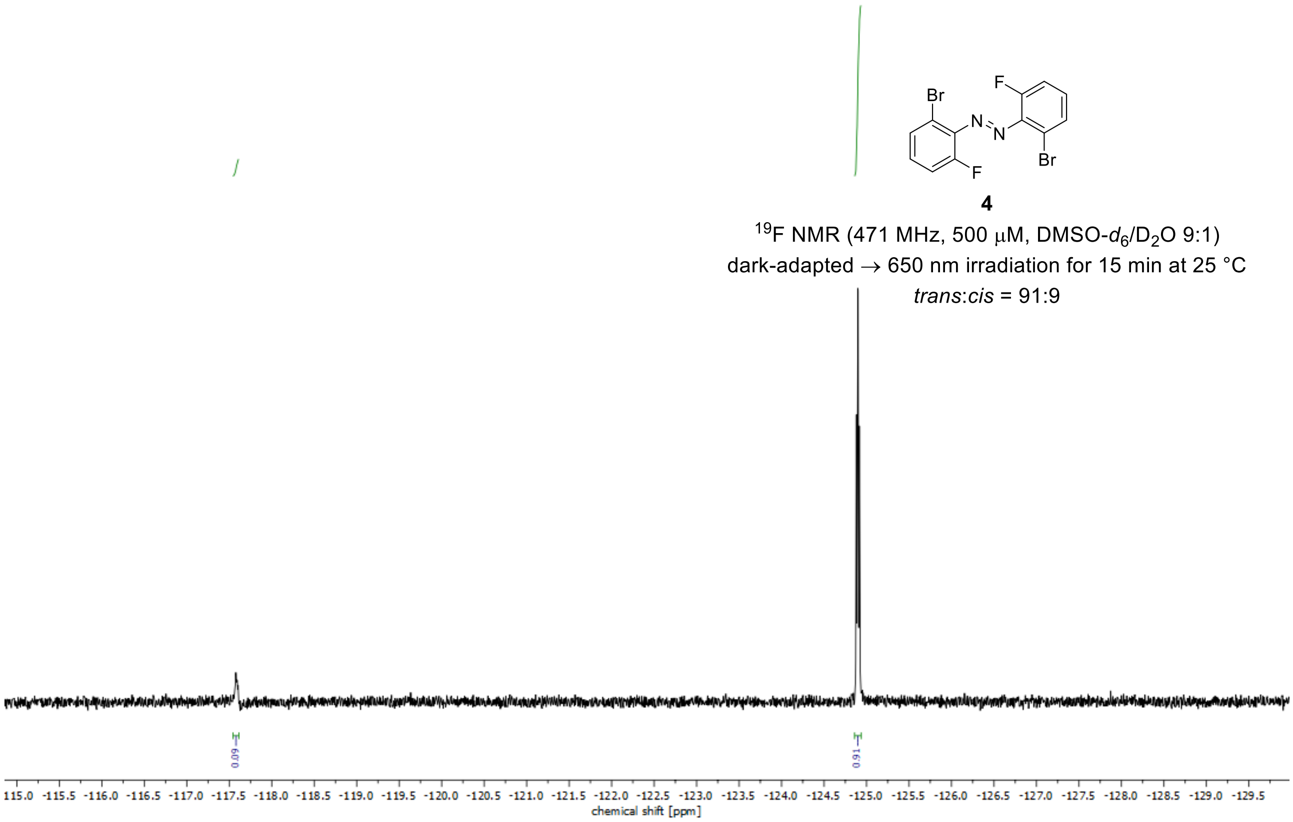

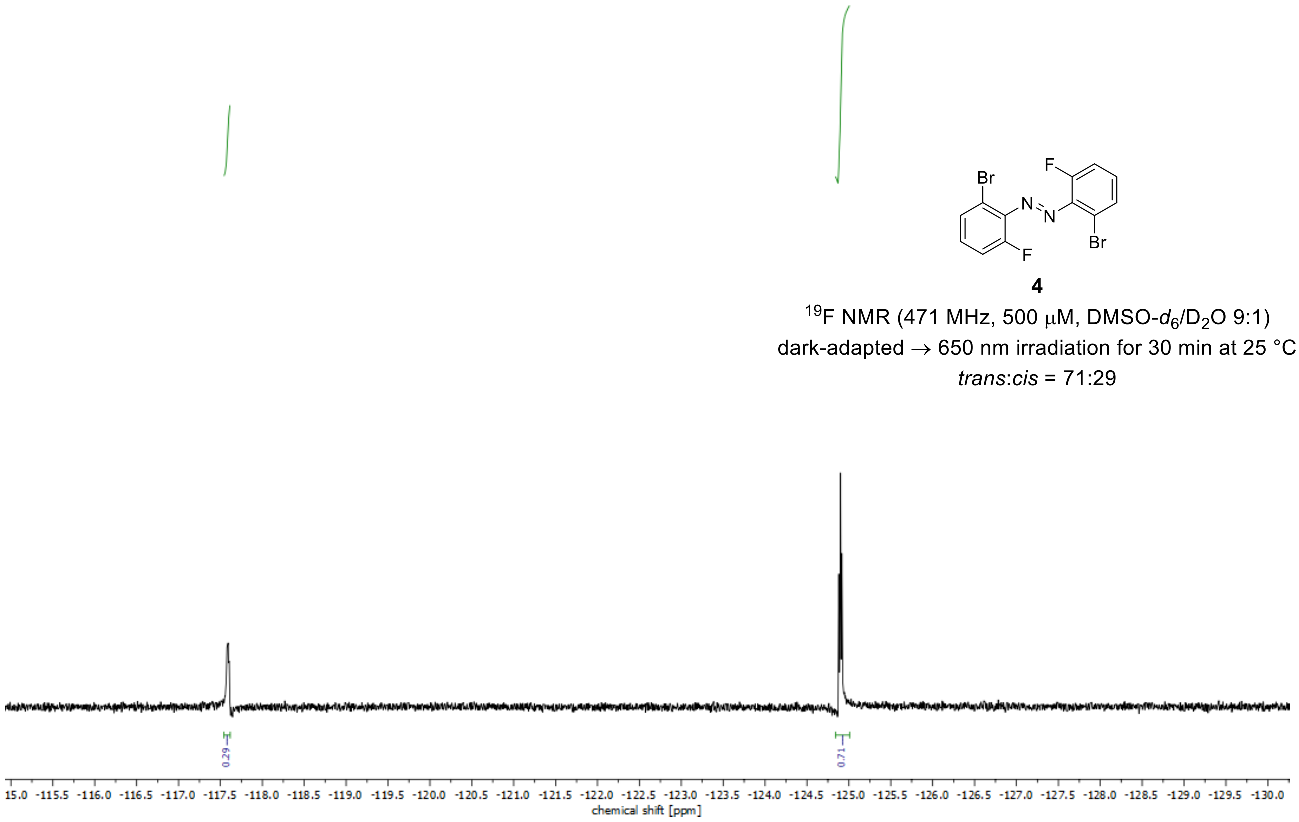
Br
F
N
N
Br
F
4
^{19}F NMR (471 MHz, 500 µM, DMSO-d_6/D_2O 9:1)
dark-adapted → 650 nm irradiation for 30 min at 25 °C
trans:*cis* = 71:29
0.29
0.71
chemical shift [ppm]

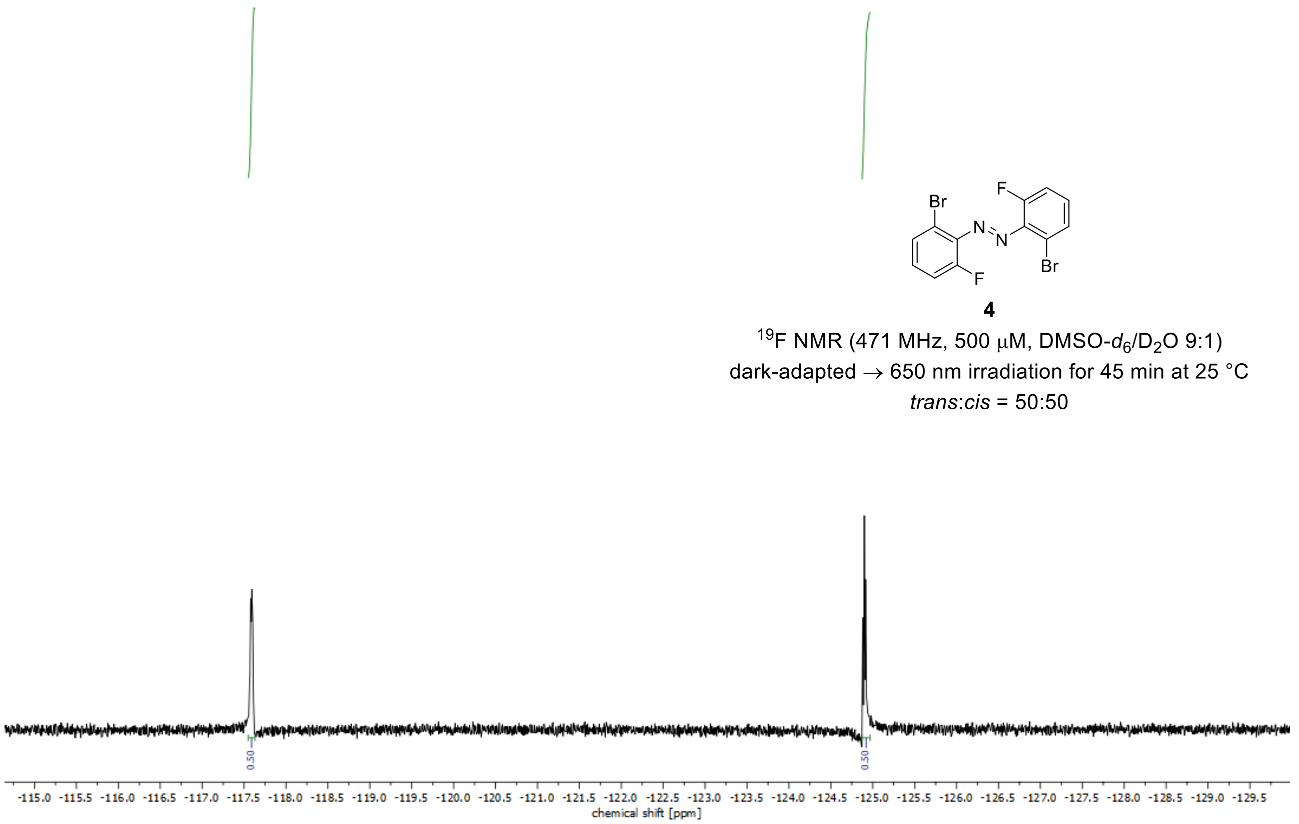
Br
F
N
N
Br
F
4
^{19}F NMR (471 MHz, 500 µM, DMSO-d_6/D_2O 9:1)
dark-adapted → 650 nm irradiation for 45 min at 25 °C
trans:*cis* = 50:50
0.50
0.50
chemical shift [ppm]

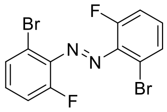

4

^{19}F NMR (471 MHz, 500 μM, DMSO-d_6/D_2O 9:1)
dark-adapted → 650 nm irradiation for 60 min at 25 °C
trans:*cis* = 43:57

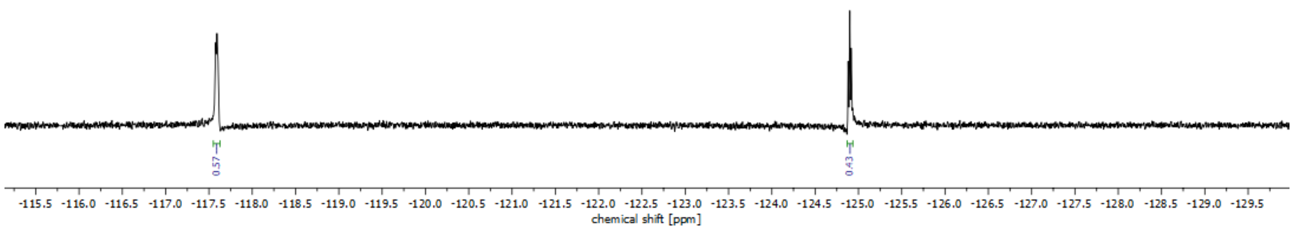

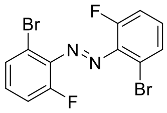

4

^{19}F NMR (471 MHz, 500 μM, DMSO-d_6/D_2O 9:1)
dark-adapted → 650 nm irradiation for 90 min at 25 °C
trans:*cis* = 36:64

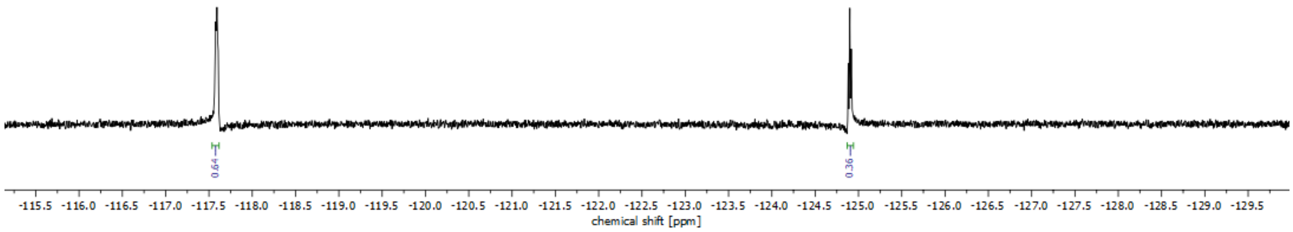

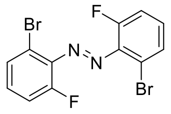

4

^{19}F NMR (471 MHz, 500 μM, DMSO-d_6/D_2O 9:1)

dark-adapted → 650 nm irradiation for 120 min at 25 °C

trans:*cis* = 32:68

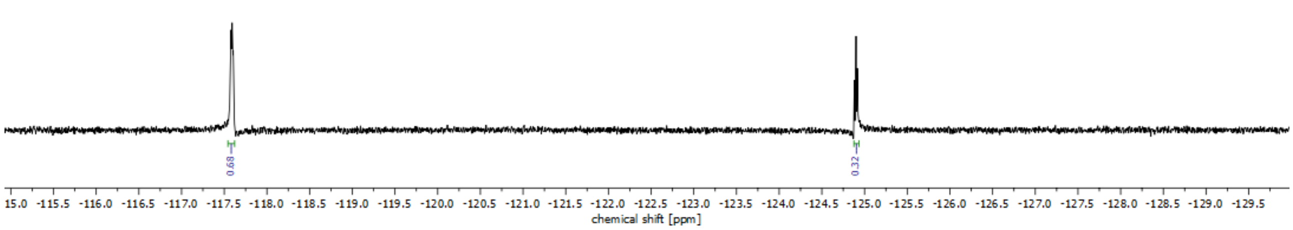

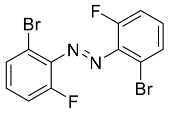

4

^{19}F NMR (471 MHz, 500 μM, DMSO-d_6/D_2O 9:1)

dark-adapted → 650 nm irradiation for 150 min at 25 °C

trans:*cis* = 29:71

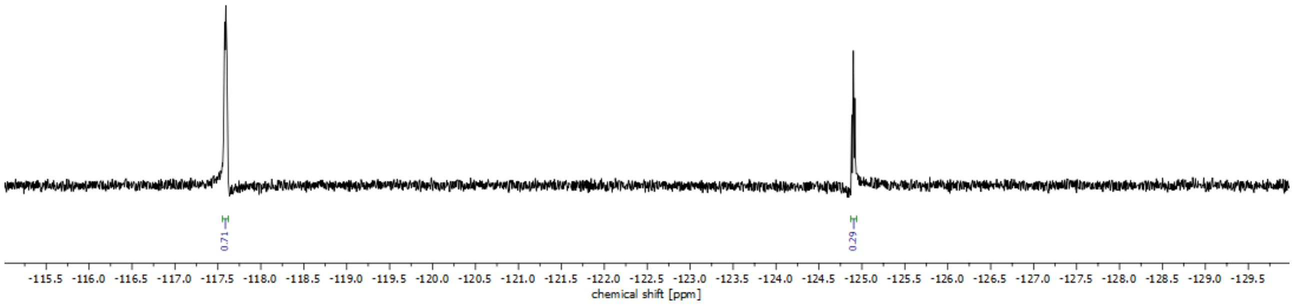

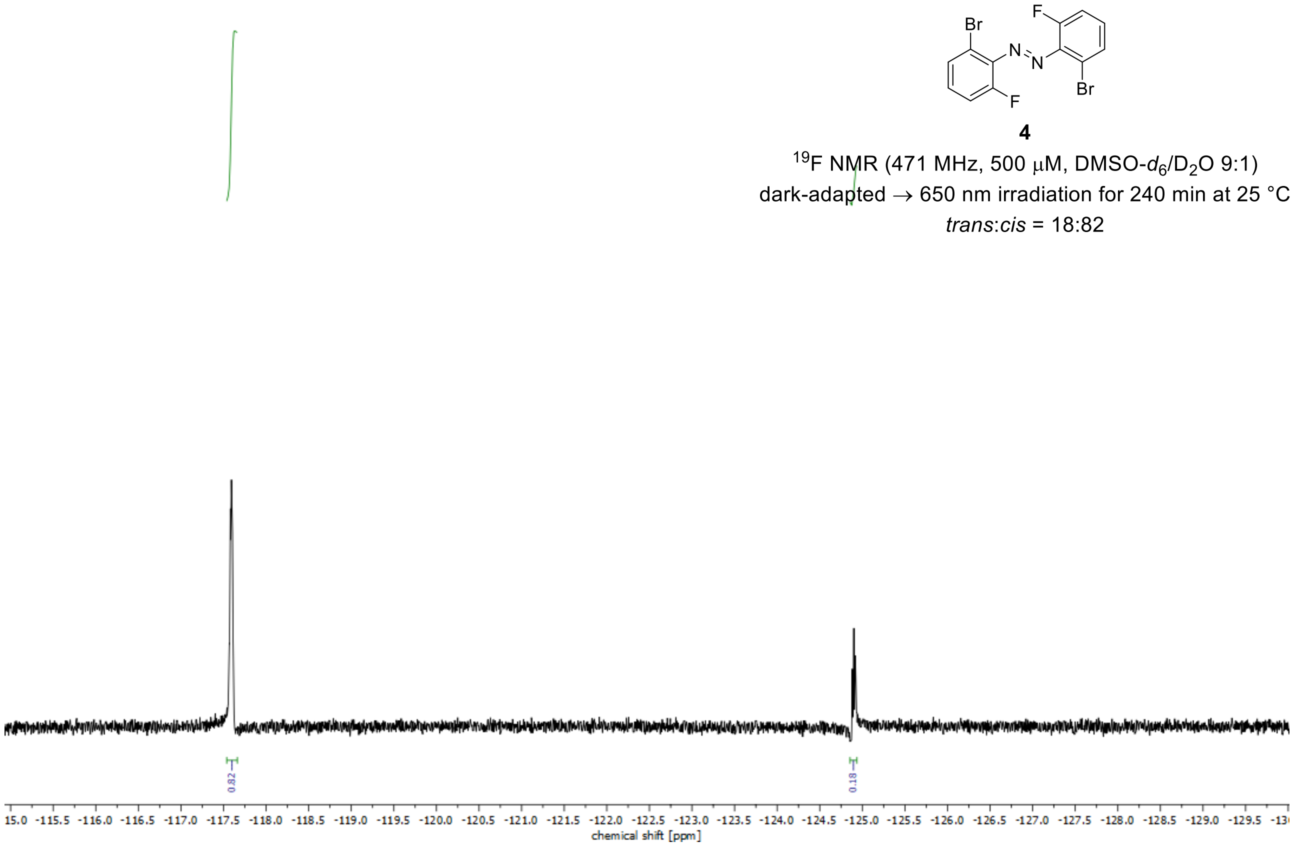

(*E*/*Z*)-1,2-Bis(2-bromo-6-fluorophenyl)diazene (4, 500 µM, DMSO-d_6/D_2O at 37 °C)

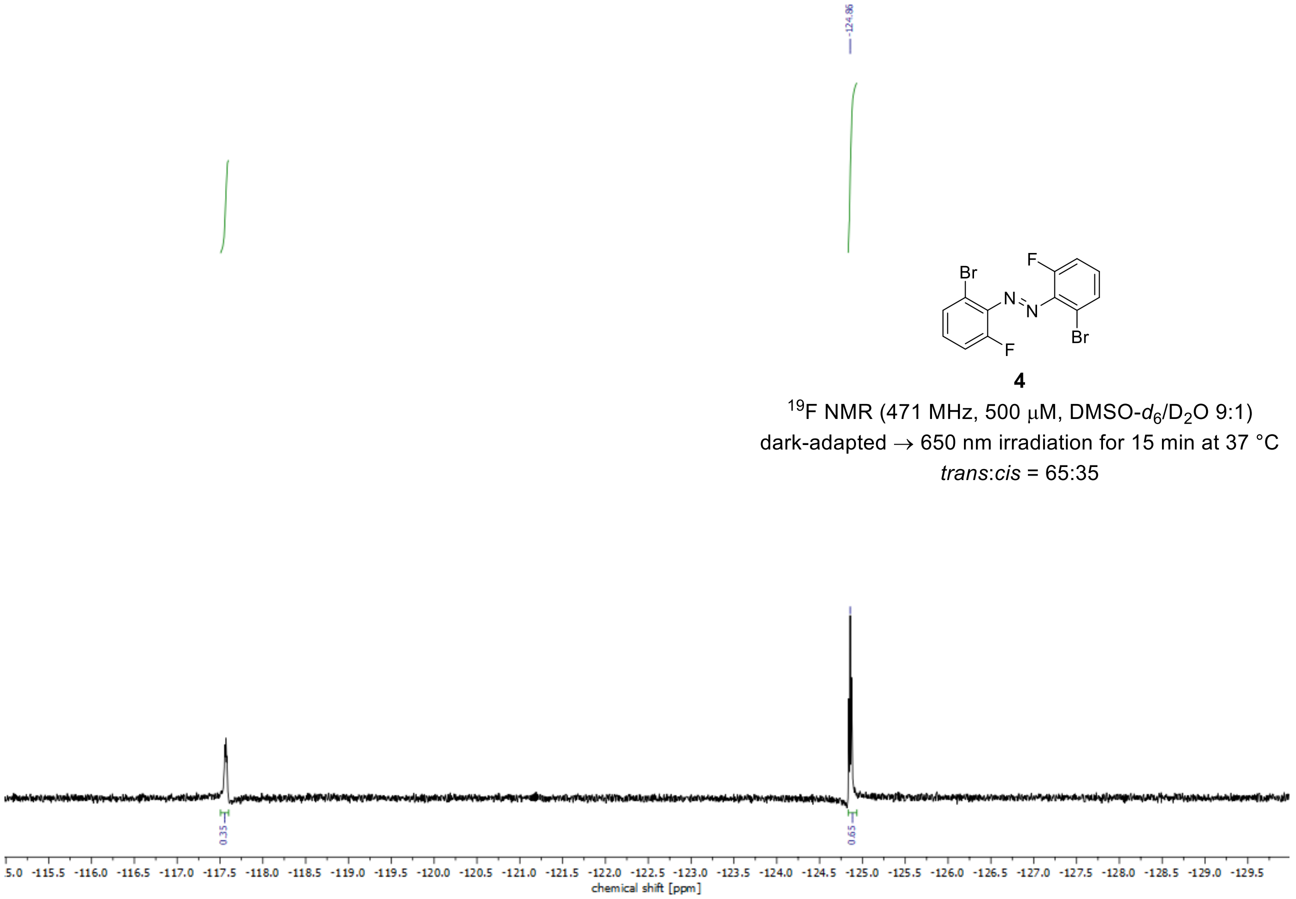

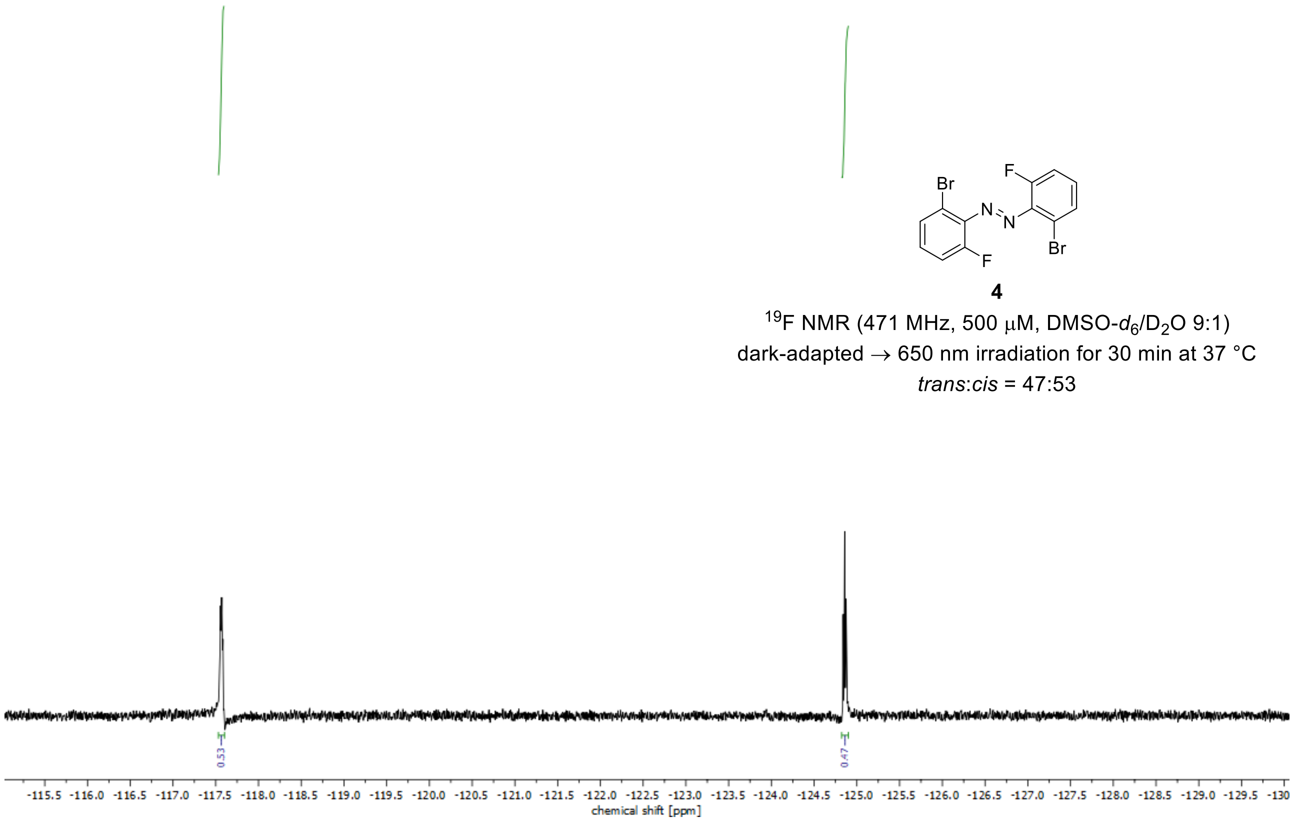
Br
F
N
N
F
Br
4
^{19}F NMR (471 MHz, 500 μM, DMSO-d_6/D_2O 9:1)
dark-adapted → 650 nm irradiation for 30 min at 37 °C
trans:*cis* = 47:53
0.53
0.47
-115.5 -116.0 -116.5 -117.0 -117.5 -118.0 -118.5 -119.0 -119.5 -120.0 -120.5 -121.0 -121.5 -122.0 -122.5 -123.0 -123.5 -124.0 -124.5 -125.0 -125.5 -126.0 -126.5 -127.0 -127.5 -128.0 -128.5 -129.0 -129.5 -130
chemical shift [ppm]

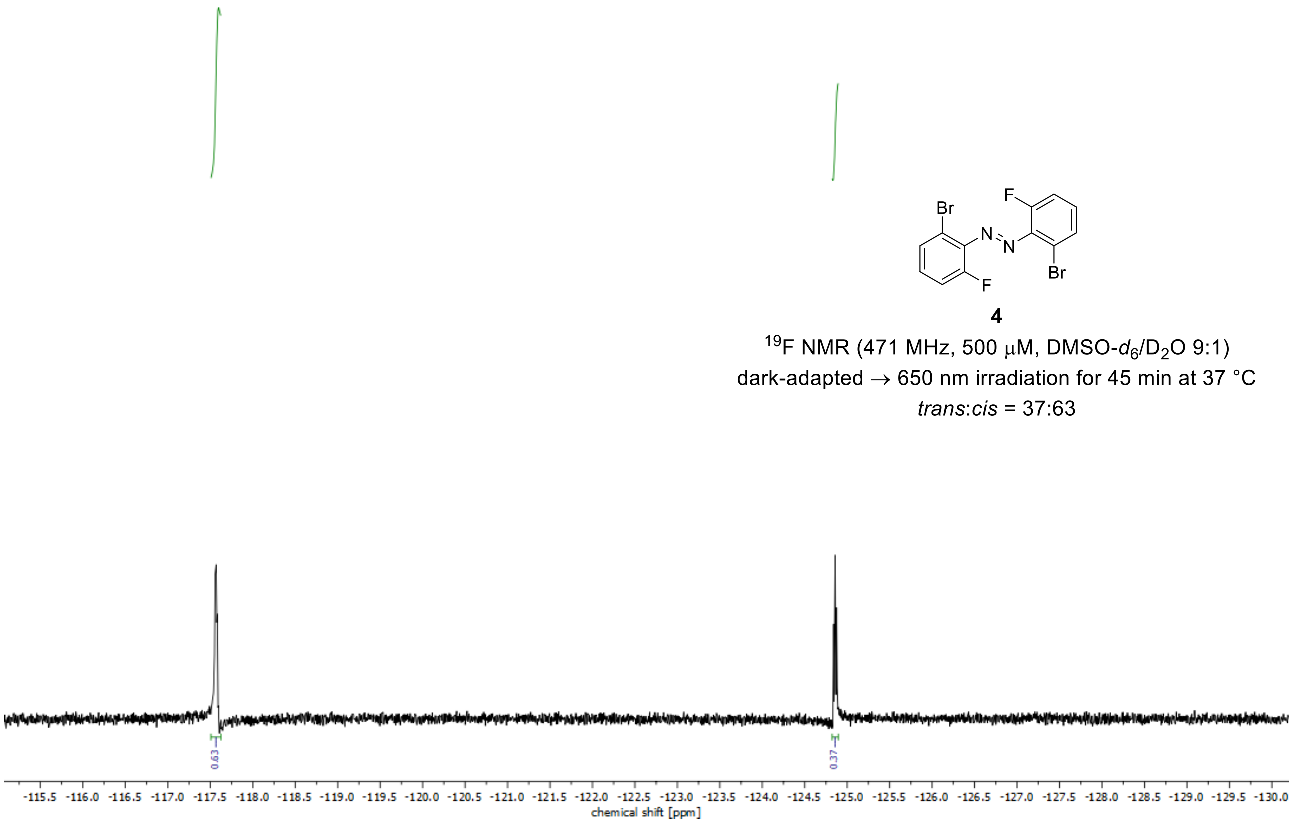
Br
F
N
N
F
Br
4
^{19}F NMR (471 MHz, 500 μM, DMSO-d_6/D_2O 9:1)
dark-adapted → 650 nm irradiation for 45 min at 37 °C
trans:*cis* = 37:63
0.63
0.37
-115.5 -116.0 -116.5 -117.0 -117.5 -118.0 -118.5 -119.0 -119.5 -120.0 -120.5 -121.0 -121.5 -122.0 -122.5 -123.0 -123.5 -124.0 -124.5 -125.0 -125.5 -126.0 -126.5 -127.0 -127.5 -128.0 -128.5 -129.0 -129.5 -130.0
chemical shift [ppm]

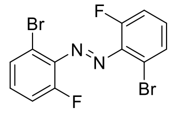

4

^{19}F NMR (471 MHz, 500 μM, DMSO-d_6/D_2O 9:1)
dark-adapted → 650 nm irradiation for 60 min at 37 °C
trans:*cis* = 31:69

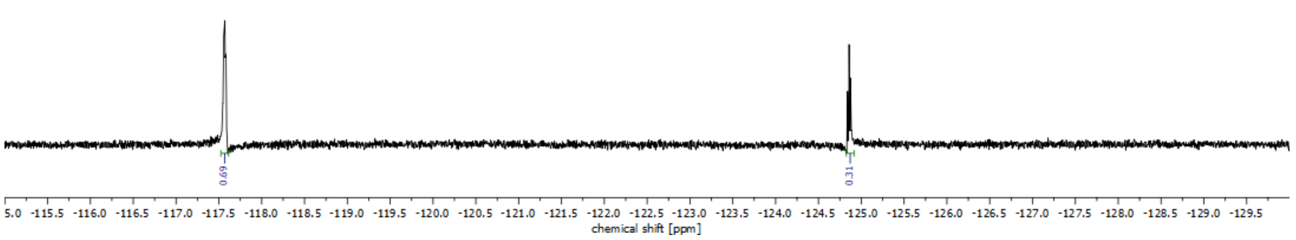

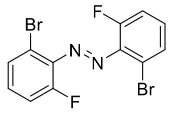

4

^{19}F NMR (471 MHz, 500 μM, DMSO-d_6/D_2O 9:1)
dark-adapted → 650 nm irradiation for 90 min at 37 °C
trans:*cis* = 25:75

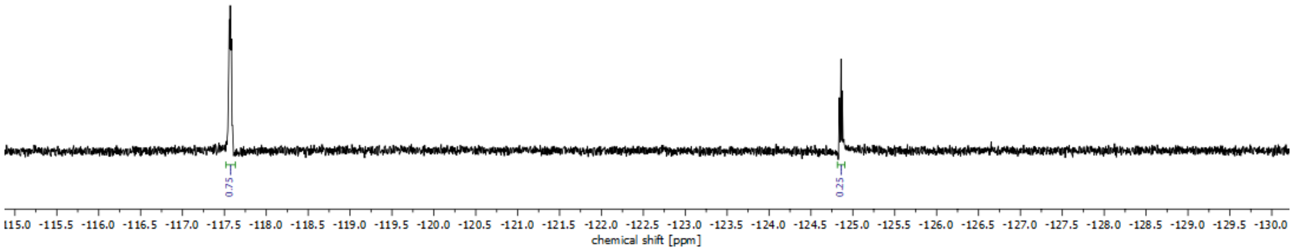

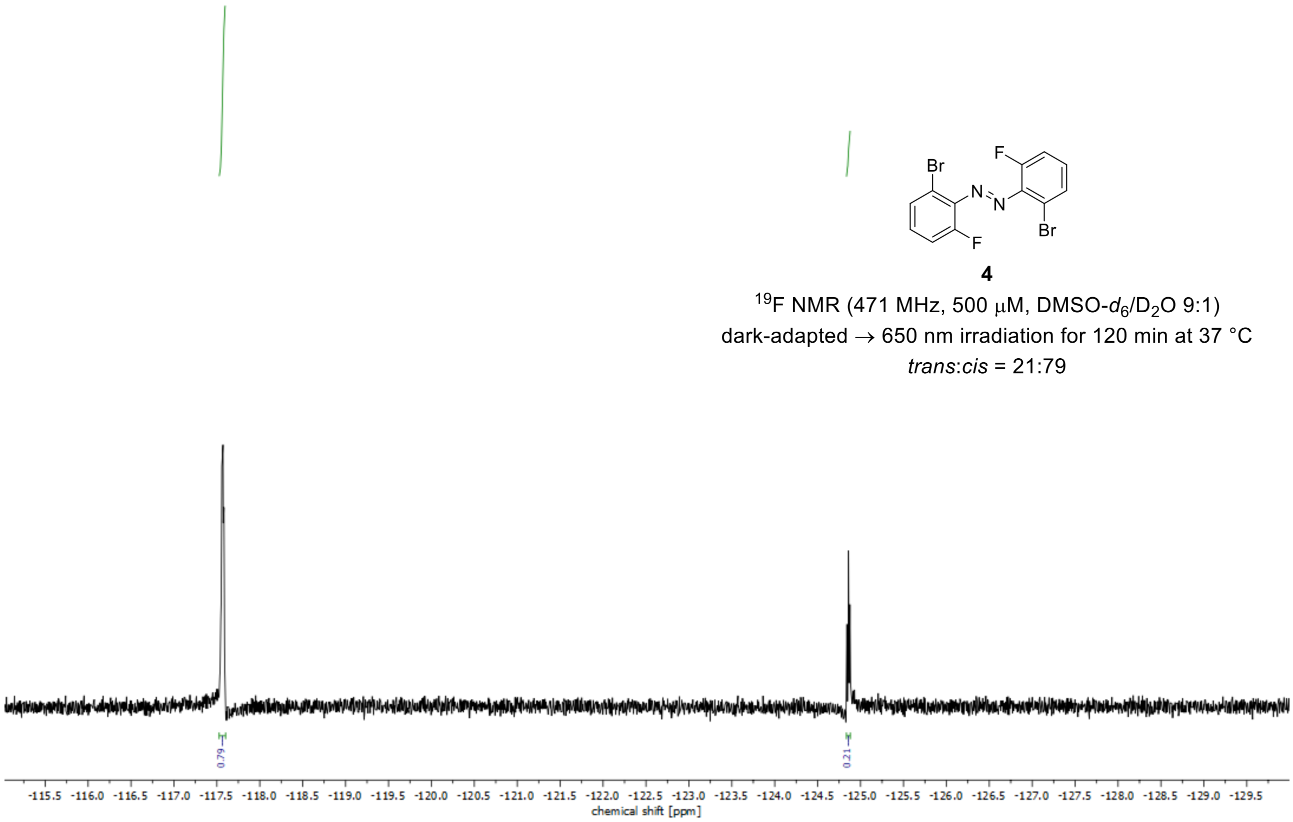

(*E*/*Z*)-1,2-Bis(2-bromo-6-fluorophenyl)diazene (4, 500 μM, DMSO-d_6/D_2O at 50 °C)

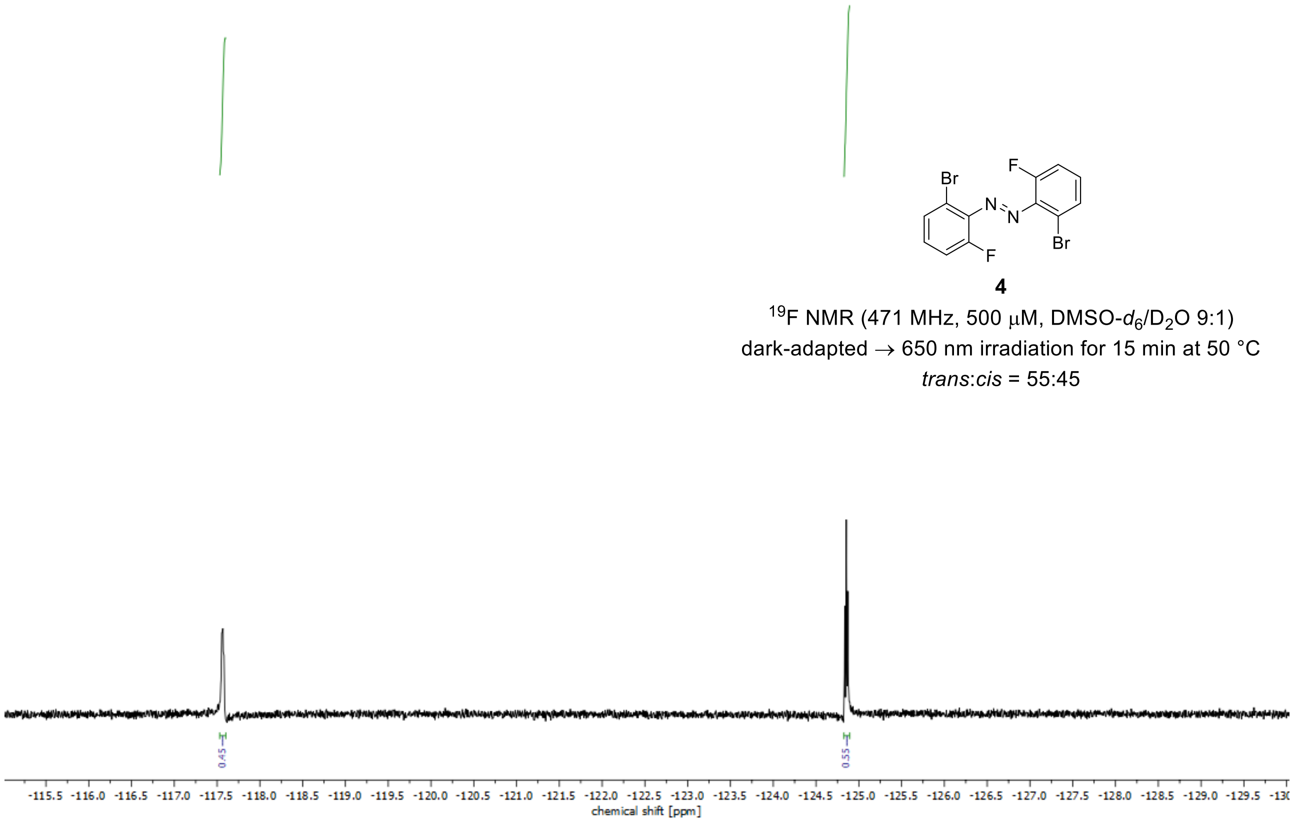

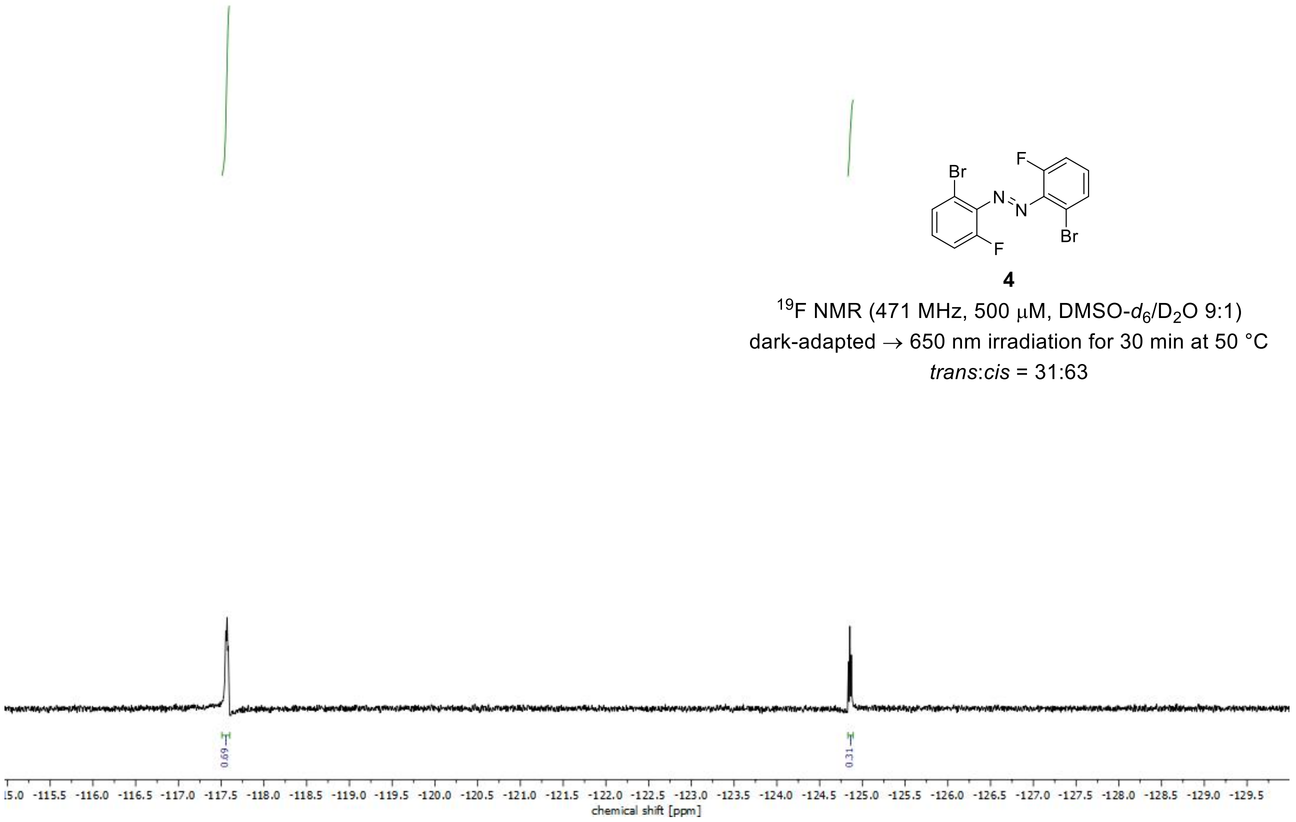

4

^{19}F NMR (471 MHz, 500 μM, DMSO-d_6/D_2O 9:1)
dark-adapted → 650 nm irradiation for 30 min at 50 °C
trans:*cis* = 31:63

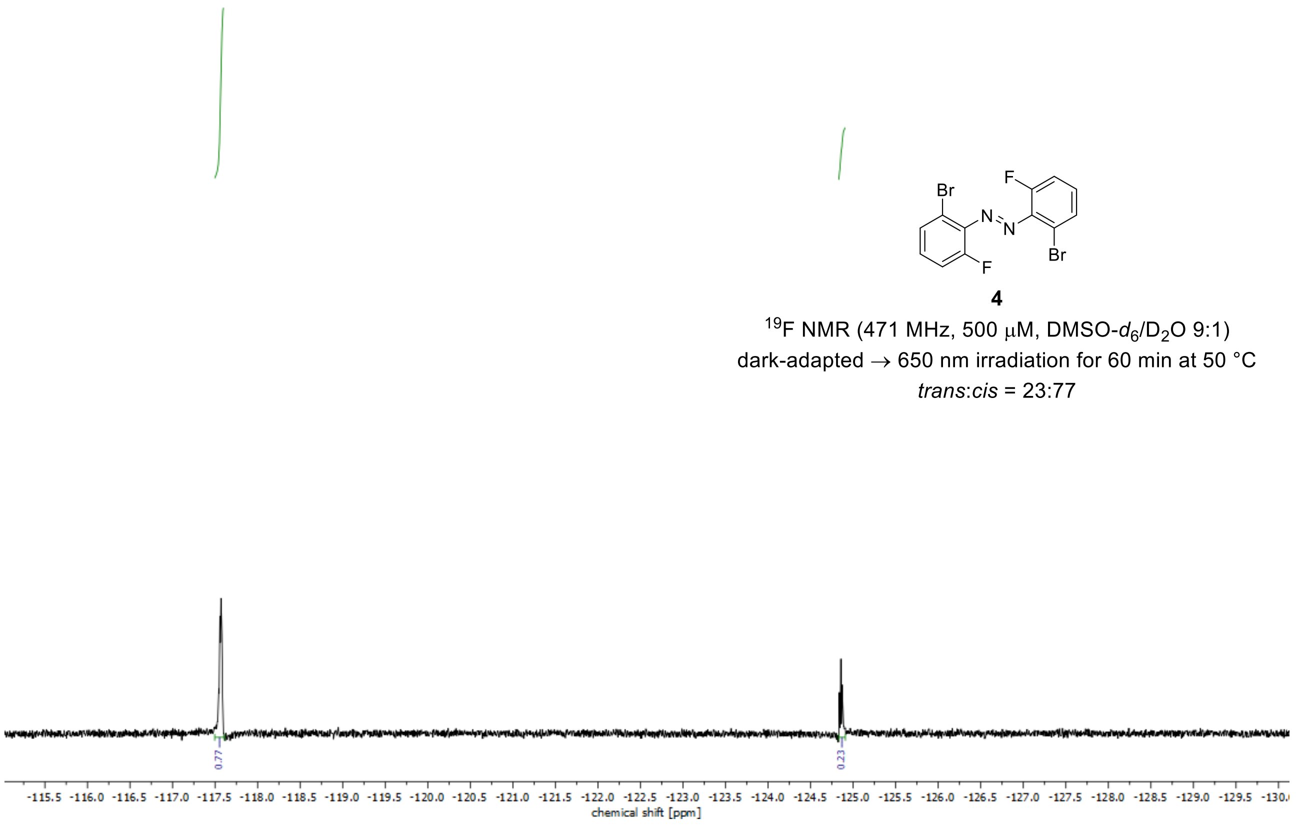

4

^{19}F NMR (471 MHz, 500 μM, DMSO-d_6/D_2O 9:1)
dark-adapted → 650 nm irradiation for 60 min at 50 °C
trans:*cis* = 23:77

(E/Z)-1,2-Bis(2,6-dichlorophenyl)diazene (6, 500 μM, DMSO-d_6/D_2O 9:1)

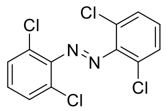

6

^{1}H NMR (500 MHz, 500 μM, DMSO-d_6/D_2O 9:1)

dark-adapted

trans:*cis* = 100:0

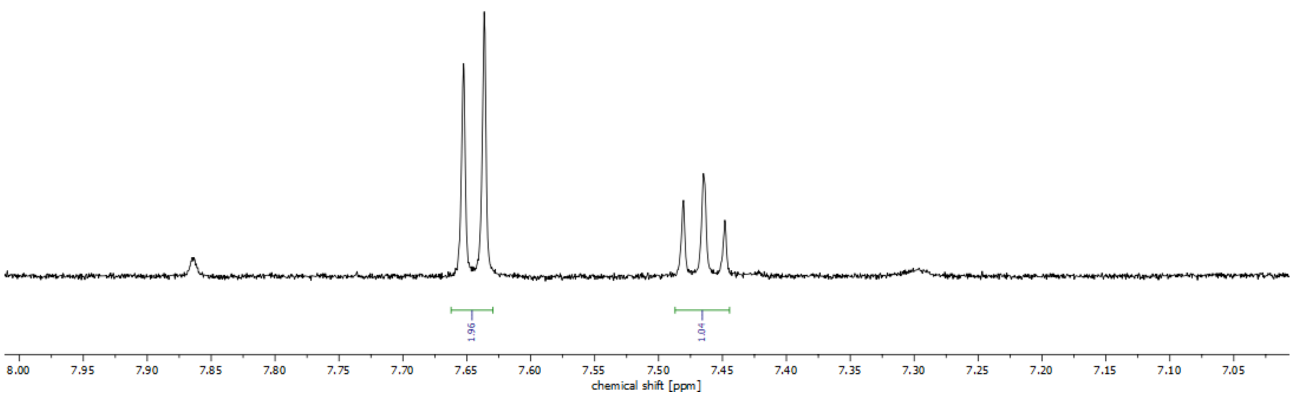

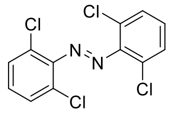

6

^{1}H NMR (500 MHz, 500 μM, DMSO-d_6/D_2O 9:1)

dark-adapted → 650 nm irradiation for 15 min

trans:*cis* = 38:62

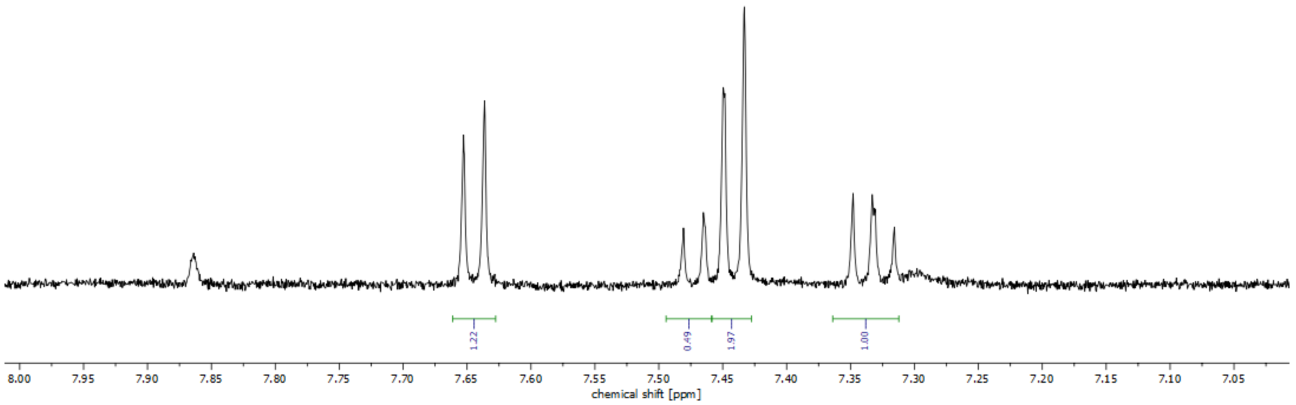

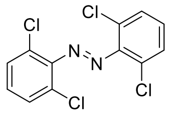

6

^{1}H NMR (500 MHz, 500 μM, DMSO-d_6/D_2O 9:1)
dark-adapted → 650 nm irradiation for 30 min
trans:*cis* = 18:82

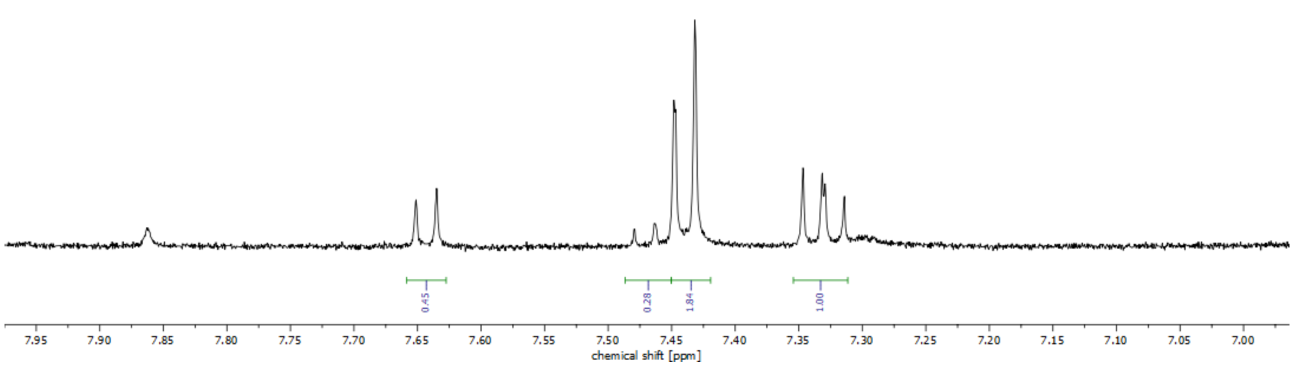

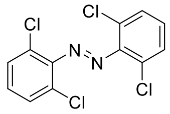

6

^{1}H NMR (500 MHz, 500 μM, DMSO-d_6/D_2O 9:1)
650 nm-adapted → 525 nm irradiation for 15 min
trans:*cis* = 44:56

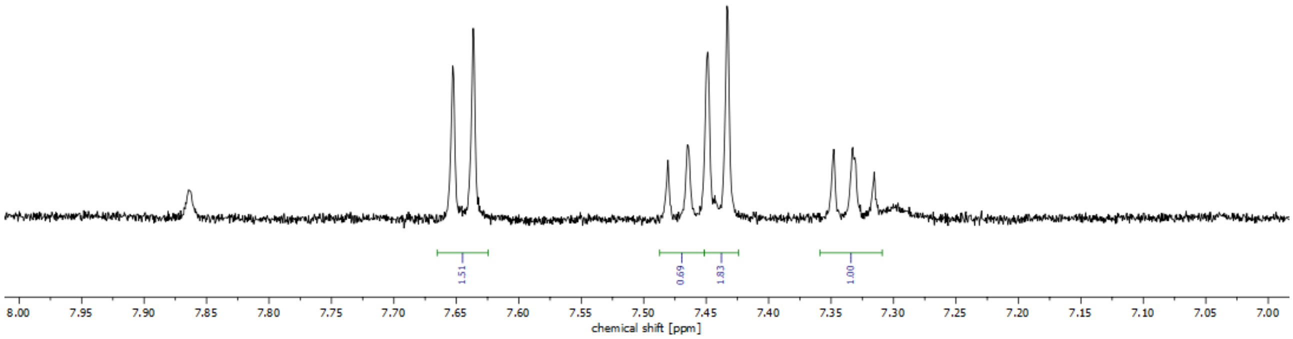

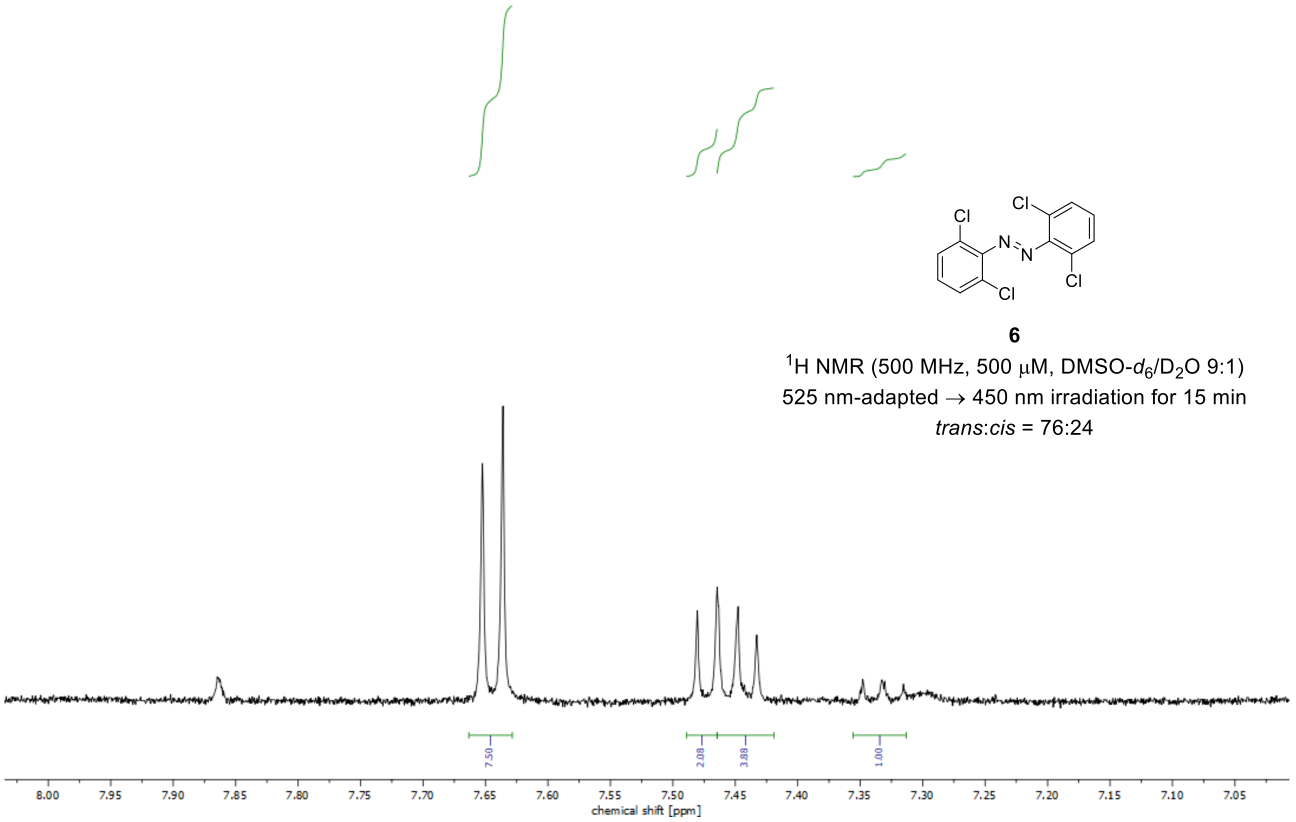

6

^{1}H NMR (500 MHz, 500 μM, DMSO-d_6/D_2O 9:1)

525 nm-adapted → 450 nm irradiation for 15 min

trans:*cis* = 76:24

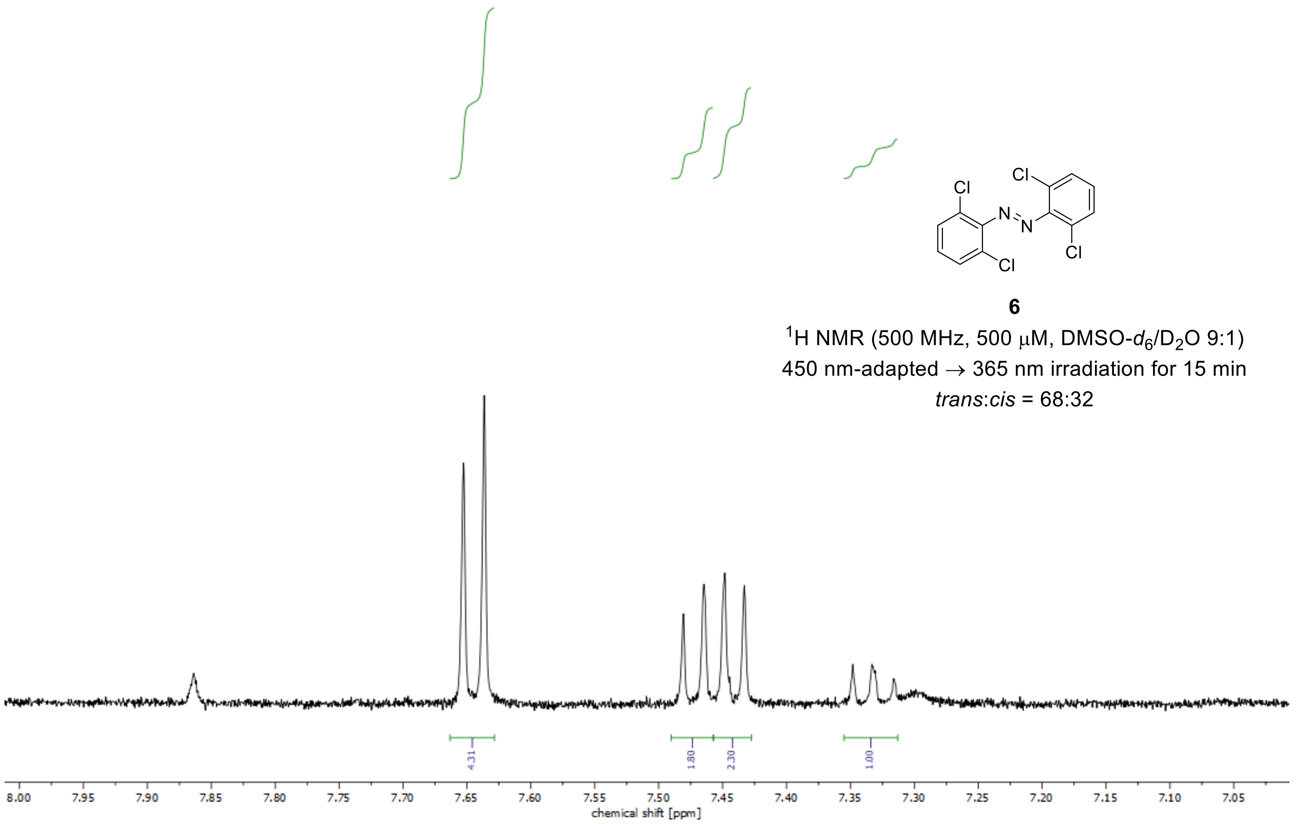

6

^{1}H NMR (500 MHz, 500 μM, DMSO-d_6/D_2O 9:1)

450 nm-adapted → 365 nm irradiation for 15 min

trans:*cis* = 68:32

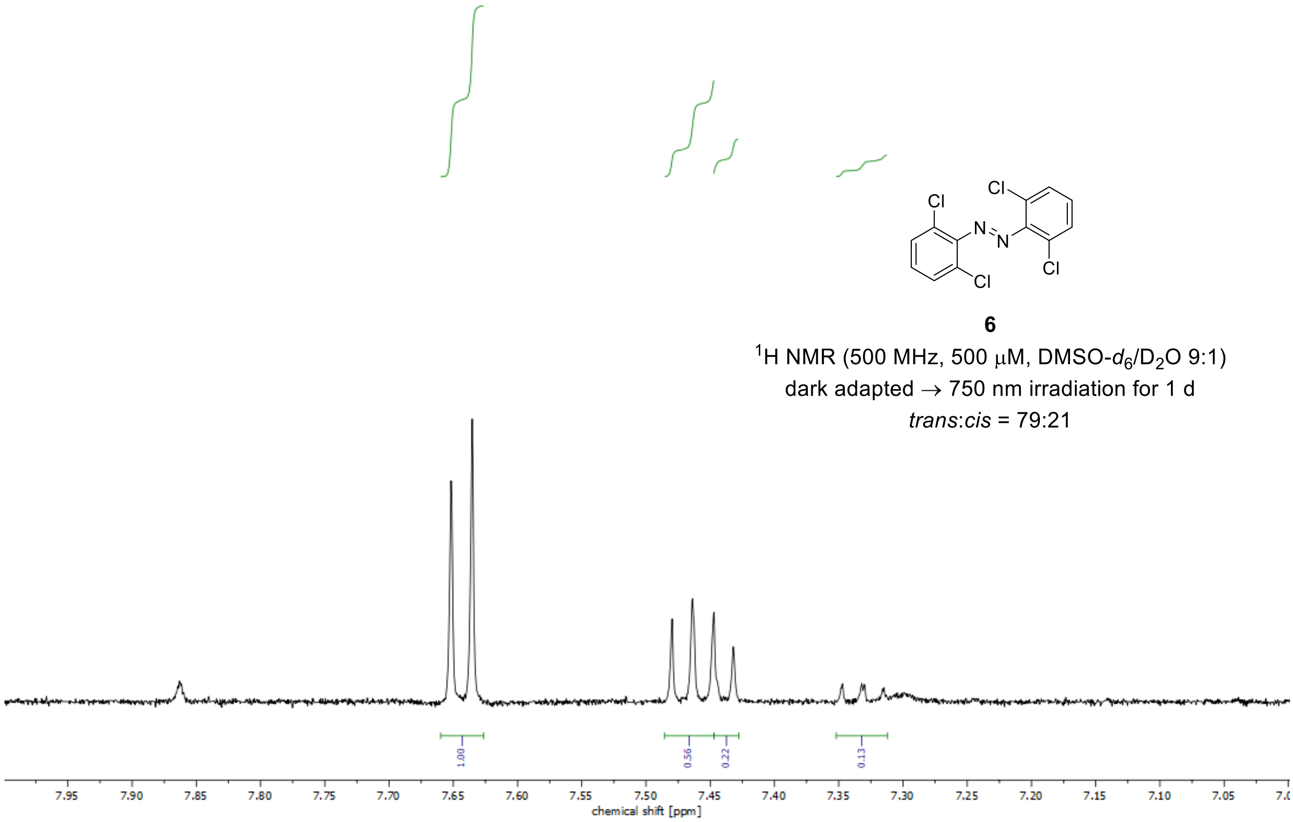

6

^{1}H NMR (500 MHz, 500 μM, DMSO-d_6/D_2O 9:1)
dark adapted → 750 nm irradiation for 1 d
trans:*cis* = 79:21

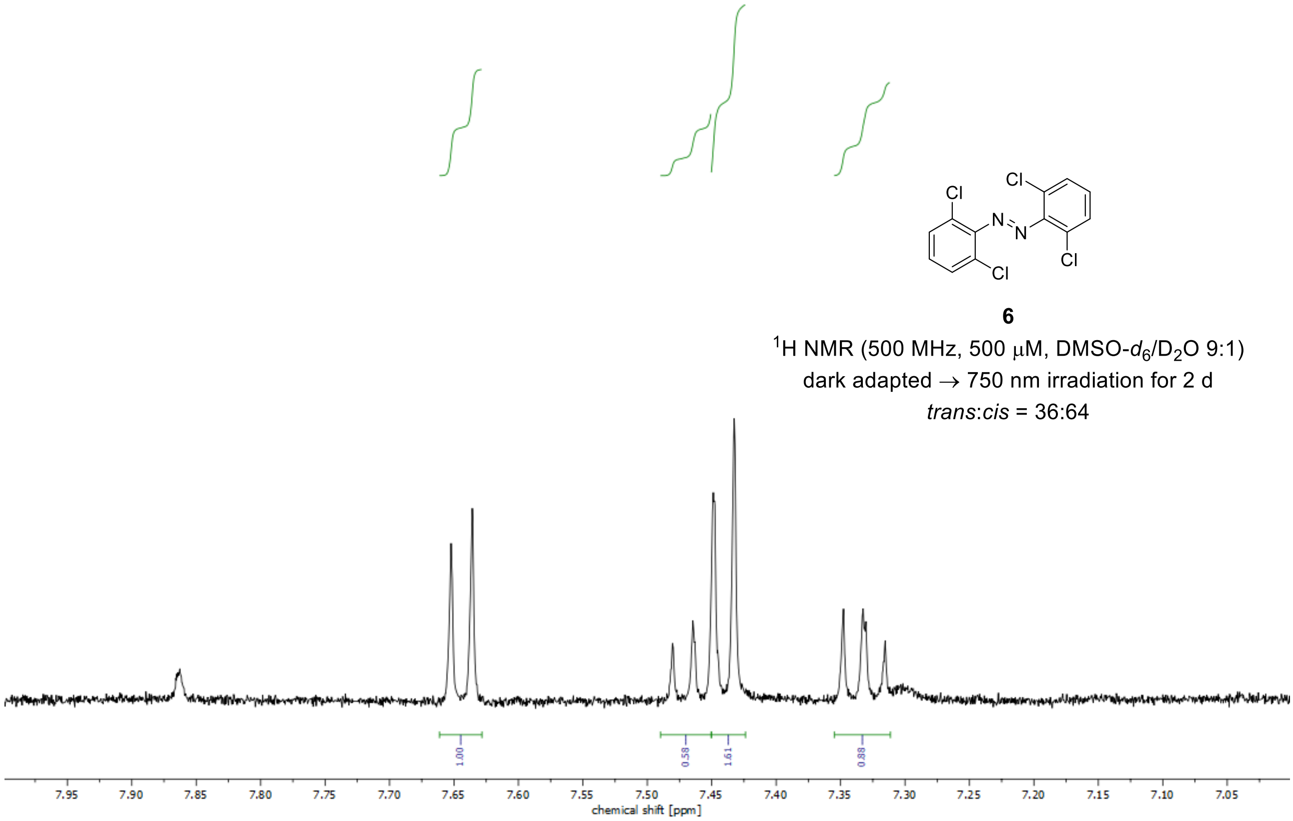

6

^{1}H NMR (500 MHz, 500 μM, DMSO-d_6/D_2O 9:1)
dark adapted → 750 nm irradiation for 2 d
trans:*cis* = 36:64

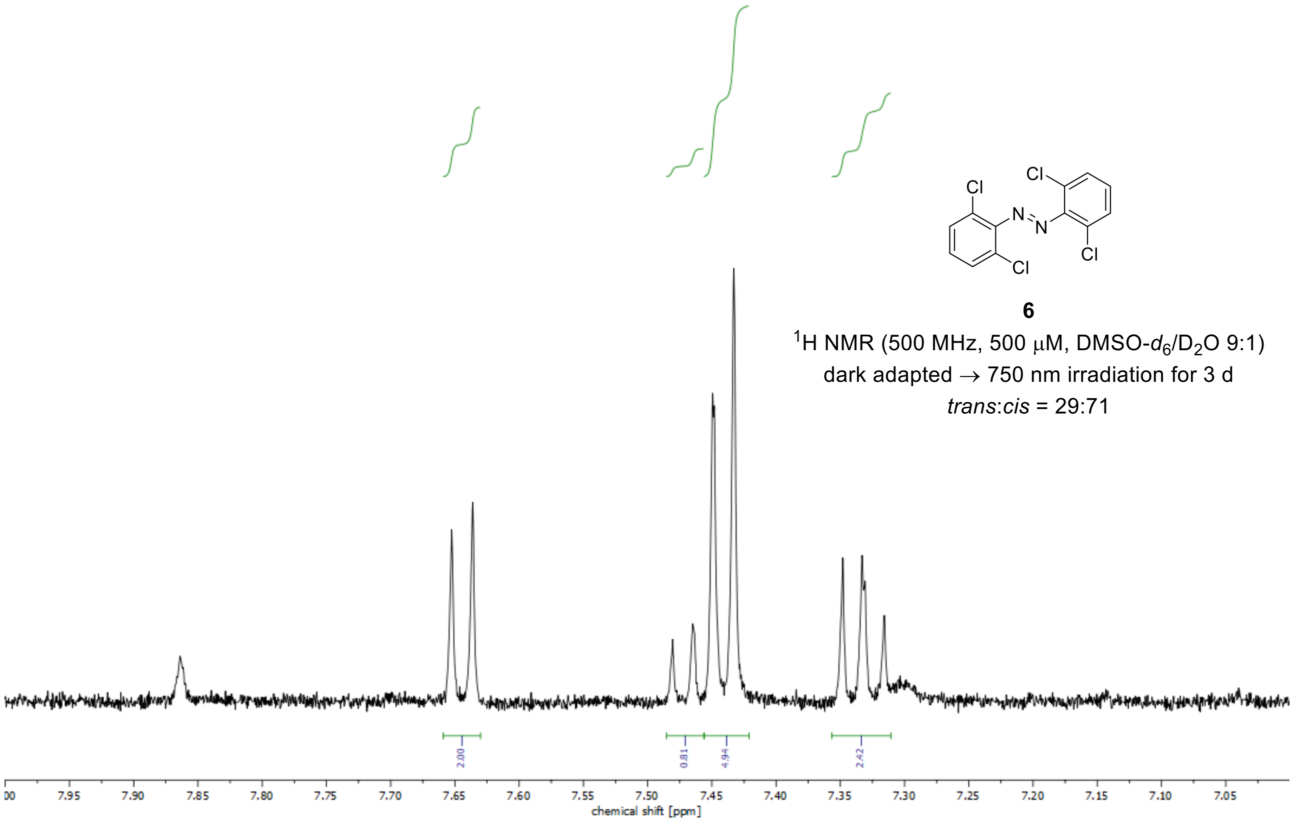

(E/Z)-1,2-Bis(2,6-dimethoxyphenyl)diazene (9, 500 μM, DMSO-d_6/D_2O 9:1)

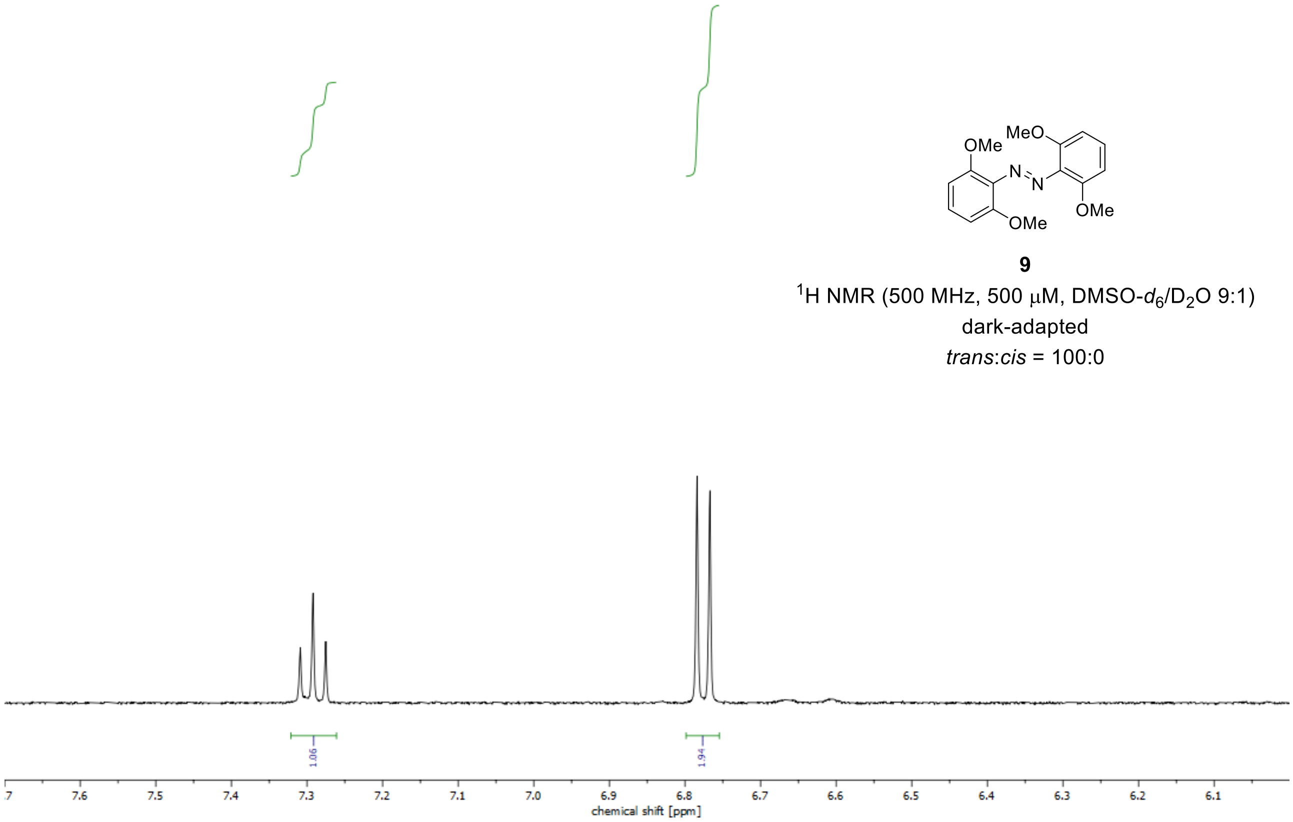

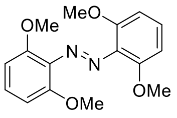

9

^{1}H NMR (500 MHz, 500 μM, DMSO-d_6/D_2O 9:1)
dark-adapted → 650 nm irradiation for 15 min
trans:*cis* = 49:51

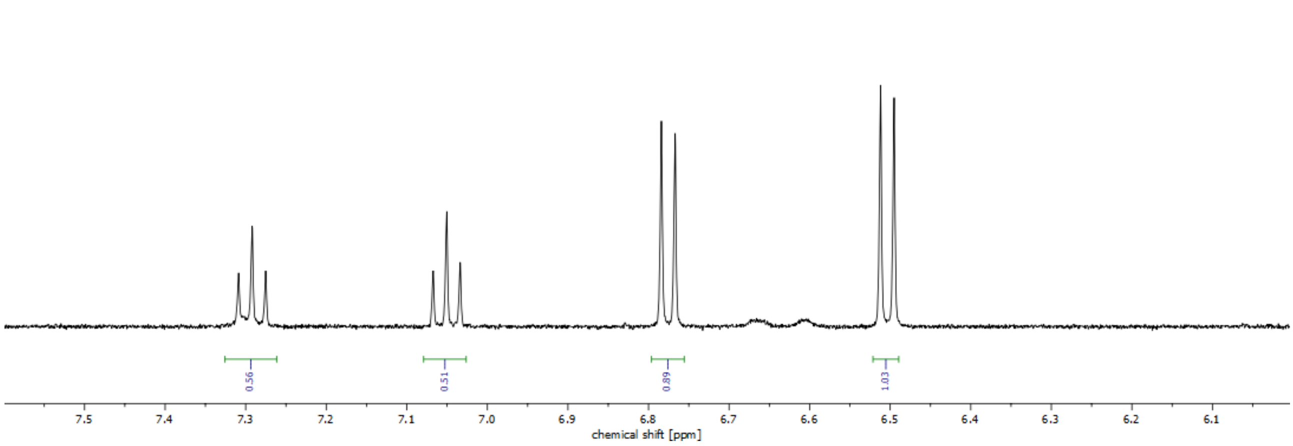

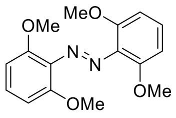

9

^{1}H NMR (500 MHz, 500 μM, DMSO-d_6/D_2O 9:1)
dark-adapted → 650 nm irradiation for 30 min
trans:*cis* = 25:75

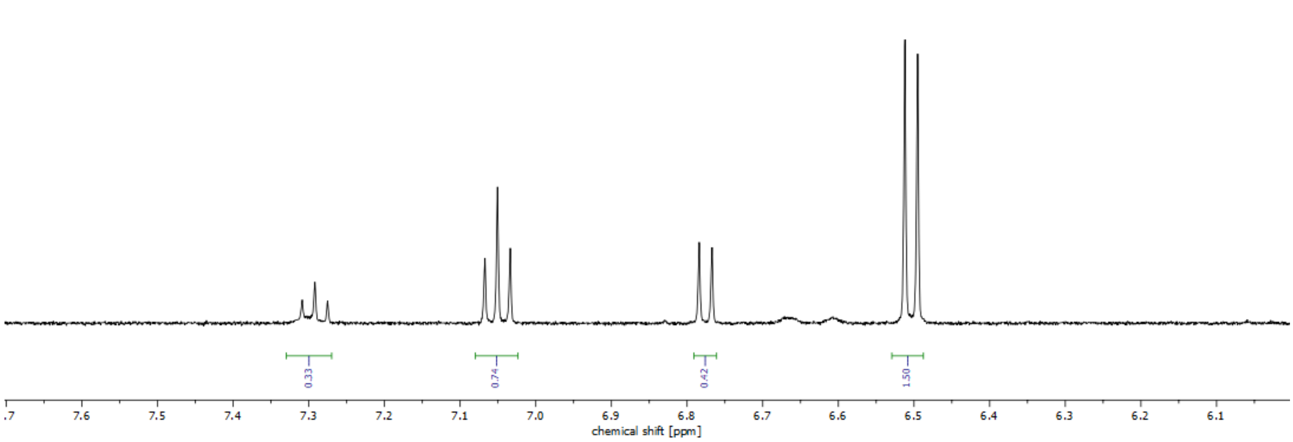

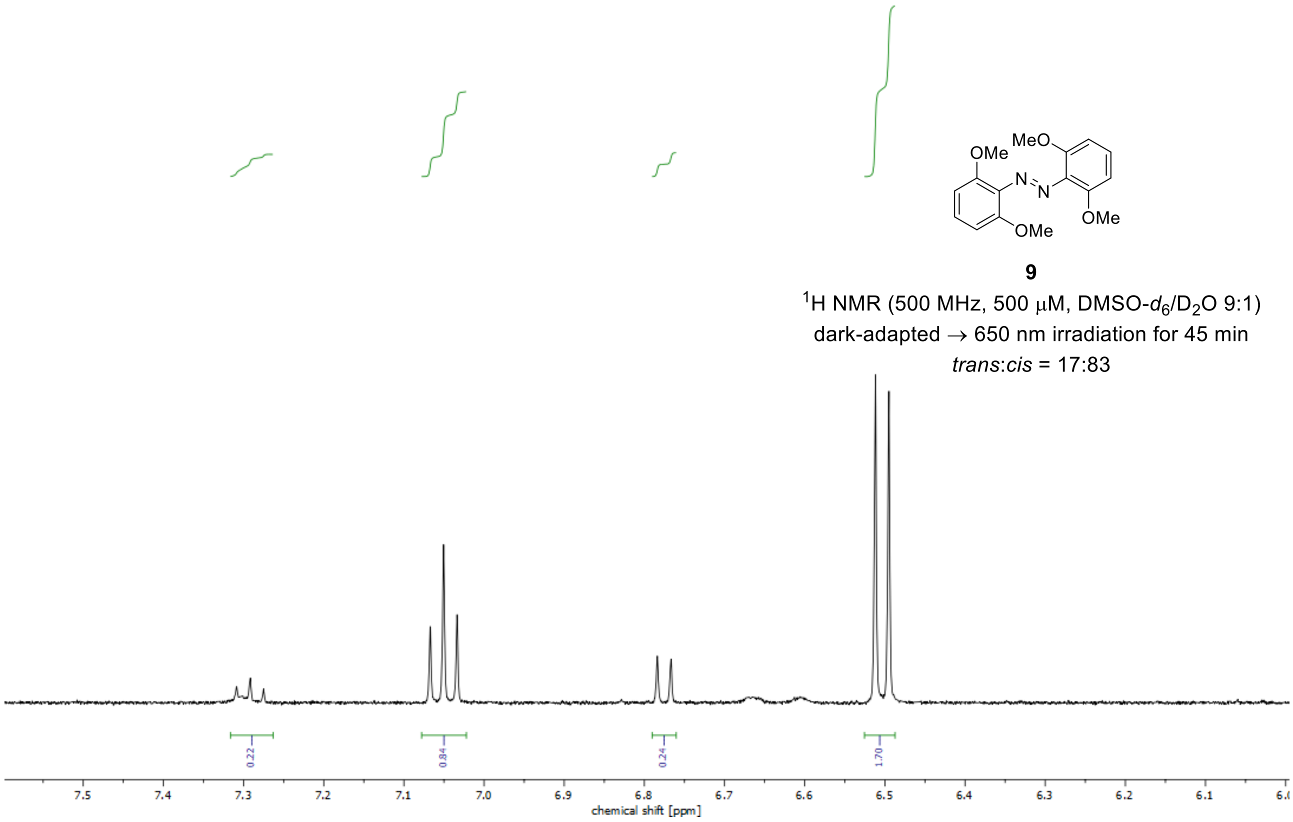

9

^{1}H NMR (500 MHz, 500 µM, DMSO-d_6/D_2O 9:1)

dark-adapted → 650 nm irradiation for 45 min

trans:*cis* = 17:83

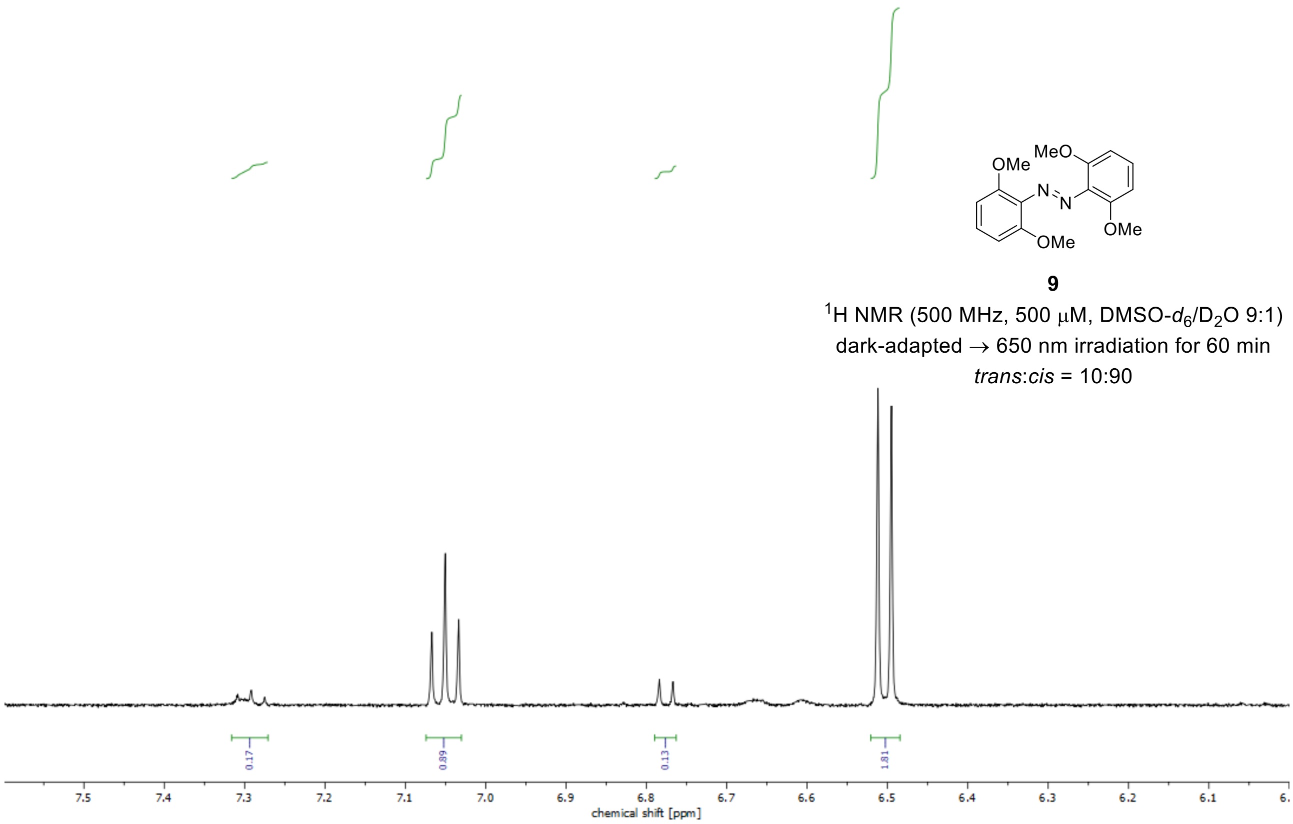

9

^{1}H NMR (500 MHz, 500 µM, DMSO-d_6/D_2O 9:1)

dark-adapted → 650 nm irradiation for 60 min

trans:*cis* = 10:90

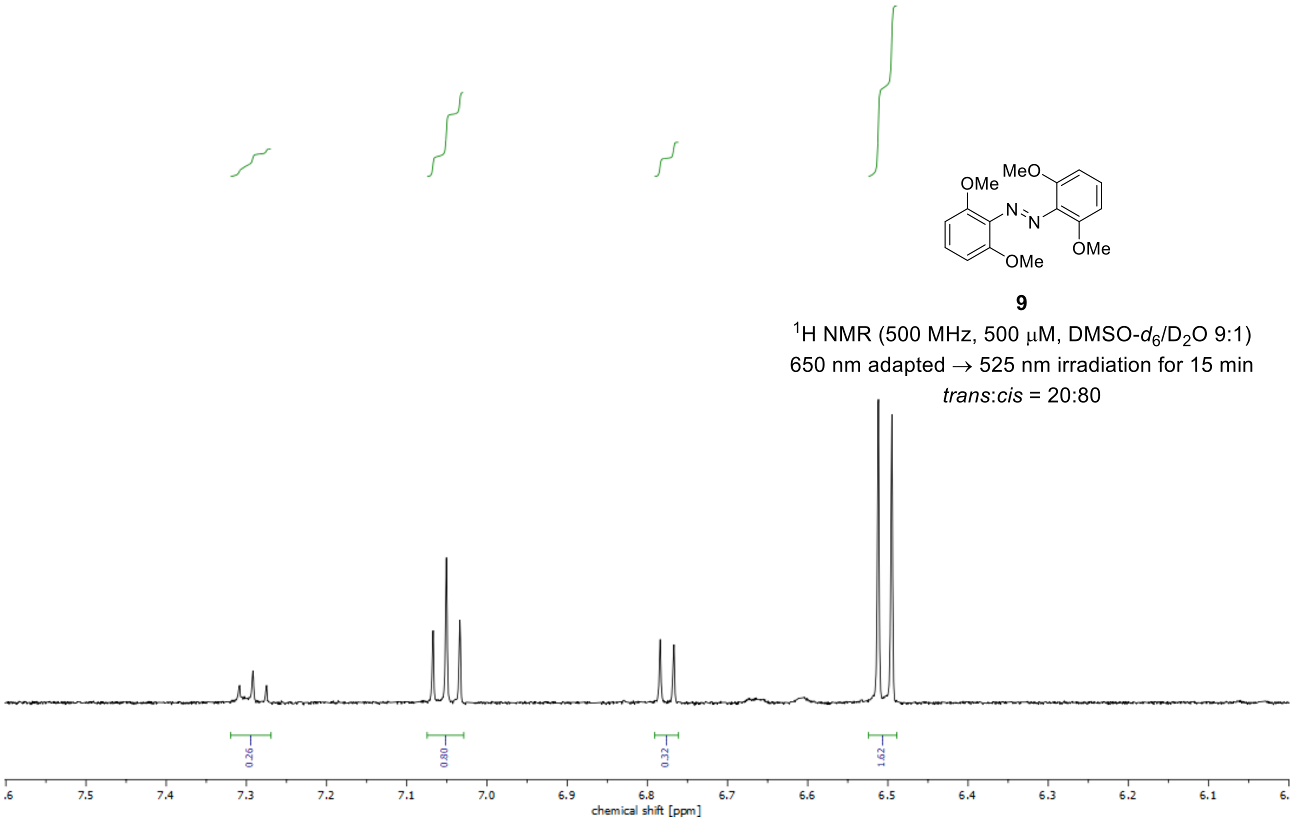

9

^{1}H NMR (500 MHz, 500 μM, DMSO-d_6/D_2O 9:1)
650 nm adapted → 525 nm irradiation for 15 min
trans:*cis* = 20:80

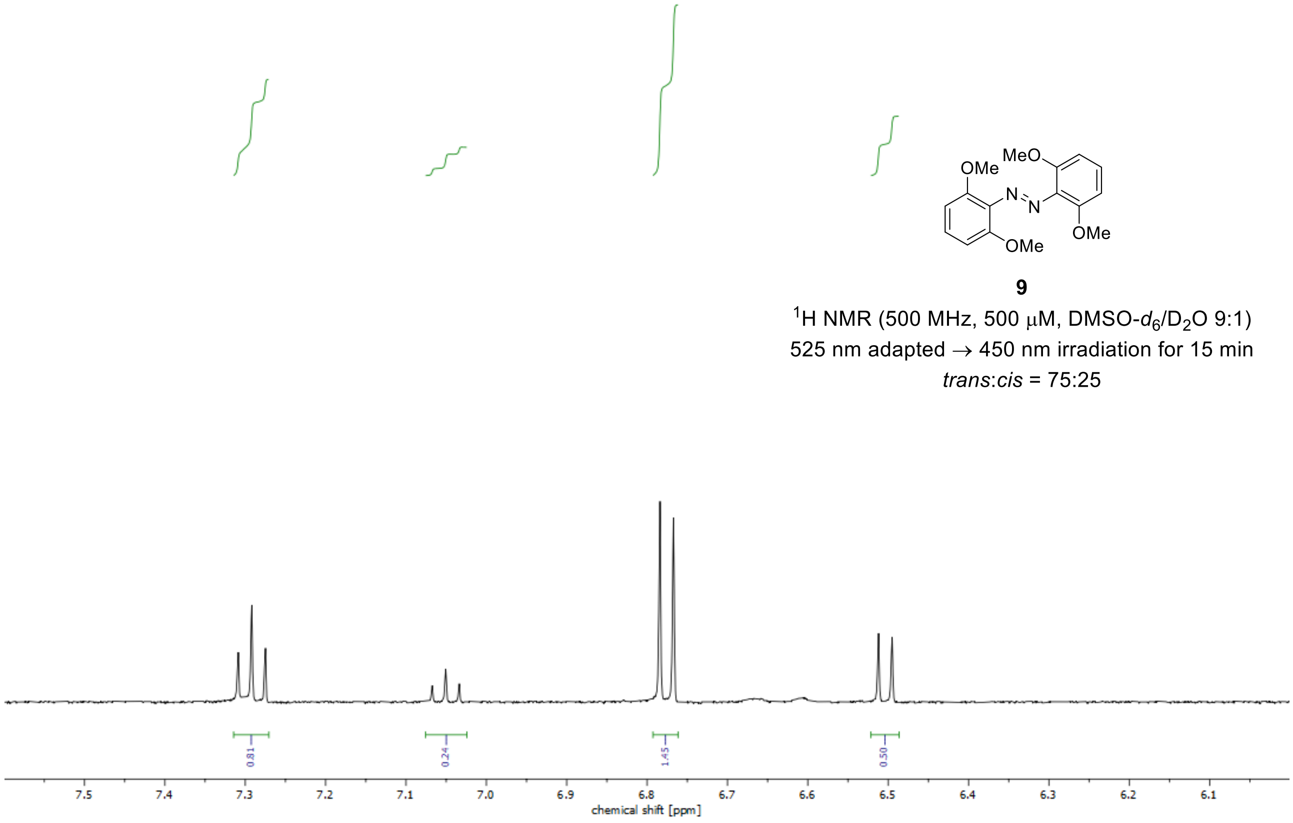

9

^{1}H NMR (500 MHz, 500 μM, DMSO-d_6/D_2O 9:1)
525 nm adapted → 450 nm irradiation for 15 min
trans:*cis* = 75:25

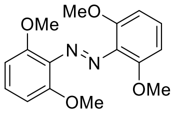

9

^{1}H NMR (500 MHz, 500 μM, DMSO-d_6/D_2O 9:1)

450 nm adapted → 365 nm irradiation for 15 min

trans:*cis* = 68:32

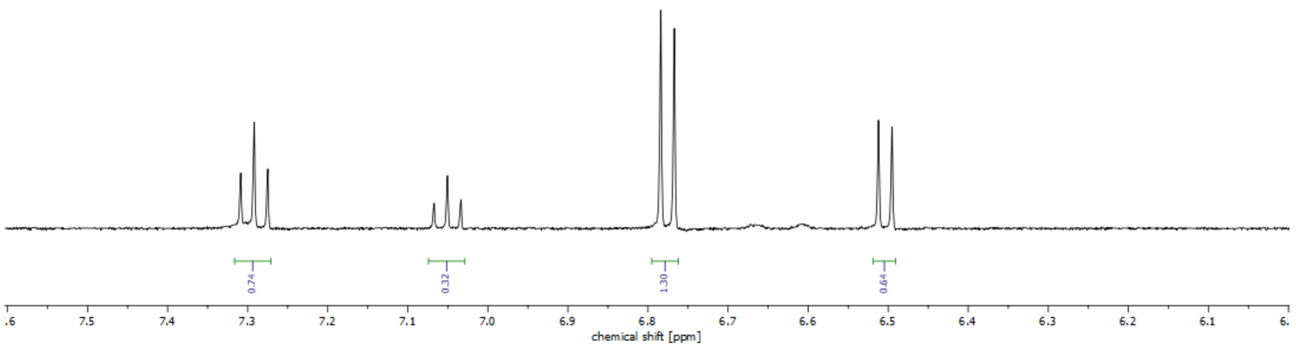

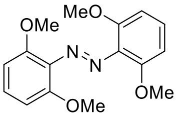

9

^{1}H NMR (500 MHz, 500 μM, DMSO-d_6/D_2O 9:1)

dark-adapted → 740 nm irradiation for 1 d

trans:*cis* = 71:29

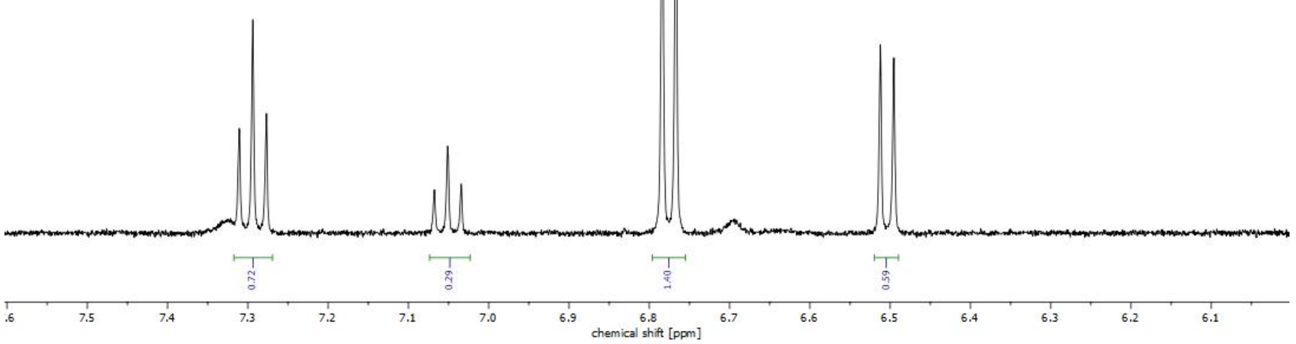

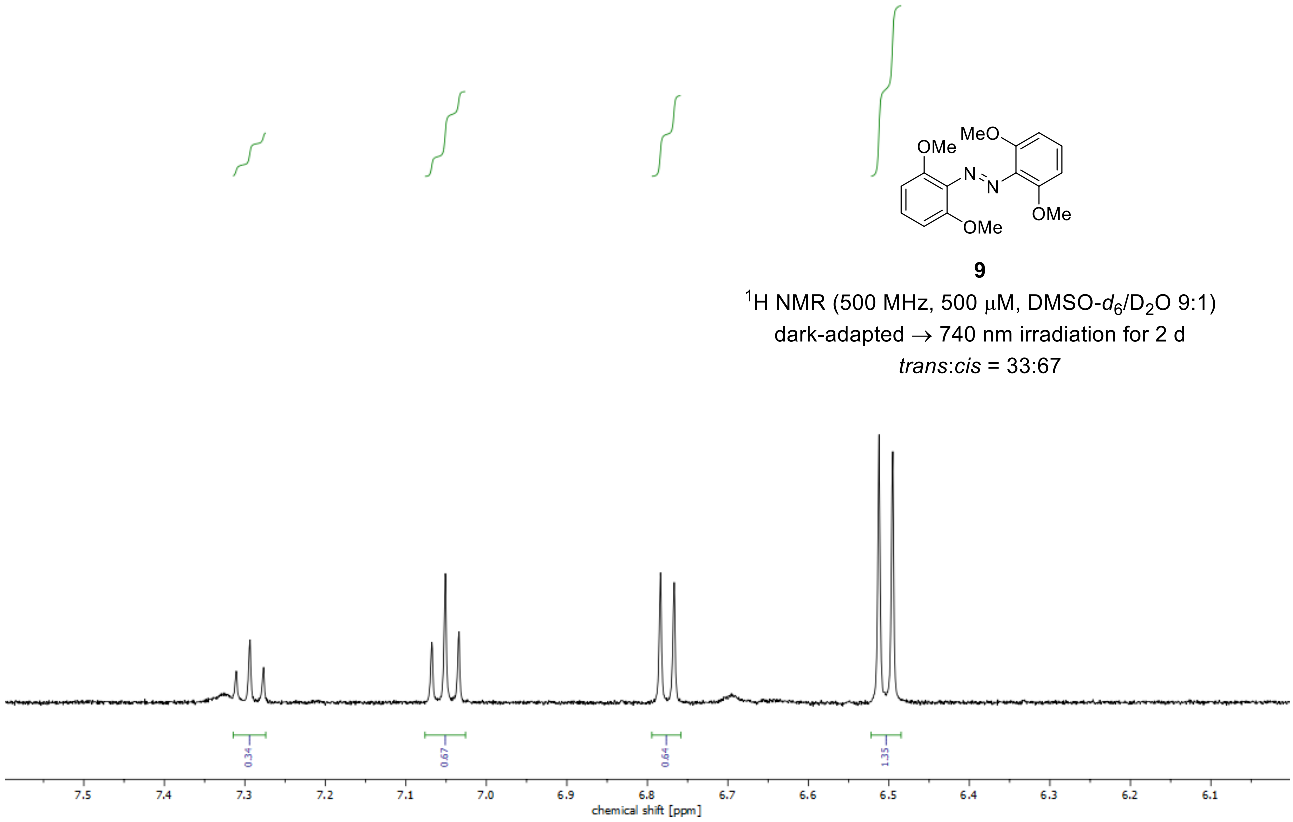

9

^1H NMR (500 MHz, 500 μM, DMSO-d_6/D_2O 9:1)
dark-adapted → 740 nm irradiation for 2 d
trans:*cis* = 33:67

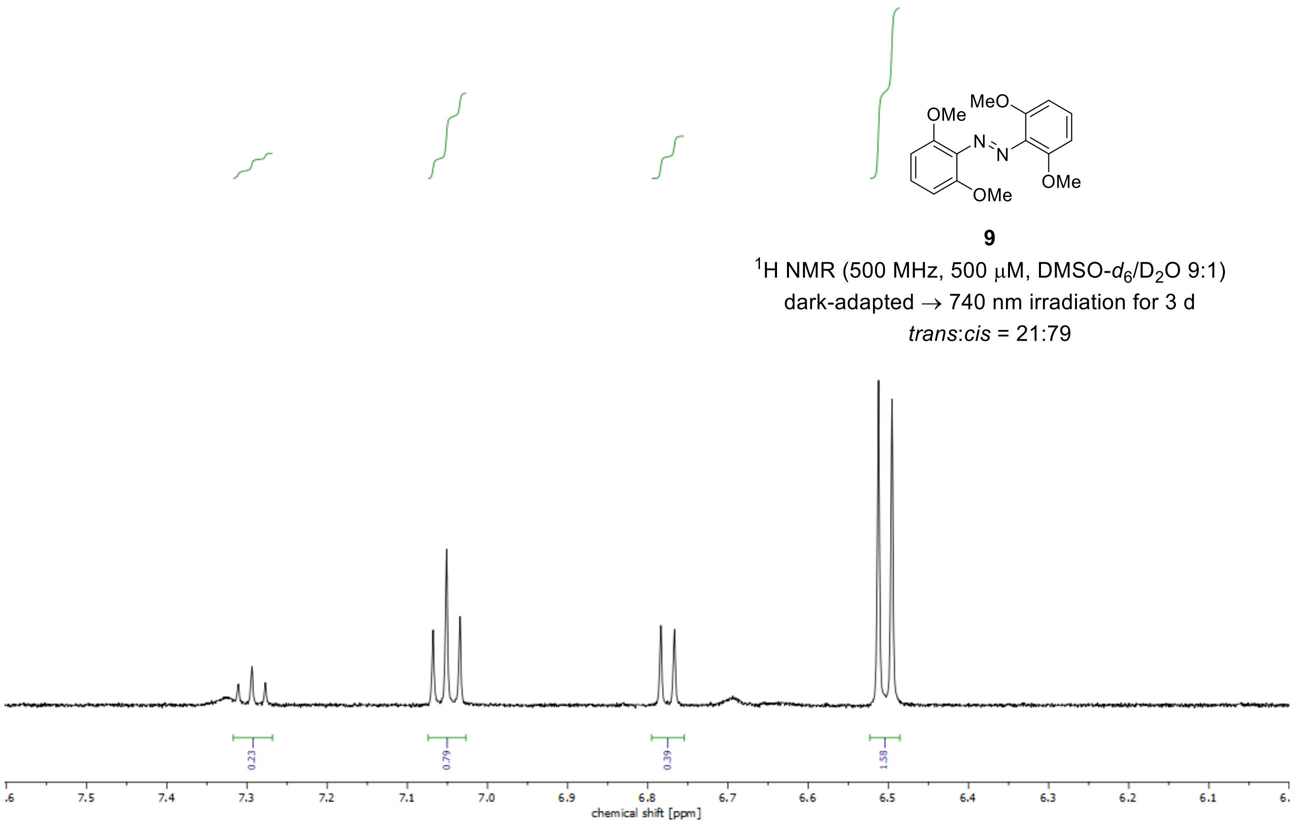

9

^1H NMR (500 MHz, 500 μM, DMSO-d_6/D_2O 9:1)
dark-adapted → 740 nm irradiation for 3 d
trans:*cis* = 21:79

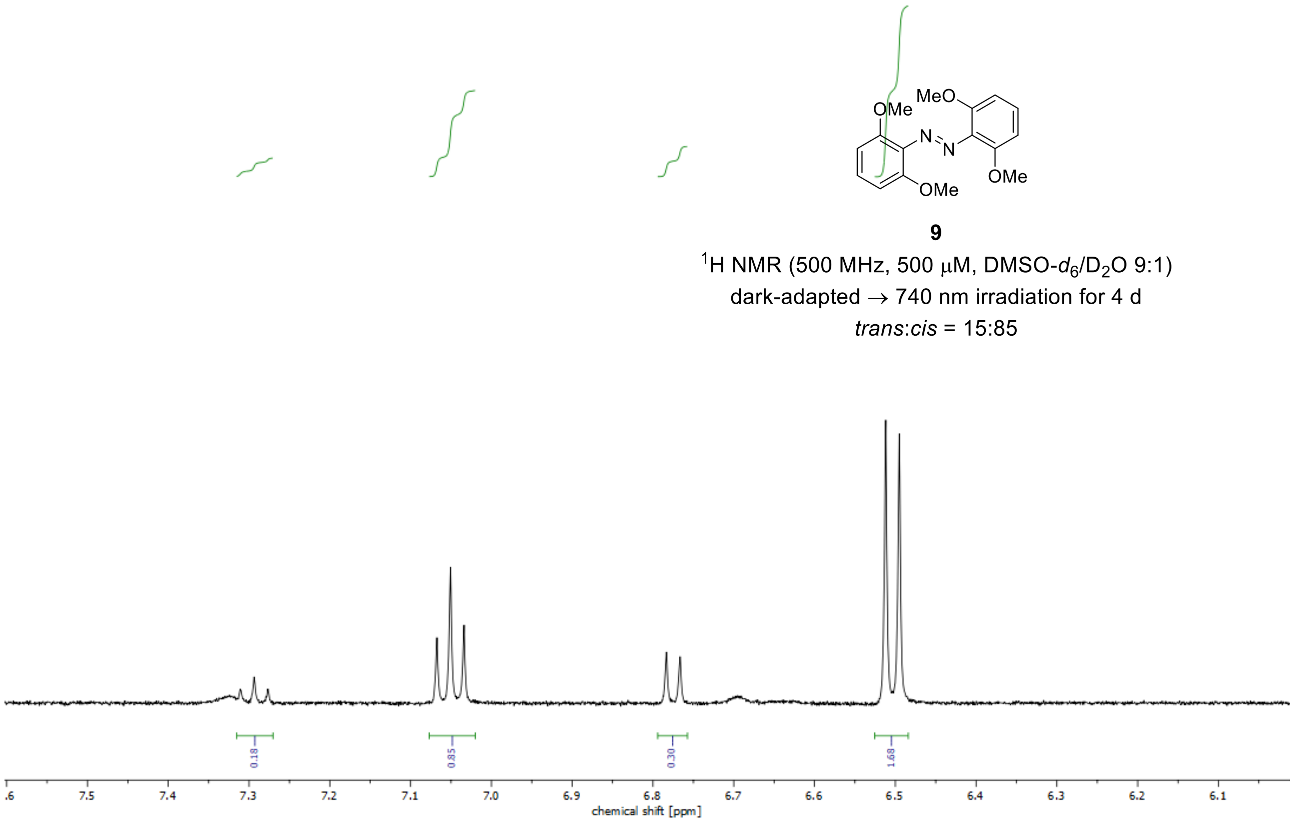

(*E*/*Z*)-1,2-Bis(3-chloro-1-fluoronaphthalen-2-yl)diazene (12, 500 μM, DMSO-d_6/D_2O 9:1)

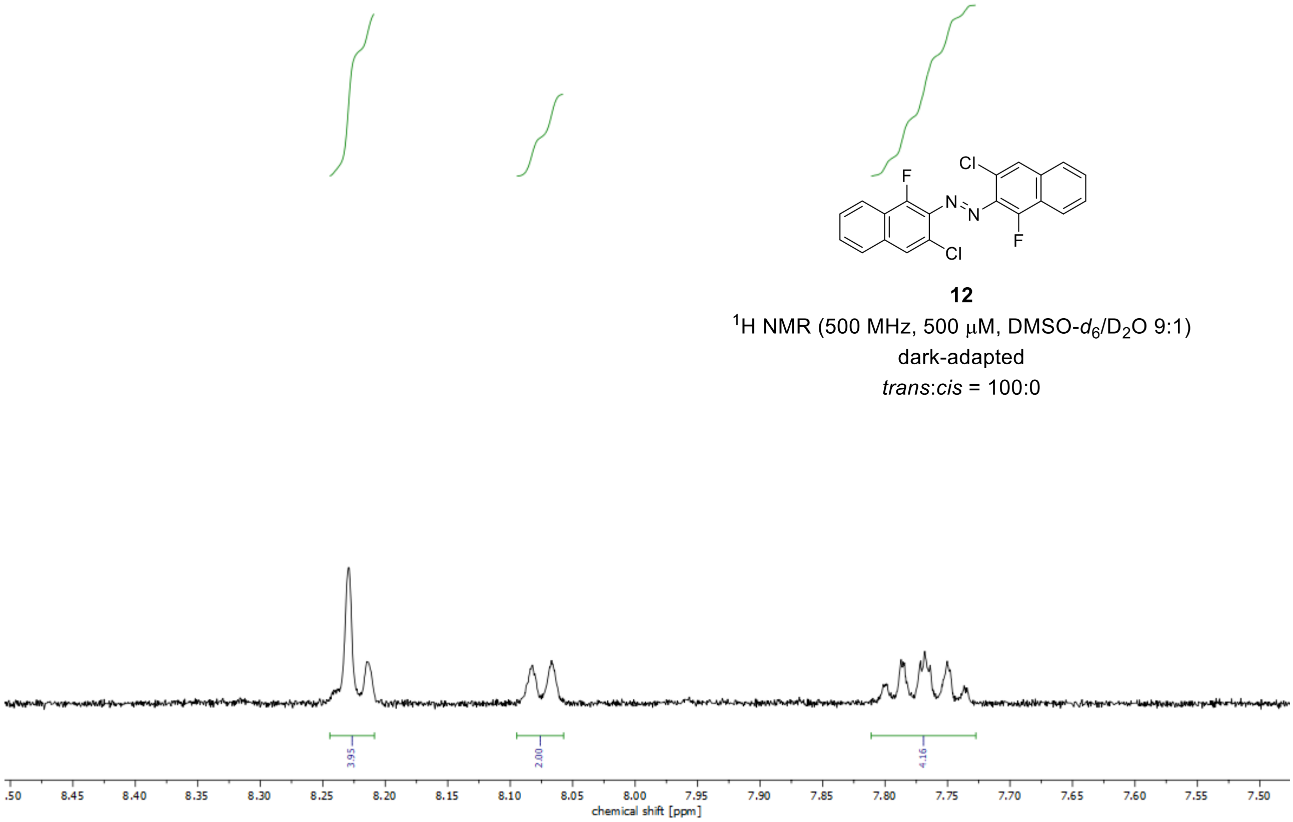

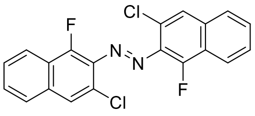

12

^{19}F NMR (471 MHz, 500 μM, DMSO-d_6/D_2O 9:1)
dark-adapted
trans:*cis* = 100:0

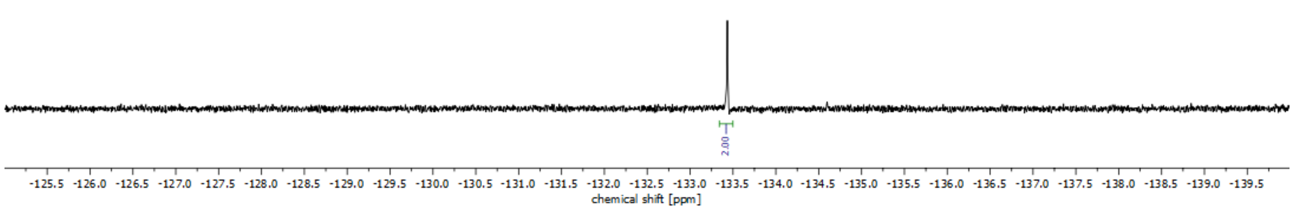

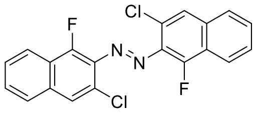

12

^{19}F NMR (471 MHz, 500 μM, DMSO-d_6/D_2O 9:1)
dark-adapted → 650 nm irradiation for 15 min
trans:*cis* = 10:90

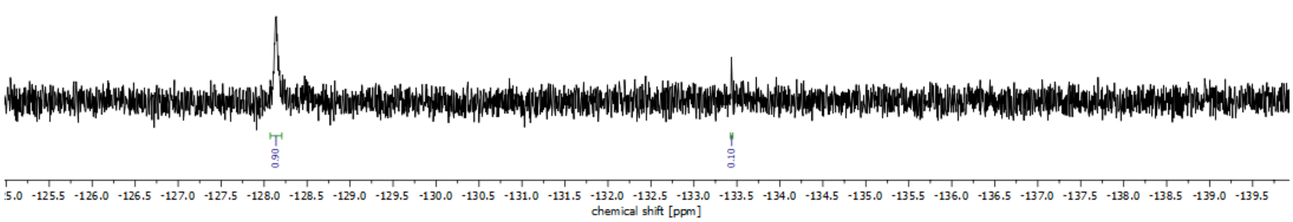

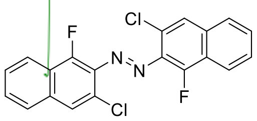

12

^{19}F NMR (471 MHz, 500 μM, DMSO-d_6/D_2O 9:1)
650 nm adapted → 525 nm irradiation for 15 min
trans:*cis* = 45:55

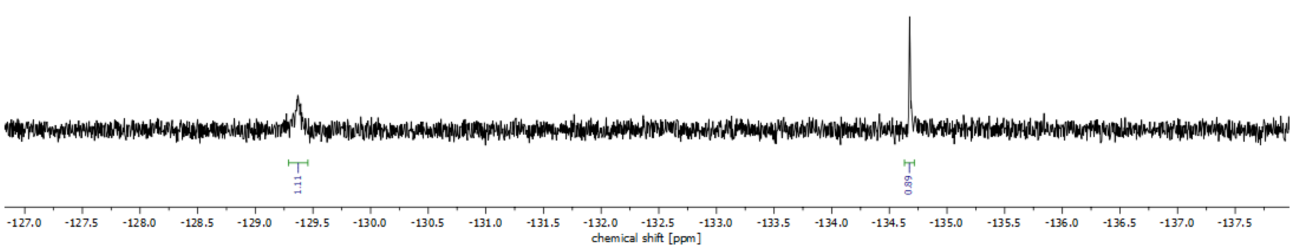

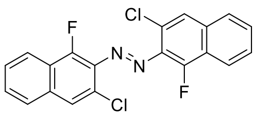

12

^{19}F NMR (471 MHz, 500 μM, DMSO-d_6/D_2O 9:1)
525 nm adapted → 450 nm irradiation for 15 min
trans:*cis* = 100:0

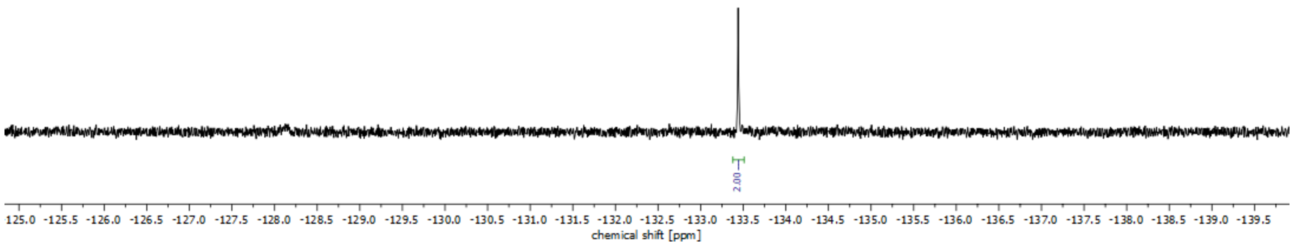

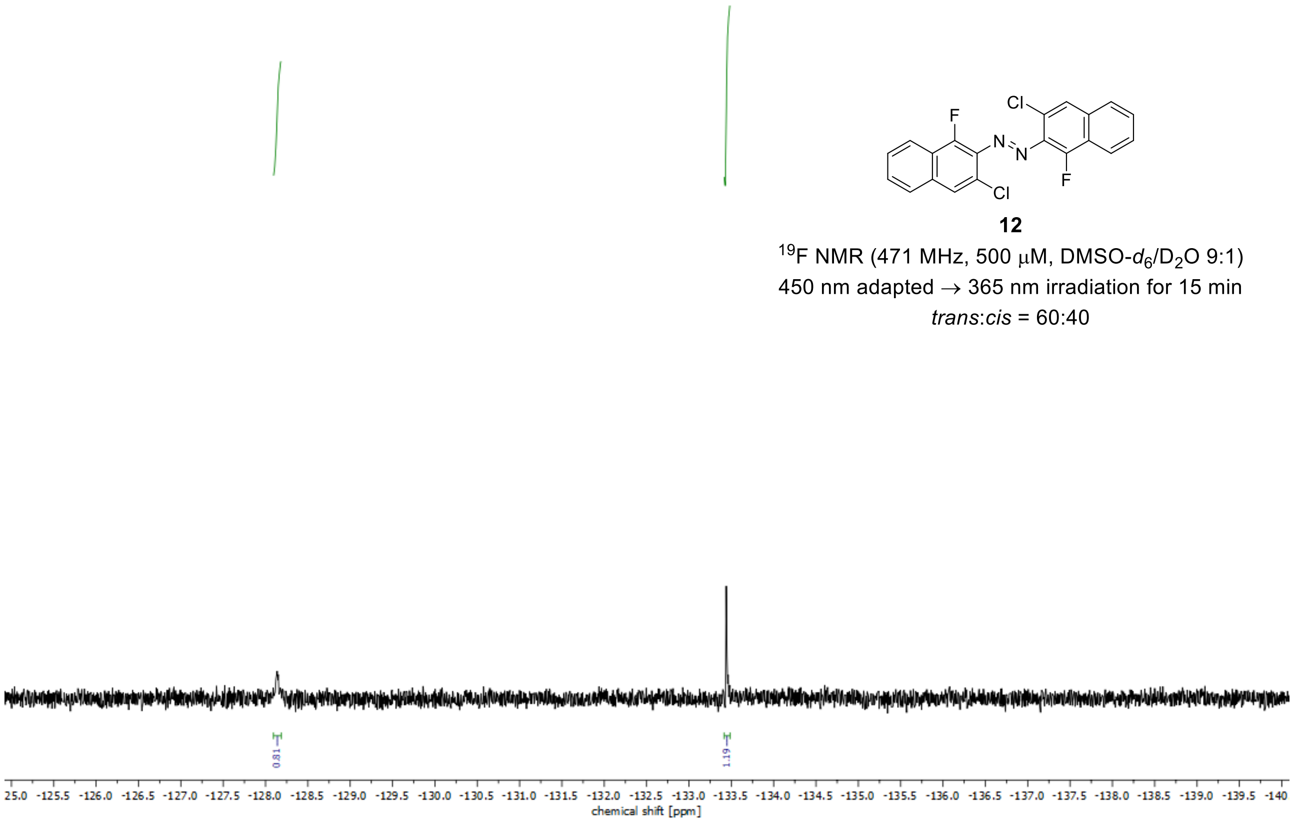

12

^{19}F NMR (471 MHz, 500 μM, DMSO-d_6/D_2O 9:1)

450 nm adapted → 365 nm irradiation for 15 min

trans:*cis* = 60:40

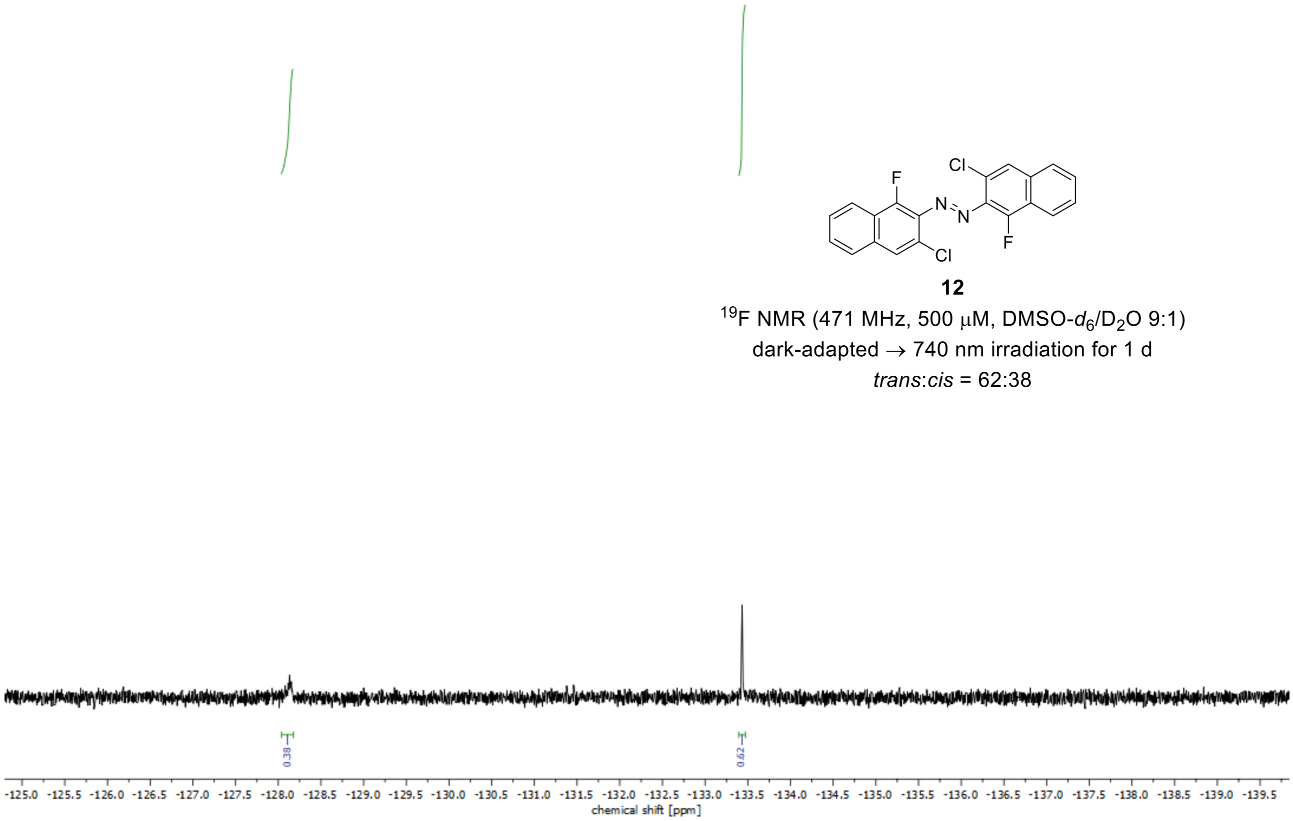

12

^{19}F NMR (471 MHz, 500 μM, DMSO-d_6/D_2O 9:1)

dark-adapted → 740 nm irradiation for 1 d

trans:*cis* = 62:38

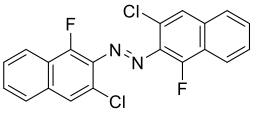

12

^{19}F NMR (471 MHz, 500 μM, DMSO-d_6/D_2O 9:1)
dark-adapted → 740 nm irradiation for 2 d
trans:*cis* = 47:53

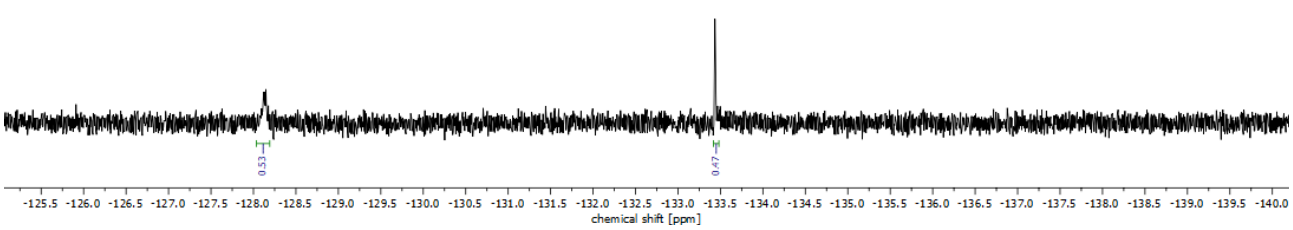

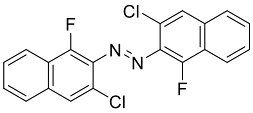

12

^{19}F NMR (471 MHz, 500 μM, DMSO-d_6/D_2O 9:1)
dark-adapted → 740 nm irradiation for 3 d
trans:*cis* = 41:59

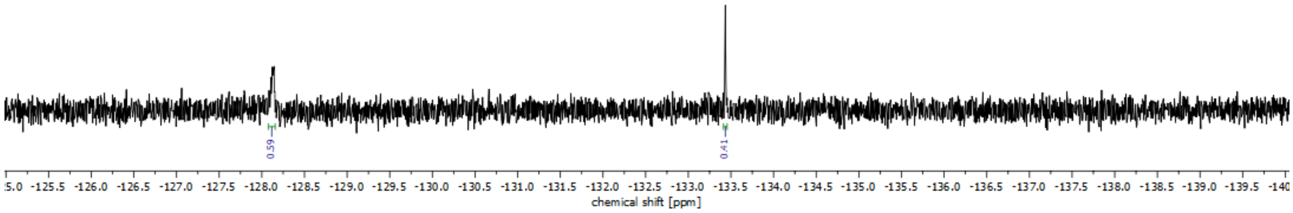

(*E*/*Z*)-3-Chloro-4-((2-chloro-6-fluorophenyl)diazenyl)-5-fluorobenzoic acid (17, 500 μM, DMSO-d_6/D_2O 9:1)

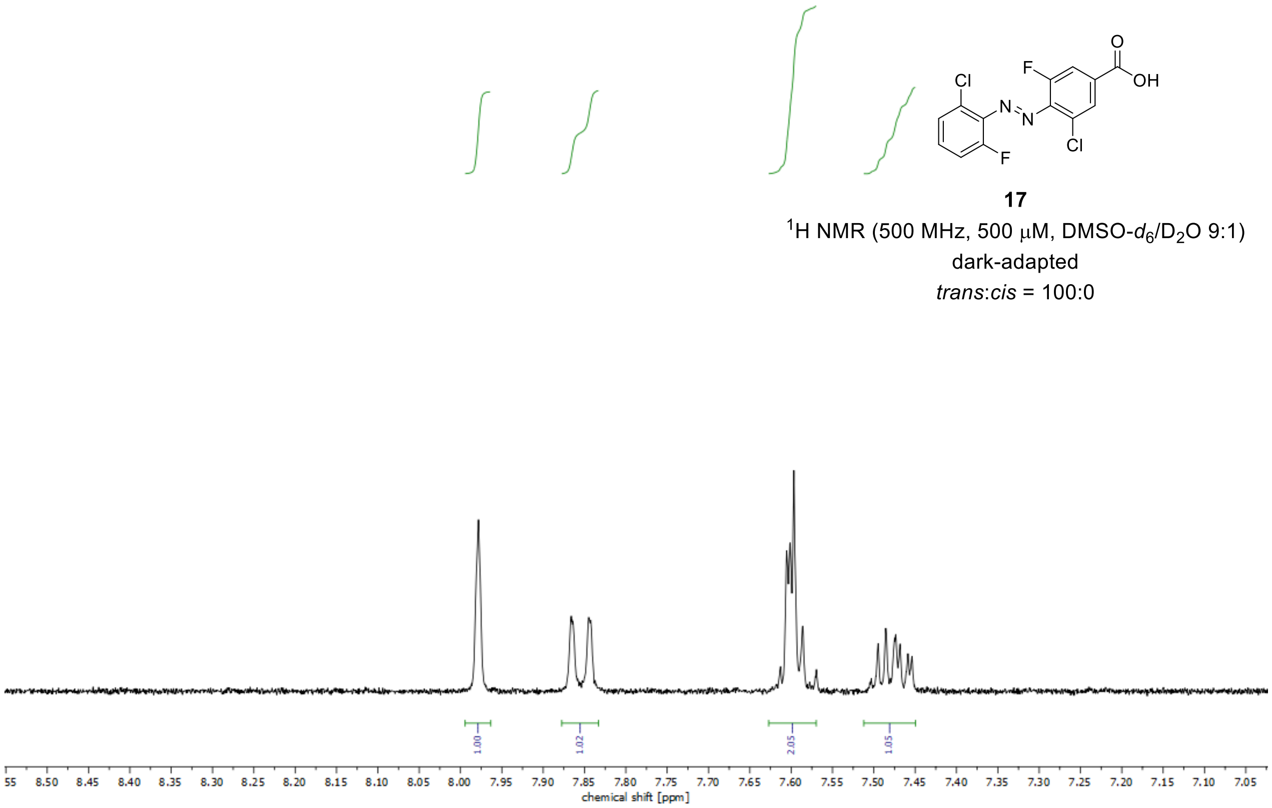

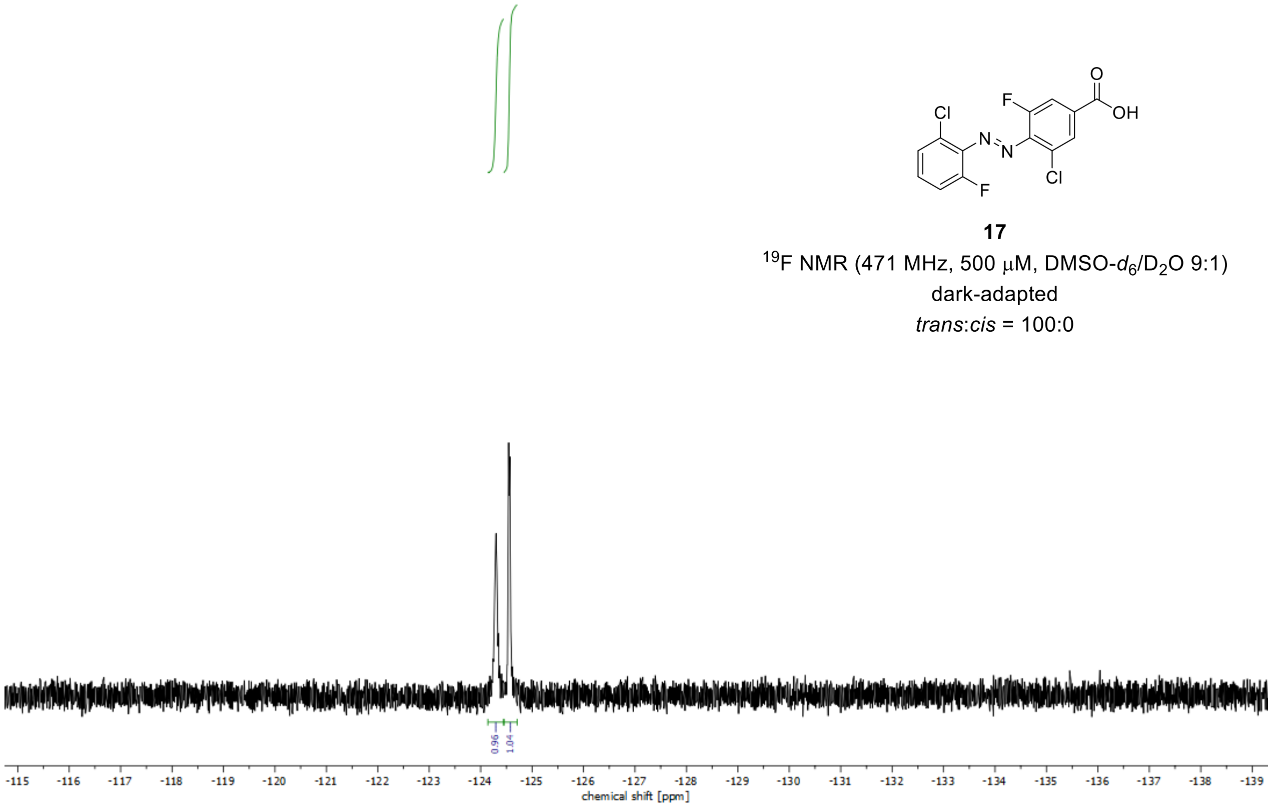

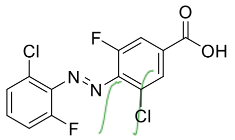

17

^{19}F NMR (471 MHz, 500 μM, DMSO-d_6/D_2O 9:1)
dark-adapted → 650 nm irradiation for 15 min
trans:*cis* = 27:73

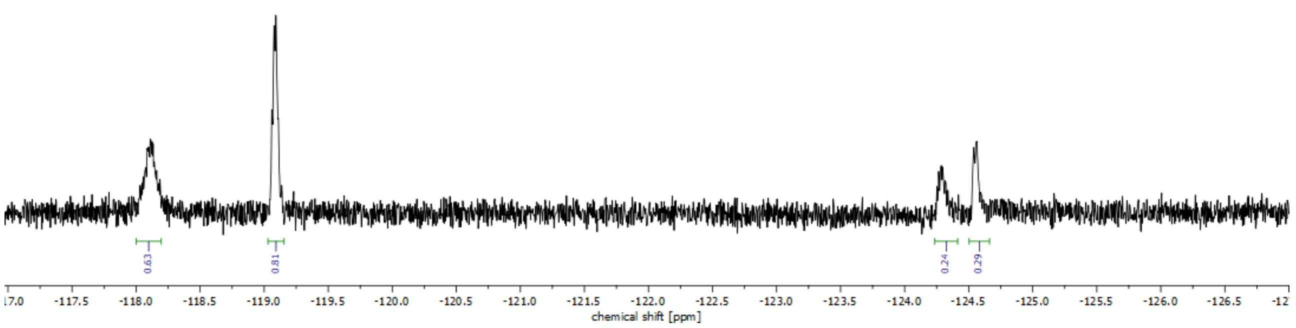

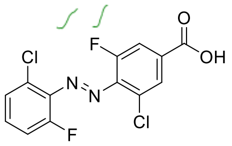

17

^{19}F NMR (471 MHz, 500 μM, DMSO-d_6/D_2O 9:1)
dark-adapted → 650 nm irradiation for 30 min
trans:*cis* = 11:89

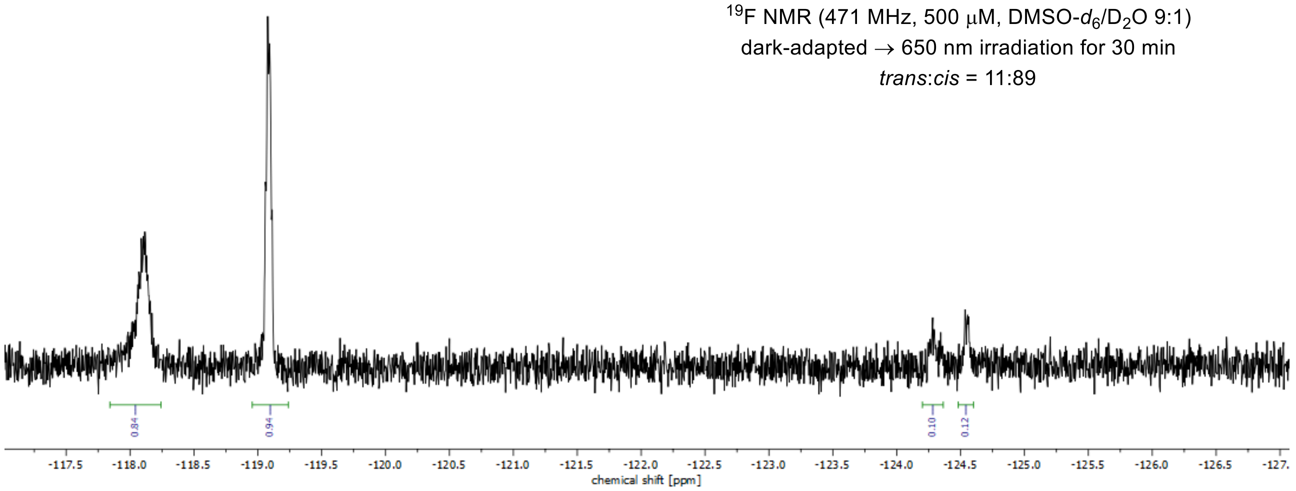

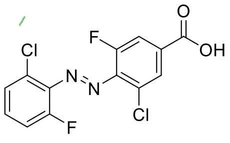

17

^{19}F NMR (471 MHz, 500 μM, DMSO-d_6/D_2O 9:1)

dark-adapted → 650 nm irradiation for 45 min

trans:*cis* = 5:95

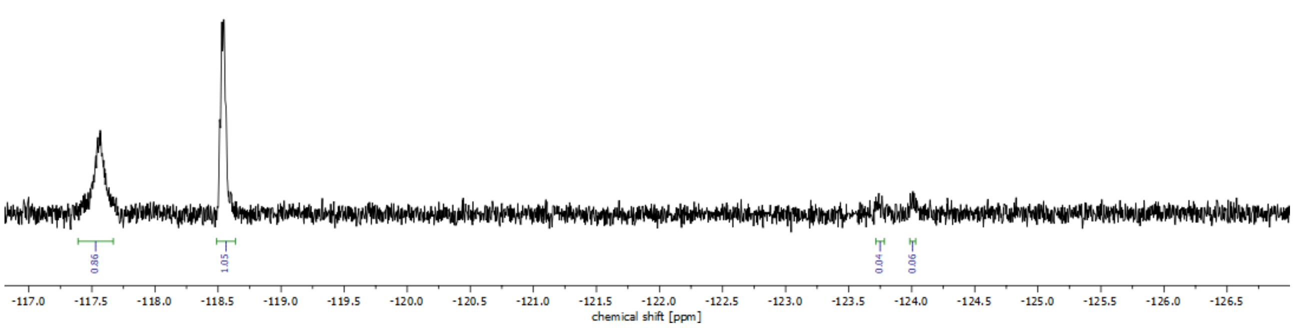

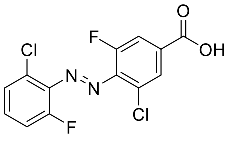

17

^{19}F NMR (471 MHz, 500 μM, DMSO-d_6/D_2O 9:1)

650 nm adapted → 525 nm irradiation for 15 min

trans:*cis* = 23:77

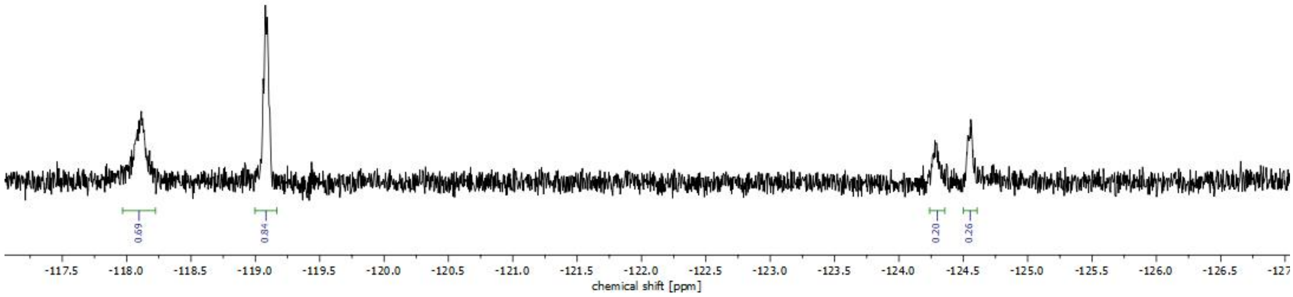

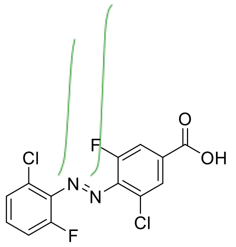

17

^{19}F NMR (471 MHz, 500 µM, DMSO-d_6/D_2O 9:1)

525 nm adapted → 450 nm irradiation for 15 min

trans:*cis* = 83:17

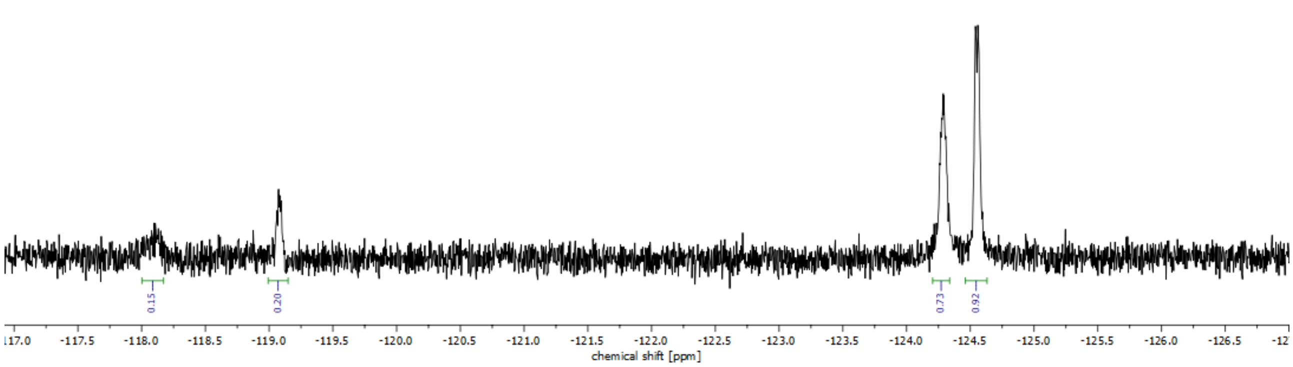

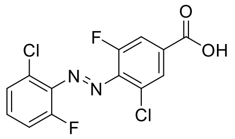

17

^{19}F NMR (471 MHz, 500 µM, DMSO-d_6/D_2O 9:1)

450 nm adapted → 365 nm irradiation for 15 min

trans:*cis* = 62:38

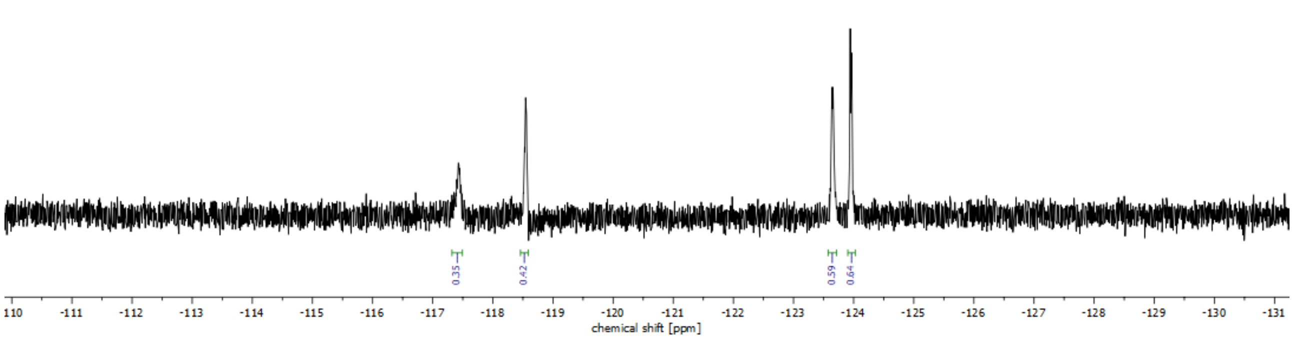

(*E*/*Z*)-1-(2-Chloro-6-fluoro-4-nitrophenyl)-2-(2-chloro-6-fluorophenyl)diazene (19, 500 µM, DMSO-d_6/D_2O 9:1)

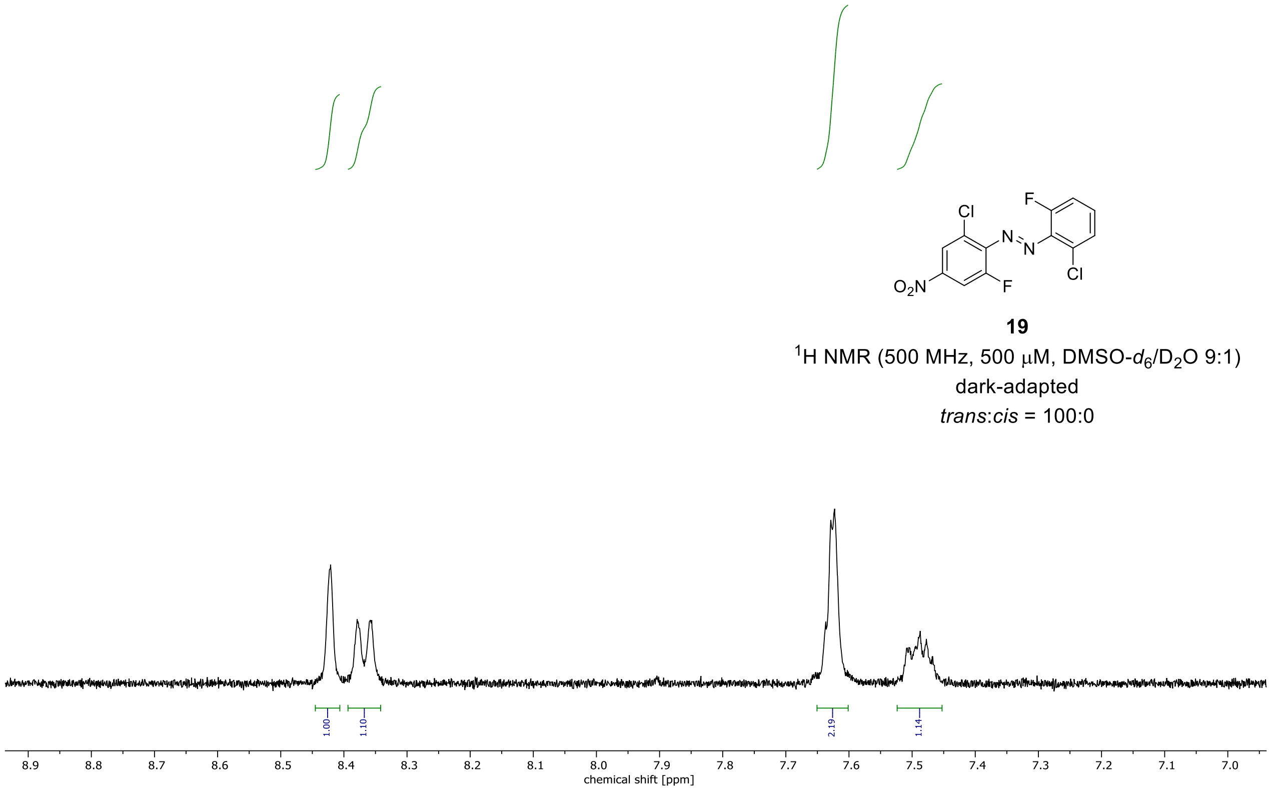

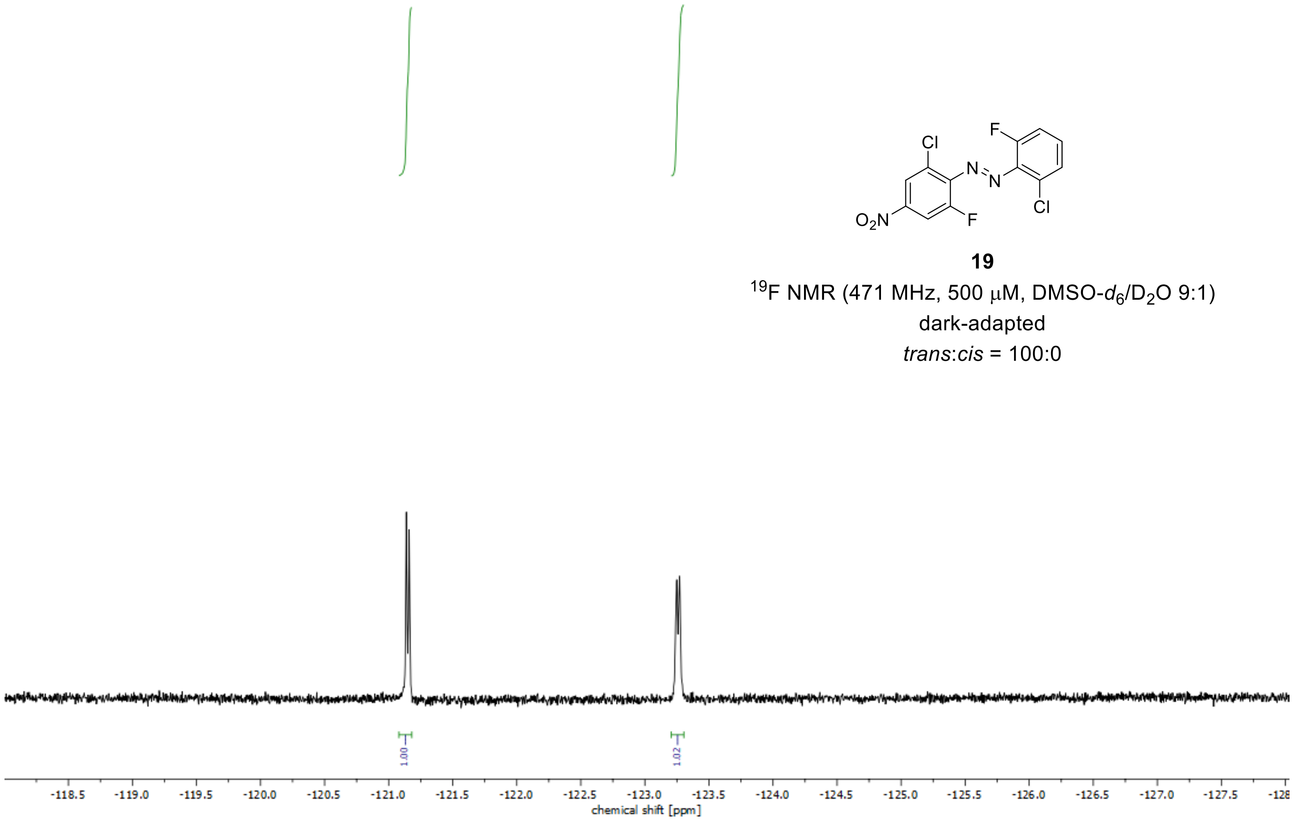

(*E*/*Z*)-3-Chloro-4-((2-chloro-6-fluoro-4-nitrophenyl)diazenyl)-5-fluorobenzoic acid (20, 500 µM, DMSO-d_6/D_2O 9:1)

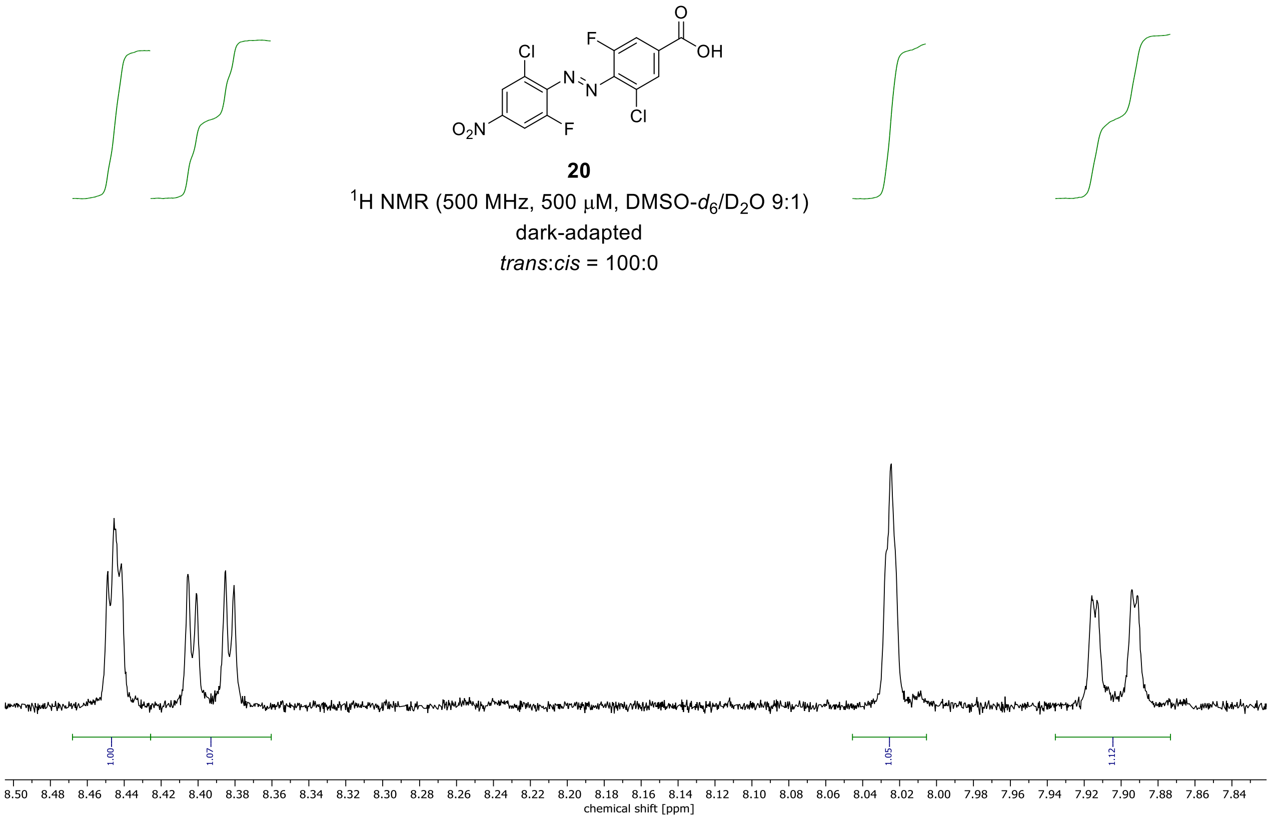

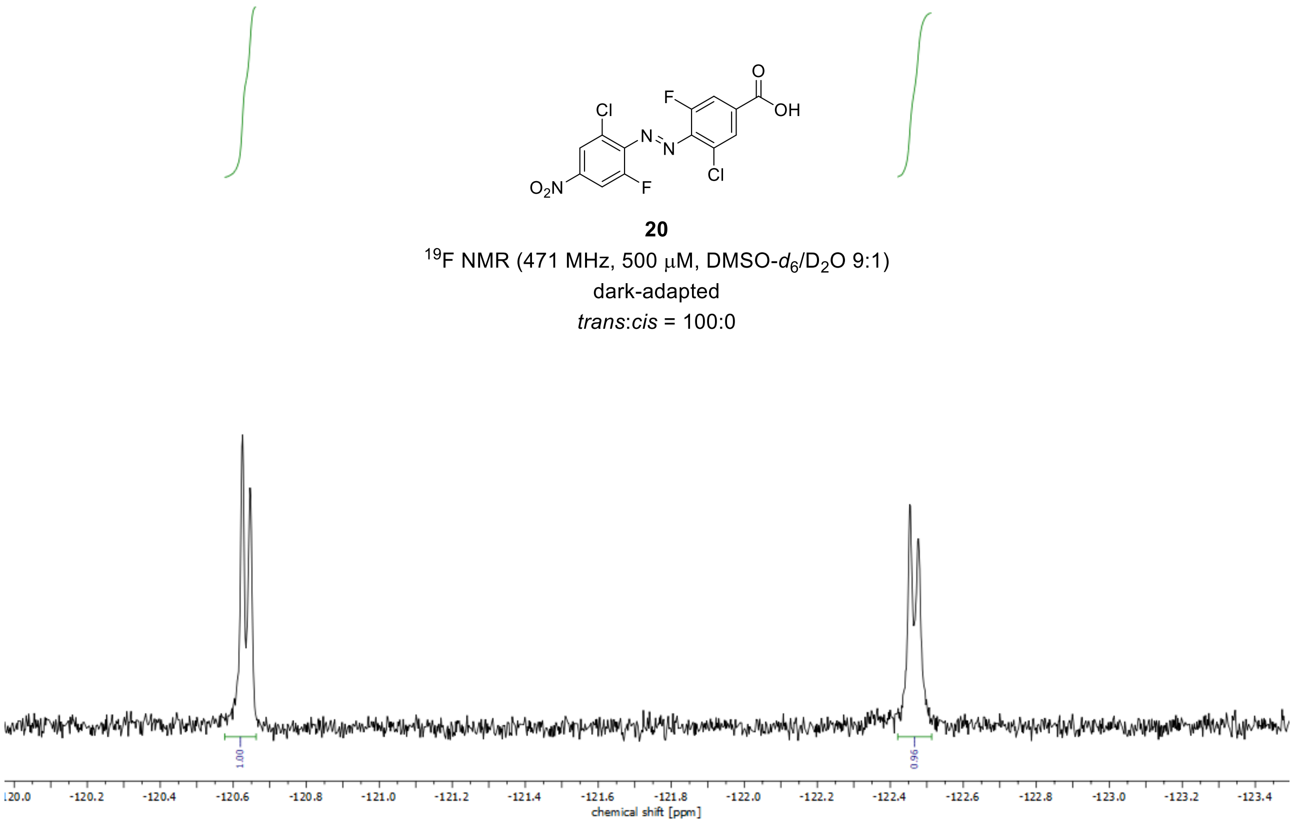

(*E*/*Z*)-Methyl-3-chloro-4-((2-chloro-6-fluorophenyl)diazenyl)-5-fluorobenzoate (21, 500 µM, DMSO-*d*$_6$/D$_2$O 9:1)

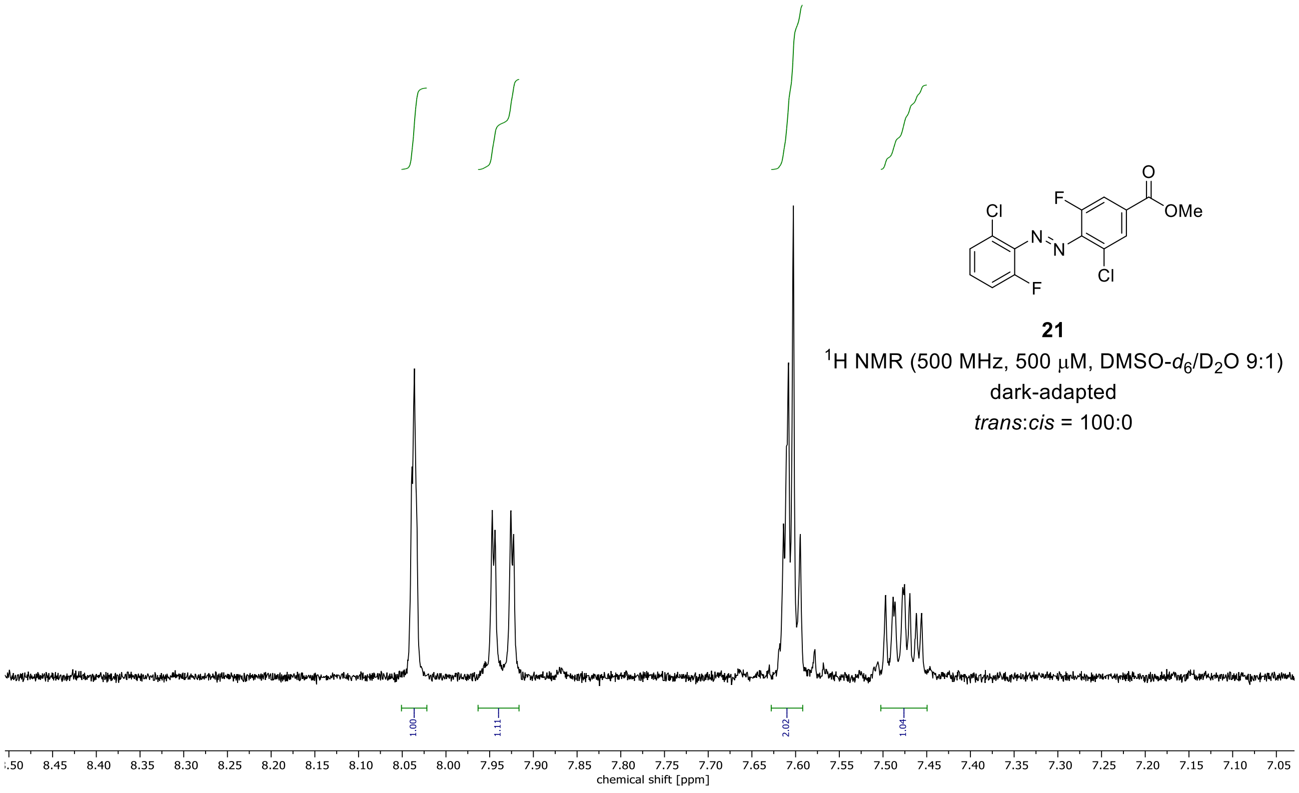

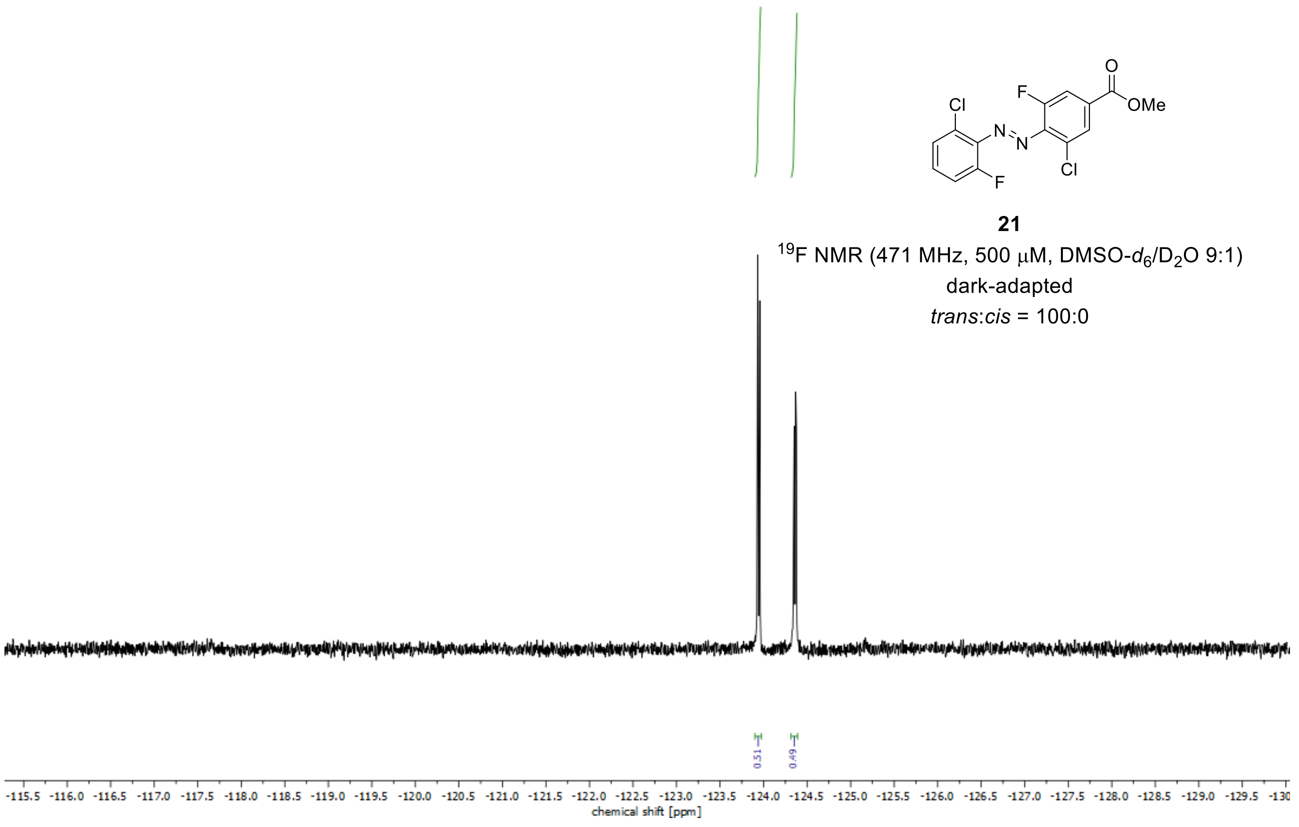

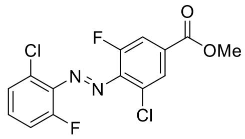

21

^{19}F NMR (471 MHz, 500 μM, DMSO-d_6/D_2O 9:1)

dark-adapted → 650 nm irradiation for 15 min

trans:*cis* = 37:63

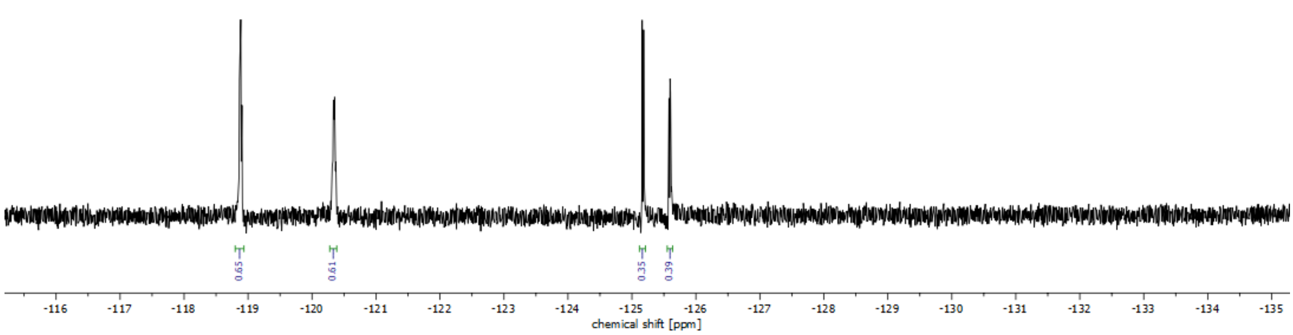

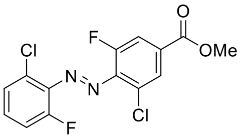

21

^{19}F NMR (471 MHz, 500 μM, DMSO-d_6/D_2O 9:1)

dark-adapted → 650 nm irradiation for 30 min

trans:*cis* = 13:87

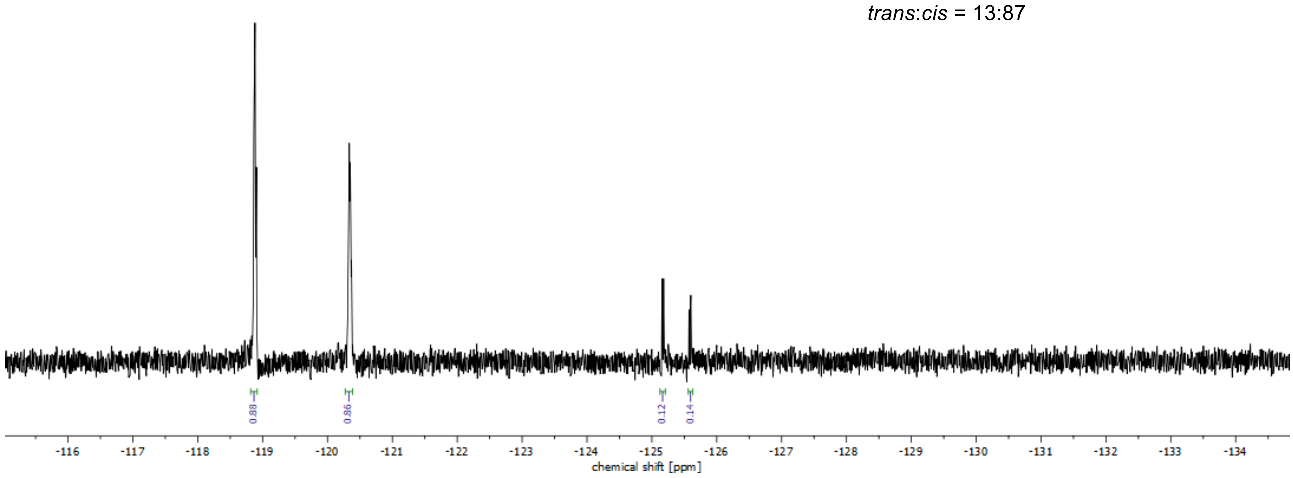

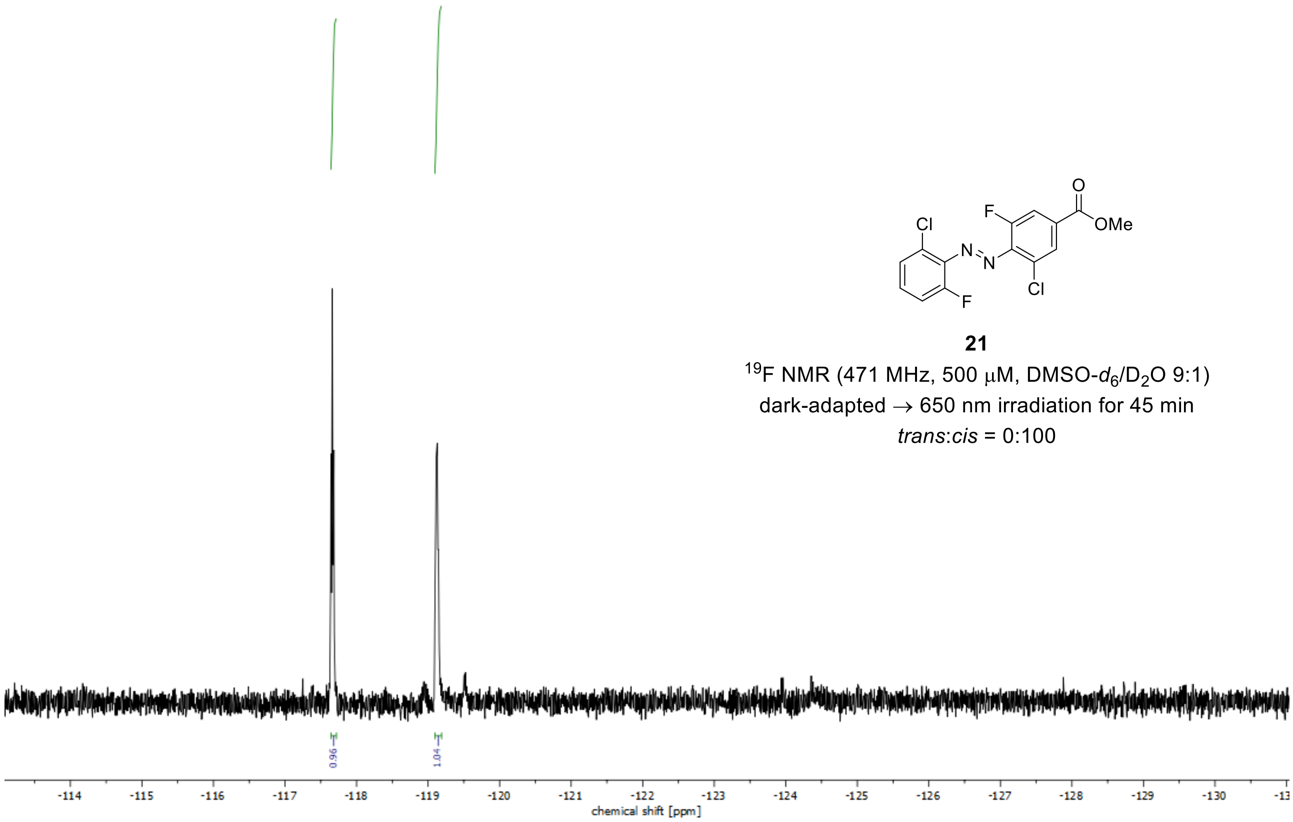

21

^{19}F NMR (471 MHz, 500 μM, DMSO-d_6/D_2O 9:1)

dark-adapted → 650 nm irradiation for 45 min

trans:*cis* = 0:100

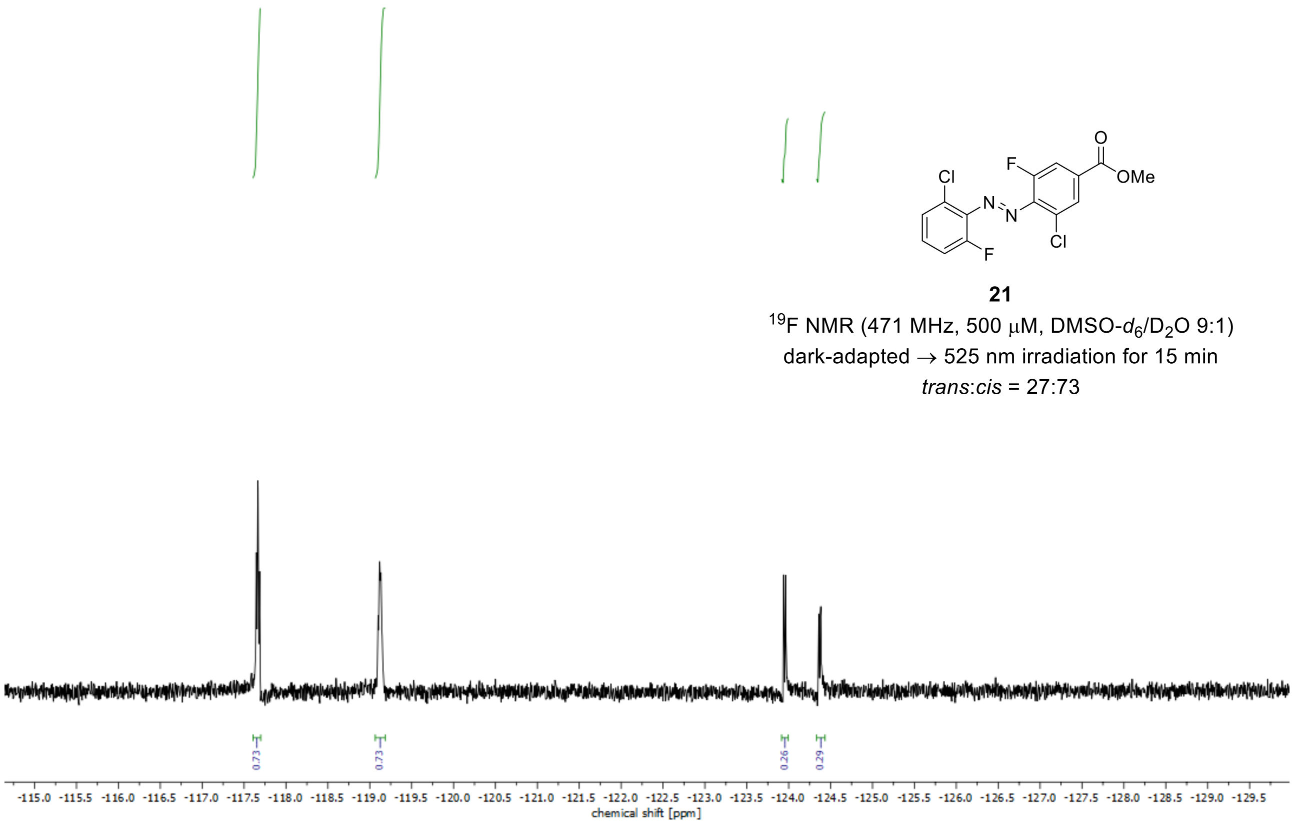

21

^{19}F NMR (471 MHz, 500 μM, DMSO-d_6/D_2O 9:1)

dark-adapted → 525 nm irradiation for 15 min

trans:*cis* = 27:73

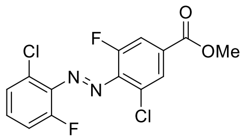

21

^{19}F NMR (471 MHz, 500 μM, DMSO-d_6/D_2O 9:1)
dark-adapted → 450 nm irradiation for 15 min
trans:*cis* = 82:18

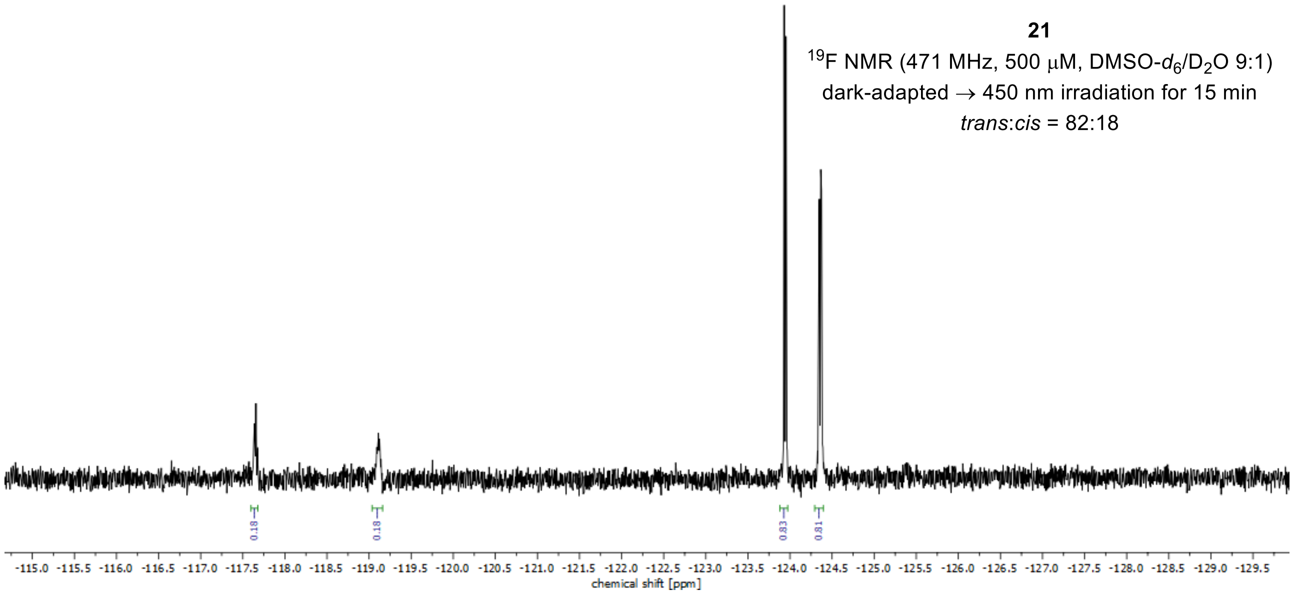

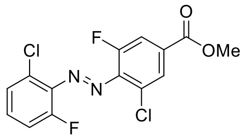

21

^{19}F NMR (471 MHz, 500 μM, DMSO-d_6/D_2O 9:1)
450 nm adapted → 365 nm irradiation for 15 min
trans:*cis* = 61:39

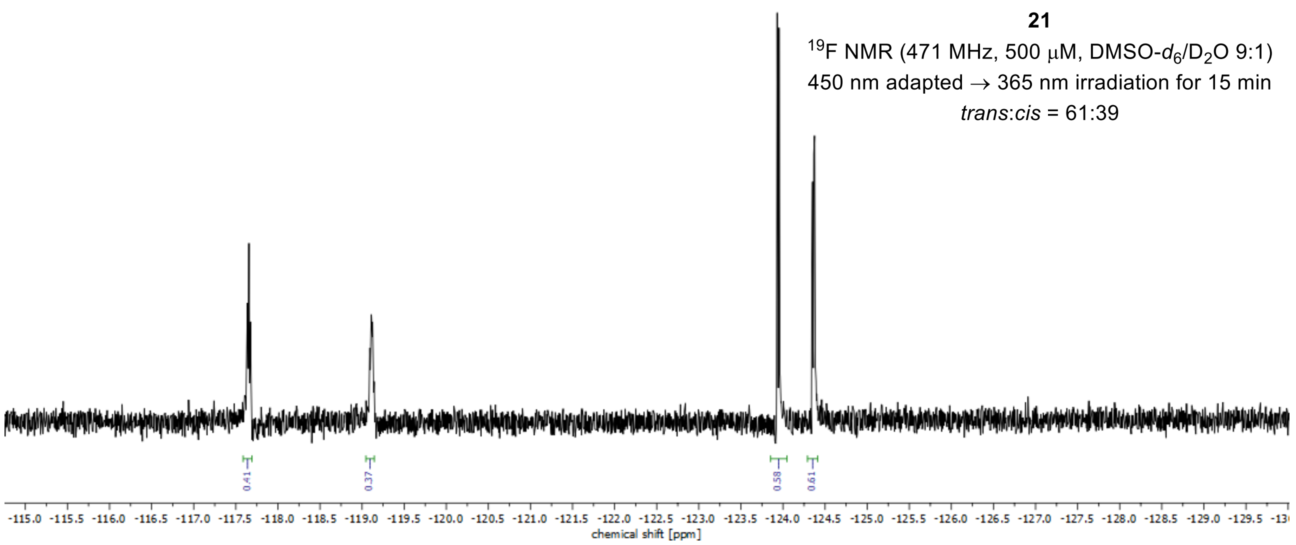

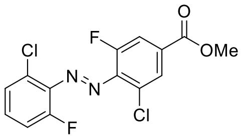

21

^{19}F NMR (471 MHz, 500 μM, DMSO-d_6/D_2O 9:1)
dark-adapted → 740 nm irradiation for 6 h
trans:*cis* = 75:25

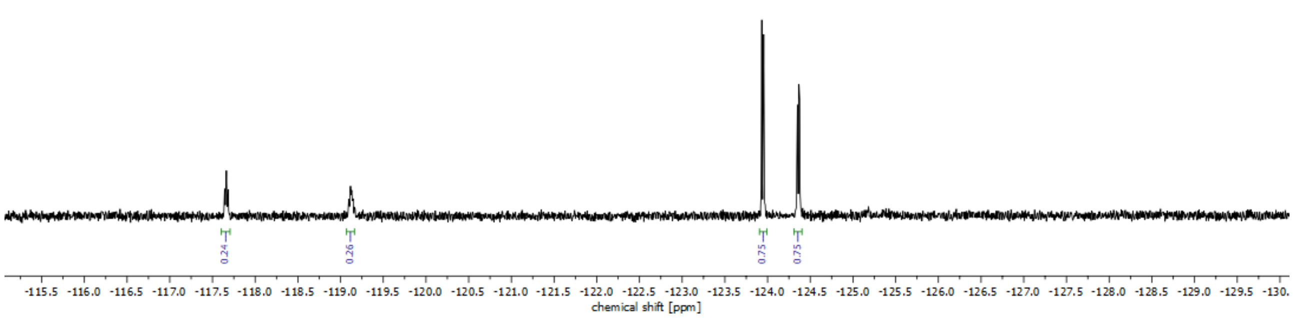

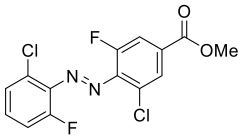

21

^{19}F NMR (471 MHz, 500 μM, DMSO-d_6/D_2O 9:1)
dark-adapted → 740 nm irradiation for 24 h
trans:*cis* = 63:37

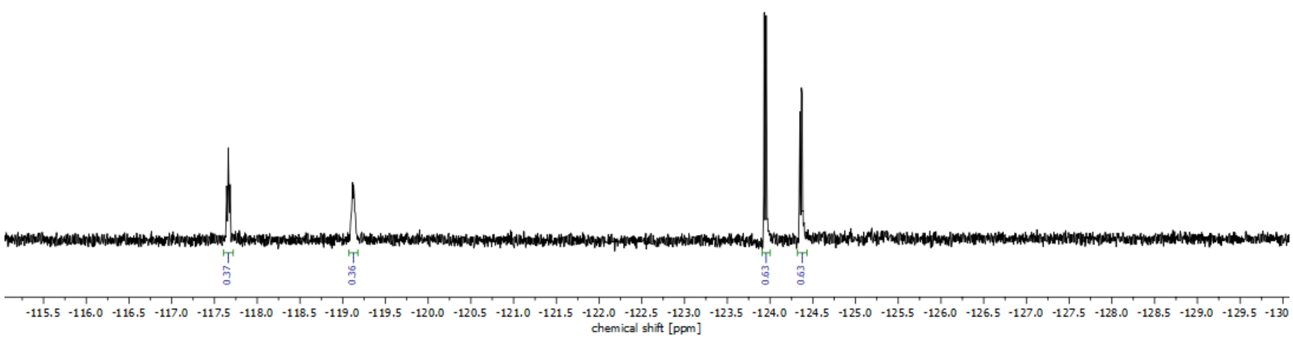

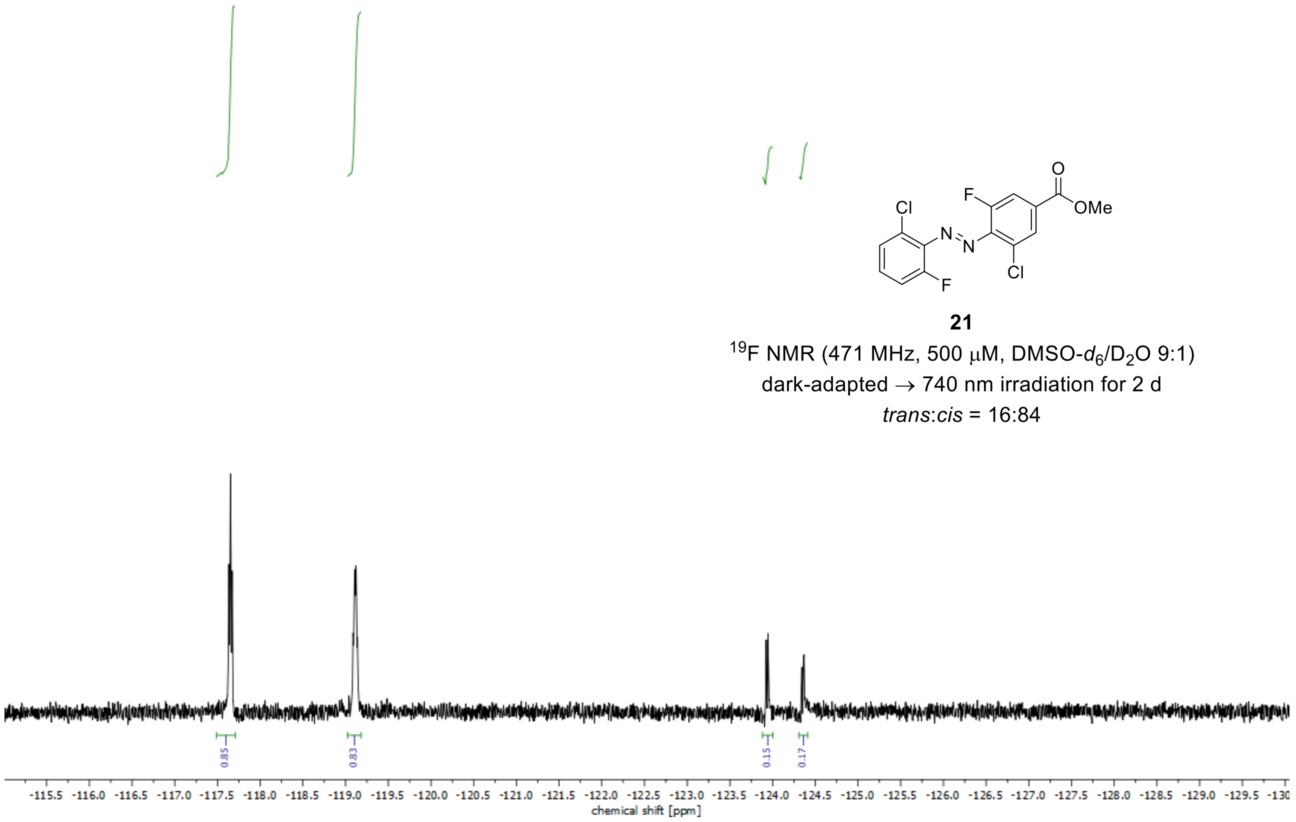

21

^{19}F NMR (471 MHz, 500 μM, DMSO-d_6/D_2O 9:1)
dark-adapted → 740 nm irradiation for 2 d
trans:*cis* = 16:84

(*E*/*Z*)-Methyl-3-bromo-4-((2-bromo-6-fluorophenyl)diazenyl)-5-fluorobenzoate (22, 500 μM, DMSO-*d*$_6$/D$_2$O 9:1)

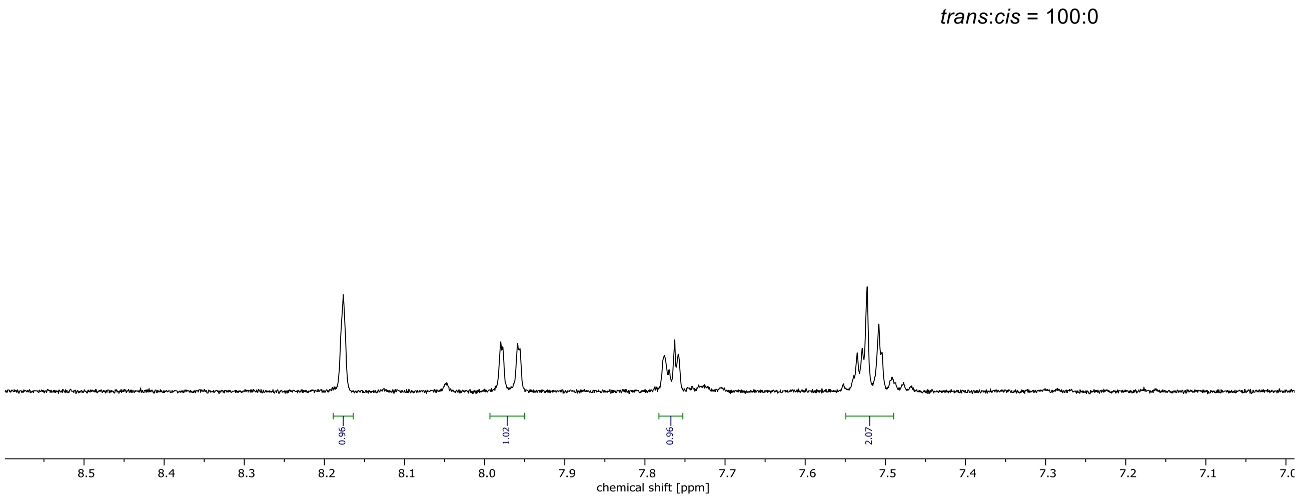

22

^{1}H NMR (500 MHz, 500 μM, DMSO-d_6/D_2O 9:1)
dark-adapted
trans:*cis* = 100:0

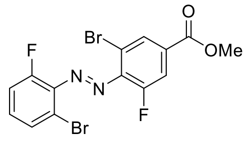

22

^{19}F NMR (471 MHz, 500 μM, DMSO-d_6/D_2O 9:1)

dark-adapted

trans:*cis* = 100:0

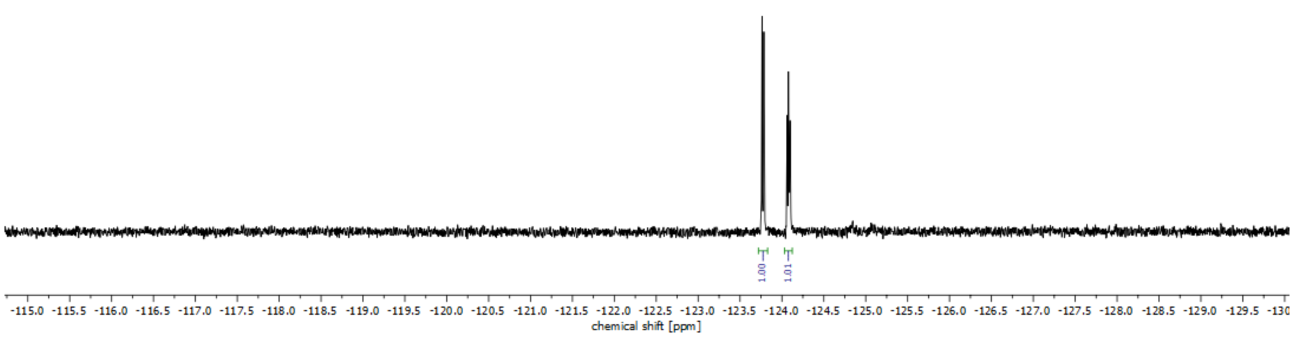

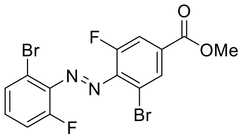

22

^{19}F NMR (471 MHz, 500 μM, DMSO-d_6/D_2O 9:1)

dark-adapted → 650 nm irradiation for 15 min

trans:*cis* = 22:78

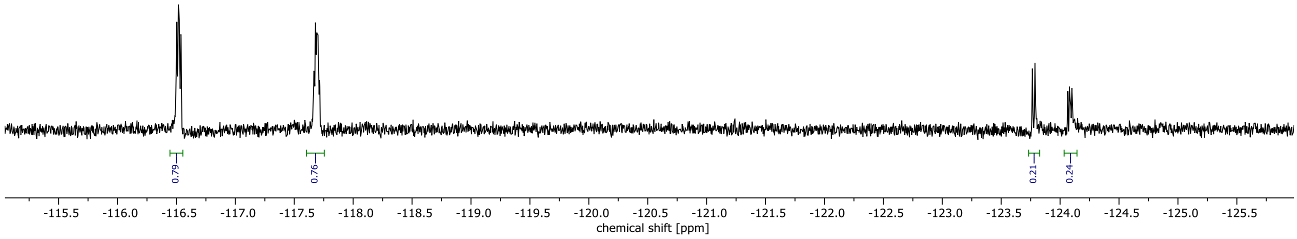

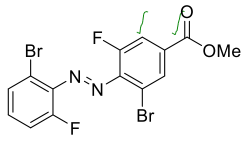

22

^{19}F NMR (471 MHz, 500 μM, DMSO-d_6/D_2O 9:1)

dark-adapted → 650 nm irradiation for 30 min

trans:*cis* = 11:89

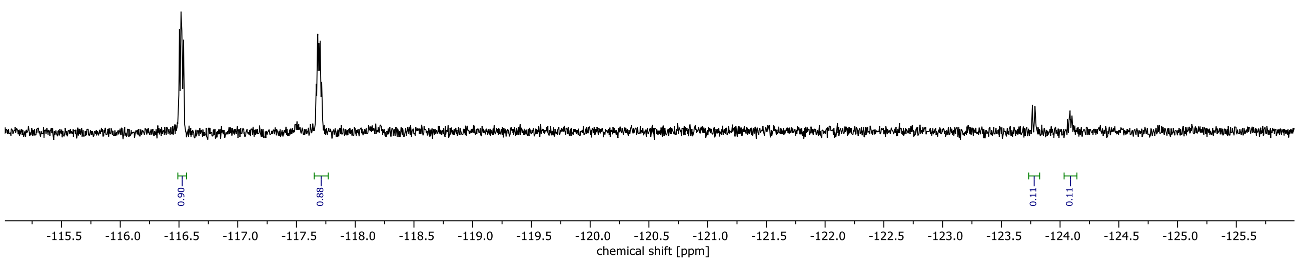

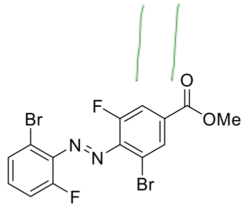

22

^{19}F NMR (471 MHz, 500 μM, DMSO-d_6/D_2O 9:1)

650 nm adapted → 525 nm irradiation for 15 min

trans:*cis* = 31:69

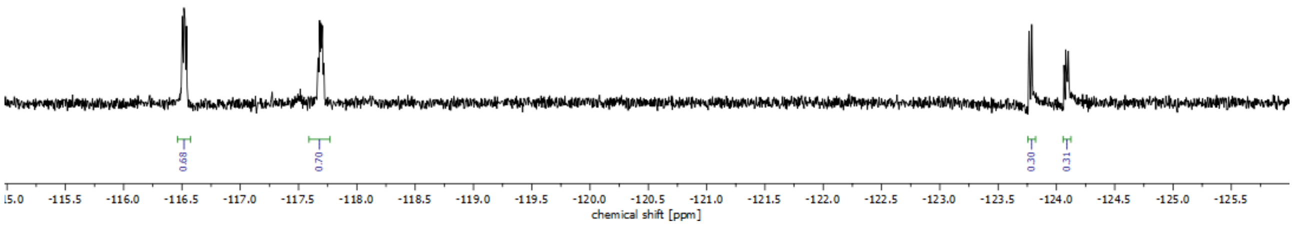

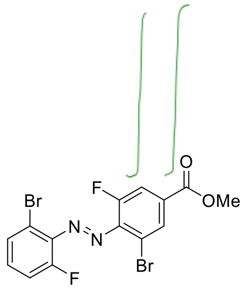

22

^{19}F NMR (471 MHz, 500 μM, DMSO-d_6/D_2O 9:1)
525 nm adapted → 450 nm irradiation for 15 min
trans:*cis* = 83:17

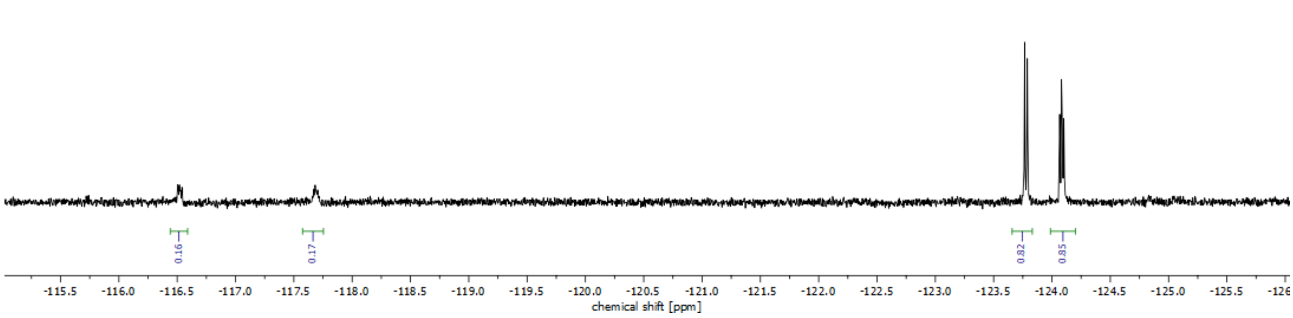

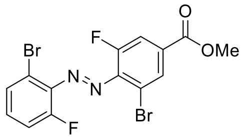

22

^{19}F NMR (471 MHz, 500 μM, DMSO-d_6/D_2O 9:1)
450 nm adapted → 365 nm irradiation for 15 min
trans:*cis* = 65:35

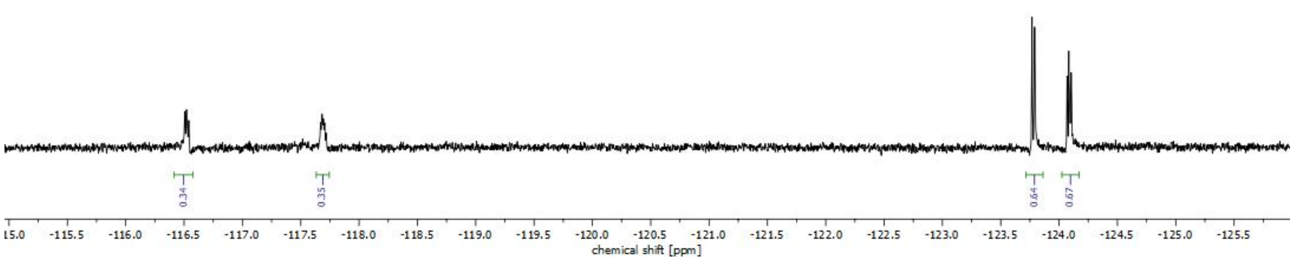

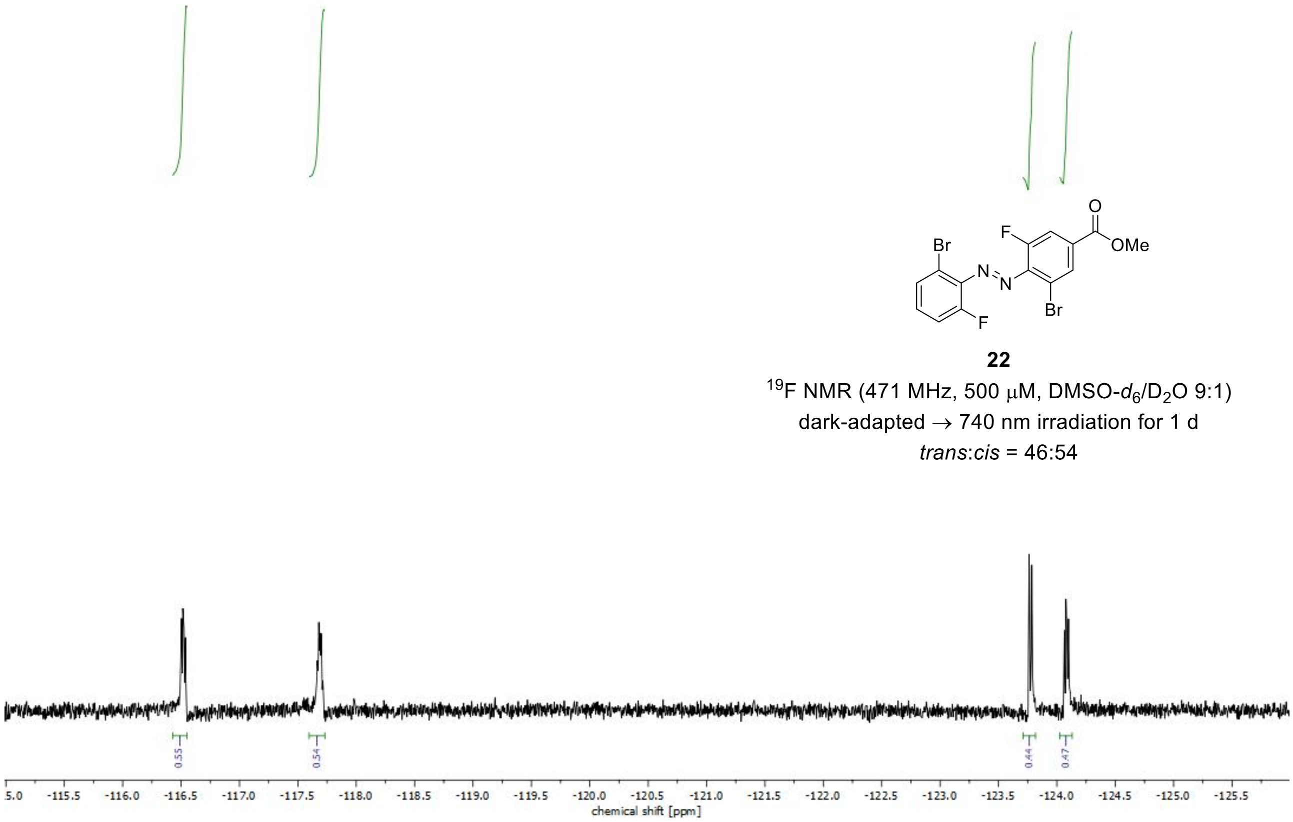

(*E*/*Z*)-Methyl-3,5-dichloro-4-((2,6-dichlorophenyl)diazenyl)benzoate (23, 500 μM, DMSO-d_6/D_2O 9:1)

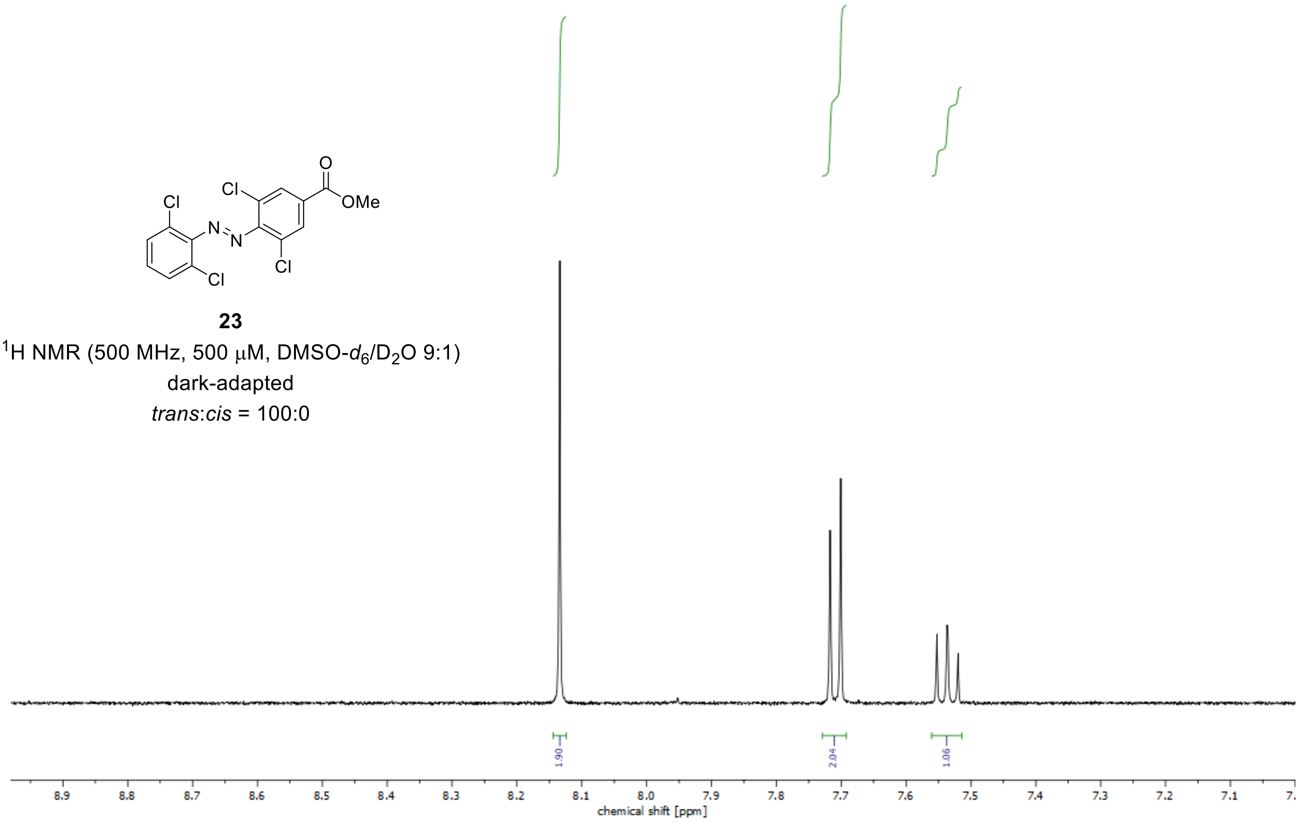

23

^{1}H NMR (500 MHz, 500 μM, DMSO-d_6/D_2O 9:1)
dark-adapted → 650 nm irradiation for 15 min
trans:*cis* = 13:87

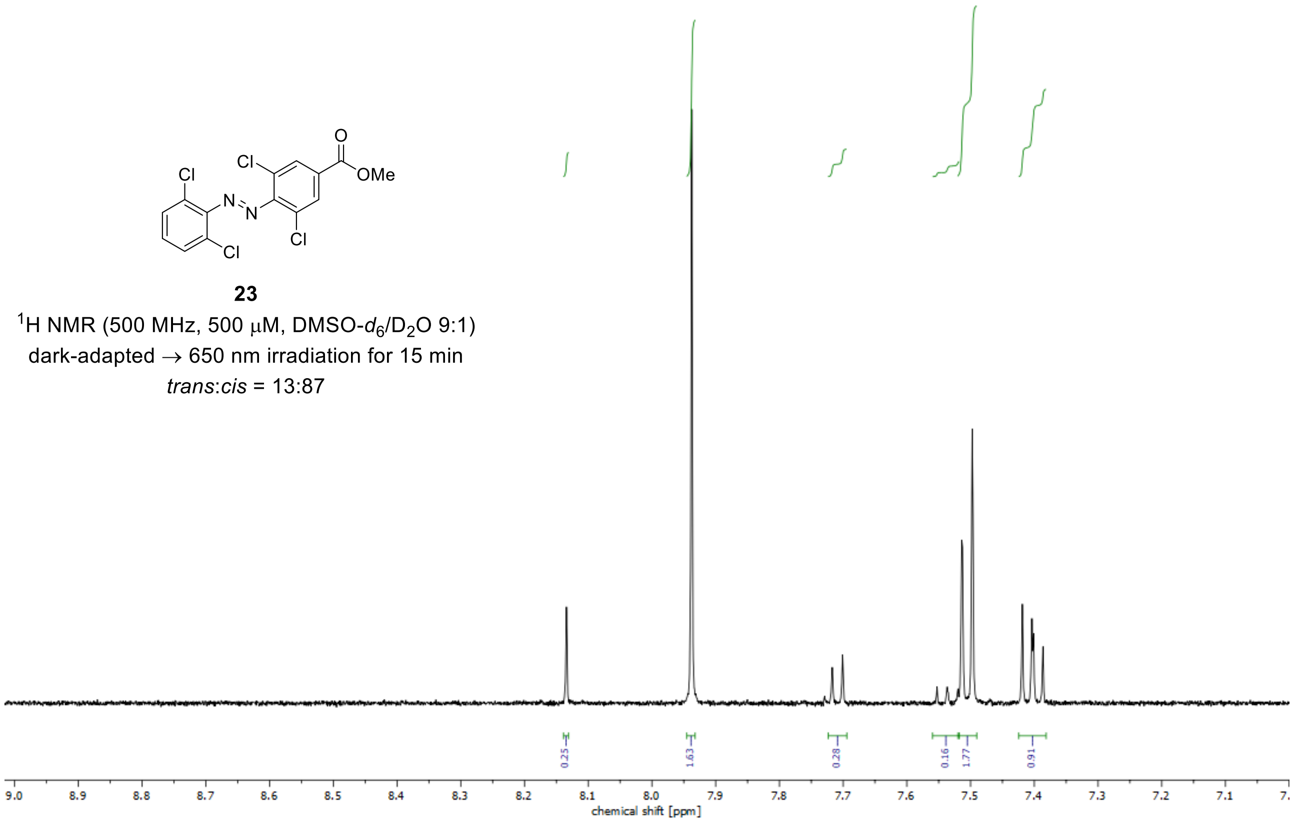

23

^{1}H NMR (500 MHz, 500 μM, DMSO-d_6/D_2O 9:1)
650 nm adapted → 525 nm irradiation for 15 min
trans:*cis* = 59:41

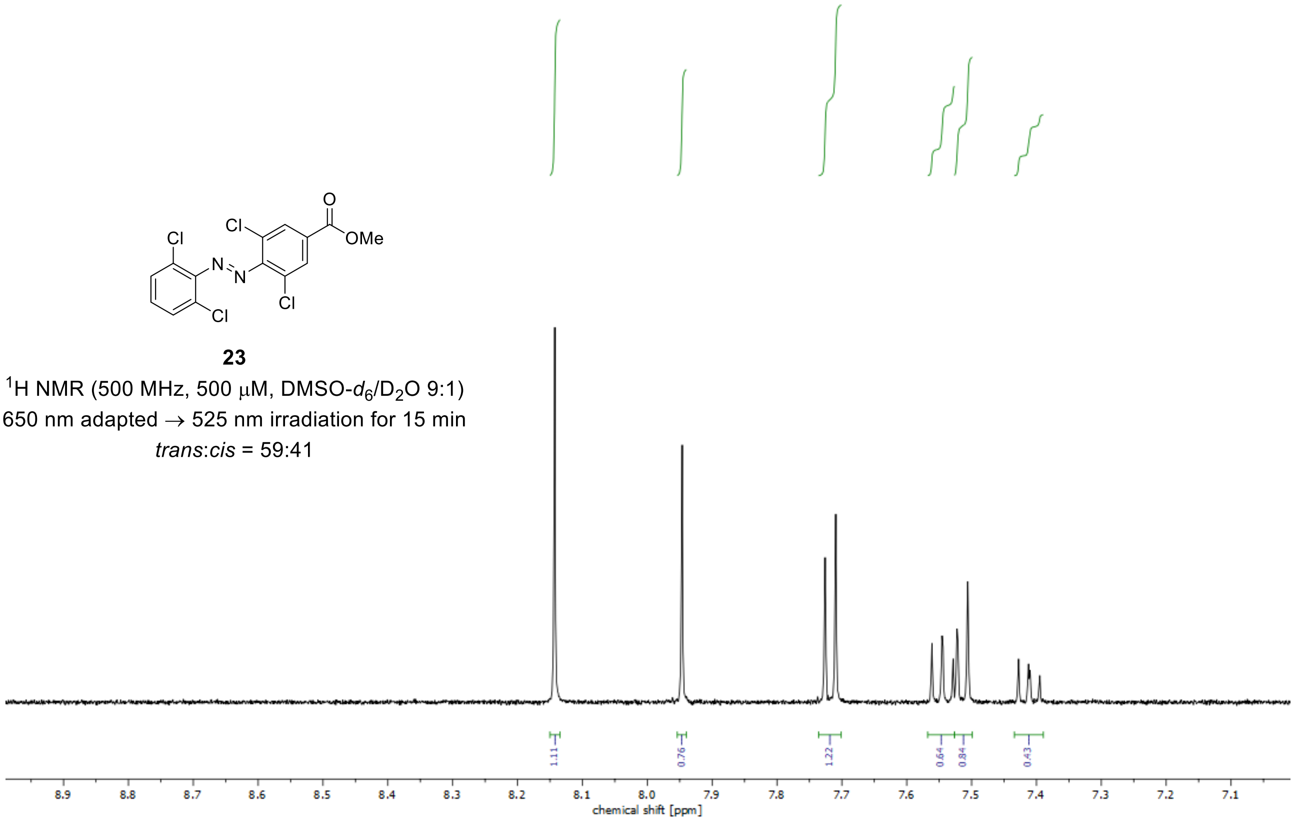

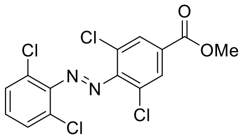

23

^{1}H NMR (500 MHz, 500 μM, DMSO-d_6/D_2O 9:1)

525 nm adapted → 450 nm irradiation for 15 min

trans:*cis* = 87:13

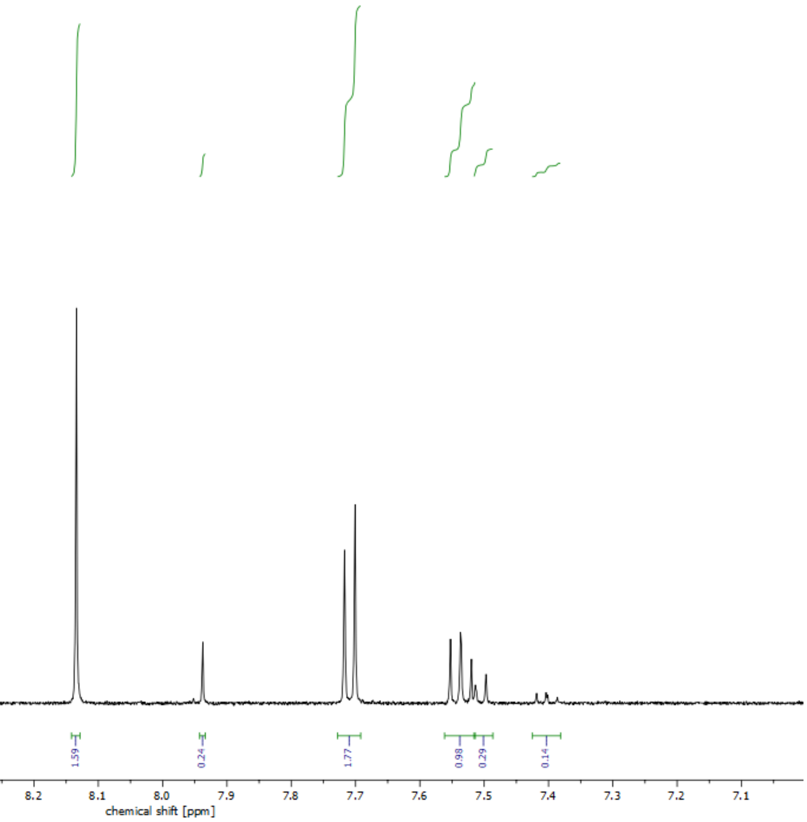

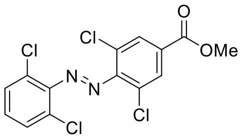

23

^{1}H NMR (500 MHz, 500 μM, DMSO-d_6/D_2O 9:1)

450 nm adapted → 365 nm irradiation for 15 min

trans:*cis* = 74:26

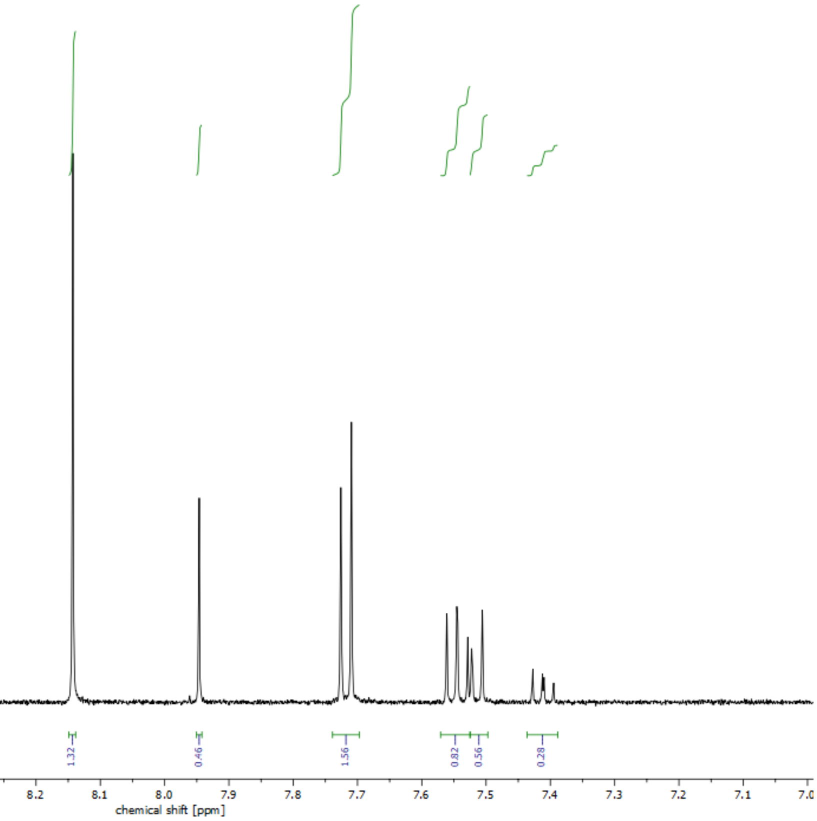

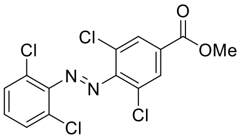

23

^{1}H NMR (500 MHz, 500 μM, DMSO-d_6/D_2O 9:1)

dark-adapted → 740 nm irradiation for 1 d

trans:*cis* = 47:53

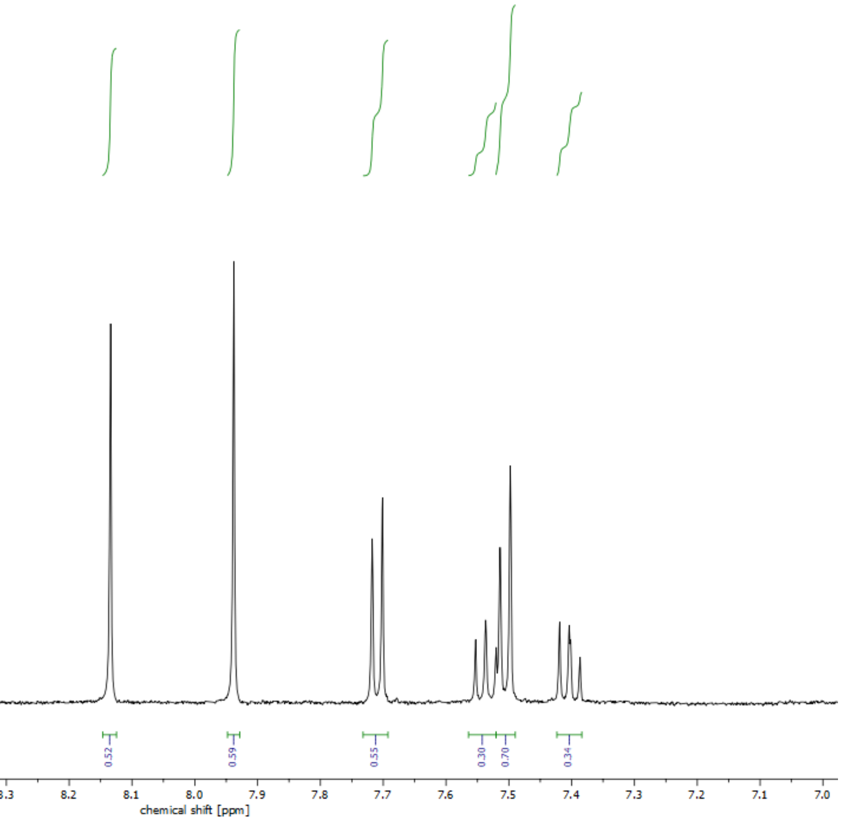

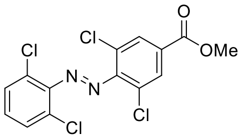

23

^{1}H NMR (500 MHz, 500 μM, DMSO-d_6/D_2O 9:1)

dark-adapted → 740 nm irradiation for 2 d

trans:*cis* = 19:81

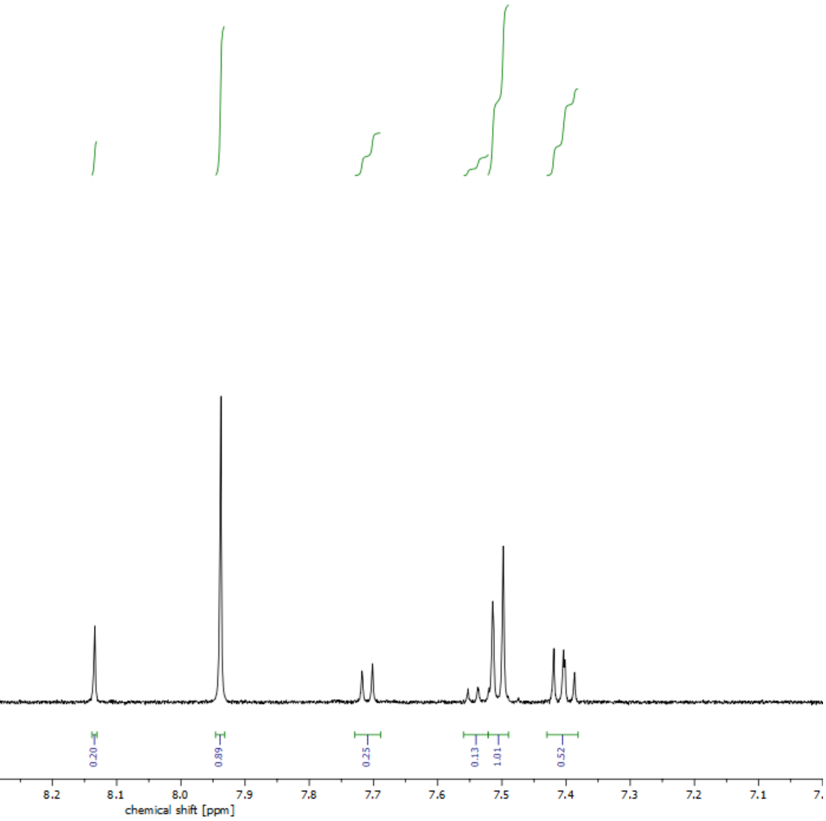

(*E*/*Z*)-3-Chloro-4-((2-chloro-6-fluorophenyl)diazenyl)-5-fluoro-N-methylbenzamide (24, 500 µM, DMSO-d_6/D_2O)

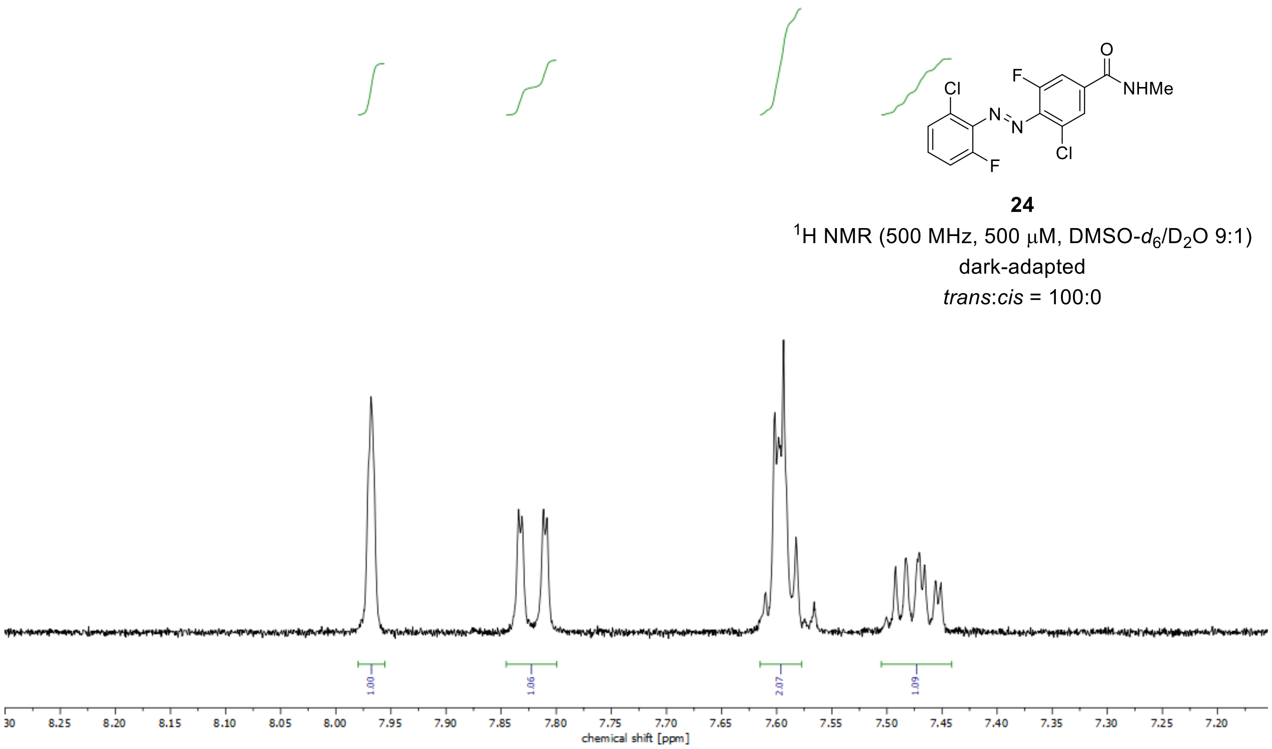

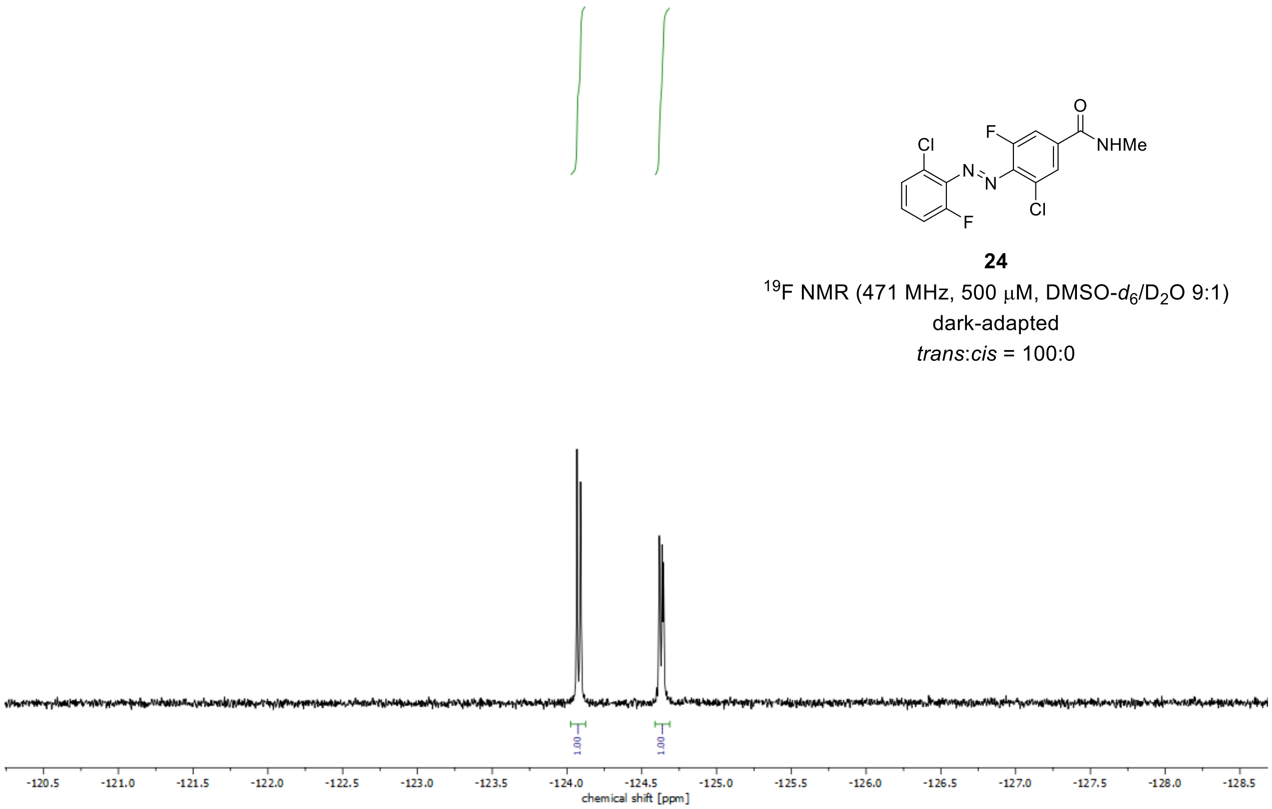

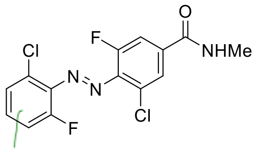

24

^{19}F NMR (471 MHz, 500 μM, DMSO-d_6/D_2O 9:1)
dark-adapted → 650 nm irradiation for 15 min
trans:*cis* = 18:82

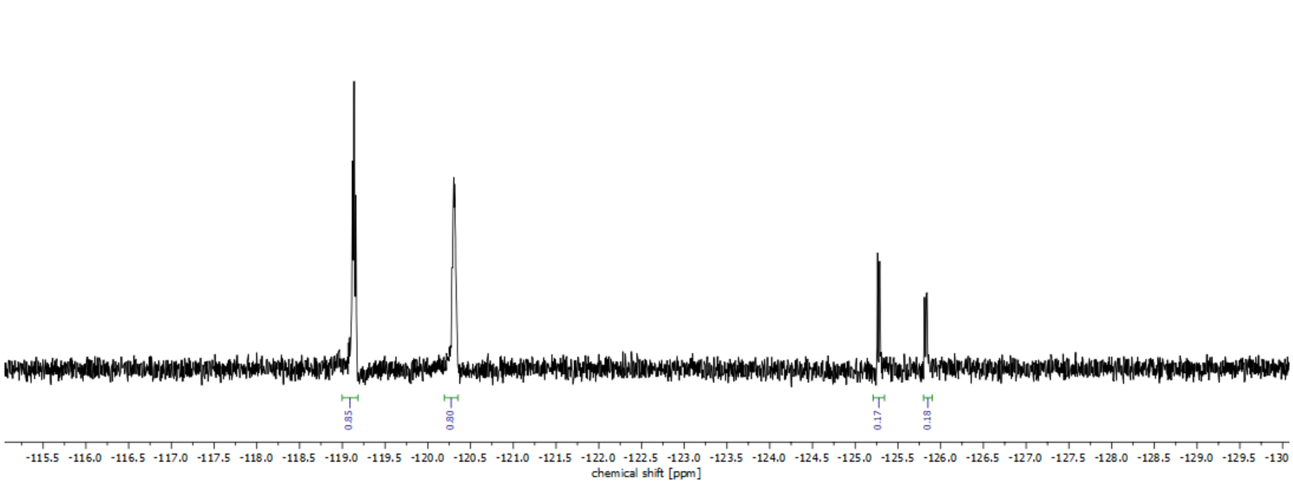

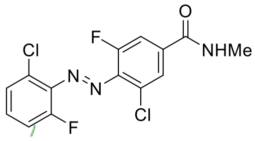

24

^{19}F NMR (471 MHz, 500 μM, DMSO-d_6/D_2O 9:1)
dark-adapted → 650 nm irradiation for 30 min
trans:*cis* = 6:94

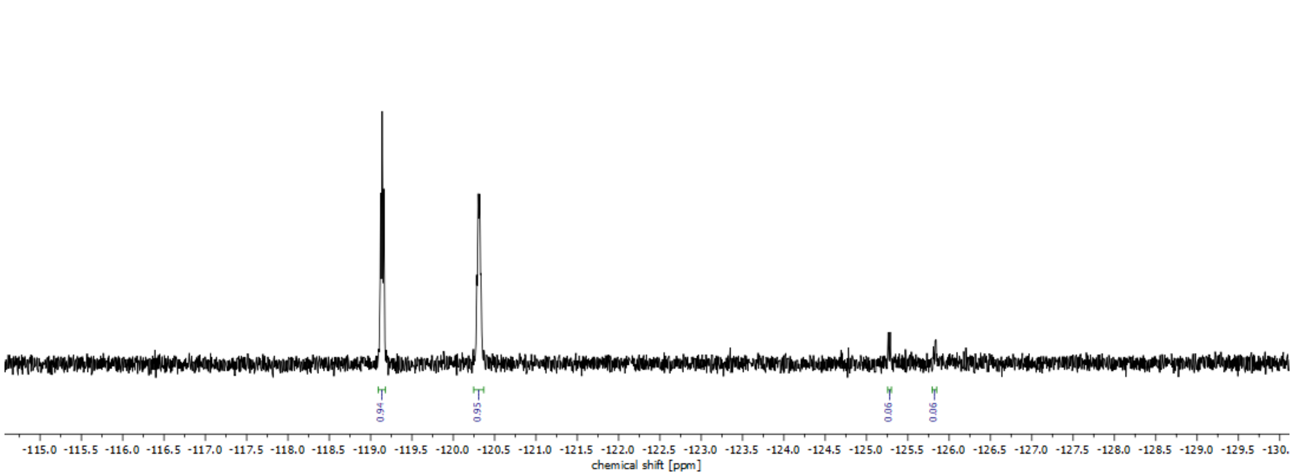

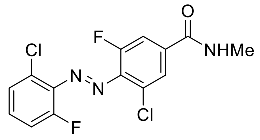

24

^{19}F NMR (471 MHz, 500 μM, DMSO-d_6/D_2O 9:1)

dark-adapted → 650 nm irradiation for 45 min

trans:*cis* = 1:99

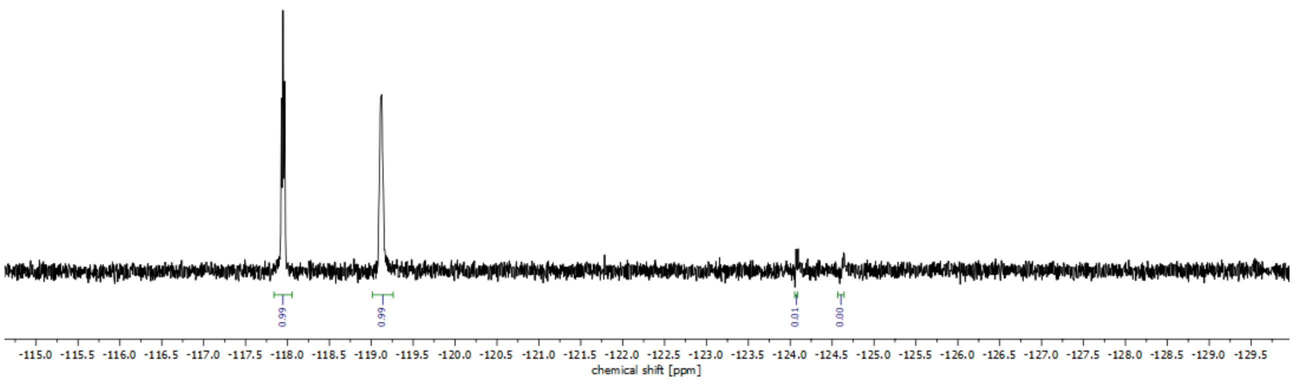

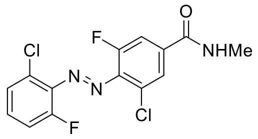

24

^{19}F NMR (471 MHz, 500 μM, DMSO-d_6/D_2O 9:1)

450 nm adapted → 525 nm irradiation for 15 min

trans:*cis* = 23:77

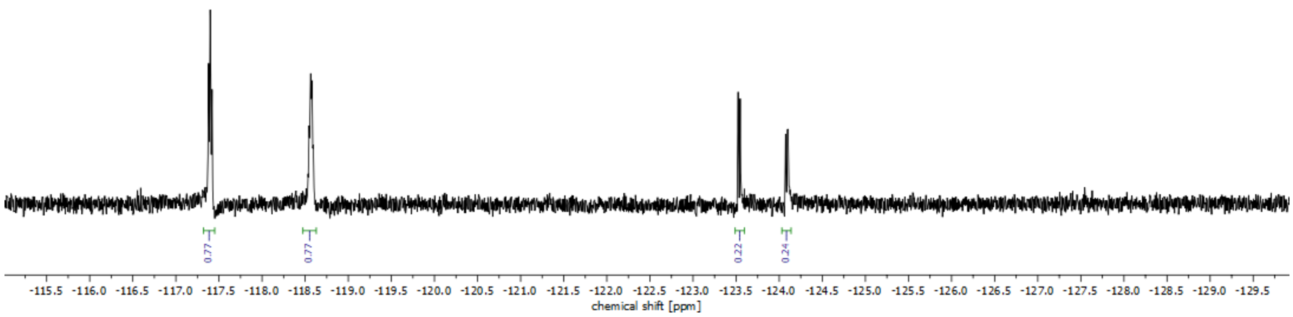

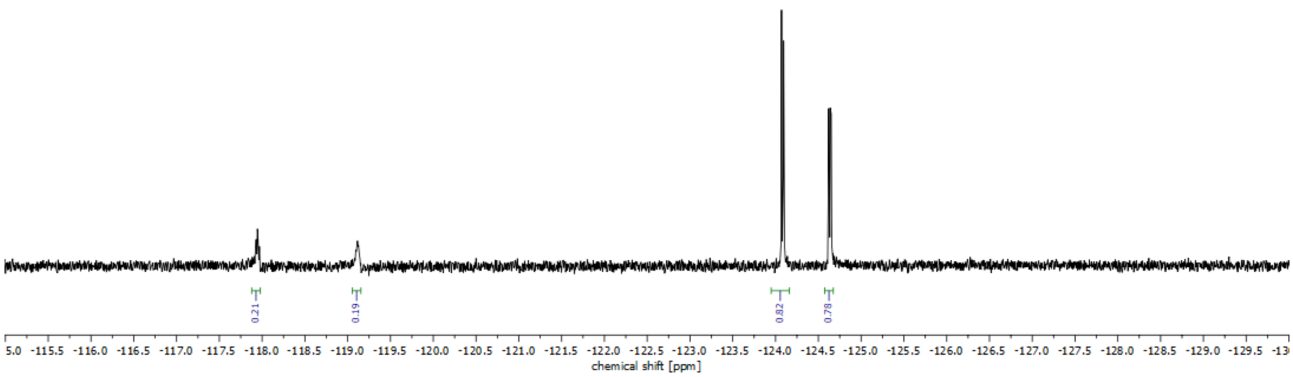

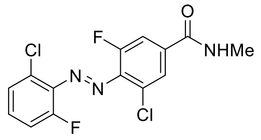

24

^{19}F NMR (471 MHz, 500 µM, DMSO-d_6/D_2O 9:1)

365 nm adapted → 450 nm irradiation for 15 min

trans:*cis* = 80:20

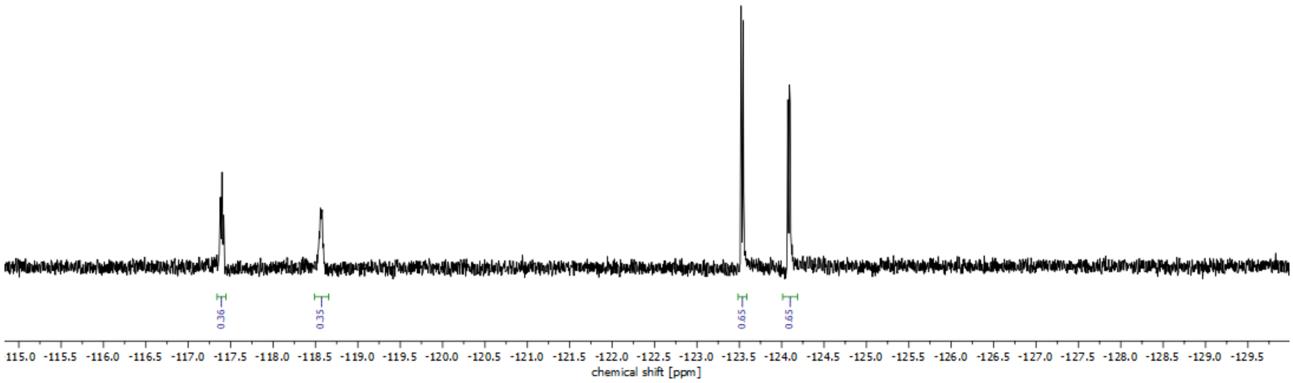

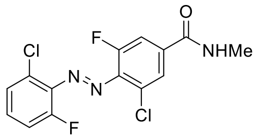

24

^{19}F NMR (471 MHz, 500 µM, DMSO-d_6/D_2O 9:1)

650 nm adapted → 365 nm irradiation for 15 min

trans:*cis* = 65:35

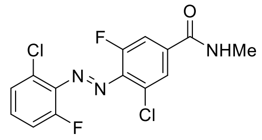

24

^{19}F NMR (471 MHz, 500 μM, DMSO-d_6/D_2O 9:1)

dark-adapted → 740 nm irradiation for 6 h

trans:*cis* = 82:18

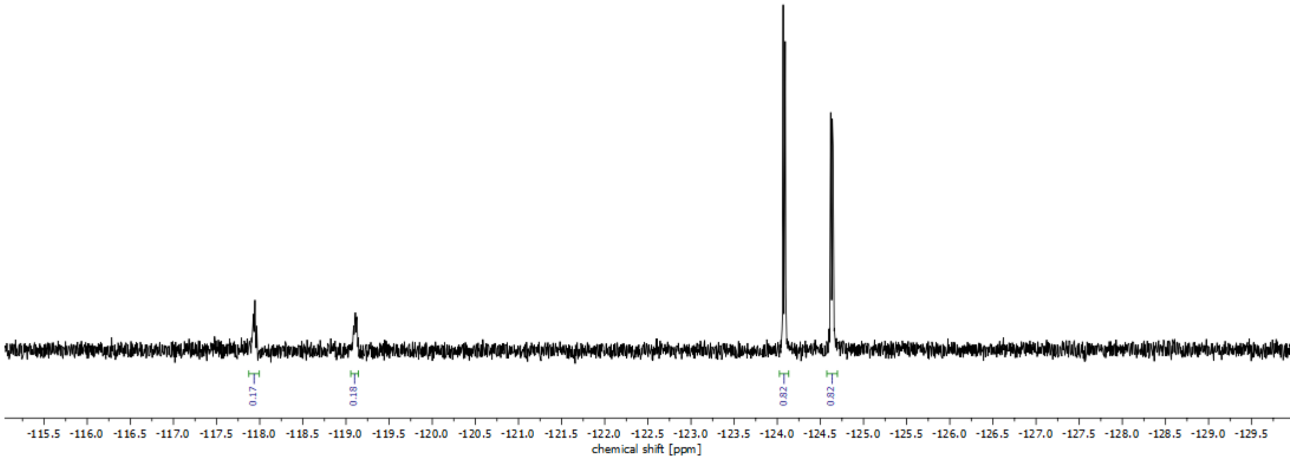

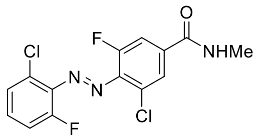

24

^{19}F NMR (471 MHz, 500 μM, DMSO-d_6/D_2O 9:1)

dark-adapted → 740 nm irradiation for 1 d

trans:*cis* = 65:35

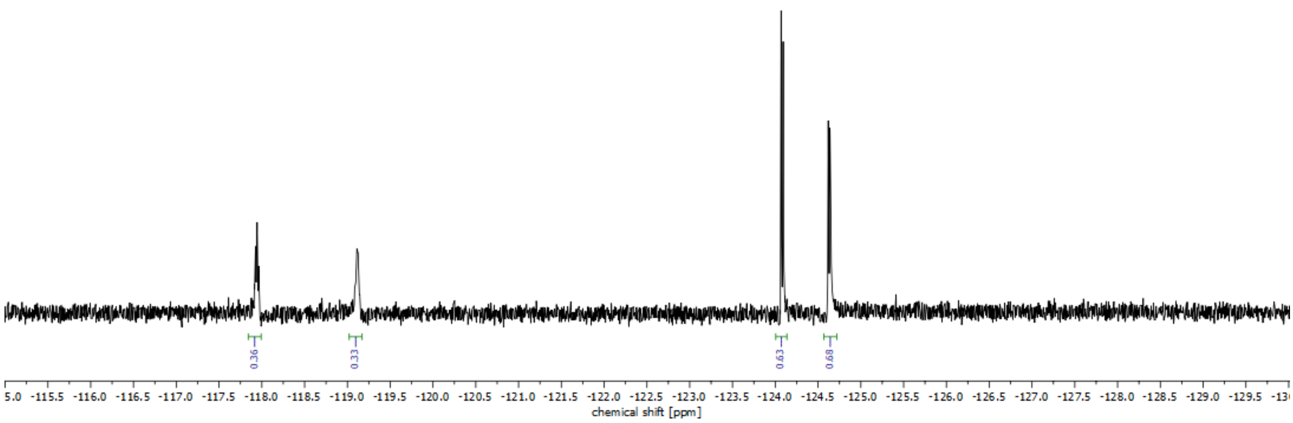

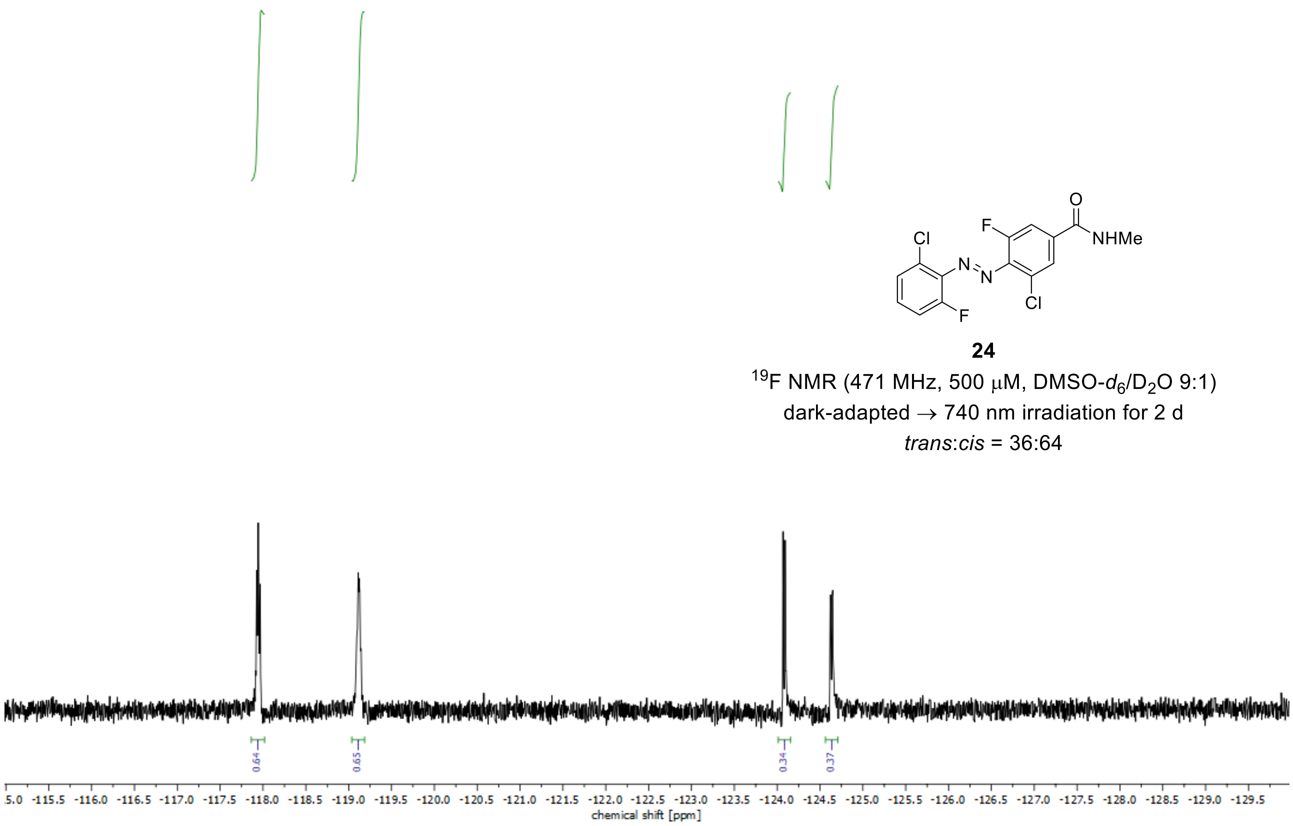

(*E*/*Z*)-Dimethyl 4,4'-(diazene-1,2-diyl)-bis(3-chloro-5-fluorobenzoate) (28, 500 µM, DMSO-d_6/D_2O 9:1)

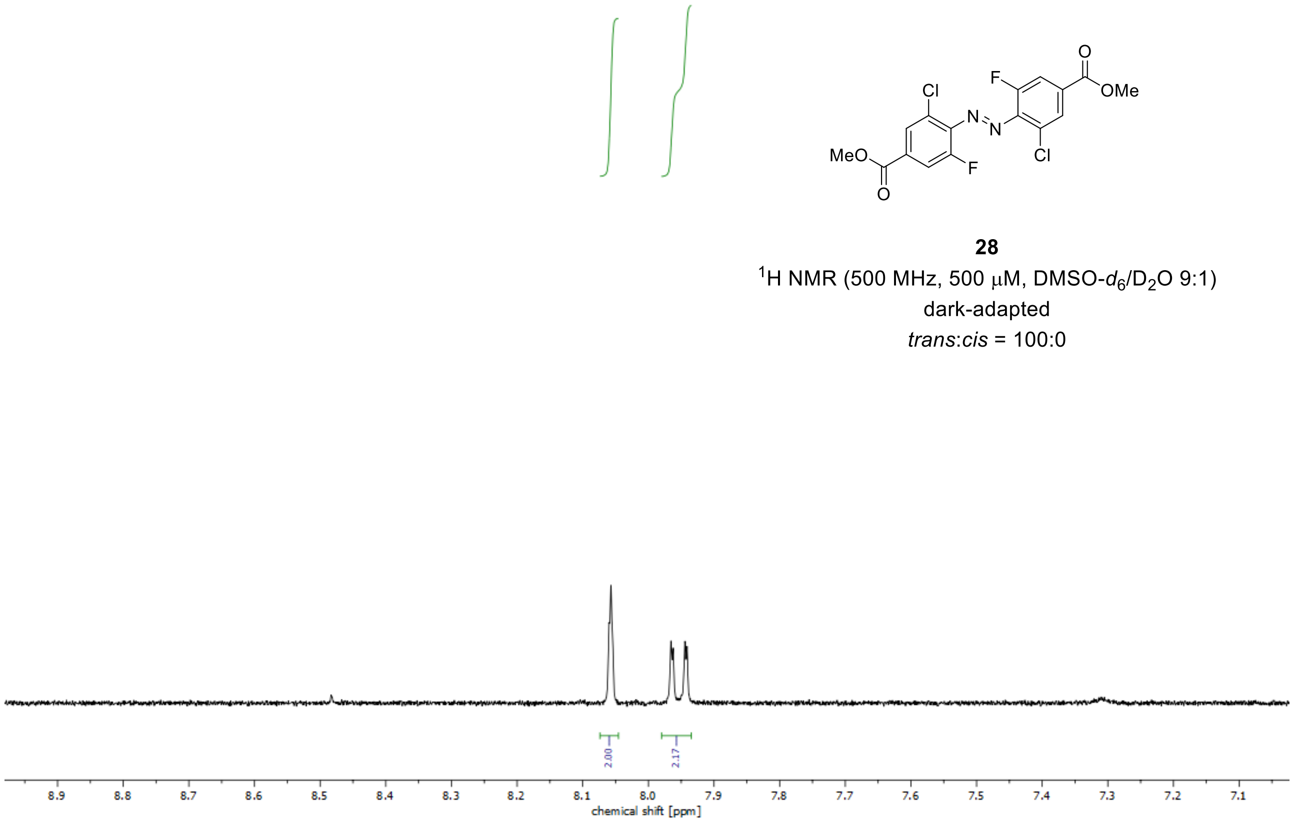

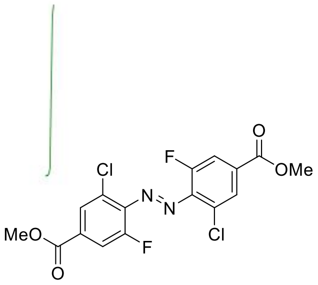

28

^{19}F NMR (471 MHz, 500 μM, DMSO-d_6/D_2O 9:1)

dark-adapted

trans:*cis* = 100:0

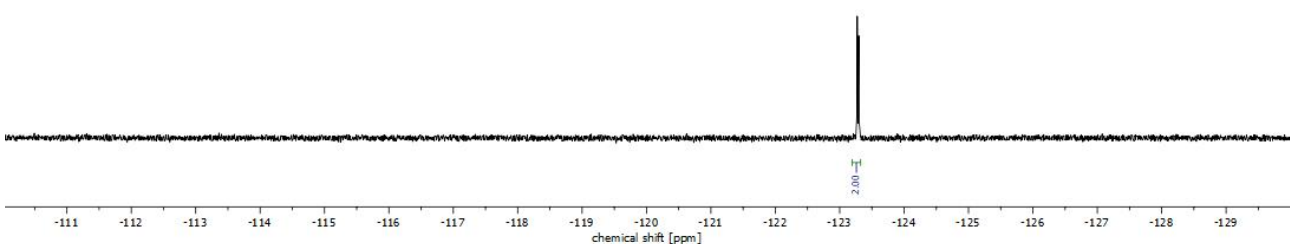

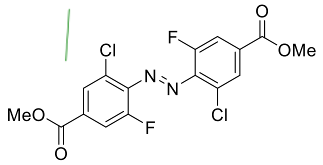

28

^{19}F NMR (471 MHz, 500 μM, DMSO-d_6/D_2O 9:1)

dark-adapted → 650 nm irradiation for 15 min

trans:*cis* = 23:77

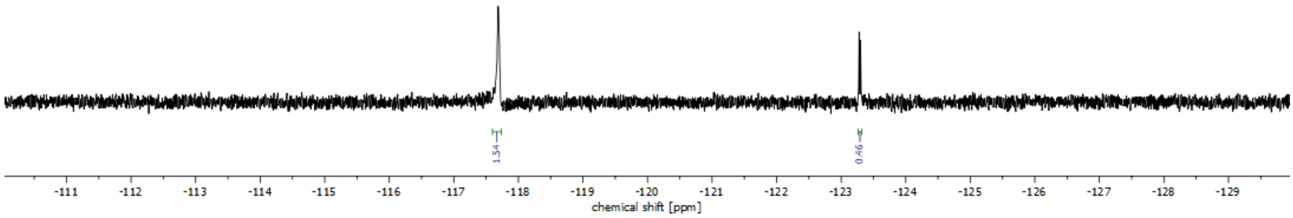

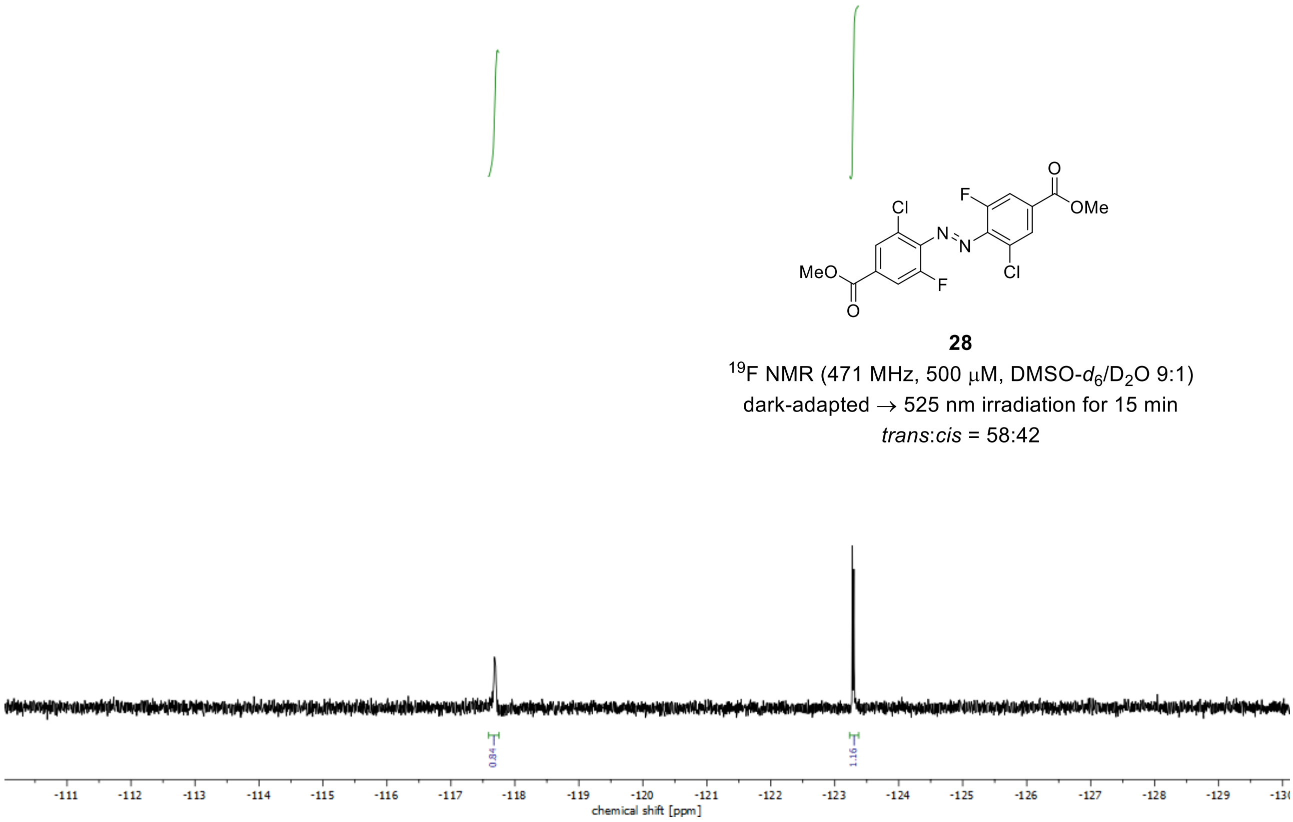
O
F
OMe
Cl
N
N
MeO
F
Cl
O
28
^{19}F NMR (471 MHz, 500 μM, DMSO-d_6/D_2O 9:1)
dark-adapted → 525 nm irradiation for 15 min
trans:*cis* = 58:42
0.84
1.16
chemical shift [ppm]

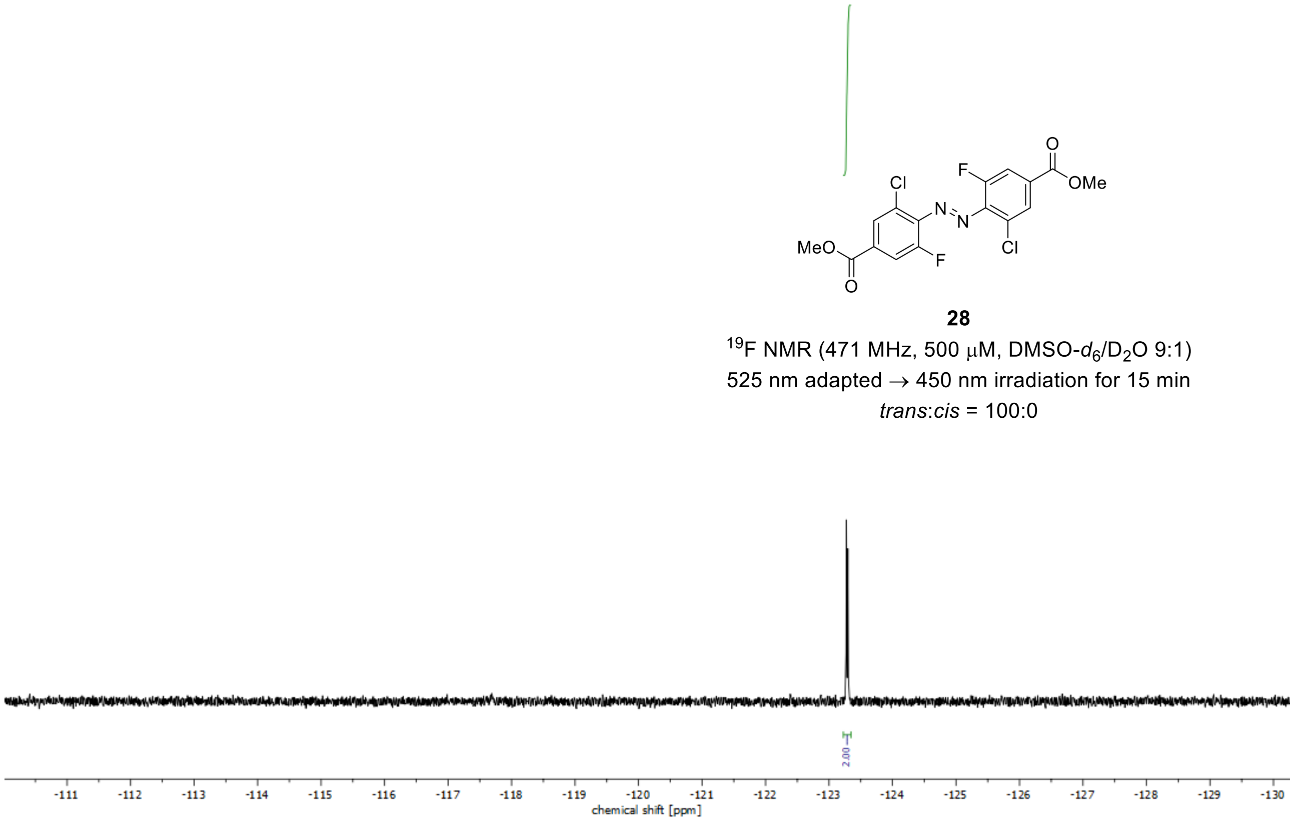
O
F
OMe
Cl
N
N
MeO
F
Cl
O
28
^{19}F NMR (471 MHz, 500 μM, DMSO-d_6/D_2O 9:1)
525 nm adapted → 450 nm irradiation for 15 min
trans:*cis* = 100:0
2.00
chemical shift [ppm]

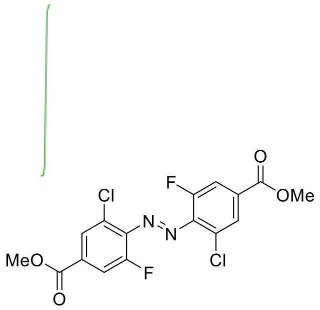

28

^{19}F NMR (471 MHz, 500 μM, DMSO-d_6/D_2O 9:1)
650 nm adapted → 365 nm irradiation for 15 min
trans:*cis* = 100:0

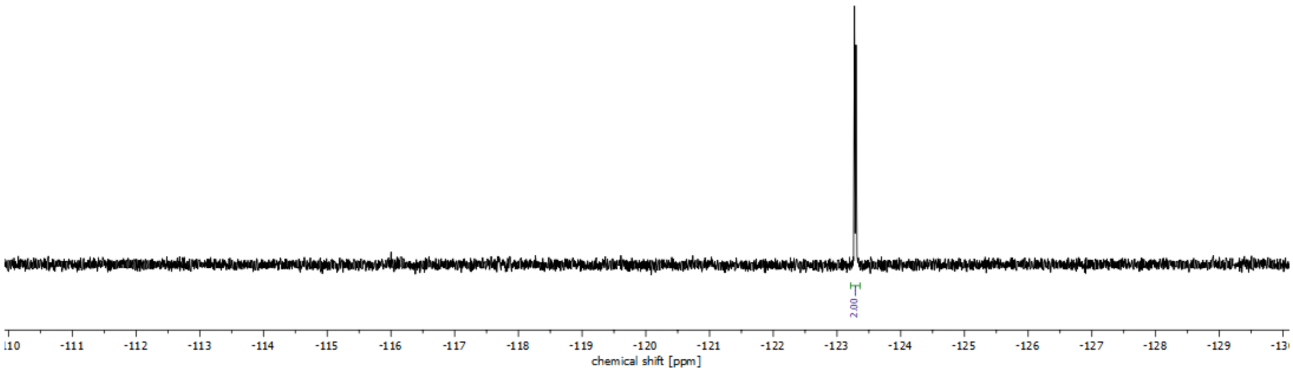

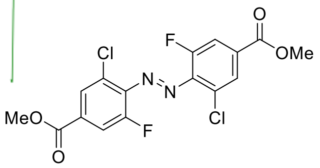

28

^{19}F NMR (471 MHz, 500 μM, DMSO-d_6/D_2O 9:1)
dark-adapted → 740 nm irradiation for 1 d
trans:*cis* = 48:52

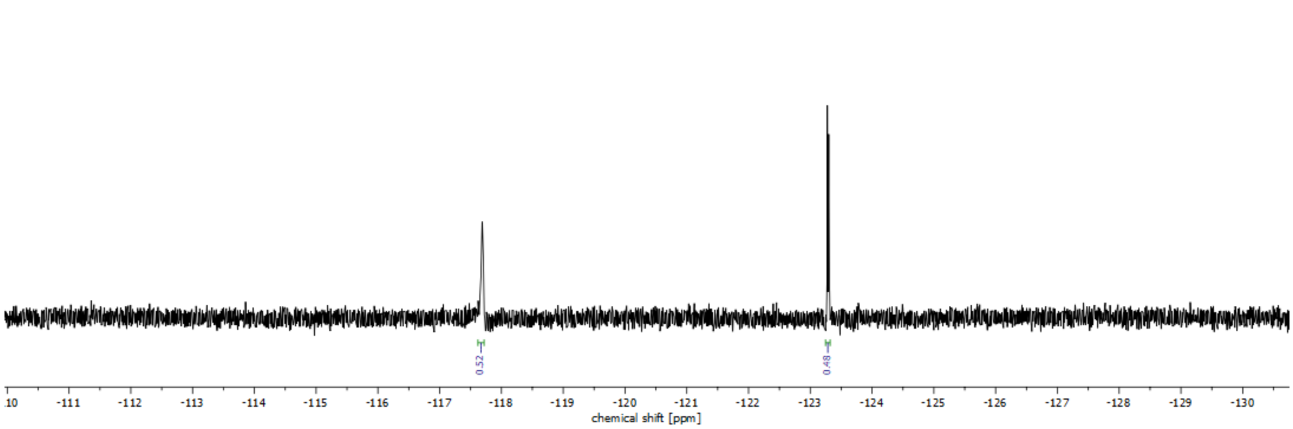

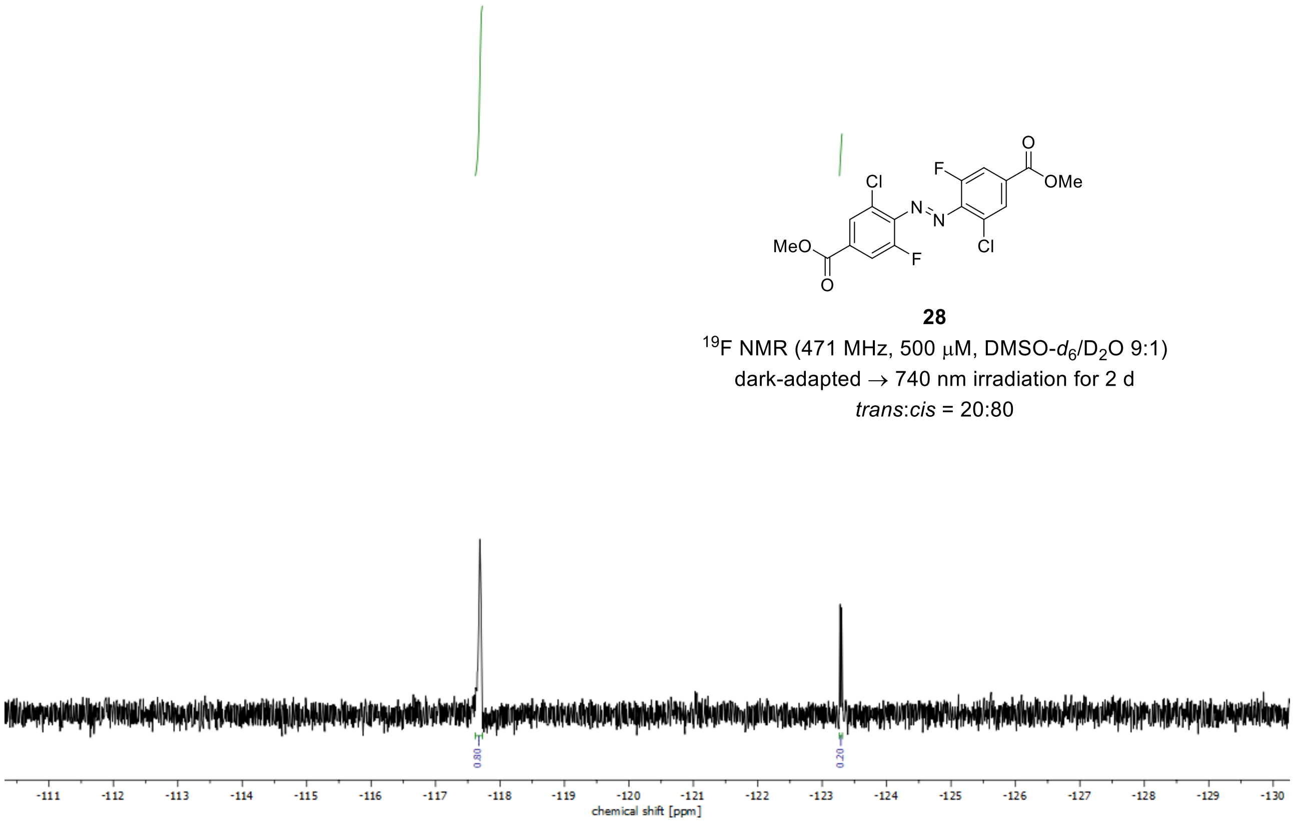

(*E*/*Z*)-3-Chloro-4-((2-chloro-6-fluorophenyl)diazenyl)-5-fluoroaniline (38, 500 μM, DMSO-d_6/D_2O 9:1)

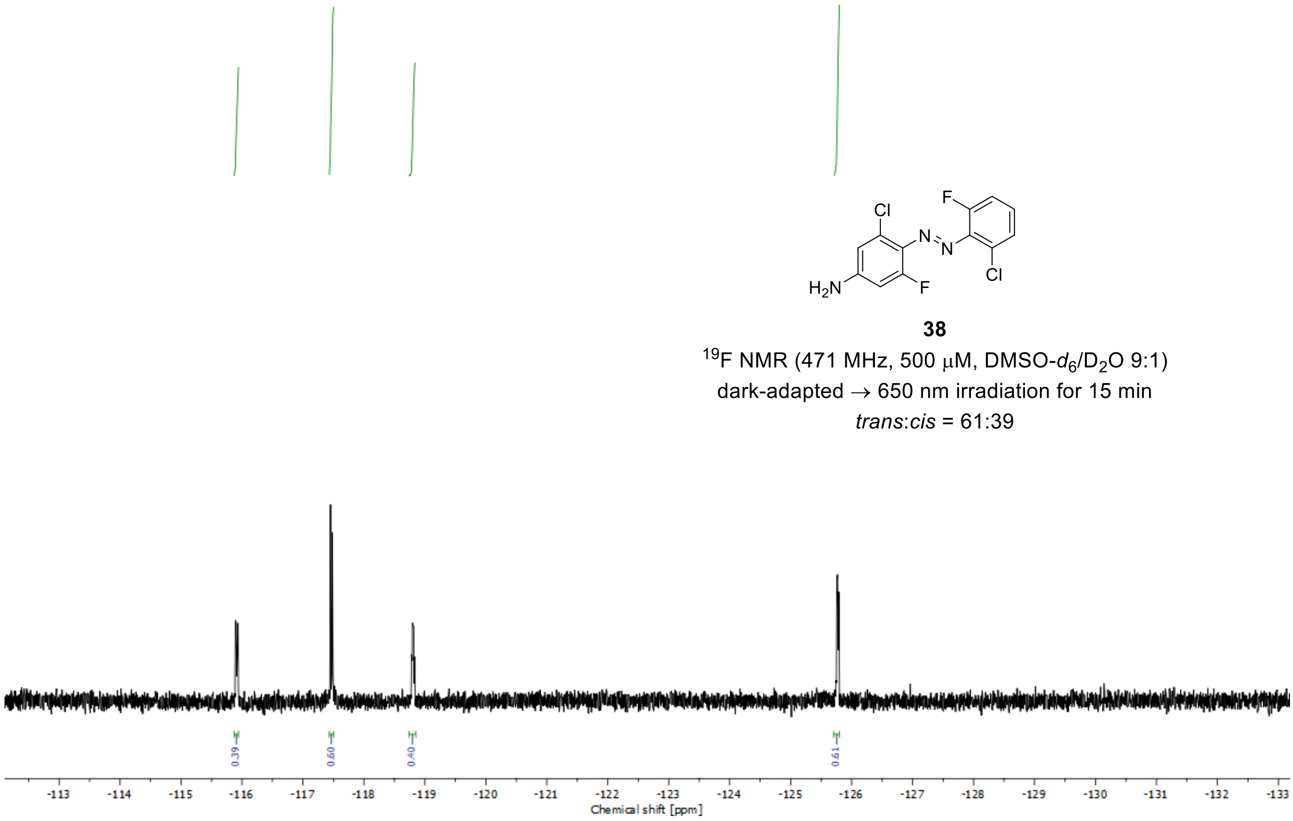

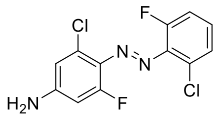

38

^{19}F NMR (471 MHz, 500 μM, DMSO-d_6/D_2O 9:1)
dark-adapted → 650 nm irradiation for 30 min
trans:*cis* = 53:47

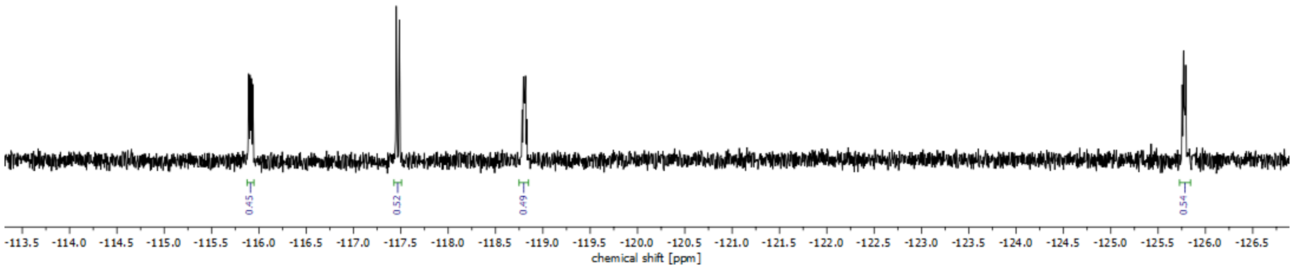

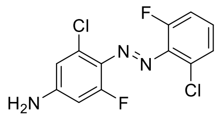

38

^{19}F NMR (471 MHz, 500 μM, DMSO-d_6/D_2O 9:1)
dark-adapted → 650 nm irradiation for 60 min
trans:*cis* = 50:50

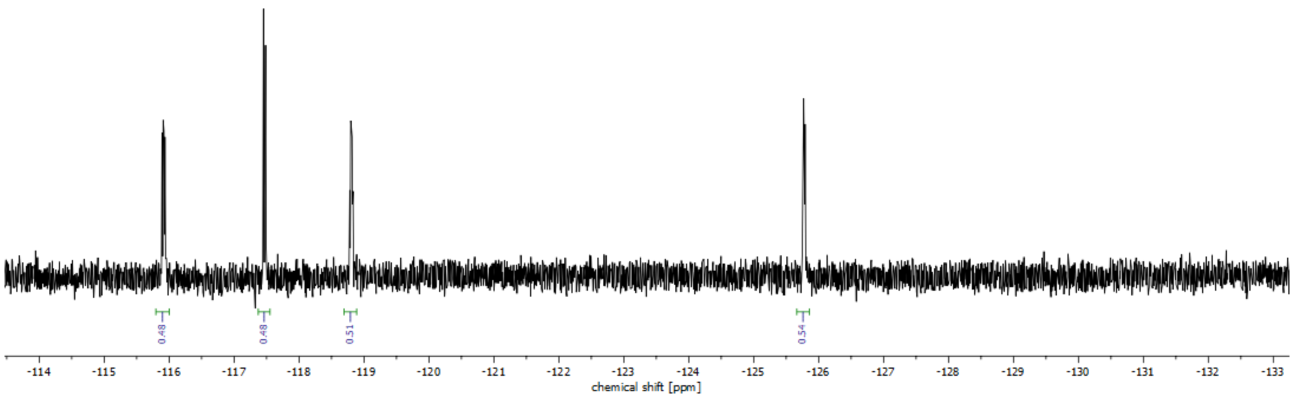

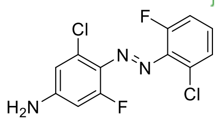

38

^{19}F NMR (471 MHz, 500 μM, DMSO-d_6/D_2O 9:1)

dark-adapted → 650 nm irradiation for 120 min

trans:*cis* = 51:49

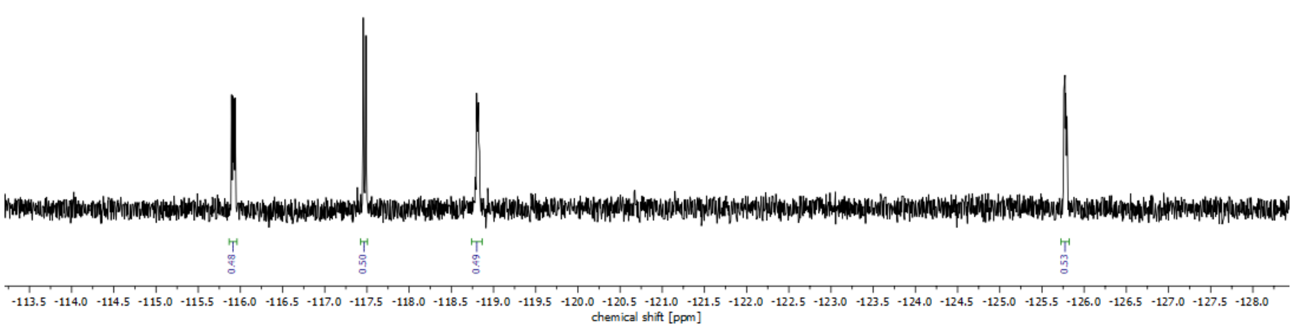

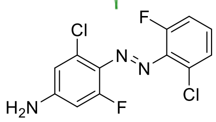

38

^{19}F NMR (471 MHz, 500 μM, DMSO-d_6/D_2O 9:1)

dark-adapted → 525 nm irradiation for 15 min

trans:*cis* = 65:35

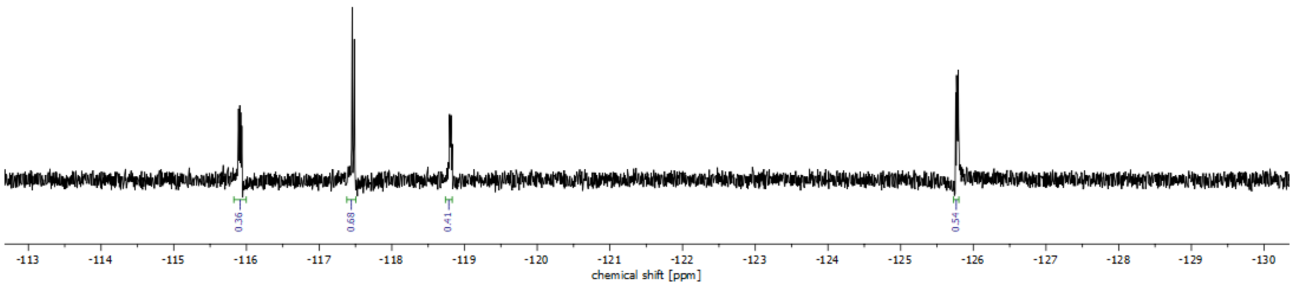

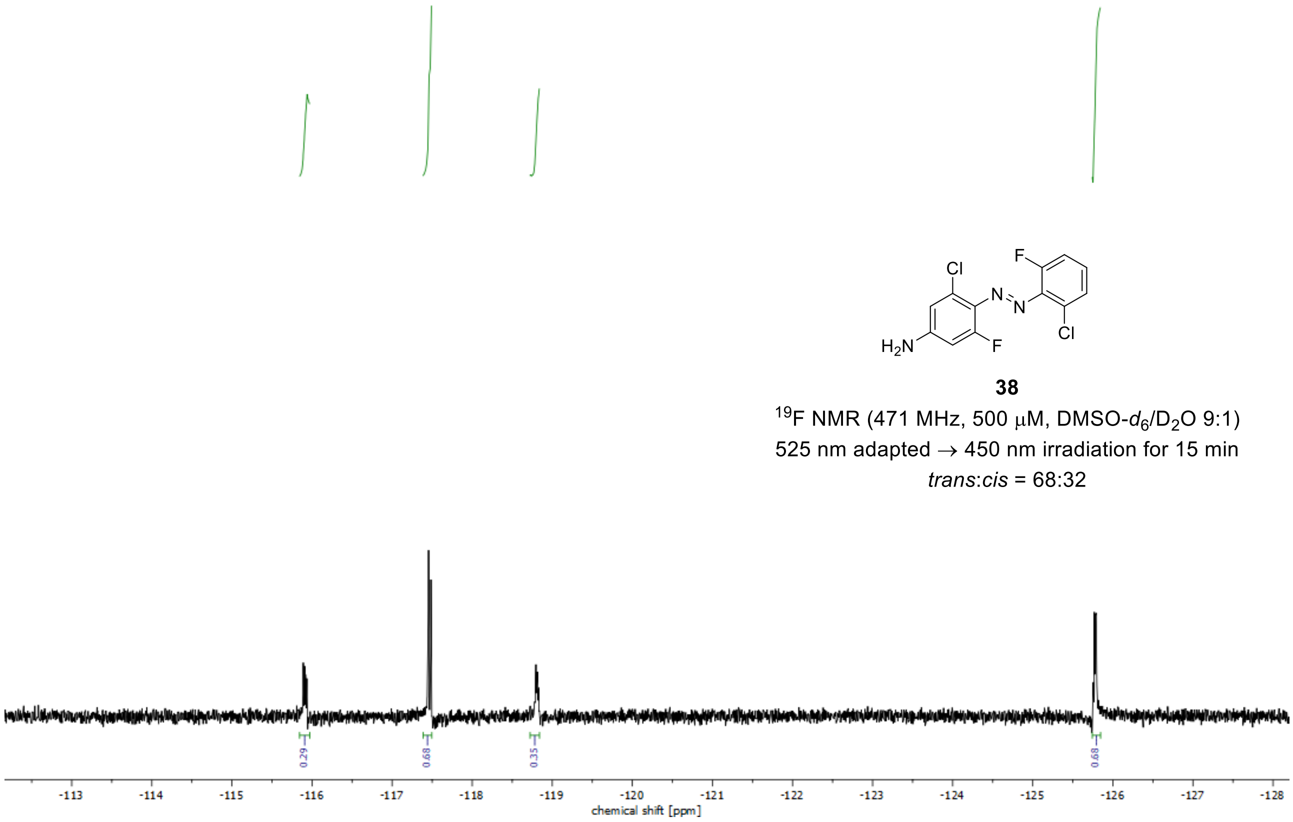

38

^{19}F NMR (471 MHz, 500 μM, DMSO-d_6/D_2O 9:1)

525 nm adapted → 450 nm irradiation for 15 min

trans:*cis* = 68:32

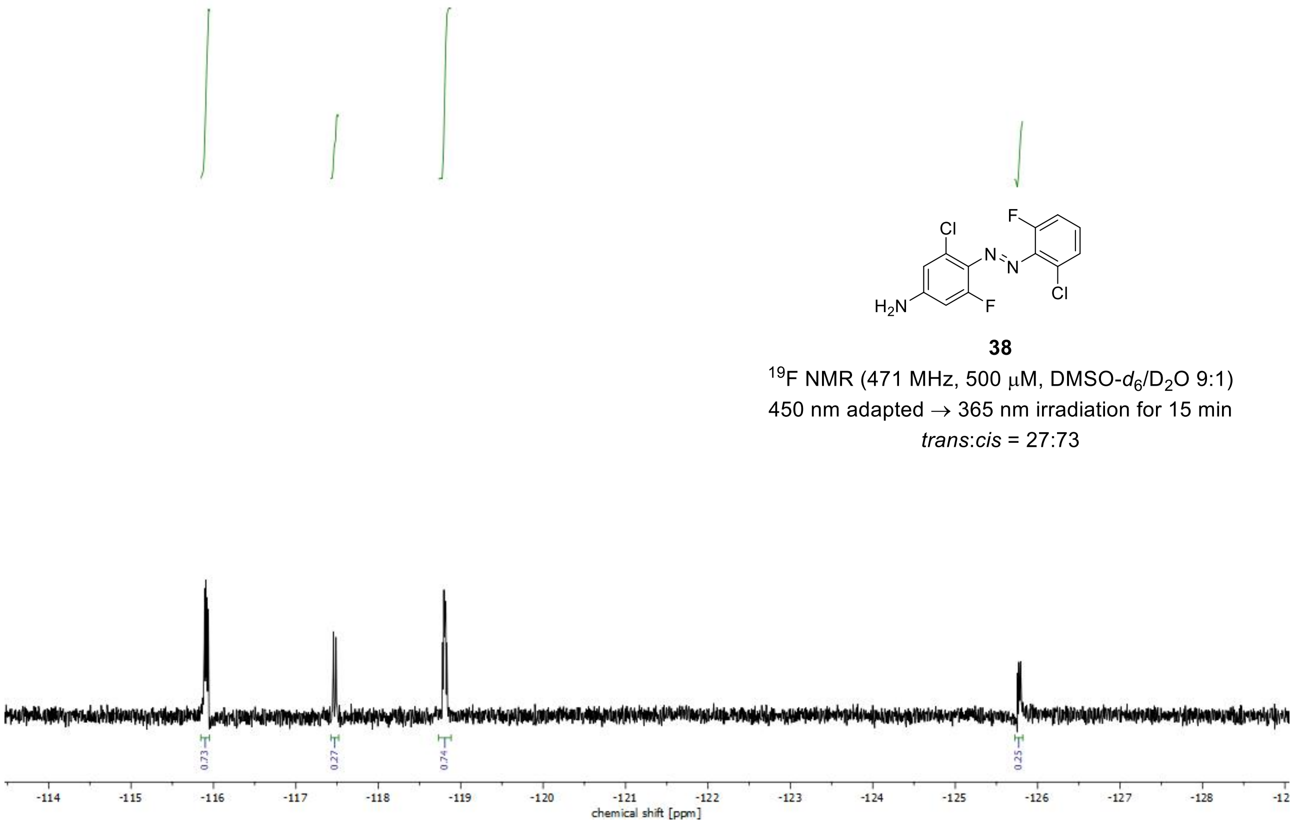

38

^{19}F NMR (471 MHz, 500 μM, DMSO-d_6/D_2O 9:1)

450 nm adapted → 365 nm irradiation for 15 min

trans:*cis* = 27:73

(*E*/*Z*)-*N*-(3-chloro-4-((2-chloro-6-fluorophenyl)diazenyl)-5-fluorophenyl)acetamide (39, 500 µM, DMSO-d_6/D_2O 9:1)

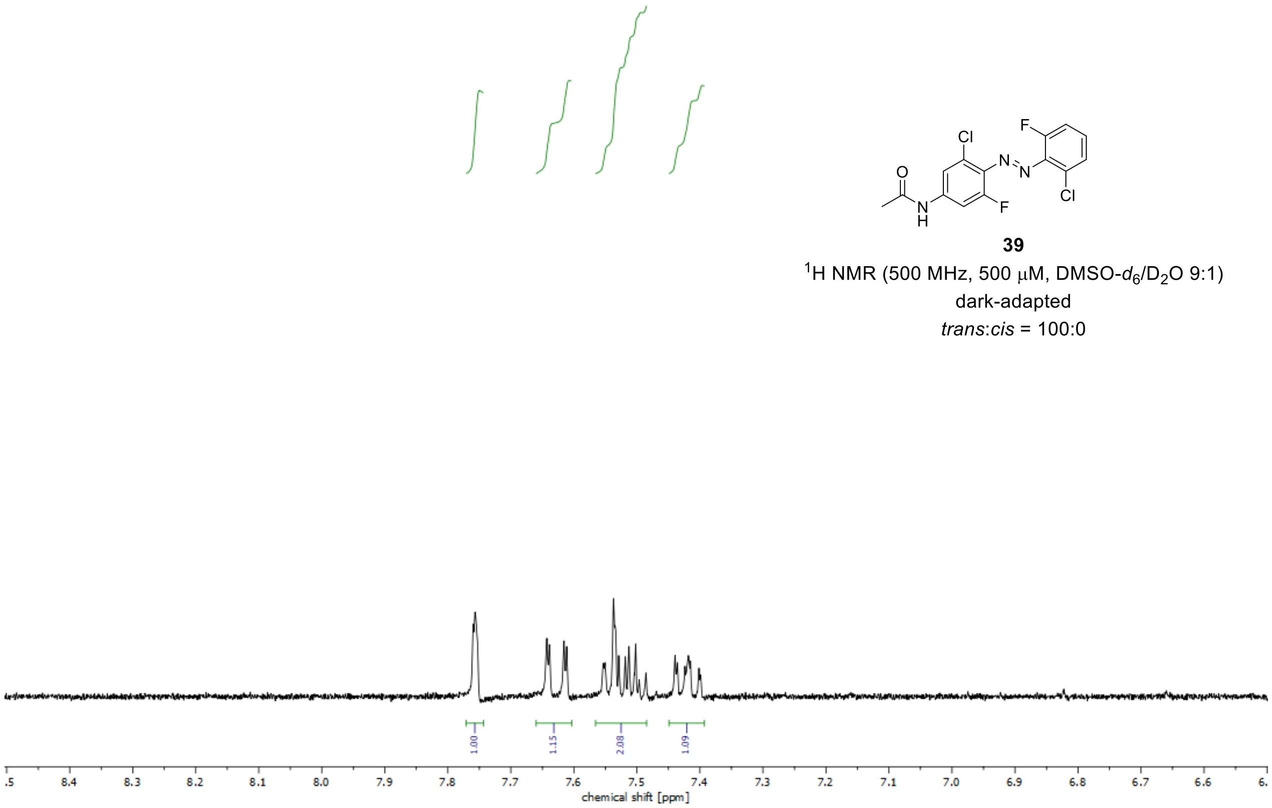

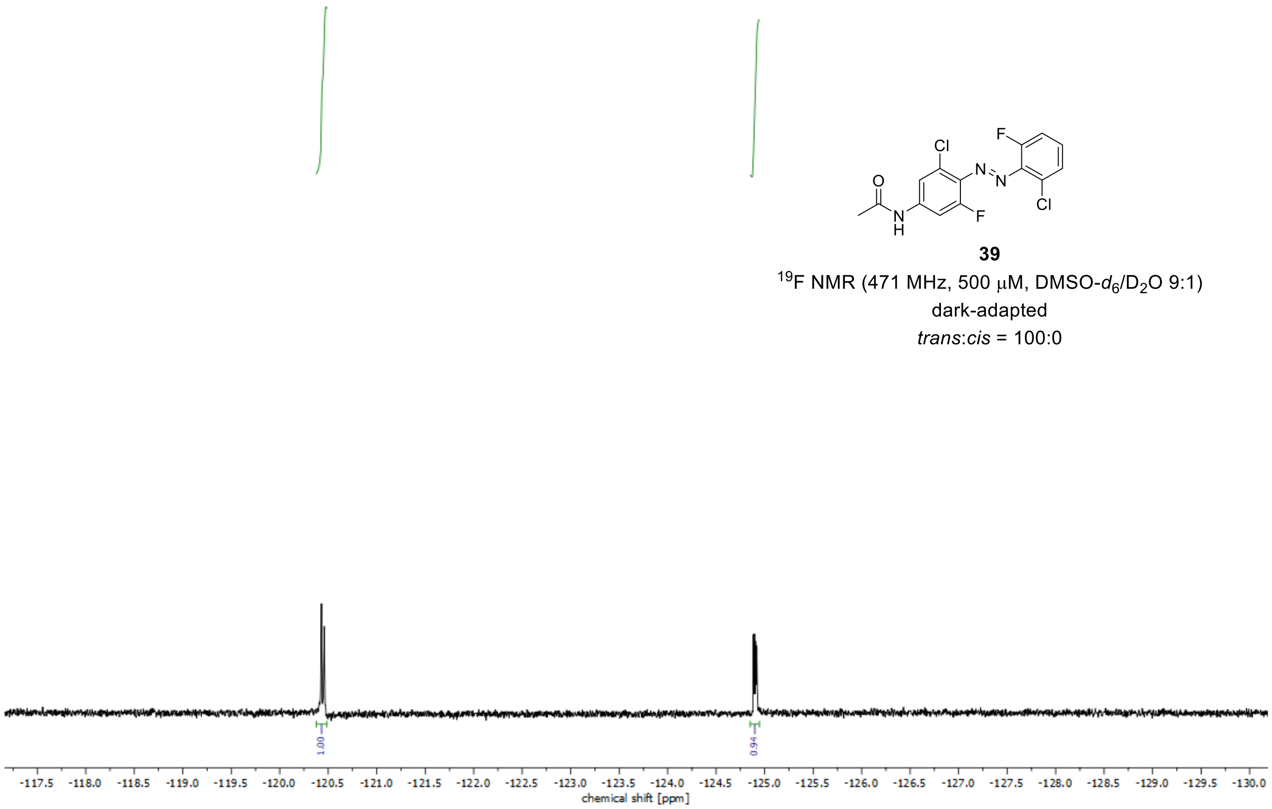

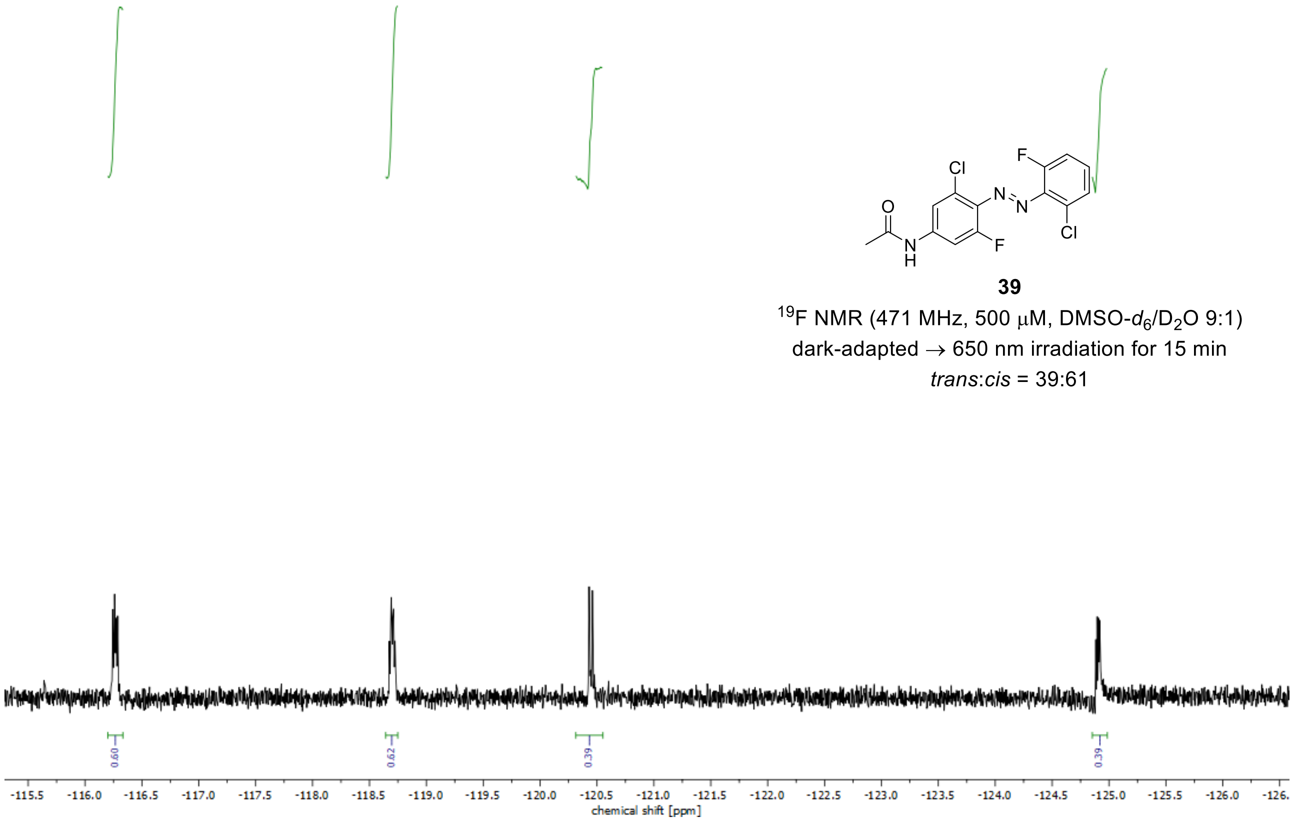

39

^{19}F NMR (471 MHz, 500 μM, DMSO-d_6/D_2O 9:1)

dark-adapted → 650 nm irradiation for 15 min

trans:*cis* = 39:61

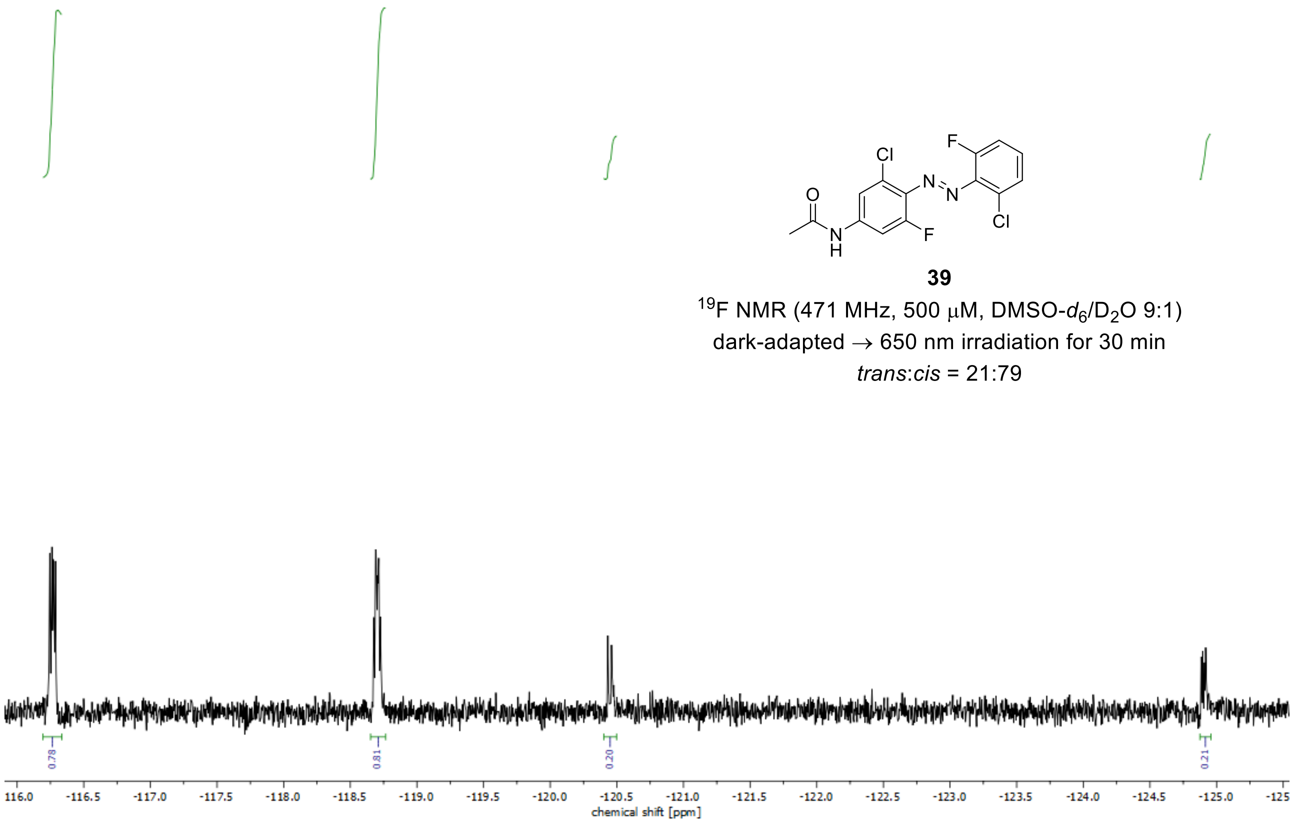

39

^{19}F NMR (471 MHz, 500 μM, DMSO-d_6/D_2O 9:1)

dark-adapted → 650 nm irradiation for 30 min

trans:*cis* = 21:79

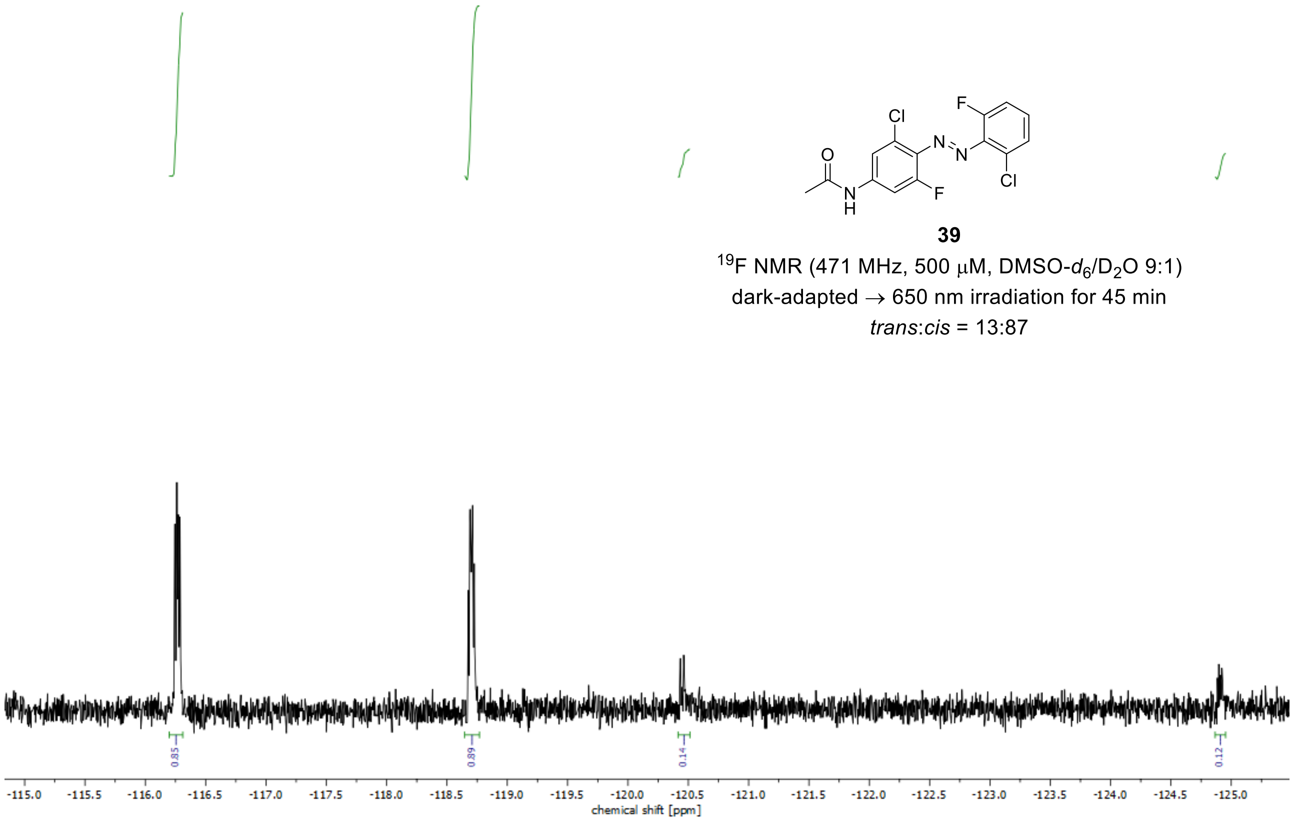
39
^{19}F NMR (471 MHz, 500 μM, DMSO-d_6/D_2O 9:1)
dark-adapted → 650 nm irradiation for 45 min
trans:*cis* = 13:87
0.85
0.89
0.14
0.12
chemical shift [ppm]

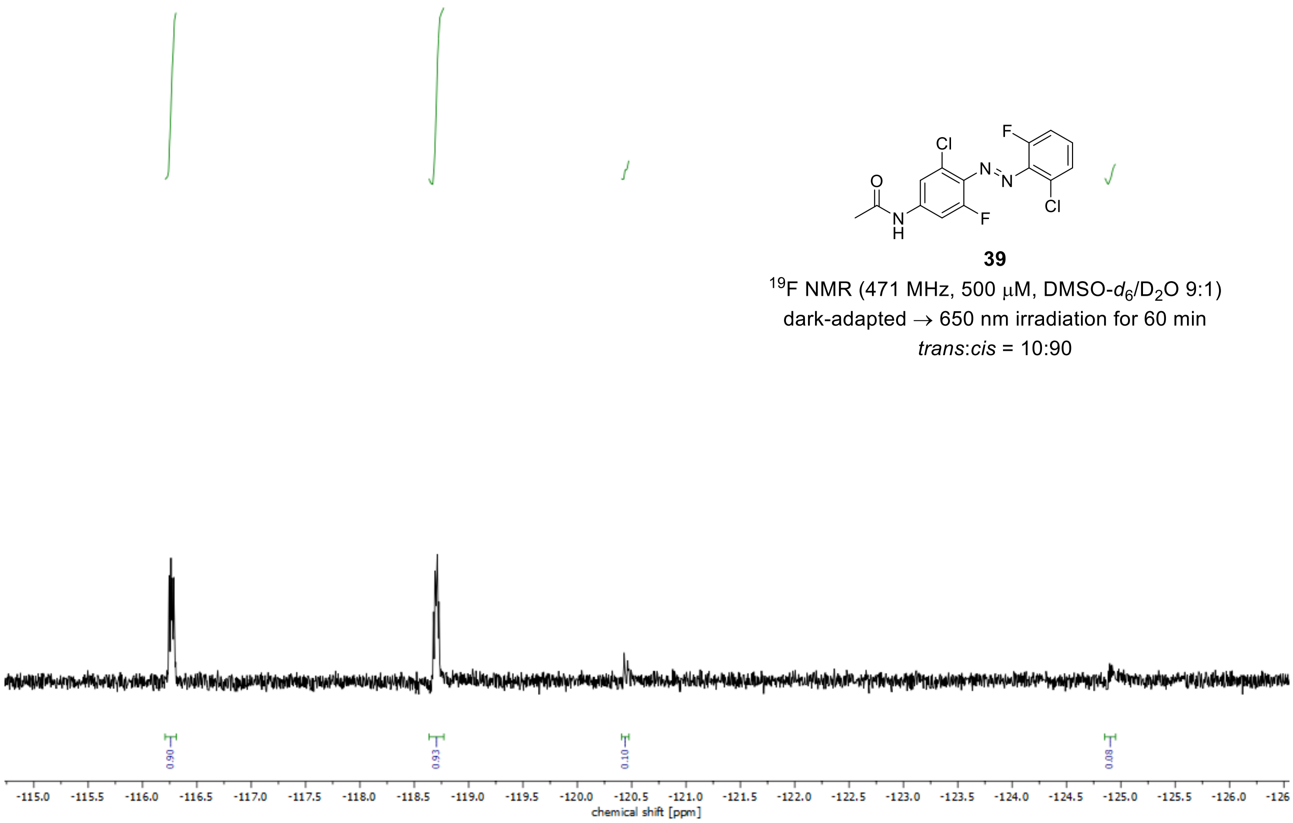
39
^{19}F NMR (471 MHz, 500 μM, DMSO-d_6/D_2O 9:1)
dark-adapted → 650 nm irradiation for 60 min
trans:*cis* = 10:90
0.90
0.93
0.10
0.08
chemical shift [ppm]

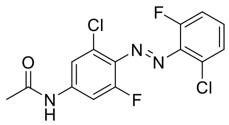

39

^{19}F NMR (471 MHz, 500 μM, DMSO-d_6/D_2O 9:1)
650 nm-adapted → 450 nm irradiation for 15 min
trans:*cis* = 77:23

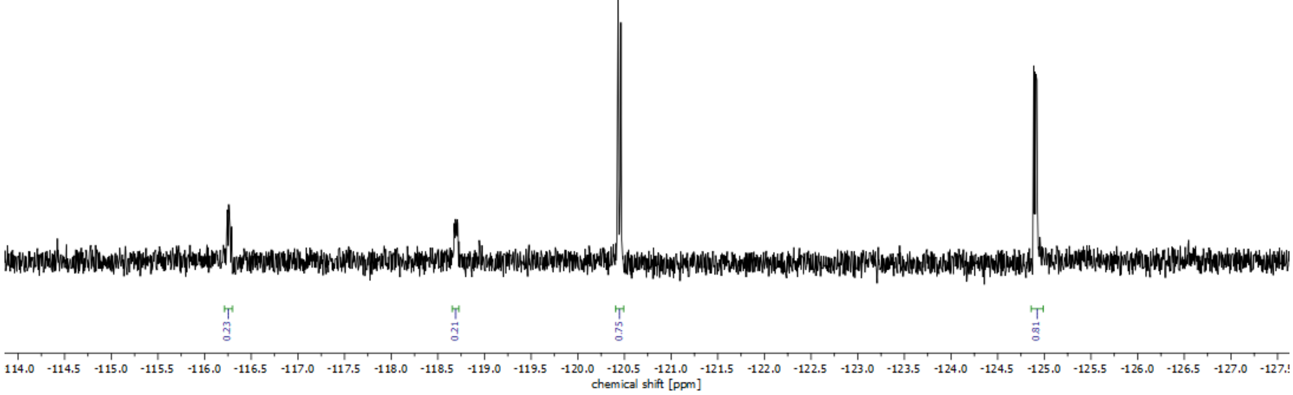

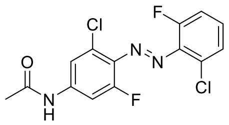

39

^{19}F NMR (471 MHz, 500 μM, DMSO-d_6/D_2O 9:1)
450 nm-adapted → 525 nm irradiation for 15 min
trans:*cis* = 25:75

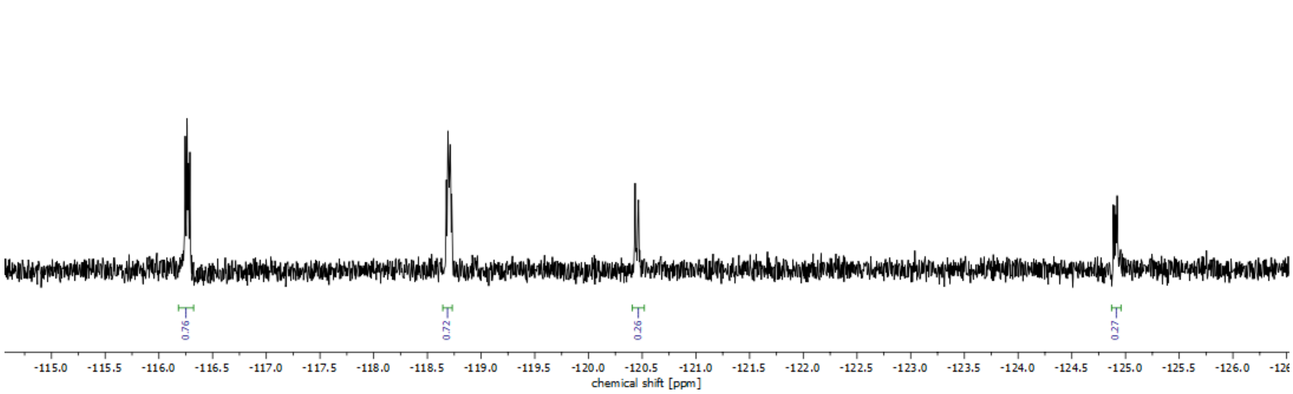

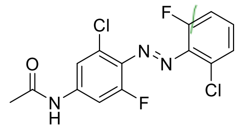

39

^{19}F NMR (471 MHz, 500 μM, DMSO-d_6/D_2O 9:1)

525 nm-adapted → 365 nm irradiation for 15 min

trans:*cis* = 10:90

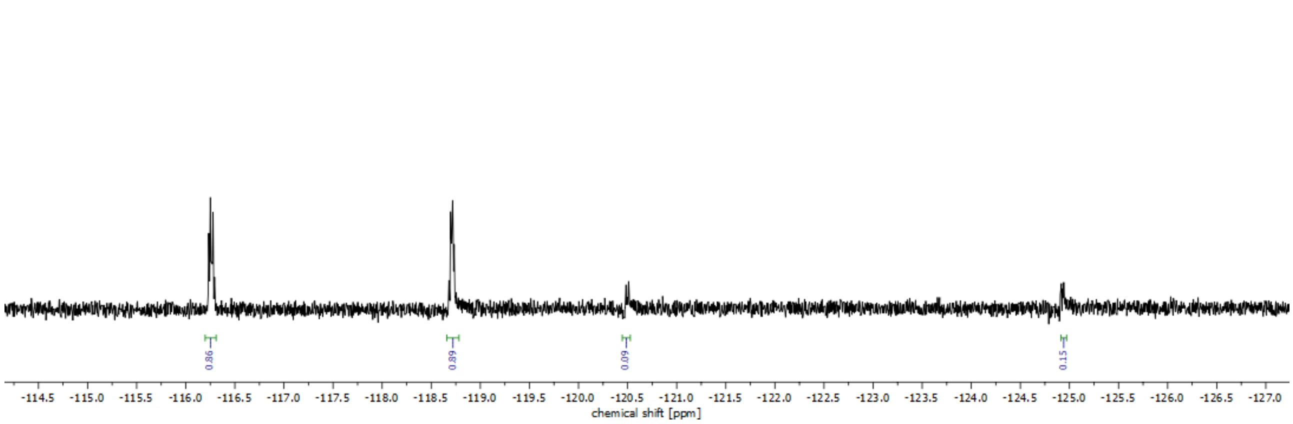

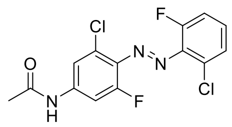

39

^{19}F NMR (471 MHz, 500 μM, DMSO-d_6/D_2O 9:1)

dark-adapted → 740 nm irradiation for 1 d

trans:*cis* = 71:29

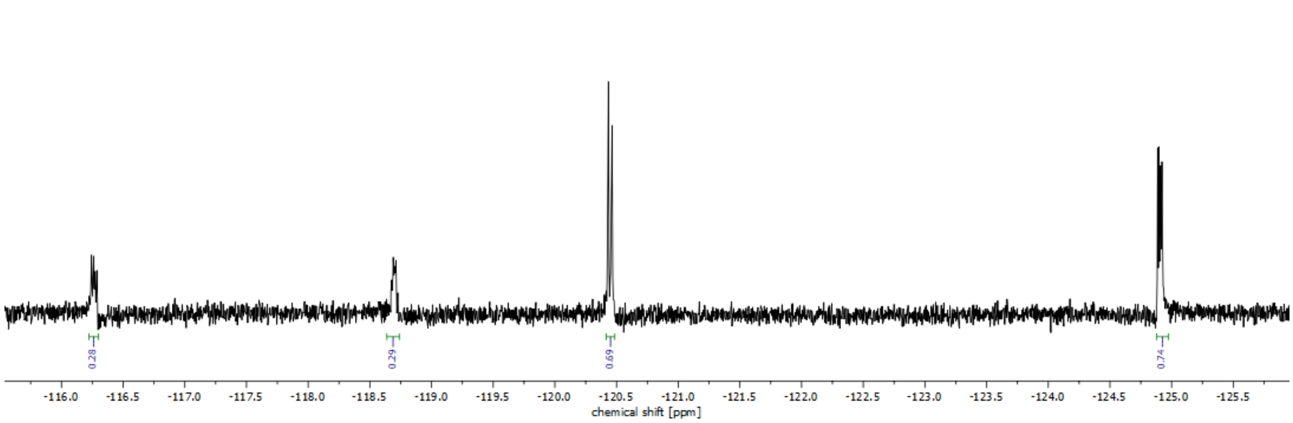

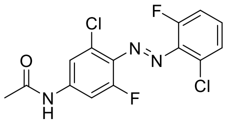

39

^{19}F NMR (471 MHz, 500 μM, DMSO-d_6/D_2O 9:1)
dark-adapted → 740 nm irradiation for 2 d
trans:*cis* = 64:36

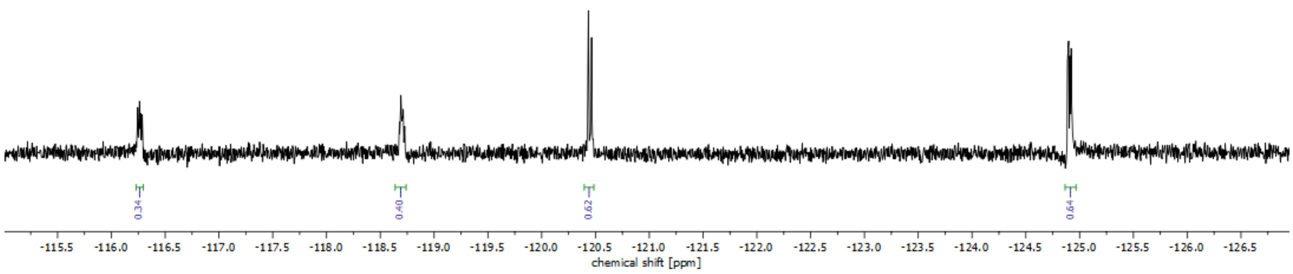

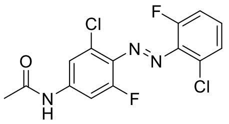

39

^{19}F NMR (471 MHz, 500 μM, DMSO-d_6/D_2O 9:1)
dark-adapted → 740 nm irradiation for 3 d
trans:*cis* = 57:43

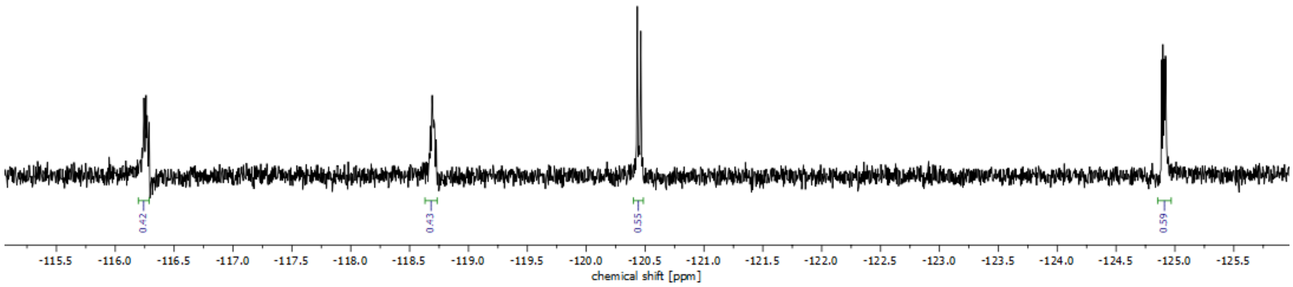

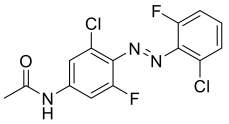

39

^{19}F NMR (471 MHz, 500 μM, DMSO-d_6/D_2O 9:1)

dark-adapted → 740 nm irradiation for 4 d

trans:*cis* = 52:48

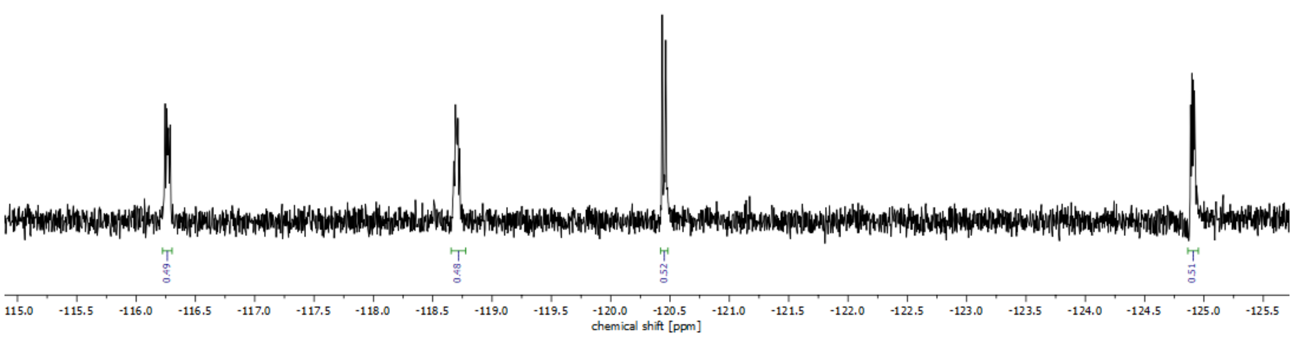

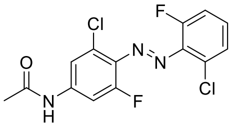

39

^{19}F NMR (471 MHz, 500 μM, DMSO-d_6/D_2O 9:1)

dark-adapted → 740 nm irradiation for 5 d

trans:*cis* = 47:53

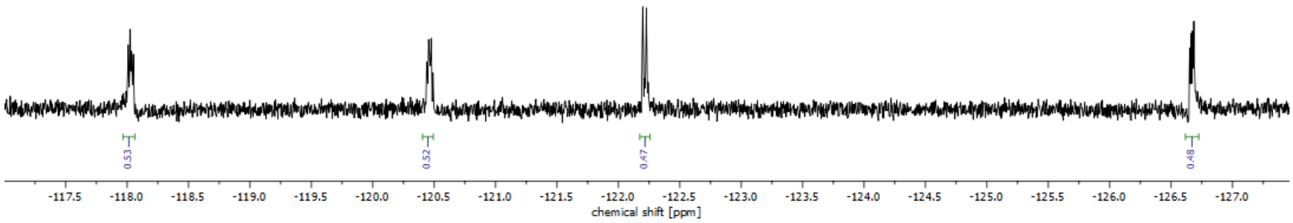

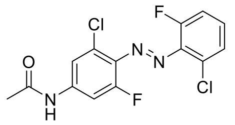

39

^{19}F NMR (471 MHz, 500 μM, DMSO-d_6/D_2O 9:1)
dark-adapted → 740 nm irradiation for 7 d
trans:*cis* = 43:57

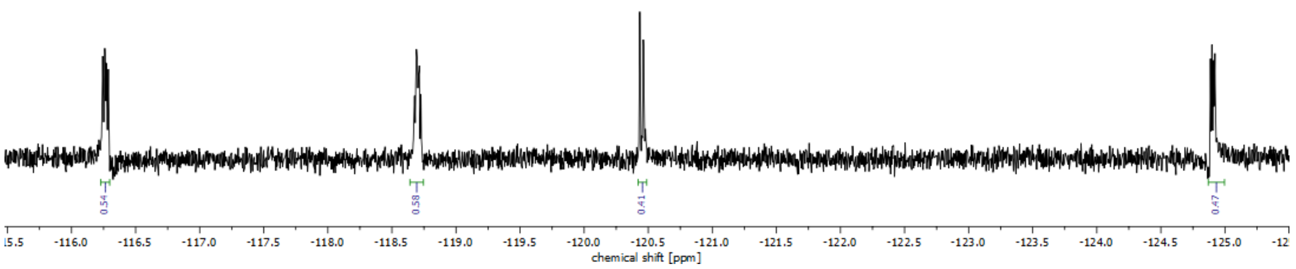

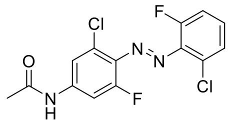

39

^{19}F NMR (471 MHz, 500 μM, DMSO-d_6/D_2O 9:1)
dark-adapted → 740 nm irradiation for 8 d
trans:*cis* = 41:59

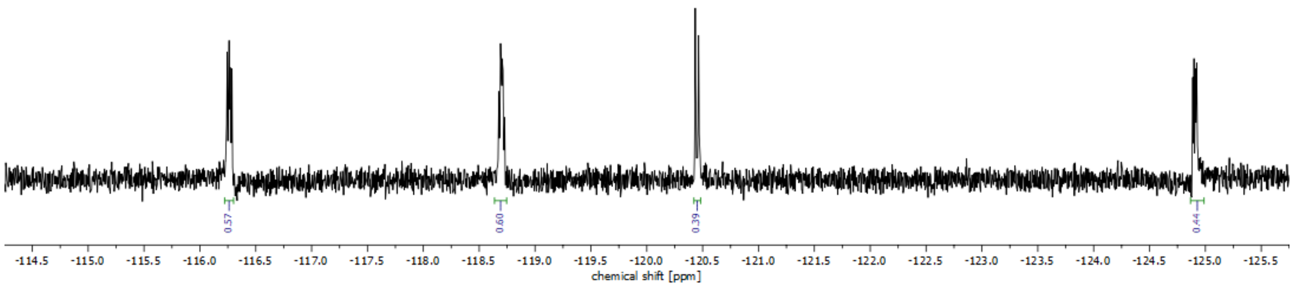

(*E*/*Z*)-Methyl-3-chloro-4-((2-chloro-6-fluoro-4-methoxyphenyl)diazenyl)-5-fluorobenzoate (46, 500 µM, DMSO-d_6/D_2O 9:1)

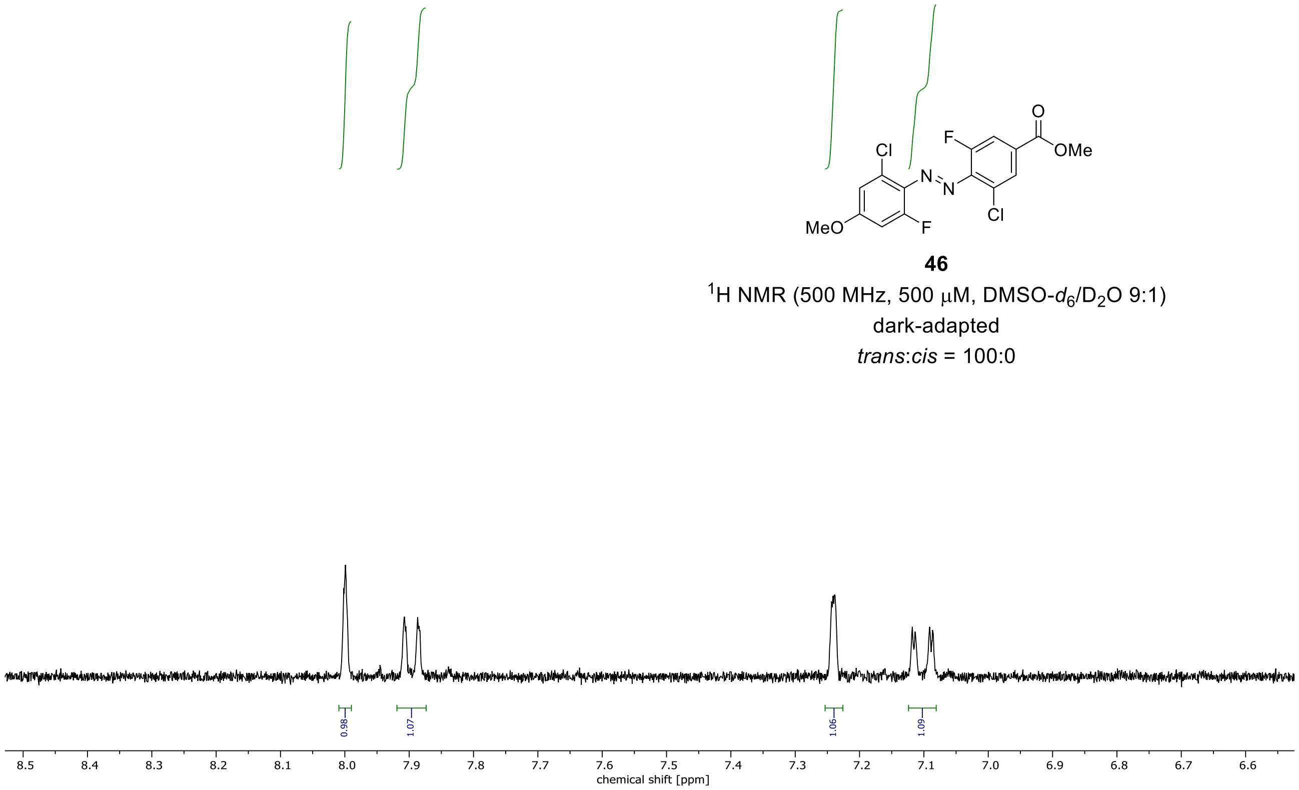

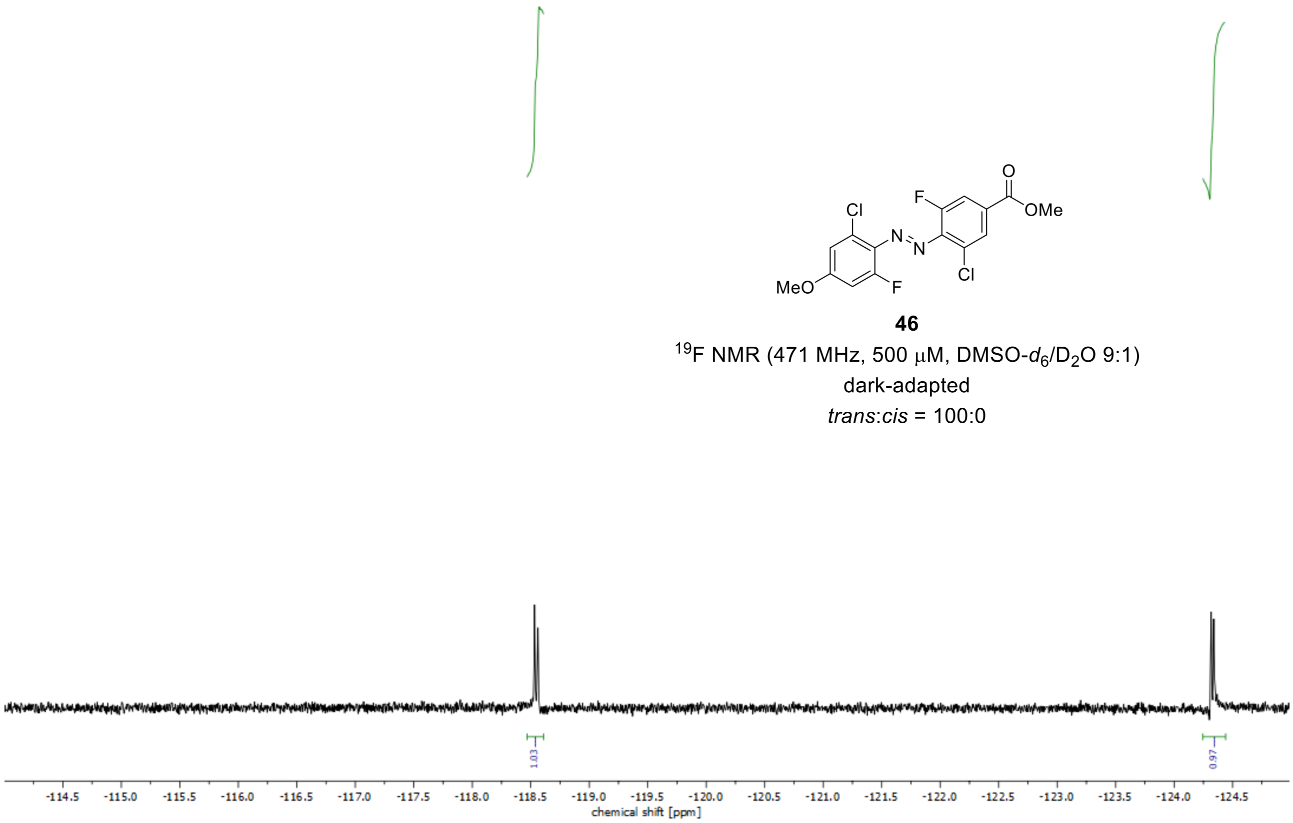

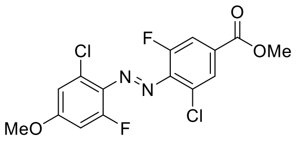

46

^{19}F NMR (471 MHz, 500 μM, DMSO-d_6/D_2O 9:1)

dark-adapted → 650 nm irradiation for 15 min

trans:*cis* = 7:93

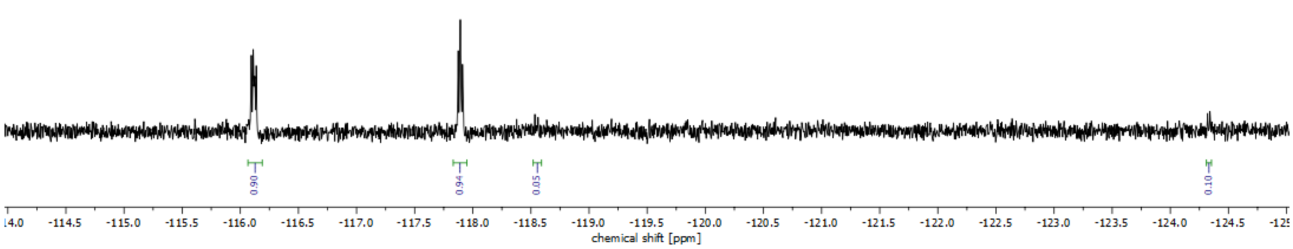

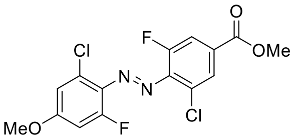

46

^{19}F NMR (471 MHz, 500 μM, DMSO-d_6/D_2O 9:1)

650nm adapted → 525 nm irradiation for 15 min

trans:*cis* = 38:62

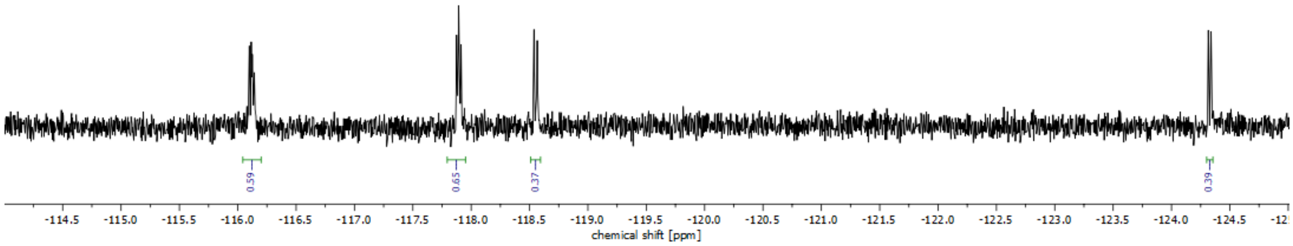

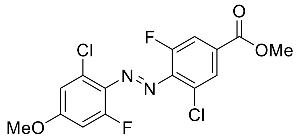

46

^{19}F NMR (471 MHz, 500 μM, DMSO-d_6/D_2O 9:1)

525 nm adapted → 450 nm irradiation for 15 min

trans:*cis* = 84:16

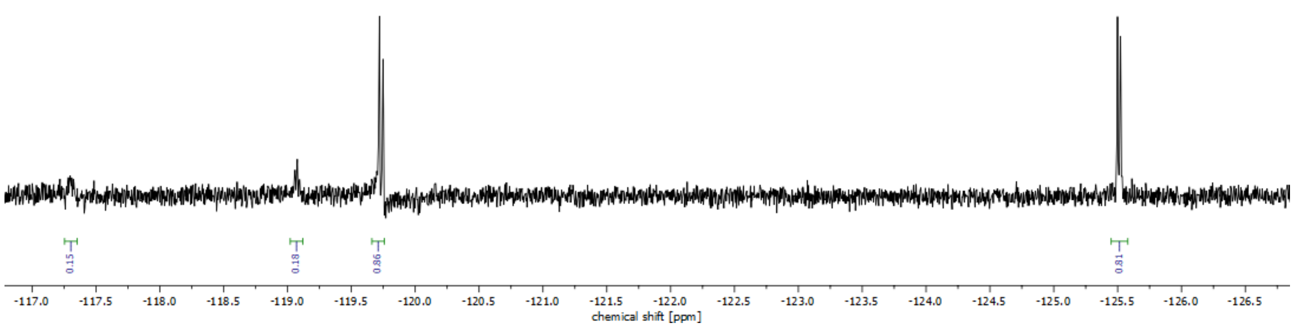

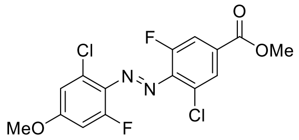

46

^{19}F NMR (471 MHz, 500 μM, DMSO-d_6/D_2O 9:1)

450 nm adapted → 365 nm irradiation for 15 min

trans:*cis* = 19:81

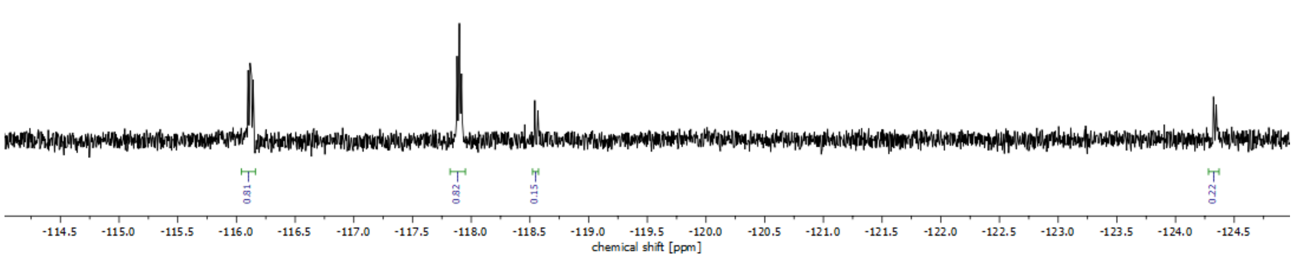

(*E*/*Z*)-1-(1-(4-(4-((4-Butyl-2-chloro-6-fluorophenyl)diazenyl)-3-chloro-5-fluorophenyl)butanoyl)-piperidin-4-yl)-1,3-dihydro-2*H*-benzo[*d*]imidazol-2-one (dfdc-OptoBI-1, 500 μM, DMSO-d_6/D_2O 9:1)

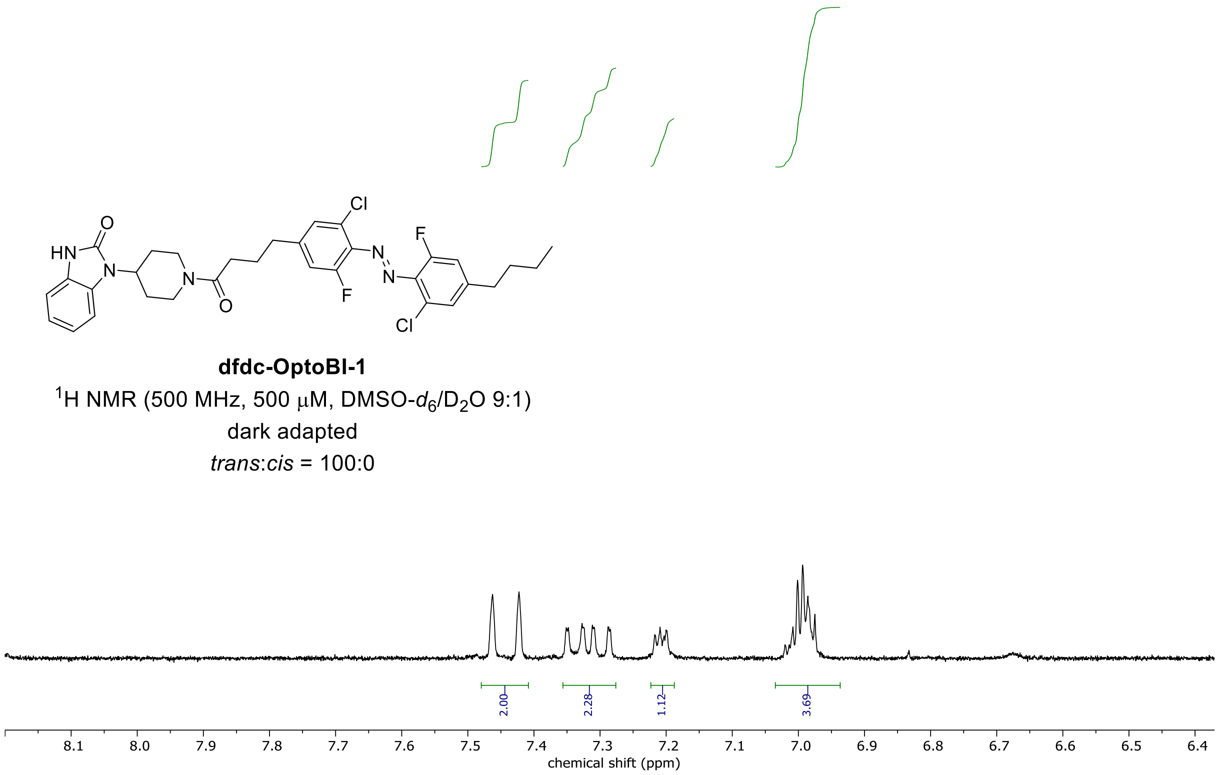

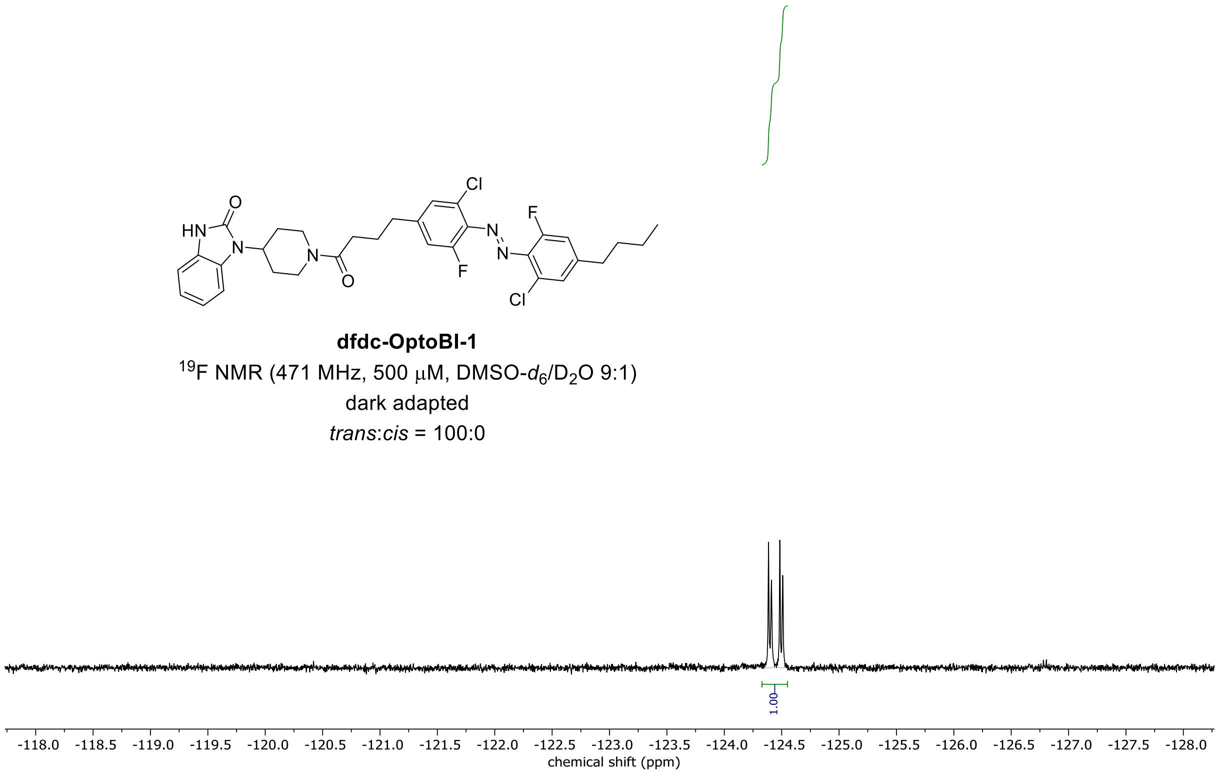

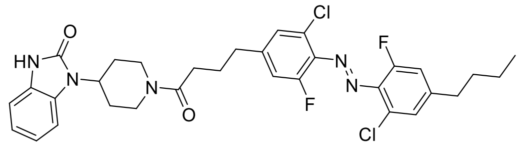

dfdc-OptoBI-1

^{19}F NMR (471 MHz, 500 μM, DMSO-d_6/D_2O 9:1)

dark-adapted → 650 nm irradiation for 15 min

trans:*cis* = 58:42

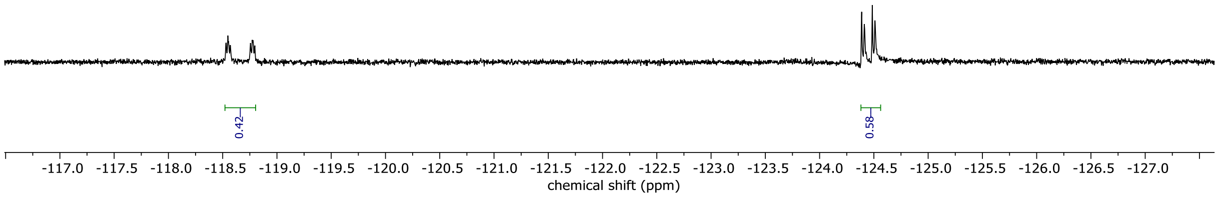

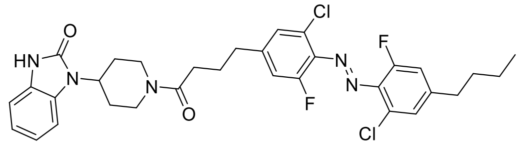

dfdc-OptoBI-1

^{19}F NMR (471 MHz, 500 μM, DMSO-d_6/D_2O 9:1)

dark-adapted → 650 nm irradiation for 30 min

trans:*cis* = 34:66

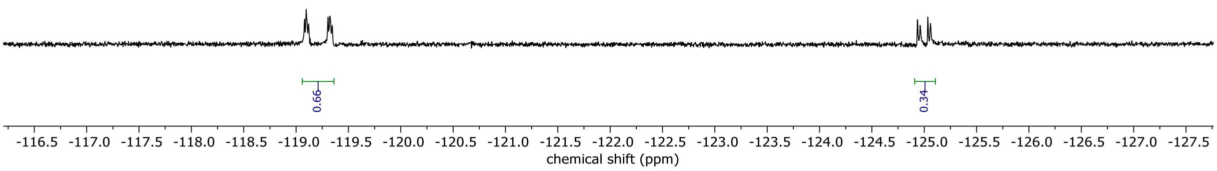

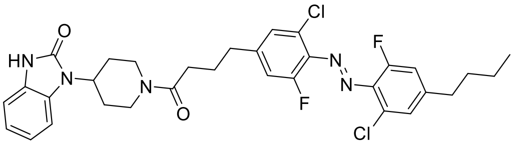

dfdc-OptoBI-1

^{19}F NMR (471 MHz, 500 μM, DMSO-d_6/D_2O 9:1)

dark-adapted → 650 nm irradiation for 45 min

trans:*cis* = 20:80

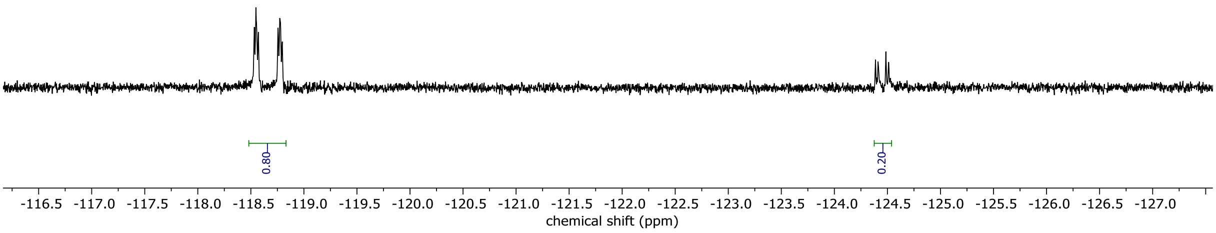

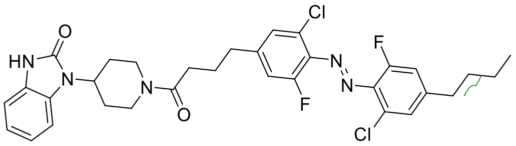

dfdc-OptoBI-1

^{19}F NMR (471 MHz, 500 μM, DMSO-d_6/D_2O 9:1)

dark-adapted → 650 nm irradiation for 60 min

trans:*cis* = 11:89

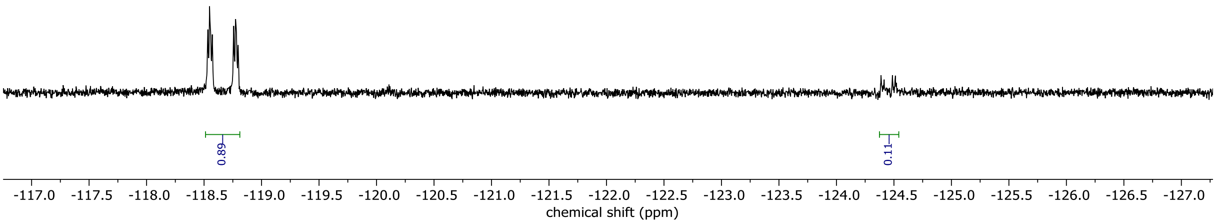

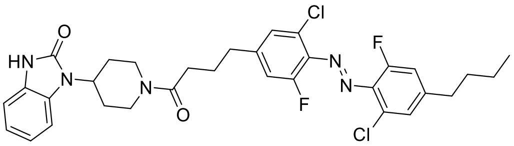

dfdc-OptoBI-1

^{19}F NMR (471 MHz, 500 μM, DMSO-d_6/D_2O 9:1)

650 nm adapted → 525 nm irradiation for 15 min

trans:*cis* = 19:81

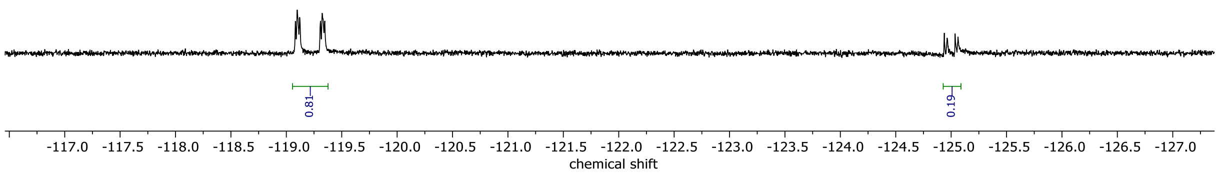

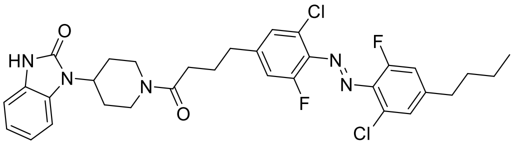

dfdc-OptoBI-1

^{19}F NMR (471 MHz, 500 μM, DMSO-d_6/D_2O 9:1)

525 nm adapted → 450 nm irradiation for 15 min

trans:*cis* = 78:22

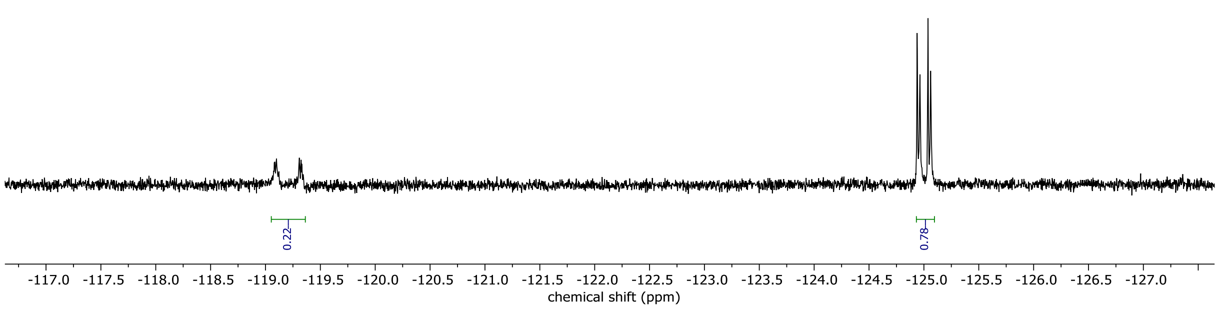

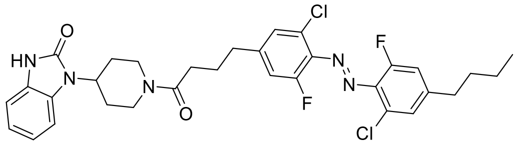

dfdc-OptoBI-1

^{19}F NMR (471 MHz, 500 μM, DMSO-d_6/D_2O 9:1)

450 nm adapted → 365 nm irradiation for 15 min

trans:*cis* = 29:71

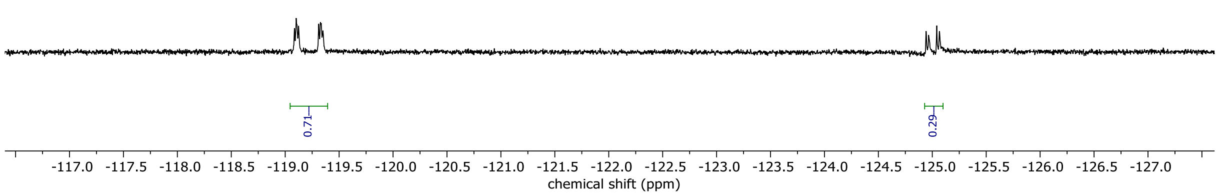

2. UV-vis Data – Photostationary State (PSS) Analysis

To determine the photostationary states (PSS), the dark adapted azobenzenes were first irradiated with 650 nm, 525 nm, 450-455 nm and 365 nm and then measured at a Cary 60 UV-vis spectrophotometer until the final PSS is reached.

(*E*/*Z*)-Azobenzene (1, 50 µM, DMSO/H_2O 9:1)

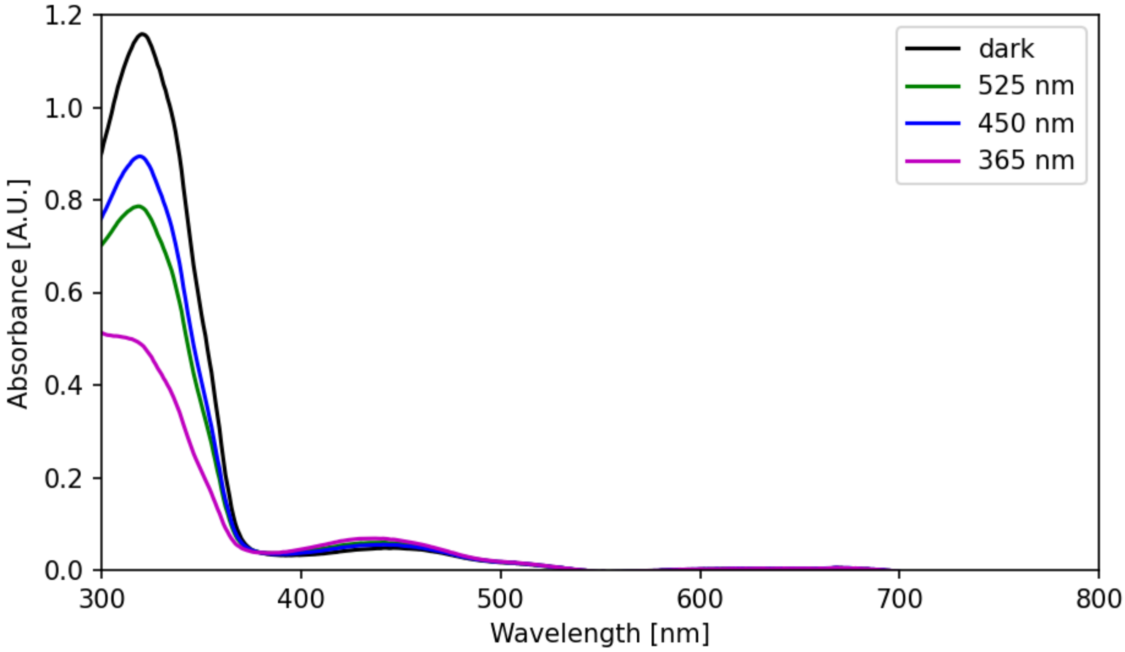

(*E*/*Z*)-Azobenzene (1, 500 µM, DMSO-d_6/D_2O 9:1)

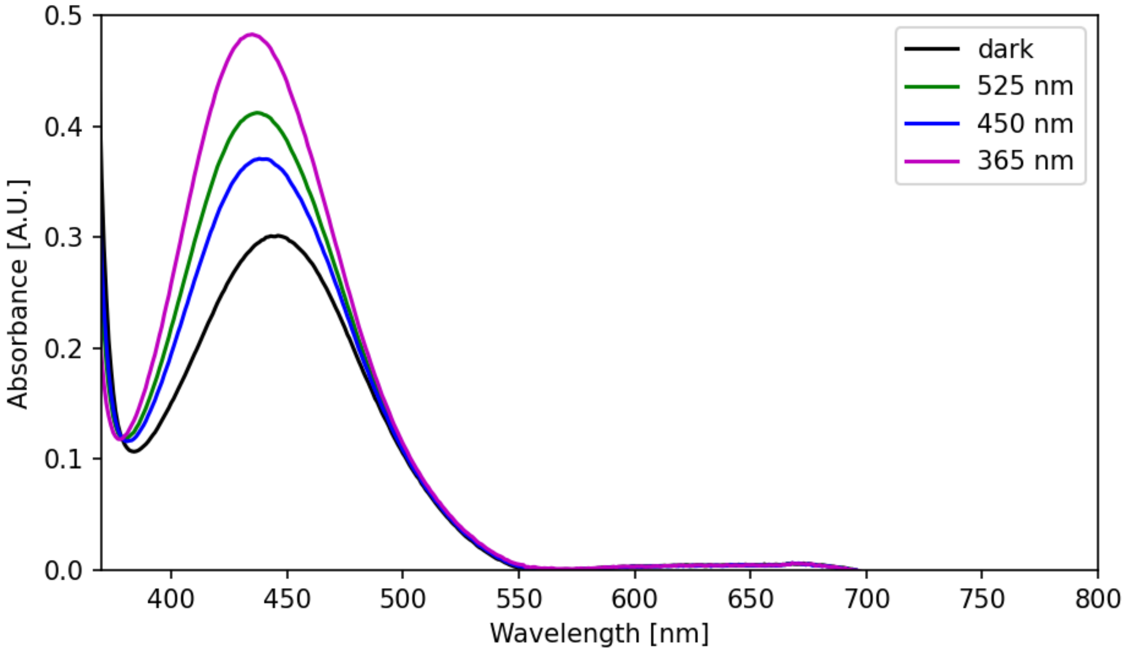

(*E*/*Z*)-1,2-Bis(2,6-difluorophenyl)diazene (2, 50 µM, DMSO/H_2O 9:1)

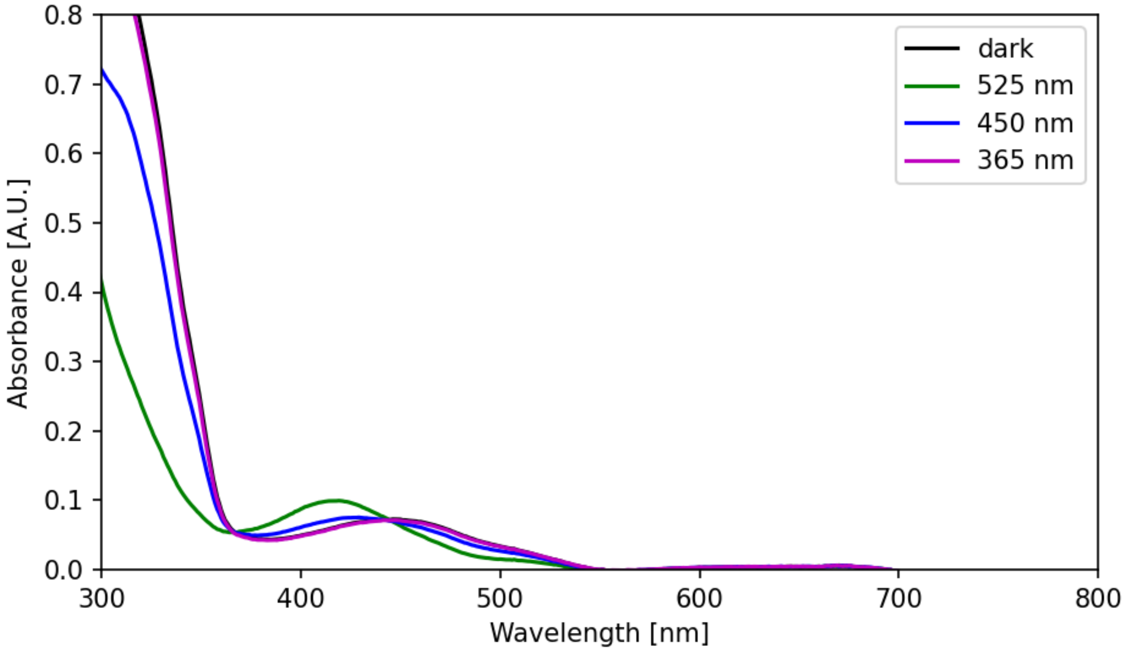

(*E*/*Z*)-1,2-Bis(2,6-difluorophenyl)diazene (2, 500 µM, DMSO-d_6/D_2O 9:1)

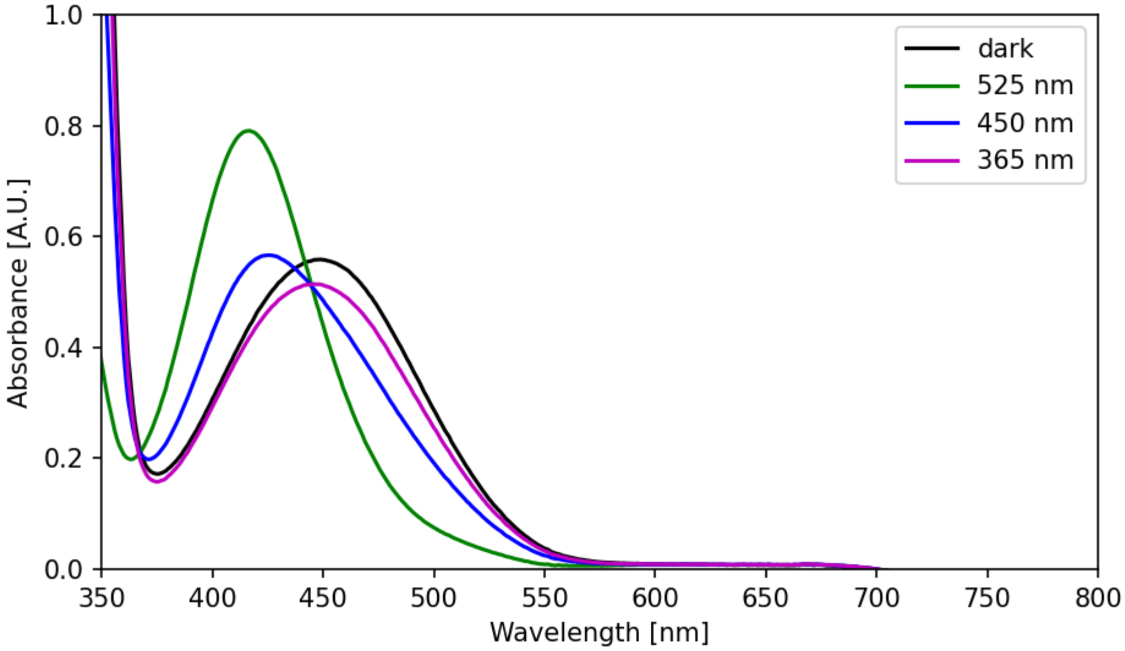

(*E*/*Z*)-1,2-Bis(2-chloro-6-fluorophenyl)diazene (3, 50 μM, DMSO/H_2O 9:1)

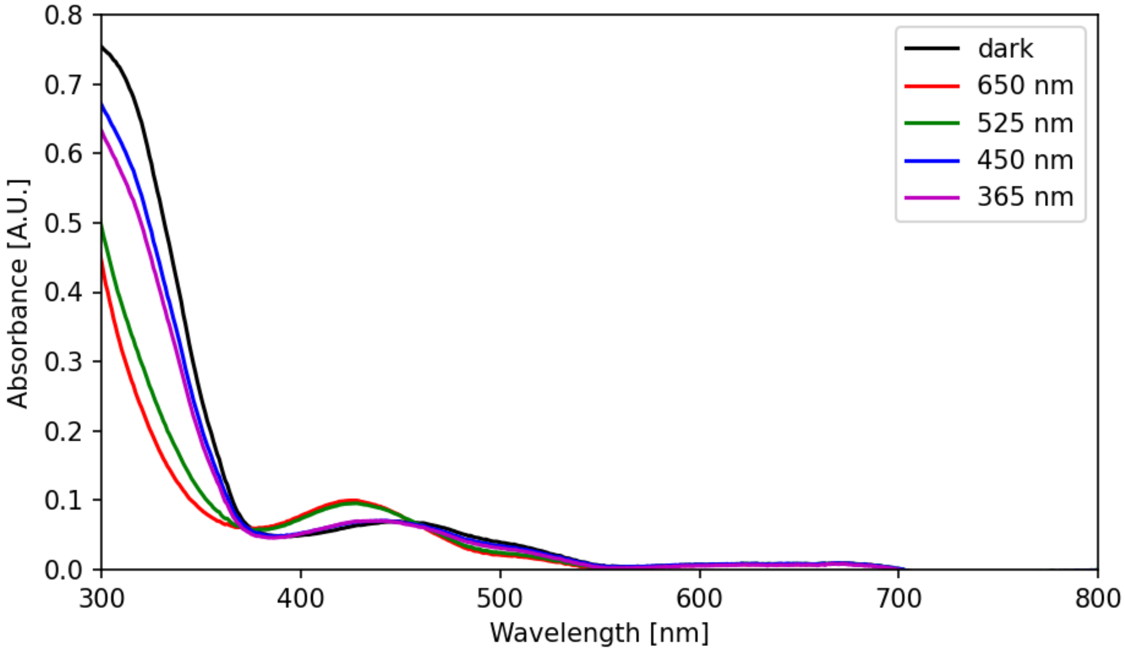

(*E*/*Z*)-1,2-Bis(2-chloro-6-fluorophenyl)diazene (3, 500 μM, DMSO-*d*$_6$/D_2O 9:1)

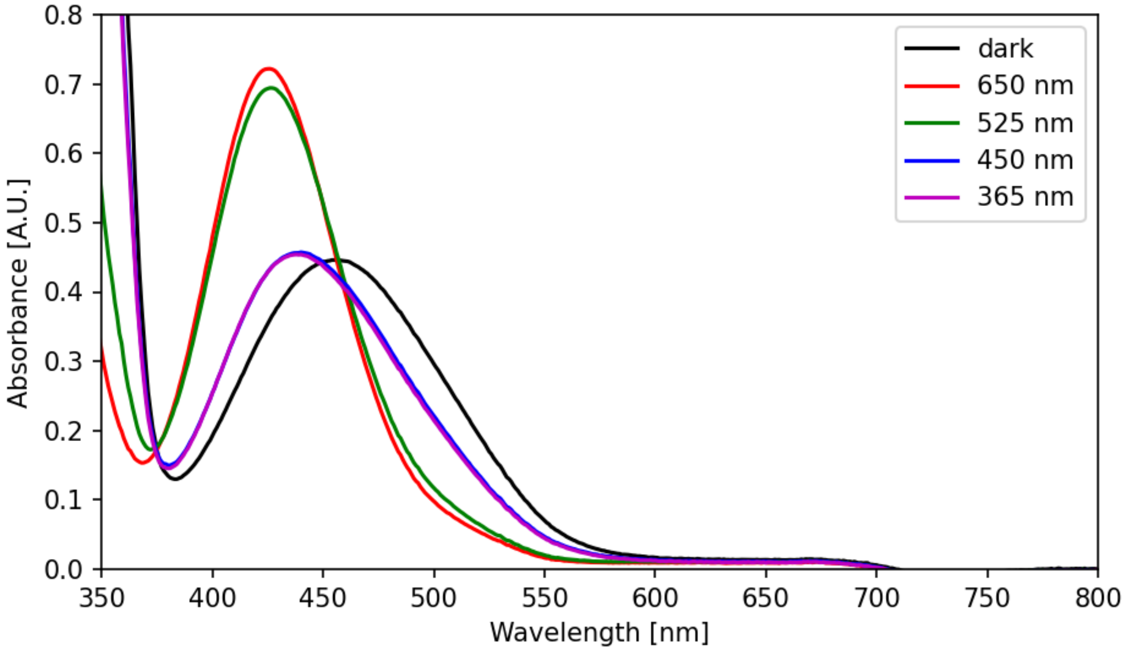

(*E*/*Z*)-1,2-Bis(2-bromo-6-fluorophenyl)diazene (4, 50 µM, DMSO/H_2O 9:1)

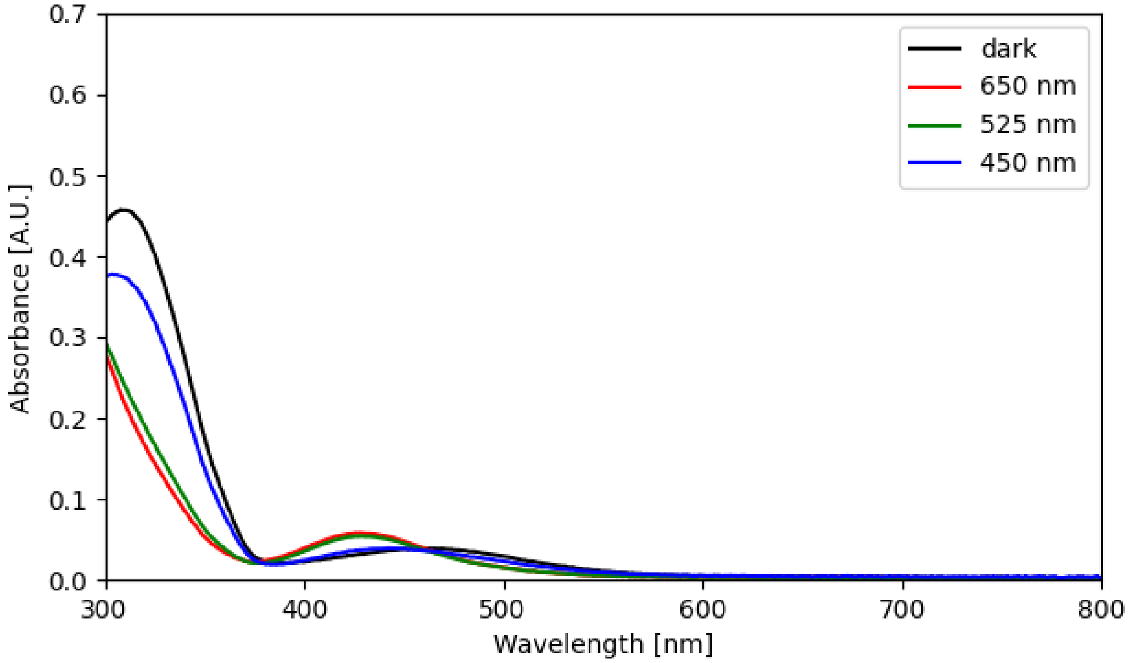

(*E*/*Z*)-1,2-Bis(2-bromo-6-fluorophenyl)diazene (4, 500 µM, DMSO-d_6/D_2O 9:1)

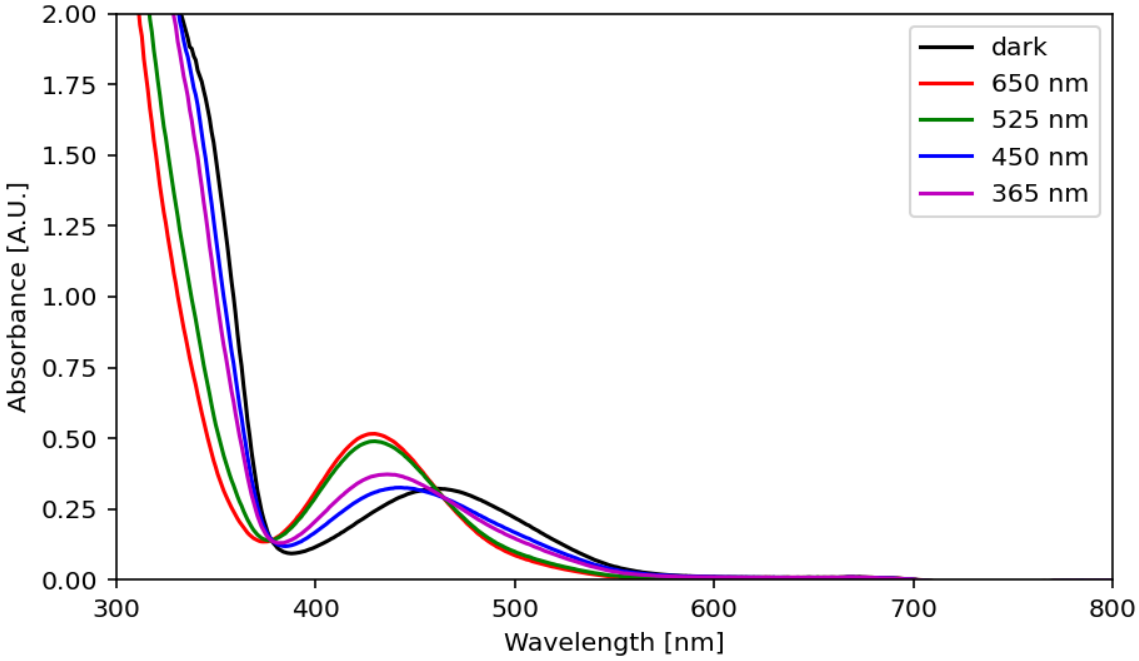

(*E*/*Z*)-1,2-Bis(2,6-dichlorophenyl)diazene (6, 50 μM, DMSO/H_2O 9:1)

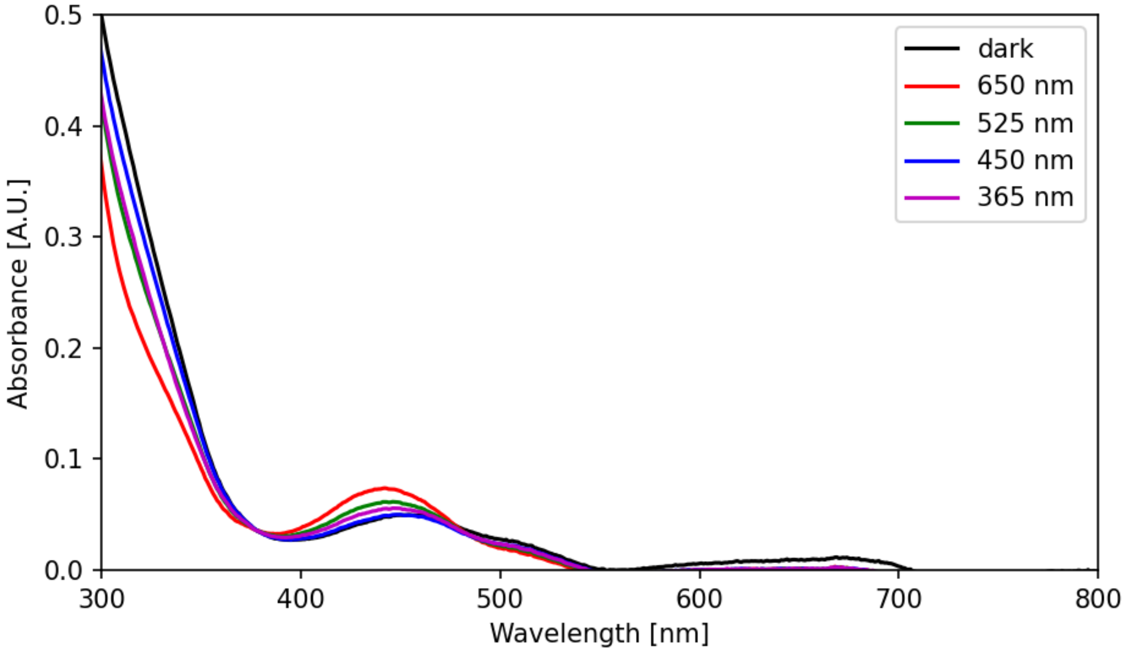

(*E*/*Z*)-1,2-Bis(2,6-dichlorophenyl)diazene (6, 500 μM, DMSO-*d_6*/D_2O 9:1)

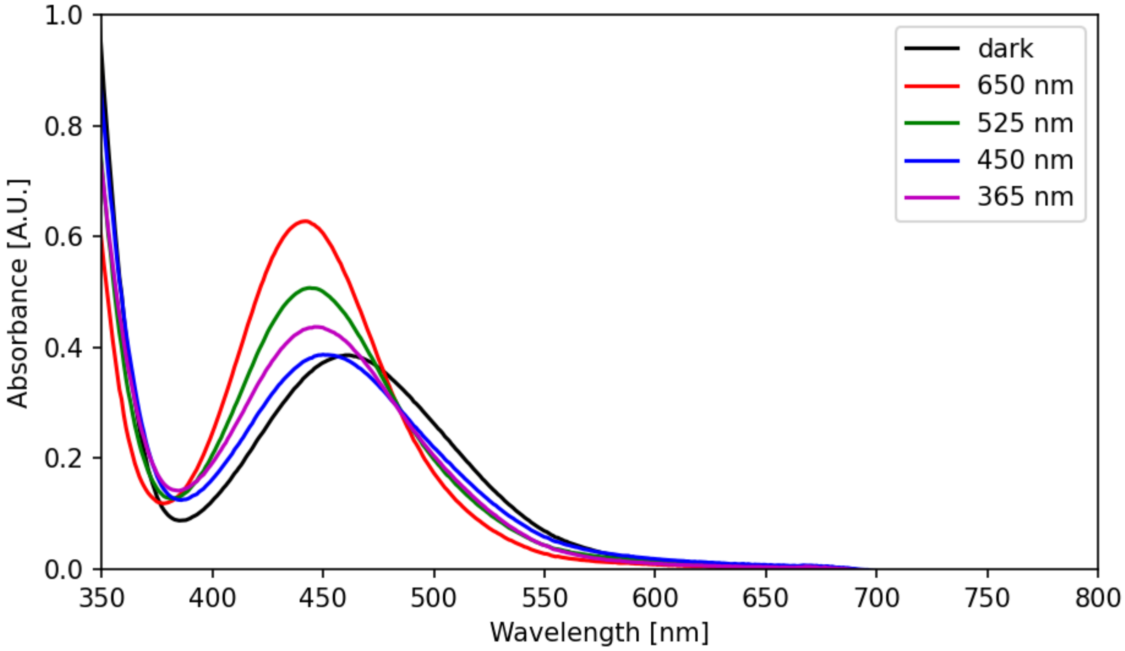

(*E*/*Z*)-1,2-Bis(2,6-dimethoxyphenyl)diazene (9, 50 μM, DMSO/H_2O 9:1)

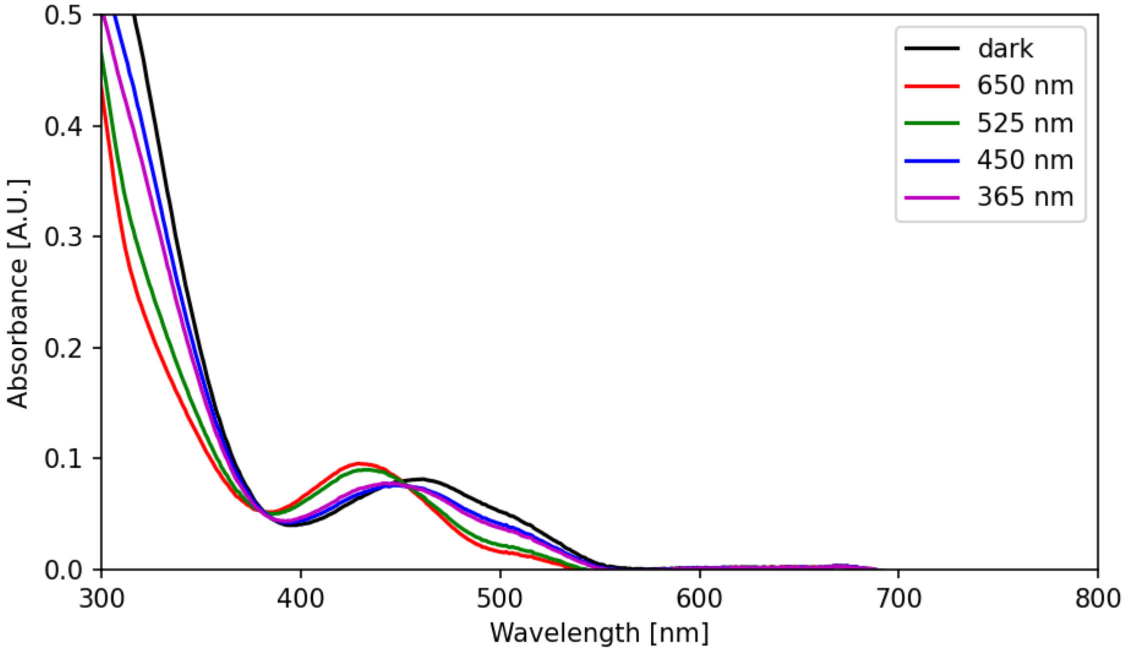

(*E*/*Z*)-1,2-Bis(2,6-dimethoxyphenyl)diazene (9, 500 μM, DMSO-d_6/D_2O 9:1)

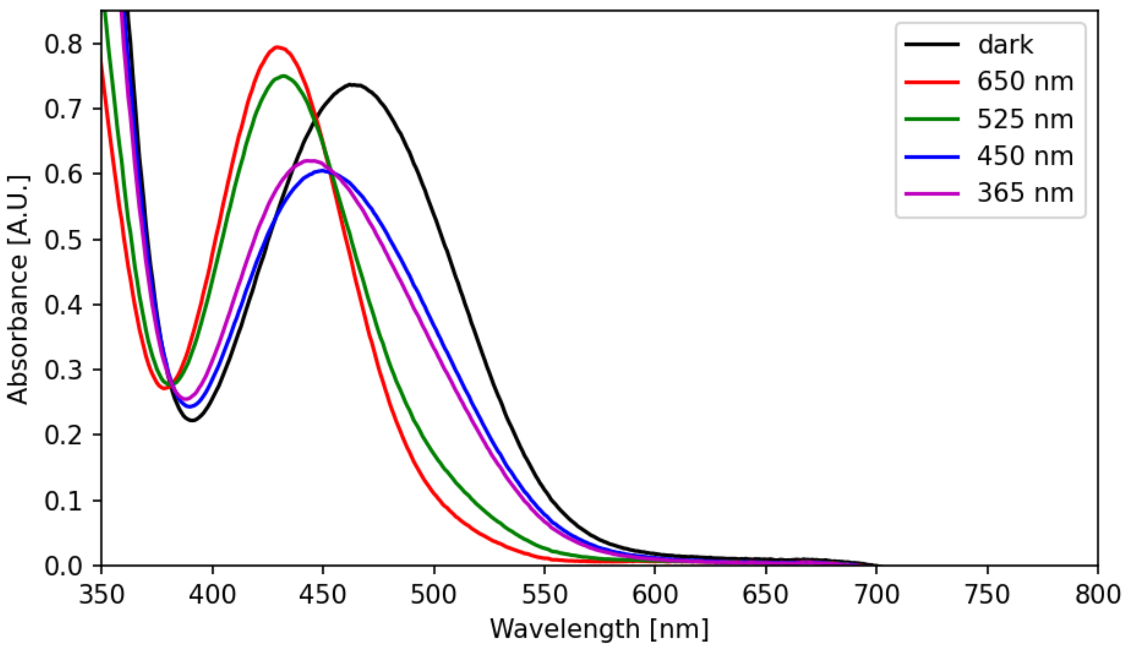

(*E*/*Z*)-1,2-Bis(3-chloro-1-fluoronaphthalen-2-yl)diazene (12, 50 µM, DMSO-d_6/D_2O 9:1)

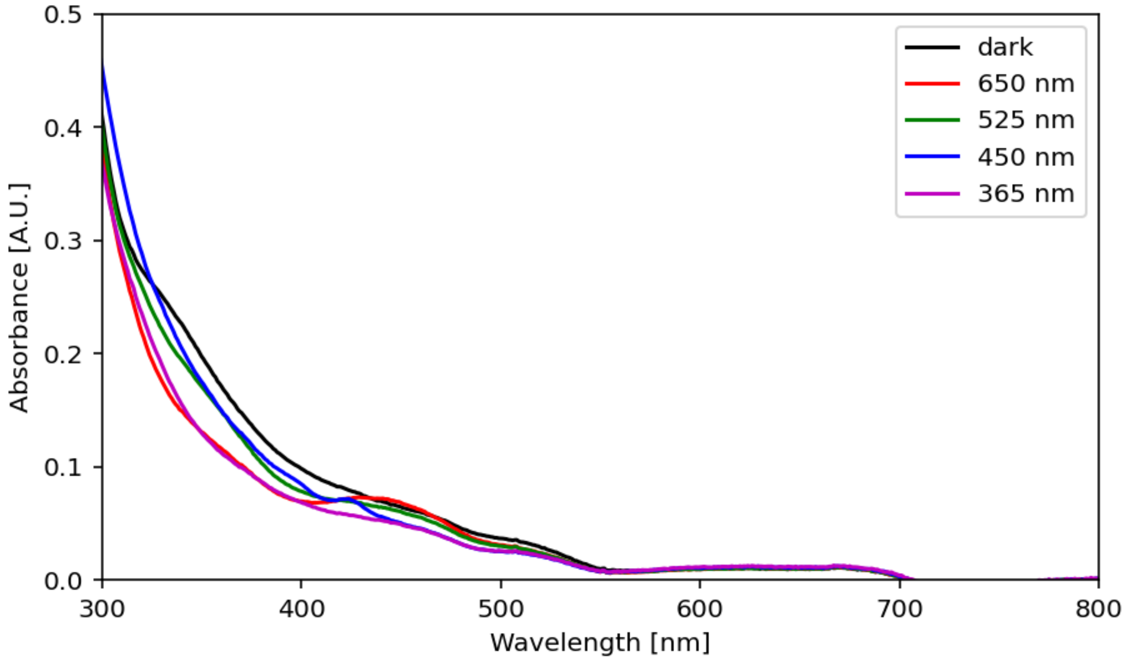

(*E*/*Z*)-1,2-Bis(3-chloro-1-fluoronaphthalen-2-yl)diazene (12, 500 µM, DMSO-d_6/D_2O 9:1)

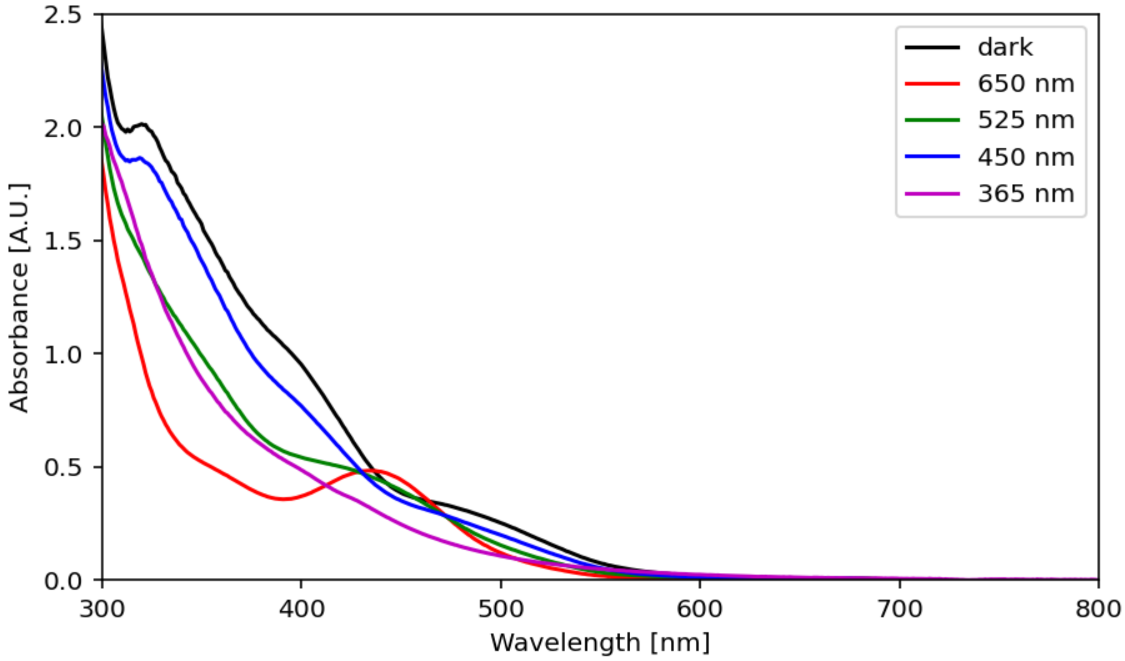

(*E*/*Z*)-3-Chloro-4-((2-chloro-6-fluorophenyl)diazenyl)-5-fluorobenzoic acid (17, 50 µM, DMSO/H_2O 9:1)

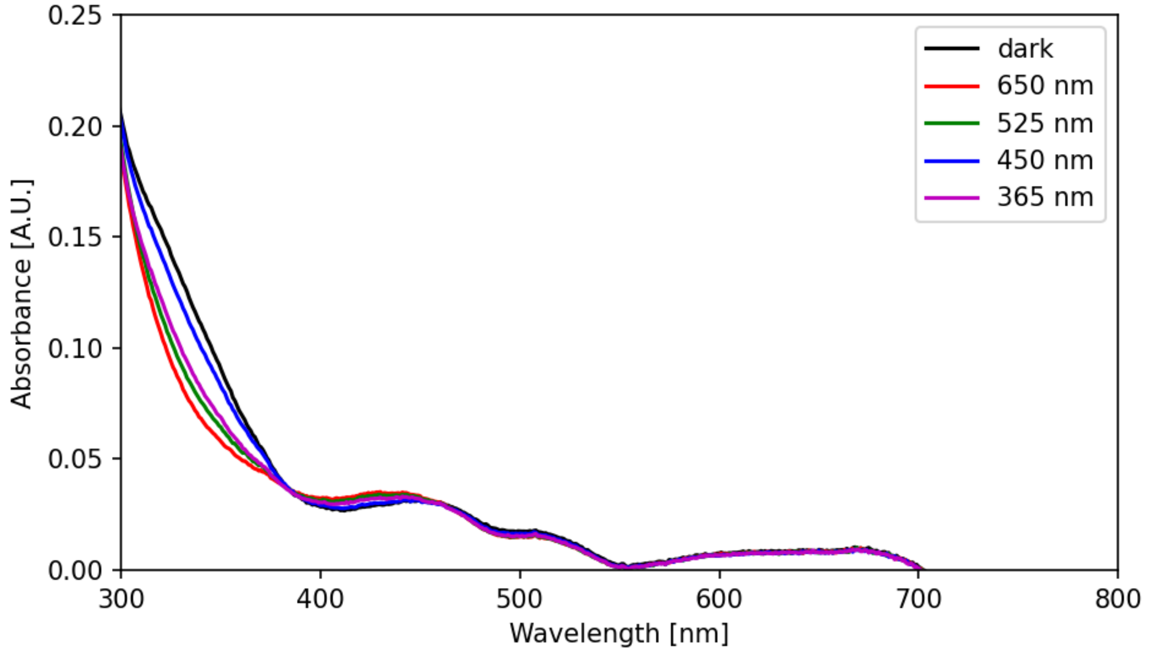

(*E*/*Z*)-3-Chloro-4-((2-chloro-6-fluorophenyl)diazenyl)-5-fluorobenzoic acid (17, 500 µM, DMSO-d_6/D_2O 9:1)

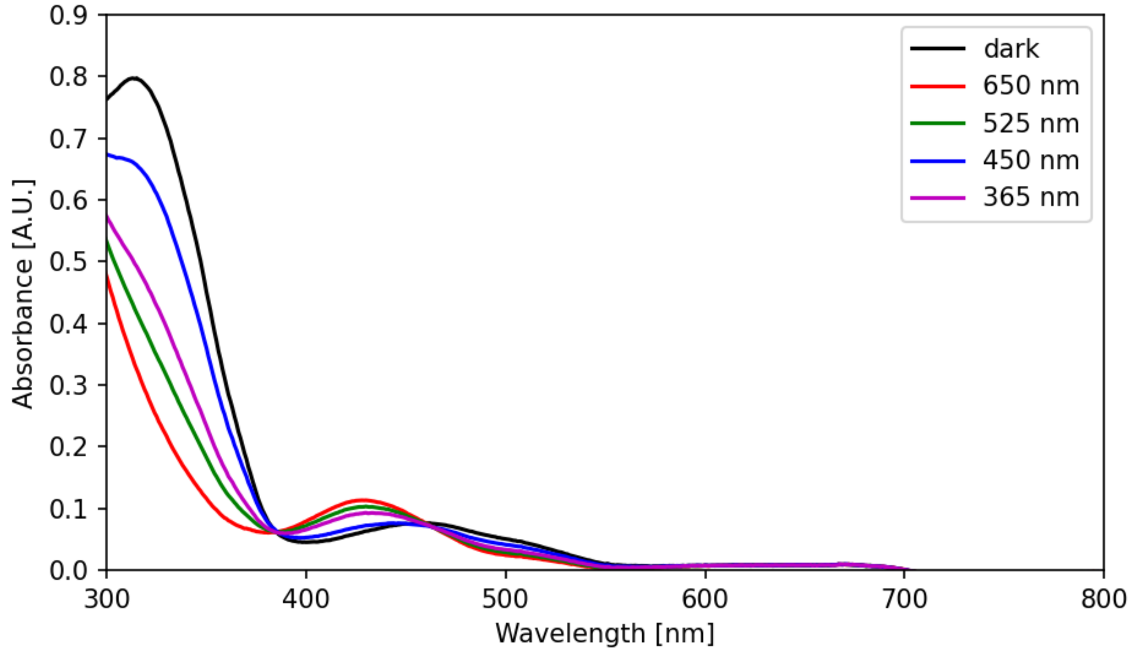

(*E*/*Z*)-1-(2-Chloro-6-fluoro-4-nitrophenyl)-2-(2-chloro-6-fluorophenyl)diazene (19, 50 µM, DMSO/H_2O 9:1)

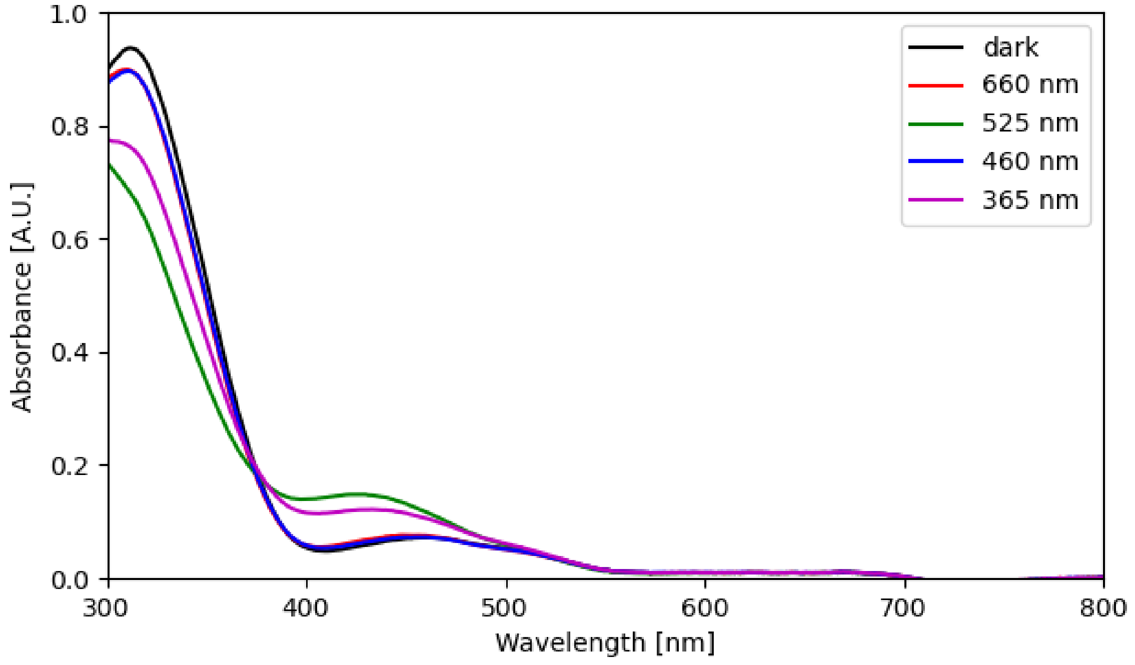

(*E*/*Z*)-1-(2-Chloro-6-fluoro-4-nitrophenyl)-2-(2-chloro-6-fluorophenyl)diazene (19, 500 µM, DMSO-*d*$_6$/D_2O 9:1)

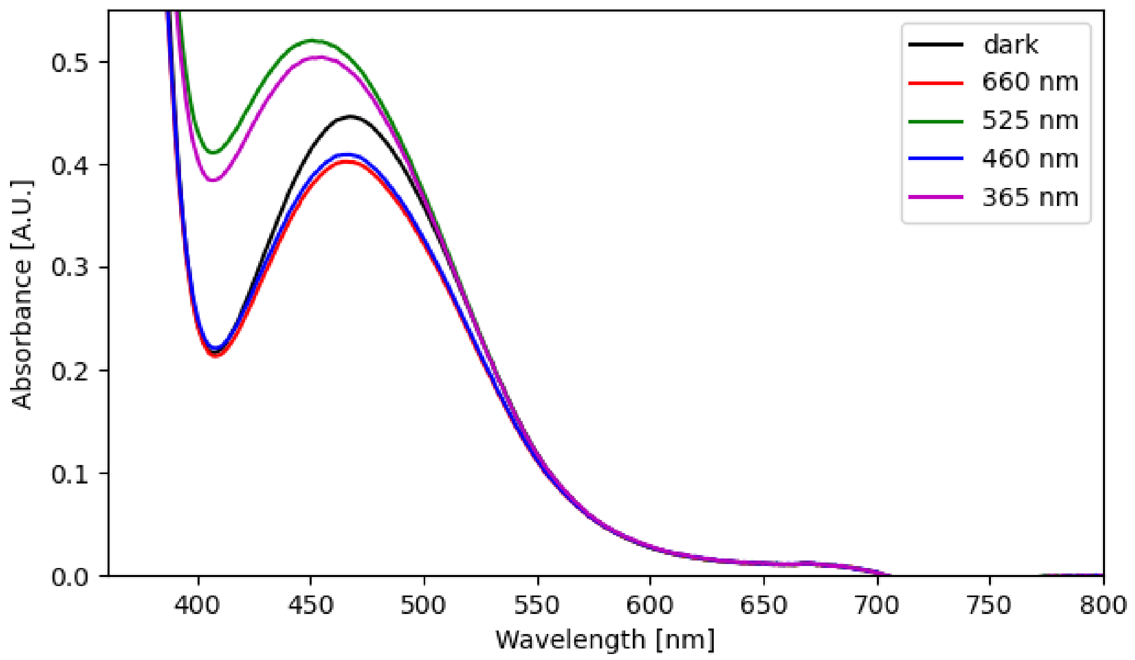

(*E*/*Z*)-3-Chloro-4-((2-chloro-6-fluoro-4-nitrophenyl)diazenyl)-5-fluorobenzoic acid (20, 50 µM, DMSO/H_2O 9:1)

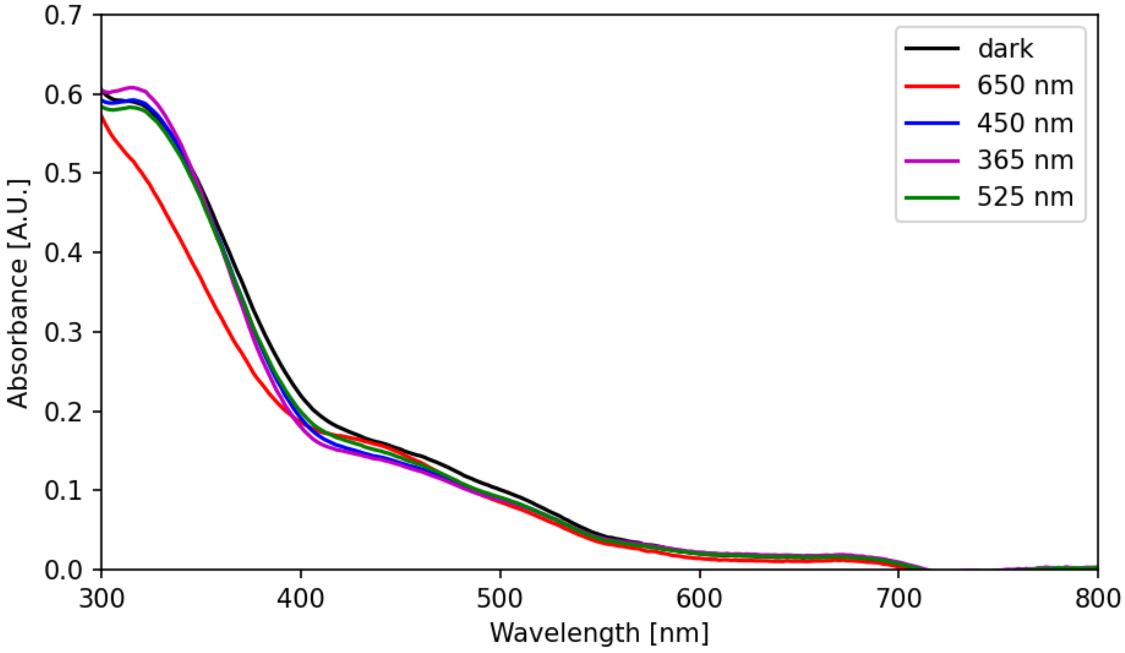

(*E*/*Z*)-3-Chloro-4-((2-chloro-6-fluoro-4-nitrophenyl)diazenyl)-5-fluorobenzoic acid (20, 500 µM, DMSO-d_6/D_2O 9:1)

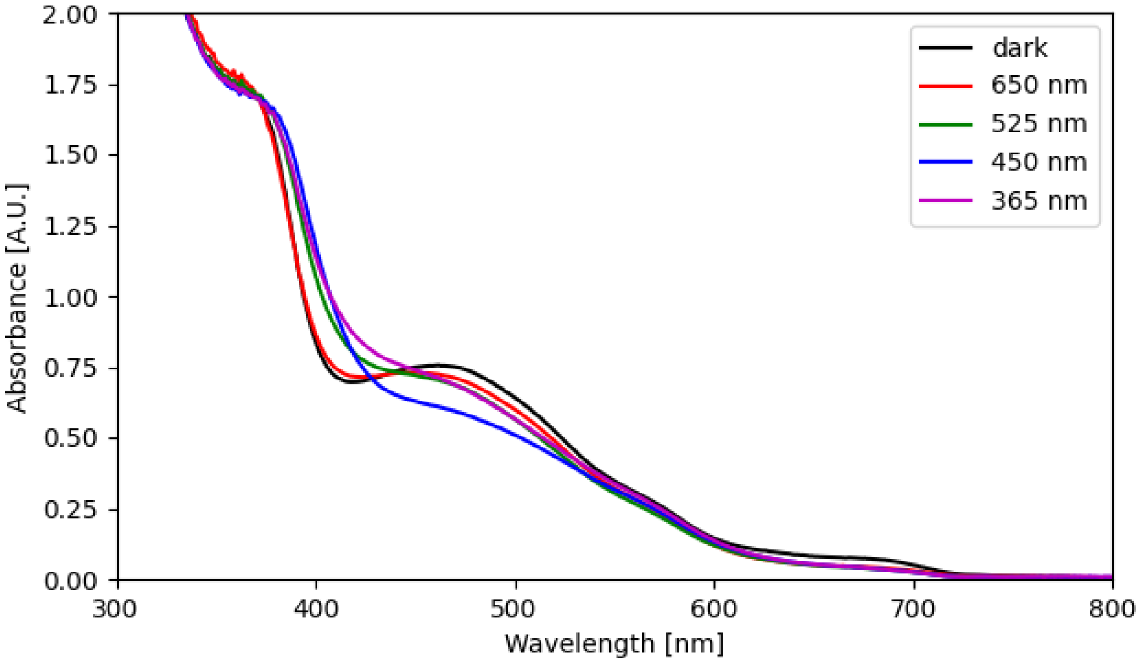

(*E*/*Z*)-Methyl-3-chloro-4-((2-chloro-6-fluorophenyl)diazenyl)-5-fluorobenzoate (21, 50 µM, DMSO/H_2O 9:1)

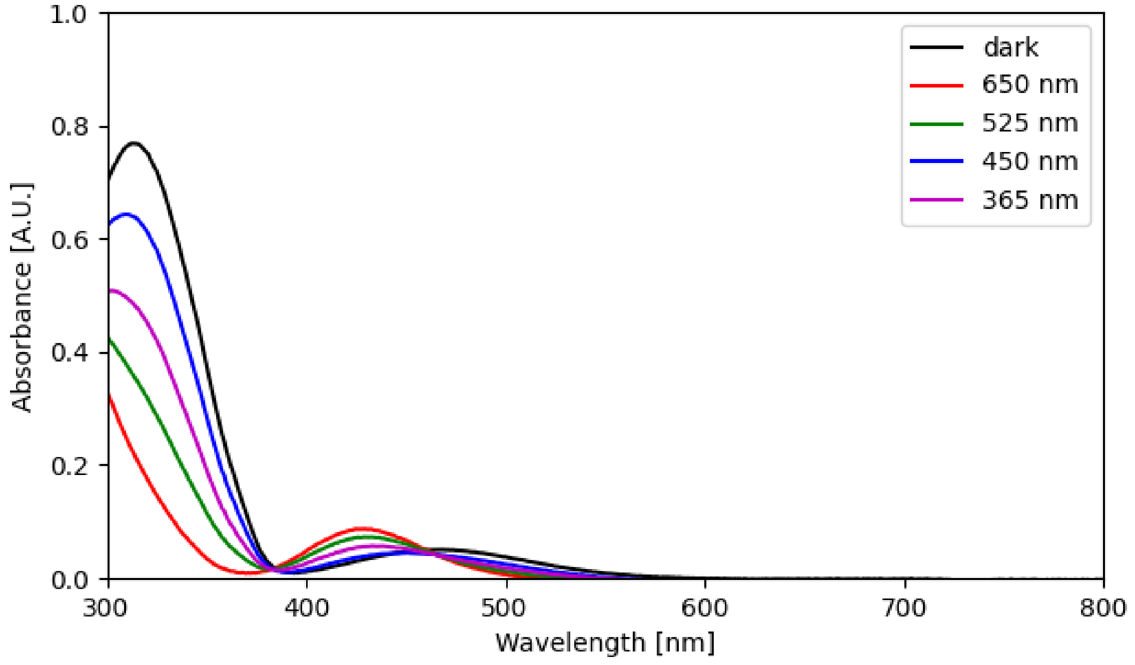

(*E*/*Z*)-Methyl-3-chloro-4-((2-chloro-6-fluorophenyl)diazenyl)-5-fluorobenzoate (21, 500 µM, DMSO-*d*$_6$/D_2O 9:1)

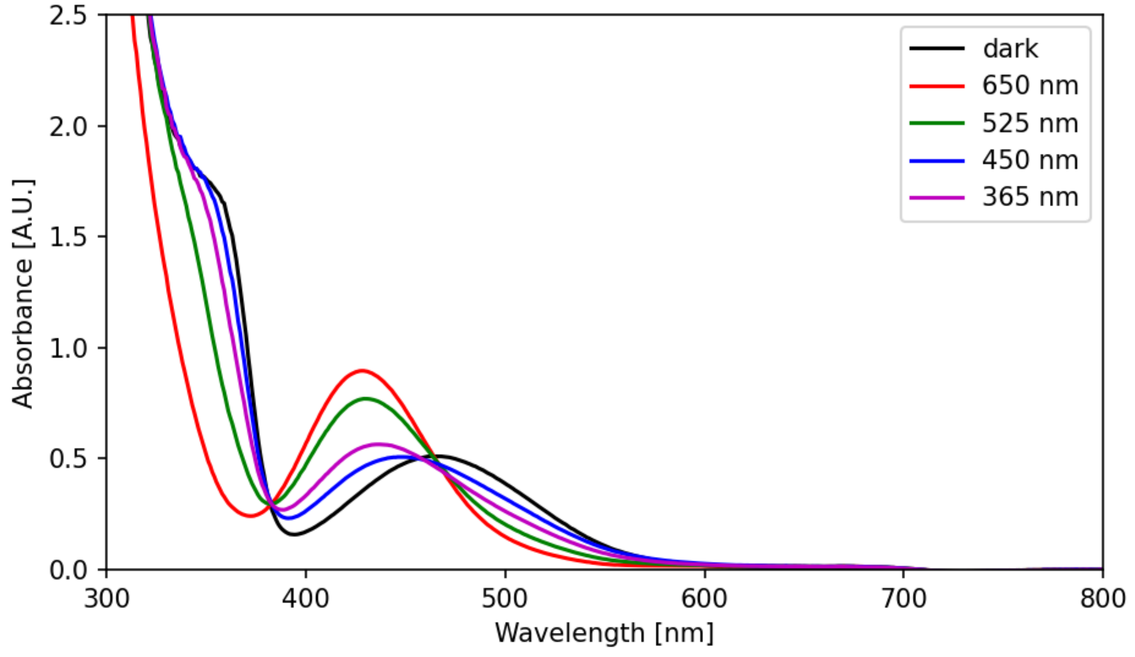

(*E*/*Z*)-Methyl-3-bromo-4-((2-bromo-6-fluorophenyl)diazenyl)-5-fluorobenzoate (22, 50 µM, DMSO/H_2O 9:1)

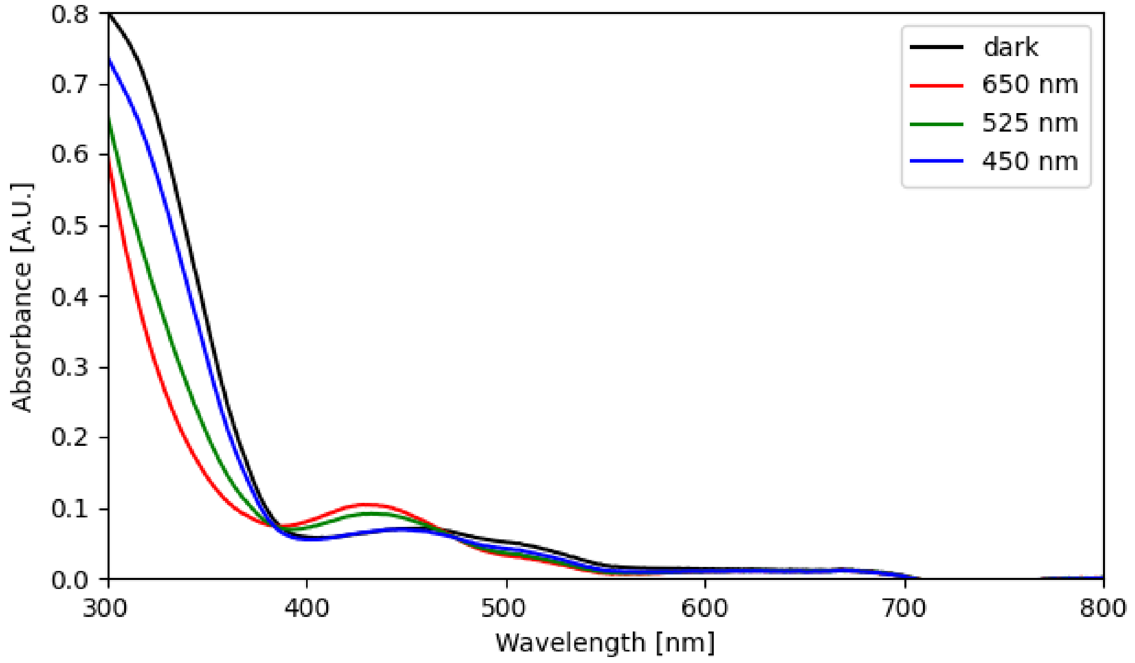

(*E*/*Z*)-Methyl-3-bromo-4-((2-bromo-6-fluorophenyl)diazenyl)-5-fluorobenzoate (22, 500 µM, DMSO-*d*$_6$/D_2O 9:1)

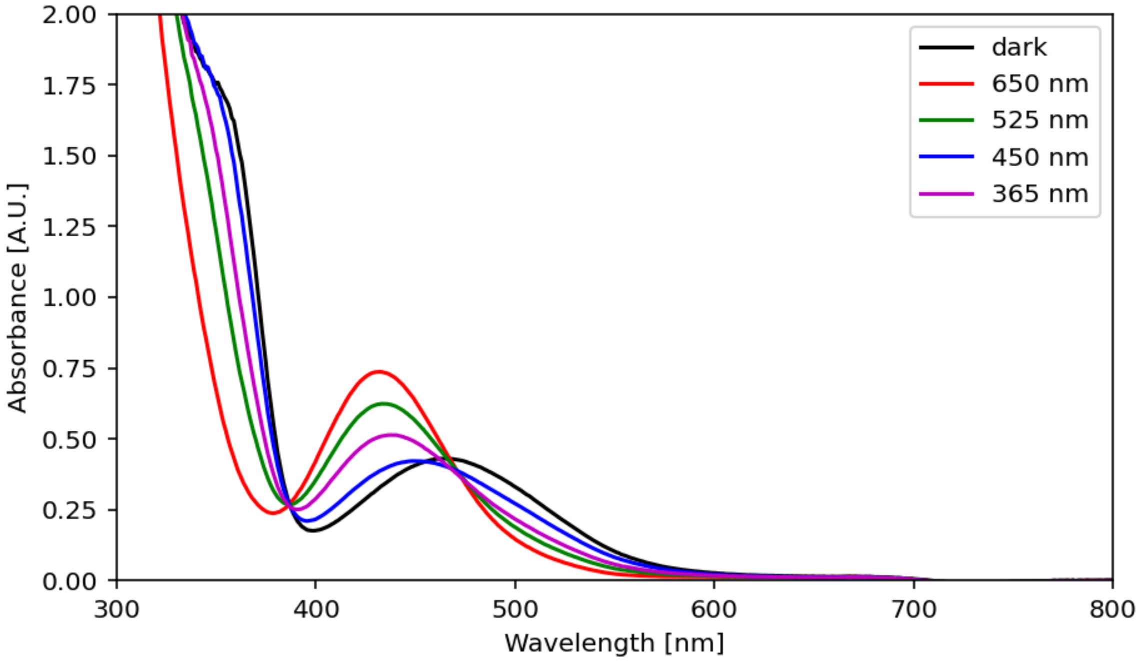

(*E*/*Z*)-Methyl-3,5-dichloro-4-((2,6-dichlorophenyl)diazenyl)benzoate (23, 50 µM, DMSO/H_2O 9:1)

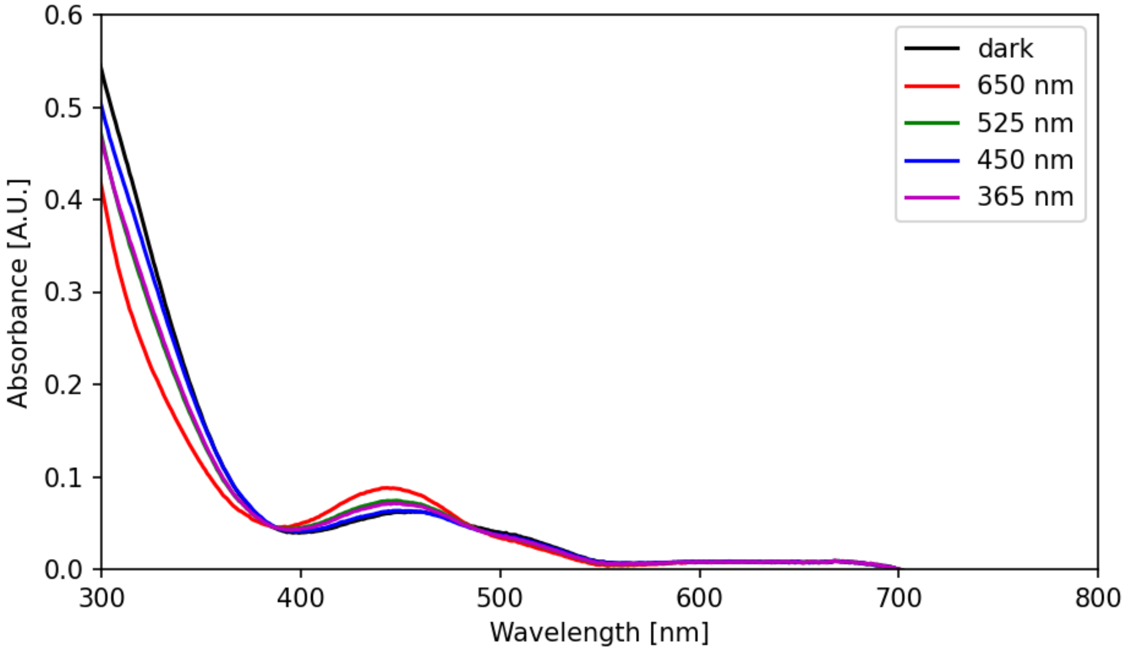

(*E*/*Z*)-Methyl-3,5-dichloro-4-((2,6-dichlorophenyl)diazenyl)benzoate (23, 500 µM, DMSO-d_6/D_2O 9:1)

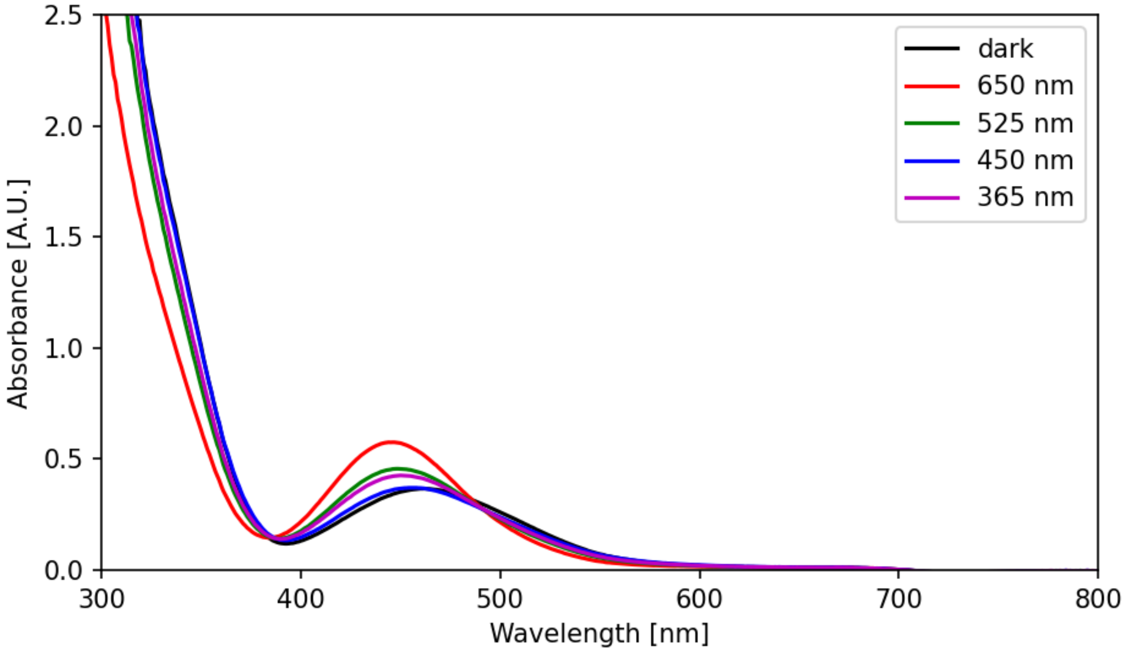

(*E*/*Z*)-3-Chloro-4-((2-chloro-6-fluorophenyl)diazenyl)-5-fluoro-N-methylbenzamide (24, 50 µM, DMSO/H_2O 9:1)

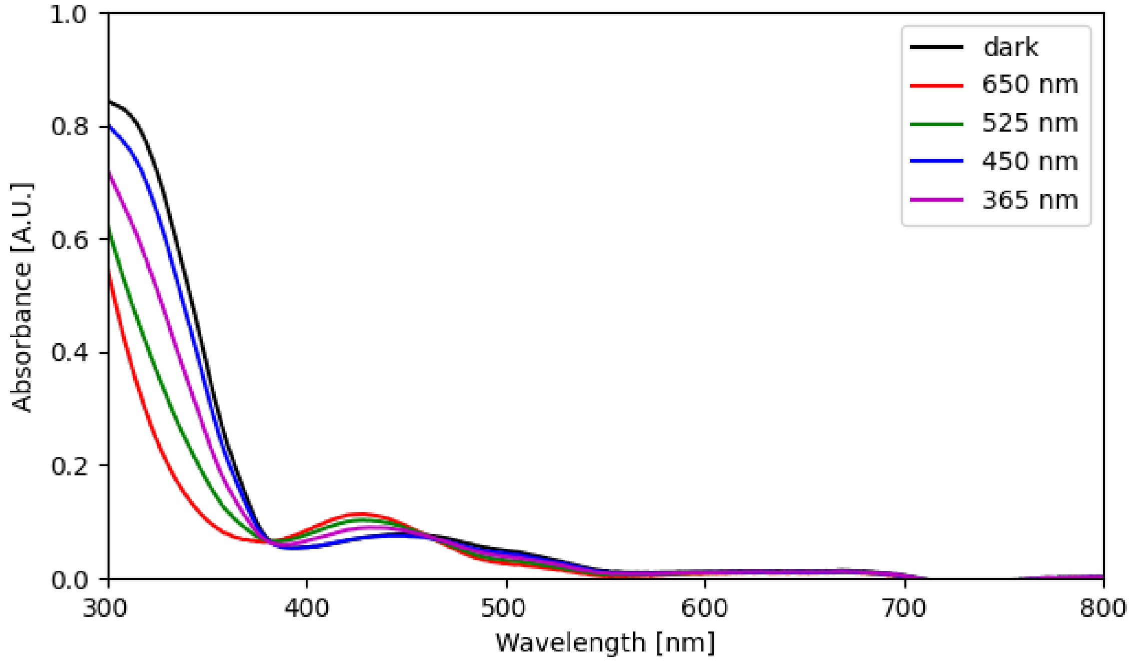

(*E*/*Z*)-3-Chloro-4-((2-chloro-6-fluorophenyl)diazenyl)-5-fluoro-N-methylbenzamide (24, 500 µM, DMSO-d_6/D_2O 9:1)

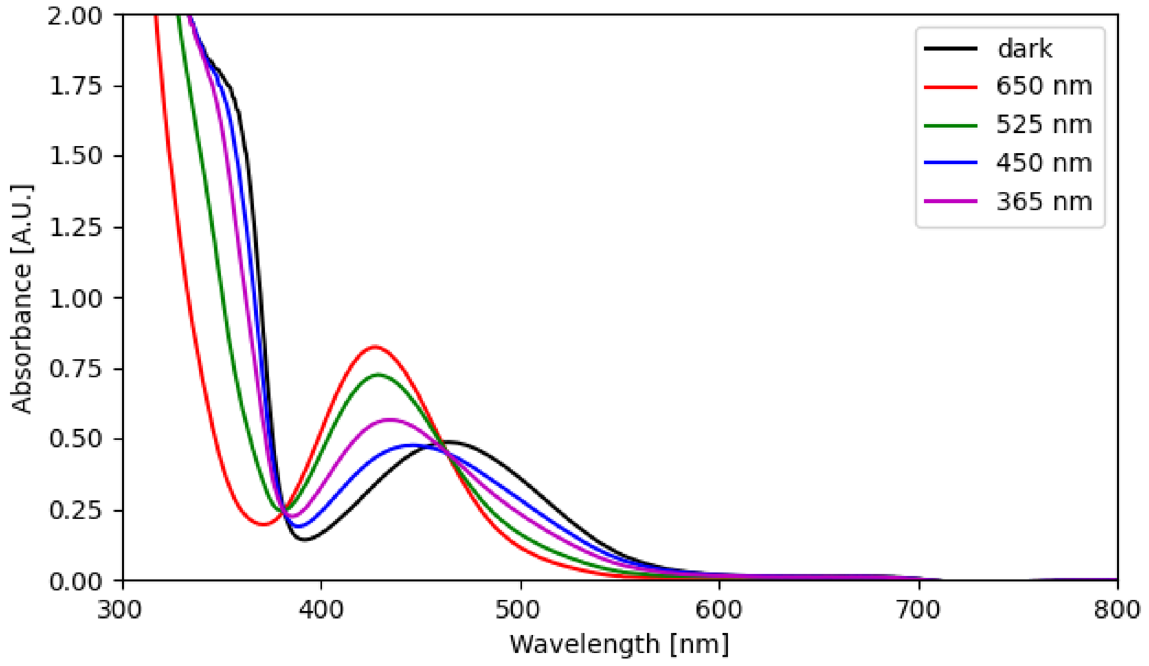

(E/Z)-Dimethyl 4,4'-(diazene-1,2-diyl)-bis(3-chloro-5-fluorobenzoate) (28, 50 μM, DMSO/H_2O 9:1)

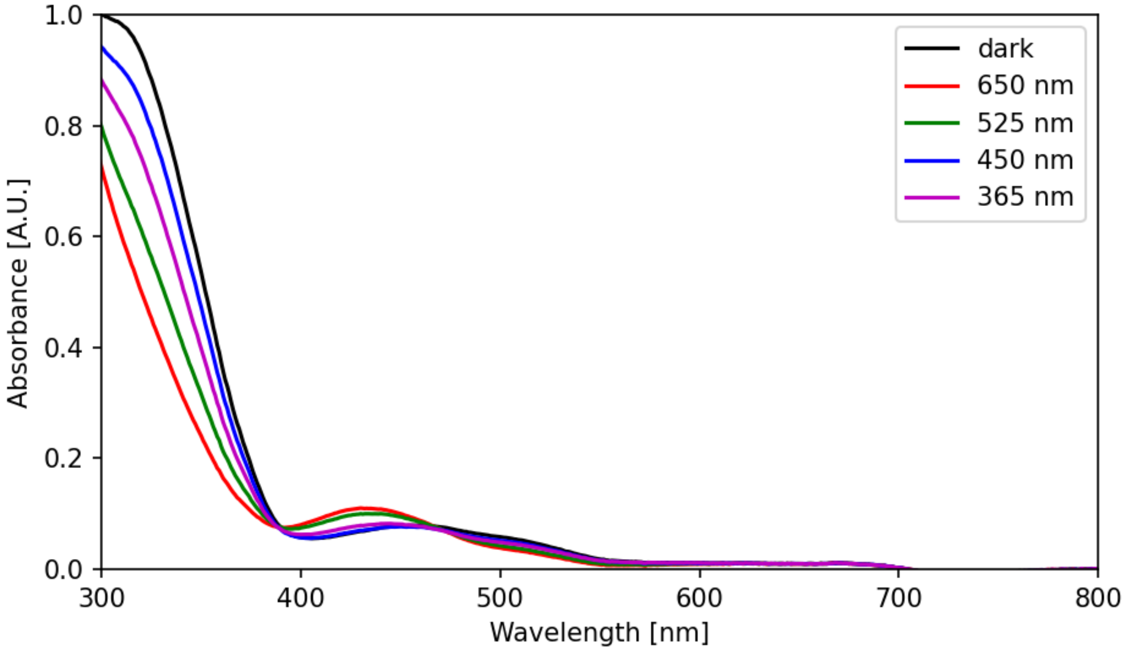

(E/Z)-Dimethyl 4,4'-(diazene-1,2-diyl)-bis(3-chloro-5-fluorobenzoate) (28, 500 μM, DMSO-d_6/D_2O 9:1)

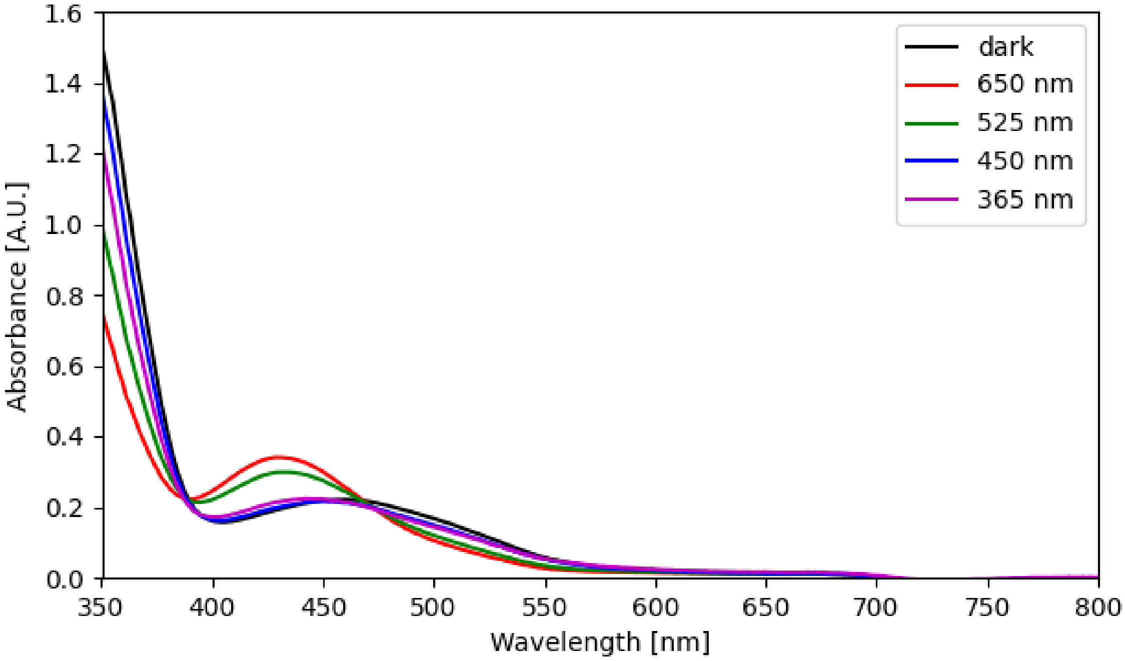

(*E*/*Z*)-3-Chloro-4-((2-chloro-6-fluorophenyl)diazenyl)-5-fluoroaniline (38, 50 µM, DMSO/H_2O 9:1)

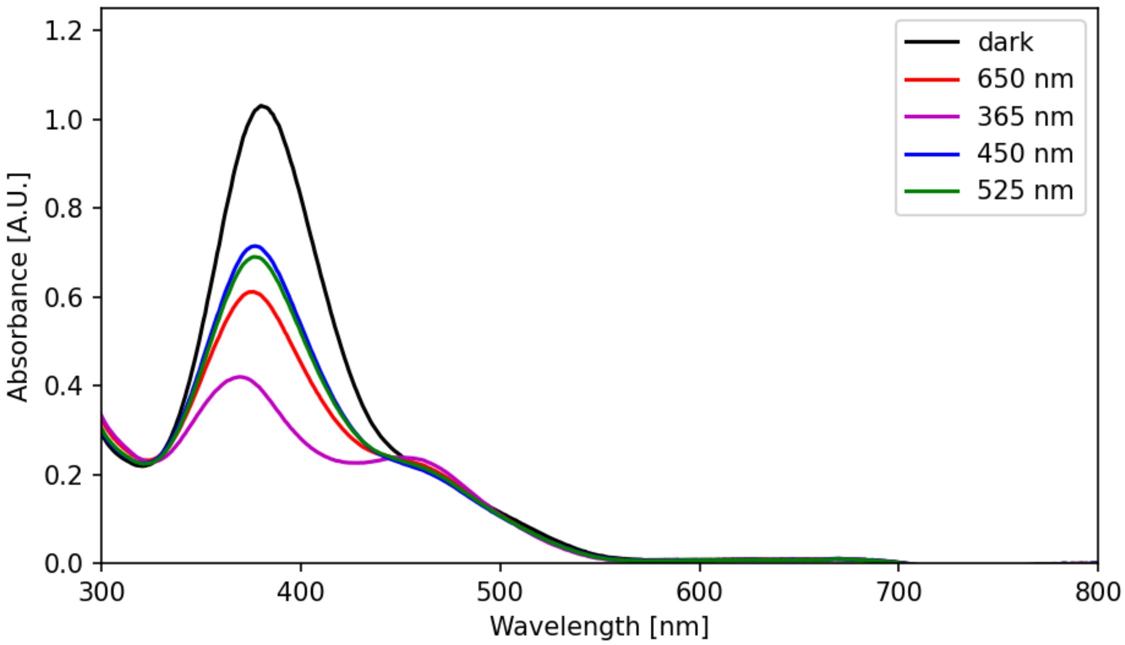

(*E*/*Z*)-3-Chloro-4-((2-chloro-6-fluorophenyl)diazenyl)-5-fluoroaniline (38, 500 µM, DMSO-d_6/D_2O 9:1)

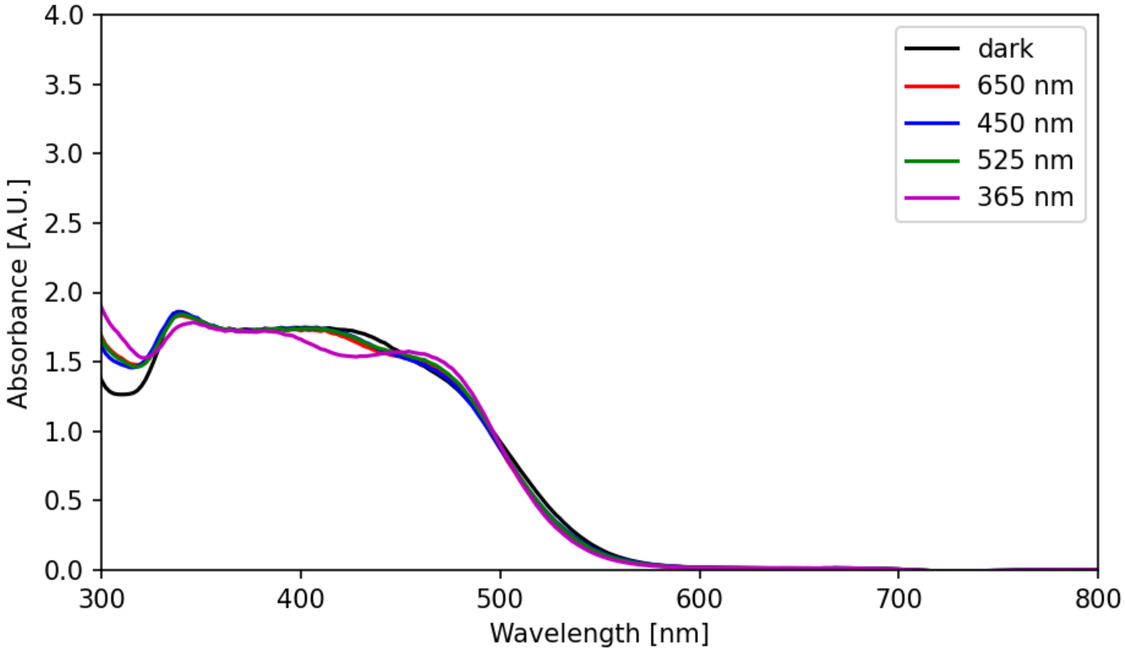

(*E*/*Z*)-*N*-(3-chloro-4-((2-chloro-6-fluorophenyl)diazenyl)-5-fluorophenyl)acetamid (39, 50 µM, DMSO/H_2O 9:1)

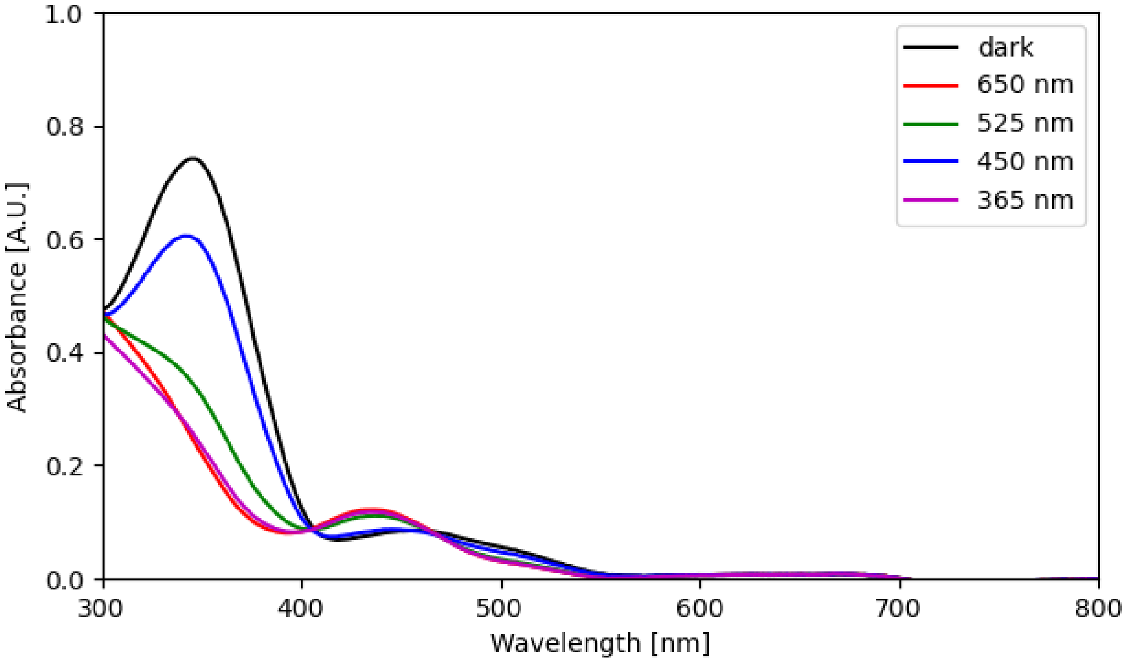

(*E*/*Z*)-*N*-(3-chloro-4-((2-chloro-6-fluorophenyl)diazenyl)-5-fluorophenyl)acetamid (39, 500 µM, DMSO-d_6/D_2O 9:1)

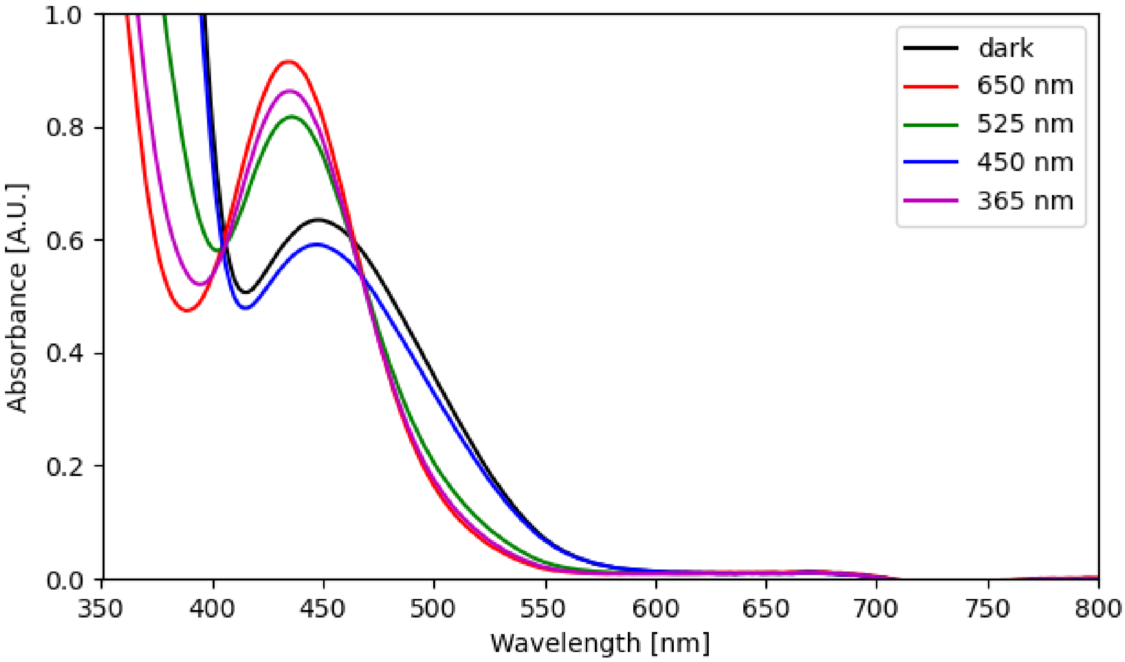

(*E*/*Z*)-Methyl-3-chloro-4-((2-chloro-6-fluoro-4-methoxyphenyl)diazenyl)-5-fluorobenzoate (46, 50 µM, DMSO/H_2O 9:1)

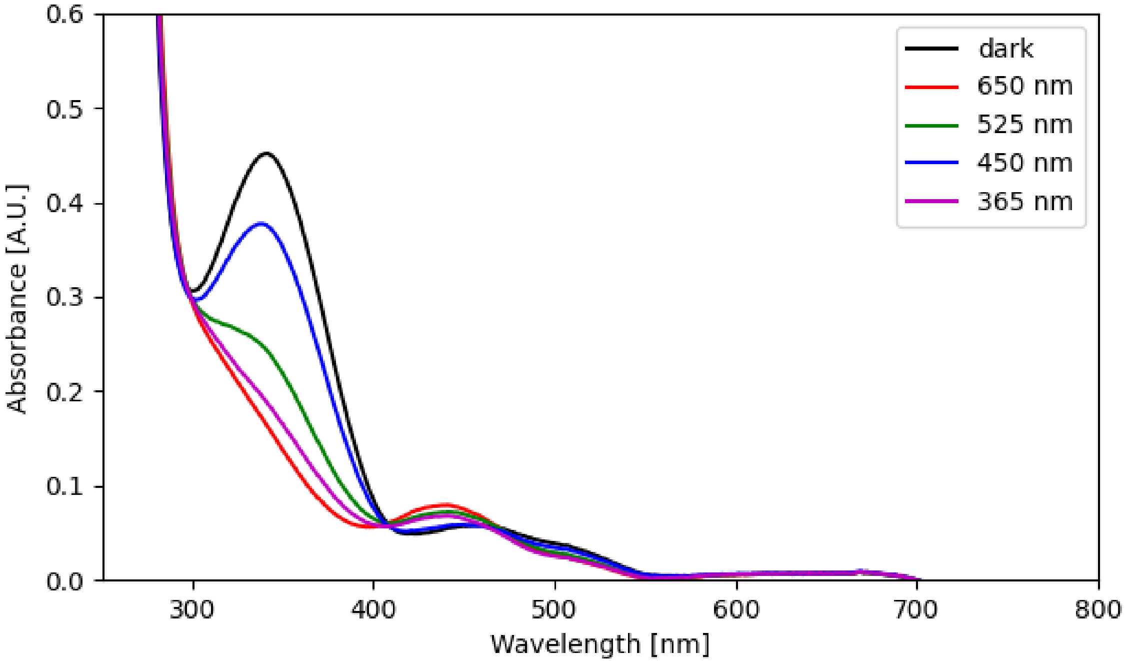

(*E*/*Z*)-Methyl-3-chloro-4-((2-chloro-6-fluoro-4-methoxyphenyl)diazenyl)-5-fluorobenzoate (46, 500 µM, DMSO-d_6/D_2O 9:1)

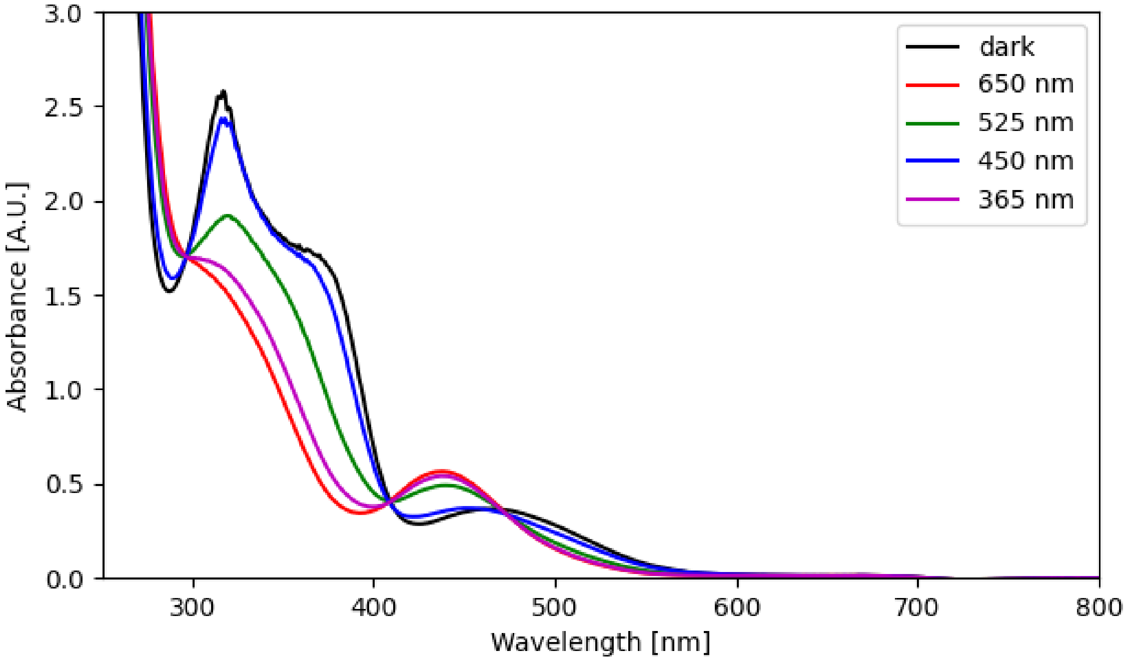

(*E*/*Z*)-4-((4-Amino-2,6-dichlorophenyl)diazenyl)-3,5-dichlorobenzenesulfonamide (48, 50 µM, DMSO/H_2O 9:1)

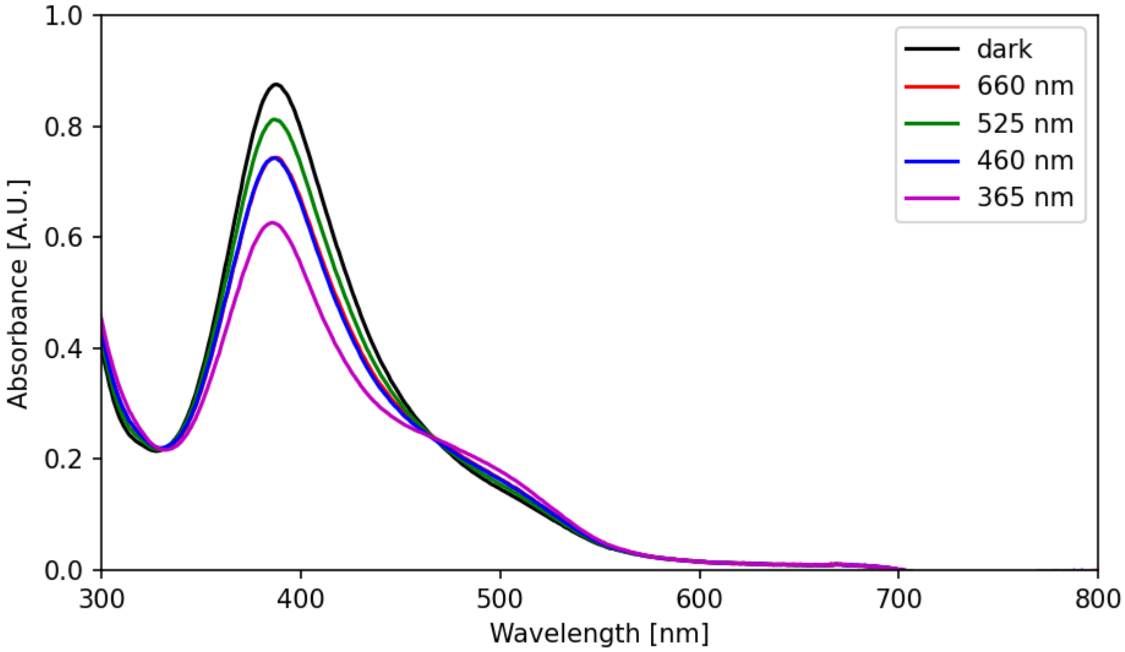

(*E*/*Z*)-4-((4-Amino-2,6-dichlorophenyl)diazenyl)-3,5-dichlorobenzenesulfonamide (48, 500 µM, DMSO-*d*$_6$/D_2O 9:1)

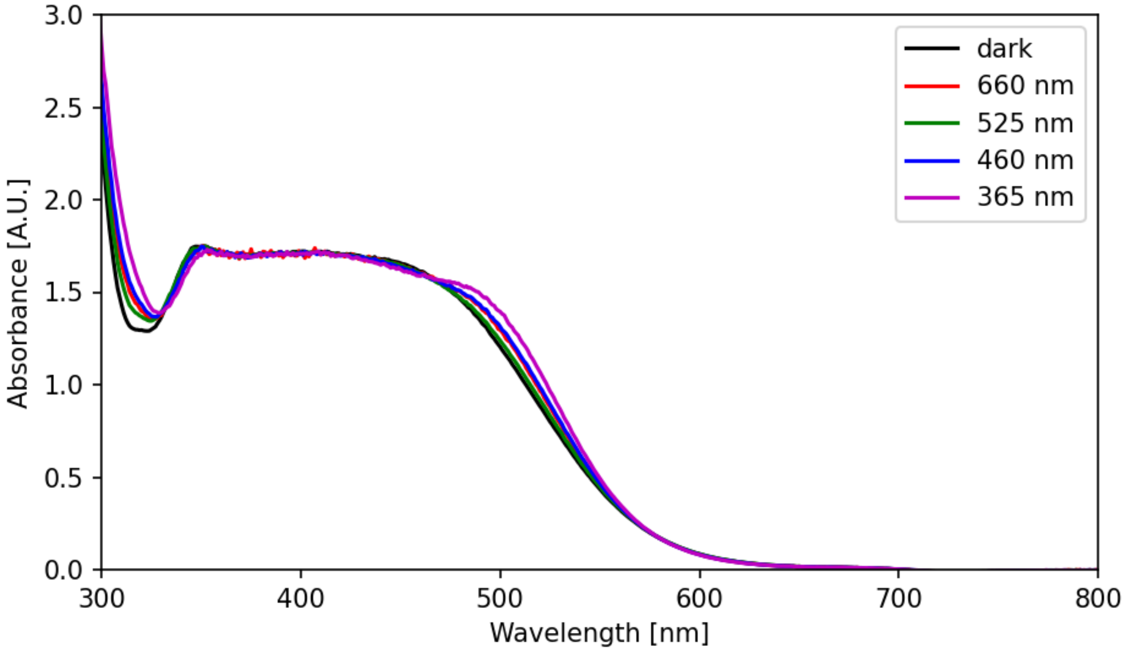

(*E*/*Z*)-3-Chloro-4-((2-chloro-6-fluoro-4-hydroxyphenyl)diazenyl)-5-fluorobenzoic acid (49, 50 µM, DMSO/H_2O 9:1)

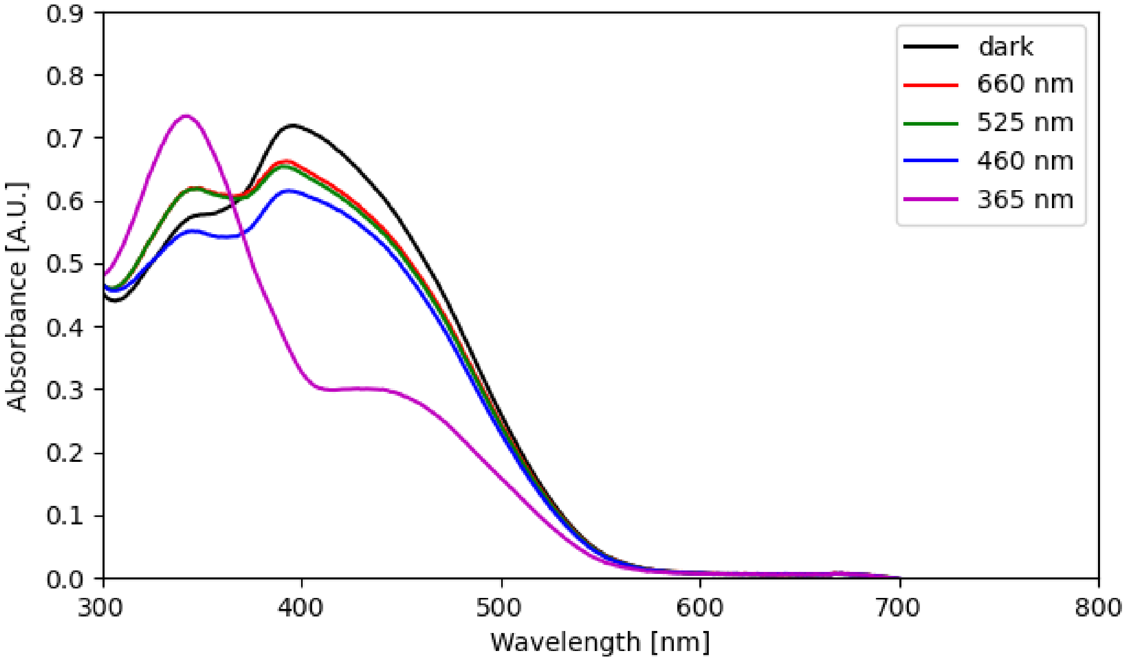

(*E*/*Z*)-3-Chloro-4-((2-chloro-6-fluoro-4-hydroxyphenyl)diazenyl)-5-fluorobenzoic acid (49, 500 µM, DMSO-d_6/D_2O 9:1)

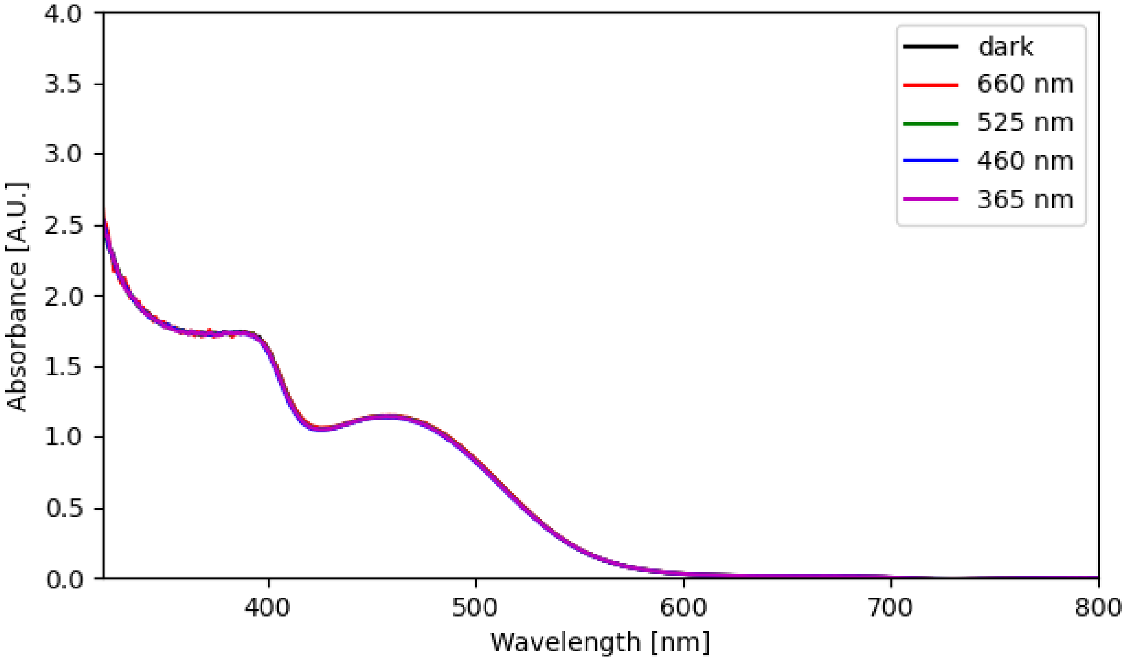

(*E*/*Z*)-1-(1-(4-(4-((4-Butyl-2-chloro-6-fluorophenyl)diazenyl)-3-chloro-5-fluorophenyl)butanoyl)-piperidin-4-yl)-1,3-dihydro-2*H*-benzo[*d*]imidazol-2-one (dfdc-OptoBI-1, 50 µM, DMSO/D_2O 9:1)

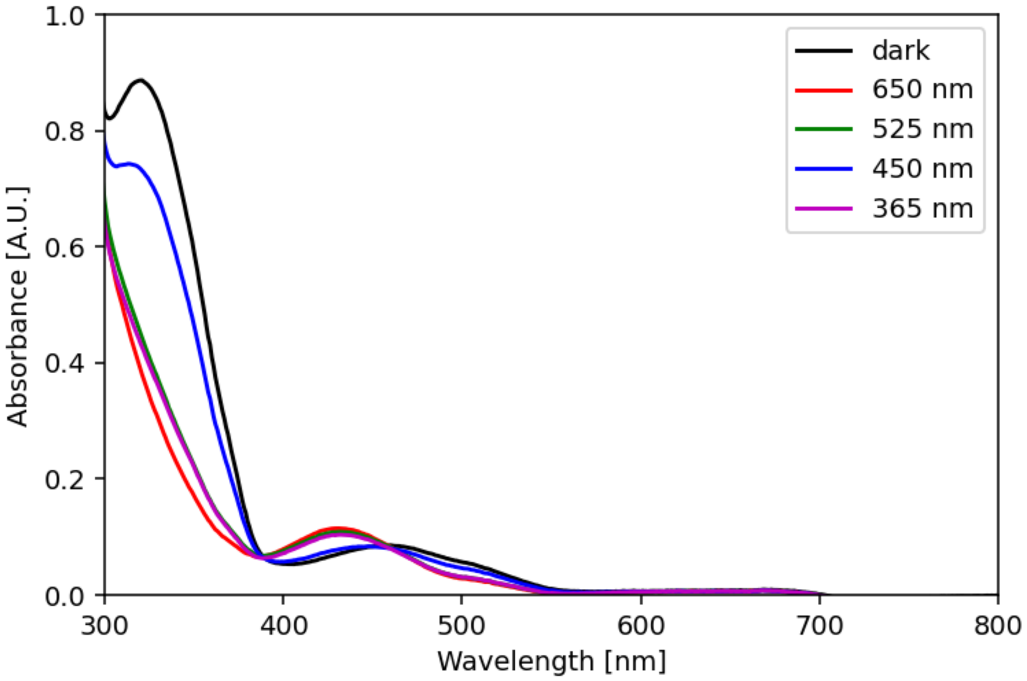

(*E*/*Z*)-1-(1-(4-(4-((4-Butyl-2-chloro-6-fluorophenyl)diazenyl)-3-chloro-5-fluorophenyl)butanoyl)-piperidin-4-yl)-1,3-dihydro-2*H*-benzo[*d*]imidazol-2-one (dfdc-OptoBI-1, 50 µM, DMSO-d_6/D_2O 9:1)

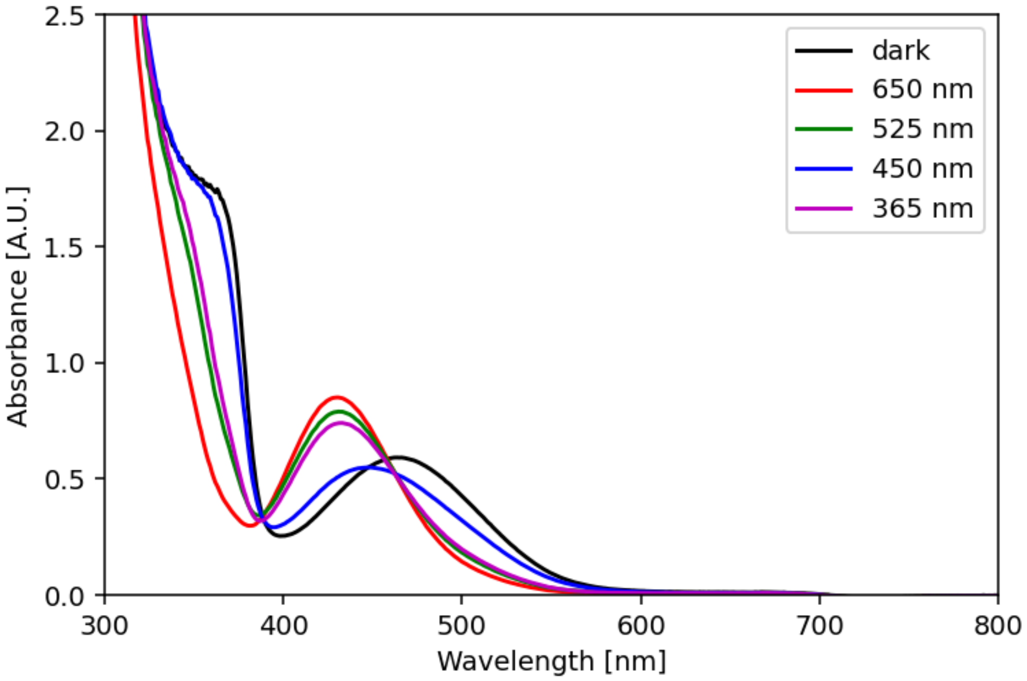

3. UV-vis Data - Reversible Photoswitching

To determine the stability of the azobenzenes, the azobenzenes were irradiated with alternating wavelengths to change between their *cis*- and *trans*-form while simultaneously measuring the absorbance at a Cary 60 UV-vis spectrophotometer over different time intervalls.

(*E*/*Z*)-Azobenzene (1, 500 µM, DMSO-d_6/D_2O 9:1)

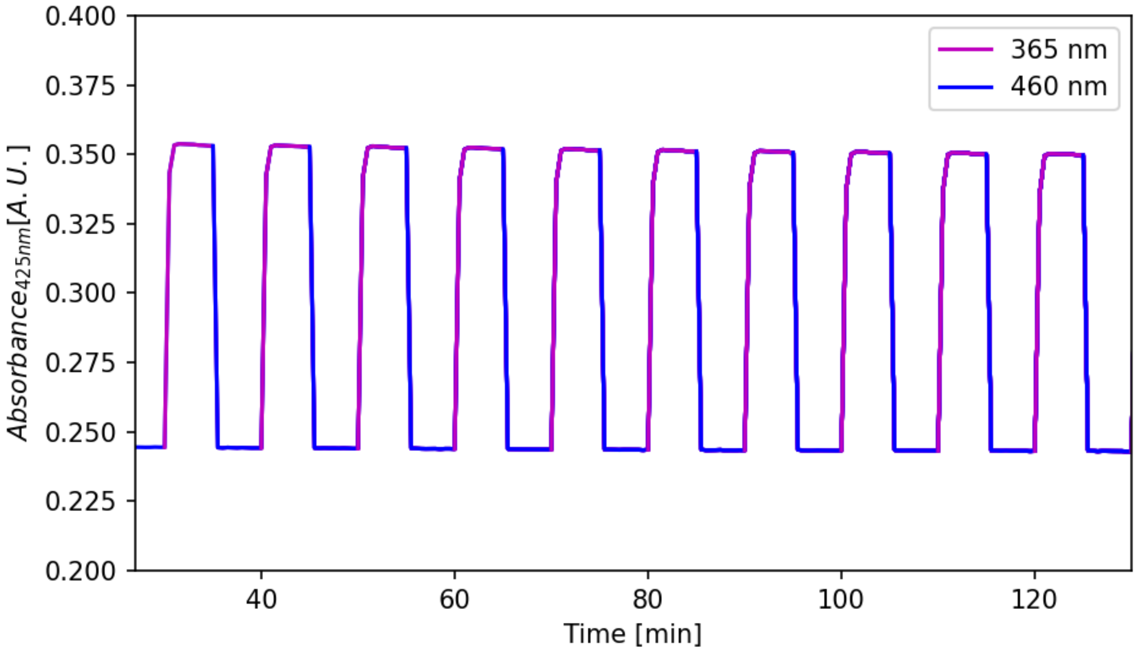

(*E*/*Z*)-1,2-Bis(2,6-difluorophenyl)diazene (2, 50 µM, DMSO/H_2O 9:1)

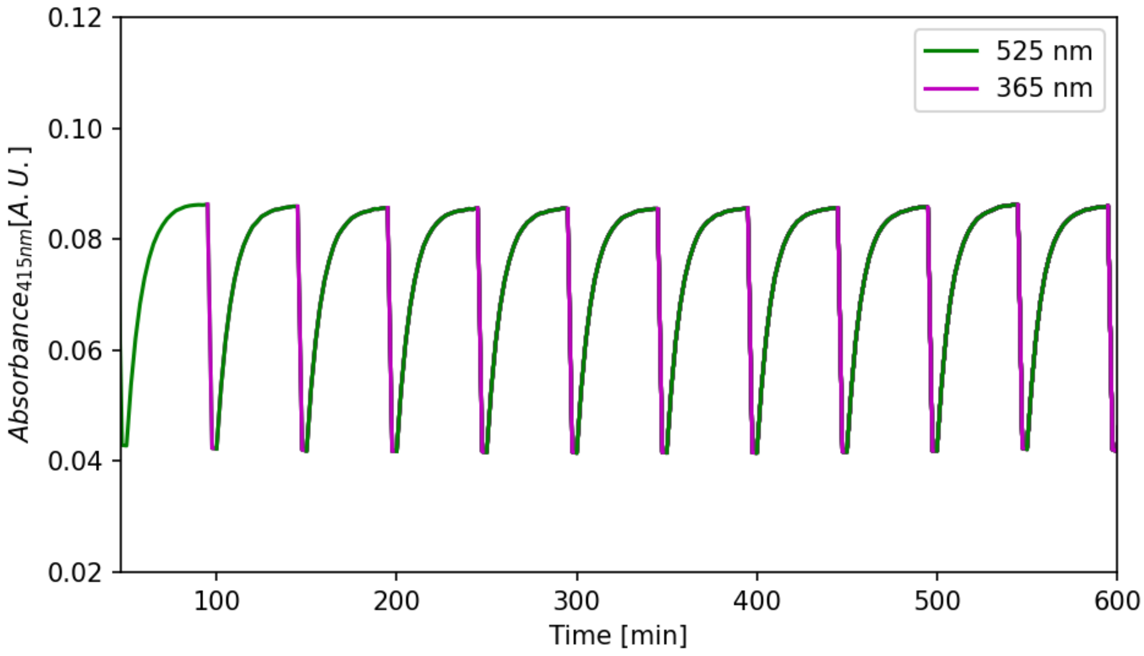

(*E*/*Z*)-1,2-Bis(2-chloro-6-fluorophenyl)diazene (3, 50 µM, DMSO/H_2O 9:1)

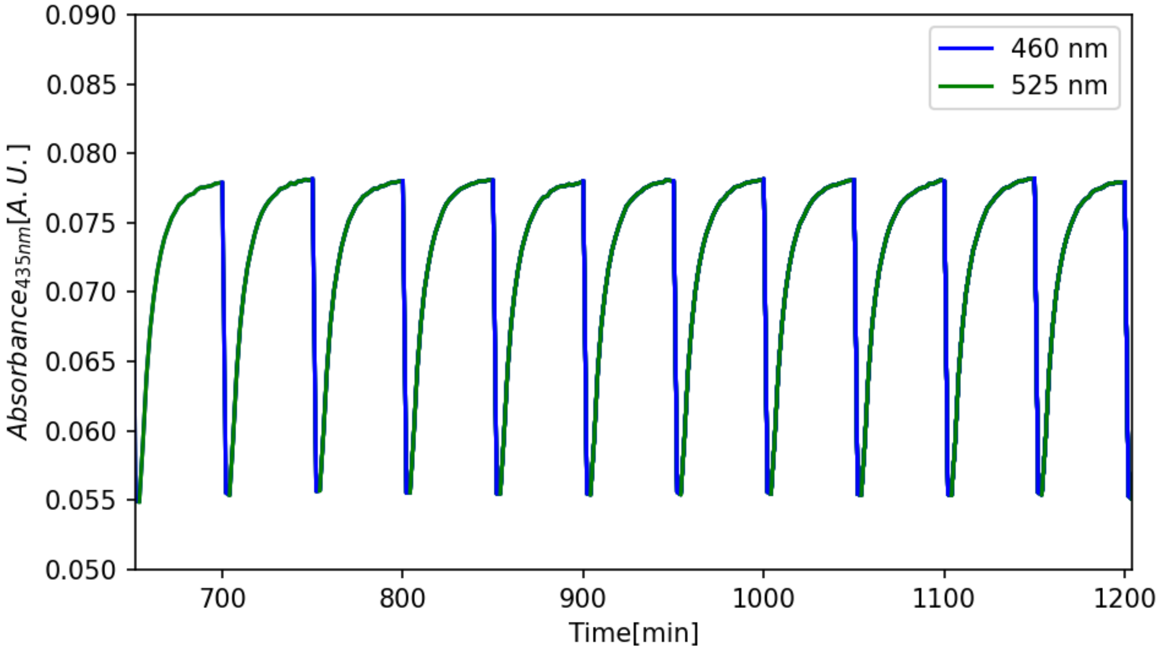

(*E*/*Z*)-1,2-Bis(2-bromo-6-fluorophenyl)diazene (4, 500 µM, DMSO-d_6/D_2O 9:1)

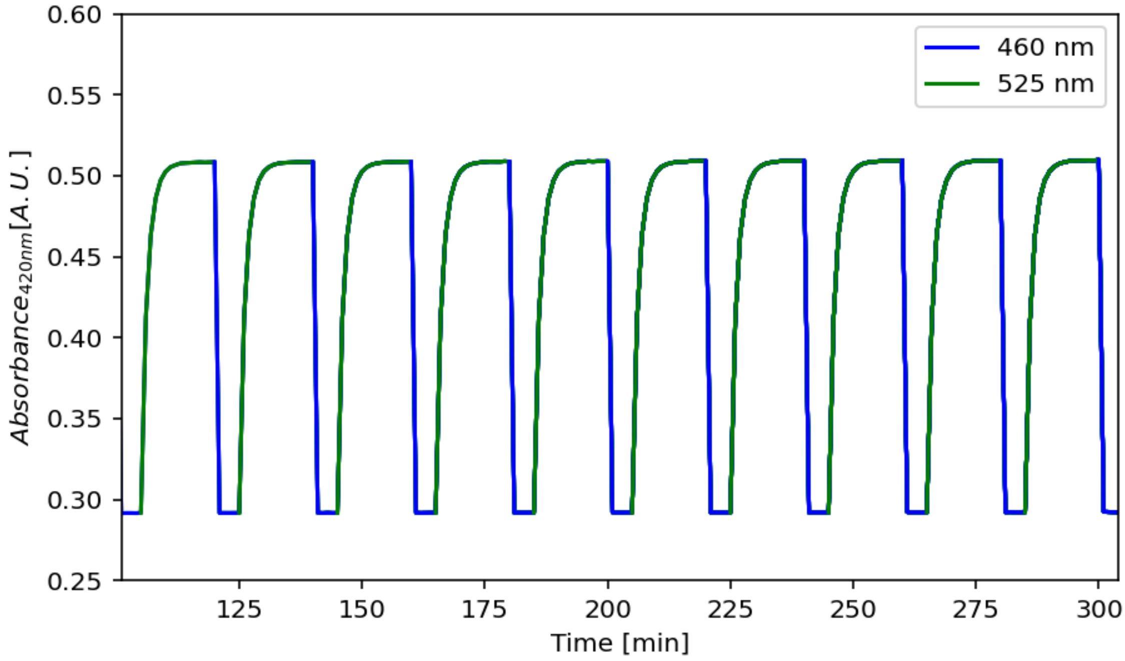

(*E*/*Z*)-1,2-Bis(2,6-dichlorophenyl)diazene (6, 500 µM, DMSO-d_6/D_2O 9:1)

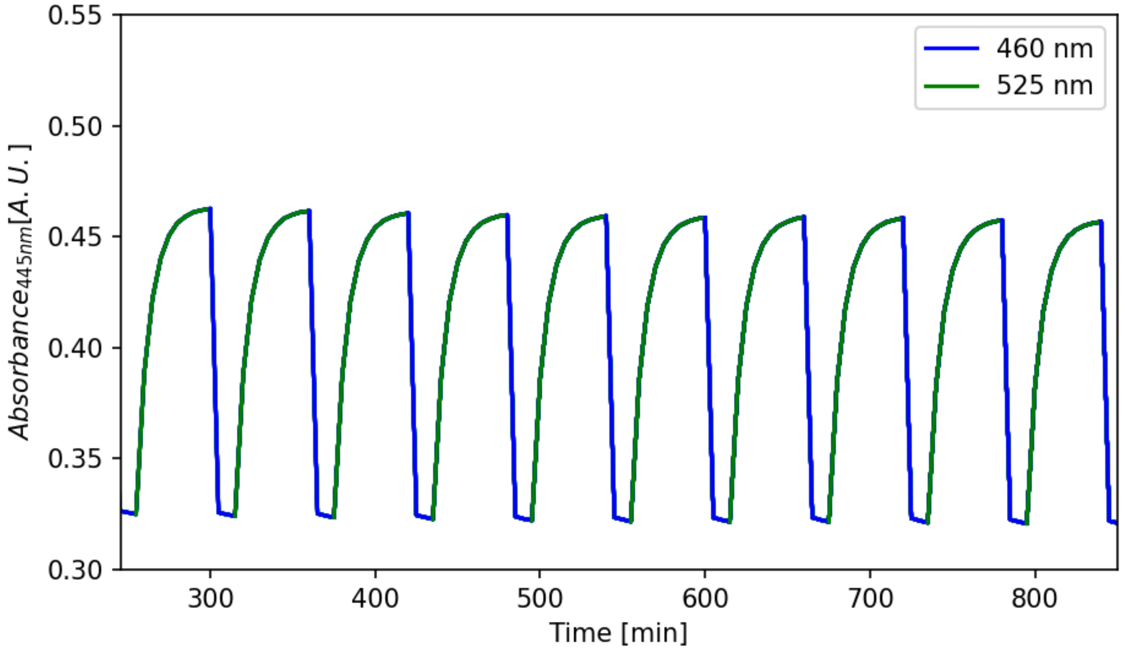

(*E*/*Z*)-1,2-Bis(2,6-dimethoxyphenyl)diazene (9, 500 µM, DMSO-d_6/D_2O 9:1)

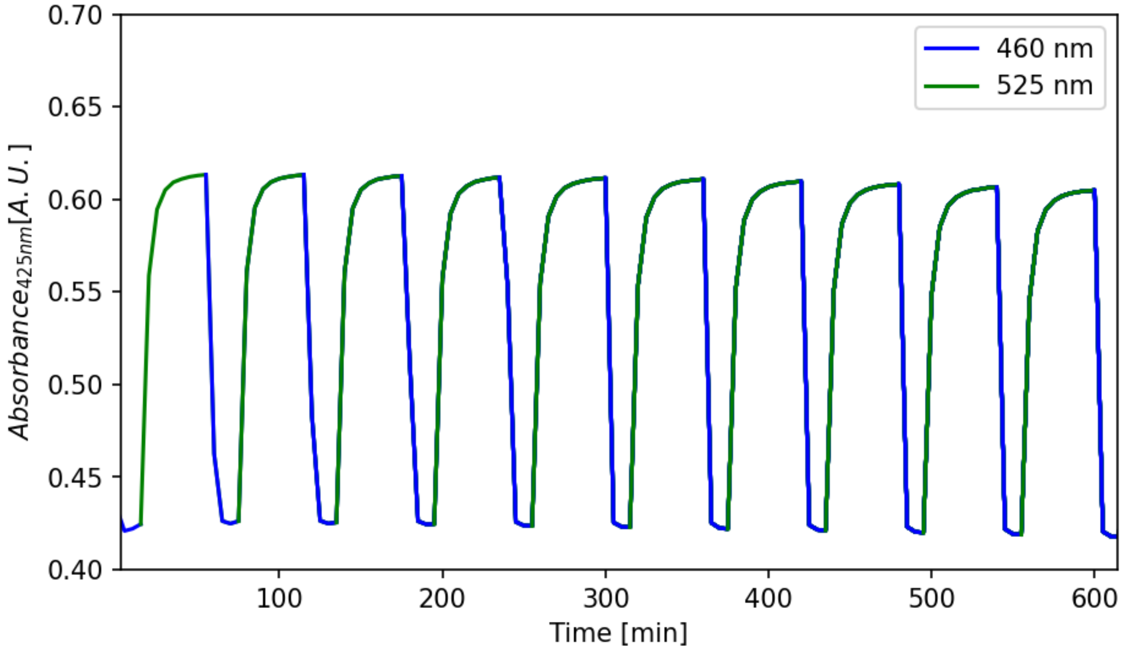

(*E*/*Z*)-1,2-Bis(3-chloro-1-fluoronaphthalen-2-yl)diazene (12, 500 µM, DMSO-d_6/D_2O 9:1)

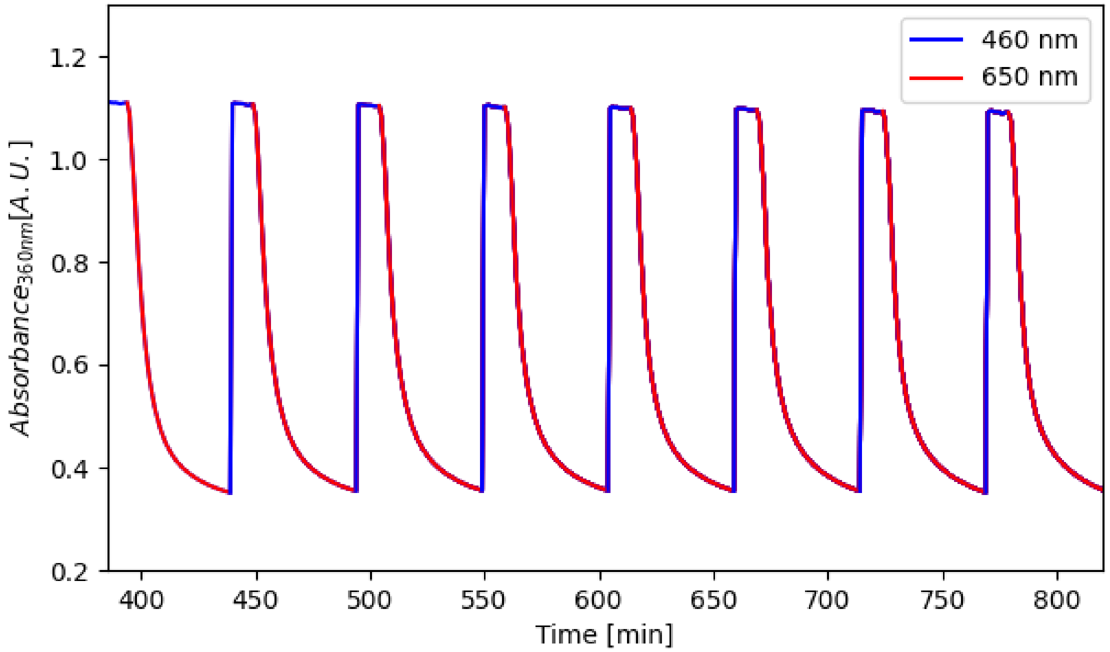

(*E*/*Z*)-3-Chloro-4-((2-chloro-6-fluorophenyl)diazenyl)-5-fluorobenzoic acid (17, 500 µM, DMSO-d_6/D_2O 9:1)

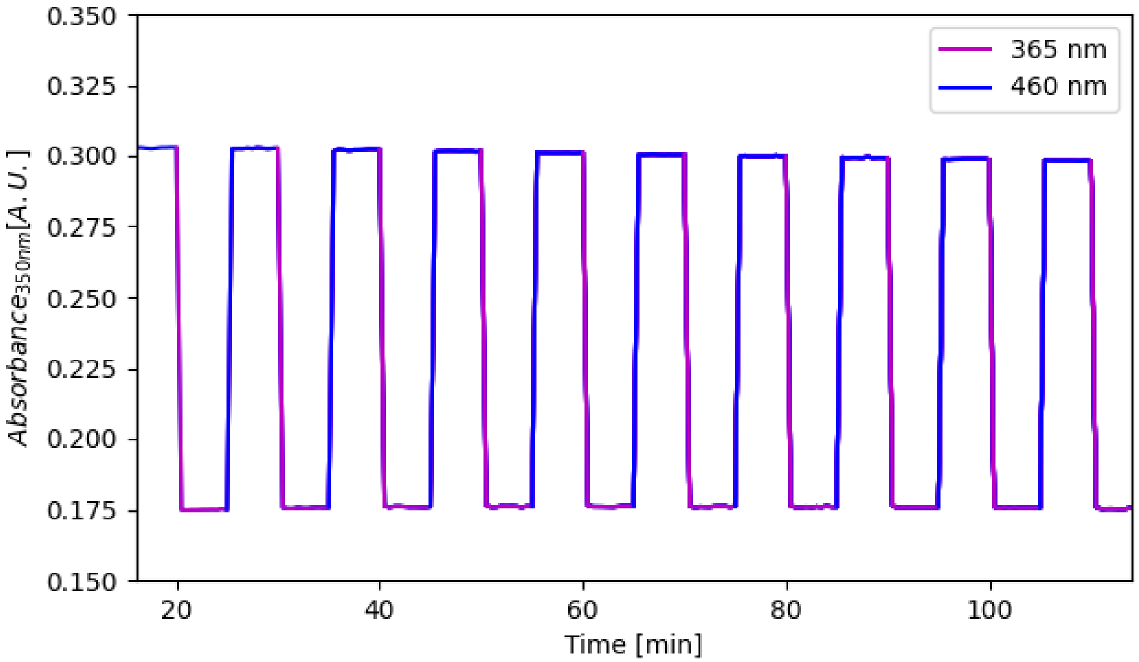

(*E*/*Z*)-1-(2-Chloro-6-fluoro-4-nitrophenyl)-2-(2-chloro-6-fluorophenyl)diazene (19, 50 µM, DMSO/H_2O 9:1)

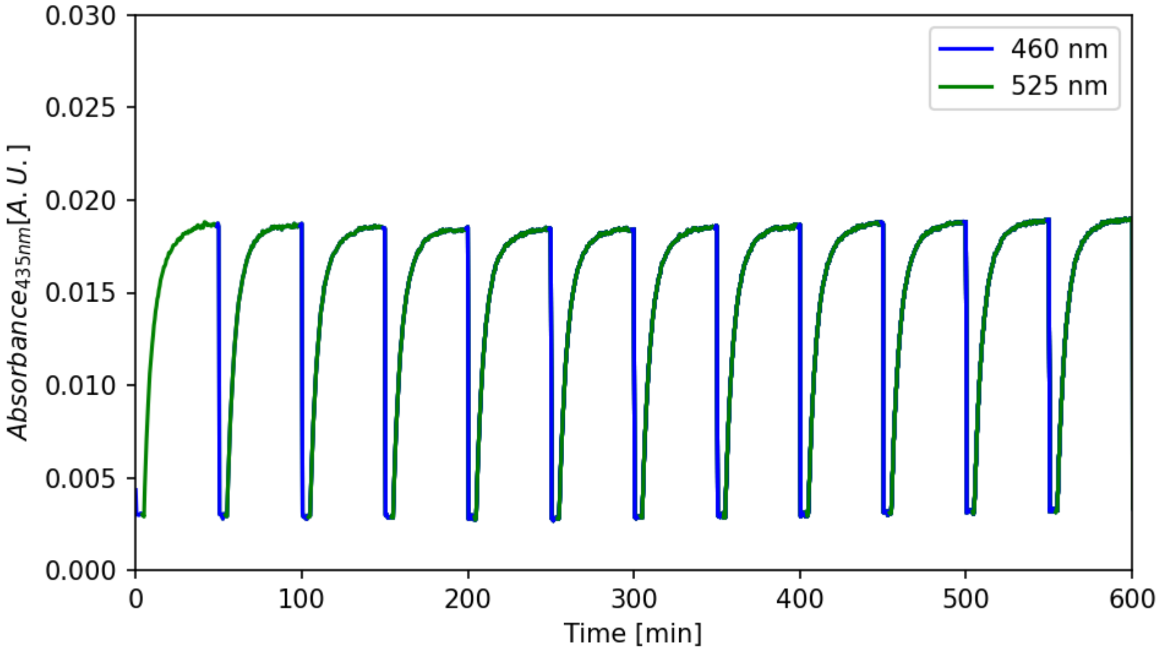

(*E*/*Z*)-3-Chloro-4-((2-chloro-6-fluoro-4-nitrophenyl)diazenyl)-5-fluorobenzoic acid (20, 50 µM, DMSO/H_2O 9:1)

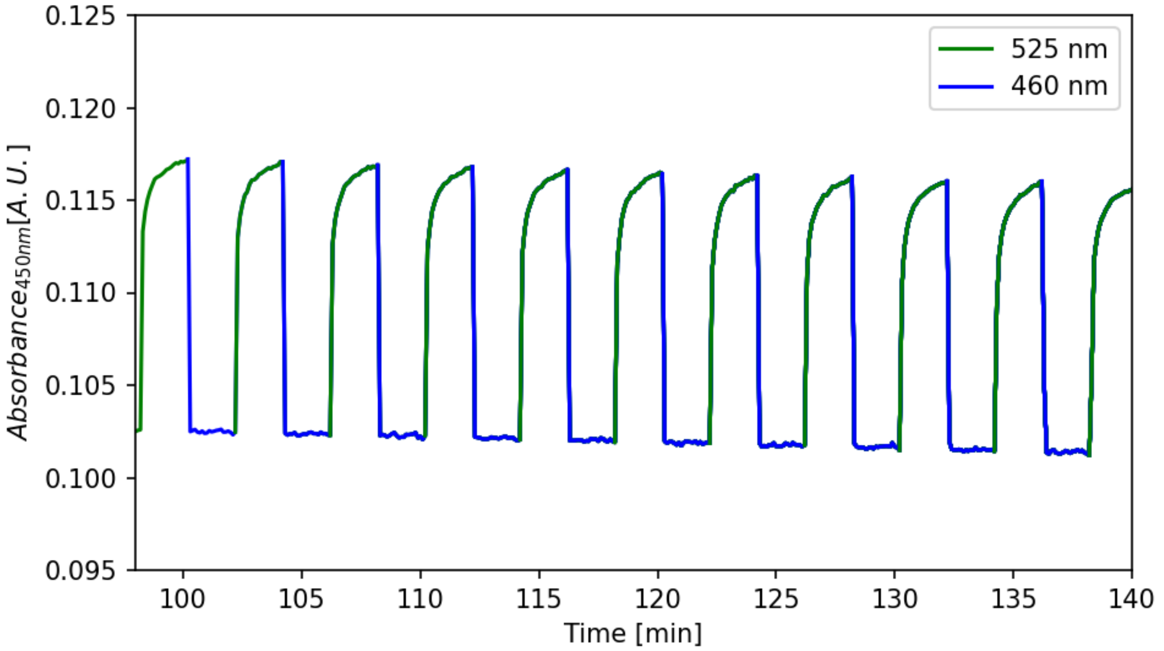

(*E*/*Z*)-Methyl-3-chloro-4-((2-chloro-6-fluorophenyl)diazenyl)-5-fluorobenzoate (21, 500 µM, DMSO-*d*$_6$/D$_2$O 9:1)

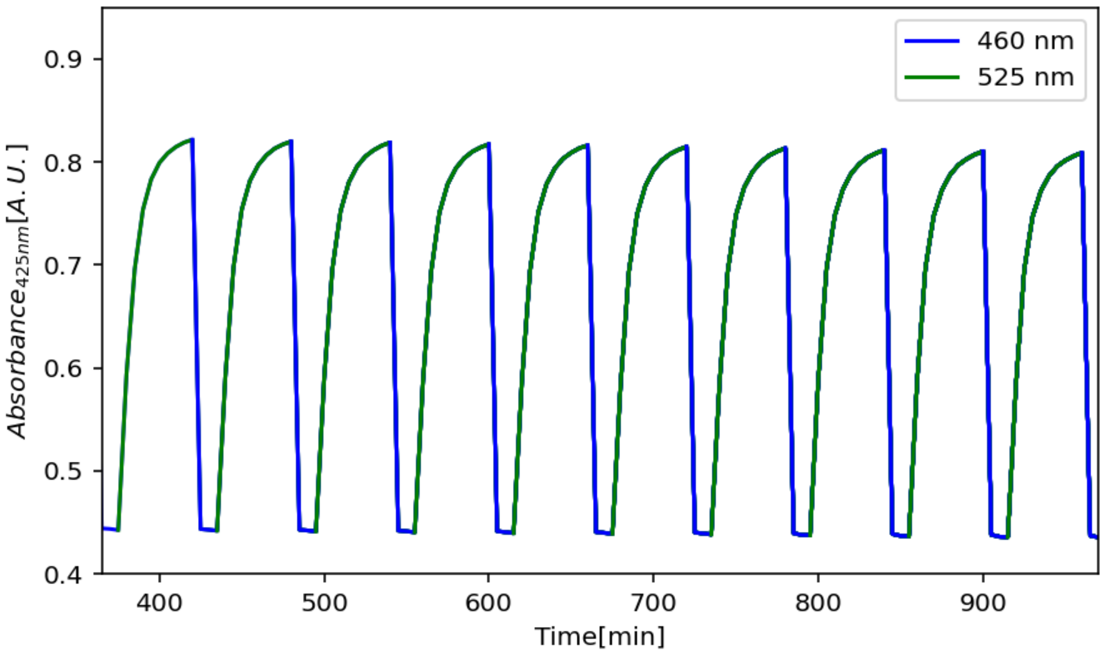

(*E*/*Z*)-Methyl-3-bromo-4-((2-bromo-6-fluorophenyl)diazenyl)-5-fluorobenzoate (22, 500 µM, DMSO-*d*$_6$/D$_2$O 9:1)

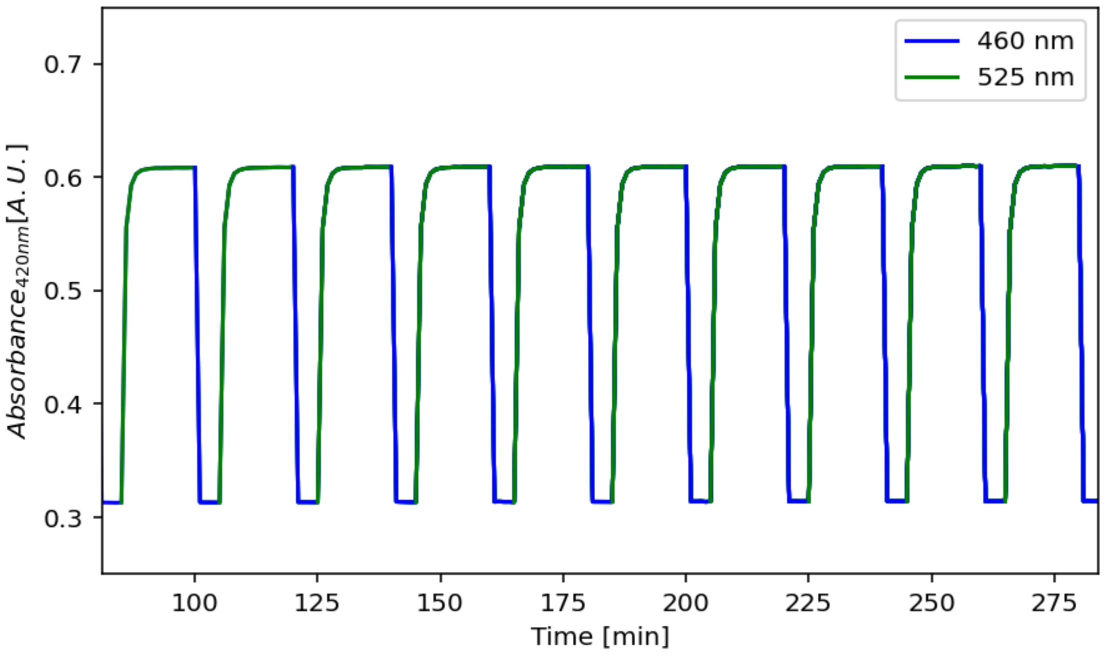

(*E*/*Z*)-Methyl-3,5-dichloro-4-((2,6-dichlorophenyl)diazenyl)benzoate (23, 500 µM, DMSO-d_6/D_2O 9:1)

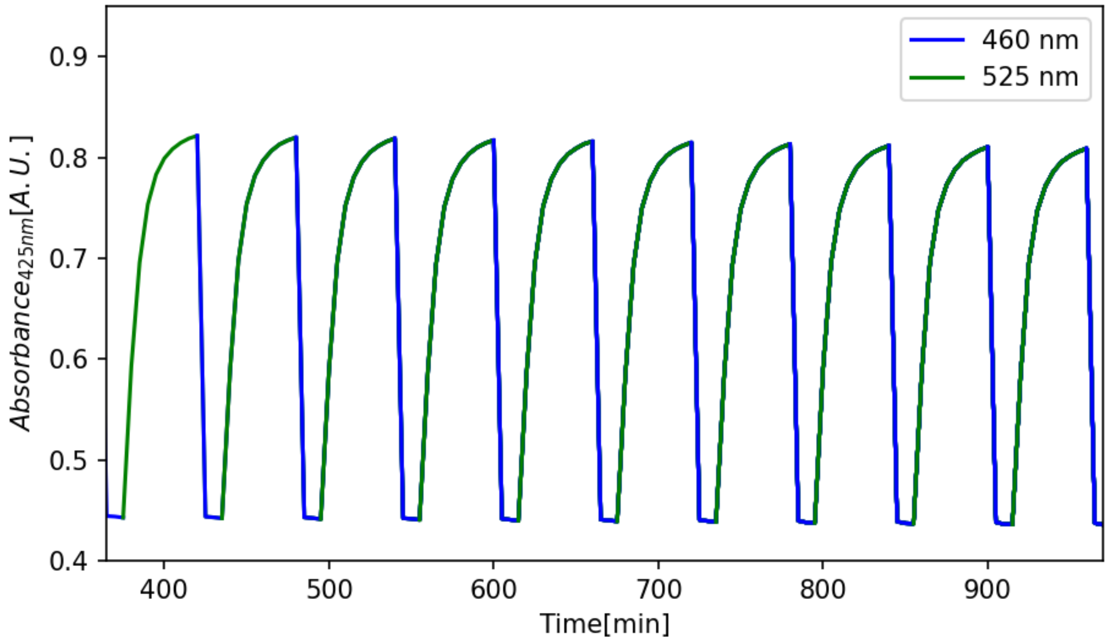

(*E*/*Z*)-3-Chloro-4-((2-chloro-6-fluorophenyl)diazenyl)-5-fluoro-N-methylbenzamide (24, 500 µM, DMSO-d_6/D_2O 9:1)

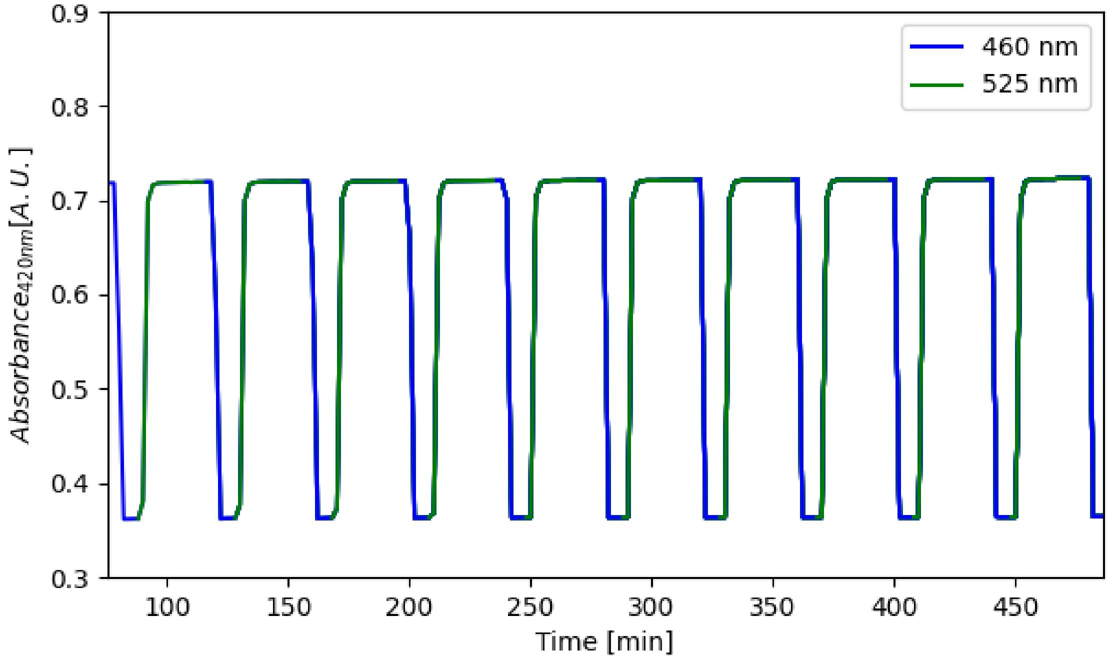

(*E*/*Z*)-Dimethyl 4,4'-(diazene-1,2-diyl)-bis(3-chloro-5-fluorobenzoate) (28, 50 µM, DMSO/H_2O 9:1)

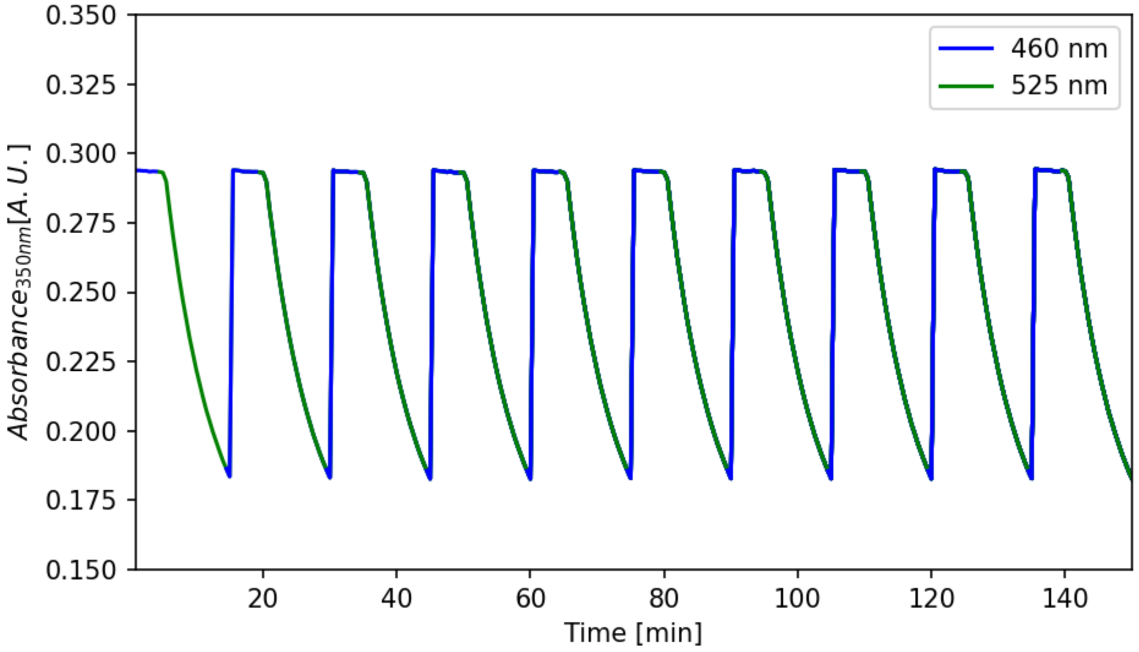

(*E*/*Z*)-3-Chloro-4-((2-chloro-6-fluorophenyl)diazenyl)-5-fluoroaniline (38, 50 µM, DMSO/H_2O 9:1)

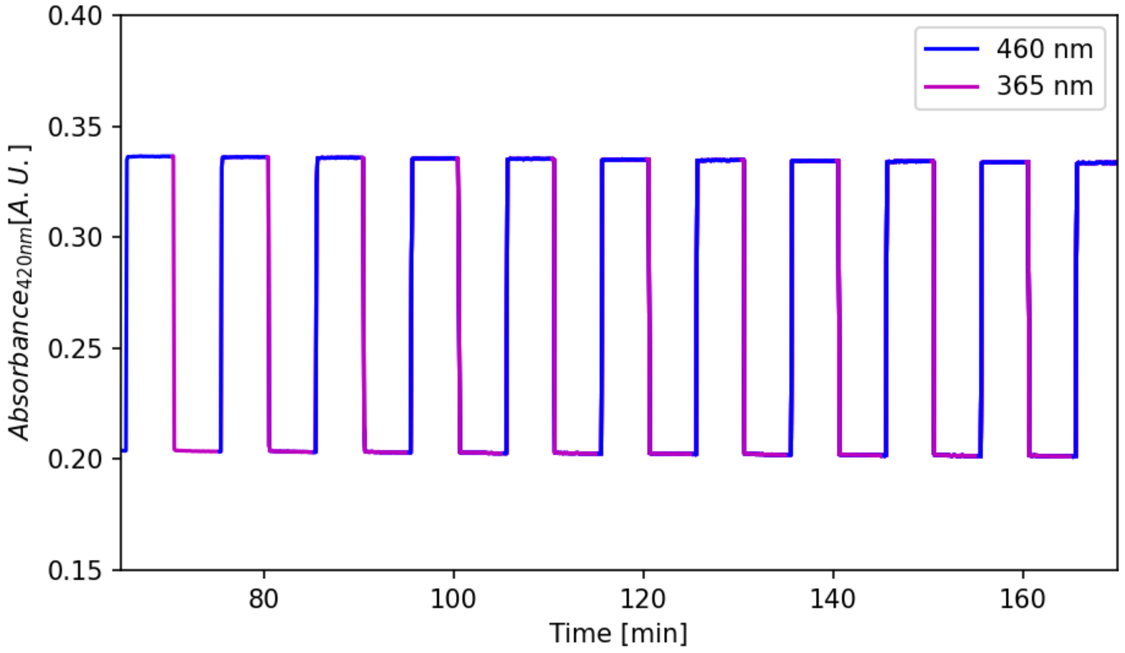

(*E*/*Z*)-*N*-(3-chloro-4-((2-chloro-6-fluorophenyl)diazenyl)-5-fluorophenyl)acetamid (39, 50 µM, DMSO/H_2O 9:1)

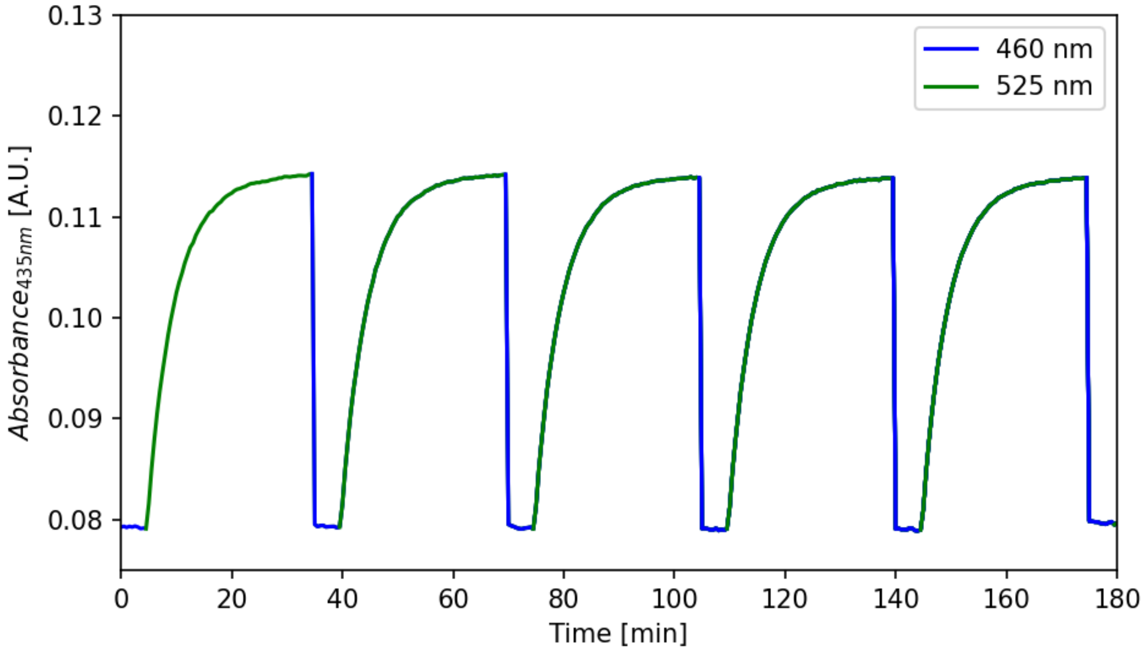

(*E*/*Z*)-Methyl-3-chloro-4-((2-chloro-6-fluoro-4-methoxyphenyl)diazenyl)-5-fluorobenzoate (46, 500 µM, DMSO-*d*$_6$/D_2O 9:1)

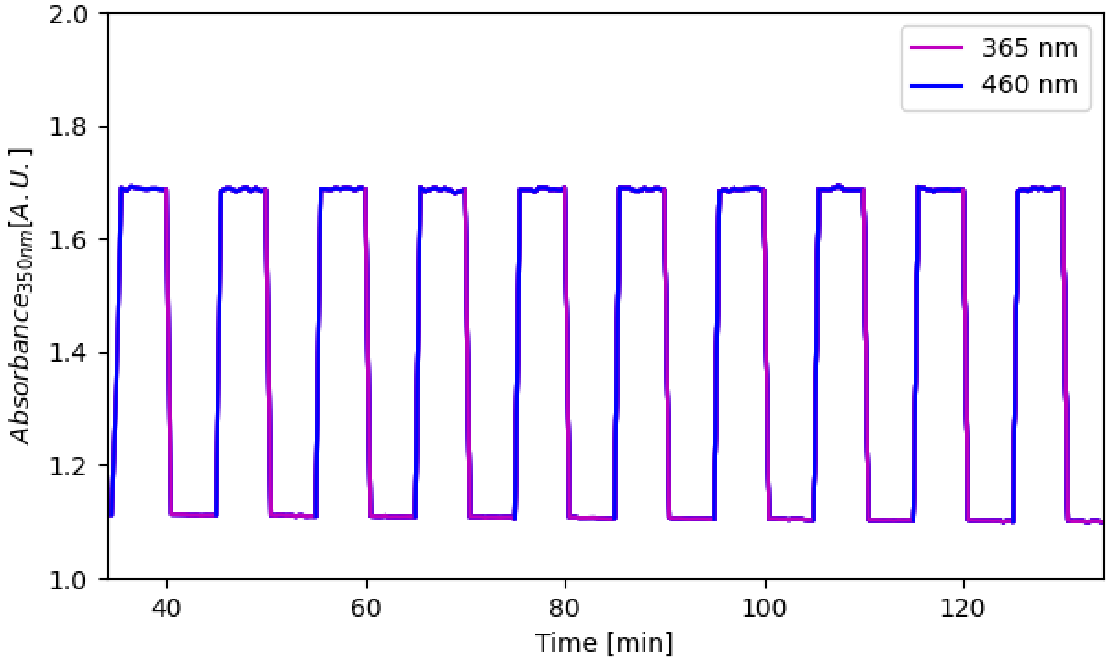

(*E*/*Z*)-4-((4-Amino-2,6-dichlorophenyl)diazenyl)-3,5-dichlorobenzenesulfonamide (48, 50 µM, DMSO/H_2O 9:1)

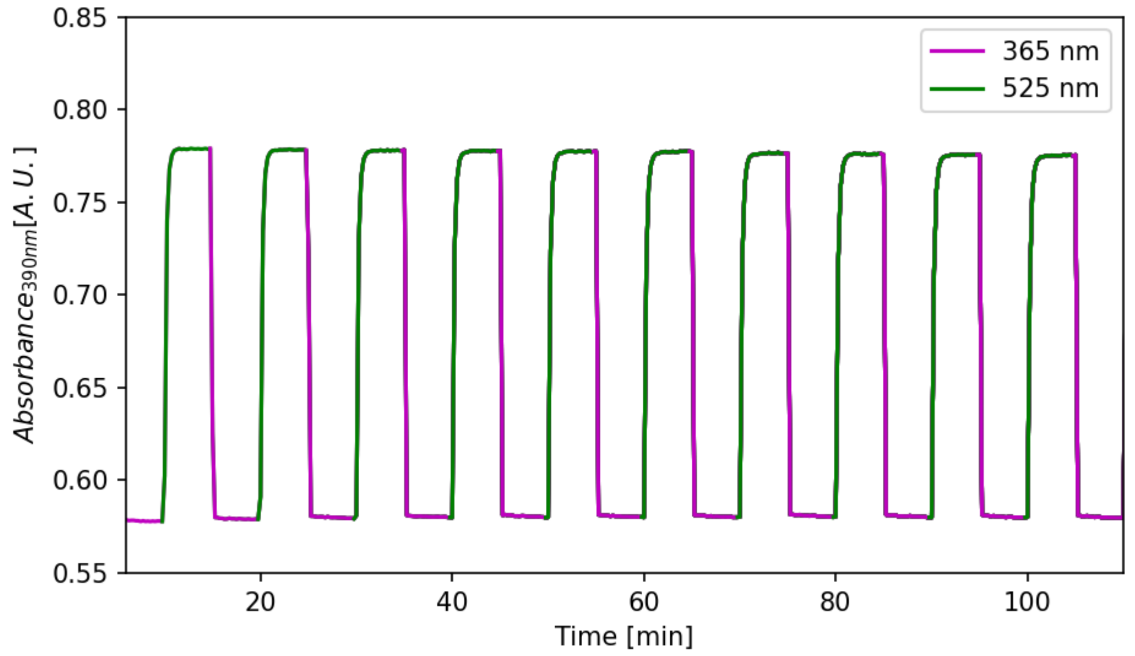

(*E*/*Z*)-3-Chloro-4-((2-chloro-6-fluoro-4-hydroxyphenyl)diazenyl)-5-fluorobenzoic acid (49, 50 µM, DMSO/H_2O 9:1)

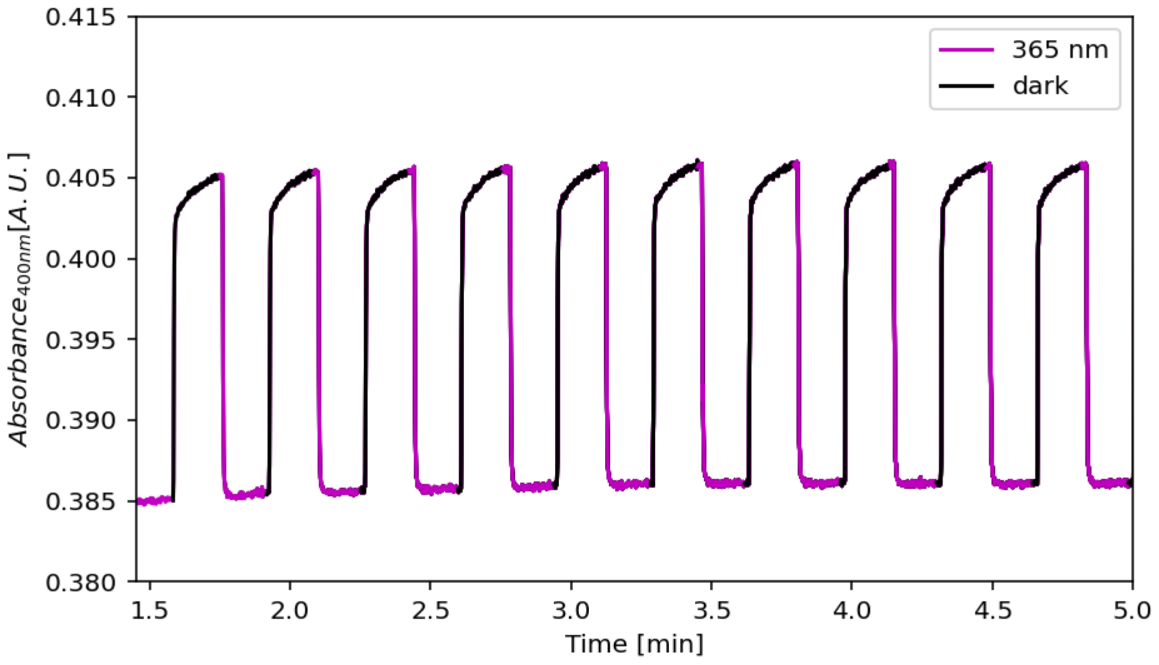

(*E*/*Z*)-1-(1-(4-(4-((4-Butyl-2-chloro-6-fluorophenyl)diazenyl)-3-chloro-5-fluorophenyl)butanoyl)-piperidin-4-yl)-1,3-dihydro-2*H*-benzo[*d*]imidazol-2-one (dfdc-OptoBI-1, 500 μM, DMSO-d_6/D_2O 9:1)

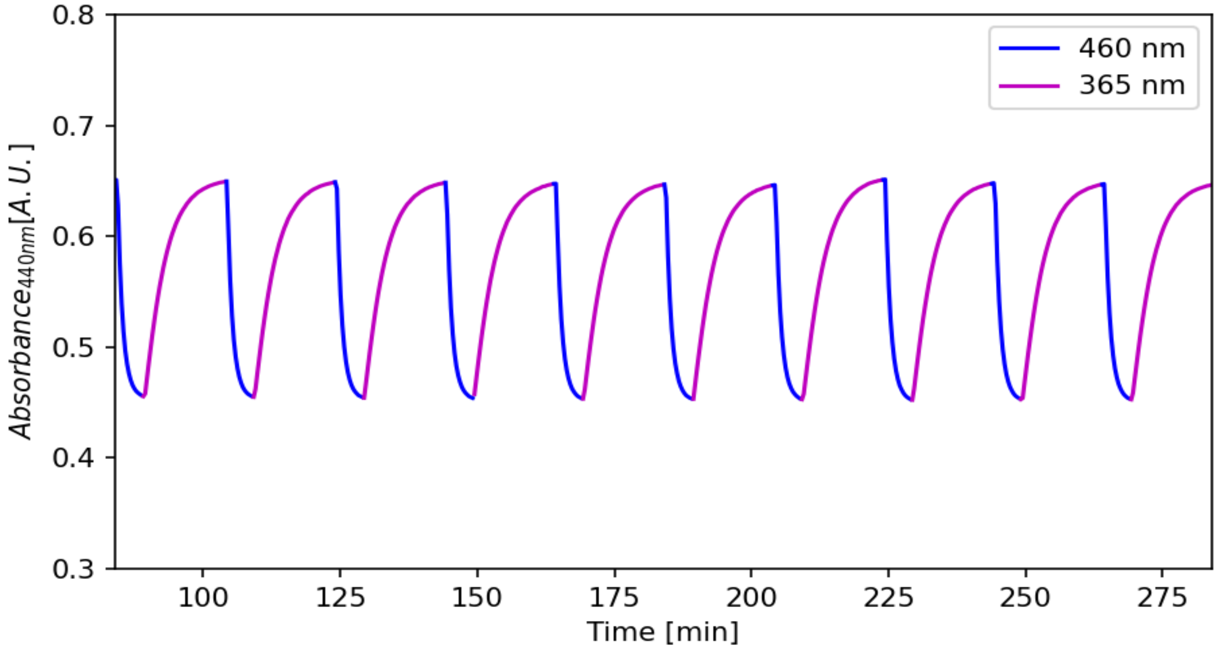

4. UV-vis Data – Stability against GSH

The azobenzene derivative was dissolved in 588 µL of a DMSO/PBS pH 7 (8:2) solution to give a concentration of 51 µM. To assess differences in the stability towards GSH based on the conformation of the photoswitch, the samples were studied in the *trans* configuration ("dark-adapted") and with a photostationary state (PSS) that contains a high fraction of the *cis* isomer, which was obtained by irradiation. In most cases, the samples were illuminated with a 525 nm LED ("green-adapted"), 460 nm LED (blue-adapted) or 660 nm LED (red-adapted) and measurements were carried out under continuous irradiation. If light sources with different wavelengths were used, they were specified under the corresponding figures. To perform the stability tests, the sample solutions were transferred into a UV-vis cuvette at 37 °C and 12 µL 0.5 mM GSH in PBS pH 7 was added to obtain a final concentration of 10 mM GSH and 50 µM azobenzene. The cuvette was sealed under air with parafilm during the measurement. The analysis was carried out using a *Cary* 60 UV-vis spectrophotometer (Agilent) for several hours and changes in the absorption spectra were measured at different time intervals.

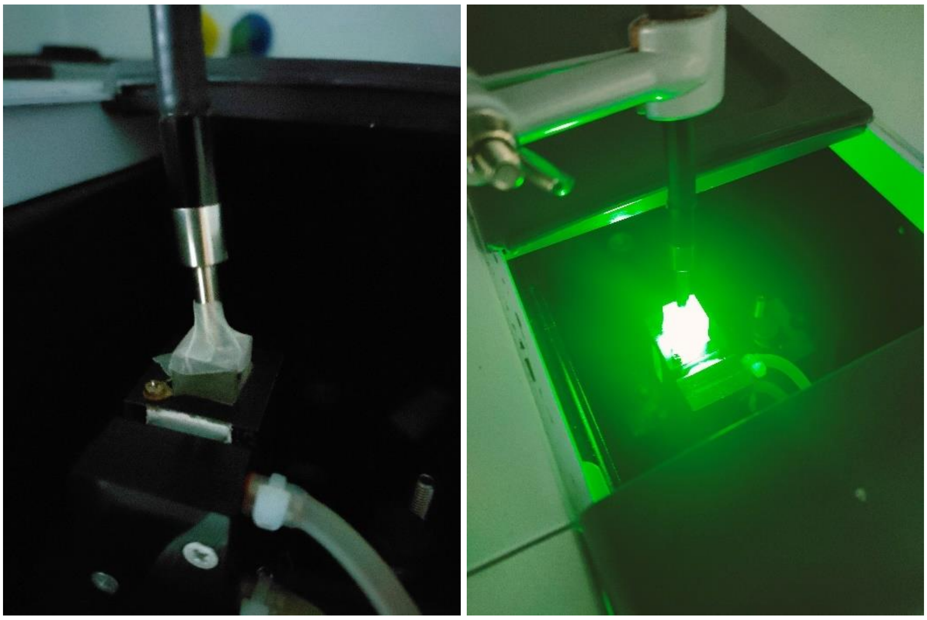

GSH reference (10 mM, DMSO/PBS pH 7 8:2, dark-adapted)

As reference a 10 mM GSH in DMSO/PBS pH 7 (8:2) sample was measured without the addition of an azobenzene derivate to ensure the stability of GSH under the conditions.

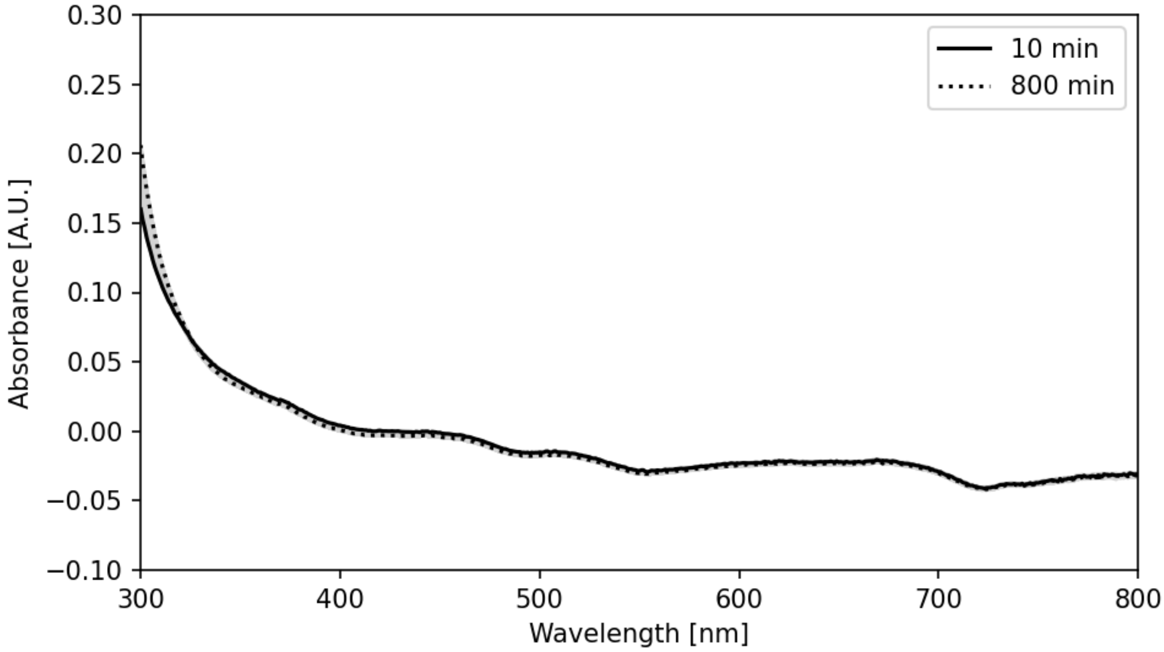

GSH reference (10 mM, DMSO/PBS pH 7 8:2, red-adapted)

As reference a 10 mM GSH in DMSO/PBS pH 7 (8:2) sample was measured without the addition of an azobenzene derivate to ensure the stability of GSH under the conditions.

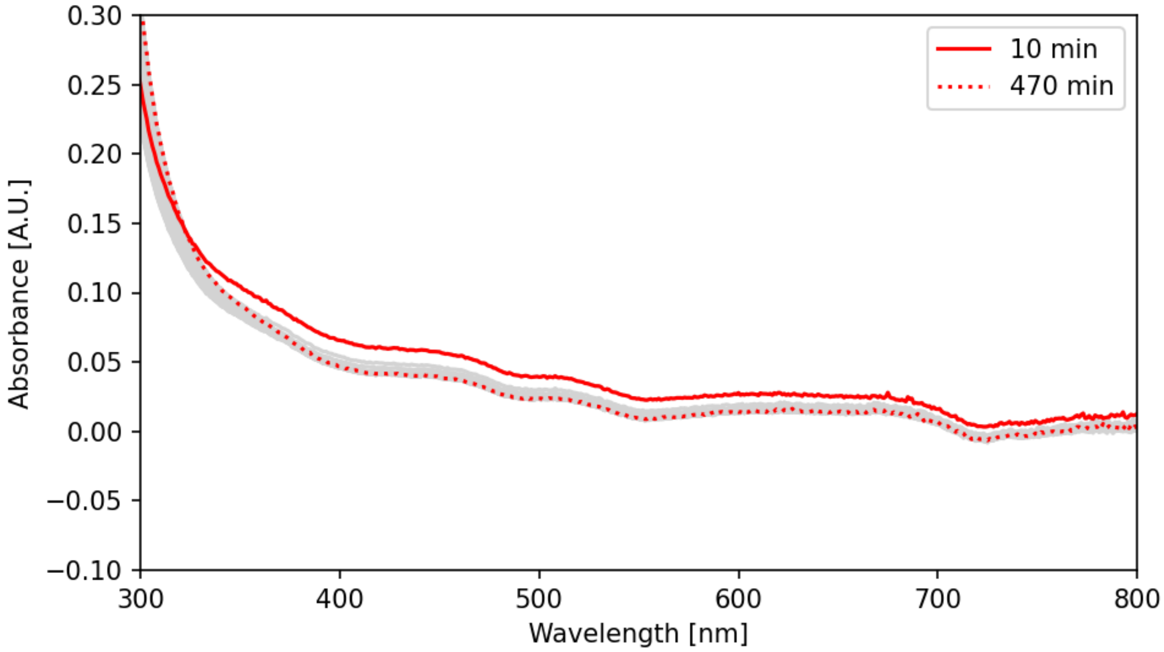

GSH reference (10 mM, DMSO/PBS pH 7 8:2, green-adapted)

As reference a 10 mM GSH in DMSO/PBS pH 7 (8:2) sample was measured without the addition of an azobenzene derivate to ensure the stability of GSH under the conditions.

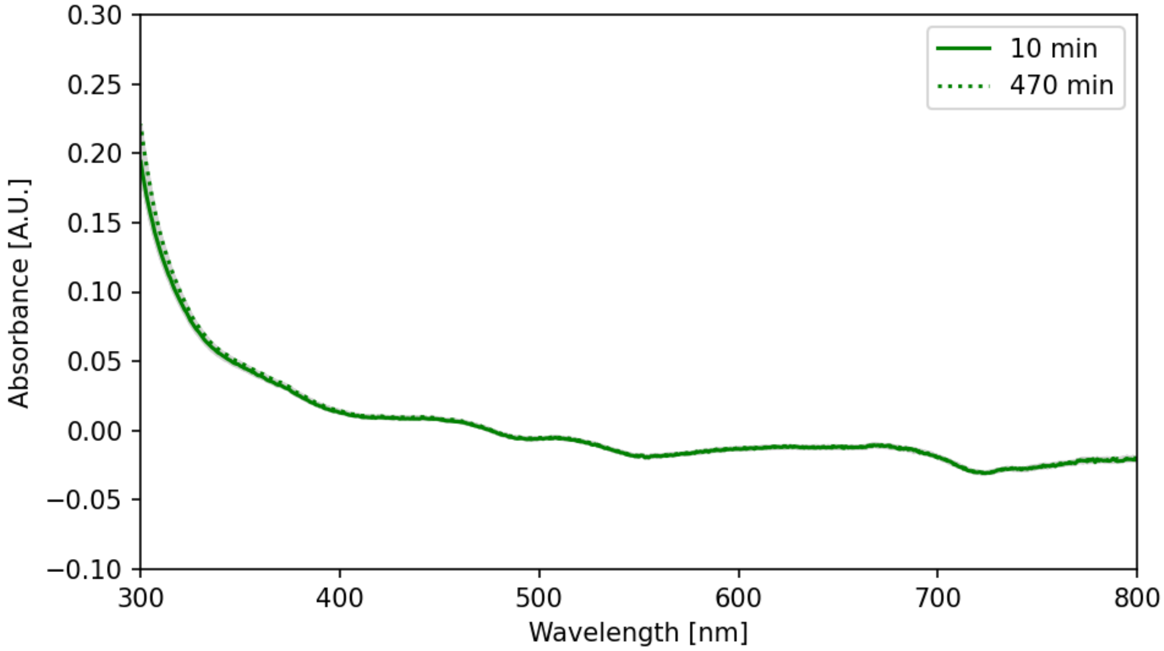

GSH reference (10 mM, DMSO/PBS pH 7 8:2, blue-adapted)

As reference a 10 mM GSH in DMSO/PBS pH 7 (8:2) sample was measured without the addition of an azobenzene derivate to ensure the stability of GSH under the conditions.

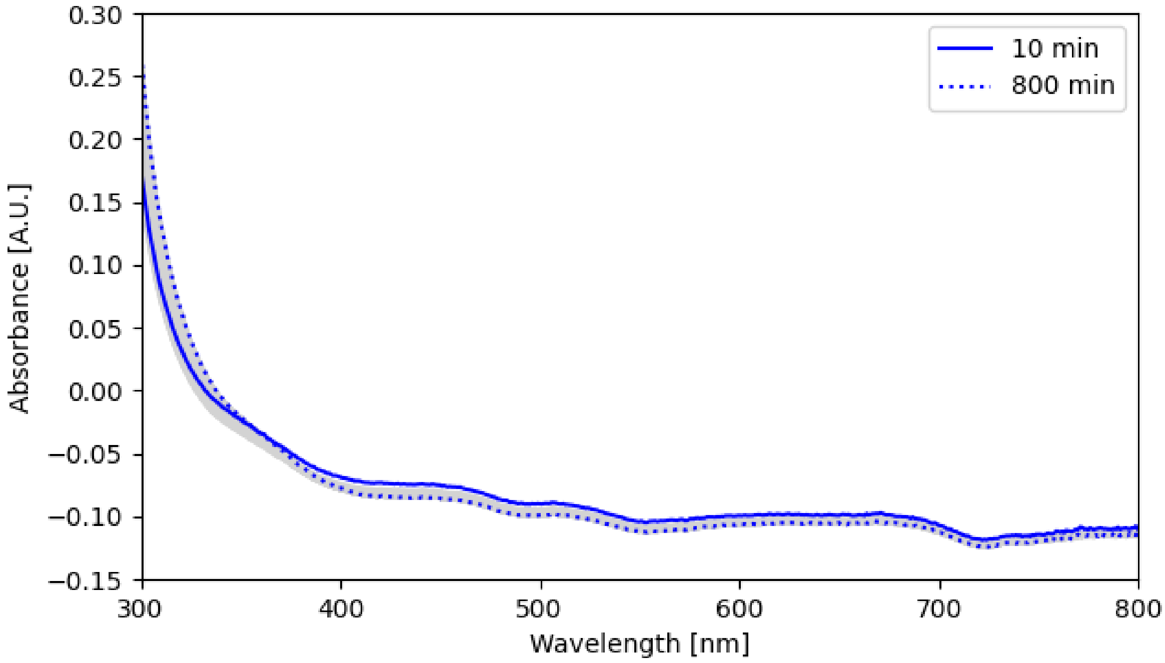

GSH reference (10 mM, DMSO/PBS pH 7 8:2, UV-adapted)

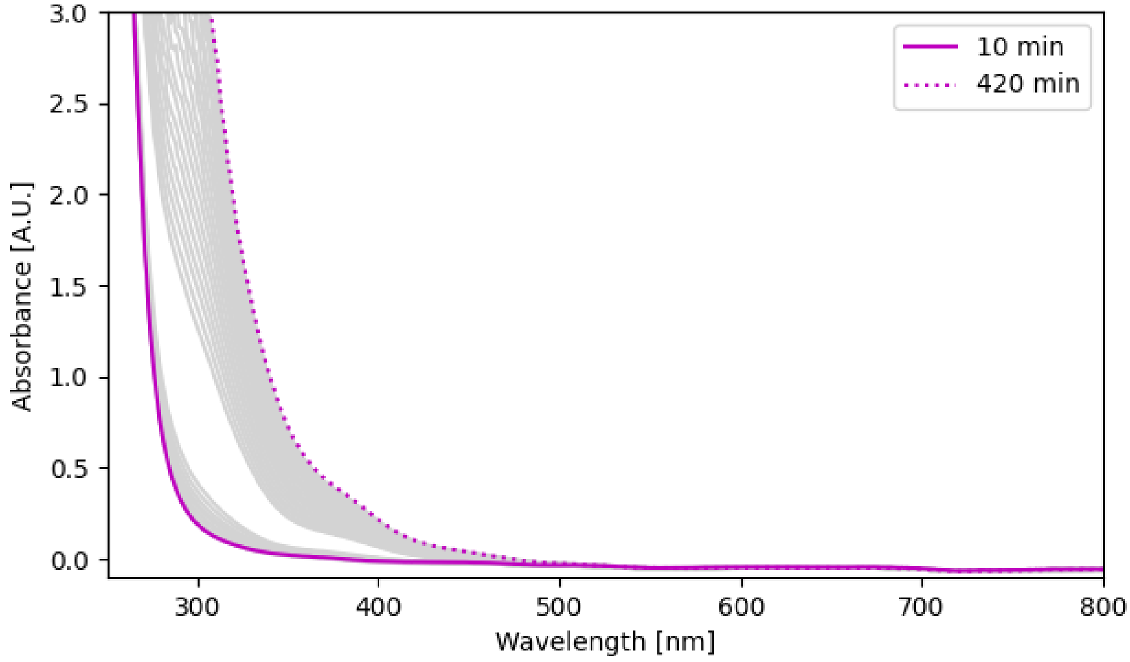

The stability of GSH under 365 nm irridation was evaluated through UV-vis-analysis with $A(t) = A \cdot e^{\frac{-ln(2) \cdot t}{T_{1/2}}} + c$.

→ $t_{1/2}$ (**GSH**, 10 mM, DMSO/PBS pH 7 8:2, 37 °C) = 165 min.

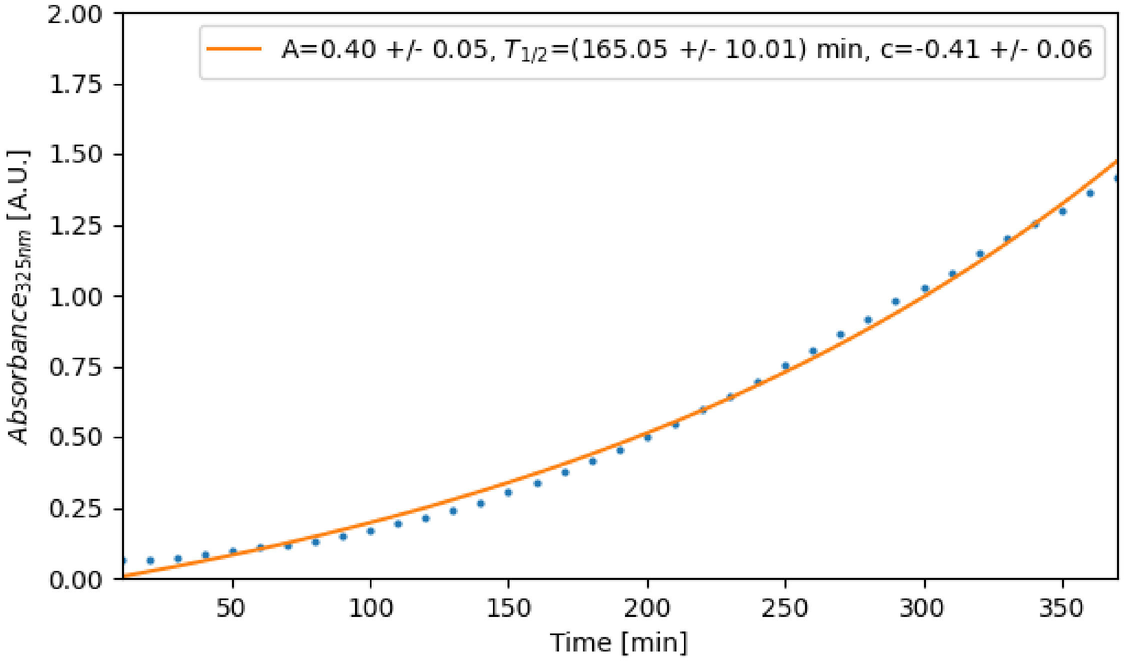

(*E*)-Azobenzene (1, 50 µM, DMSO/PBS pH 7 8:2 with 10 mM GSH, dark-adapted)

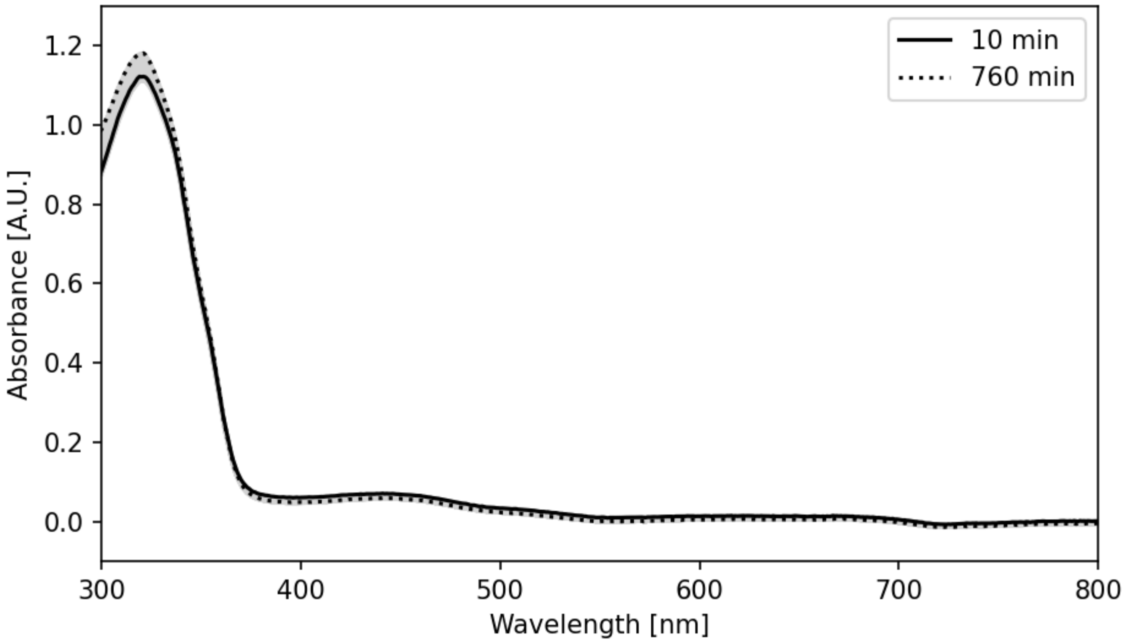

(*Z*)-Azobenzene (1, 50 µM, DMSO/PBS pH 7 8:2 with 10 mM GSH, green-adapted)

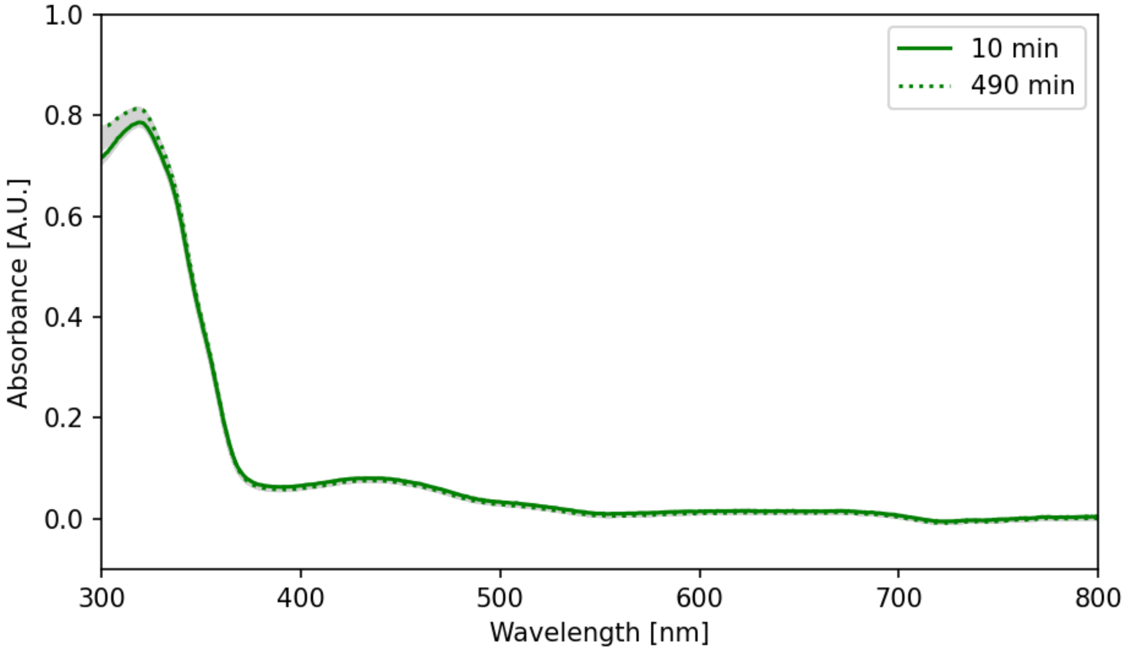

(*E*)-1,2-Bis(2,6-difluorophenyl)diazene (2, 50 µM, DMSO/PBS pH 7 8:2 with 10 mM GSH, dark-adapted)

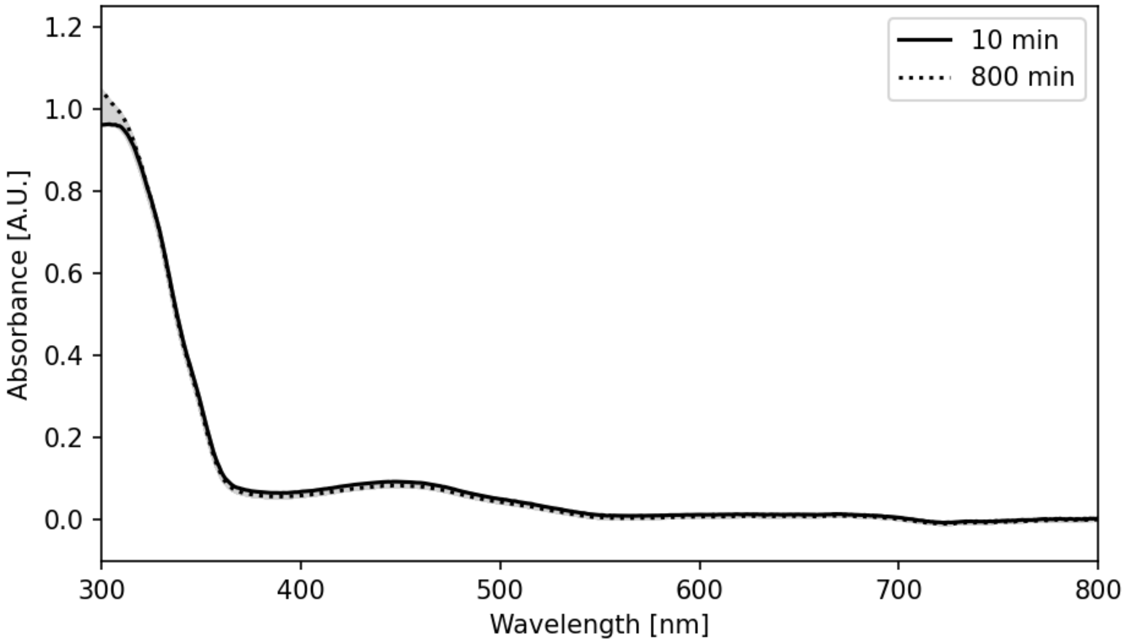

(*Z*)-1,2-Bis(2,6-difluorophenyl)diazene (2, 50 µM, DMSO/PBS pH 7 8:2 with 10 mM GSH, green-adapted)

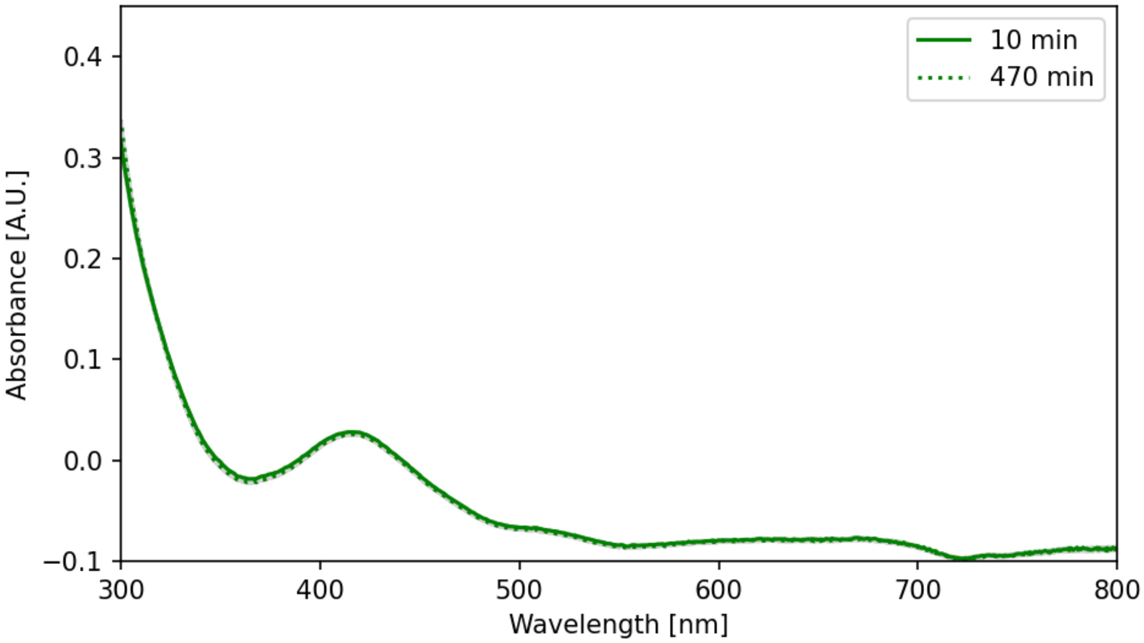

(*E*)-1,2-Bis(2-chloro-6-fluorophenyl)diazene (3, 50 µM, DMSO/PBS pH 7 8:2 with 10 mM GSH, dark-adapted)

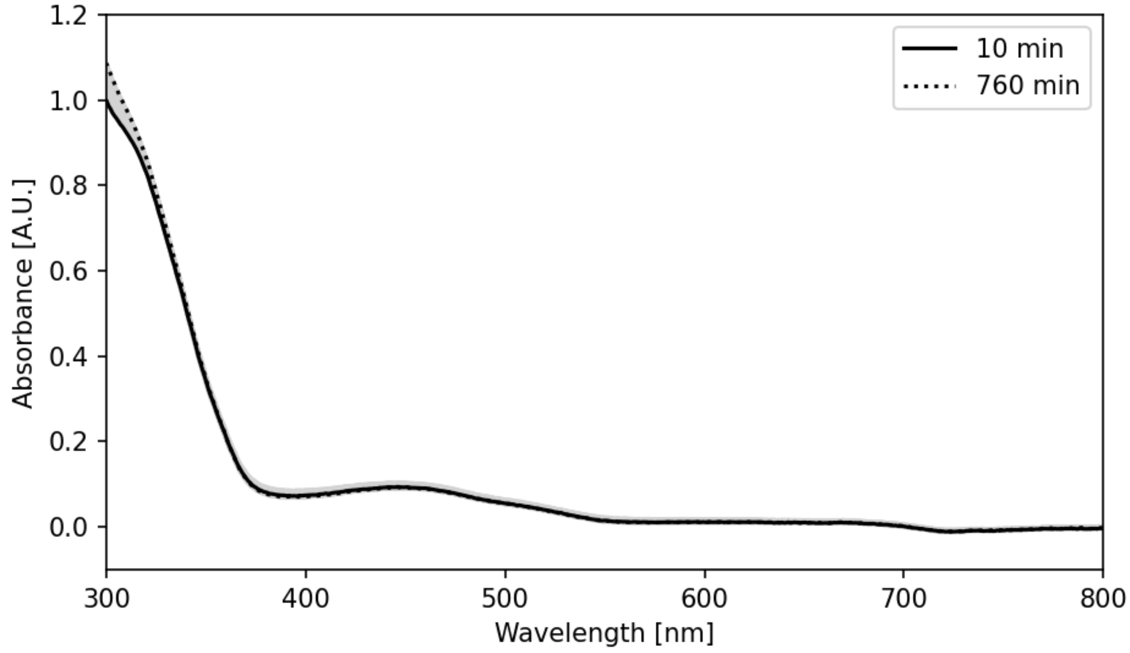

(*Z*)-1,2-Bis(2-chloro-6-fluorophenyl)diazene (3, 50 µM, DMSO/PBS pH 7 8:2 with 10 mM GSH, green-adapted)

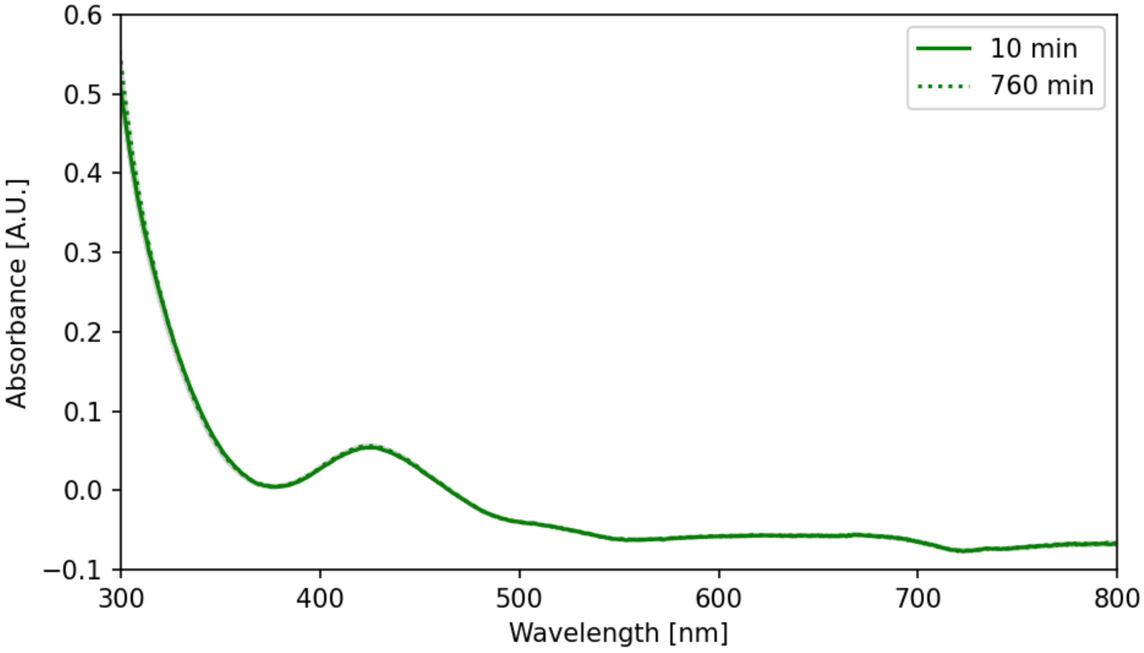

(*E*)-1,2-Bis(2-bromo-6-fluorophenyl)diazene (4, 50 μM, DMSO/PBS pH 7 8:2 with 10 mM GSH, dark-adapted)

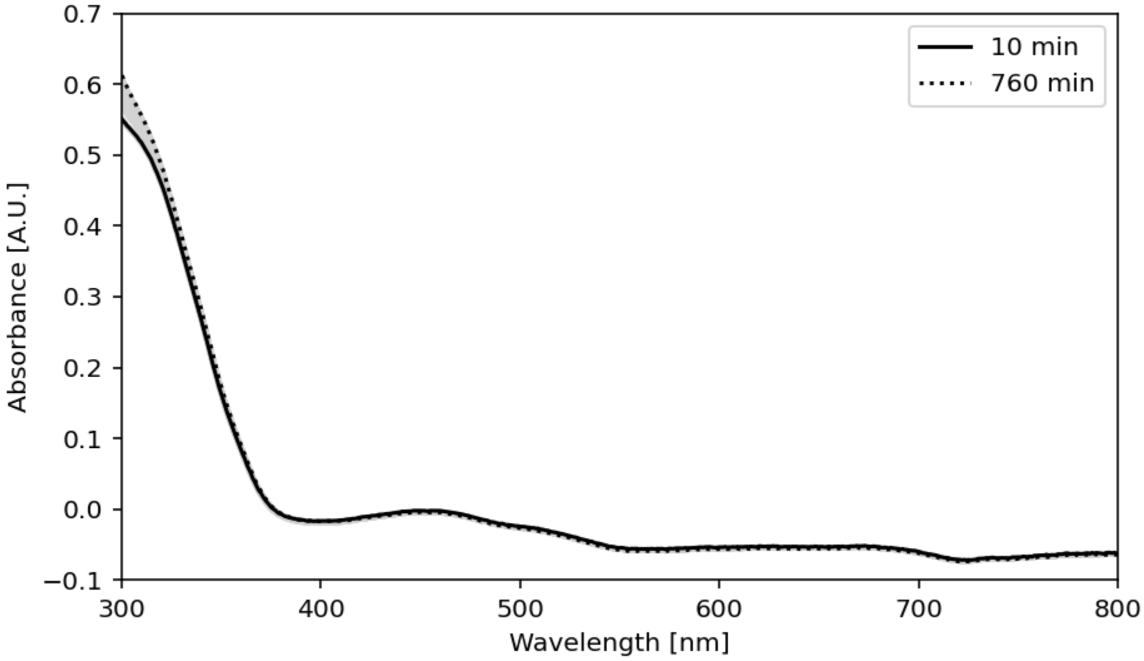

(*Z*)-1,2-Bis(2-bromo-6-fluorophenyl)diazene (4, 50 μM, DMSO/PBS pH 7 8:2 with 10 mM GSH, green-adapted)

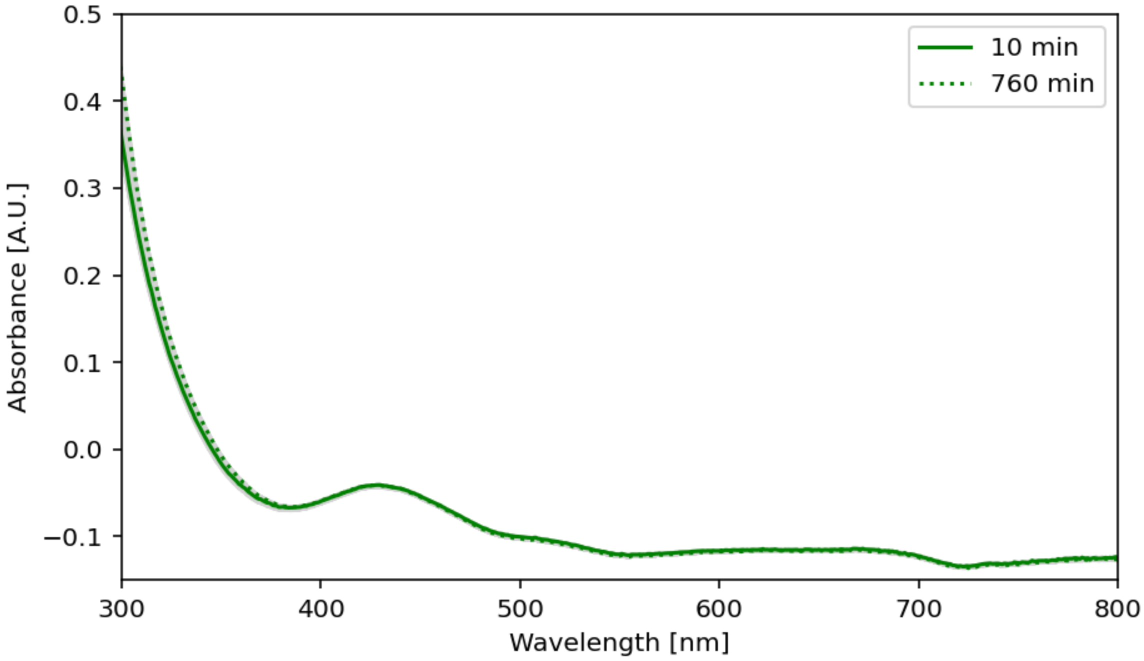

(*E*)-1,2-Bis(2,6-dichlorophenyl)diazene (6, 50 µM, DMSO/PBS pH 7 8:2 with 10 mM GSH, dark-adapted)

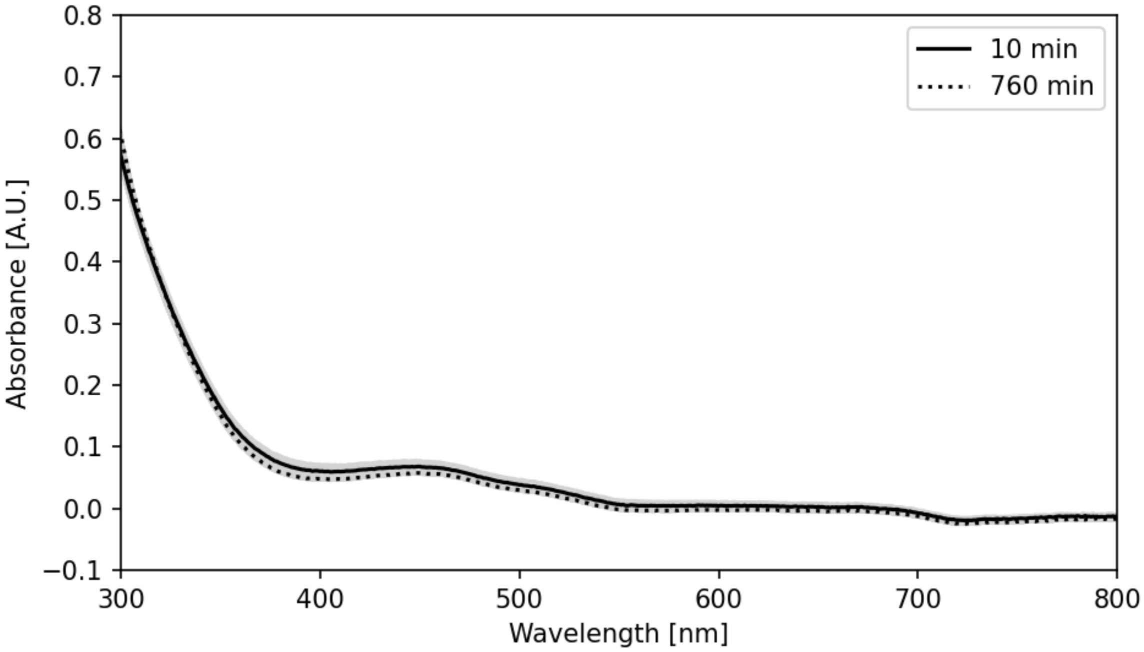

(*Z*)-1,2-Bis(2,6-dichlorophenyl)diazene (6, 50 µM, DMSO/PBS pH 7 8:2 with 10 mM GSH, green-adapted)

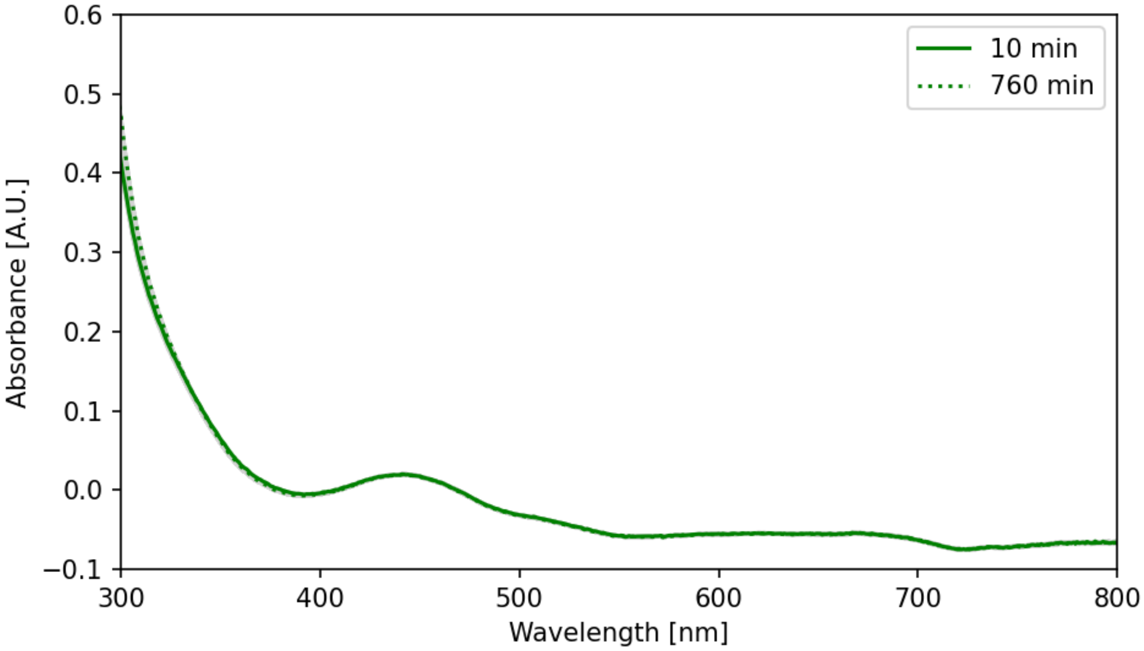

(*E*)-1,2-Bis(2,6-dimethoxyphenyl)diazene (9, 50 µM, DMSO/PBS pH 7 8:2 with 10 mM GSH, dark-adapted)

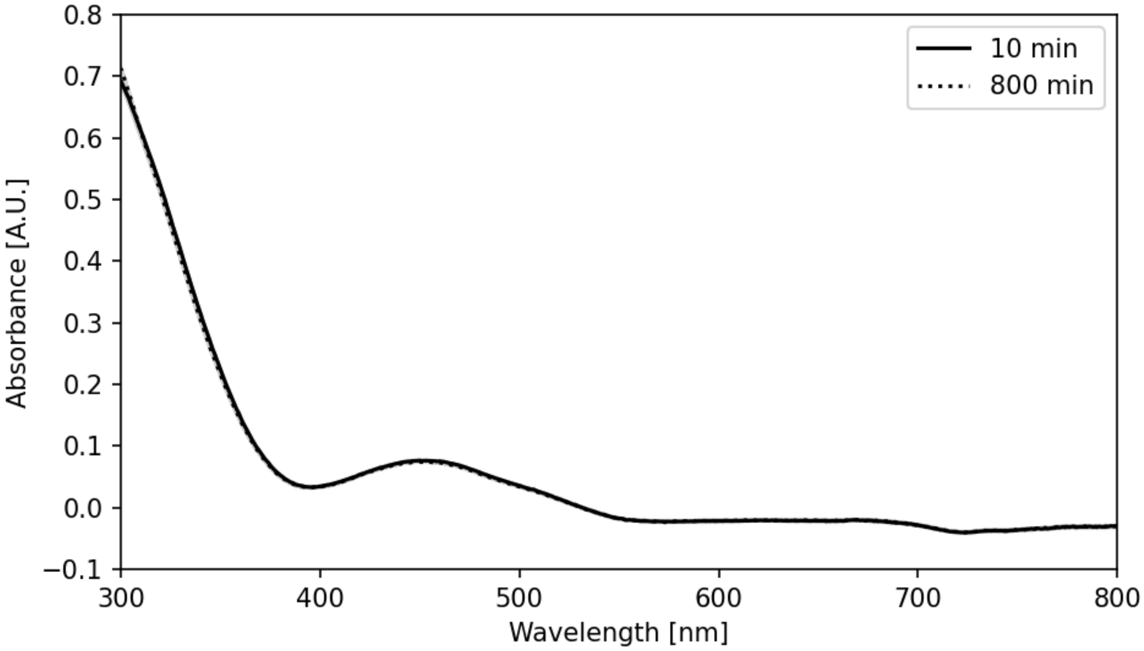

(*Z*)-1,2-Bis(2,6-dimethoxyphenyl)diazene (9, 50 µM, DMSO/PBS pH 7 8:2 with 10 mM GSH, green-adapted)

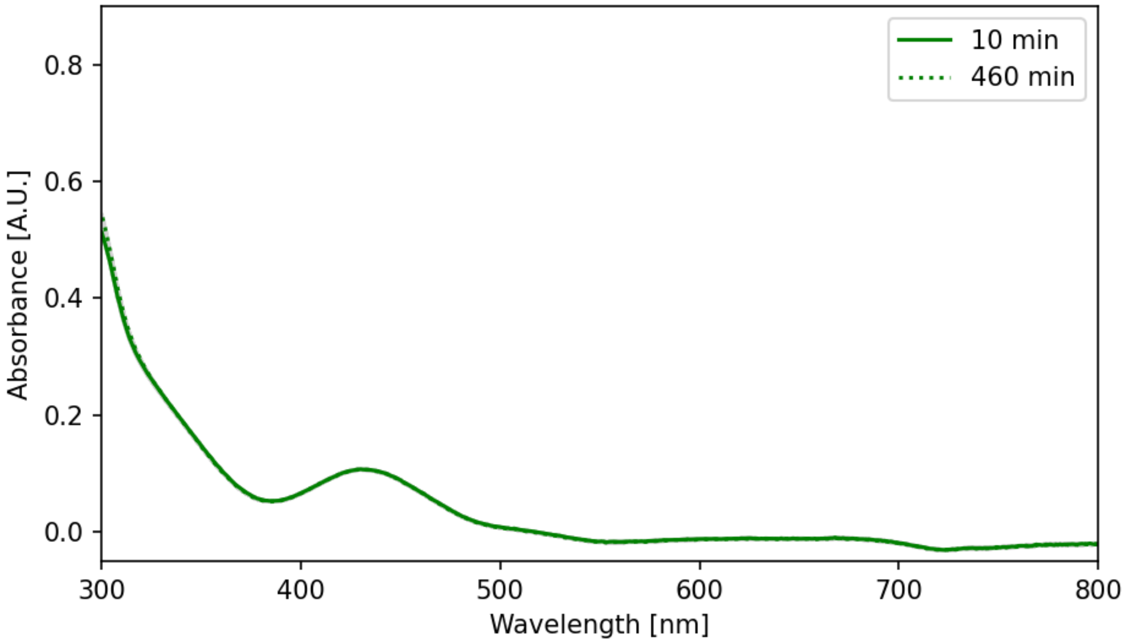

(*E*)-,2-Bis(3-chloro-1-fluoronaphthalen-2-yl)diazene (12, 50 µM, DMSO/PBS pH 7 8:2 with 10 mM GSH, dark-adapted)

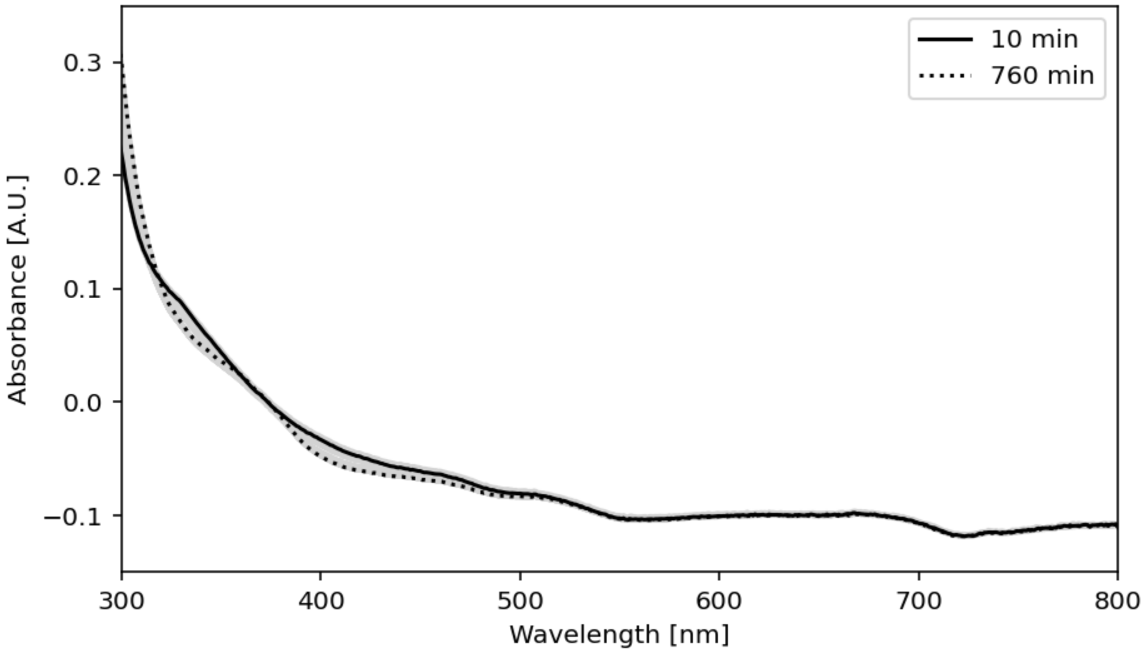

(*Z*)-1,2-Bis(3-chloro-1-fluoronaphthalen-2-yl)diazene (12, 50 µM, DMSO/PBS pH 7 8:2 with 10 mM GSH, red-adapted)

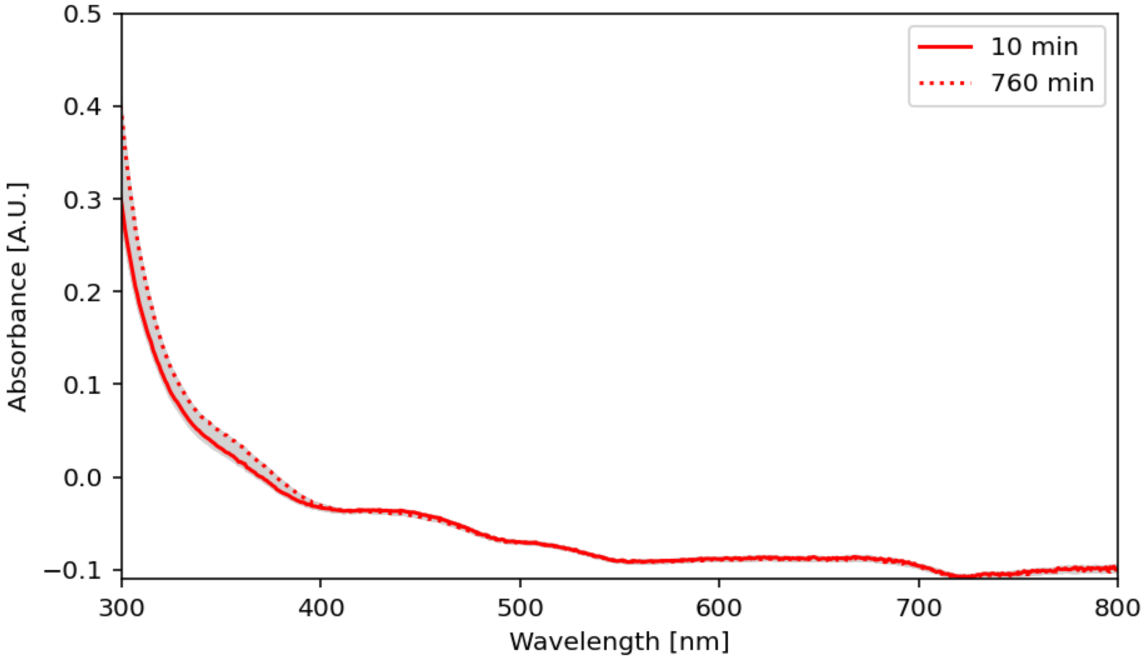

(*E*)-3-Chloro-4-((2-chloro-6-fluorophenyl)diazenyl)-5-fluorobenzoic acid (17, 50 µM, DMSO/PBS pH 7 8:2 with 10 mM GSH, dark-adapted)

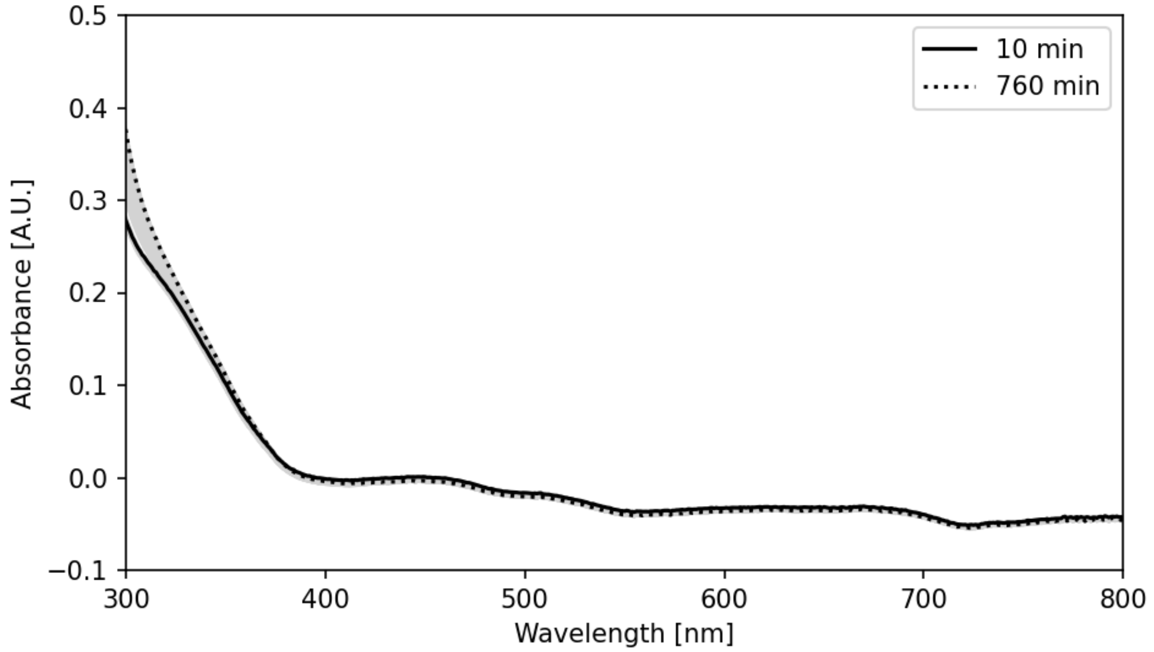

(*Z*)-3-Chloro-4-((2-chloro-6-fluorophenyl)diazenyl)-5-fluorobenzoic acid (17, 50 µM, DMSO/PBS pH 7 8:2 with 10 mM GSH, green-adapted)

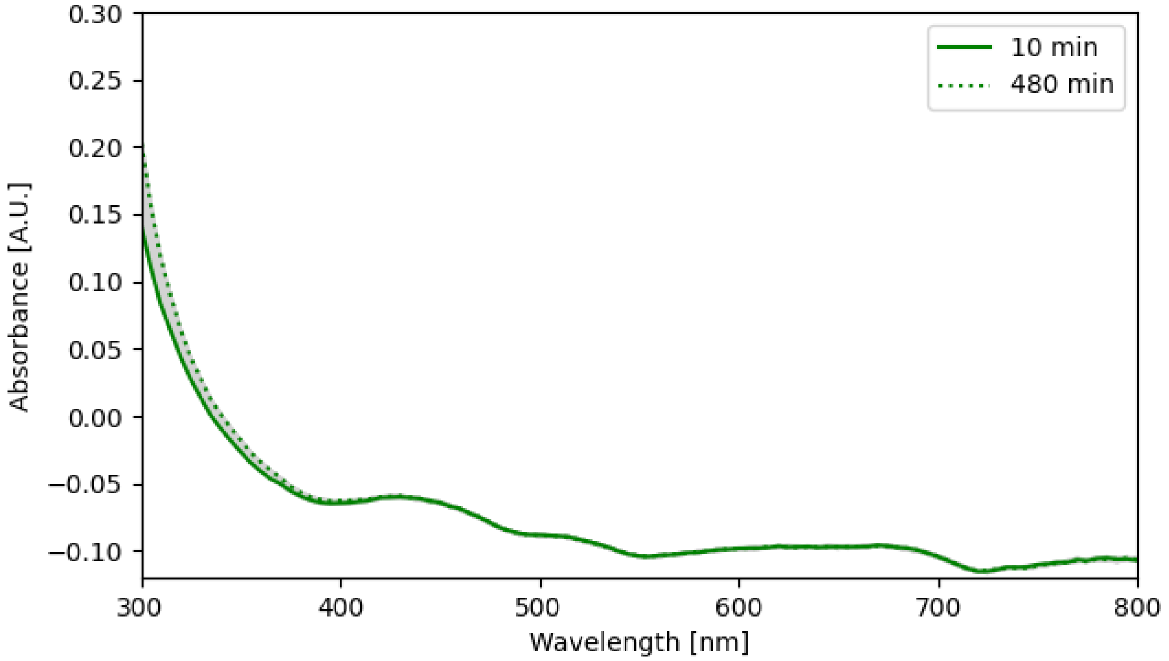

(*E*)-1-(2-Chloro-6-fluoro-4-nitrophenyl)-2-(2-chloro-6-fluorophenyl)diazene (19, 50 µM, DMSO/PBS pH 7 8:2 with 10 mM GSH, dark-adapted)

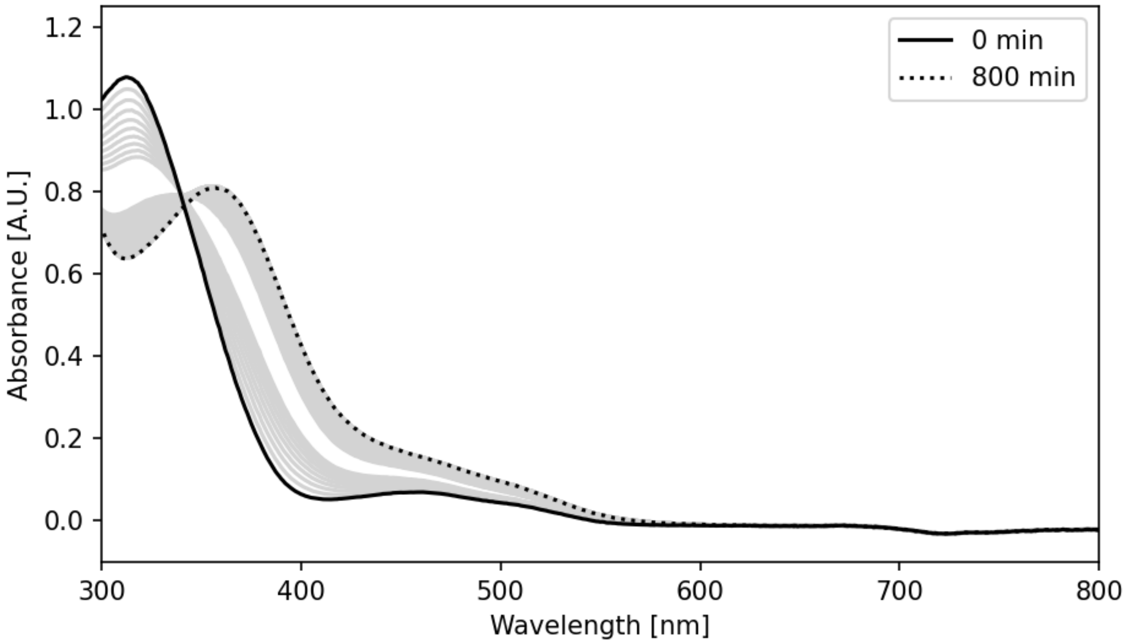

The stability of 1-(2-chloro-6-fluoro-4-nitrophenyl)-2-(2-chloro-6-fluorophenyl)diazene (dark-adapted) against GSH was evaluated through UV-vis-analysis with $A(t) = A \cdot e^{\frac{-\ln(2) \cdot t}{T_{1/2}}} + c$.

→ $t_{1/2}$ (**19**, 50 µM, DMSO/PBS pH 7 8:2 with 10 mM GSH, 37 °C) = 112 min.

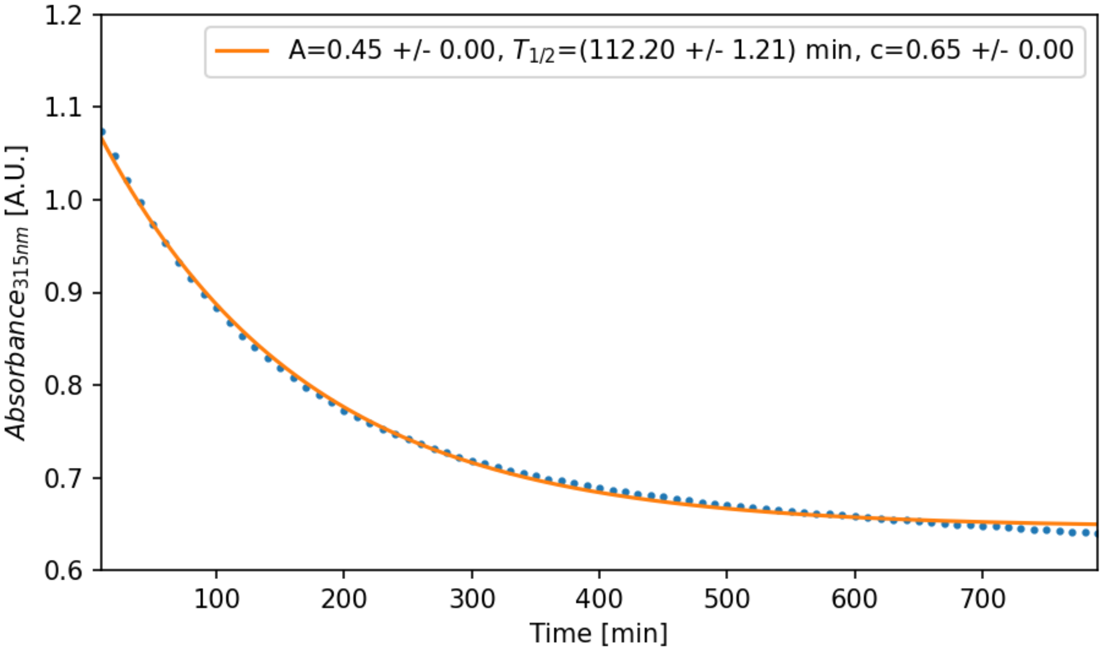

(Z)-1-(2-Chloro-6-fluoro-4-nitrophenyl)-2-(2-chloro-6-fluorophenyl)diazene (19, 50 µM, DMSO/PBS pH 7 8:2 with 10 mM GSH, green-adapted)

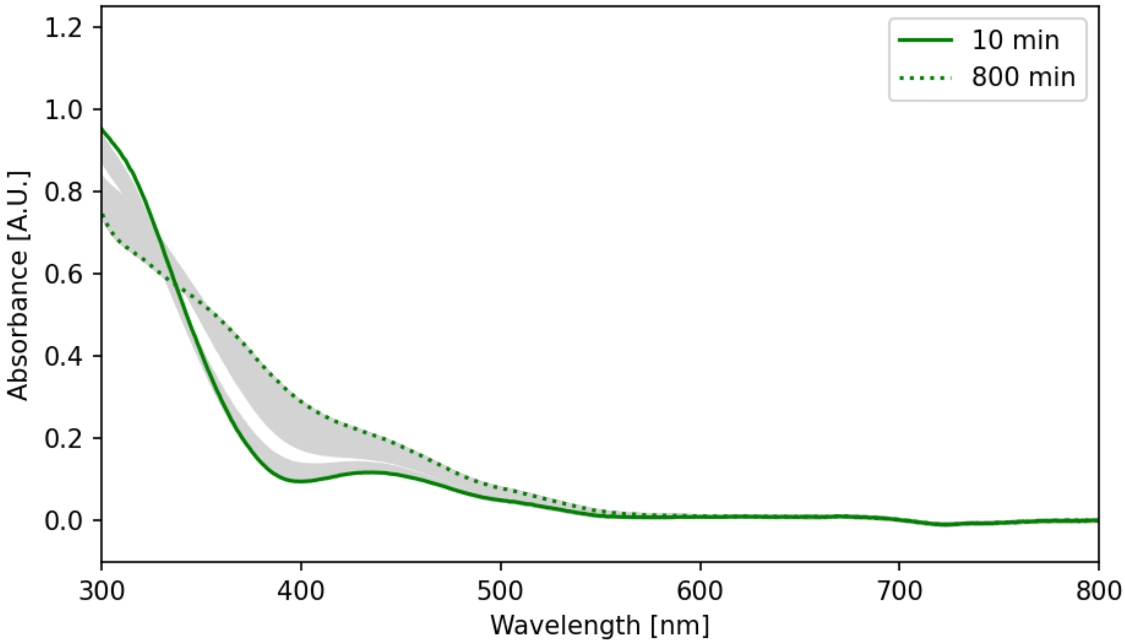

The stability of 1-(2-chloro-6-fluoro-4-nitrophenyl)-2-(2-chloro-6-fluorophenyl)diazene (green-adapted) against GSH was evaluated through UV-vis-analysis with $A(t) = A \cdot e^{\frac{-ln(2) \cdot t}{T_{1/2}}} + c$.

→ $t^a_{1/2}$ (**19**, 50 µM, DMSO/PBS pH 7 8:2 with 10 mM GSH, 37 °C) = 20.0 min.

→ $t^b_{1/2}$ (**19**, 50 µM, DMSO/PBS pH 7 8:2 with 10 mM GSH, 37 °C) = 160 min.

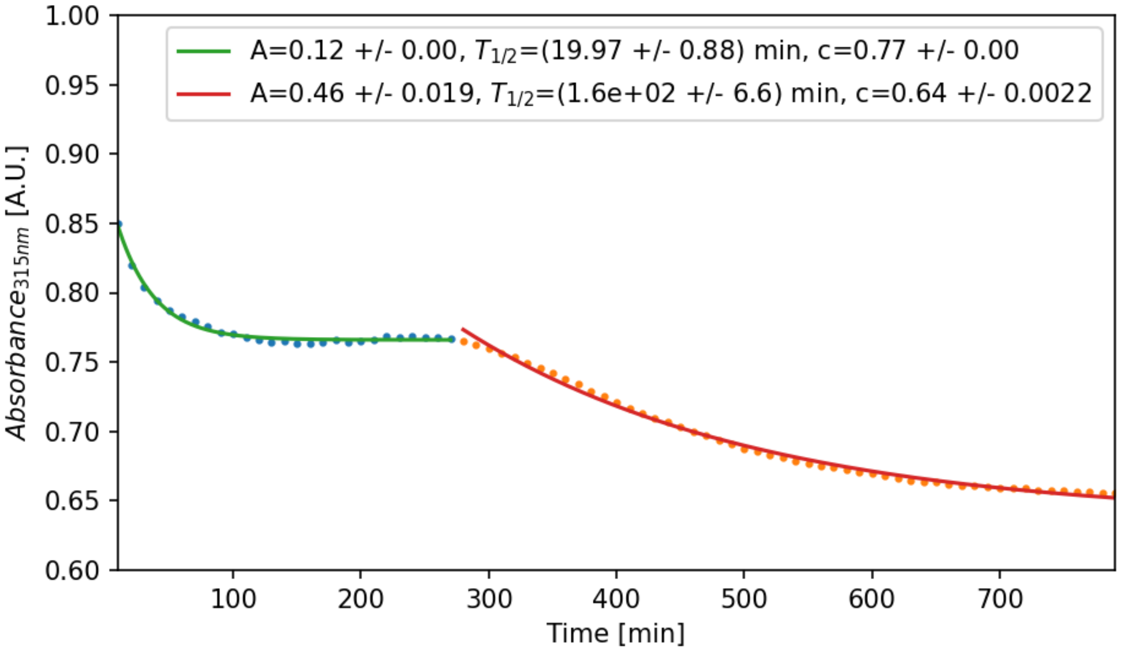

(*E*)-3-Chloro-4-((2-chloro-6-fluoro-4-nitrophenyl)diazenyl)-5-fluorobenzoic acid (20, 50 µM, DMSO/PBS pH 7 8:2 with 10 mM GSH, dark-adapted)

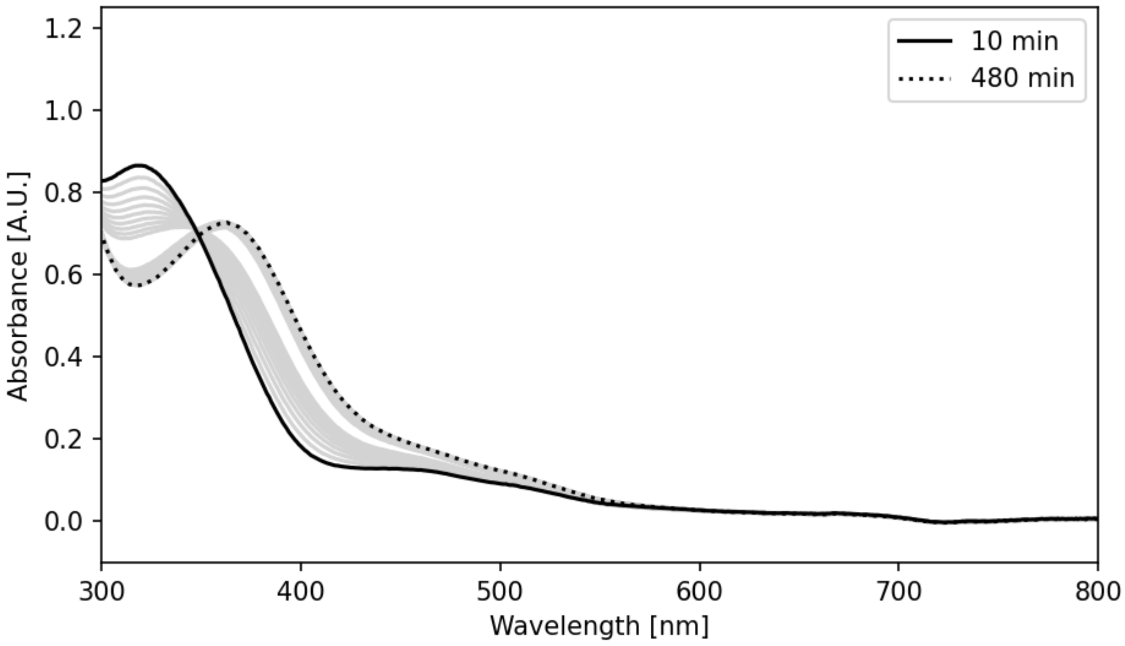

The stability of 3-chloro-4-((2-chloro-6-fluoro-4-nitrophenyl)diazenyl)-5-fluorobenzoic acid (dark-adapted) against GSH was evaluated through UV-vis-analysis with $A(t) = A \cdot e^{\frac{-\ln(2)\cdot t}{T_{1/2}}} + c$.

→ $t_{1/2}$ (**20**, 50 µM, DMSO/PBS pH 7 8:2 with 10 mM GSH, 37 °C) = 69.3 min.

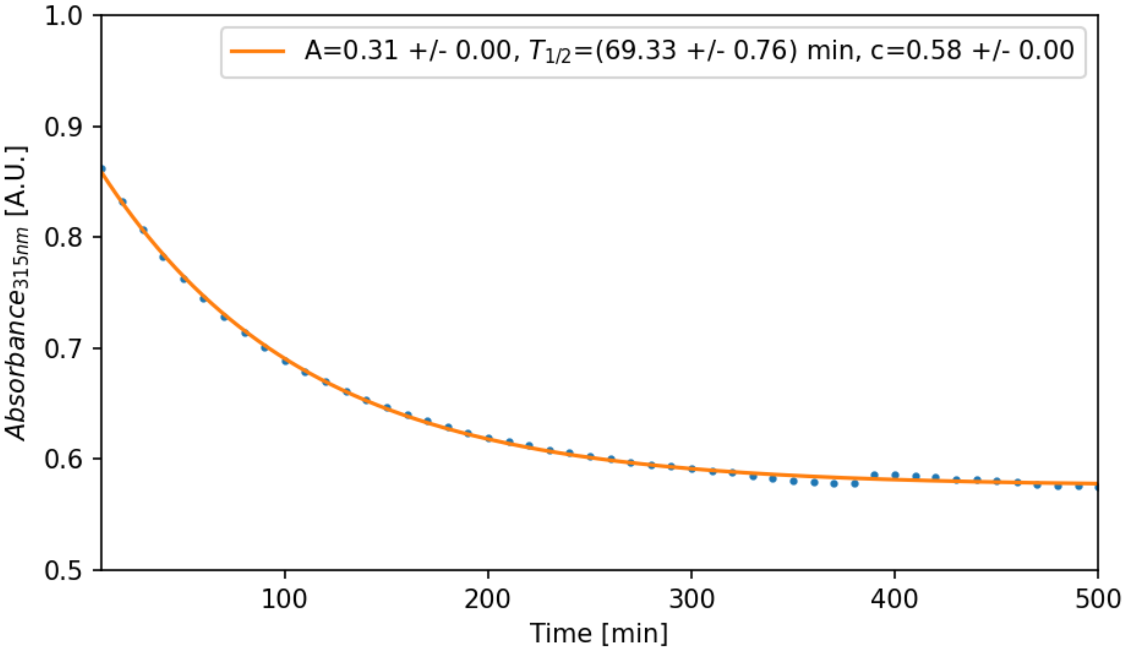

(*Z*)-3-Chloro-4-((2-chloro-6-fluoro-4-nitrophenyl)diazenyl)-5-fluorobenzoic acid (20, 50 µM, DMSO/PBS pH 7 8:2 with 10 mM GSH, green-adapted)

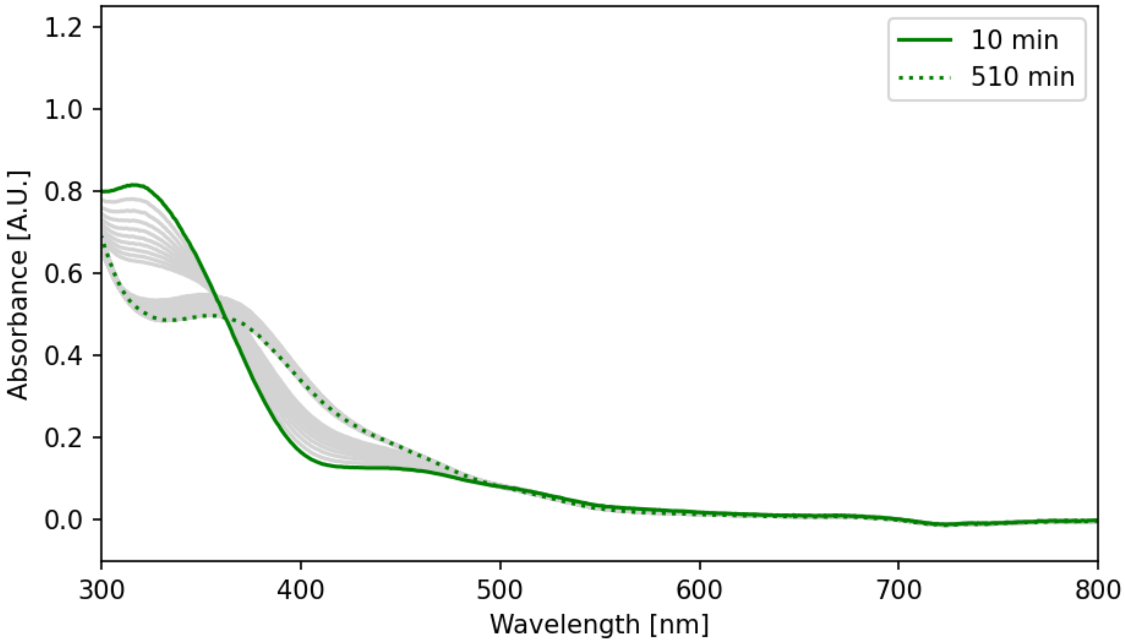

The stability of 3-chloro-4-((2-chloro-6-fluoro-4-nitrophenyl)diazenyl)-5-fluorobenzoic acid (green-adapted) against GSH was evaluated through UV-vis-analysis with $A(t) = A \cdot e^{\frac{-ln(2) \cdot t}{T_{1/2}}} + c$.

→ $t_{1/2}$ (**20**, 50 µM, DMSO/PBS pH 7 8:2 with 10 mM GSH, 37 °C) = 68.9 min.

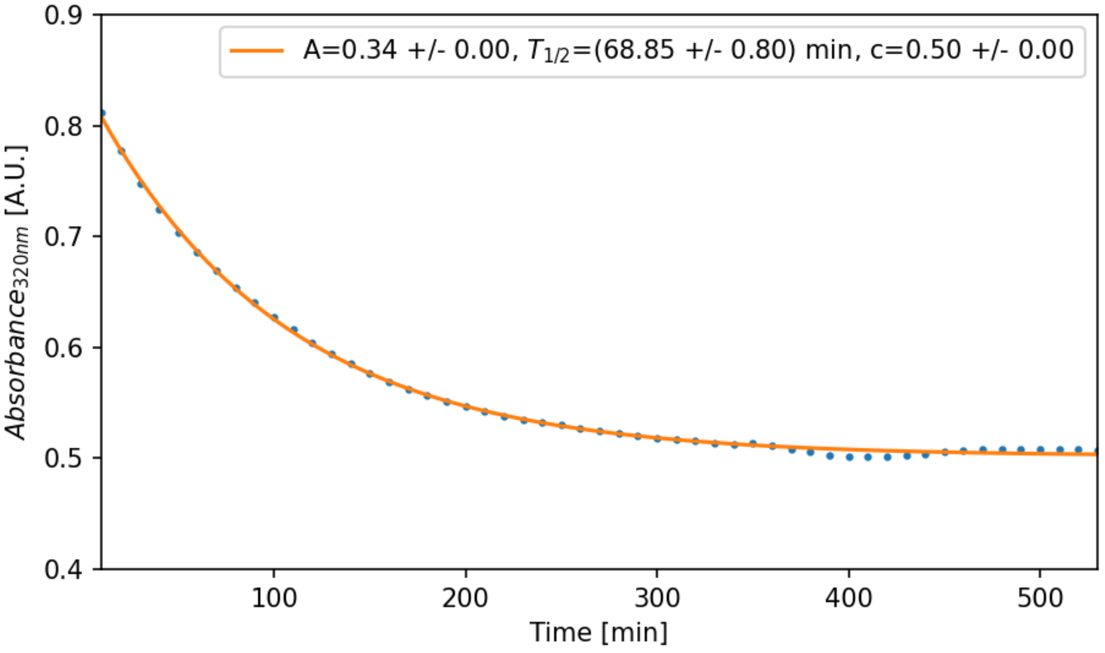

(*E*)-Methyl-3-chloro-4-((2-chloro-6-fluorophenyl)diazenyl)-5-fluorobenzoate (21, 50 µM, DMSO/PBS pH 7 8:2 with 10 mM GSH, dark-adapted)

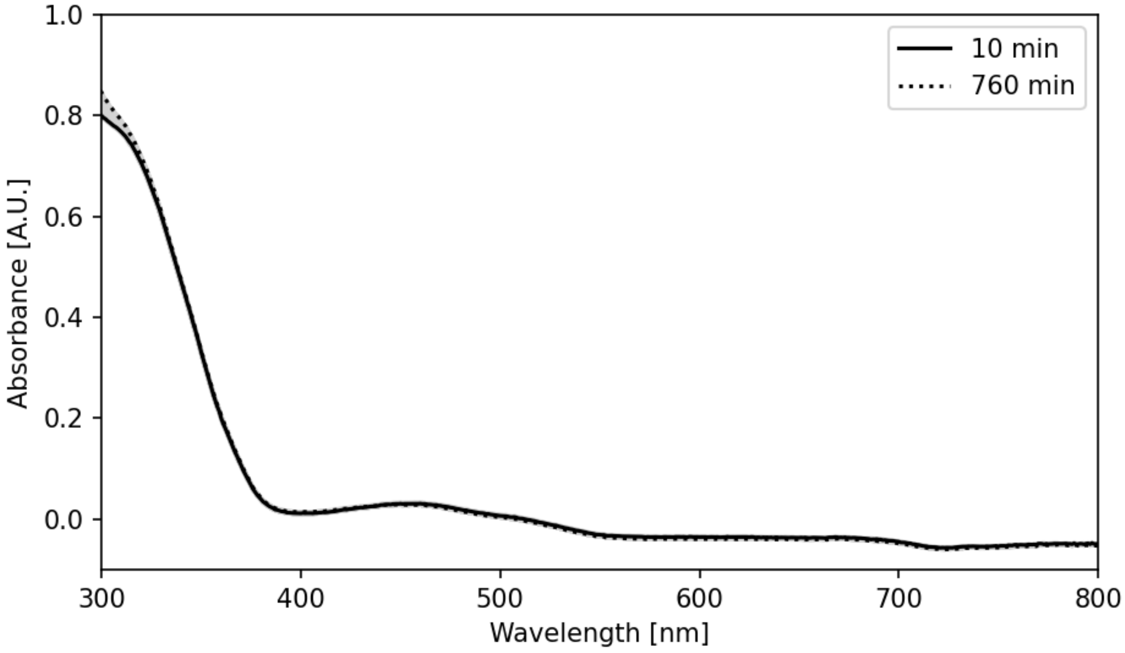

(*Z*)-Methyl-3-chloro-4-((2-chloro-6-fluorophenyl)diazenyl)-5-fluorobenzoate (21, 50 µM, DMSO/PBS pH 7 8:2 with 10 mM GSH, green-adapted)

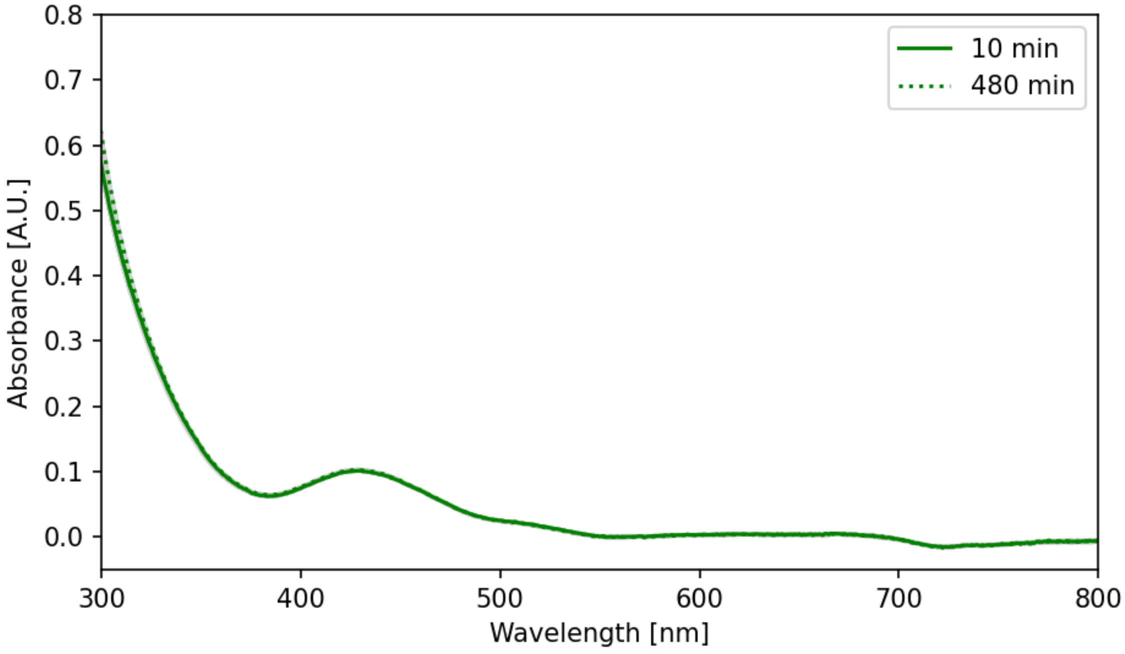

(*E*)-Methyl-3-bromo-4-((2-bromo-6-fluorophenyl)diazenyl)-5-fluorobenzoate (22, 50 µM, DMSO/PBS pH 7 8:2 with 10 mM GSH, dark-adapted)

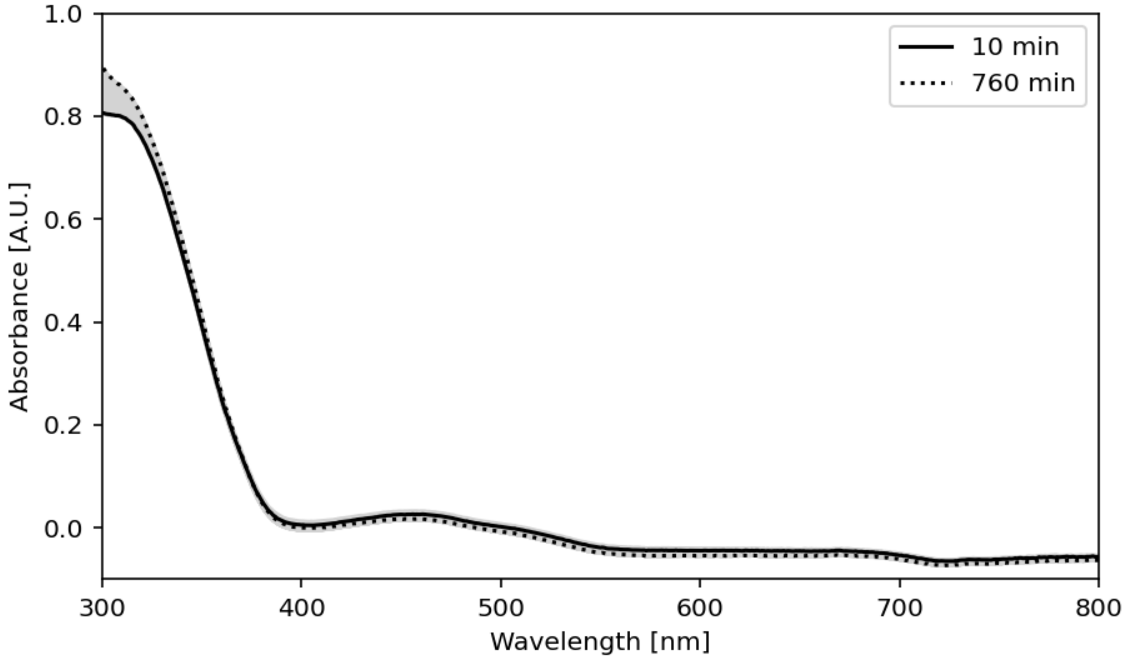

(*Z*)-Methyl-3-bromo-4-((2-bromo-6-fluorophenyl)diazenyl)-5-fluorobenzoate (22, 50 µM, DMSO/PBS pH 7 8:2 with 10 mM GSH, green-adapted)

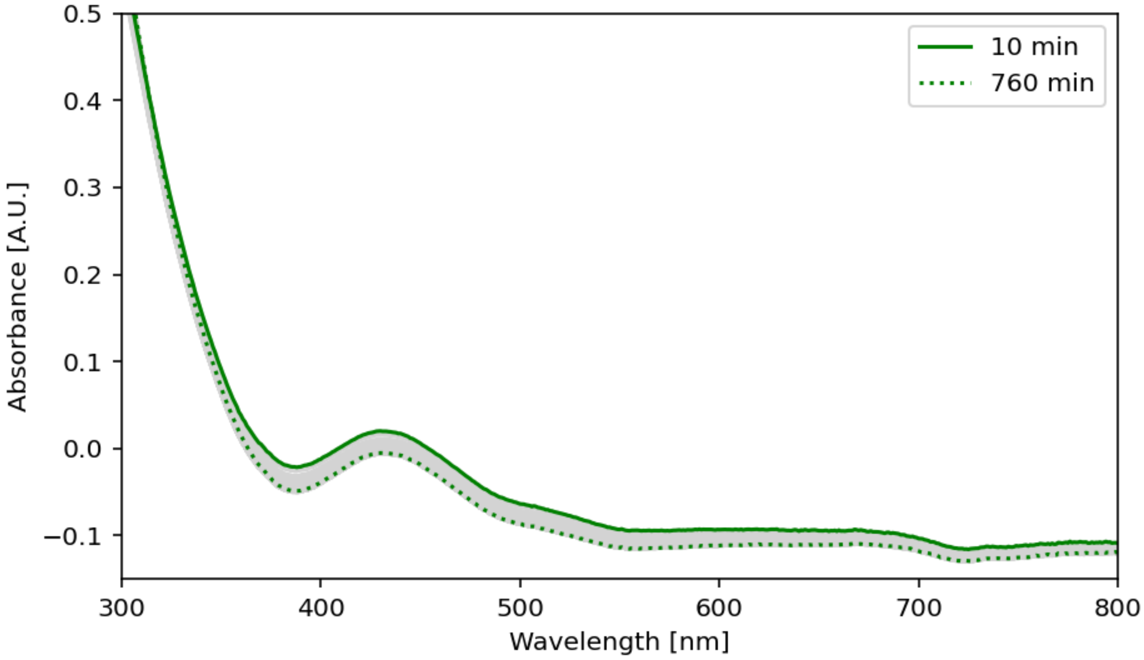

(*E*)-Methyl-3,5-dichloro-4-((2,6-dichlorophenyl)diazenyl)benzoate (23, 50 µM, DMSO/PBS pH 7 8:2 with 10 mM GSH, dark-adapted)

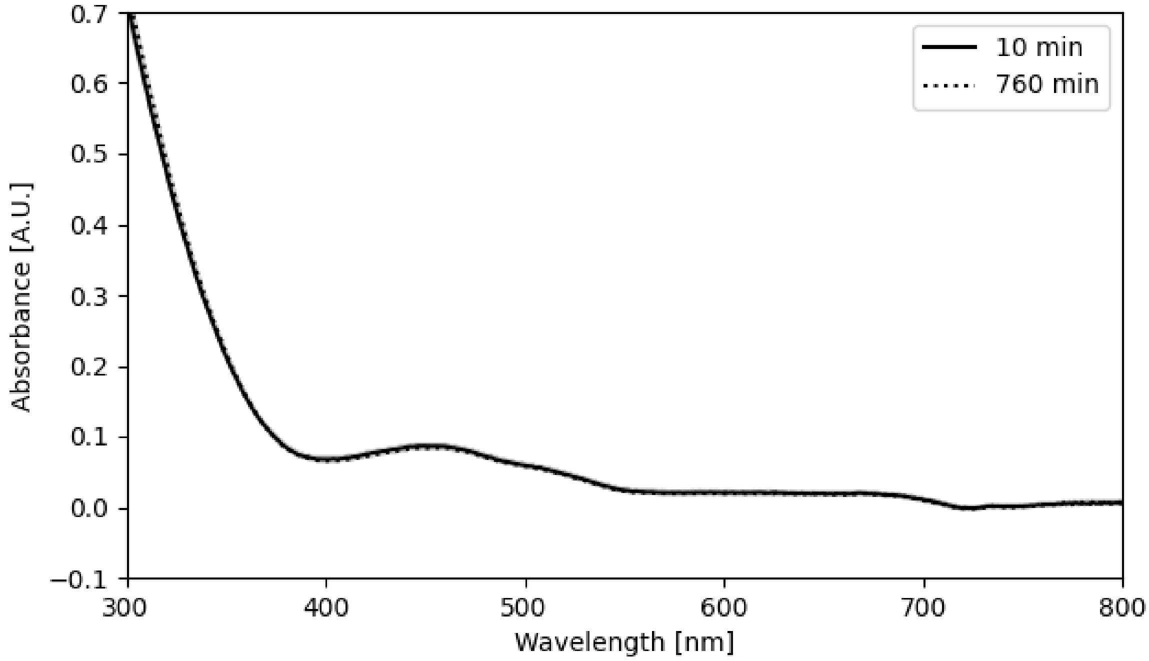

(*Z*)-Methyl-3,5-dichloro-4-((2,6-dichlorophenyl)diazenyl)benzoate (23, 50 µM, DMSO/PBS pH 7 8:2 with 10 mM GSH, red-adapted)

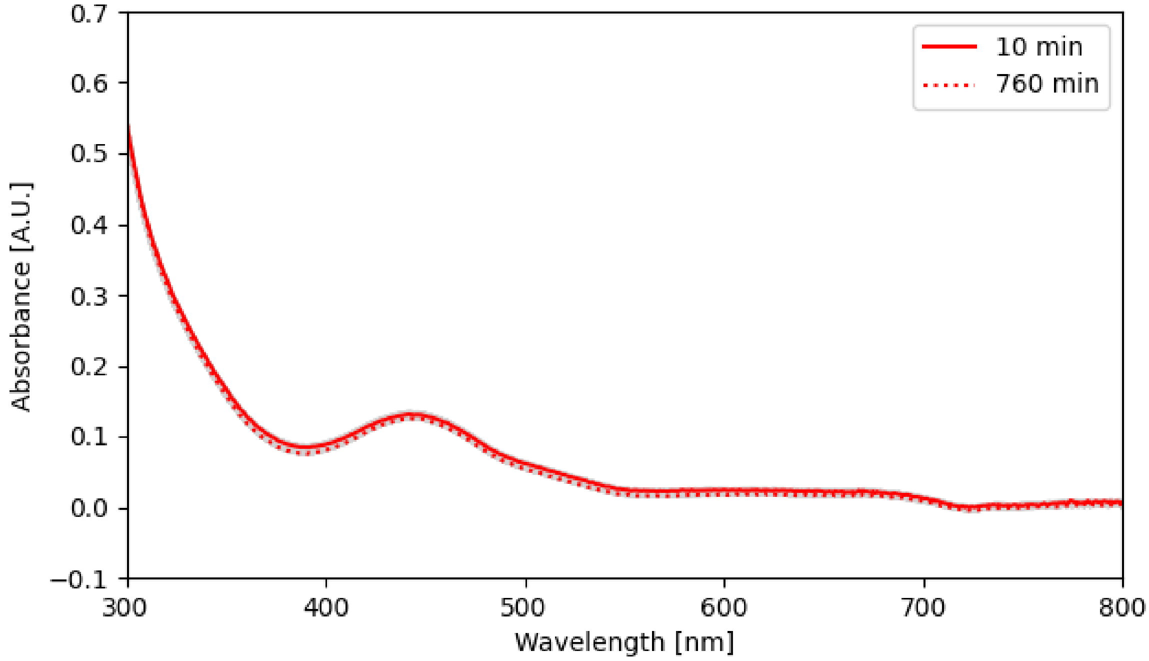

(*E*)-3-Chloro-4-((2-chloro-6-fluorophenyl)diazenyl)-5-fluoro-N-methylbenzamide (24, 50 µM, DMSO/PBS pH 7 8:2 with 10 mM GSH, dark-adapted)

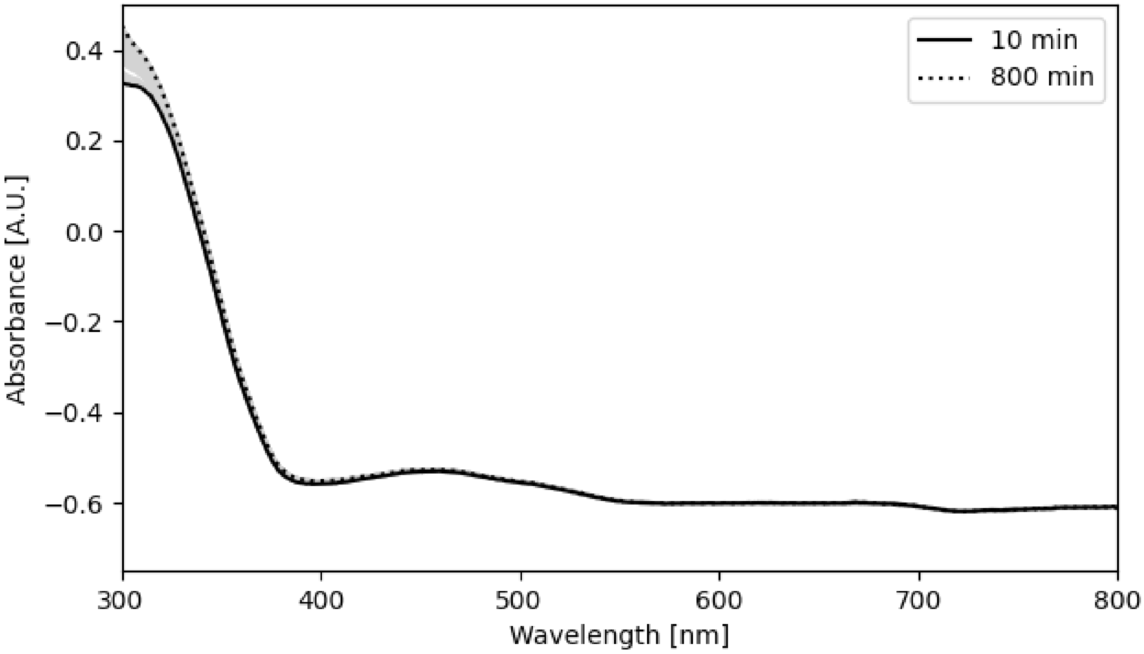

(*Z*)-3-Chloro-4-((2-chloro-6-fluorophenyl)diazenyl)-5-fluoro-N-methylbenzamide (24, 50 µM, DMSO/PBS pH 7 8:2 with 10 mM GSH, dark-adapted)

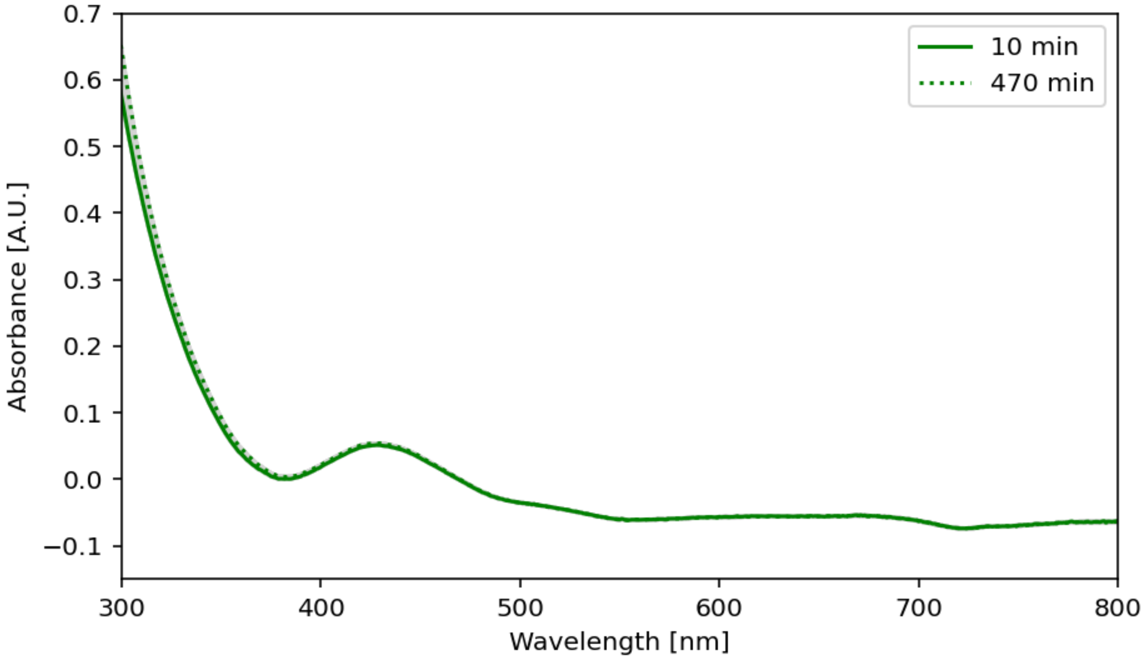

(*E*)-Dimethyl 4,4'-(diazene-1,2-diyl)(E)-bis(3-chloro-5-fluorobenzoate) (28, 50 μM, DMSO/PBS pH 7 8:2 with 10 mM GSH, dark-adapted)

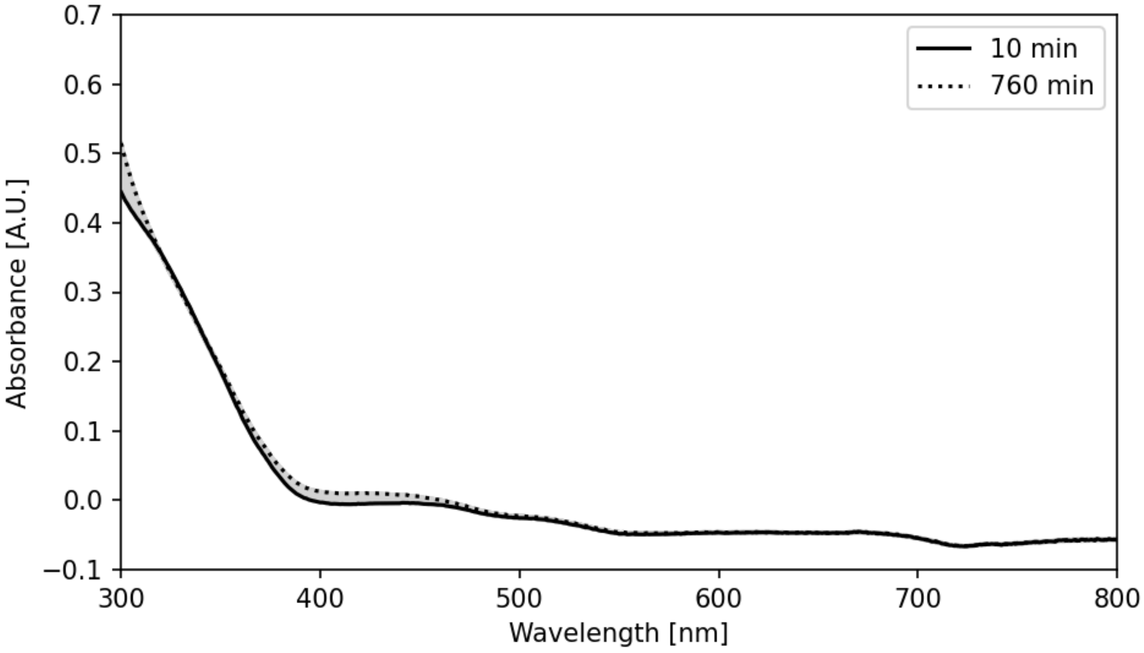

(*Z*)-Dimethyl 4,4'-(diazene-1,2-diyl)(E)-bis(3-chloro-5-fluorobenzoate) (28, 50 μM, DMSO/PBS pH 7 8:2 with 10 mM GSH, green-adapted)

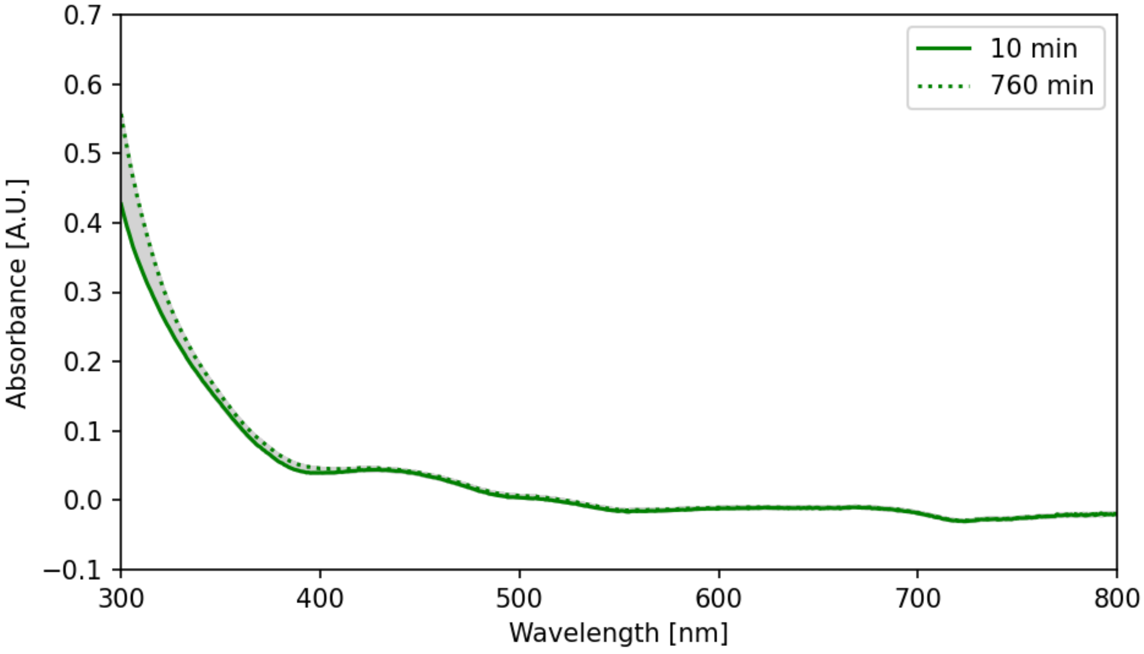

(*E*)-3-Chloro-4-((2-chloro-6-fluorophenyl)diazenyl)-5-fluoroaniline (38, 50 μM, DMSO/PBS pH 7 8:2 with 10 mM GSH, dark-adapted)

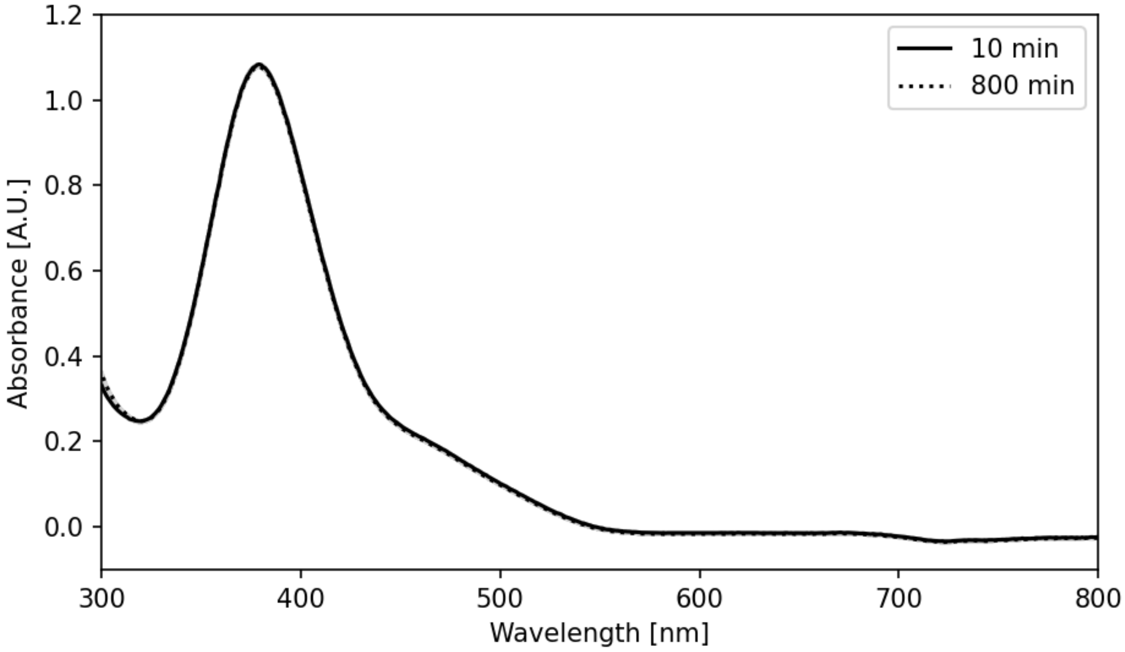

(*Z*)-3-Chloro-4-((2-chloro-6-fluorophenyl)diazenyl)-5-fluoroaniline (38, 50 μM, DMSO/PBS pH 7 8:2 with 10 mM GSH, green-adapted)

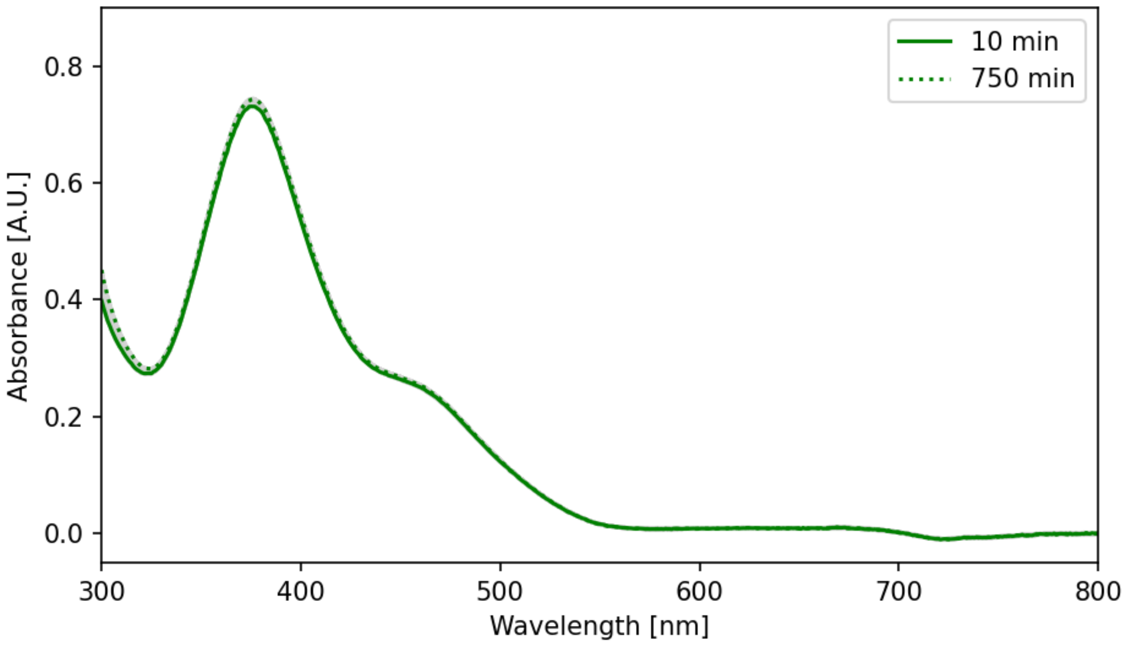

(*E*)-*N*-(3-chloro-4-((2-chloro-6-fluorophenyl)diazenyl)-5-fluorophenyl)acetamid (39, 50 µM, DMSO/PBS pH 7 8:2 with 10 mM GSH, dark-adapted)

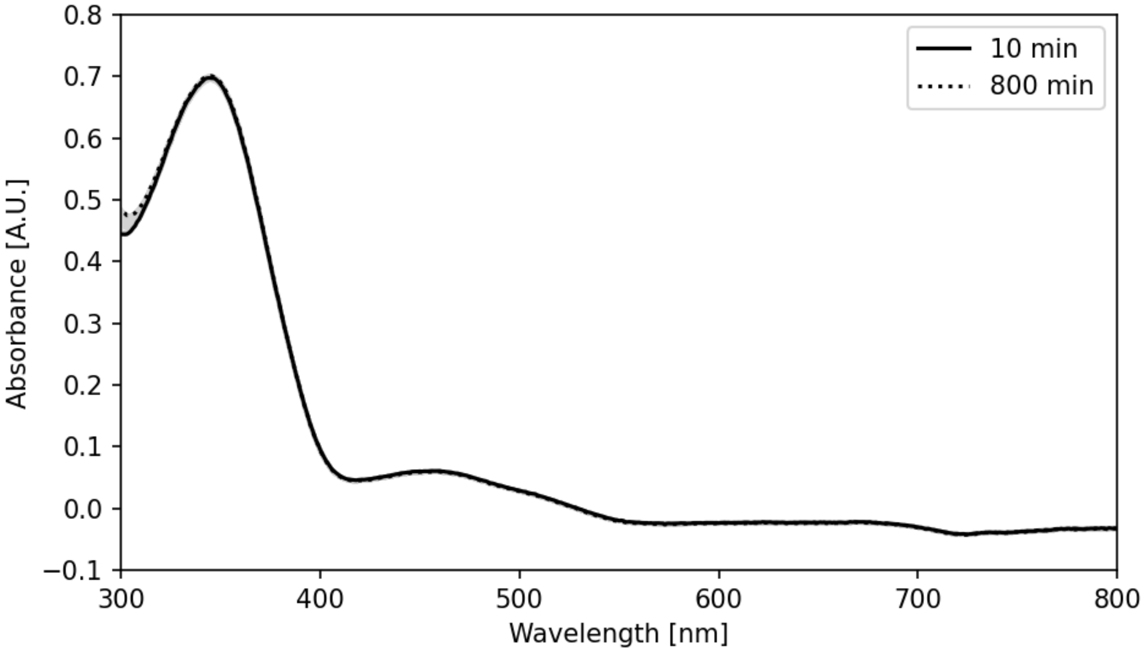

(*Z*)-*N*-(3-chloro-4-((2-chloro-6-fluorophenyl)diazenyl)-5-fluorophenyl)acetamid (39, 50 µM, DMSO/PBS pH 7 8:2 with 10 mM GSH, green-adapted)

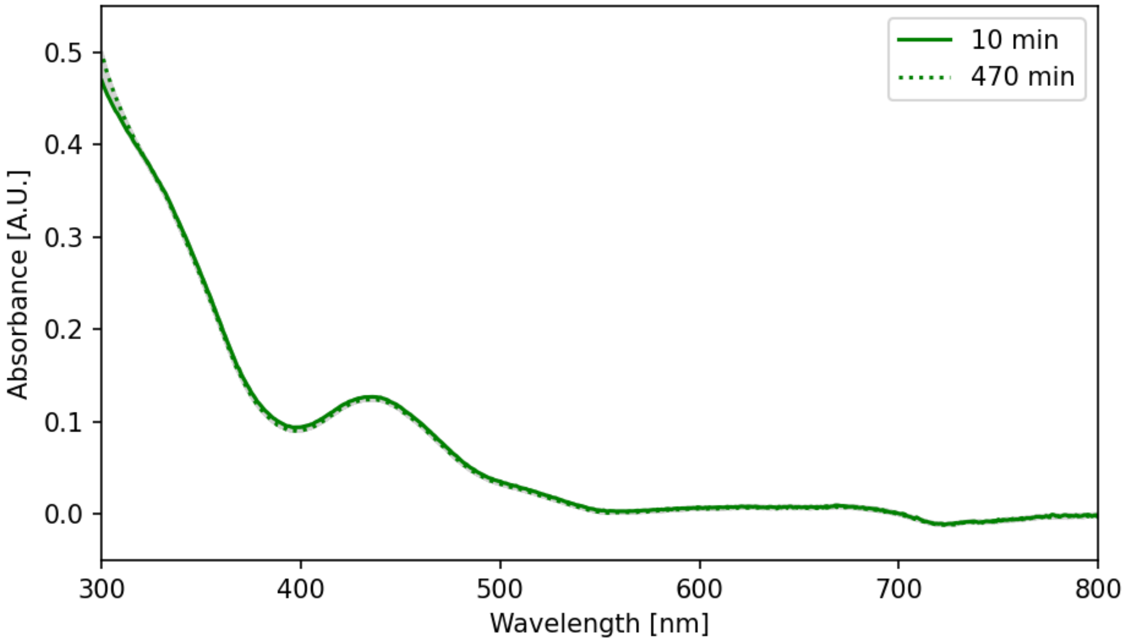

(*E*)-Methyl-3-chloro-4-((2-chloro-6-fluoro-4-methoxyphenyl)diazenyl)-5-fluorobenzoate (46, 50 µM, DMSO/PBS pH 7 8:2 with 10 mM GSH, dark-adapted)

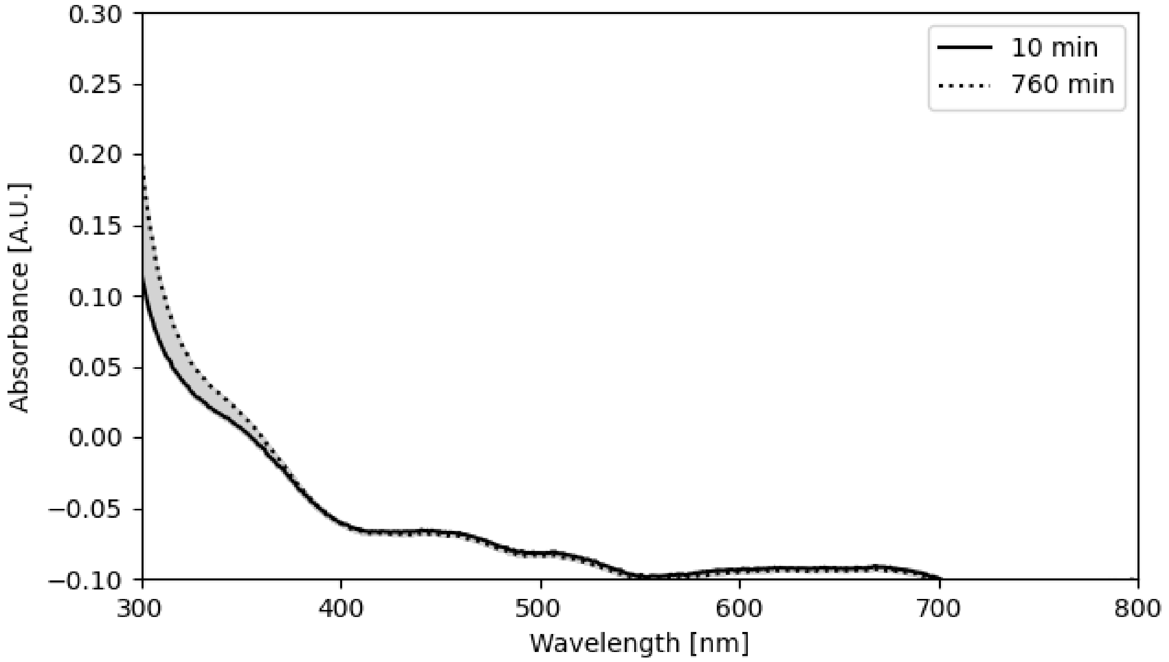

(*Z*)-Methyl-3-chloro-4-((2-chloro-6-fluoro-4-methoxyphenyl)diazenyl)-5-fluorobenzoate (46, 50 µM, DMSO/PBS pH 7 8:2 with 10 mM GSH, red-adapted)

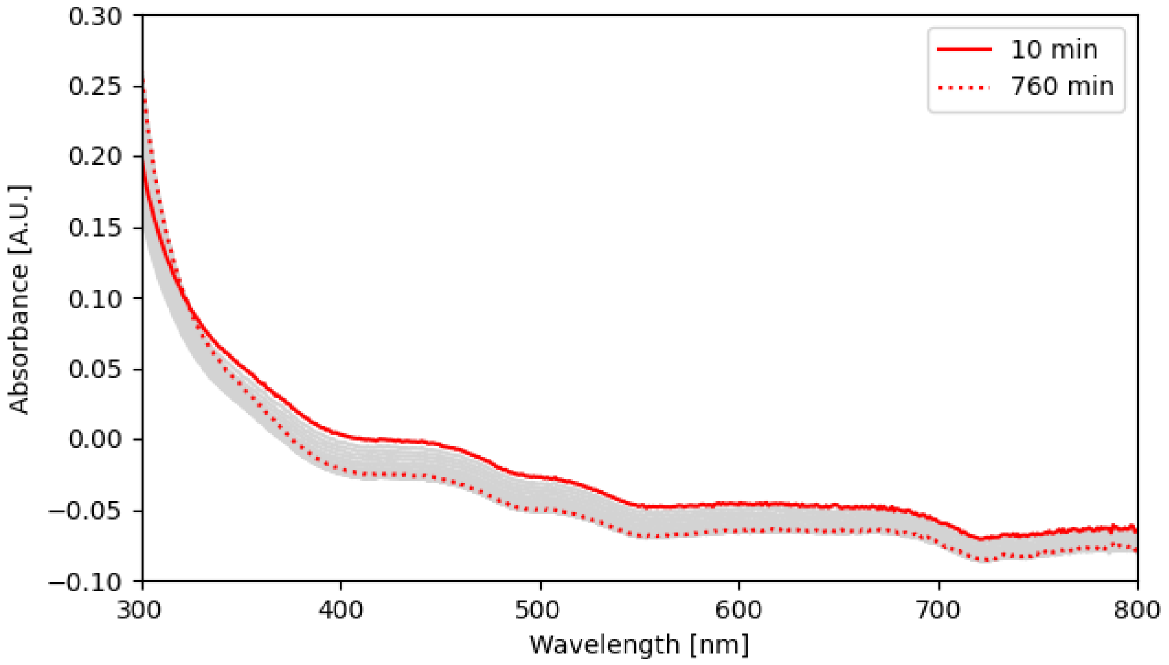

(*E*)-4-((4-Amino-2,6-dichlorophenyl)diazenyl)-3,5-dichlorobenzenesulfonamide (48, 50 µM, DMSO/PBS pH 7 8:2 with 10 mM GSH, dark-adapted)

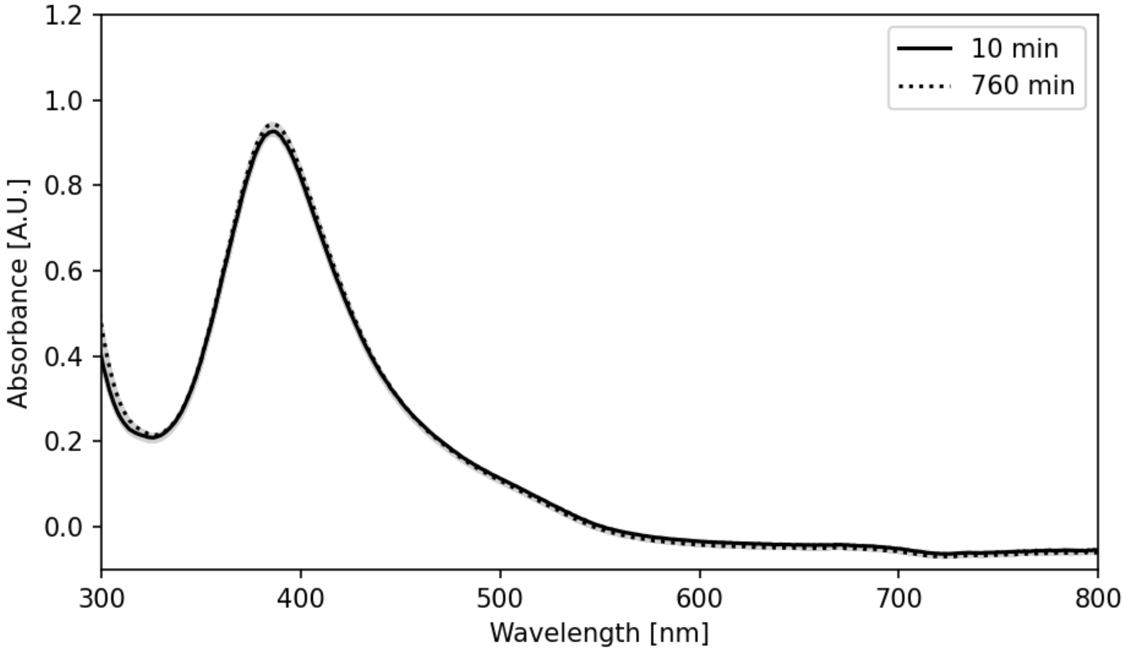

(*Z*)-4-((4-Amino-2,6-dichlorophenyl)diazenyl)-3,5-dichlorobenzenesulfonamide (48, 50 µM, DMSO/PBS pH 7 8:2 with 10 mM GSH, red-adapted)

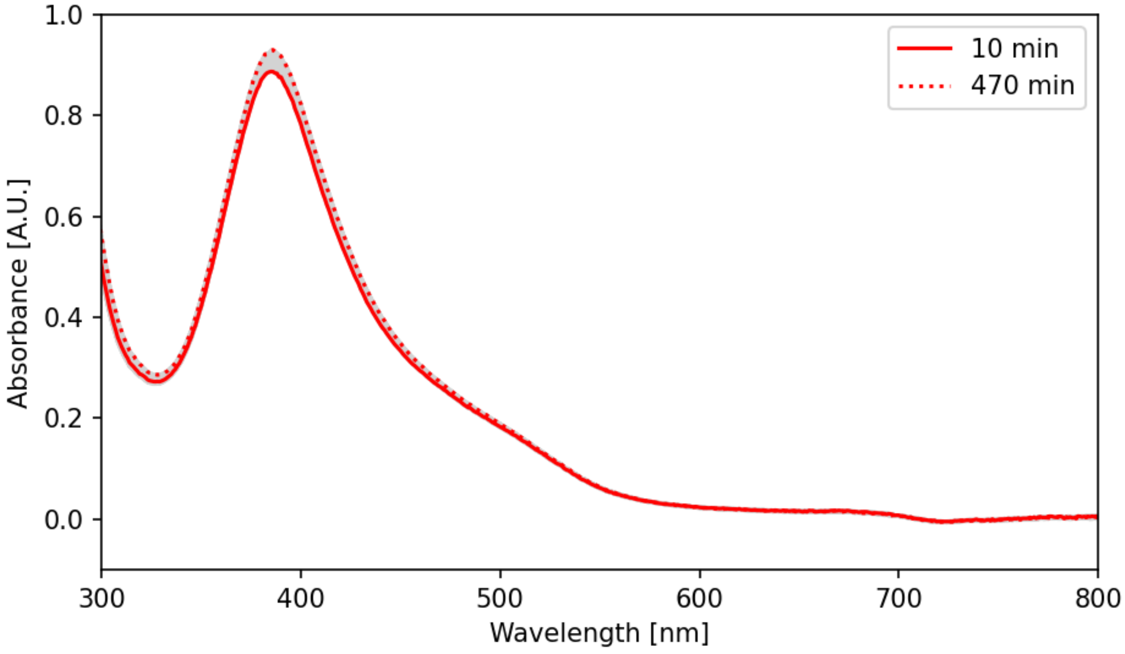

(*E*)-3-Chloro-4-((2-chloro-6-fluoro-4-hydroxyphenyl)diazenyl)-5-fluorobenzoic acid (49, 50 µM, DMSO/PBS pH 7 8:2 with 10 mM GSH, dark-adapted)

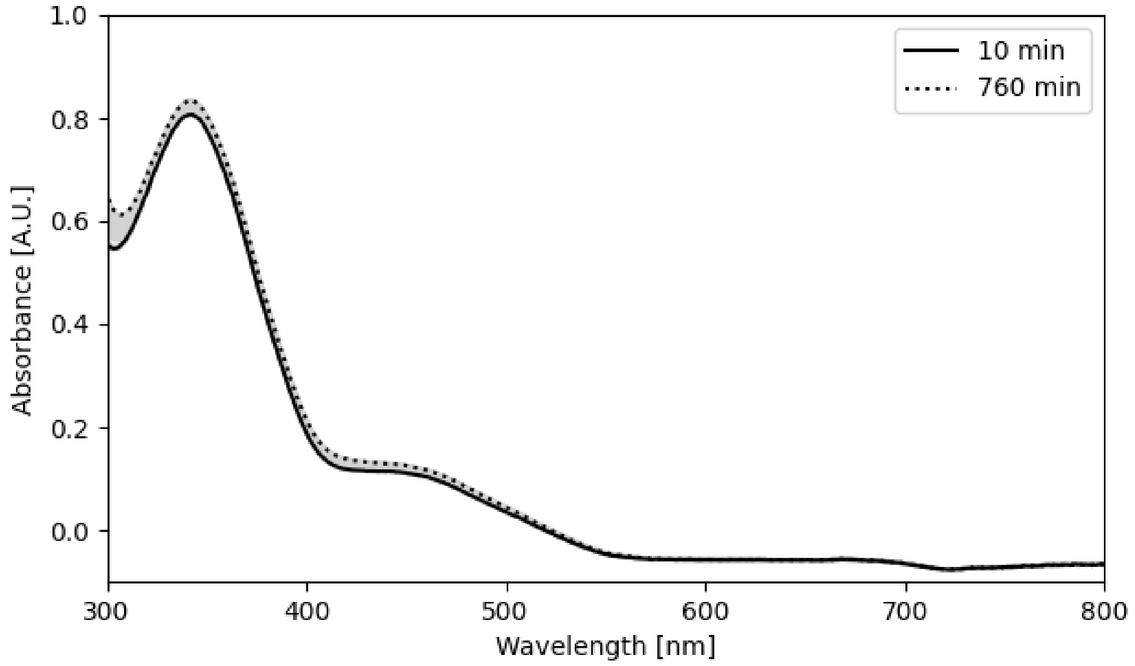

(*Z*)-3-Chloro-4-((2-chloro-6-fluoro-4-hydroxyphenyl)diazenyl)-5-fluorobenzoic acid (49, 50 µM, DMSO/PBS pH 7 8:2 with 10 mM GSH, blue-adapted)

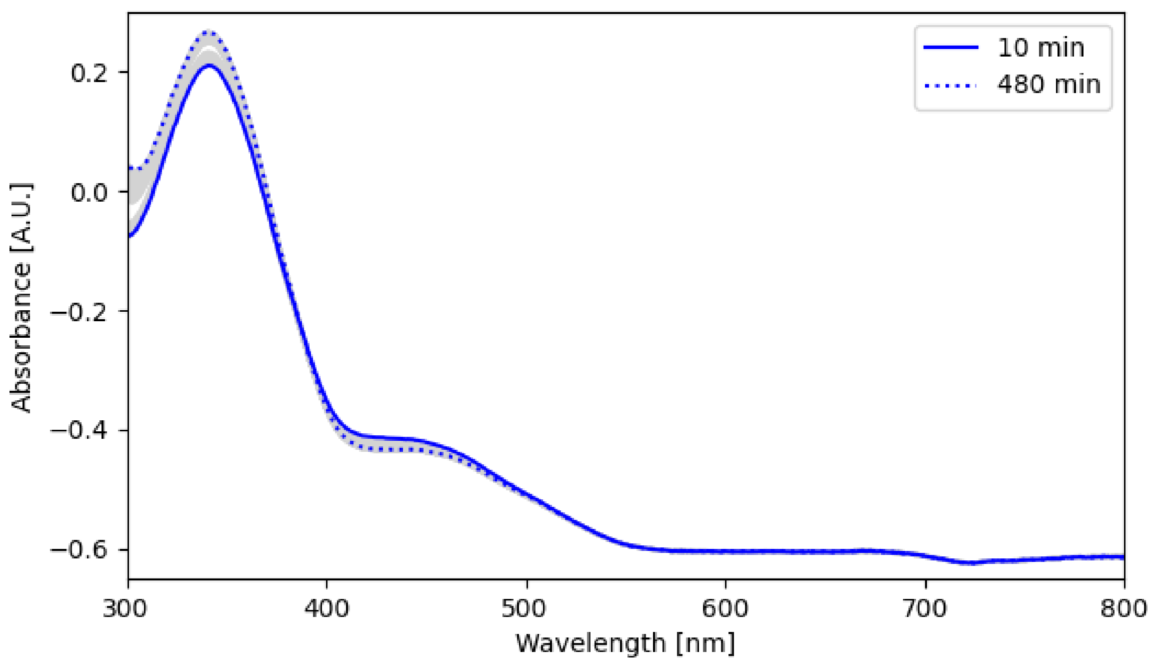

5. Thermal Relaxation

The azobenzenes were irradiated for 15 min to their *cis*-form followed by relaxation in the dark at a temperature between rt and 90 °C. The PSS were determined either through UV-vis analysis or by ^{1}H or ^{19}F NMR analysis applying equation $A(t) = A \cdot e^{\frac{-ln(2) \cdot t}{T_{1/2}}} + c$.

The corresponding variations are indicated at the individual experiments.

(*E*/*Z*)-Azobenzene (1)

Irradiation with 365 nm

$t_{1/2}$ (**1**, UV-vis at 440 nm, 500 µM, DMSO-d_6/D_2O 9:1, 55 °C) = 3.16 h.

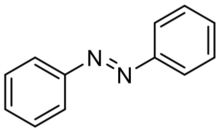

1
(440 nm, DMSO-d_6/H_2O 9:1, 55 °C)

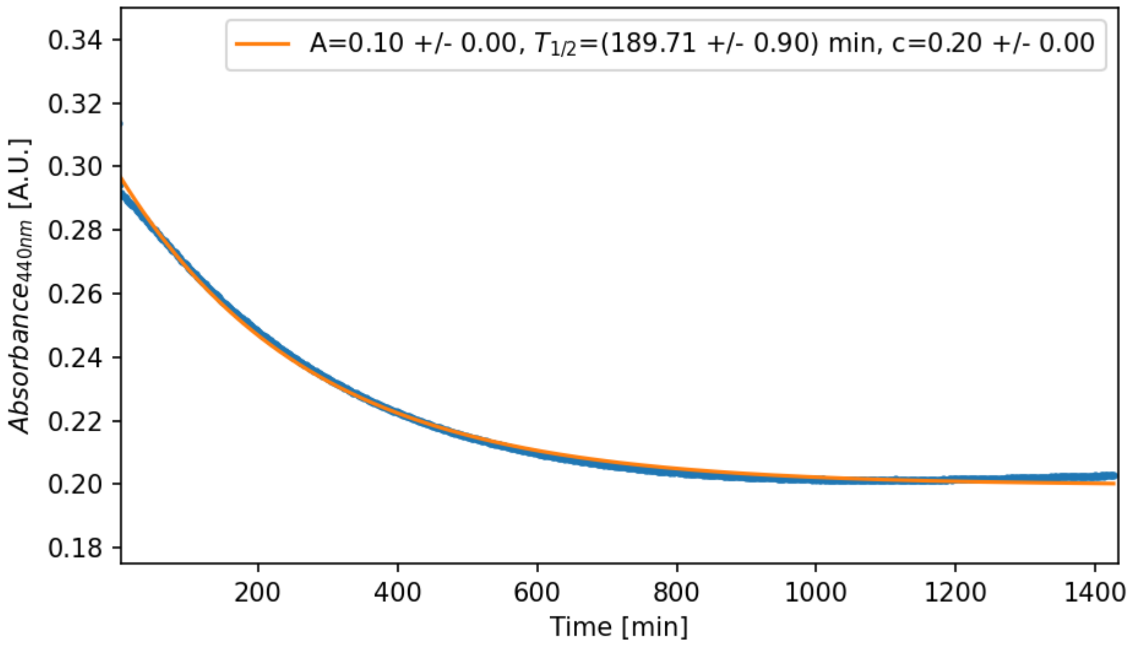

(*E*/*Z*)-1,2-Bis(2,6-difluorophenyl)diazene (2)

Irradiation with 525 nm

$t_{1/2}$ (**2**, ^{19}F NMR, 500 µM, DMSO-d_6/D_2O 9:1, 90 °C) = 3.97 h.

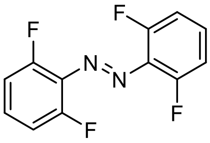

2
^{19}F NMR (471 MHz, DMSO-d_6/D_2O 9:1, 90 °C)

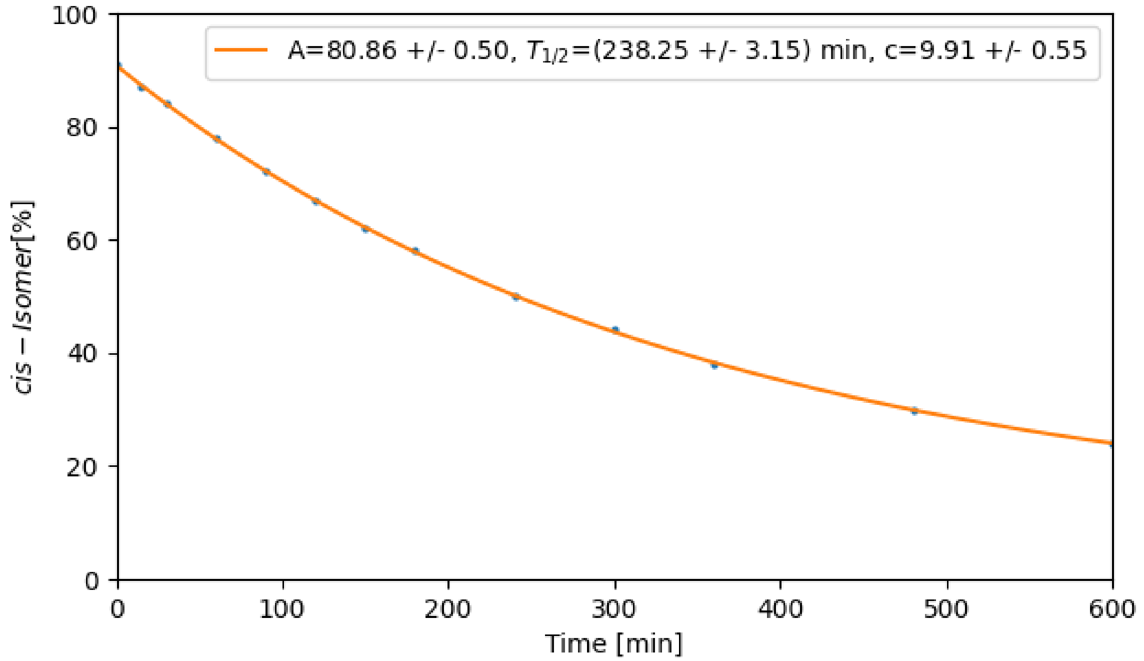
A=80.86 +/- 0.50, $T_{1/2}$=(238.25 +/- 3.15) min, c=9.91 +/- 0.55
cis – Isomer[%]
Time [min]
0
20
40
60
80
100
0
100
200
300
400
500
600

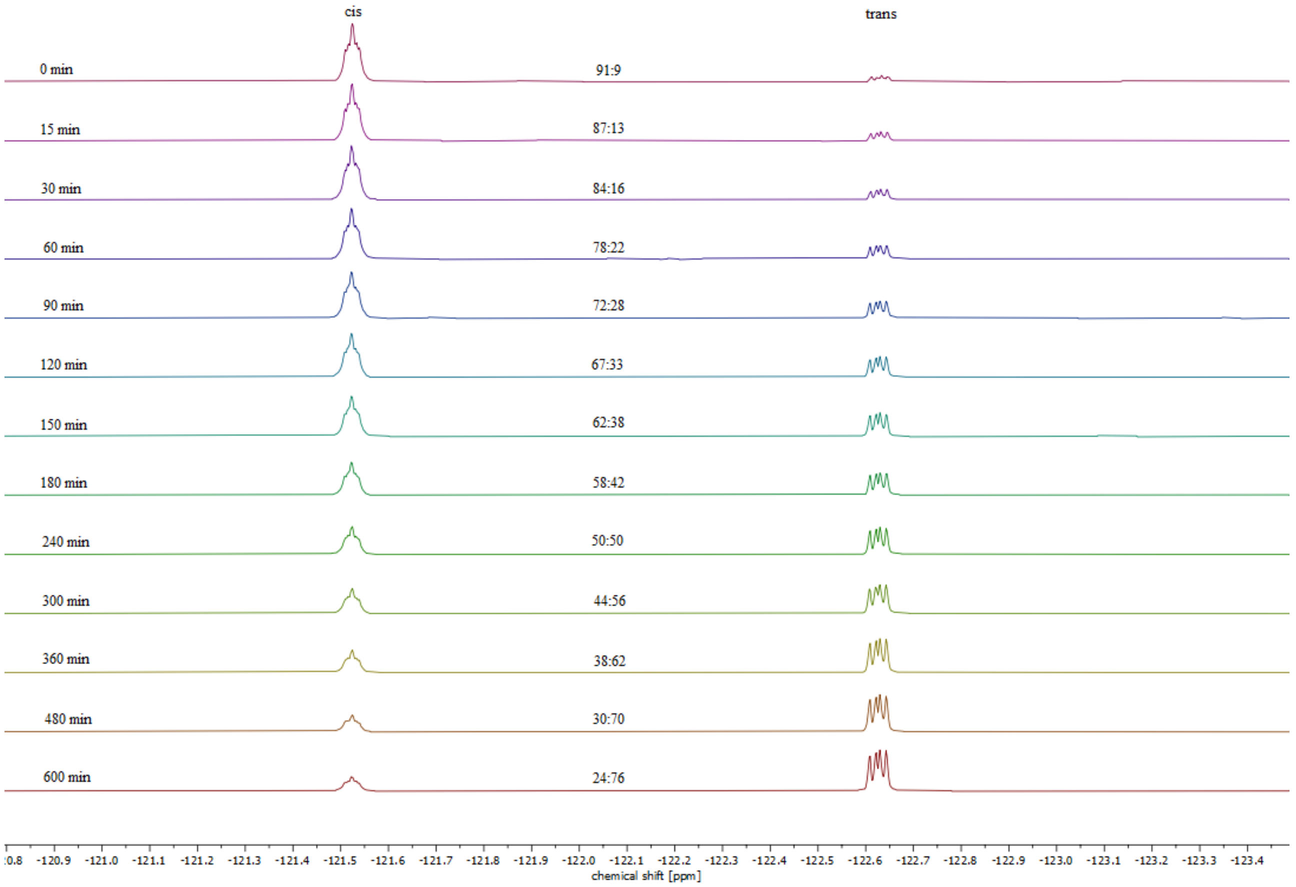
cis
trans
0 min
91:9
15 min
87:13
30 min
84:16
60 min
78:22
90 min
72:28
120 min
67:33
150 min
62:38
180 min
58:42
240 min
50:50
300 min
44:56
360 min
38:62
480 min
30:70
600 min
24:76
chemical shift [ppm]

(*E*/*Z*)-1,2-Bis(2-chloro-6-fluorophenyl)diazene (3)

Irradiation with 525 nm

$t_{1/2}$ (**3**, ^{19}F NMR, 500 µM, DMSO-d_6/D_2O 9:1, 90 °C) = 3.7 h.

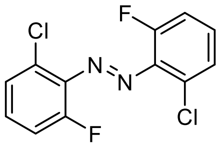

3

^{19}F NMR (471 MHz, DMSO-d_6/H_2O 9:1, 90 °C)

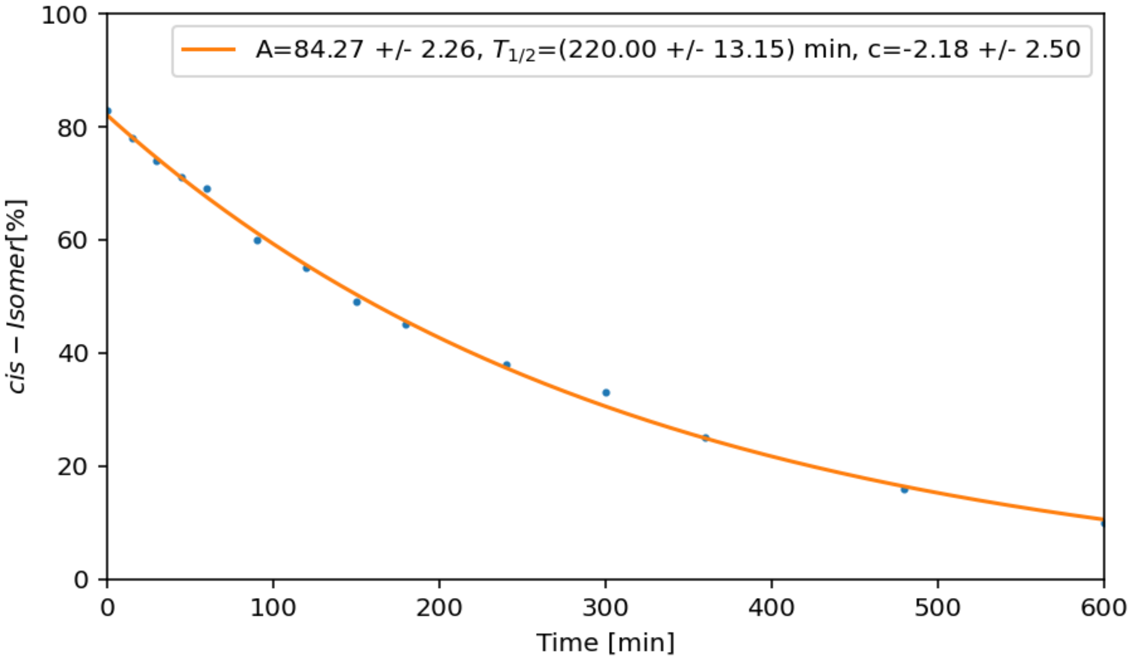

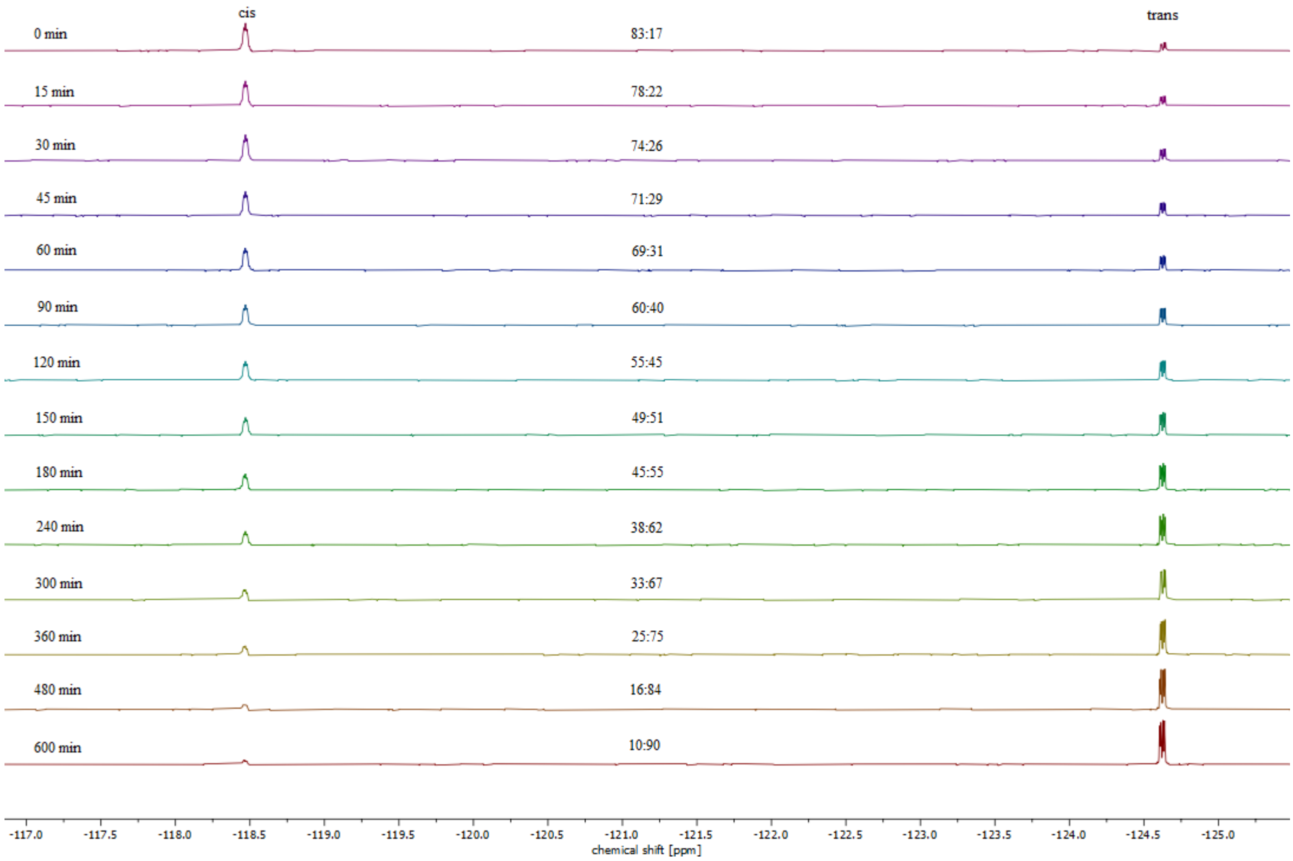

(*E*/*Z*)-1,2-Bis(2-bromo-6-fluorophenyl)diazene (4)

Irradiation with 525 nm

$t_{1/2}$ (**4**, ^{19}F NMR, 500 µM, DMSO-d_6/D_2O 9:1, 90 °C) = 1.82 h.

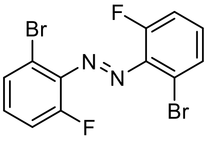

4

^{19}F NMR (471 MHz, DMSO-d_6/D_2O 9:1, 90 °C)

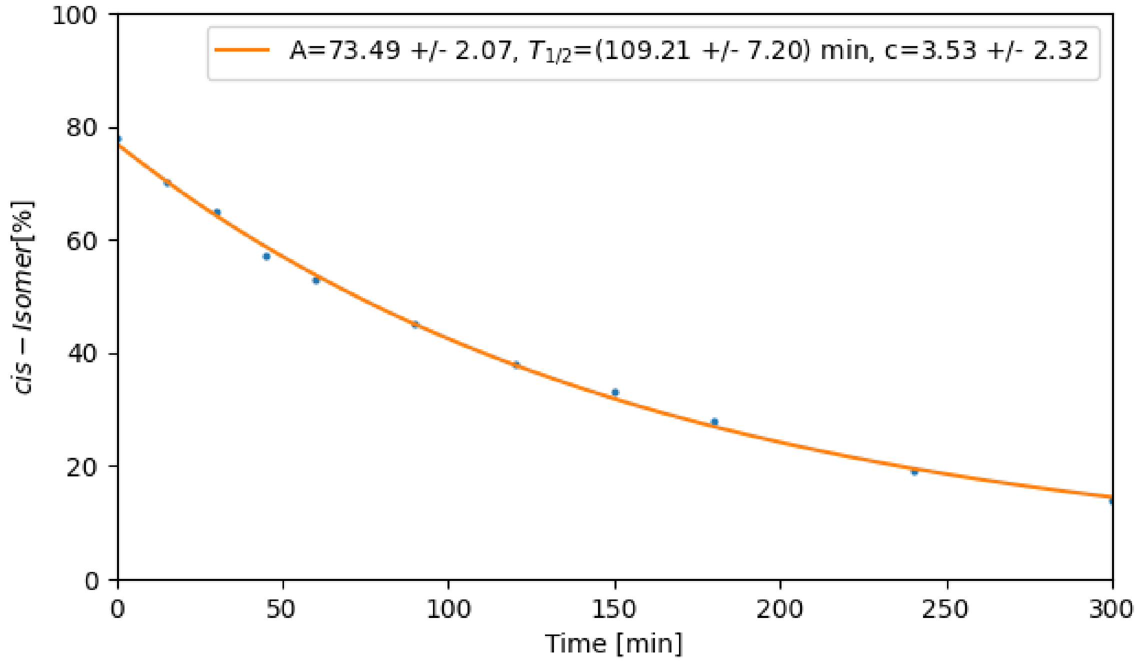
A=73.49 +/- 2.07, T1/2=(109.21 +/- 7.20) min, c=3.53 +/- 2.32
cis − Isomer[%]
Time [min]
0
20
40
60
80
100
0
50
100
150
200
250
300

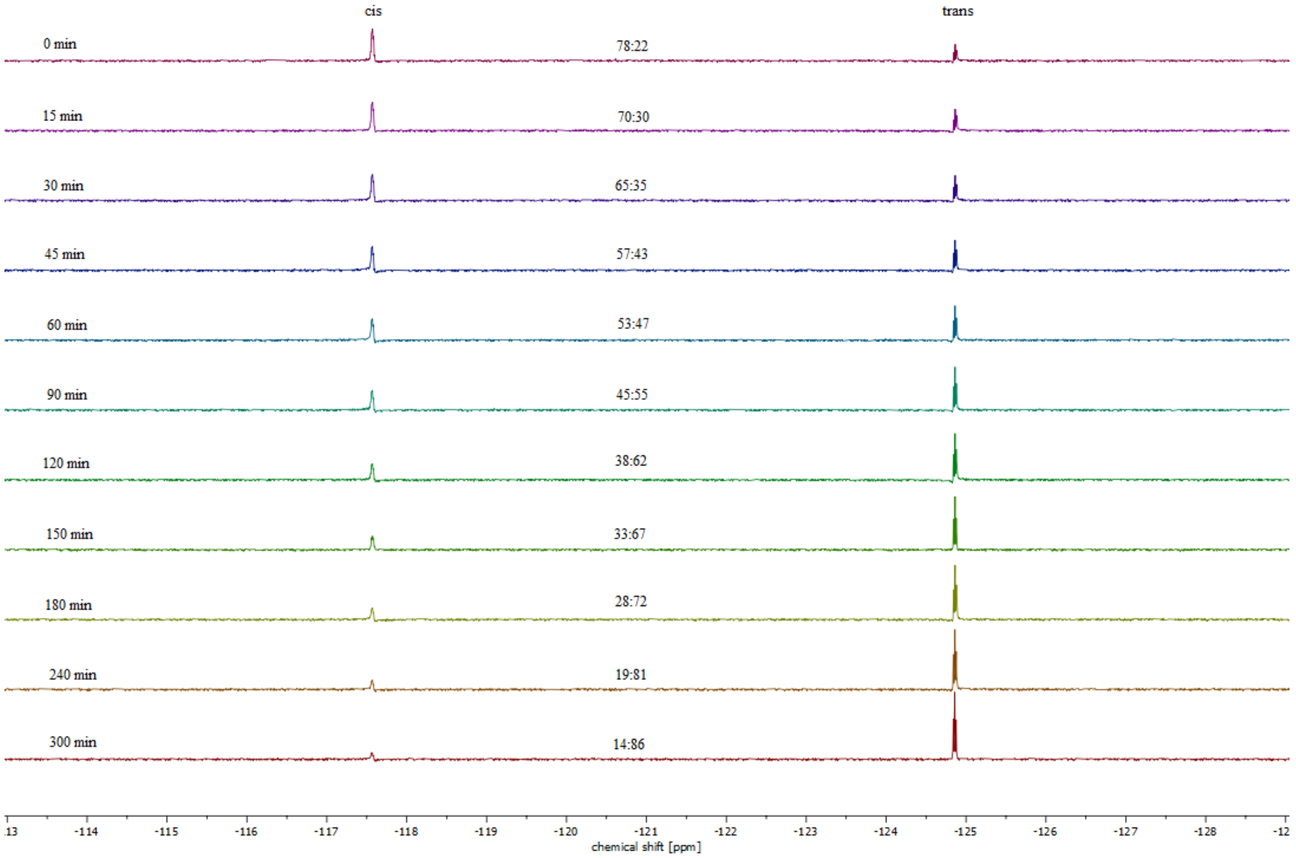
cis
trans
0 min
78:22
15 min
70:30
30 min
65:35
45 min
57:43
60 min
53:47
90 min
45:55
120 min
38:62
150 min
33:67
180 min
28:72
240 min
19:81
300 min
14:86
chemical shift [ppm]

(*E*/*Z*)-1,2-Bis(2,6-dichlorophenyl)diazene (6)

Irradiation with 650 nm

$t_{1/2}$ (**6**, UV-vis at 440 nm, 500 µM, DMSO-d_6/D_2O 9:1, 55 °C) = 9.03 h.

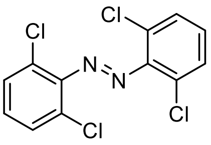

6
(440 nm, DMSO-d_6/D_2O 9:1, 55 °C)

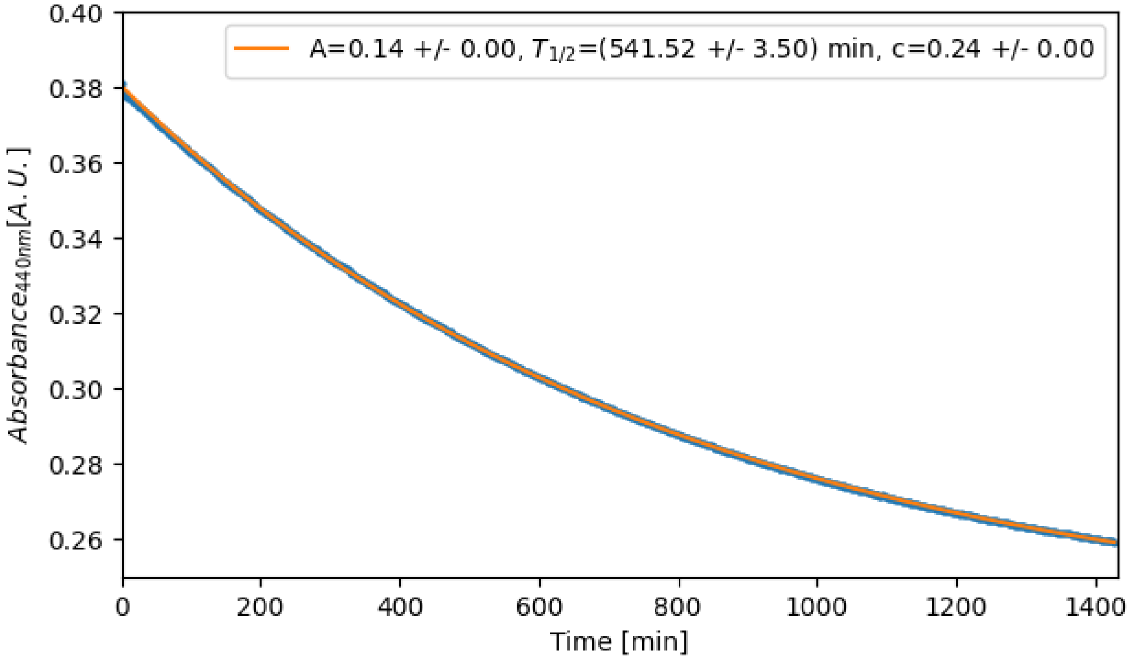

(*E*/*Z*)-1,2-Bis(2,6-dimethoxyphenyl)diazene (9)

Irradiation with 525 nm

$t_{1/2}$ (**9**, ^{1}H NMR, 500 µM, DMSO-d_6/D_2O 9:1, 90 °C) = 3.27 h

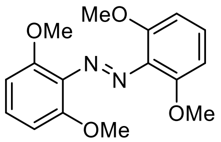

9
^{1}H NMR (500 MHz, DMSO-d_6/D_2O 9:1, 90 °C)

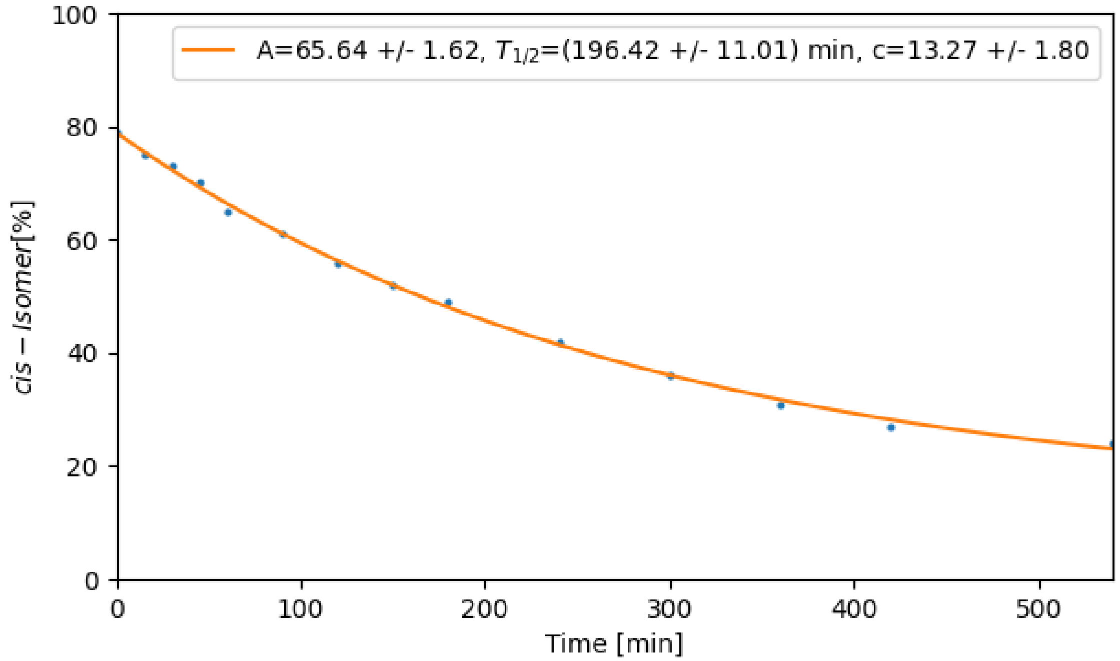
A=65.64 +/- 1.62, $T_{1/2}$=(196.42 +/- 11.01) min, c=13.27 +/- 1.80
cis – Isomer[%]
Time [min]
0
20
40
60
80
100
0
100
200
300
400
500

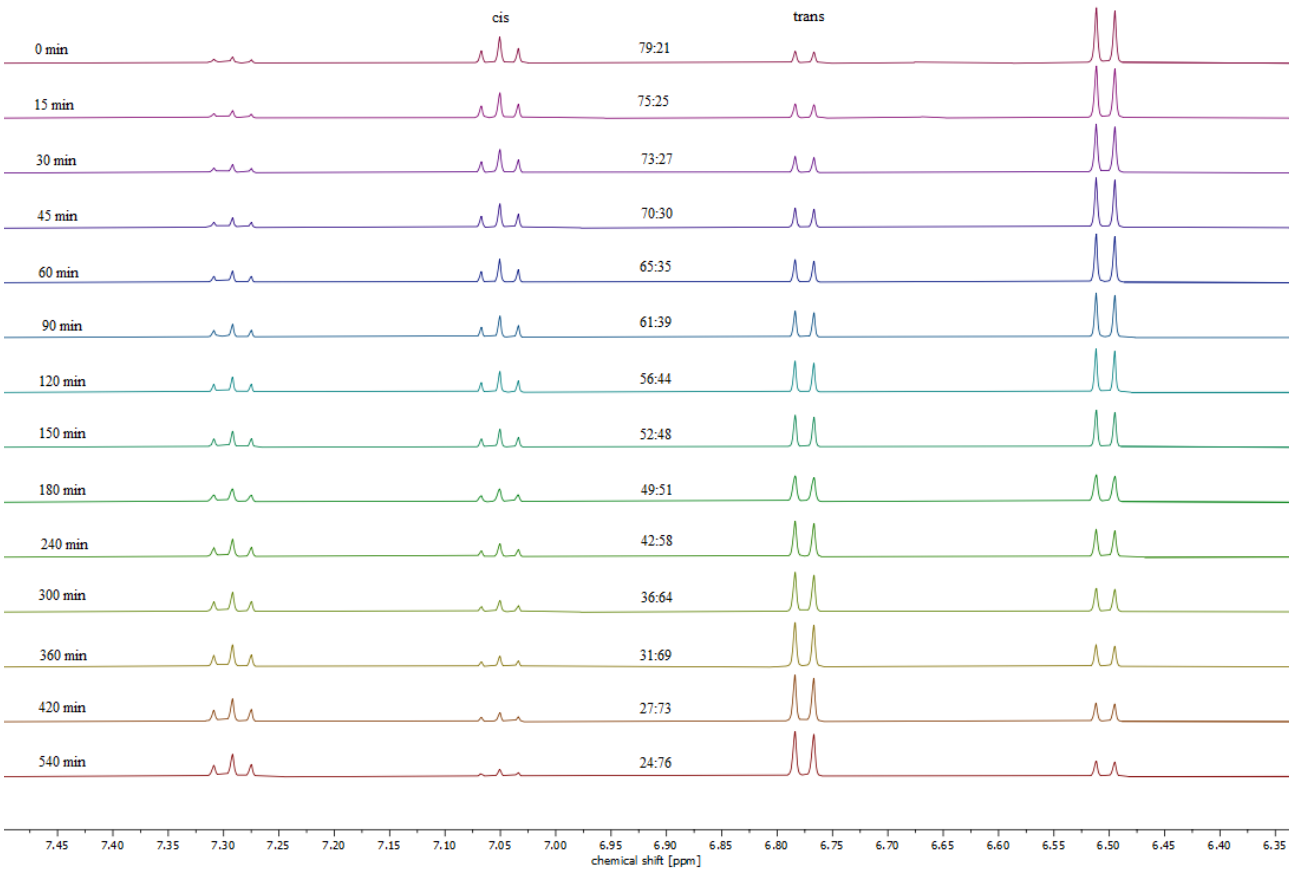
cis
trans
0 min
79:21
15 min
75:25
30 min
73:27
45 min
70:30
60 min
65:35
90 min
61:39
120 min
56:44
150 min
52:48
180 min
49:51
240 min
42:58
300 min
36:64
360 min
31:69
420 min
27:73
540 min
24:76
chemical shift [ppm]

(*E*/*Z*)-1,2-Bis(3-chloro-1-fluoronaphthalen-2-yl)diazene (12)

Irradiation with 650 nm

$t_{1/2}$ (**12**, ^{19}F NMR, 500 µM, DMSO-d_6/D_2O 9:1, 70 °C) = 60.1 min.

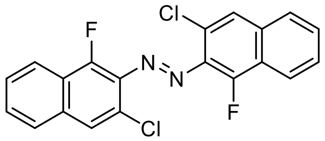

12

^{19}F NMR (471 MHz, DMSO-d_6/D_2O 9:1, 70 °C)

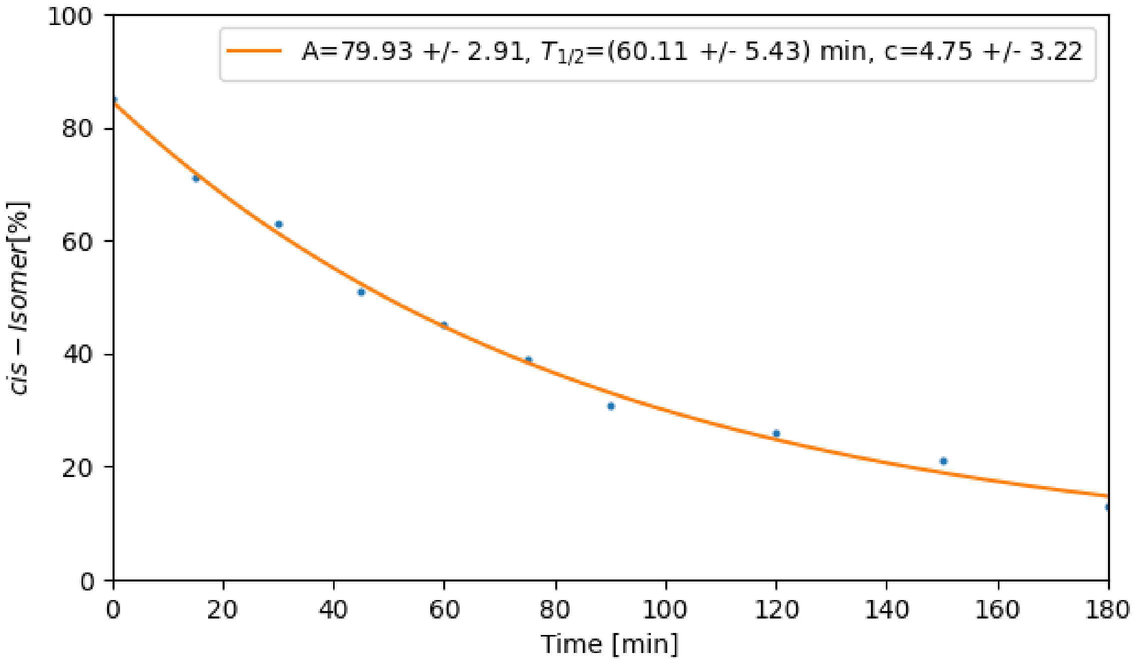

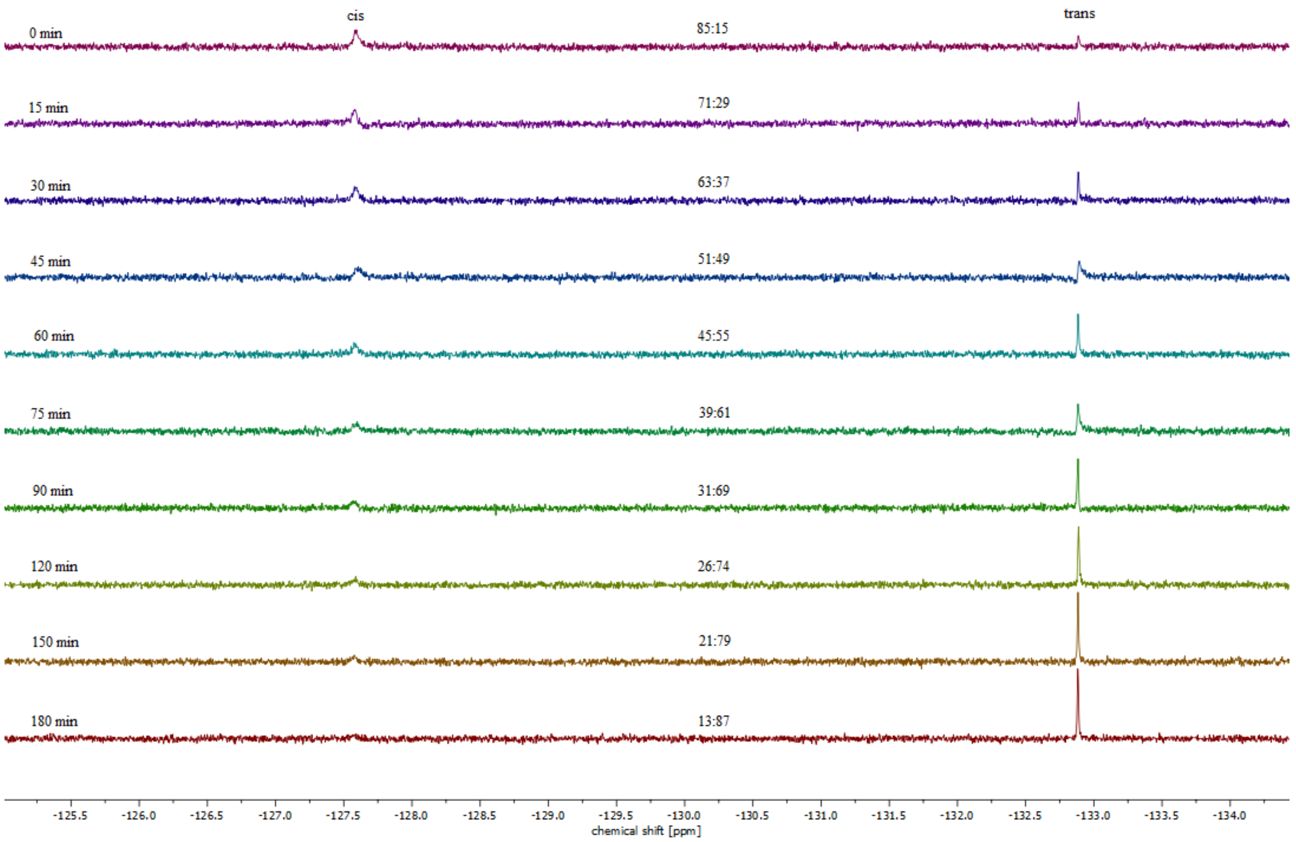

(*E*/*Z*)-3-Chloro-4-((2-chloro-6-fluorophenyl)diazenyl)-5-fluorobenzoic acid (17)

Irradiation with 525 nm

$t_{1/2}$ (**17**, ^{19}F NMR, 500 µM, DMSO-d_6/D_2O 9:1, 90 °C) = 69.6 min.

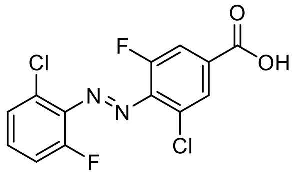

17

^{19}F NMR (471 MHz, DMSO-d_6/D_2O 9:1, 90 °C)

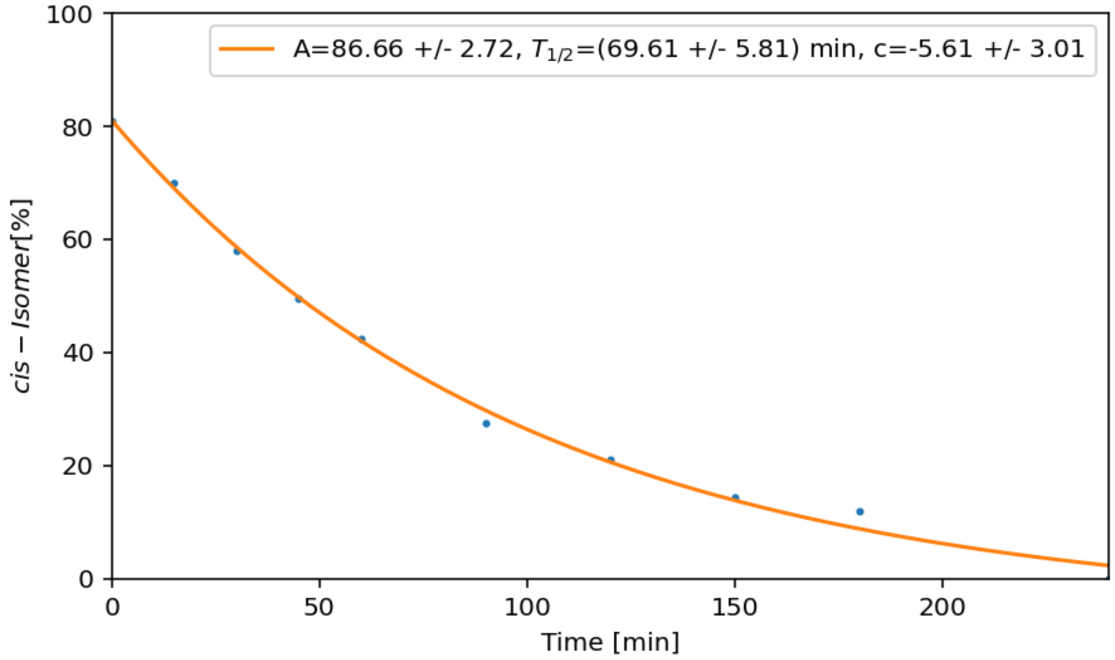
A=86.66 +/- 2.72, $T_{1/2}$=(69.61 +/- 5.81) min, c=-5.61 +/- 3.01
cis − Isomer[%]
Time [min]
0
20
40
60
80
100
0
50
100
150
200

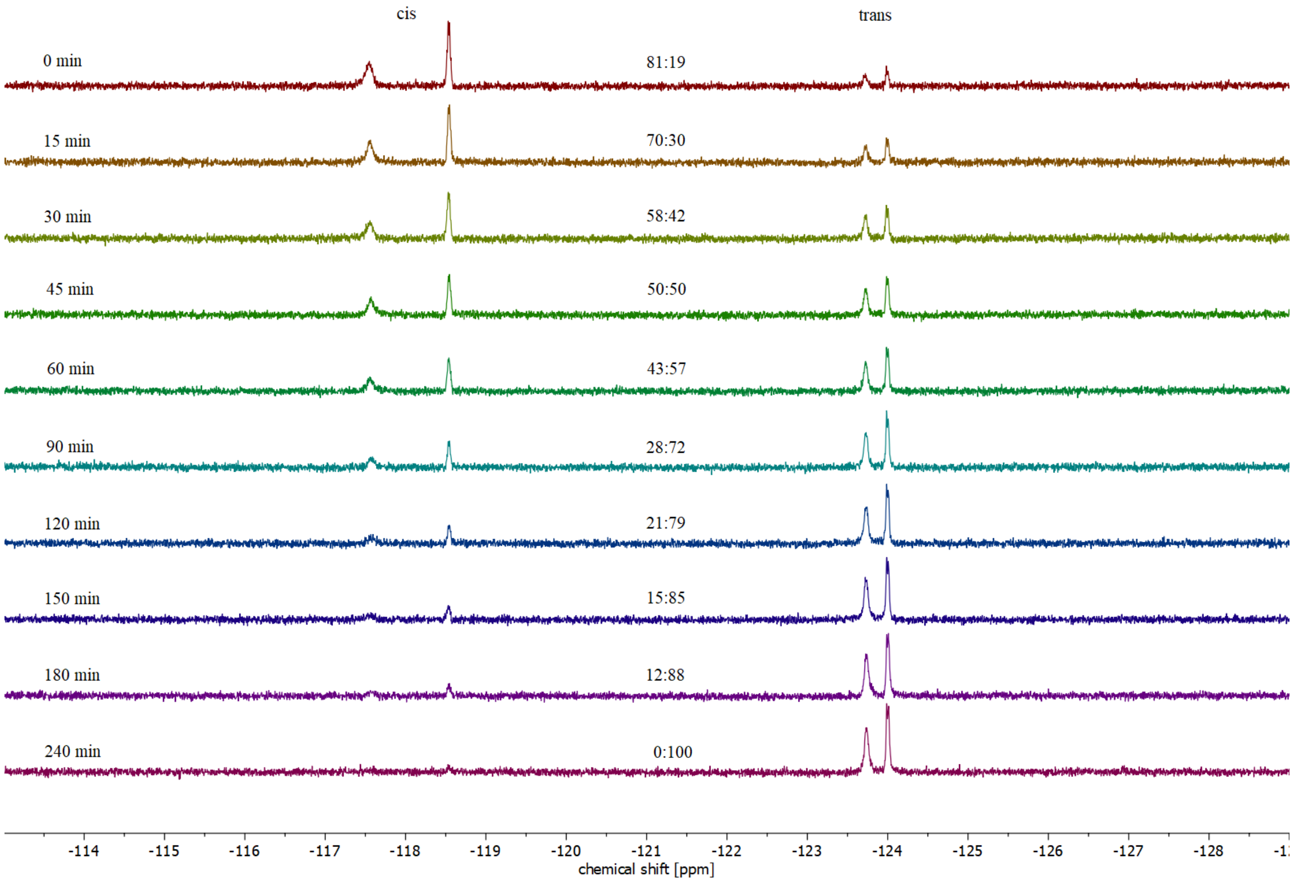
cis
trans
0 min
81:19
15 min
70:30
30 min
58:42
45 min
50:50
60 min
43:57
90 min
28:72
120 min
21:79
150 min
15:85
180 min
12:88
240 min
0:100
-114
-115
-116
-117
-118
-119
-120
-121
-122
-123
-124
-125
-126
-127
-128
chemical shift [ppm]

(*E*/*Z*)-1-(2-Chloro-6-fluoro-4-nitrophenyl)-2-(2-chloro-6-fluorophenyl)diazene (19)

Irradiation with 525 nm

$t_{1/2}$ (**19**, UV-vis at 425 nm, 50 µM, DMSO/H_2O 9:1, rt) = 12.6 s.

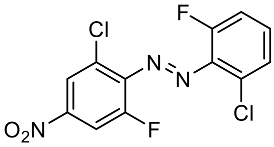

19

(425 nm, DMSO/H_2O 9:1, rt)

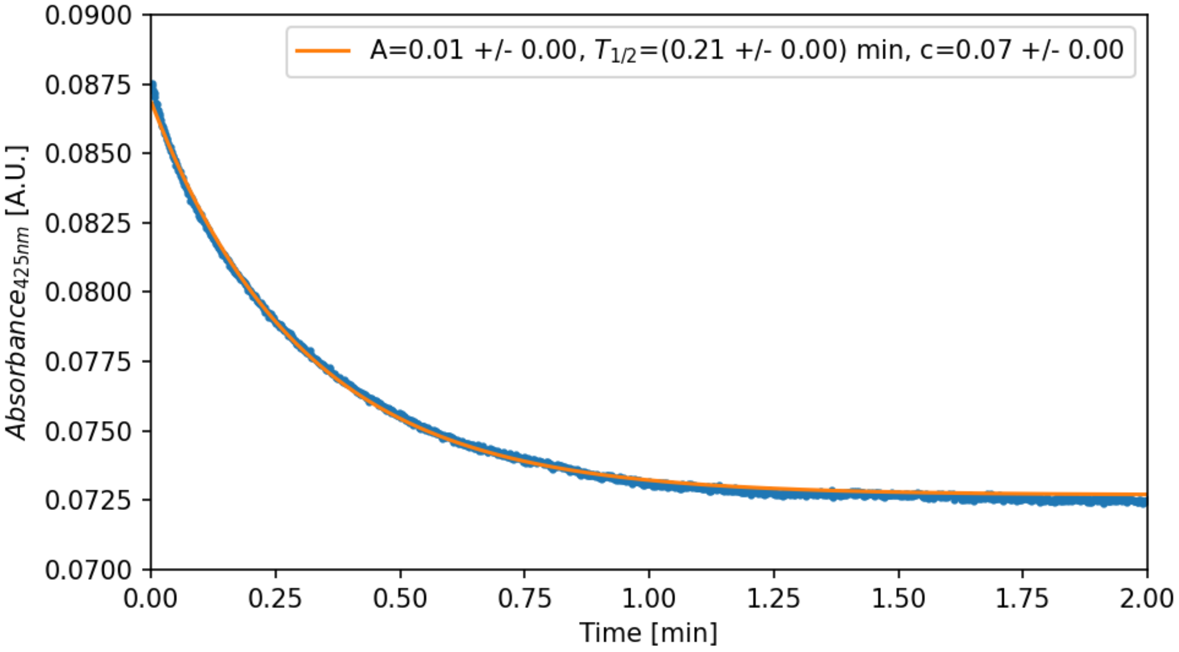

(*E*/*Z*)-3-Chloro-4-((2-chloro-6-fluoro-4-nitrophenyl)diazenyl)-5-fluorobenzoic acid (20)

Irradiation with 365 nm

$t_{1/2}$ (**20**, UV-vis at 325 nm, 50 µM, DMSO/H_2O 9:1, rt) = 25.7 min.

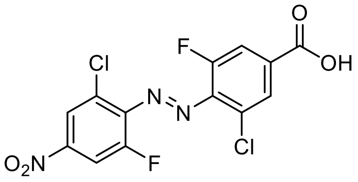

20

(325 nm, DMSO/H_2O 9:1, rt)

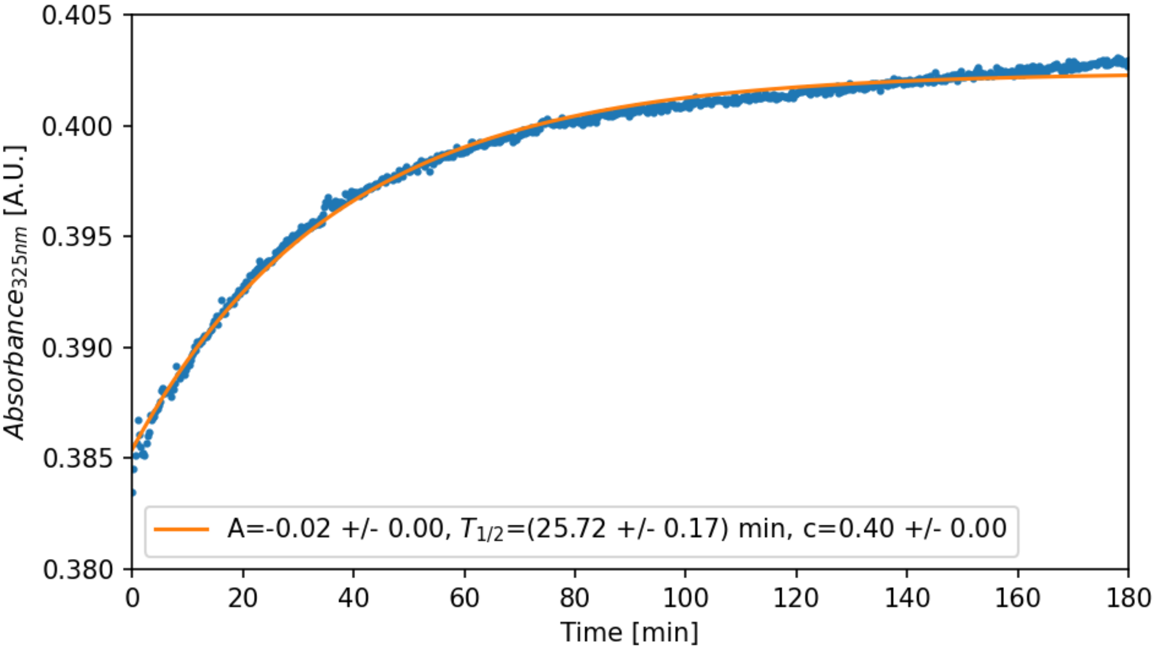

(*E*/*Z*)-Methyl-3-chloro-4-((2-chloro-6-fluorophenyl)diazenyl)-5-fluorobenzoate (21)

Irradiation with 650 nm

$t_{1/2}$ (**21**, ^{19}F NMR, 500 µM, DMSO-d_6/D_2O 9:1, 90 °C) = 44.0 min.

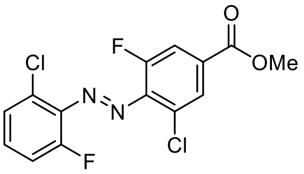

21

^{19}F NMR(471 MHz, DMSO-d_6/D_2O 9:1, 90 °C)

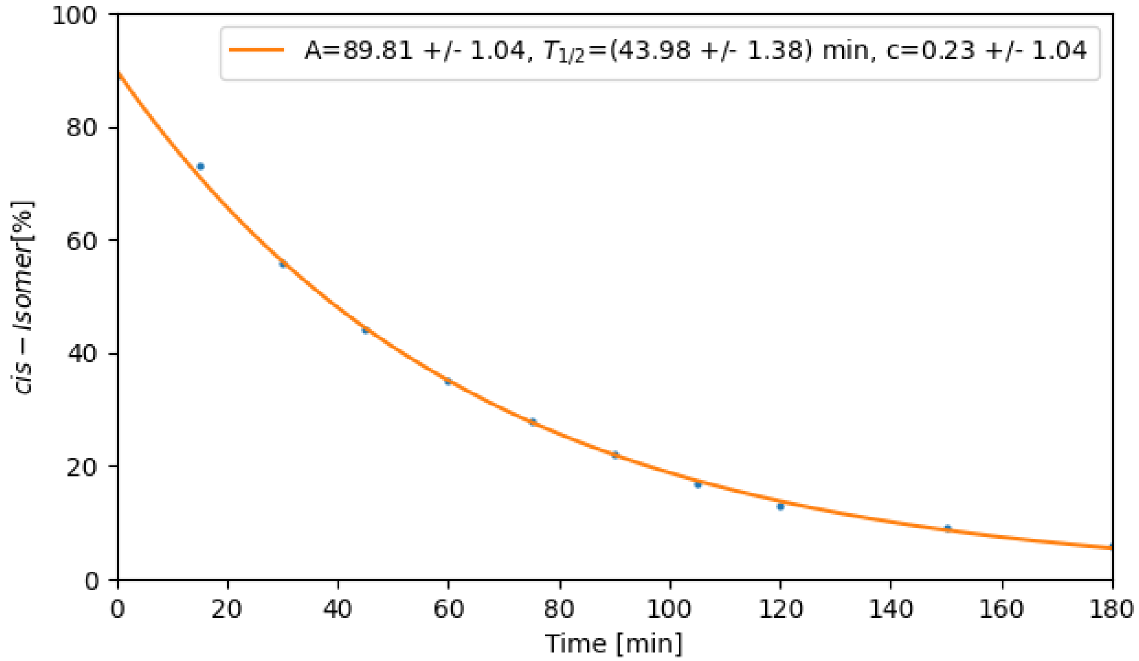
A=89.81 +/- 1.04, $T_{1/2}$=(43.98 +/- 1.38) min, c=0.23 +/- 1.04
cis – Isomer[%]
Time [min]
0
20
40
60
80
100
120
140
160
180

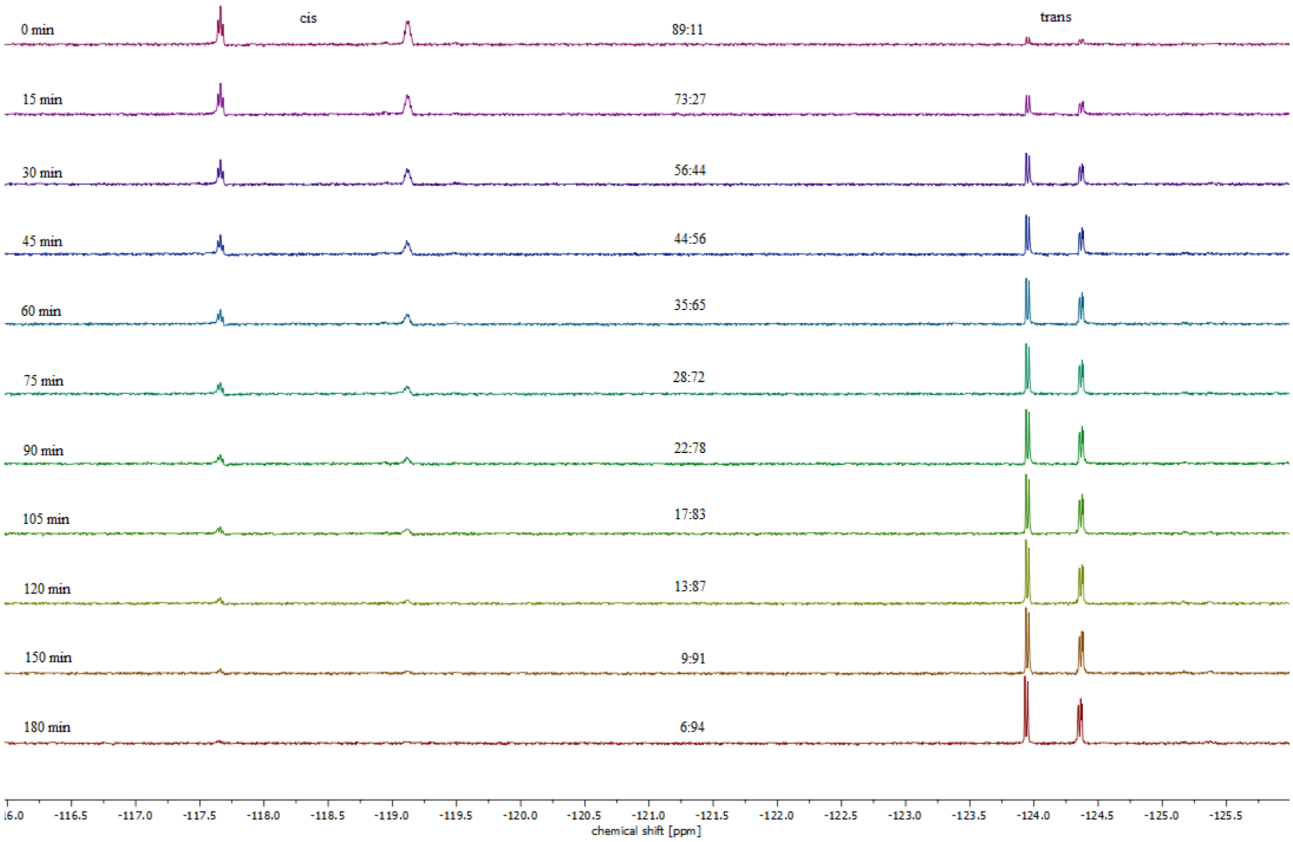
cis
trans
0 min
89:11
15 min
73:27
30 min
56:44
45 min
44:56
60 min
35:65
75 min
28:72
90 min
22:78
105 min
17:83
120 min
13:87
150 min
9:91
180 min
6:94
chemical shift [ppm]

(*E*/*Z*)-Methyl-3-bromo-4-((2-bromo-6-fluorophenyl)diazenyl)-5-fluorobenzoate (22)

Irradiation with 650 nm

$t_{1/2}$ (**22**, ^{19}F NMR, 500 µM, DMSO-d_6/D_2O 9:1, 90 °C) = 27.9 min.

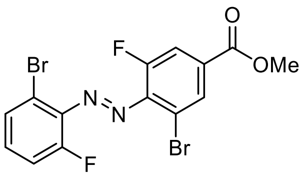

22

^{19}F NMR (471 MHz, DMSO-d_6/D_2O 9:1, 90 °C)

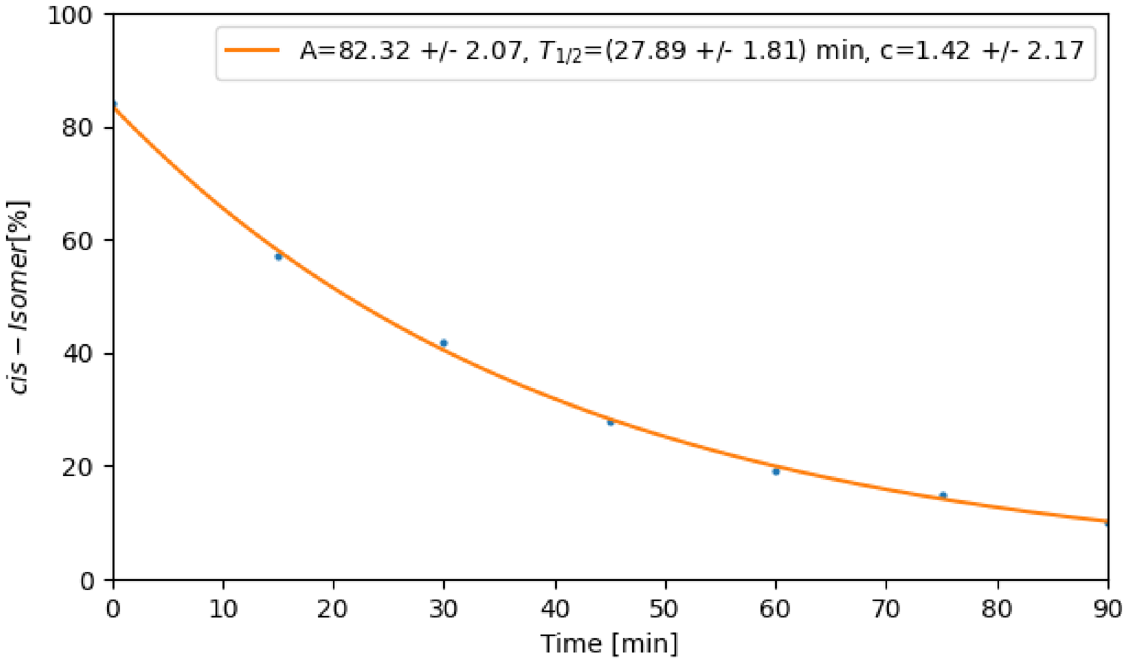

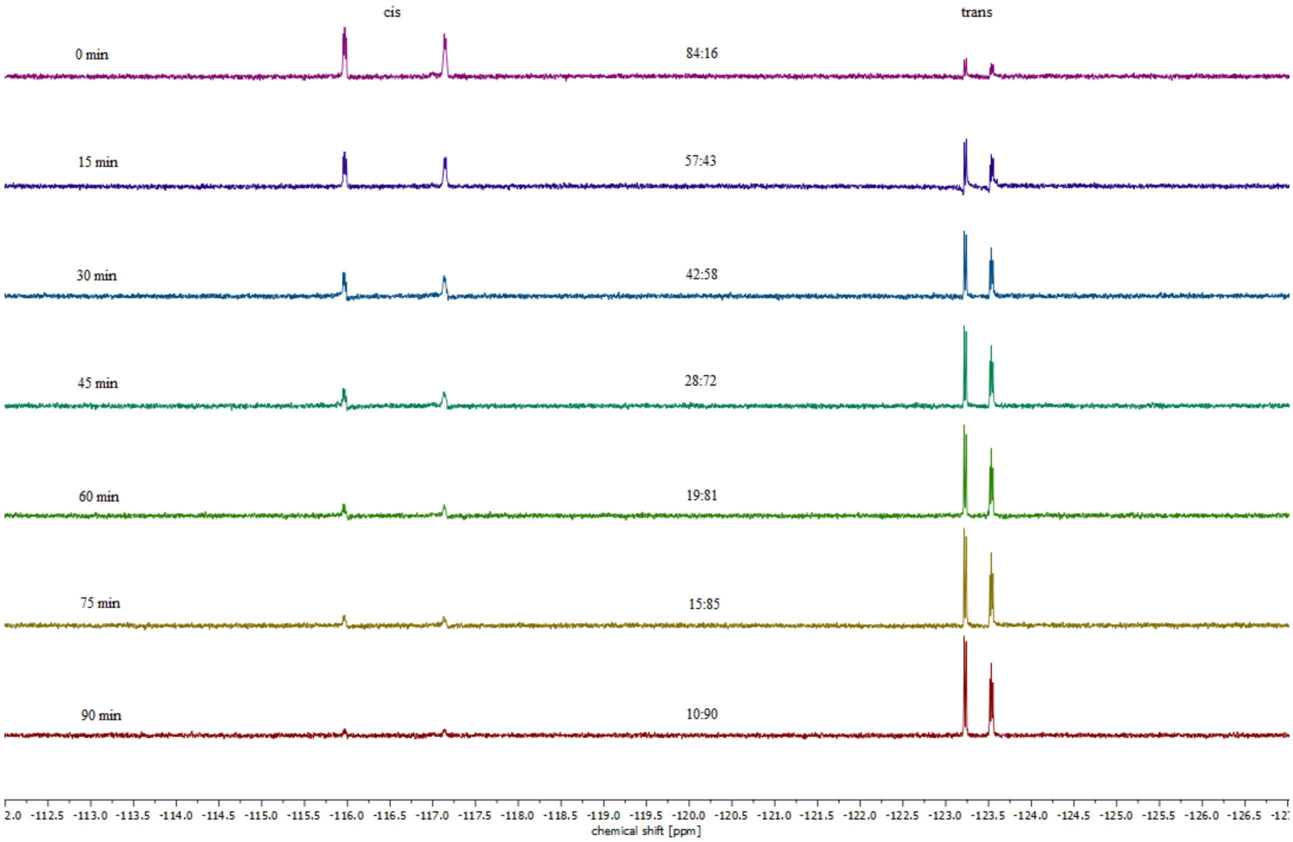

cis
trans
0 min
84:16
15 min
57:43
30 min
42:58
45 min
28:72
60 min
19:81
75 min
15:85
90 min
10:90
chemical shift [ppm]

(*E*/*Z*)-Methyl-3,5-dichloro-4-((2,6-dichlorophenyl)diazenyl)benzoate (23)

Irradiation with 525 nm

$t_{1/2}$ (**23**, UV-vis at 435 nm, 500 µM, DMSO-d_6/D_2O 9:1, 45 °C) = 1.95 h.

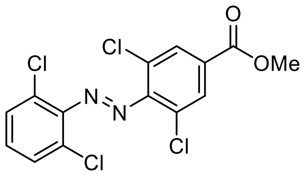

23

(435 nm, DMSO-d_6/D_2O 9:1, 45 °C)

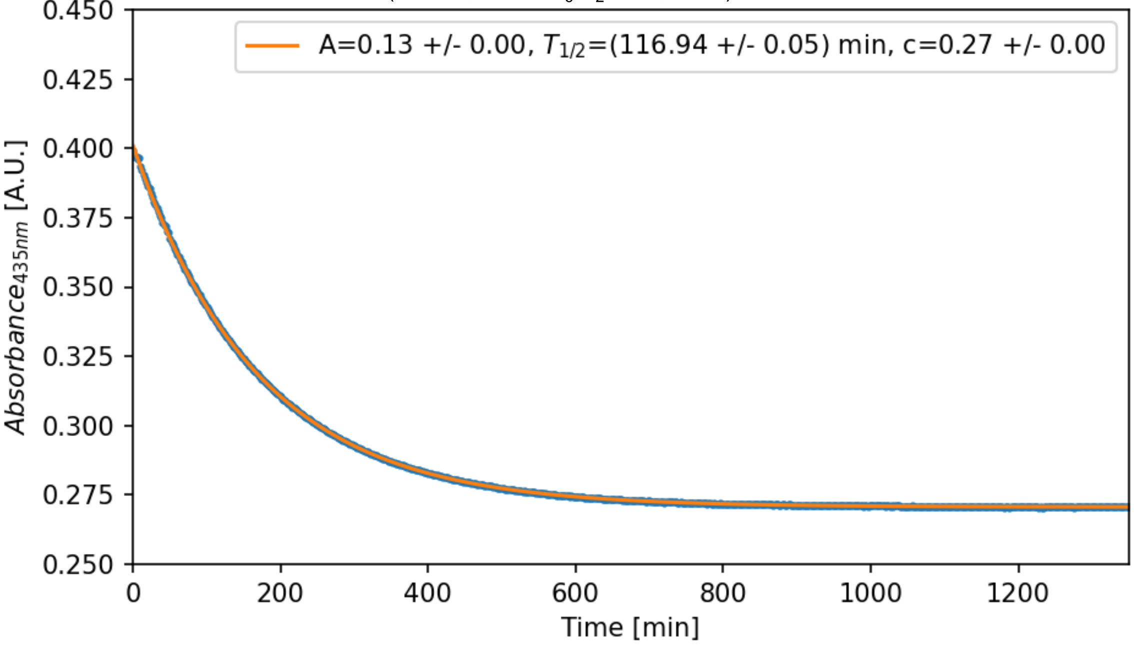

(*E*/*Z*)-3-Chloro-4-((2-chloro-6-fluorophenyl)diazenyl)-5-fluoro-*N*-methylbenzamide (24)

Irradiation with 650 nm

$t_{1/2}$ (**24**, ^{19}F NMR, 500 µM, DMSO-d_6/D_2O 9:1, 90 °C) = 79.0 min.

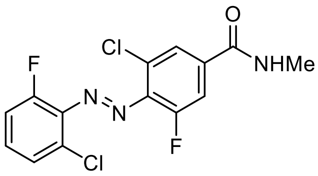

23

^{19}F NMR (471 MHz, DMSO-d_6/D_2O 9:1, 90 °C)

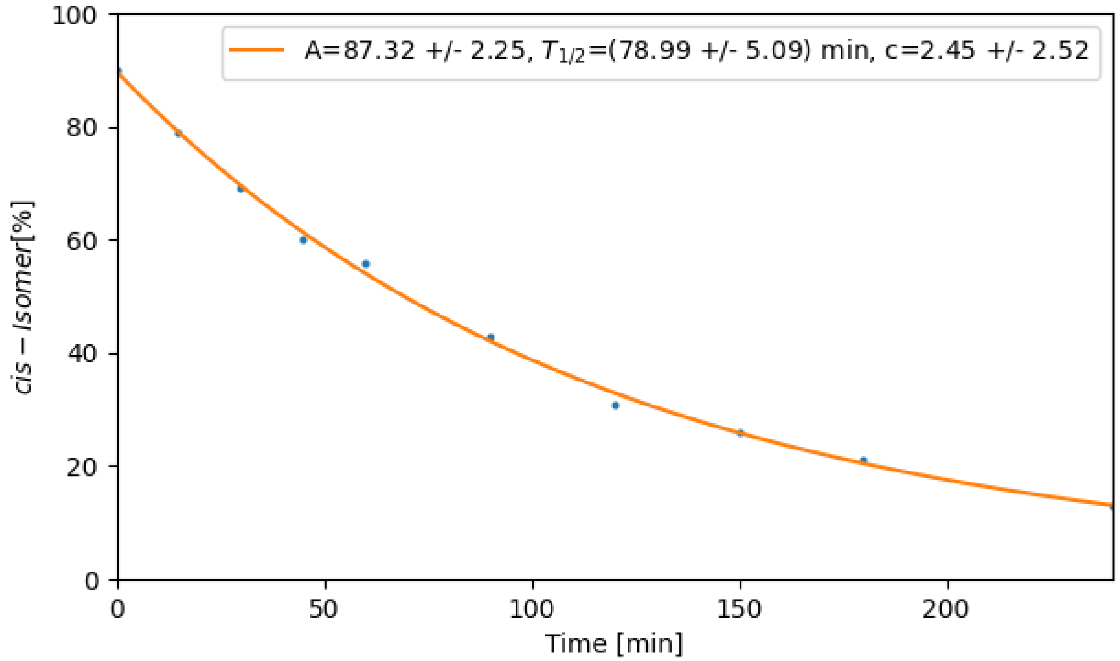
A=87.32 +/- 2.25, $T_{1/2}$=(78.99 +/- 5.09) min, c=2.45 +/- 2.52
cis – Isomer[%]
Time [min]
0
50
100
150
200
0
20
40
60
80
100

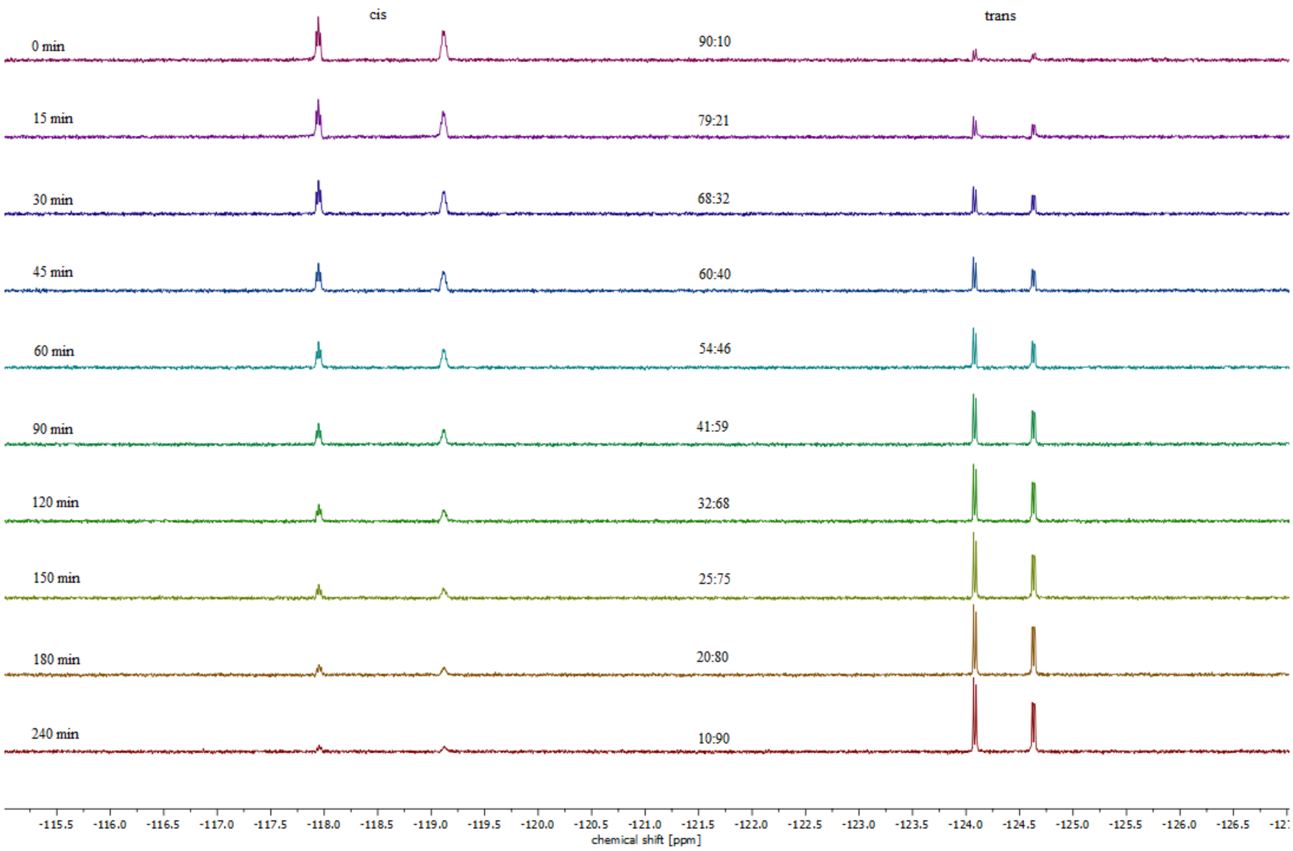
cis
trans
0 min
90:10
15 min
79:21
30 min
68:32
45 min
60:40
60 min
54:46
90 min
41:59
120 min
32:68
150 min
25:75
180 min
20:80
240 min
10:90
-115.5 -116.0 -116.5 -117.0 -117.5 -118.0 -118.5 -119.0 -119.5 -120.0 -120.5 -121.0 -121.5 -122.0 -122.5 -123.0 -123.5 -124.0 -124.5 -125.0 -125.5 -126.0 -126.5
chemical shift [ppm]

(*E*/*Z*)-Dimethyl 4,4'-(diazene-1,2-diyl)-bis(3-chloro-5-fluorobenzoate) (28)

Irradiation with 650 nm

$t_{1/2}$ (**28**, ^{19}F NMR, 500 µM, DMSO-d_6/D_2O 9:1, 55 °C) = 38.8 min.

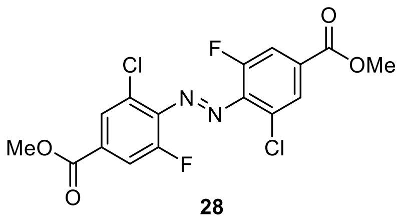

28

^{19}F NMR (471 MHz, DMSO-d_6/D_2O 9:1, 55 °C)

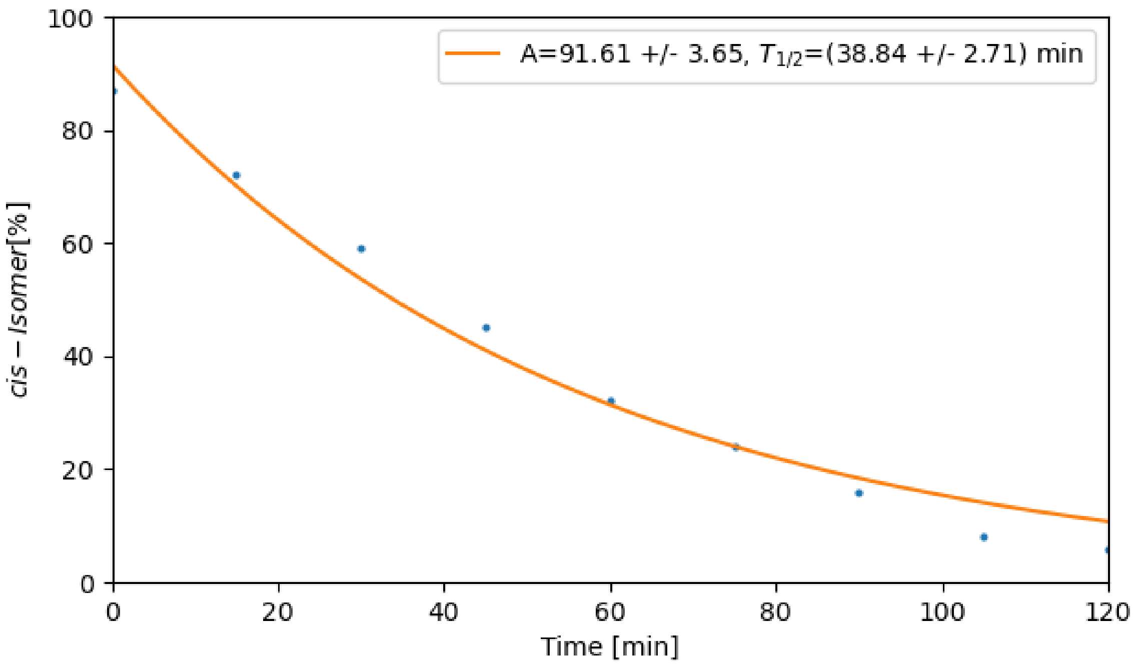

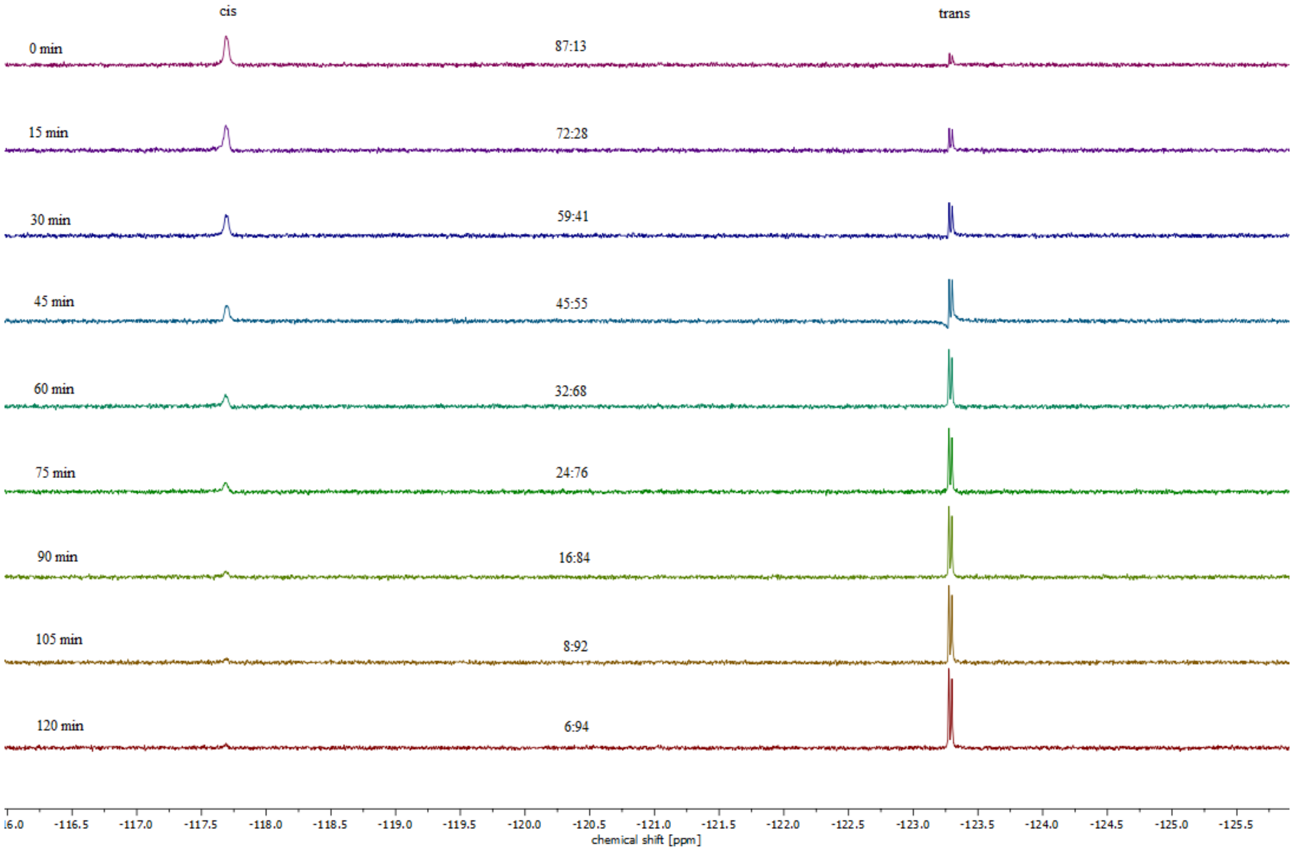

(*E*/*Z*)-3-Chloro-4-((2-chloro-6-fluorophenyl)diazenyl)-5-fluoroaniline (38)

Irradiation with 365 nm

$t_{1/2}$ (**38**, ^{19}F NMR, 500 µM, DMSO-d_6/D_2O 9:1, 60 °C) = 52.2 min.

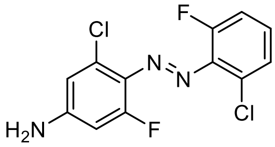

38

^{19}F NMR (471 MHz, DMSO-d_6/D_2O 9:1, 60 °C)

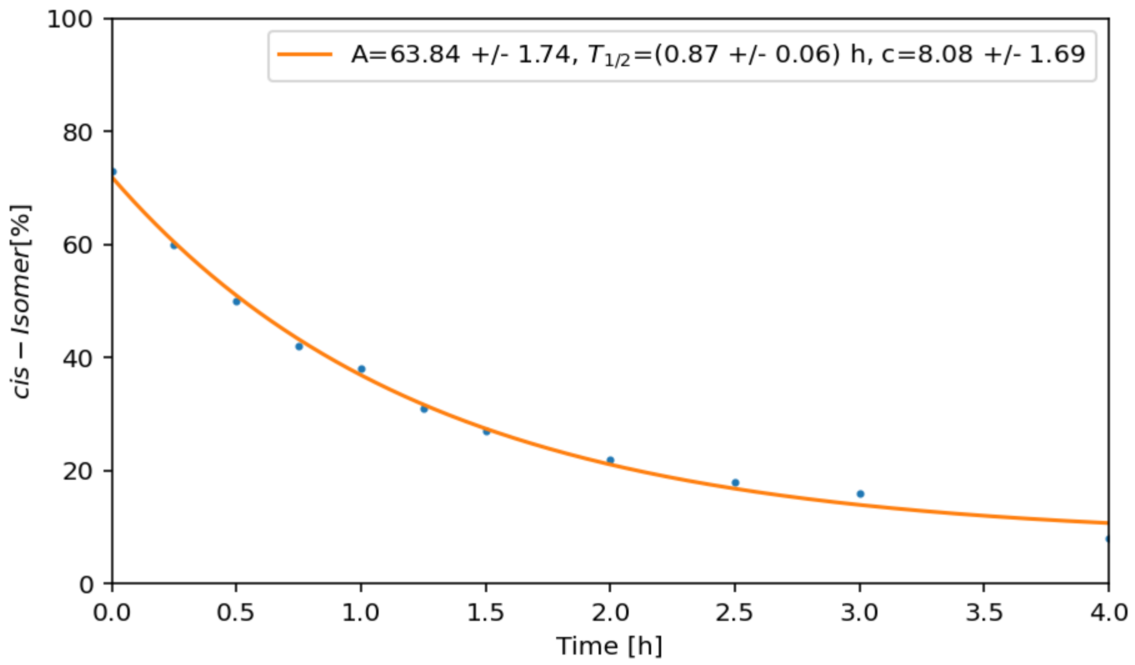
A=63.84 +/- 1.74, $T_{1/2}$=(0.87 +/- 0.06) h, c=8.08 +/- 1.69
cis − Isomer[%]
Time [h]
100
80
60
40
20
0
0.0
0.5
1.0
1.5
2.0
2.5
3.0
3.5
4.0

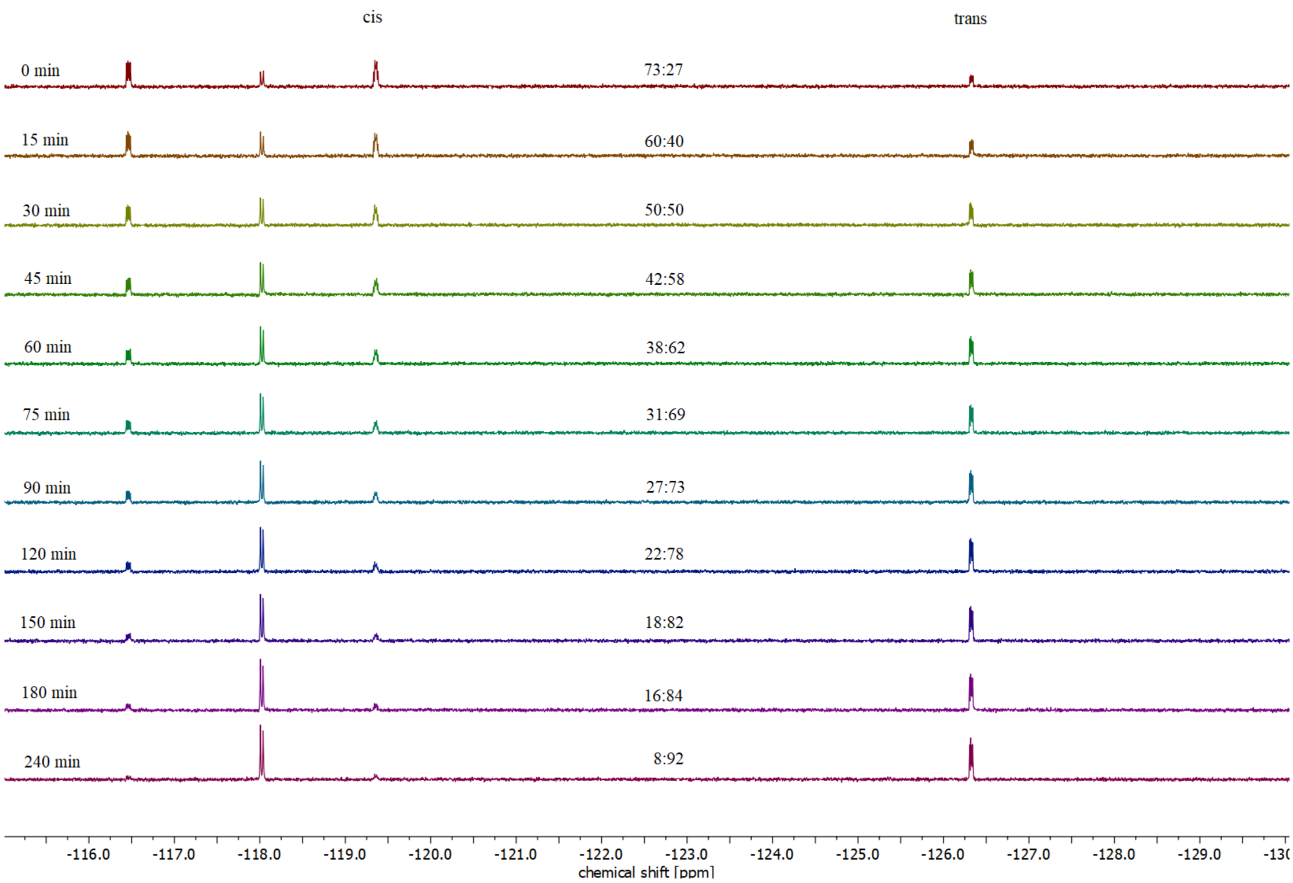
cis
trans
0 min
73:27
15 min
60:40
30 min
50:50
45 min
42:58
60 min
38:62
75 min
31:69
90 min
27:73
120 min
22:78
150 min
18:82
180 min
16:84
240 min
8:92
-116.0
-117.0
-118.0
-119.0
-120.0
-121.0
-122.0
-123.0
-124.0
-125.0
-126.0
-127.0
-128.0
-129.0
chemical shift [ppm]

(*E*/*Z*)-*N*-(3-chloro-4-((2-chloro-6-fluorophenyl)diazenyl)-5-fluorophenyl)acetamide)

Irradiation with 365 nm

$t_{1/2}$ (**39**, ^{19}F NMR, 500 µM, DMSO-d_6/D_2O 9:1, 90 °C) = 46.2 min.

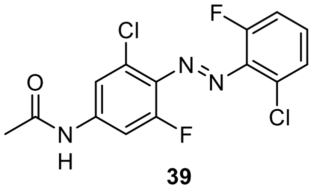

39

^{19}F NMR (471 MHz, DMSO-d_6/D_2O 9:1, 90 °C)

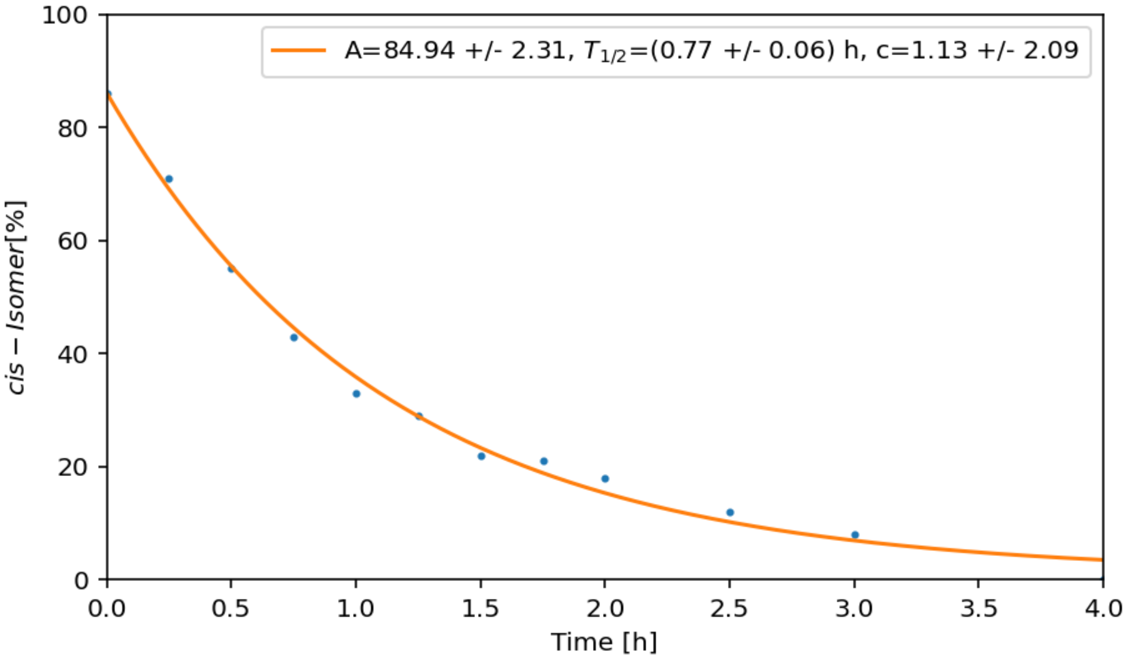

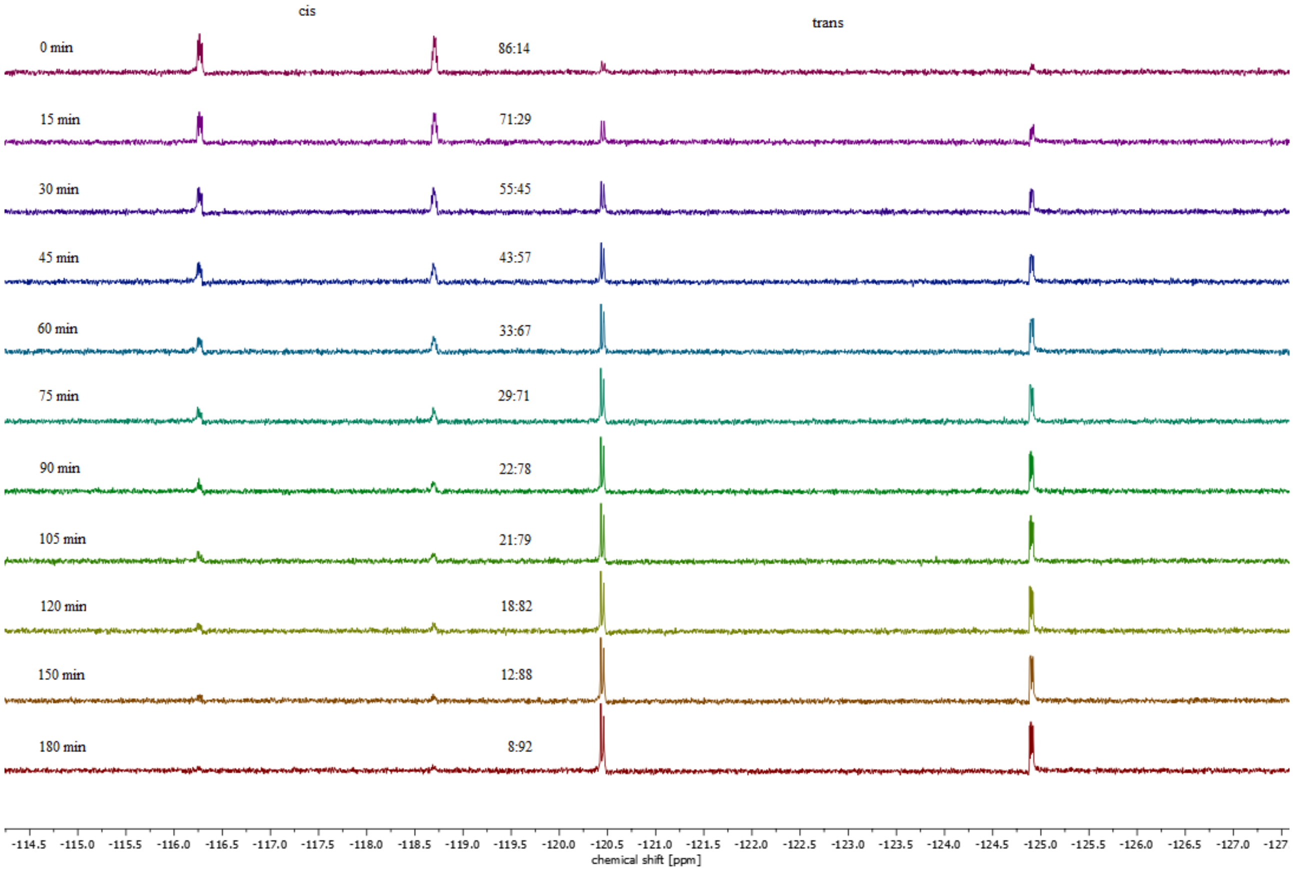
cis
trans
0 min
86:14
15 min
71:29
30 min
55:45
45 min
43:57
60 min
33:67
75 min
29:71
90 min
22:78
105 min
21:79
120 min
18:82
150 min
12:88
180 min
8:92
chemical shift [ppm]

(*E*/*Z*)-Methyl-3-chloro-4-((2-chloro-6-fluoro-4-methoxyphenyl)diazenyl)-5-fluorobenzoate (46)

Irradiation with 525 nm

$t_{1/2}$ (**46**, UV-vis at 360 nm, 500 µM, DMSO-d_6/D_2O 9:1, 45 °C) = 4.31°h.

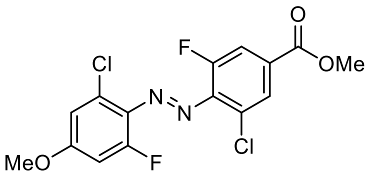

46

(360 nm, DMSO-d_6/D_2O 9:1, 45 °C)

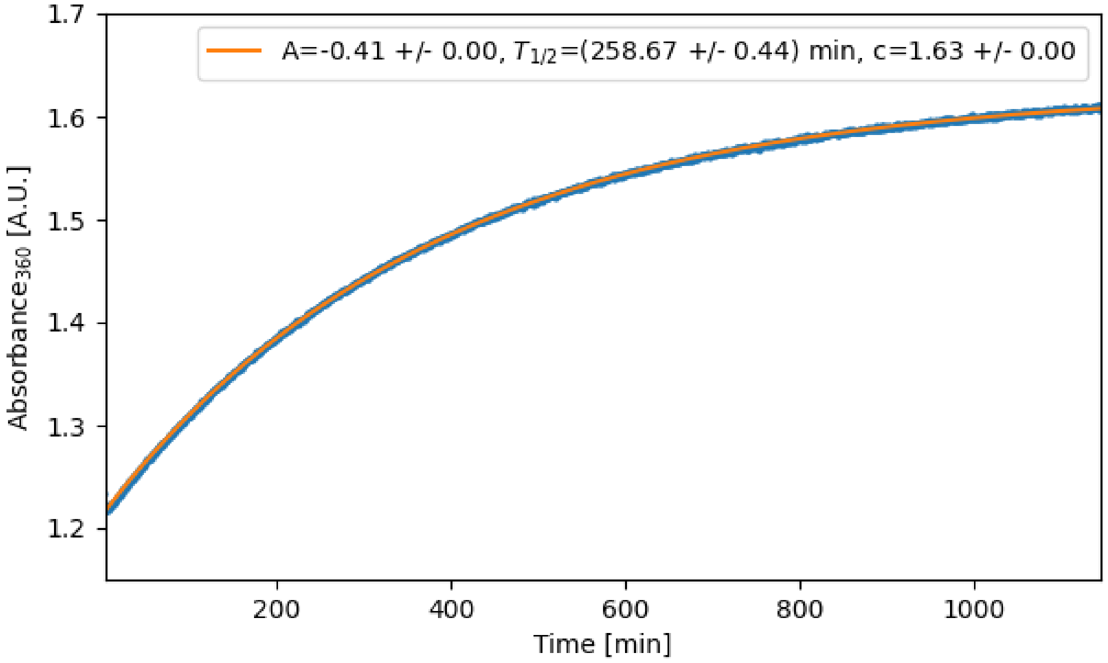

(*E*/*Z*)-4-((4-Amino-2,6-dichlorophenyl)diazenyl)-3,5-dichlorobenzenesulfonamide (48)

Irradiation with 365 nm

$t_{1/2}$ (**48**, UV-vis at 390 nm, 50 µM, DMSO/H_2O 9:1, rt) = 33.0°s.

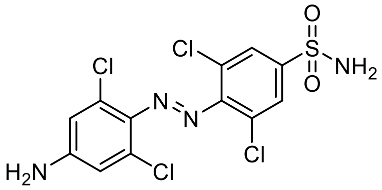

48

(390 nm, 9:1 $(CH_3)_2SO$:H_2O, rt)

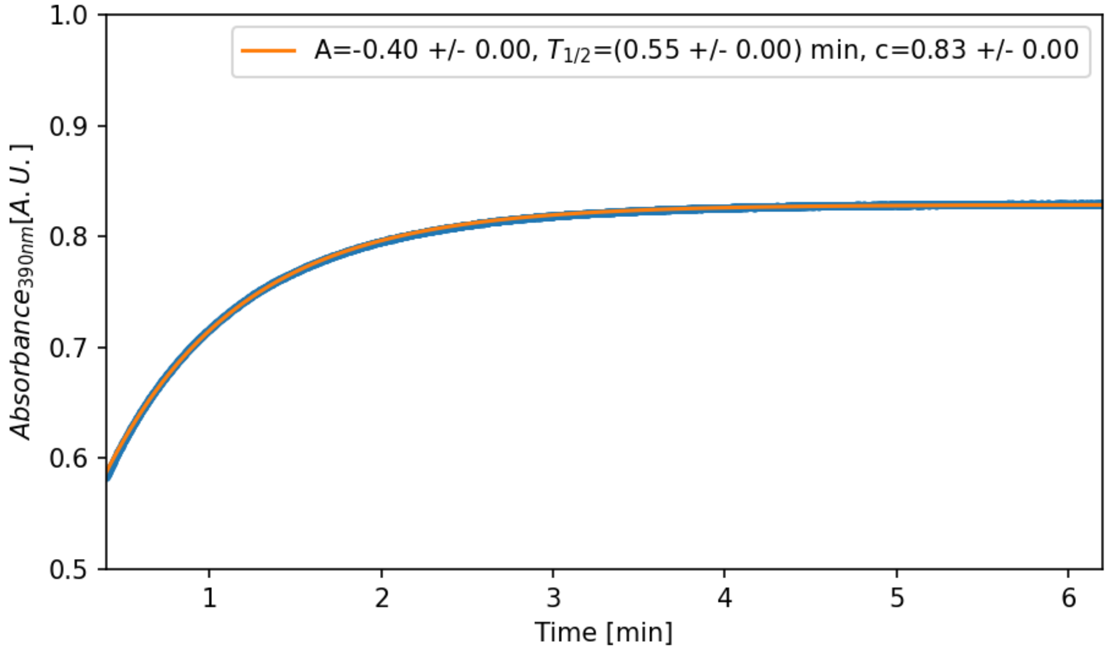

(*E*/*Z*)-3-Chloro-4-((2-chloro-6-fluoro-4-hydroxyphenyl)diazenyl)-5-fluorobenzoic acid (49)

Irradiation with 365 nm

$t_{1/2}$ (**49**, UV-vis at 420 nm, 50 µM, DMSO/D_2O 9:1, rt) = 0.12°s.

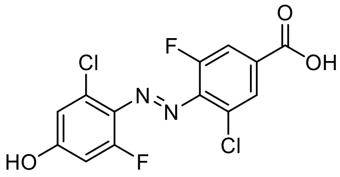

49

(420 nm, DMSO-d_6/D_2O 9:1, rt)

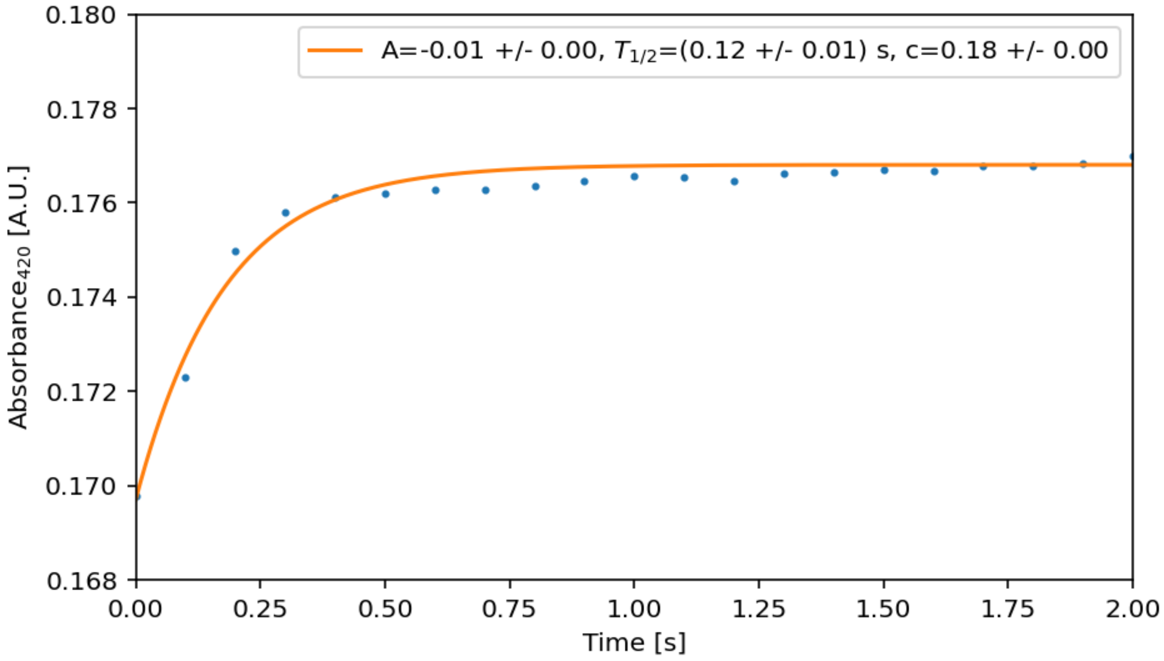

(*E*/*Z*)-1-(1-(4-(4-((4-Butyl-2-chloro-6-fluorophenyl)diazenyl)-3-chloro-5-fluorophenyl)butanoyl)-piperidin-4-yl)-1,3-dihydro-2*H*-benzo[*d*]imidazol-2-one (dfdc-OptoBI-1)

Irradiation with 525 nm

$t_{1/2}$ (UV-vis at 435 nm, 500 µM, DMSO-d_6/D_2O 9:1, 60 °C) = 409.7 min.

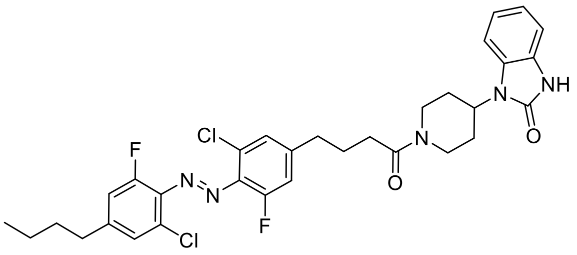

dfdc-OptoBI-1

(435 nm, 500 µM, DMSO-d_6/D_2O 9:1, 60 °C)

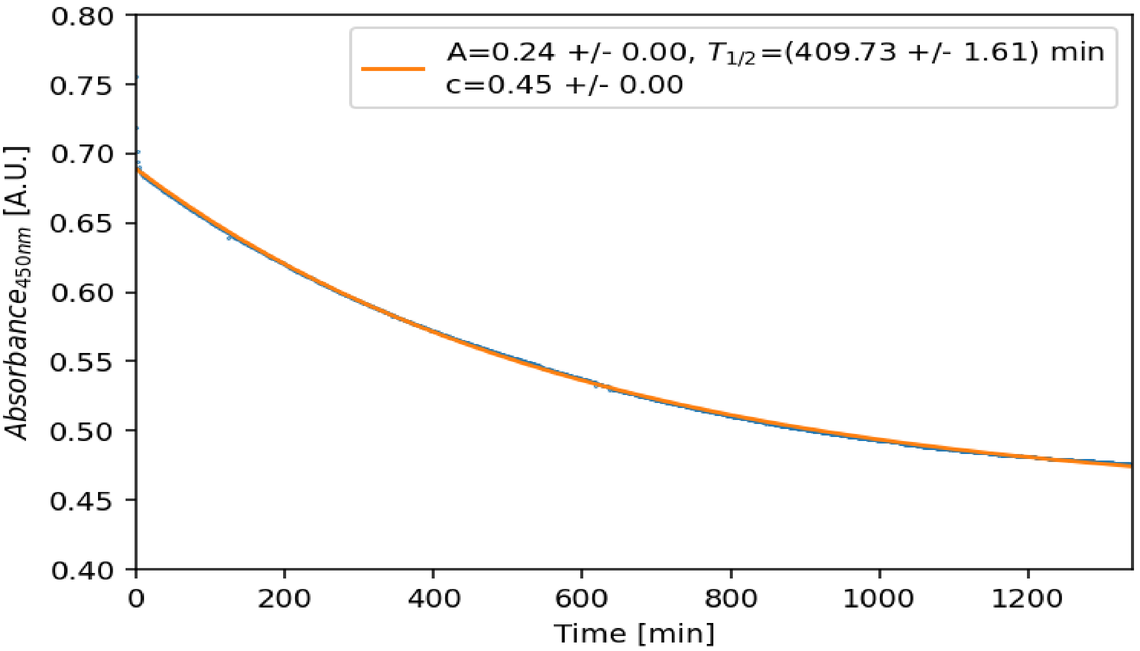

6. Molecular Absorption Coefficient ε

The molecular absorption coefficient ε was calculated by the Lambert-Beer law using the measured UV-vis data for the corresponding azobenzene derivates with A being the absorbance, c being the concentration and l being the path length.

$$\varepsilon = \frac{A}{cl} \quad \textbf{(6.1)}$$

7. Quantum Yield Φ

To assess the quantum yield Φ of selected azobenzene derivates [500 μM DMSO-d_6/D_2O 9:1], the absorbance change during the E→Z isomerization over time was measured via UV-vis while the PSS were determined via NMR analysis. From the absorbance data A (t), the integrated photokinetic factor x(t) was calculated, which was then used to plot the molar fraction of the *Z*-isomer χ_Z versus the photokinetic factor x(t) (cf. equation (**7.1**)) according to Knie *et al.*[1]

$$x(t) = \int_{t_0}^{t} \frac{1-10^{-A(t)}}{A\,(t)}\, dt \quad \textbf{(7.1)}$$

These were fitted using an exponential model to extract the kinetic parameter B and the final composition at PSS $\chi_{Z,\infty}$. With I_0 being the intensity of the light source, l the length of the path, ε_E the molar absorption coefficient from the *E*-isomer and V the volume, the quantum yield Φ_{EZ} was calculated using equation (**7.2**).[1]

$$B = \frac{I_0 \cdot l \cdot \Phi_{EZ} \cdot \varepsilon_E}{V \cdot \chi_{Z,\infty}} \quad \textbf{(7.2)}$$

(*E*/*Z*)-1,2-Bis(2-chloro-6-fluorophenyl)diazene (3)

$\Phi_{E\rightarrow Z}$ =0.001257

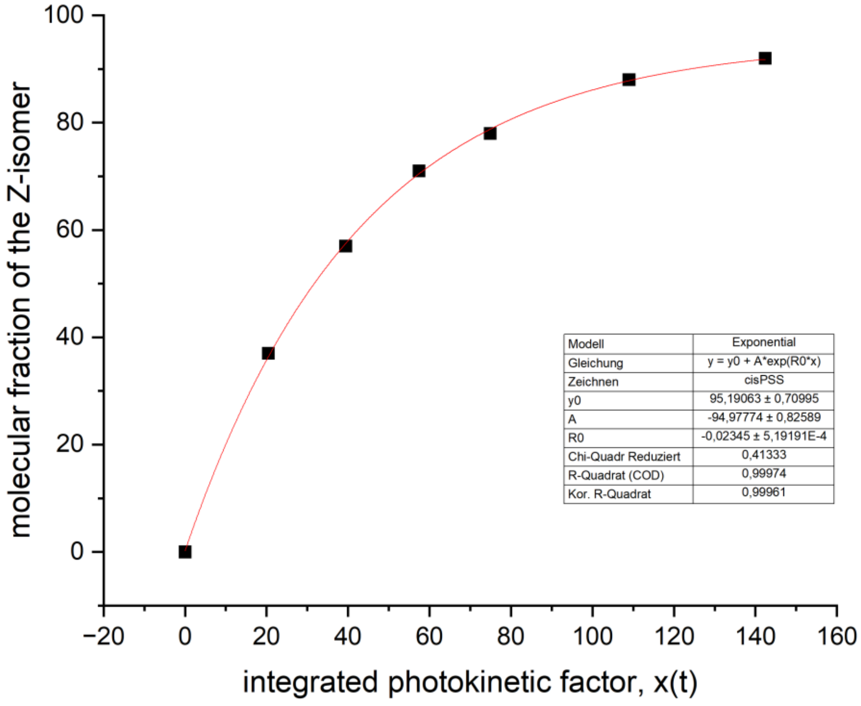

(*E*/*Z*)-1,2-Bis(2-bromo-6-fluorophenyl)diazene (4)

$\Phi_{E\rightarrow Z}$ = 0.002988

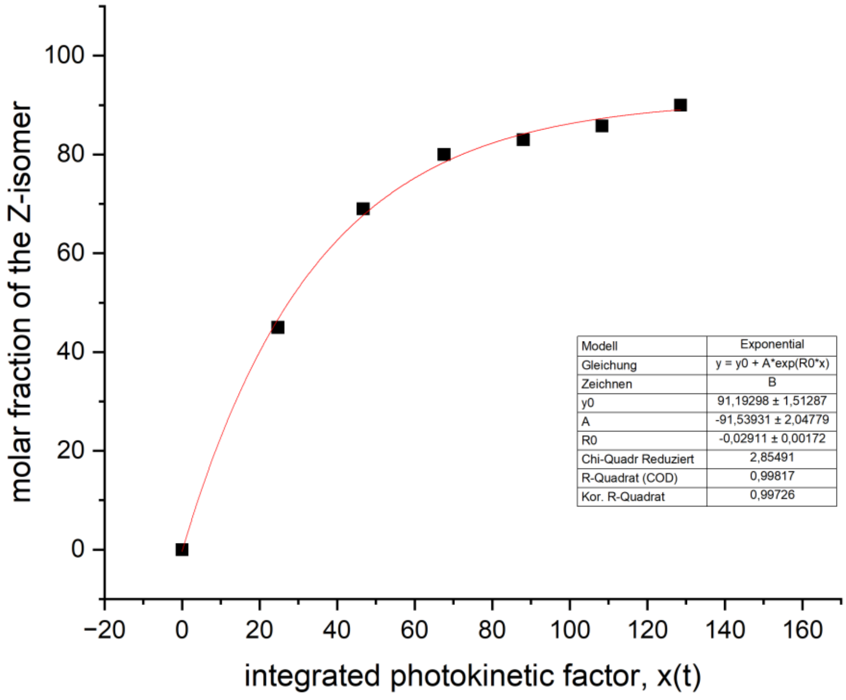

(*E*/*Z*)-1,2-Bis(2,6-dichlorophenyl)diazene (6)

$\Phi_{E \to Z} = 0.00446$

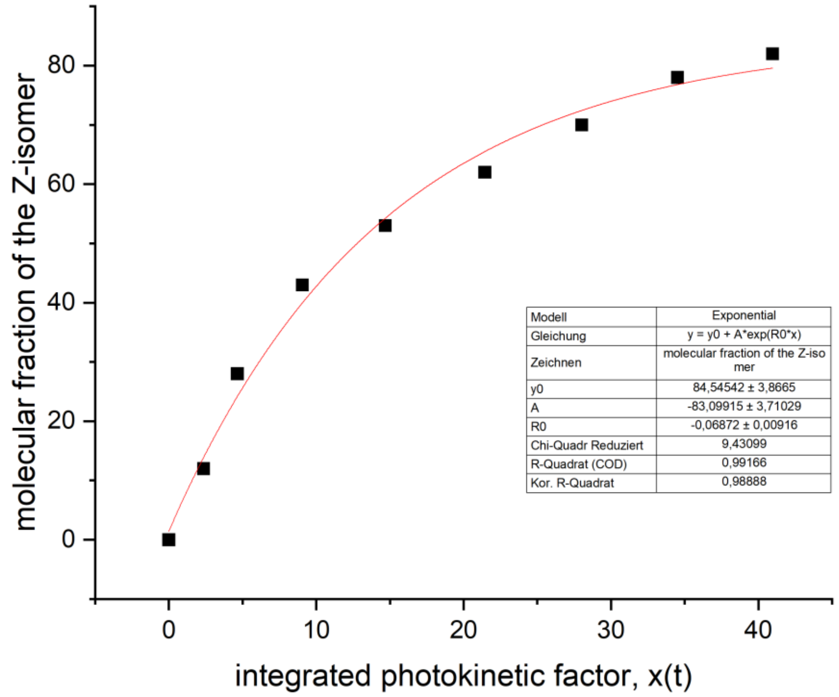

(*E*/*Z*)-1,2-Bis(2,6-dimethoxyphenyl)diazene (9)

$\Phi_{E \to Z} = 0.002218$

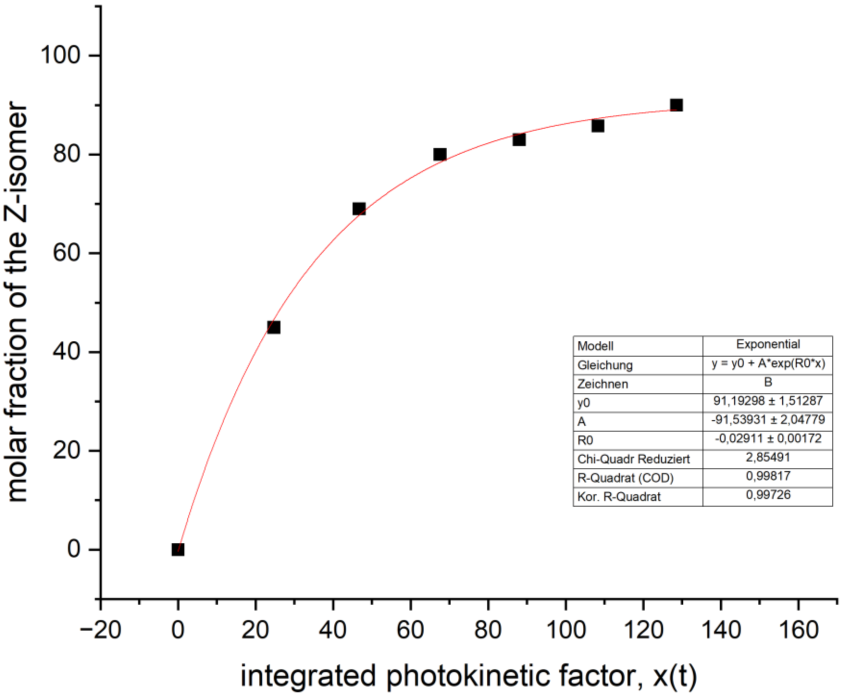

(*E*/*Z*)-Methyl-3-chloro-4-((2-chloro-6-fluorophenyl)diazenyl)-5-fluorobenzoate (21)

$\Phi_{E\rightarrow Z} = 0.00395$

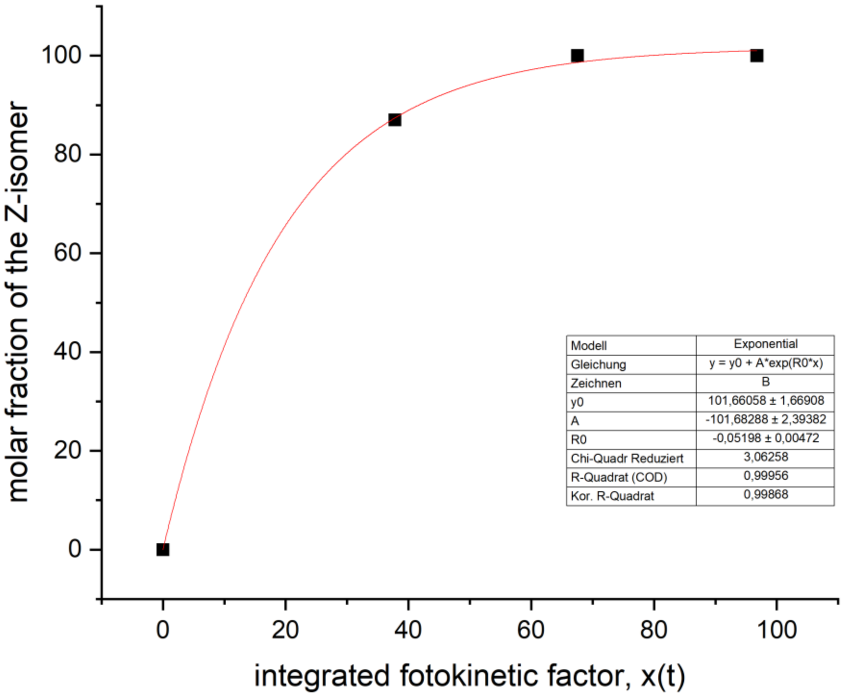

(*E*/*Z*)-*N*-(3-Chloro-4-((2-chloro-6-fluorophenyl)diazenyl)-5-fluorophenyl)acetamide (39)

$\Phi_{E\rightarrow Z} = 0.00215$

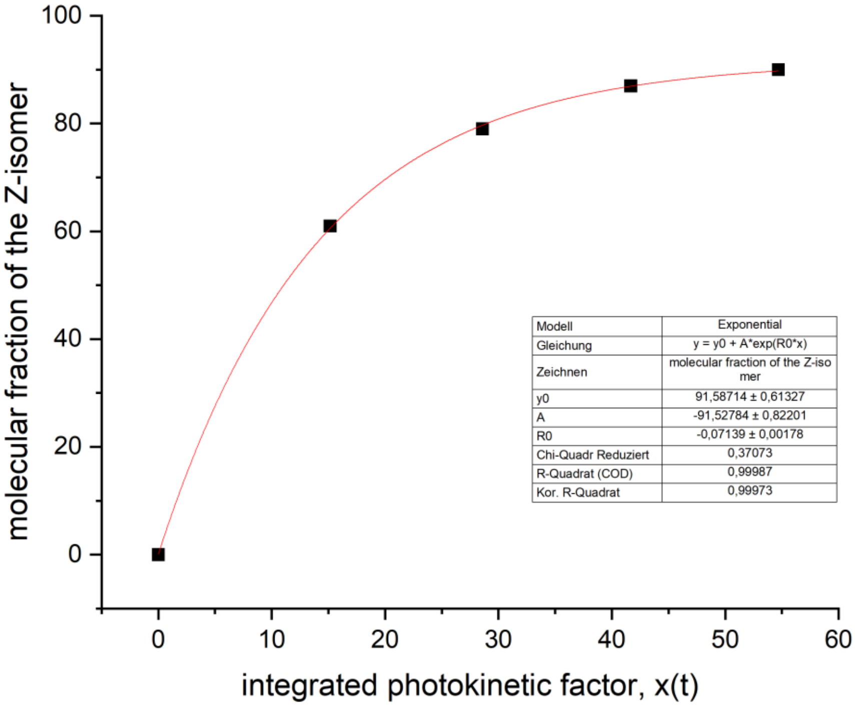

(*E*/*Z*)-Methyl-3-chloro-4-((2-chloro-6-fluoro-4-methoxyphenyl)diazenyl)-5-fluorobenzoate (46)

$\Phi_{E \rightarrow Z} = 0.0114$

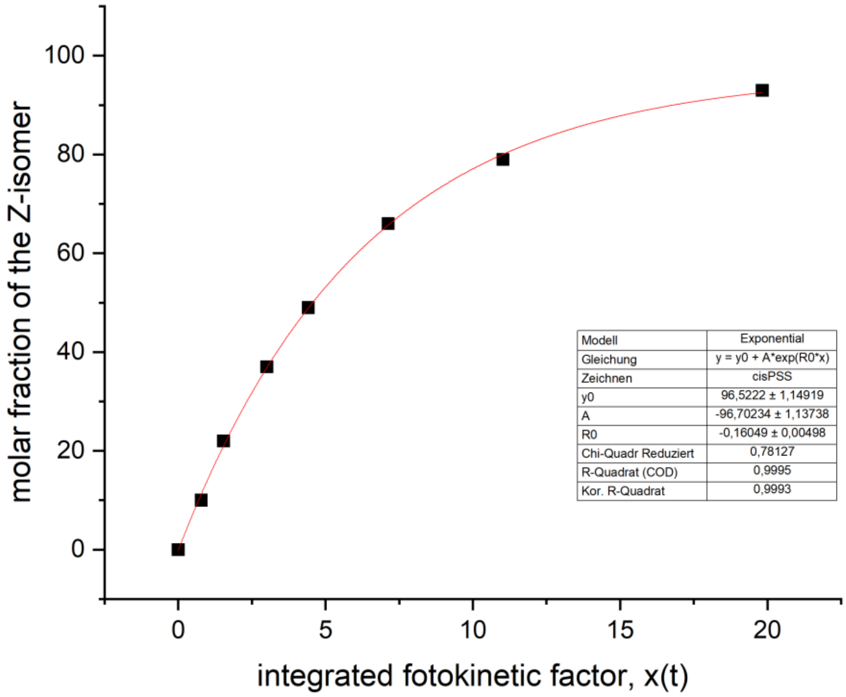

8. X-Ray Data

(*Cis*)-1,2-bis(2,6-difluorophenyl)diazene ((*Z*)—2, CCDC 2387092)

Table S2.1 Details for X-ray data collection and structure refinement for compound ***cis*-2.**

	***cis*-2**
Empirical formula	$C_{12}H_6F_4N_2$
Formula mass	254.19
T[K]	123(2)
Crystal size [mm]	0.30 × 0.20 × 0.15
Crystal description	orange yellow block
Crystal system	orthorhombic
Space group	*Pna*21
a [Å]	13.8935(3)
b [Å]	11.5390(2)
c [Å]	6.56050(10)
α [°]	90.0
β [°]	90.0
γ [°]	90.0
V [$Å^3$]	1051.76(3)
Z	4
$\rho_{calcd.}$ [g cm^{-3}]	1.605
μ [mm^{-1}]	0.147
F(000)	512
Θ range [°]	2.93 – 25.24
Index ranges	$-19 \leq h \leq 19$
	$-16 \leq k \leq 16$
	$-9 \leq l \leq 9$
Reflns. collected	20324
Reflns. obsd.	2987
Reflns. unique	3211 (R_{int} = 0.0247)
R_1, wR_2 (2σ data)	0.0292, 0.0709
R_1, wR_2 (all data)	0.0327, 0.0735
GOOF on F^2	1.032
Peak/hole [e $Å^{-3}$]	0.219 / -0.130

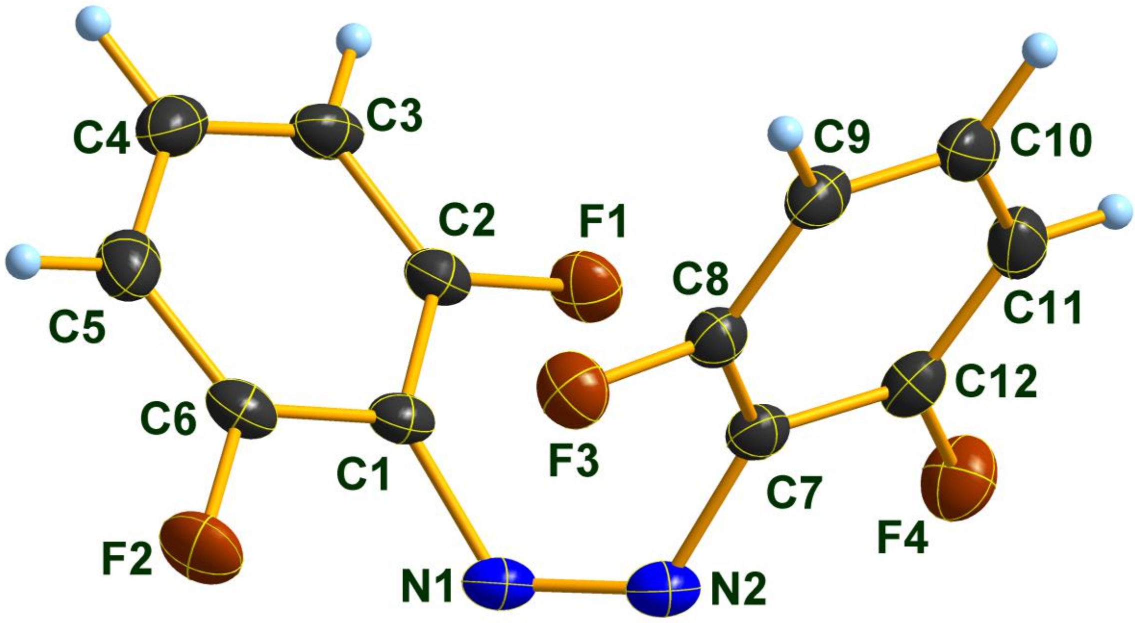

Figure S2.1: Molecular structure of compound ***cis*-2** in the crystal. DIAMOND[e)] representation; thermal ellipsoids are drawn at 50 % probability level.

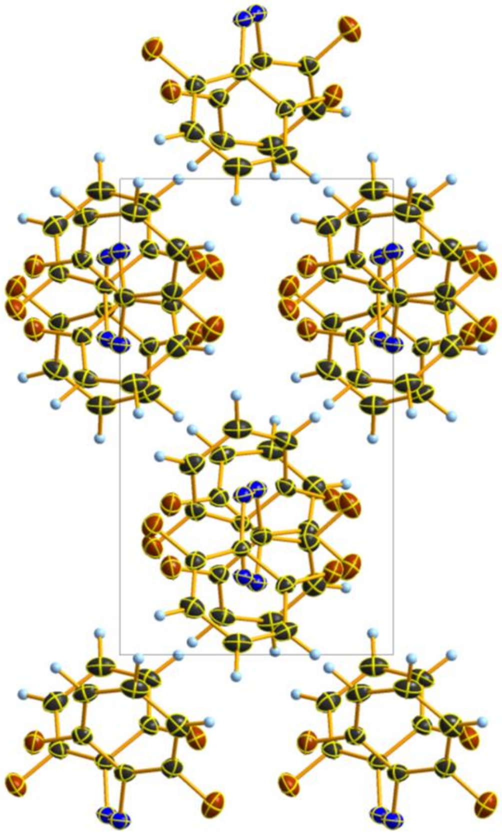

Figure S2.2 Crystal structure of compound ***cis*-2**, view of the unit cell along the *a*-axis. DIAMOND[e)] representation; thermal ellipsoids are drawn at 50 % probability level.

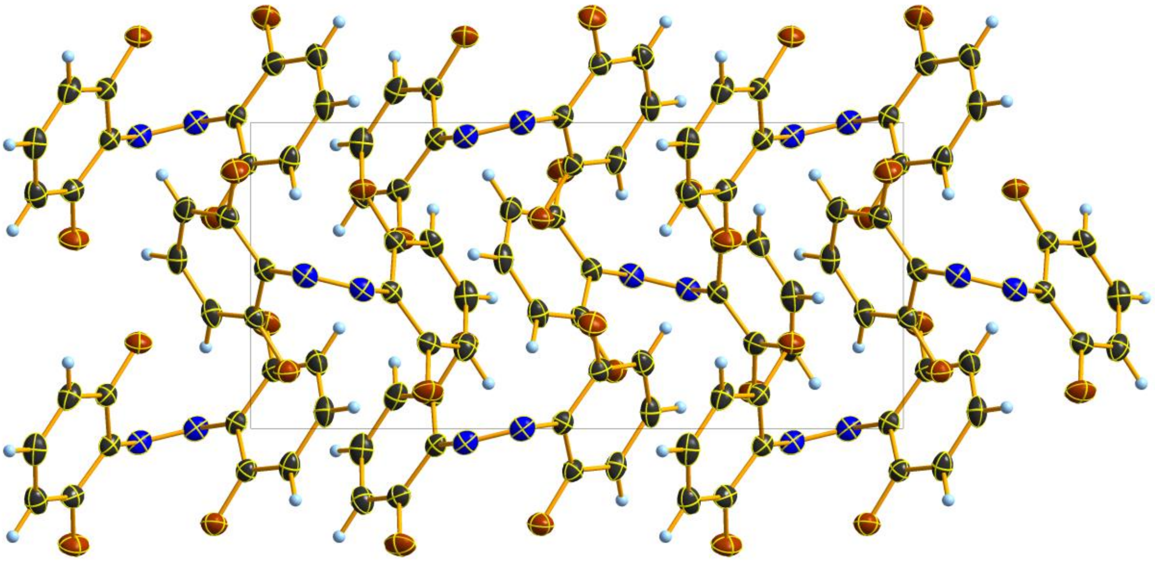

Figure S2.3 Crystal structure of compound ***cis*-2**, view of the unit cell along the *b*-axis. DIAMOND[e)] representation; thermal ellipsoids are drawn at 50 % probability level.

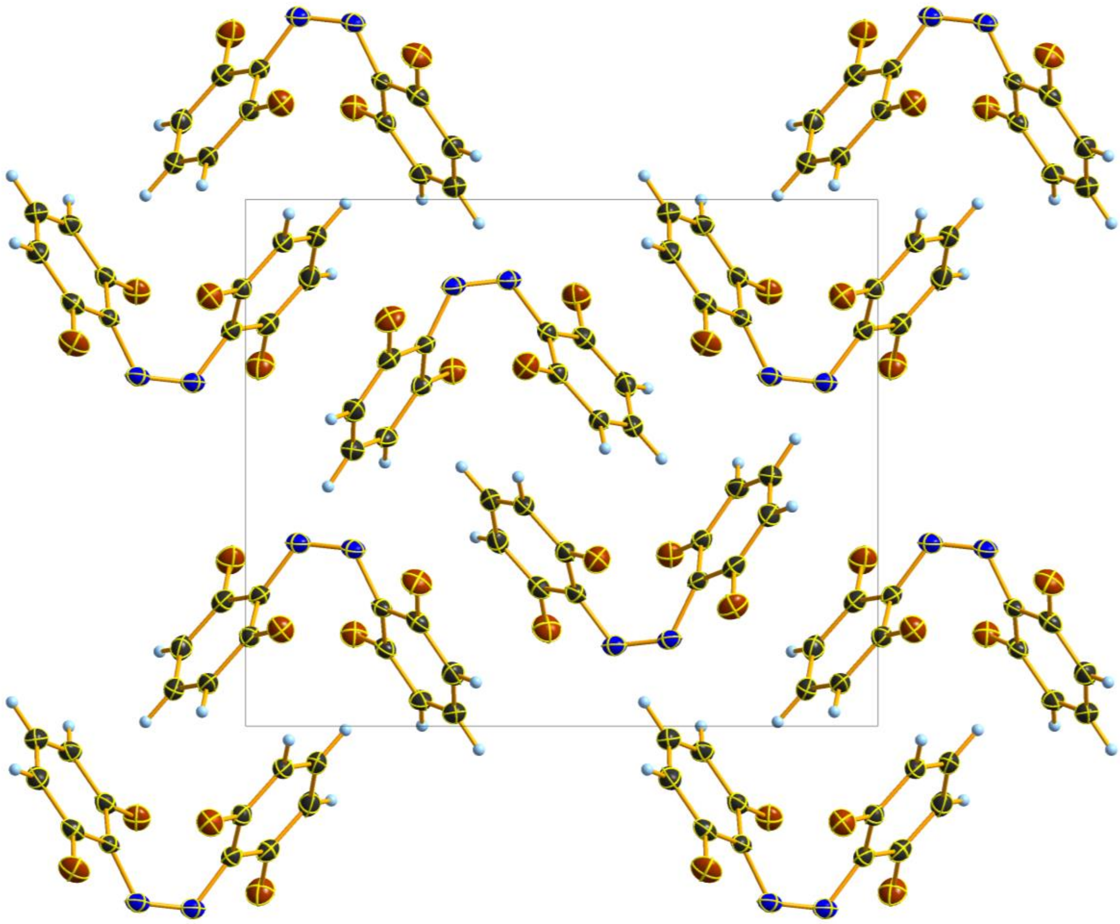

Figure S2.4 Crystal structure of compound ***cis*-2**, view of the unit cell along the *c*-axis. DIAMOND[e)] representation; thermal ellipsoids are drawn at 50 % probability level.

Table S2.2. Selected bond lengths (Å) of compound ***cis*-2.**

C1 – C2	1.388(2)	C4 – H4	0.97(2)
C1 – C6	1.393(2)	C5 – C6	1.374(2)
C1 – N1	1.4388(19)	C5 – H5	0.94(3)
F2 – C6	1.3503(18)	C7 – C8	1.387(2)
F1 – C2	1.353(2)	C7 – C12	1.390(2)
N1 – N2	1.250(2)	C8 – C9	1.378(2)
N2 – C7	1.438(2)	C9 – C10	1.387(3)
C2 – C3	1.379(2)	C9 – H9	0.96(2)
F3 – C8	1.350(2)	C12 – C11	1.380(3)
C3 – C4	1.382(3)	C10 – C11	1.385(3)
C3 – H3	0.91(2)	C10 – H10	0.96(2)
F4 – C12	1.344(2)	C11 – H11	0.89(3)
C4 – C5	1.390(3)		

Table S2.3. Selected bond angles (°) of compound ***cis*-2.**

C2 – C1 – C6	116.0(1)	C5 – C6 – C1	123.2(2)
C2 – C1 – N1	124.3(2)	C8 – C7 – C12	116.9(1)
C6 – C1 – N1	118.7(1)	C8 – C7 – N2	123.4(2)
N2 – N1 – C1	121.9(1)	C12 – C7 – N2	119.1(1)
N1 – N2 – C7	120.8(1)	F3 – C8 – C9	119.1(2)
F1 – C2 – C3	119.4(2)	F3 – C8 – C7	118.0(1)
F1 – C2 – C1	117.9(1)	C9 – C8 – C7	122.8(2)
C3 – C2 – C1	122.7(2)	C8 – C9 – C10	118.2(2)
C2 – C3 – C4	118.9(2)	F4 – C12 – C11	120.1(2)
C3 – C4 – C5	120.7(2)	F4 – C12 – C7	117.7(2)
C6 – C5 – C4	118.3(2)	C11 – C12 – C7	122.2(2)
F2 – C6 – C5	119.8(2)	C11 – C10 – C9	121.1(2)
F2 – C6 – C1	116.9(1)	C12 – C11 – C10	118.7(2)

Table S2.4. Selected torsion angles (°) of compound ***cis*-2.**

C2 – C1 – N1 – N2	59.1(2)	N1 – N2 – C7 – C8	58.5(2)
C6 – C1 – N1 – N2	-133.0(2)	N1 – N2 – C7 – C12	-130.4(2)
C1 – N1 – N2 – C7	8.2(2)	C12 – C7 – C8 – F3	-175.2(1)
C6 – C1 – C2 – F1	-175.9(1)	N2 – C7 – C8 – F3	-3.9(2)
N1 – C1 – C2 – F1	-7.7(2)	C12 – C7 – C8 – C9	2.7(2)
C6 – C1 – C2 – C3	1.8(2)	N2 – C7 – C8 – C9	173.9(2)
N1 – C1 – C2 – C3	170.0(1)	F3 – C8 – C9 – C10	177.5(1)
F1 – C2 – C3 – C4	177.8(2)	C7 – C8 – C9 – C10	-0.3(2)
C1 – C2 – C3 – C4	0.1(2)	C8 – C7 – C12 – F4	177.7(1)
C2 – C3 – C4 – C5	-1.0(3)	N2 – C7 – C12 – F4	6.1(2)
C3 – C4 – C5 – C6	-0.1(3)	C8 – C7 – C12 – C11	-3.6(2)
C4 – C5 – C6 – F2	-178.9(2)	N2 – C7 – C12 – C11	-175.3(2)
C4 – C5 – C6 – C1	2.3(3)	C8 – C9 – C10 – C11	-1.2(2)
C2 – C1 – C6 – F2	178.0(1)	F4 – C12 – C11 – C10	-179.2(2)
N1 – C1 – C6 – F2	9.1(2)	C7 – C12 – C11 – C10	2.2(2)
C2 – C1 – C6 – C5	-3.1(2)	C9 – C10 – C11 – C12	0.3(2)
N1 – C1 – C6 – C5	-172.0(2)		

Cis-1,2-bis(2,6-dichlorophenyl)diazene (*cis*-6, CCDC 2387095)

Table S2.5 Details for X-ray data collection and structure refinement for compound ***cis*-6.**

	***cis*-6**
Empirical formula	$C_{12}H_6Cl_4N_2$
Formula mass	319.99
T[K]	123(2)
Crystal size [mm]	0.40 × 0.10 × 0.03
Crystal description	orange red needle
Crystal system	monoclinic
Space group	*P*21/*c*
a [Å]	10.9158(4)
b [Å]	7.3957(3)
c [Å]	16.2571(6)
α [°]	90.0
β [°]	107.751(4)
γ [°]	90.0
V [Å^3]	1249.95(9)
Z	4
$\rho_{calcd.}$ [g cm^{-3}]	1.700
μ [mm^{-1}]	0.926
F(000)	640
Θ range [°]	2.63 – 25.24
Index ranges	$-15 \leq h \leq 15$
	$-10 \leq k \leq 10$
	$-23 \leq l \leq 23$
Reflns. collected	24231
Reflns. obsd.	3174
Reflns. unique	3823 (R_{int} = 0.0390)
R_1, wR_2 (2σ data)	0.0356, 0.0861
R_1, wR_2 (all data)	0.0471, 0.0927
GOOF on F^2	1.066
Peak/hole [e Å^{-3}]	0.547 / -0.283

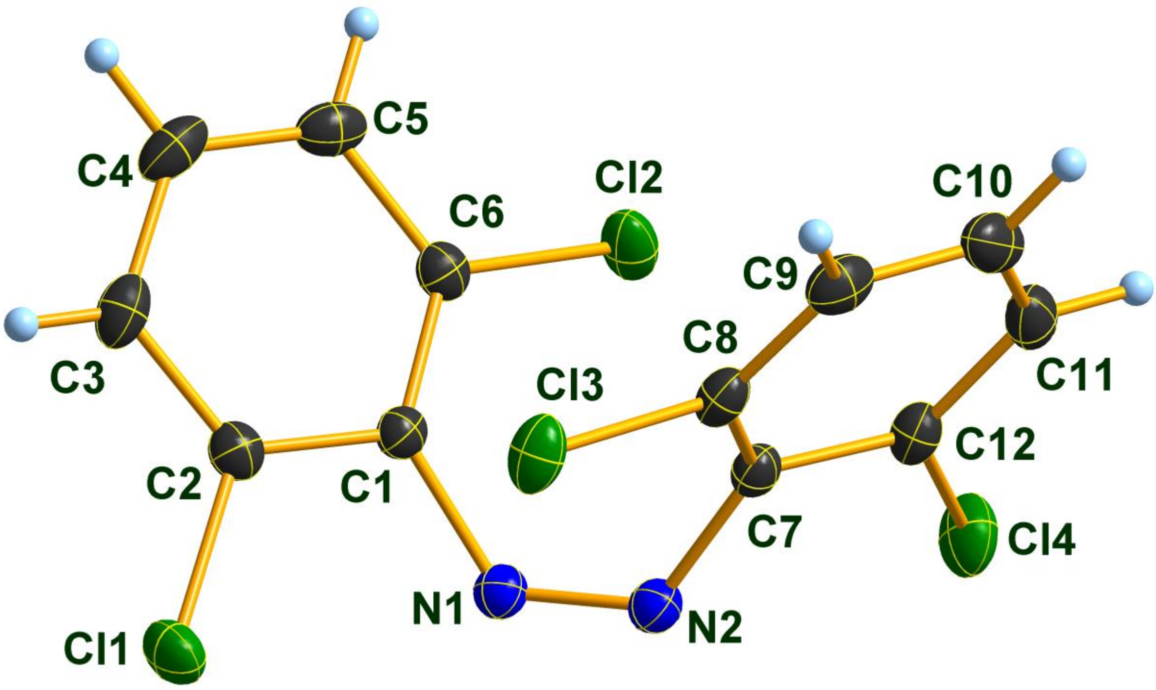

Figure S2.5 Molecular structure of compound ***cis*-6** in the crystal. DIAMOND[e)] representation; thermal ellipsoids are drawn at 50 % probability level.

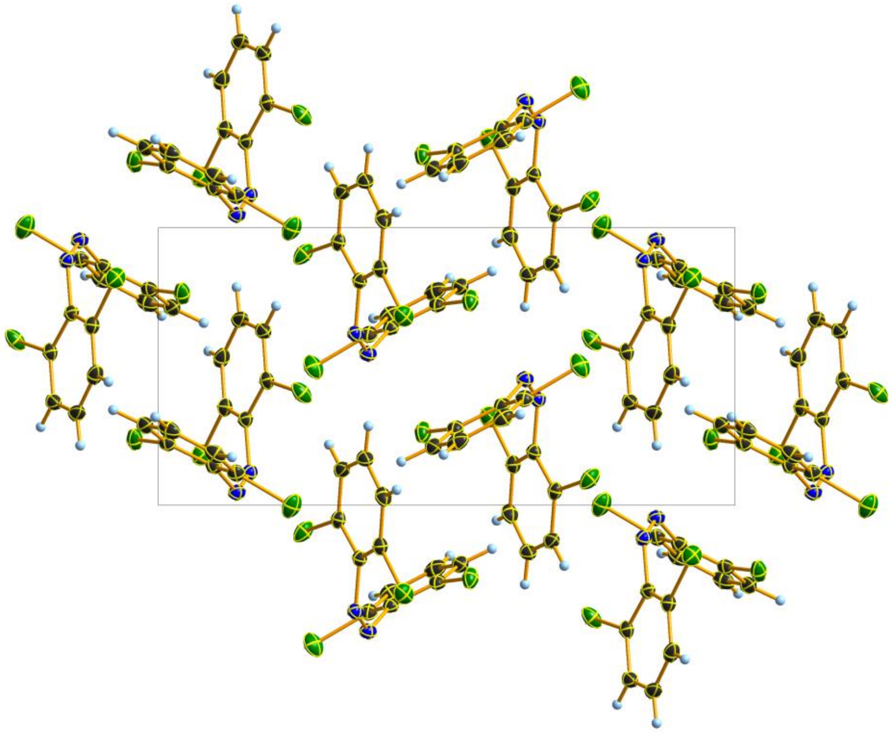

Figure S2.6 Crystal structure of compound ***cis*-6**, view of the unit cell along the *a*-axis. DIAMOND[e)] representation; thermal ellipsoids are drawn at 50 % probability level.

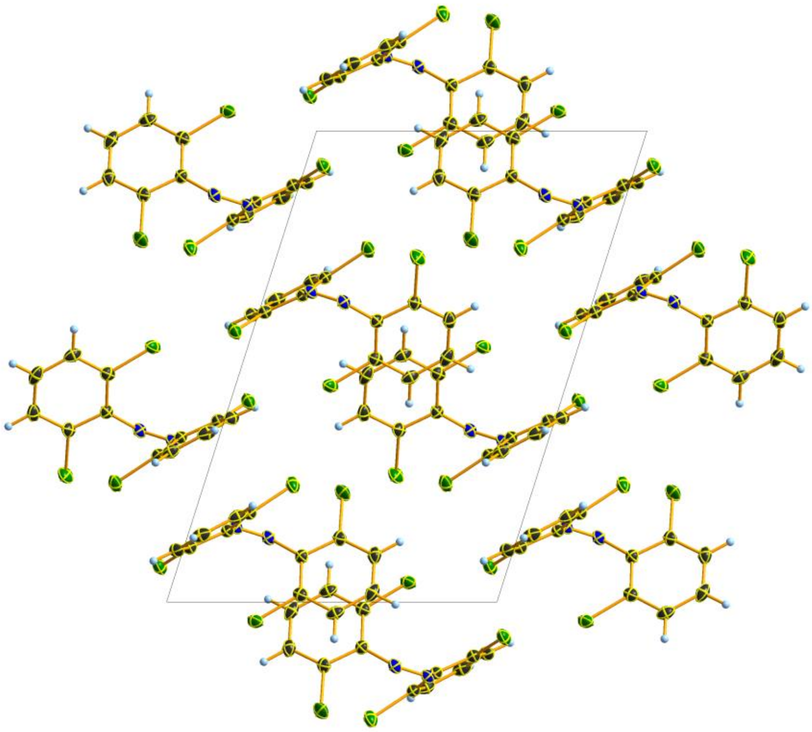

Figure S2.7 Crystal structure of compound ***cis*-6**, view of the unit cell along the *b*-axis. DIAMOND[e)] representation; thermal ellipsoids are drawn at 50 % probability level.

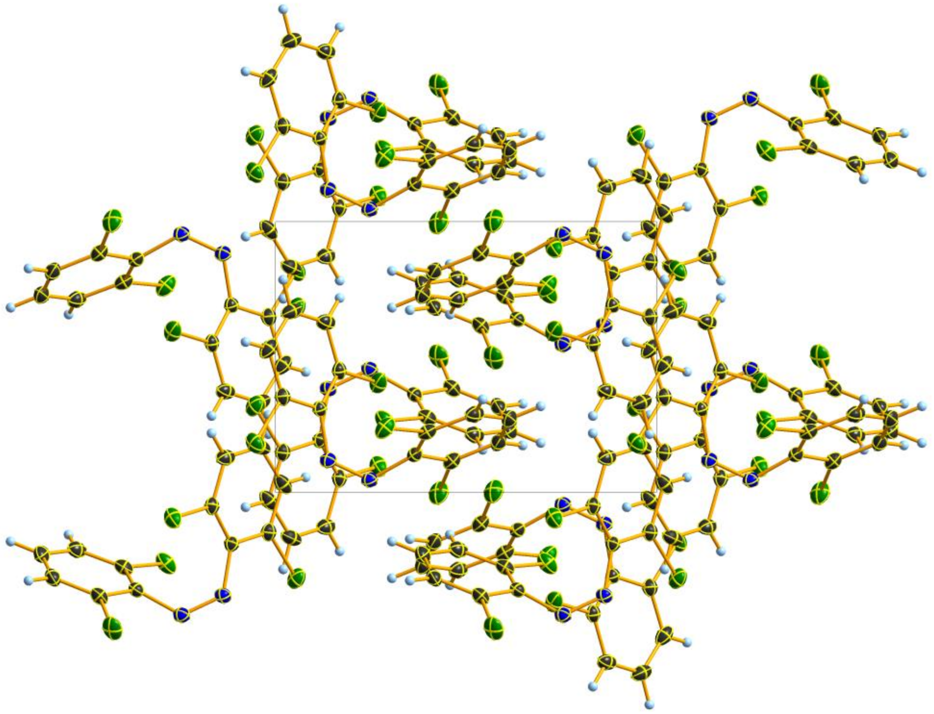

Figure S2.8 Crystal structure of compound ***cis*-6**, view of the unit cell along the *c*-axis. DIAMOND[e)] representation; thermal ellipsoids are drawn at 50 % probability level.

Table S2.6. Selected bond lengths (Å) of compound ***cis*-6.**

N1 – N2	1.247(2)	C5 – C6	1.393(2)
N1 – C1	1.440(2)	C7 – C8	1.395(2)
C1 – C2	1.399(2)	C7 – C12	1.400(2)
C1 – C6	1.400(2)	C8 – C9	1.387(3)
Cl2 – C6	1.729(2)	C9 – C10	1.388(3)
Cl1 – C2	1.731(2)	C10 – C11	1.386(3)
N2 – C7	1.437(2)	C11 – C12	1.381(3)
C2 – C3	1.386(2)	Cl4 – C12	1.730(2)
Cl3 – C8	1.735(2)	C4 – C5	1.377(3)
C3 – C4	1.391(3)		

Table S2.7. Selected bond angles (°) of compound ***cis*-6.**

N2 – N1 – C1	124.1(1)	C1 – C6 – Cl2	121.0(1)
C2 – C1 – C6	117.2(2)	C8 – C7 – C12	117.7(2)
C2 – C1 – N1	115.5(1)	C8 – C7 – N2	125.2(2)
C6 – C1 – N1	126.4(2)	C12 – C7 – N2	116.1(2)
N1 – N2 – C7	124.9(1)	C9 – C8 – C7	121.2(2)
C3 – C2 – C1	121.7(2)	C9 – C8 – Cl3	118.3(1)
C3 – C2 – Cl1	119.4(1)	C7 – C8 – Cl3	120.4(1)
C1 – C2 – Cl1	118.9(1)	C8 – C9 – C10	119.4(2)
C2 – C3 – C4	119.4(2)	C11 – C10 – C9	120.7(2)
C5 – C4 – C3	120.4(2)	C12 – C11 – C10	119.1(2)
C4 – C5 – C6	119.7(2)	C11 – C12 – C7	121.7(2)
C5 – C6 – C1	121.3(2)	C11 – C12 – Cl4	119.5(1)
C5 – C6 – Cl2	117.6(1)	C7 – C12 – Cl4	118.9(1)

Table S2.8 Selected torsion angles (°) of compound ***cis*-6.**

N2 – N1 – C1 – C2	-133.8(2)	N1 – N2 – C7 – C8	63.6(2)
N2 – N1 – C1 – C6	57.1(2)	N1 – N2 – C7 – C12	-128.5(2)
C1 – N1 – N2 – C7	3.4(3)	C12 – C7 – C8 – C9	3.1(2)
C6 – C1 – C2 – C3	-5.5(2)	N2 – C7 – C8 – C9	170.8(2)
N1 – C1 – C2 – C3	-175.7(2)	C12 – C7 – C8 – Cl3	-173.6(1)
C6 – C1 – C2 – Cl1	176.2(1)	N2 – C7 – C8 – Cl3	-5.8(2)
N1 – C1 – C2 – Cl1	6.0(2)	C7 – C8 – C9 – C10	0.6(3)
C1 – C2 – C3 – C4	1.0(3)	Cl3 – C8 – C9 – C10	177.3(1)
Cl1 – C2 – C3 – C4	179.3(1)	C8 – C9 – C10 – C11	-2.3(3)
C2 – C3 – C4 – C5	3.1(3)	C9 – C10 – C11 – C12	0.2(3)
C3 – C4 – C5 – C6	-2.3(3)	C10 – C11 – C12 – C7	3.7(3)
C4 – C5 – C6 – C1	-2.5(3)	C10 – C11 – C12 – Cl4	-176.7(1)
C4 – C5 – C6 – Cl2	173.8(1)	C8 – C7 – C12 – C11	-5.3(3)
C2 – C1 – C6 – C5	6.3(2)	N2 – C7 – C12 – C11	-174.1(2)
N1 – C1 – C6 – C5	175.3(2)	C8 – C7 – C12 – Cl4	175.1(1)
C2 – C1 – C6 – Cl2	-169.9(1)	N2 – C7 – C12 – Cl4	6.3(2)
N1 – C1 – C6 – Cl2	-0.9(2)		

***Trans*-1-(2-chloro-6-fluoro-4-nitrophenyl)-2-(2-chloro-6-fluorophenyl)diazene (*trans*-19, CCDC 2387091)**

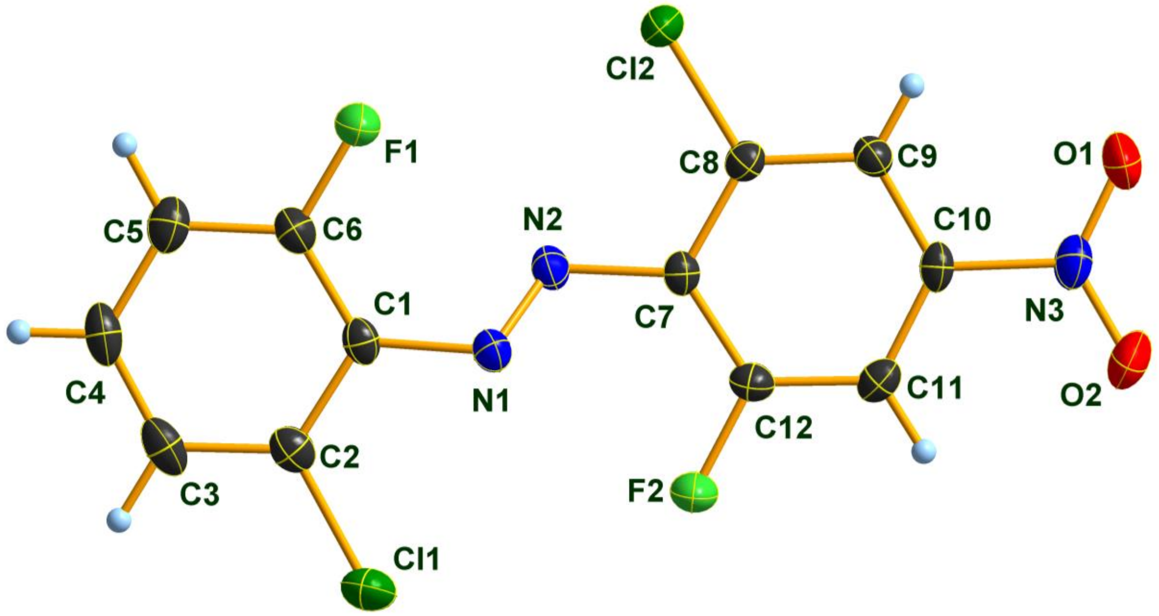

Figure S2.9 Molecular structure of *trans*-1-(2-chloro-6-fluoro-4-nitrophenyl)-2-(2-chloro-6-fluorophenyl)diazene (***trans*-19**) in the crystal. DIAMOND[g)] representation; thermal ellipsoids are drawn at 50 % probability level.

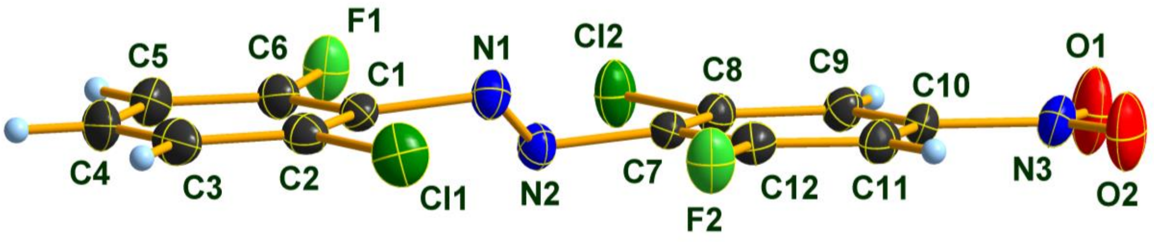

Figure S2.10 Molecular structure of *trans*-1-(2-chloro-6-fluoro-4-nitrophenyl)-2-(2-chloro-6-fluorophenyl)diazene (***trans*-19**) in the crystal, side view. DIAMOND[g)] representation; thermal ellipsoids are drawn at 50 % probability level.

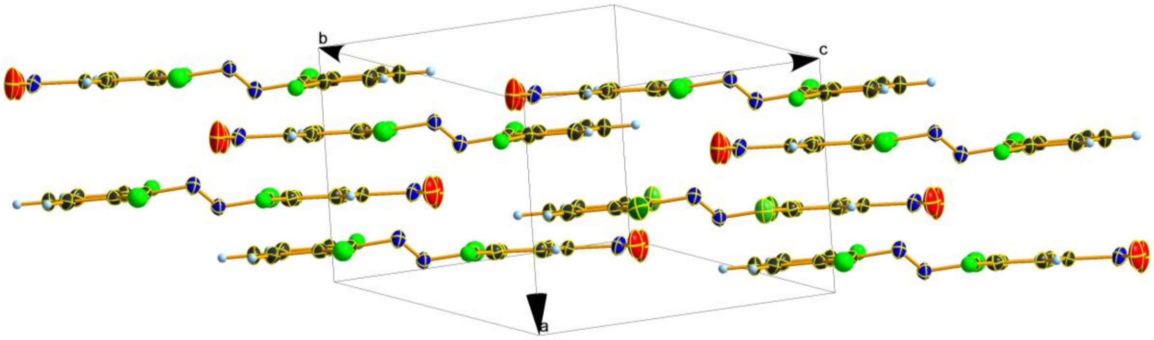

Figure S2.11 Crystal structure of *trans*-1-(2-chloro-6-fluoro-4-nitrophenyl)-2-(2-chloro-6-fluorophenyl)diazene (***trans*-19**); view of the unit cell. DIAMOND[g)] representation; thermal ellipsoids are drawn at 50 % probability level.

Table S2.9. Details for X-ray data collection and structure refinement *trans*-1-(2-chloro-6-fluoro-4-nitrophenyl)-2-(2-chloro-6-fluorophenyl)diazene (***trans*-19**).

	**19**
Empirical formula	$C_{12}H_5Cl_2F_2N_3O_2$
Formula mass	332.09
T[K]	173(2)
Crystal size [mm]	0.18 × 0.03 × 0.02
Crystal description	yellow needle
Crystal system	triclinic
Space group	*P*-1
a [Å]	6.9311(5)
b [Å]	8.8971(6)
c [Å]	10.9455(8)
α [°]	92.506(3)
β [°]	98.159(3)
γ [°]	108.060(2)
V [$Å^3$]	632.46(8)
Z	2
$\rho_{calcd.}$ [g cm^{-3}]	1.744
μ [mm^{-1}]	0.545
F(000)	332
Θ range [°]	2.92 – 25.24
Index ranges	$-8 \leq h \leq 8$
	$-11 \leq k \leq 11$
	$-14 \leq l \leq 14$
Reflns. collected	11474
Reflns. obsd.	2292
Reflns. unique	2846 (R_{int} = 0.0414)
R_1, wR_2 (2σ data)	0.0401, 0.0822
R_1, wR_2 (all data)	0.0550, 0.0882
GOOF on F^2	1.078
Peak/hole [e $Å^{-3}$]	0.297 / -0.218

Table S2.10. Selected bond lengths (Å) of *trans*-1-(2-chloro-6-fluoro-4-nitrophenyl)-2-(2-chloro-6-fluorophenyl)diazene (***trans*-19**).

Cl1 – C2	1.727(2)	C4 – C5	1.385(3)
F1 – C6	1.350(2)	C5 – C6	1.374(3)
O1 – N3	1.217(2)	C7 – C8	1.396(3)
N1 – N2	1.239(2)	C7 – C12	1.402(3)
N1 – C1	1.431(2)	C9 – C10	1.380(3)
C1 – C6	1.394(3)	C9 – C8	1.383(3)
C1 – C2	1.395(3)	C11 – C12	1.374(3)
F2 – C12	1.348(2)	C11 – C10	1.377(3)
Cl2 – C8	1.717(2)	C2 – C3	1.391(3)
O2 – N3	1.217(2)	N3 – C10	1.474(2)
N2 – C7	1.430(2)	C3 – C4	1.380(3)

Table S2.11 Selected bond angles (°) of *trans*-1-(2-chloro-6-fluoro-4-nitrophenyl)-2-(2-chloro-6-fluorophenyl)diazene (***trans*-19**).

N2 – N1 – C1	112.8(2)	F1 – C6 – C1	118.8(2)
C6 – C1 – C2	116.9(2)	C5 – C6 – C1	122.9(2)
C6 – C1 – N1	123.8(2)	C8 – C7 – C12	117.5(2)
C2 – C1 – N1	118.9(2)	C8 – C7 – N2	117.6(2)
N1 – N2 – C7	114.5(2)	C12 – C7 – N2	124.5(2)
C3 – C2 – C1	121.4(2)	C10 – C9 – C8	117.7(2)
C3 – C2 – Cl1	119.4(2)	C9 – C8 – C7	121.8(2)
C1 – C2 – Cl1	119.2(2)	C9 – C8 – Cl2	119.0(2)
O1 – N3 – O2	124.3(2)	C7 – C8 – Cl2	119.1(1)
O1 – N3 – C10	117.7 (2)	C12 – C11 – C10	117.8(2)
O2 – N3 – C10	118.0(2)	C11 – C10 – C9	123.1(2)
C4 – C3 – C2	119.1(2)	C11 – C10 – N3	119.0(2)
C3 – C4 – C5	121.1(2)	C9 – C10 – N3	117.9(2)
C6 – C5 – C4	118.4(2)	F2 – C12 – C11	118.6(2)
F1 – C6 – C5	118.2(2)	F2 – C12 – C7	119.4(2)
C11 – C12 – C7	122.1(2)		

Table S2.12 Selected torsion angles (°) of *trans*-1-(2-chloro-6-fluoro-4-nitrophenyl)-2-(2-chloro-6-fluorophenyl)diazene (***trans*-19**).

N2 – N1 – C1 – C6	-44.8(3)	C10 – C9 – C8 – Cl2	179.0(2)
N2 – N1 – C1 – C2	141.6(2)	C12 – C7 – C8 – C9	-0.8(3)
C1 – N1 – N2 – C7	179.3(2)	N2 – C7 – C8 – C9	-173.9(2)
C6 – C1 – C2 – C3	1.2(3)	C12 – C7 – C8 – Cl2	-178.5(1)
N1 – C1 – C2 – C3	175.3(2)	N2 – C7 – C8 – Cl2	8.4(2)
C6 – C1 – C2 – Cl1	179.5(2)	C12 – C11 – C10 – C9	-0.4(3)
N1 – C1 – C2 – Cl1	-6.5(2)	C12 – C11 – C10 – N3	179.9(2)
C1 – C2 – C3 – C4	-1.3(3)	C8 – C9 – C10 – C11	-0.6(3)
Cl1 – C2 – C3 – C4	-179.6(2)	C8 – C9 – C10 – N3	179.0(2)
C2 – C3 – C4 – C5	0.4(3)	O1 – N3 – C10 – C11	177.2(2)
C3 – C4 – C5 – C6	0.6(3)	O2 – N3 – C10 – C11	-1.8(3)
C4 – C5 – C6 – F1	-177.3(2)	O1 – N3 – C10 – C9	-2.5(3)
C4 – C5 – C6 – C1	-0.7(3)	O2 – N3 – C10 – C9	178.5(2)
C2 – C1 – C6 – F1	176.4(2)	C10 – C11 – C12 – F2	179.9(2)
N1 – C1 – C6 – F1	2.6(3)	C10 – C11 – C12 – C7	1.0(3)
C2 – C1 – C6 – C5	-0.2(3)	C8 – C7 – C12 – F2	-179.3(2)
N1 – C1 – C6 – C5	-174.0(2)	N2 – C7 – C12 – F2	-6.7(3)
N1 – N2 – C7 – C8	-143.5(2)	C8 – C7 – C12 – C11	-0.4(3)
N1 – N2 – C7 – C12	44.0(3)	N2 – C7 – C12 – C11	172.2(2)
C10 – C9 – C8 – C7	1.3(3)		

Cis-methyl-3-chloro-4-((2-chloro-6-fluorophenyl)diazenyl)-5-fluorobenzoate (c_is_-21, CCDC 2387093)

Table S2.13 Details for X-ray data collection and structure refinement for compound ***cis*-21**.

***cis*-21**	
Empirical formula	$C_{14}H_8Cl_2F_2N_2O_2$
Formula mass	345.12
T[K]	123(2)
Crystal size [mm]	0.30 × 0.10 × 0.10
Crystal description	orange block
Crystal system	orthorhombic
Space group	*P*212121
a [Å]	7.0603(2)
b [Å]	13.0920(3)
c [Å]	15.2661(4)
α [°]	90.0
β [°]	90.0
γ [°]	90.0
V [Å^3]	1411.10(6)
Z	4
$\rho_{calcd.}$ [g cm^{-3}]	1.625
μ [mm^{-1}]	0.490
F(000)	696
Θ range [°]	2.05 – 25.24
Index ranges	-10 ≤ *h* ≤ 10
	-18 ≤ *k* ≤ 18
	-21 ≤ *l* ≤ 21
Reflns. collected	28683
Reflns. obsd.	3934
Reflns. unique	4299 (R_{int} = 0.0428)
R_1, wR_2 (2σ data)	0.0306, 0.0703
R_1, wR_2 (all data)	0.0357, 0.0731
GOOF on F^2	1.031
Peak/hole [e Å^{-3}]	0.321 / -0.151

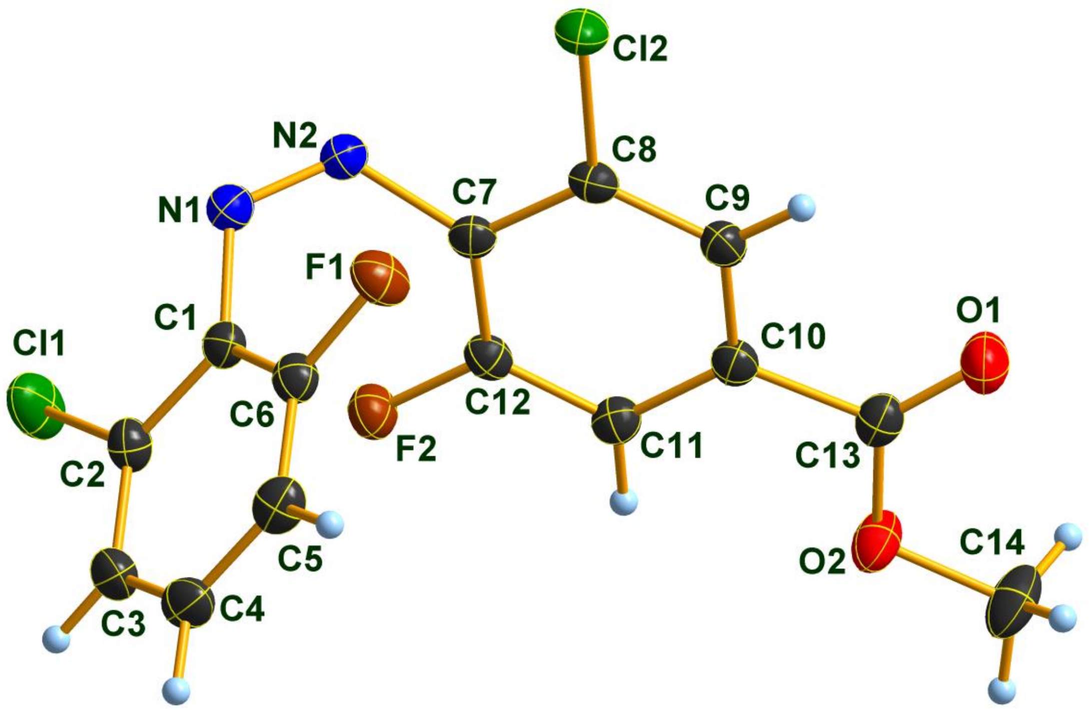

Figure S2.12 Molecular structure of compound ***cis*-21** in the crystal. DIAMOND[e)] representation; thermal ellipsoids are drawn at 50 % probability level.

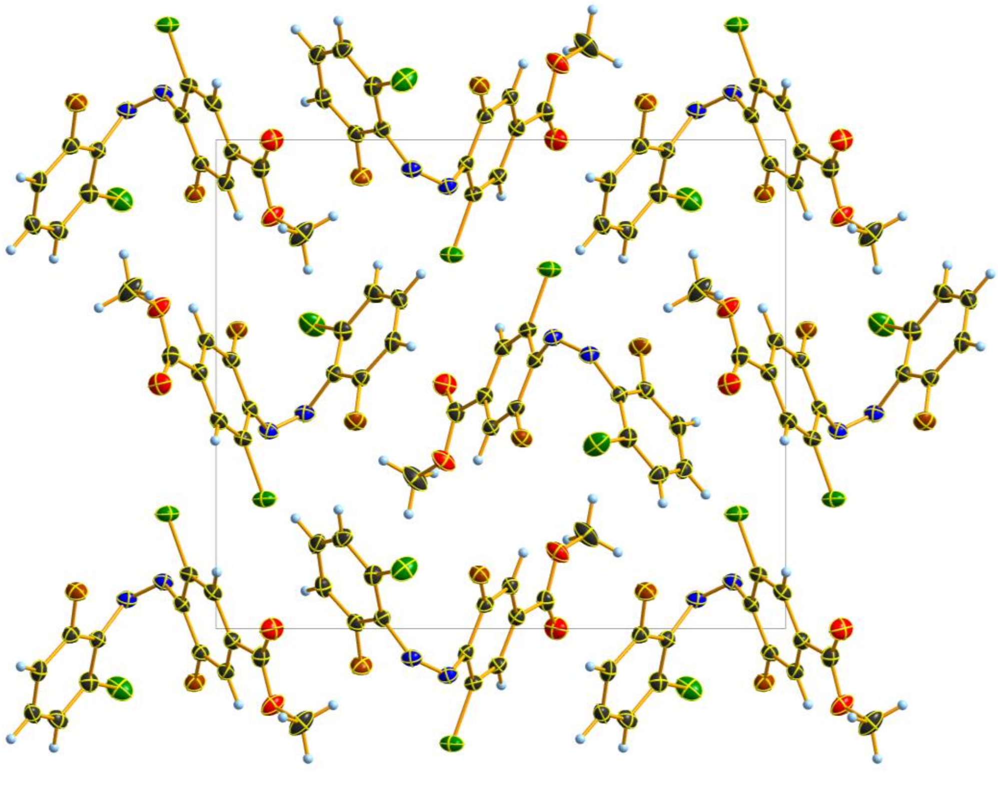

Figure S2.13 Crystal structure of compound ***cis*-21**. View of the unit cell along the *a*-axis. DIAMOND[e)] representation; thermal ellipsoids are drawn at 50 % probability level.

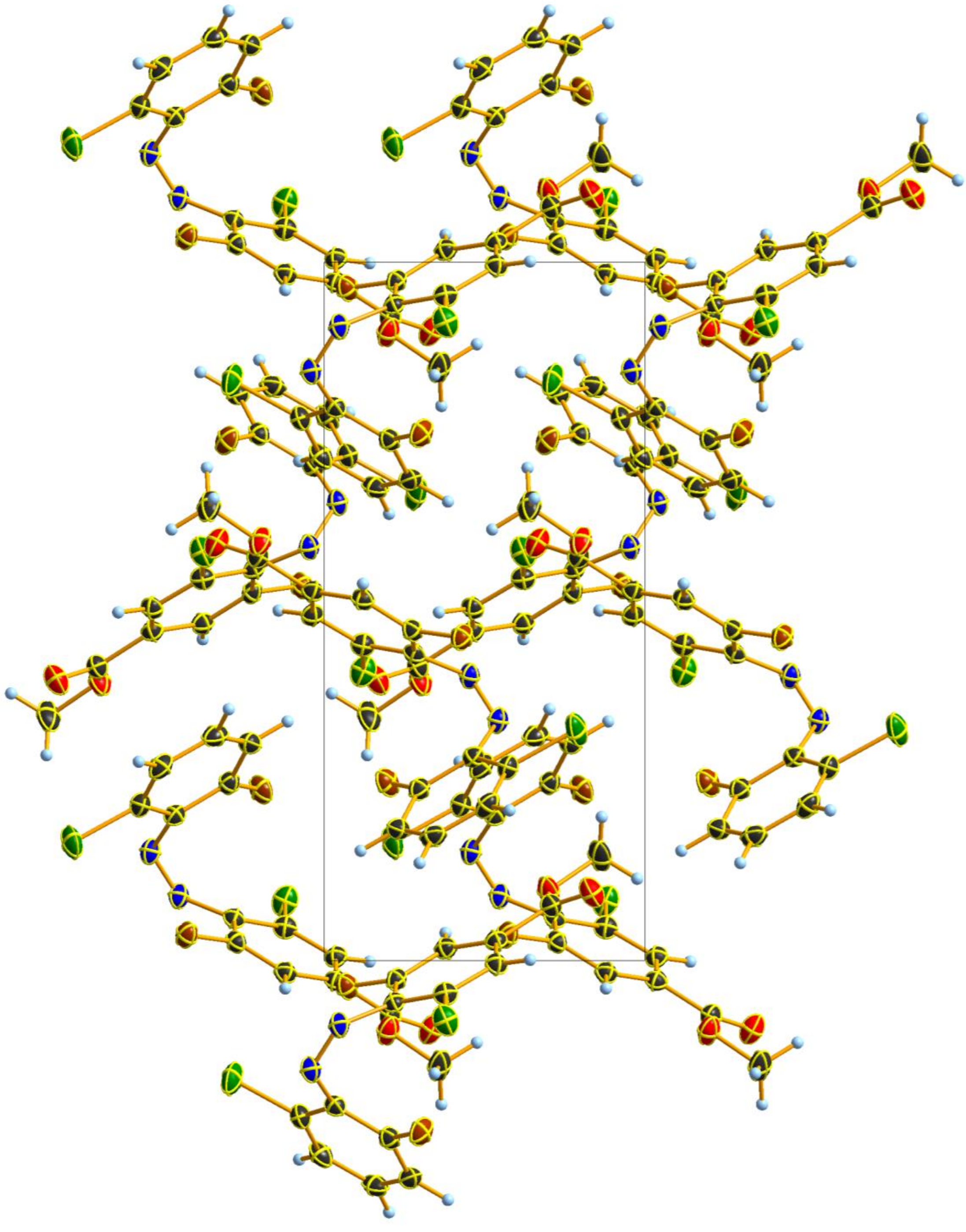

Figure S2.14 Crystal structure of compound ***cis*-21**. View of the unit cell along the *b*-axis. DIAMOND[e)] representation; thermal ellipsoids are drawn at 50 % probability level.

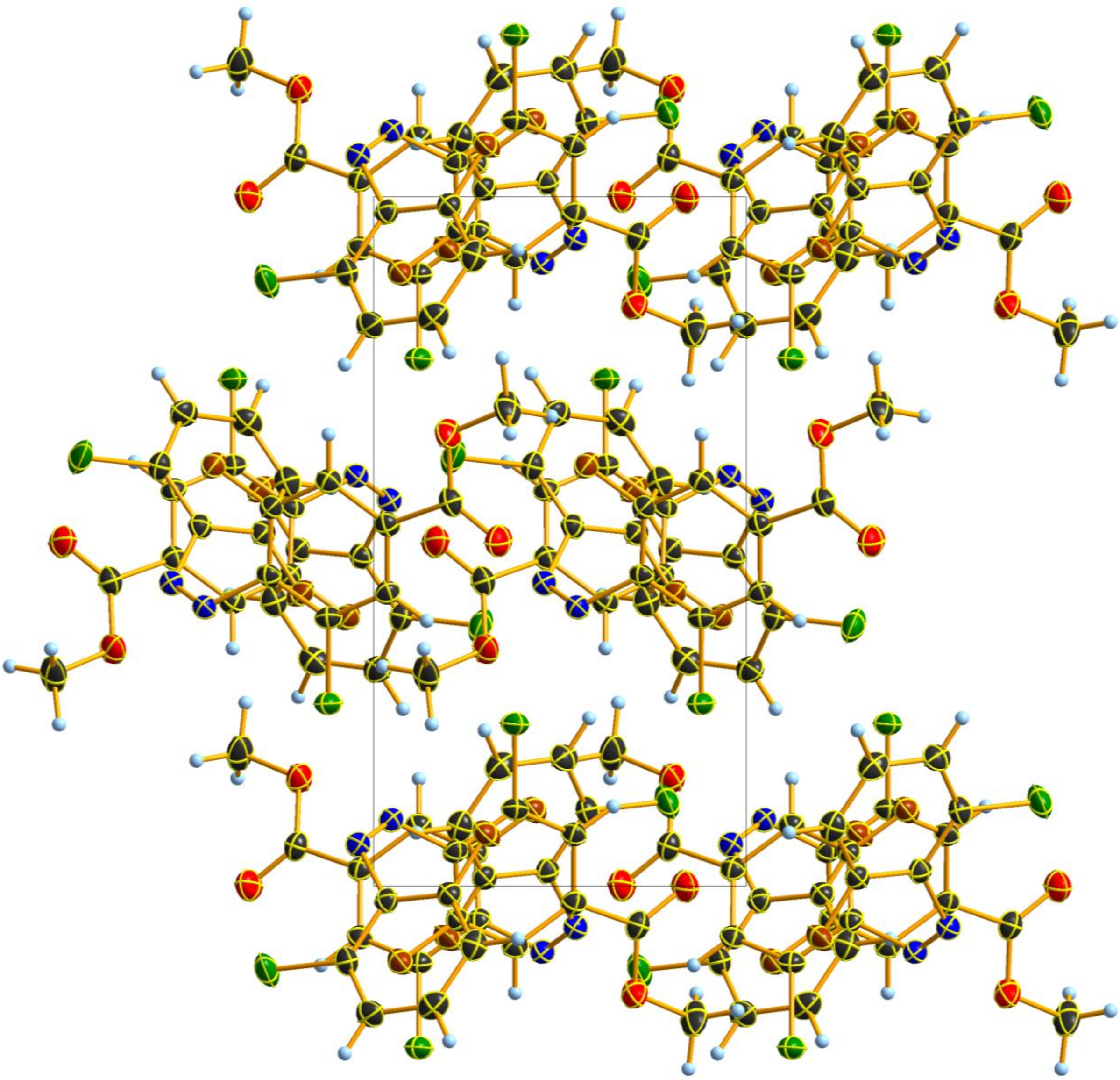

Figure S2.15 Crystal structure of compound ***cis*-21**. View of the unit cell along the *c*-axis. DIAMOND[e)] representation; thermal ellipsoids are drawn at 50 % probability level.

Table S2.14 Selected bond lengths (Å) of compound ***cis*-21.**

Cl1 – C2	1.726(2)	C5 – C6	1.381(3)
F1 – C6	1.358(2)	C7 – C8	1.396(3)
O1 – C13	1.202(3)	C7 – C12	1.398(3)
N1 – N2	1.248(2)	C8 – C9	1.377(3)
N1 – C1	1.436(3)	C9 – C10	1.398(3)
C1 – C6	1.391(3)	C10 – C11	1.391(3)
C1 – C2	1.395(3)	C10 – C13	1.496(3)
Cl2 – C8	1.725(2)	C11 – C12	1.373(3)
F2 – C12	1.358(2)	C4 – C5	1.388(3)
O2 – C13	1.338(3)	C2 – C3	1.379(3)
O2 – C14	1.445(3)	C3 – C4	1.384(3)
N2 – C7	1.440(3)		

Table S2.15 Selected bond angles (°) of compound ***cis*-21**.

N2 – N1 – C1	121.3(2)	C9 – C8 – C7	121.7(2)
C6 – C1 – C2	117.1(2)	C9 – C8 – Cl2	118.9(2)
C6 – C1 – N1	122.4(2)	C7 – C8 – Cl2	119.4(2)
C2 – C1 – N1	119.9(2)	C8 – C9 – C10	119.3(2)
C13 – O2 – C14	114.7(2)	C11 – C10 – C9	120.7(2)
N1 – N2 – C7	122.3(2)	C11 – C10 – C13	122.5(2)
C3 – C2 – C1	121.3(2)	C9 – C10 – C13	116.8(2)
C3 – C2 – Cl1	120.1(2)	C12 – C11 – C10	118.3(2)
C1 – C2 – Cl1	118.6(2)	F2 – C12 – C11	118.6(2)
C2 – C3 – C4	119.6(2)	F2 – C12 – C7	118.4(2)
C3 – C4 – C5	121.1(2)	C11 – C12 – C7	123.0(2)
C6 – C5 – C4	117.8(2)	O1 – C13 – O2	124.8(2)
F1 – C6 – C5	119.2(2)	O1 – C13 – C10	123.6(2)
F1 – C6 – C1	117.8(2)	O2 – C13 – C10	111.6(2)
C5 – C6 – C1	123.0(2)	C8 – C7 – N2	119.5(2)
C8 – C7 – C12	117.1(2)	C12 – C7 – N2	122.5(2)

Table S2.16 Selected torsion angles (°) of compound ***cis*-21.**

N2 – N1 – C1 – C6	61.4(3)	C12 – C7 – C8 – Cl2	178.9(1)
N2 – N1 – C1 – C2	-127.3(2)	N2 – C7 – C8 – Cl2	9.7(3)
C1 – N1 – N2 – C7	7.4(3)	C7 – C8 – C9 – C10	1.8(3)
C6 – C1 – C2 – C3	-3.5(3)	Cl2 – C8 – C9 – C10	-177.8(2)
N1 – C1 – C2 – C3	-175.2(2)	C8 – C9 – C10 – C11	-1.6(3)
C6 – C1 – C2 – Cl1	178.4(2)	C8 – C9 – C10 – C13	176.3(2)
N1 – C1 – C2 – Cl1	6.7(3)	C9 – C10 – C11 – C12	0.4(3)
C1 – C2 – C3 – C4	3.1(3)	C13 – C10 – C11 – C12	-177.4(2)
Cl1 – C2 – C3 – C4	-178.8(2)	C10 – C11 – C12 – F2	179.0(2)
C2 – C3 – C4 – C5	-0.9(3)	C10 – C11 – C12 – C7	0.7(3)
C3 – C4 – C5 – C6	-0.8(3)	C8 – C7 – C12 – F2	-178.8(2)
C4 – C5 – C6 – F1	177.6(2)	N2 – C7 – C12 – F2	-10.0(3)
C4 – C5 – C6 – C1	0.4(3)	C8 – C7 – C12 – C11	-0.5(3)
C2 – C1 – C6 – F1	-175.5(2)	N2 – C7 – C12 – C11	168.4(2)
N1 – C1 – C6 – F1	-4.1(3)	C14 – O2 – C13 – O1	-0.9(3)
C2 – C1 – C6 – C5	1.8(3)	C14 – O2 – C13 – C10	178.9(2)
N1 – C1 – C6 – C5	173.2(2)	C11 – C10 – C13 – O1	171.3(2)
N1 – N2 – C7 – C8	-129.2(2)	C9 – C10 – C13 – O1	-6.6(3)
N1 – N2 – C7 – C12	62.3(3)	C11 – C10 – C13 – O2	-8.5(3)
C12 – C7 – C8 – C9	-0.8(3)	C9 – C10 – C13 – O2	173.6(2)
N2 – C7 – C8 – C9	-170.0(2)		

Trans-dimethyl 4,4'-(diazene-1,2-diyl)(E)-bis(3-chloro-5-fluorobenzoate) (_trans_-28, CCDC 2387094)

Table S2.17 Details for X-ray data collection and structure refinement for compound ***trans*-28**.

	***trans*-28**
Empirical formula	$C_{16}H_{10}Cl_2F_2N_2O_4$
Formula mass	403.16
T[K]	123(2)
Crystal size [mm]	0.40 × 0.03 × 0.01
Crystal description	orange yellow needle
Crystal system	triclinic
Space group	*P*-1
a [Å]	3.8788(2)
b [Å]	9.6203(6)
c [Å]	11.5370(9)
α [°]	105.920(6)
β [°]	98.288(6)
γ [°]	100.436(5)
V [Å^3]	398.50(5)
Z	1
$\rho_{calcd.}$ [g cm^{-3}]	1.680
μ [mm^{-1}]	0.456
F(000)	204
Θ range [°]	2.26 – 25.24
Index ranges	$-5 \leq h \leq 5$
	$-12 \leq k \leq 12$
	$-15 \leq l \leq 15$
Reflns. collected	6841
Reflns. obsd.	1670
Reflns. unique	1953 (R_{int} = 0.0439)
R_1, wR_2 (2σ data)	0.0389, 0.0963
R_1, wR_2 (all data)	0.0481, 0.1023
GOOF on F^2	1.061
Peak/hole [e Å^{-3}]	0.425 / -0.263

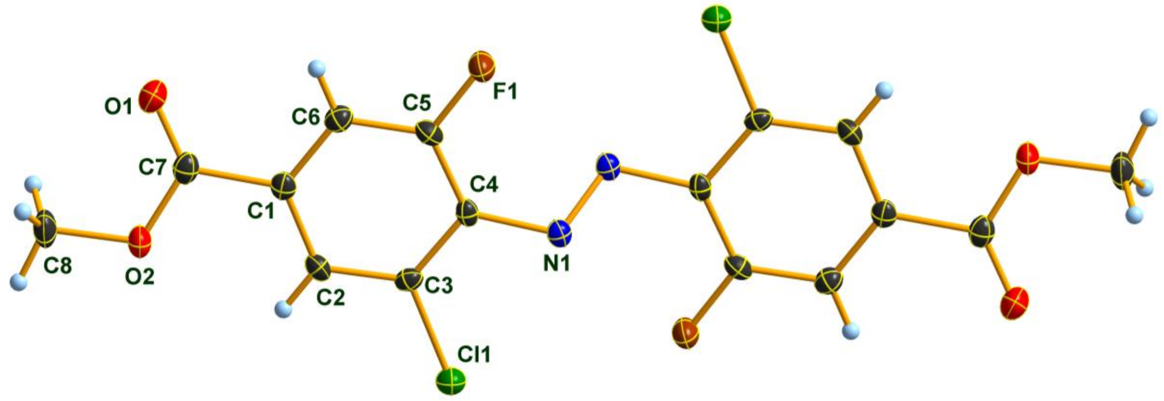

Figure S2.16 Molecular structure of compound ***trans*-28** in the crystal. DIAMOND[e)] representation; thermal ellipsoids are drawn at 50 % probability level. Symmetry code for the symmetry generated half of the molecule: 2-x, -y, 1-z.

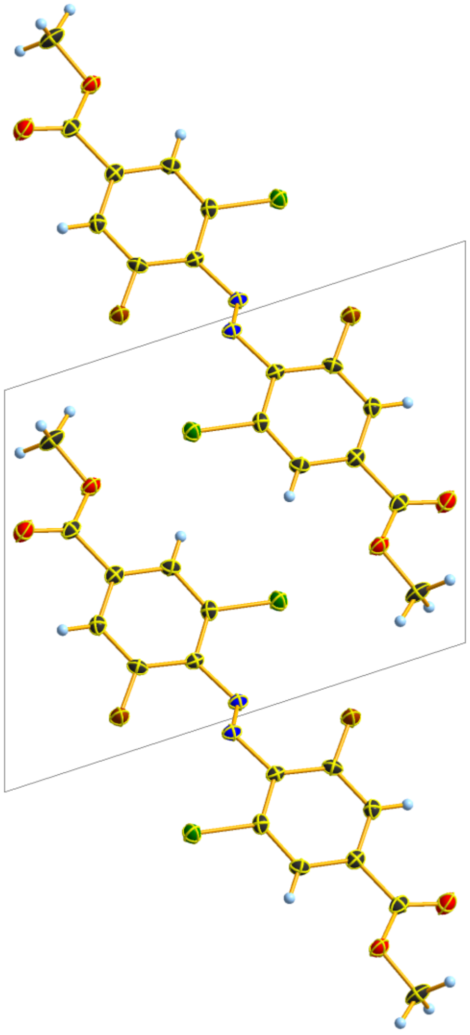

Figure S2.17 Crystal structure of compound ***trans*-28**, view of the unit cell along the *a*-axis. DIAMOND[e)] representation; thermal ellipsoids are drawn at 50 % probability level.

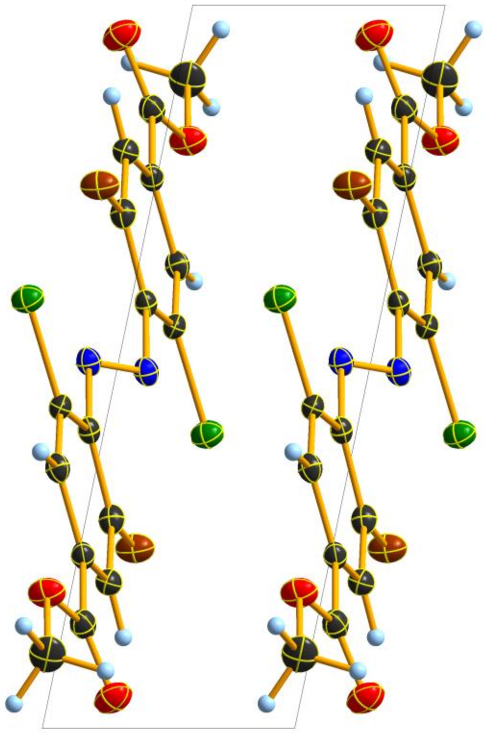

Figure S2.18 Crystal structure of compound ***trans*-28**, view of the unit cell along the *b*-axis. DIAMOND[e)] representation; thermal ellipsoids are drawn at 50 % probability level.

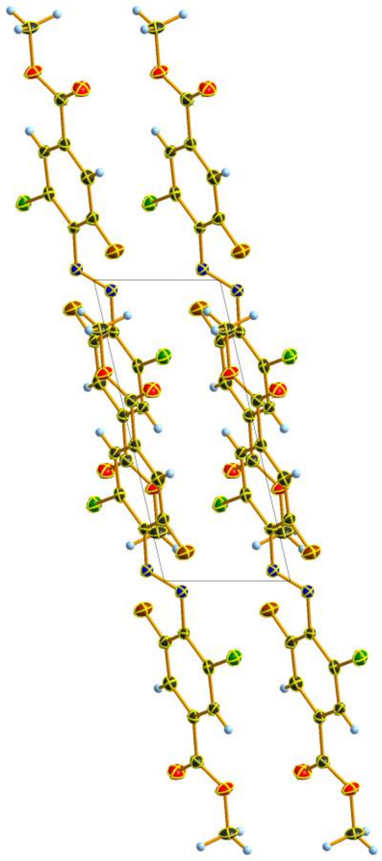

Figure S2.19 Crystal structure of compound ***trans*-28**, view of the unit cell along the *c*-axis. DIAMOND[e)] representation; thermal ellipsoids are drawn at 50 % probability level.

Table S2.18 Selected bond lengths (Å) of compound ***trans*-28.**

Cl1 – C3	1.724(2)	C2 – C3	1.385(2)
F1 – C5	1.369(2)	C3 – C4	1.401(2)
O1 – C7	1.206(2)	C4 – C5	1.403(2)
N1 – N1^{i}	1.251(3)	C5 – C6	1.380(2)
N1 – C4	1.425(2)	C1 – C7	1.496(2)
C1 – C6	1.390(2)	O2 – C7	1.337(2)
C1 – C2	1.394(2)	O2 – C8	1.444(2)

Symmetry code *i*: 2-x, -y, 1-z

Table S2.19 Selected bond angles (°) of compound ***trans*-28.**

N1^{i} – N1 – C4	113.7(2)	F1 – C5 – C4	120.1(2)
C6 – C1 – C2	121.0(2)	C6 – C5 – C4	122.1(2)
C6 – C1 – C7	118.0(2)	O1 – C7 – O2	124.1(2)
C2 – C1 – C7	121.0(2)	O1 – C7 – C1	124.1(2)
C7 – O2 – C8	115.1(2)	O2 – C7 – C1	111.8(2)
C3 – C2 – C1	118.6(2)	C5 – C6 – C1	119.1(2)
C2 – C3 – C4	122.2(2)	C3 – C4 – N1	117.0(2)
C2 – C3 – Cl1	118.3(1)	C5 – C4 – N1	125.9(2)
C4 – C3 – Cl1	119.5(1)	F1 – C5 – C6	117.8(2)
C3 – C4 – C5	117.0(2)		

Symmetry code *i*: 2-x, -y, 1-z

Table S2.20 Selected torsion angles (°) of compound ***trans*-28.**

C6 – C1 – C2 – C3	-1.1(2)	C3 – C4 – C5 – C6	-1.9(2)
C7 – C1 – C2 – C3	178.8(1)	N1 – C4 – C5 – C6	-179.0(2)
C1 – C2 – C3 – C4	-1.1(2)	C8 – O2 – C7 – O1	1.2(3)
C1 – C2 – C3 – Cl1	179.2(1)	C8 – O2 – C7 – C1	-177.8(1)
C2 – C3 – C4 – C5	2.5(2)	C6 – C1 – C7 – O1	8.3(3)
Cl1 – C3 – C4 – C5	-177.8(1)	C2 – C1 – C7 – O1	-171.5(2)
C2 – C3 – C4 – N1	179.9(1)	C6 – C1 – C7 – O2	-172.6(1)
Cl1 – C3 – C4 – N1	-0.4(2)	C2 – C1 – C7 – O2	7.5(2)
N1 – N1 – C4 – C3	150.8(2)	F1 – C5 – C6 – C1	-178.0(1)
N1 – N1 – C4 – C5	-32.0(3)	C4 – C5 – C6 – C1	-0.2(3)
C3 – C4 – C5 – F1	176.0(1)	C2 – C1 – C6 – C5	1.7(2)
N1 – C4 – C5 – F1	-1.1(2)	C7 – C1 – C6 – C5	-178.2(1)

Symmetry code *i*: 2-x, -y, 1-z

9. References

[1] C. Knie, M. Utecht, F. Zhao, H. Kulla, S. Kovalenko, A. M. Brouwer, P. Saalfrank, S. Hecht, D. Bléger, *Chem. – Eur. J.* **2014**, *20*, 16492–16501.
