# Supplementary material for: A Platform for the Development of Highly Red‐Shifted Azobenzene‐Based Optical Tools
Source: Angew Chem Int Ed Engl. 2025 Jun 23;64(32):e202501779. doi: 10.1002/anie.202501779 (PMC12322654; doi:10.1002/anie.202501779)
Supplement: Supplementary file 3 — Supporting Information [file ANIE-64-e202501779-s004.pdf]

A Platform for the Development of Highly Red-Shifted Azobenzene-based Optical Tools Supporting Information: Quantum-Chemical Calculations

Kyra Lützel,# Henryk Laqua,# Manjima B. Sathian,# Benedikt Nißl# Judit Katalin Szántó, Christina-Anna Senser, Gökcen Savasci, Lars Allmendinger, Bilal Kicin, Vincent Ruf, Dominik Kammerer, Theobald Lohmüller, Konstantin Karaghiosoff, Ahmed M. Ali, Ursula Storch,* Michael Mederos y Schnitzler,* Christian Ochsenfeld,* David B. Konrad*

1. Methodology and workflow

The quantum-chemical calculations were performed according to the following computational workflow:

1. Minimum conformer searches with CREST [1] (GFN2-xTB [2,3], GFN-FF [4]) were performed to find start-structures for all conformers (*cis* and *trans*) of each compound. Similarly, CREST was also used to find start structures for the *cis*→*trans* isomerization transition structure employing a constrained transition state search with a N-N-C bond angle of 180 degrees and a force-constant of 0.25 a u .

2. All of the so found structures were subsequently QM-optimized using the PBEh-3c composite method (see text below for details regarding the electronic structure methods). [5] The minimum structures were optimized using the FermiONs++ [6,7] internal optimizer, whereas the transition structures were optimized using the dimer method as implemented in dl-find [8], and we employed the pychemshell [9] program to facilitate the interfacing between the electronic structure program (FermiONs++) with the optimization library (dl-find). The most stable conformers of each *cis*, *trans* and transition structure of each compound were then verified by frequency analysis to contain zero (for minima) or one (for transition structures) significant imaginary modes. Some structures featured additional very low frequency imaginary modes which are caused by numerical noise slightly altering these very flat potential energy surfaces. We tried optimization along those frequencies, but no significant (<0.1 kcal/mol) change in energy was observed. The final *cis*→trans energies and barriers were then obtained from energy differences of the most stable conformers and corrected for zero-point vibrational energy contributions. We decided not to correct for finite temperature effects, since such corrections are extremely sensitive to low frequency vibrational modes where the underlying harmonic approximation itself breaks down.

3. Starting from the most stable *cis* and *trans* conformers, respectively, unbiased ground-state *ab-initio* MD (AIMD) simulations were performed, employing the same PBEh-3c composite electronic structure method as in step 2. The starting velocities were initialized with pseudo-random values corresponding to the simulation temperature of 300 K, which was then maintained throughout the simulation employing the Bussi-Donadio-Parinello thermostat. [10] For each structure (*cis* and *trans* isomers are considered separate structures here), two MD trajectories with a step-time of 1 fs and a simulation time of 30 ps each were performed. The two separate simulations started from the same optimized minimum structure but employed different pseudo-random starting velocities and a different seed for the pseudo-random number generator used in the thermostat. Therefore, both trajectories diverge from their common starting structure quite quickly (<1 ps) meaning they provide virtually uncorrelated sampling after a short initialization period.

4. Every 10 fs a snapshot of the structure was taken and its excitation line-spectrum (linear-response time-dependent density-functional-theory (lr-TD-DFT) using the random phase approximation (RPA) [11]; first two

Prof. Dr. D. B. Konrad*, Mrs. K. Lützel#, Mrs. M. B. Sathian#, Mr. B. Nißl#, Dr. L. Allmendinger, Mr. B. Kicin, Mr. V. Ruf, Dr. Ahmed M. Ali
Department of Pharmacy
Ludwig Maximilian University of Munich
Butenandtstr. 5-13, 81377 Munich, Germany
E-mail: david.konrad@cup.lmu.de

Prof. Dr. C. Ochsenfeld*, Dr. H. Laqua#, Mrs. J. K. Szántó, Mr. G. Savasci, Prof. Dr. K. Karaghiosoff
Department of Chemistry
Ludwig Maximilian University of Munich
Butenandtstr. 5-13, 81377 Munich, Germany
E-mail: c.ochsenfeld@fkf.mpg.de

Prof. Dr. U. Storch*, Prof. Dr. M. Mederos y Schnitzler*
Walther Straub Institute of Pharmacology and Toxicology
Ludwig Maximilian University of Munich
Goethestr. 33, 80336 Munich, German
Email: mederos@lrz.uni-muenchen.de

Dr. H. Laqua#
College of Chemistry
University of California
Berkeley, California 94720, United States of America

Prof. Dr. C. Ochsenfeld*, Mr. G. Savasci
Max Planck Institute for Solid State Research
Heisenbergstr. 1, 70569 Stuttgart, Germany

Mr. D. Kammerer, PD Dr. T. Lohmüller
Chair for Photonics and Optoelectronics
Nano-Institute Munich, Department of Physics
Ludwig Maximilian University of Munich
Königinstraße 10, 80539 Munich, Germany

Dr. A. M. Ali
Department of Medicinal Chemistry
Faculty of Pharmacy
Assiut University
Assiut, 71515 Egypt

Mrs. C.-A. Senser, Prof. Dr. U. Storch*
Institute of Pharmacy
University of Regensburg
Universitätsstr. 31, 93040 Regensburg, Germany
Email: ursula.storch@chemie.uni-regensburg.de

Prof. Dr. D. B. Konrad*
Department of Pharmaceutical Sciences
University of Vienna
Josef-Holaubek-Platz 2, 1090 Vienna, Austria
E-mail: david.benjamin.konrad@univie.ac.at

[#] these authors contributed equally to this work
[*] corresponding authors

excitations; $n \to \pi^*$ and $\pi \to \pi^*$) was computed for a total of 6000 line-spectra per structure. These 6000 line-spectra were assembled into one complete spectrum (intensity in arbitrary units as a function of the wavenumber $I(\tilde{\nu})$) by averaging the Gaussian-smoothed line-spectra according to

$$I(\tilde{\nu}) = \sum_{i=1}^{6000} I_i \exp\big(-\frac{(\tilde{\nu}-\tilde{\nu}_i)^2}{2\sigma}\big), \tag{1}$$

where I_i denotes the intensity (i.e., the computed oscillator strength) of each data point i, $\tilde{\nu}_i$ denotes the computed excitation energy (in wavenumbers) of the corresponding data point, and σ denotes the standard deviation of the Gaussian smoothing which was set to $\sigma = 800\,\mathrm{cm}^{-1}$ as this produces smooth spectra without meaningfully altering the line form. That is, the line-width of the smoothed spectra is almost completely caused by the spread of the individual data points and not the smoothing procedure.

For our "workhorse" electronic structure method we chose the PBEh-3c composite method of Grimme and co-workers, which provides a very reasonable compromise between accuracy and performance. In particular, it combines the Perdew-Burke-Ernzerhof (PBE)[12] semi-local density functional with exact (Fock-)exchange analogously to the PBE0 hybrid functional[13,14] but uses a re-optimized parameter set in the PBE part and a higher fraction of exact-exchange (42 % instead of 25 % PBE0). Moreover, it employs a purpose-build double-zeta basis

set (def2-mSVP)[15] and the D3-dispersion correction was adapted to cancel some of the basis set superposition error (BSSE) causes by the modest basis size. Therefore, since functional, basis set, and dispersion correction are co-designed, PBEh-3c provides a better accuracy than comparable combinations of functionals and basis sets, (e.g., PBE0-d3/def2-SVP) at the same computational cost. Additionally, the higher fraction of exact-exchange makes it better suited for studying reaction energies as well as excitation energies, since it has been empirically shown that higher admixtures of exact-exchange increases the accuracy for both properties.[16,17]

2. Spectroscopy and Thermochemistry

In Tables S3.1 and S3.2 we provide a summary of the computed $n \rightarrow \pi^*$ excitation spectra of the *cis* and *trans* isomers of each compound as well as the *cis*→*trans* reaction energies and barriers computed in vacuum and in DMSO as implicit solvent (PCM), respectively. In addition, Table S3.3 contains the position of computed absorption maxima blue-shifted by 900 cm^{-1} to better match the experimental spectra. The purpose of this blue-shift is to correct for systematic errors in our methodology (predominantly the errors of the PBEh-3c electronic structure method). It does not affect differences between compounds, meaning that trends arising from varying substitution patterns are preserved.

Table S3.1. Calculated absorption maxima (Abs. Max), relative absorption intensity (i.e., peak integral [arbitrary units]) of the $n \rightarrow \pi^*$ excitation of *cis* and *trans* isomers as well as the *cis*→*trans* reaction energy and barrier (energy differences of most stable conformers corrected for zero-point vibrational energies) *in vacuum*.

Molecule	Abs. Max [nm]		rel. Intensity		*cis*→*trans* [kcal/mol]	
	trans	*cis*	*trans*	*cis*	energy	barrier
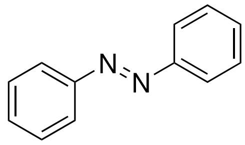 **1**	459	461	20	65	-13.6	26.1
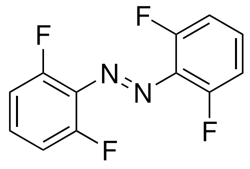 **2**	476	435	49	67	-6.5	31.1
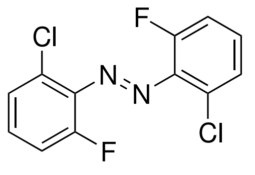 **3**	490	441	37	58	-5.7	29.8
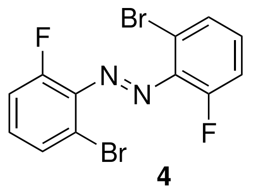 **4**	495	452	39	53	-5.7	29.0
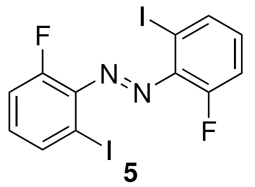 **5**	498	455	37	42	-5.9	28.8

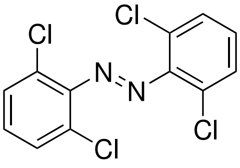 **6**	500	465	39	56	-6.0	26.3
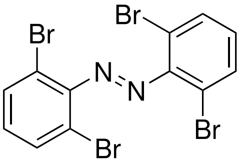 **7**	500	481	40	44	-8.4	23.6
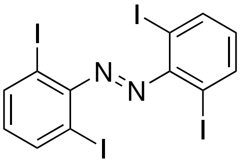 **8**	524	505	30	36	-12.5	20.8
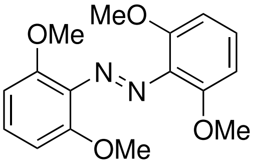 **9**	490	444	88	67	-3.2	30.7
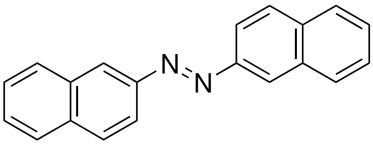 **10**	446	469	46	137	-14.6	25.5
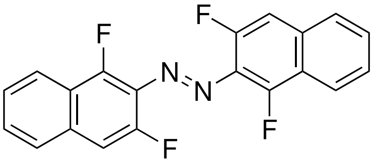 **11**	483	441	110	140	-6.5	30.7
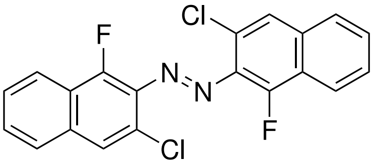 **12**	495	446	98	116	-5.5	30.1
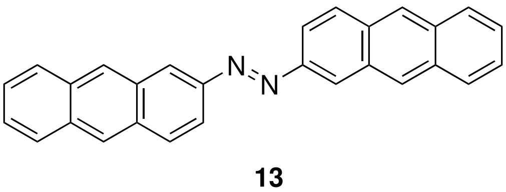 **13**	439	481	255	260	-15.2	25.1

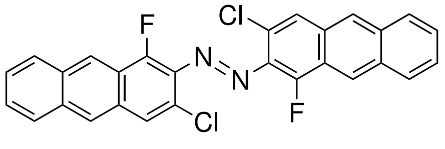 **14**	505	459	184	232	-5.6	29.9
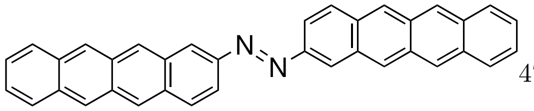 **15**	476	505	1376	374	-15.3	24.9
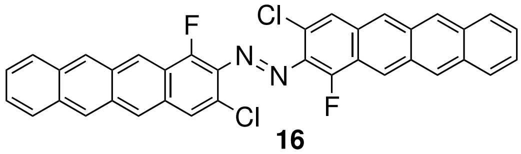 **16**	518	481	496	339	-5.5	29.8
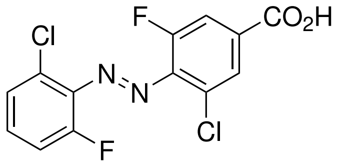 **17**	500	446	51	77	-5.4	25.9
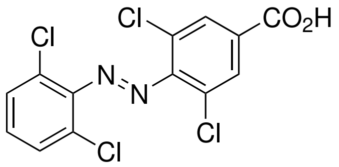 **18**	503	467	63	73	-5.8	22.9
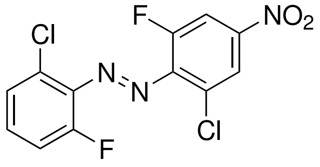 **19**	493	448	61	86	-5.6	24.3
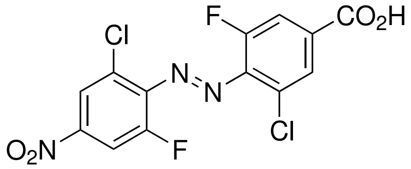 **20**	515	452	62	99	-5.1	25.6
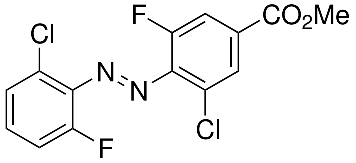 **21**	498	446	53	81	-5.4	26.3

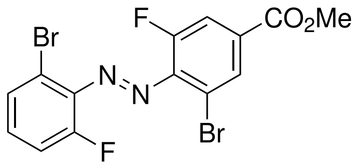 **22**	493	459	60	74	-5.6	25.9
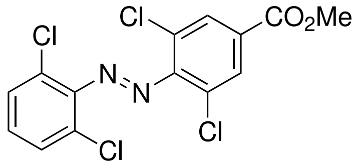 **23**	513	469	44	74	-5.8	23.3
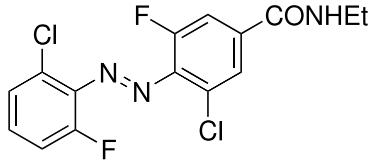 **24**	498	446	51	79	-5.2	27.6
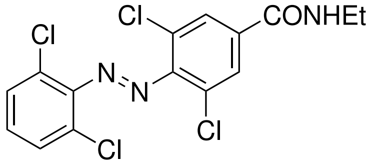 **25**	503	467	49	71	-5.9	24.5
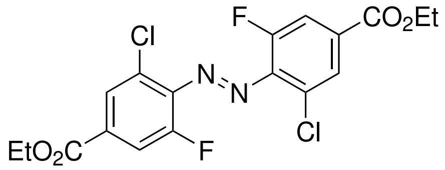 **26**	503	450	81	104	-5.0	27.5
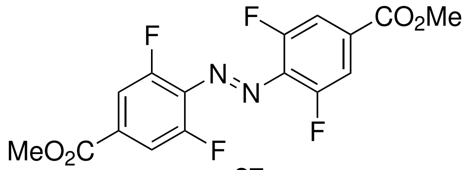 **27**	493	444	87	118	-5.9	28.5
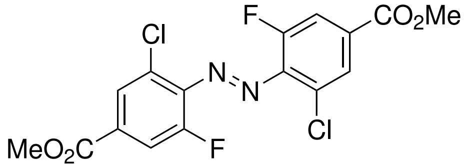 **28**	503	455	89	102	-5.9	26.6
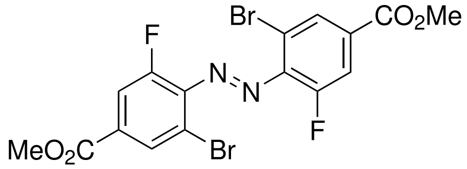 **29**	510	463	58	94	-5.2	27.0

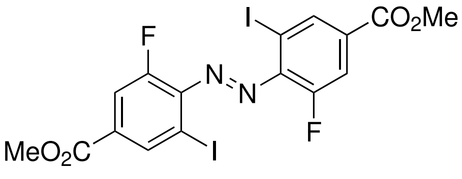 **30**	513	472	76	78	-5.6	26.6
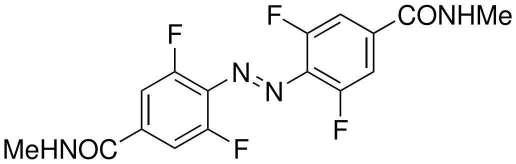 **31**	485	441	73	109	-6.3	28.0
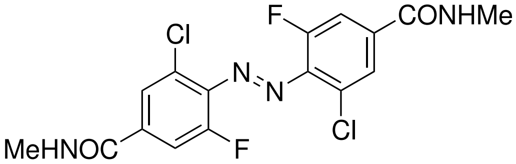 **32**	498	448	62	94	-5.2	28.4
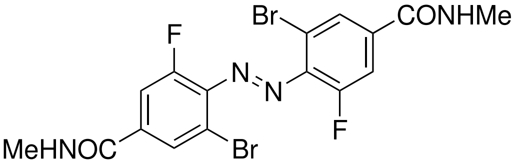 **33**	503	459	62	89	-5.3	28.1
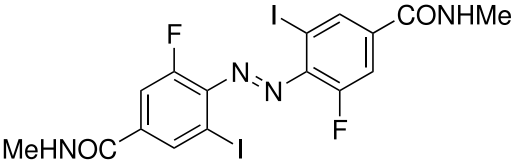 **34**	505	465	66	75	-5.4	27.5
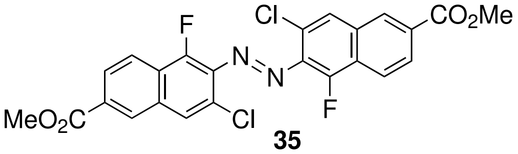 **35**	500	452	126	161	-5.3	29.3
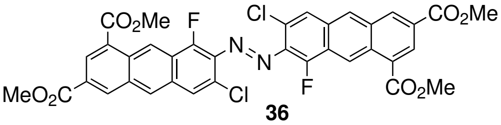 **36**	498	450	274	338	-5.1	29.3
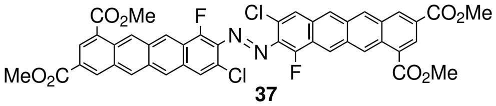 **37**	508	483	758	519	-5.5	27.7

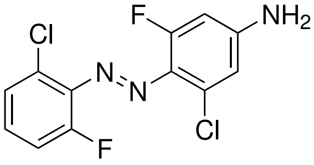 **38**	476	444	59	103	-7.3	26.8
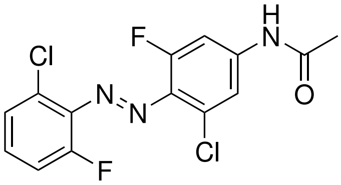 **39**	488	442	69	96	-6.2	28.5
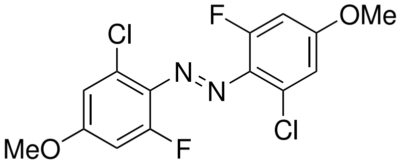 **40**	476	442	50	107	-7.4	29.7
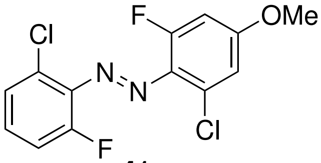 **41**	478	444	52	82	-6.6	28.0
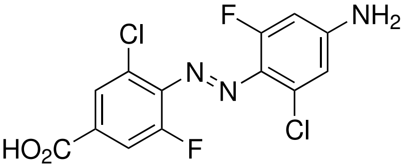 **42**	485	455	83	149	-7.1	22.8
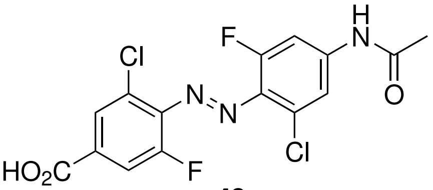 **43**	495	448	78	129	-6.2	24.5
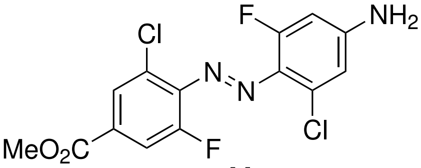 **44**	508	452	48	100	-5.5	23.1
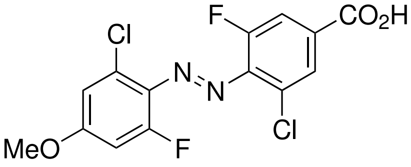 **45**	485	452	70	115	-6.3	24.4

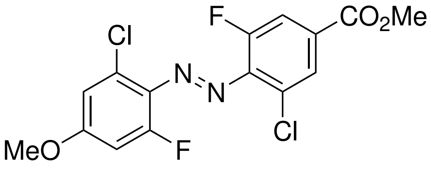 **46**	490	448	66	116	-7.1	24.1
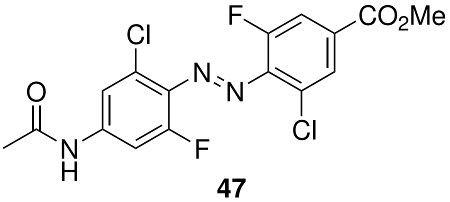 **47**	493	450	89	125	-6.2	24.9
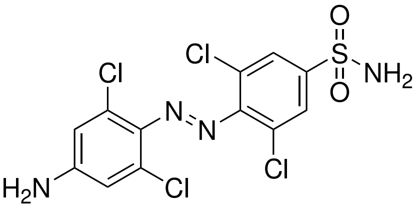 **48**	500	472	78	129	-7.1	19.9
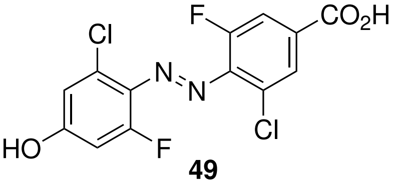 **49**	488	448	62	104	-6.4	24.4
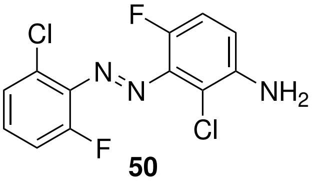 **50**	490	442	32	63	-5.2	29.7
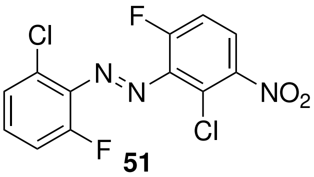 **51**	483	444	46	58	-5.7	27.7
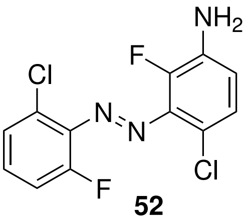 **52**	446	441	59	57	-5.5	29.5

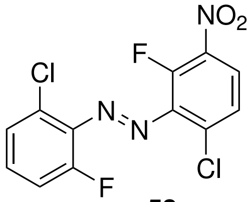 **53**	488	442	42	56	-5.5	28.2
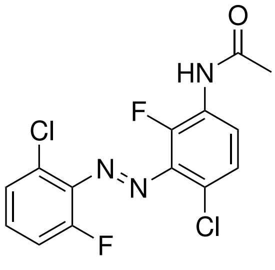 **54**	488	442	50	56	-7.7	26.9
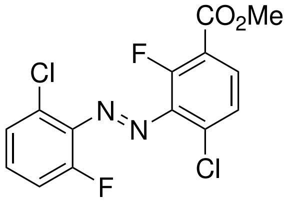 **55**	490	442	49	58	-5.4	29.7

Table S3.2. Calculated absorption maxima (Abs. Max), relative absorption intensity (i.e., peak integral [arbitrary units]) of the $n \rightarrow \pi^*$ excitation of *cis* and *trans* isomers as well as the *cis*→*trans* reaction energy and barrier (energy differences of most stable conformers corrected for zero-point vibrational energies) employing an implicit solvent environment (polarizable continuum model (C-PCM) [18,19] with dimethyl-sulfoxide (DMSO)).

Molecule	Abs. Max [nm]		rel. Intensity		*cis*→*trans* [kcal/mol]	
	trans	*cis*	*trans*	*cis*	energy	barrier
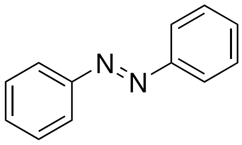 **1**	455	457	56	131	-11.5	27.2
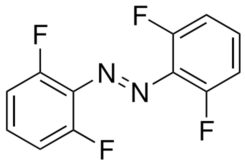 **2**	465	435	125	141	-6.3	32.2
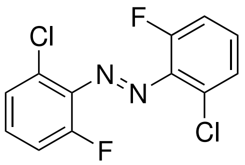 **3**	481	439	91	124	-5.2	31.1

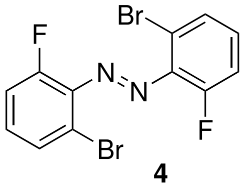 **4**	485	448	96	118	-5.2	30.6
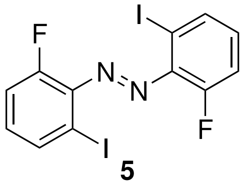 **5**	488	450	92	100	-5.2	30.5
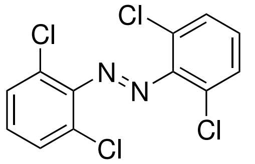 **6**	493	461	91	120	-5.4	27.6
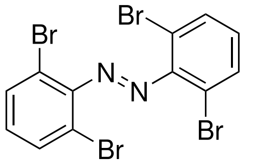 **7**	493	474	95	103	-7.9	25.3
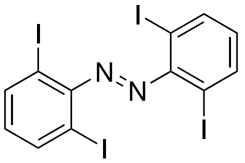 **8**	515	498	76	90	-11.8	22.4
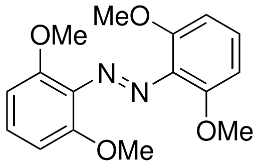 **9**	485	444	209	147	-2.9	34.4
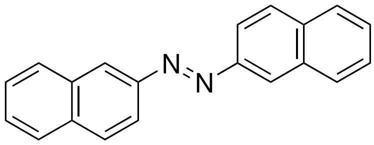 **10**	441	465	212	285	-12.7	27.5
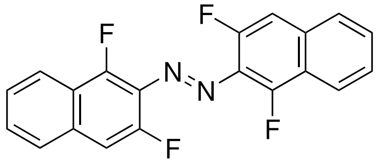 **11**	472	441	352	302	-6.5	31.5

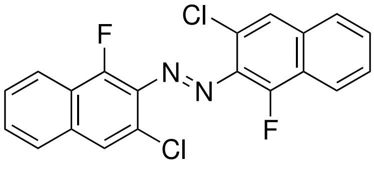 **12**	485	444	279	272	-5.1	31.5
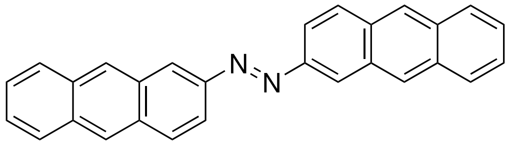 **13**	442	481	2116	560	-13.3	25.7
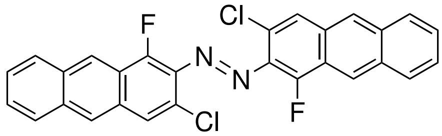 **14**	493	461	809	568	-5.1	30.9
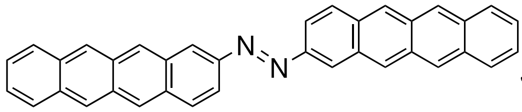 **15**	518	513	3188	681	-13.6	25.4
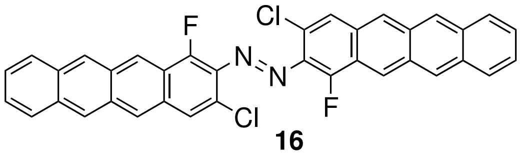 **16**	549	508	1800	821	-4.9	30.9
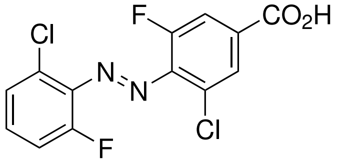 **17**	490	444	112	150	-5.0	26.2
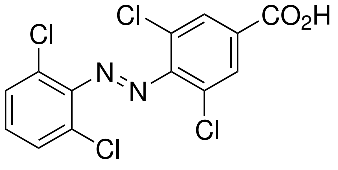 **18**	498	463	132	142	-5.3	23.4
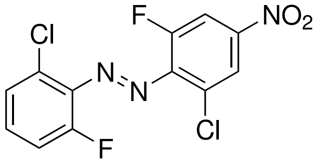 **19**	488	444	138	175	-5.1	23.4

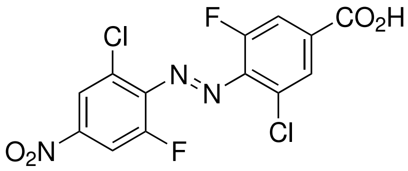 **20**	510	450	125	179	-5.6	24.1
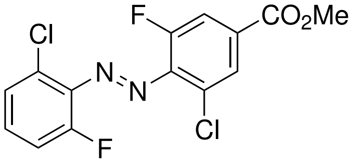 **21**	490	444	115	155	-5.0	26.9
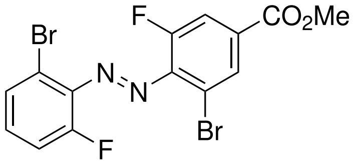 **22**	485	455	129	147	-5.1	26.1
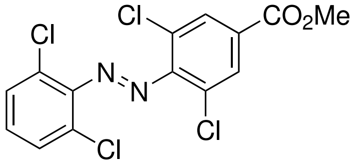 **23**	508	467	90	144	-5.4	23.5
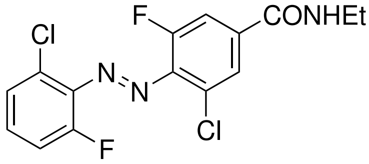 **24**	488	444	113	153	-5.0	28.7
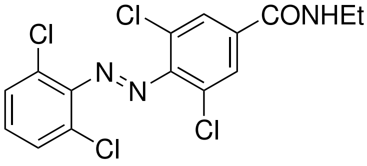 **25**	498	463	103	140	-5.5	26.2
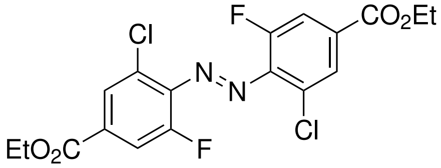 **26**	495	448	152	180	-4.6	27.9
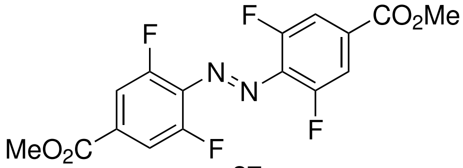 **27**	483	442	173	203	-5.9	28.7

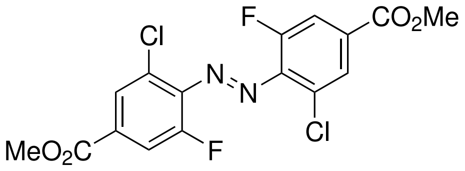 **28**	495	452	168	178	-5.7	26.8
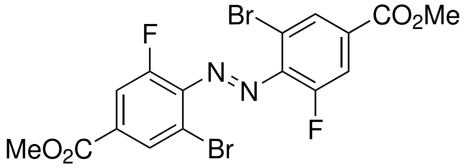 **29**	500	459	115	171	-4.8	27.4
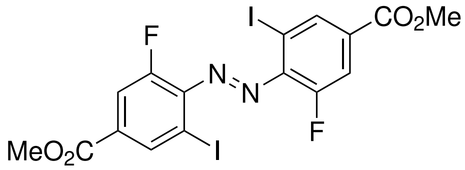 **30**	508	465	149	147	-3.1	29.4
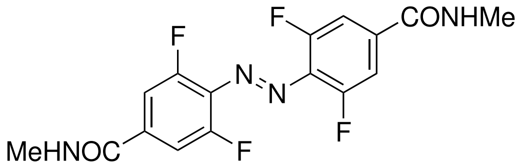 **31**	478	439	152	195	-6.1	28.7
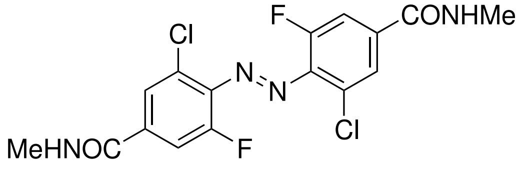 **32**	490	444	122	171	-4.9	29.3
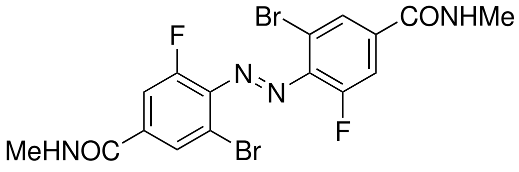 **33**	495	455	126	168	-5.0	28.9
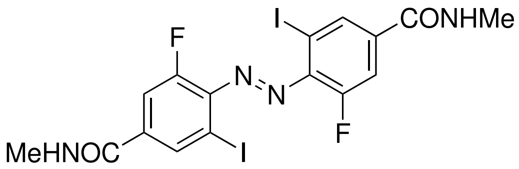 **34**	498	461	133	146	-5.3	28.9
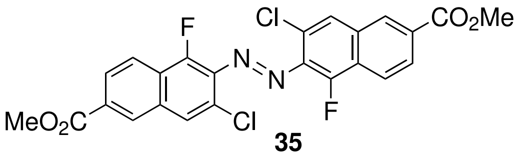 **35**	493	448	290	327	-4.8	30.1

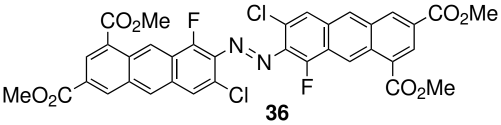 36	490	455	779	701	-4.9	29.8
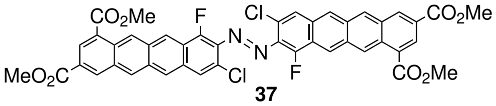 37	541	515	1511	1039	-5.0	30.2
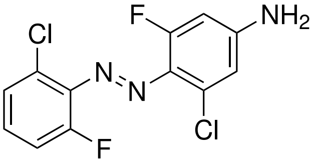 38	463	446	236	295	-7.3	27.2
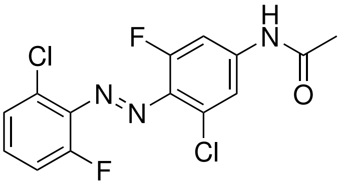 39	478	441	187	212	-6.0	29.6
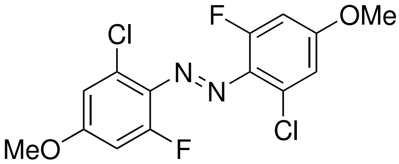 40	467	444	159	245	-6.8	30.8
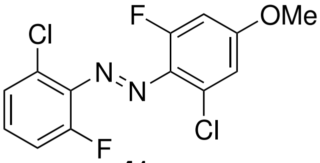 41	469	442	144	183	-6.2	29.2
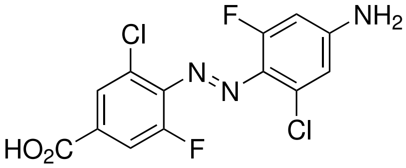 42	476	459	318	395	-7.7	21.7
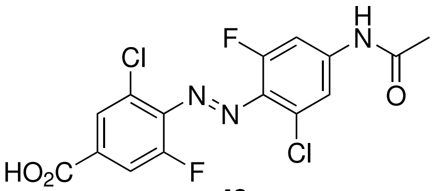 43	485	448	195	266	-6.1	24.6

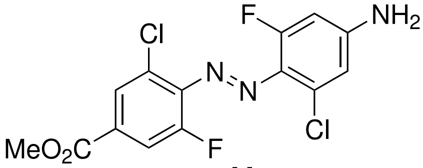 **44**	505	448	296	381	-7.6	22.1
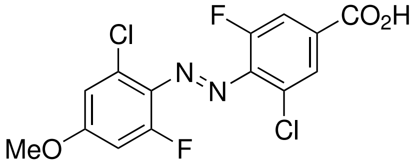 **45**	478	450	177	236	-6.1	24.3
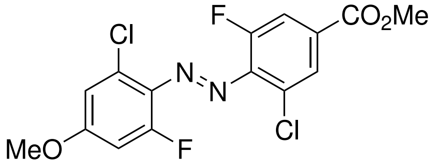 **46**	481	446	167	233	-6.1	24.8
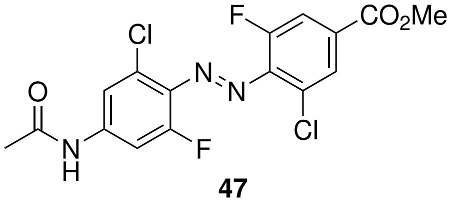 **47**	483	450	211	259	-5.9	24.9
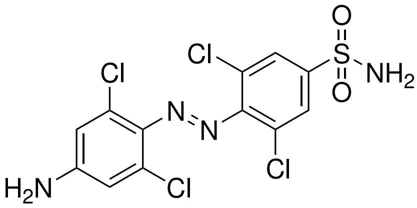 **48**	493	476	253	336	-7.4	18.9
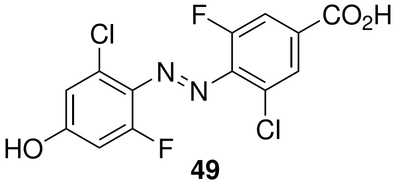 **49**	481	446	162	220	-6.2	24.3
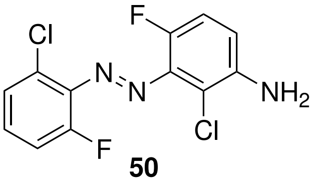 **50**	481	441	88	146	-4.9	31.1
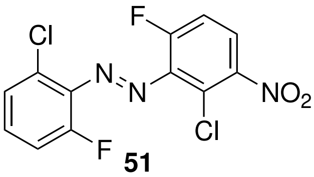 **51**	476	442	105	122	-5.1	28.9

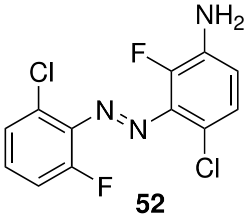 **52**	444	439	126	123	-5.0	31.1
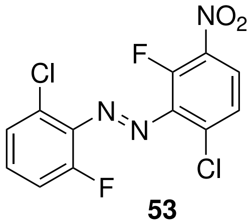 **53**	481	439	97	117	-5.2	29.0
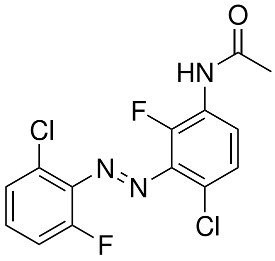 **54**	476	439	137	118	-8.8	26.8
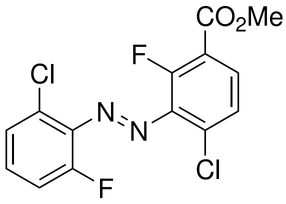 **55**	483	441	110	122	-4.9	30.4

Table S3.3. Calculated absorption maxima (Abs. Max), relative absorption intensity (rel. Intensity) analogous to Table S3.2 (implicit solvent employing C-PCM) but blue-shifted by 900 cm^{-1} to better match the experimental spectra. Absorption intensities are unchanged.

Molecule	Abs. Max [nm]		rel. Intensity	
	trans	*cis*	*trans*	*cis*
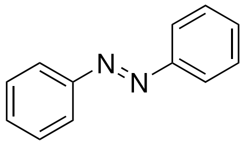 **1**	441	442	56	131
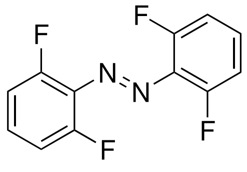 **2**	457	418	125	141

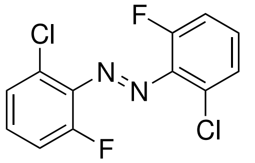 **3**	469	424	91	124
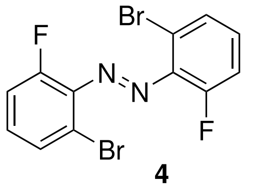 **4**	474	435	96	118
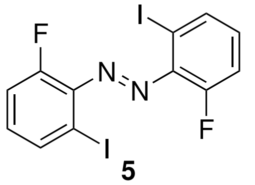 **5**	476	437	92	100
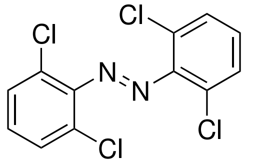 **6**	478	446	91	120
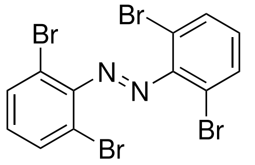 **7**	478	461	95	103
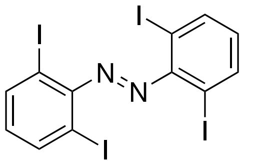 **8**	500	483	76	90
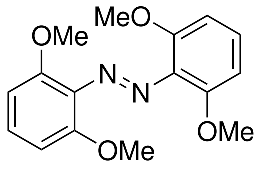 **9**	469	427	209	147
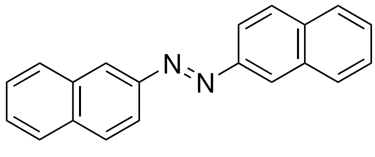 **10**	429	450	212	285

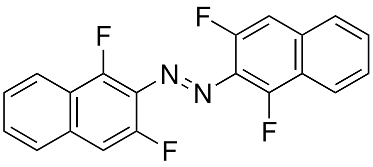 **11**	463	424	352	302
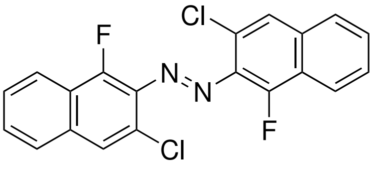 **12**	474	429	279	272
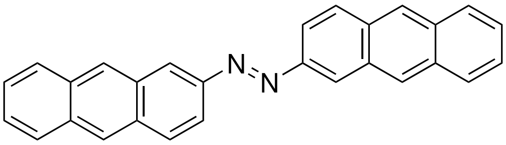 **13**	422	461	2116	560
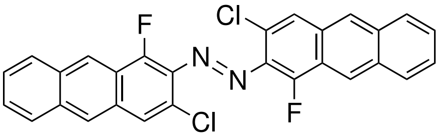 **14**	483	441	809	568
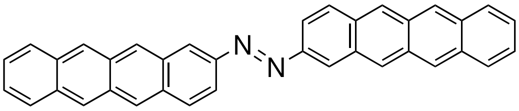 **15**	457	483	3188	681
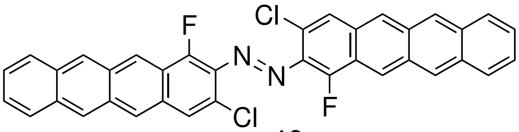 **16**	495	461	1800	821
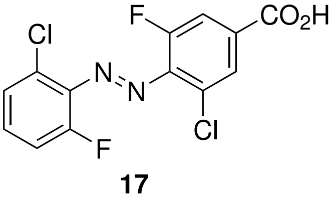 **17**	478	429	112	150
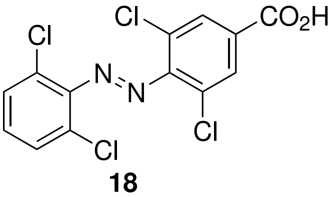 **18**	481	448	132	142

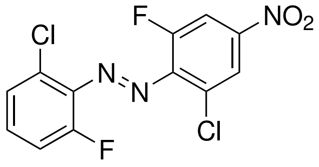 **19**	472	431	138	175
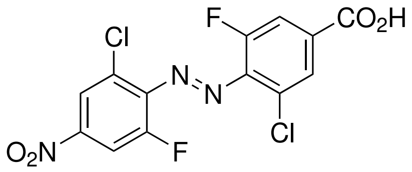 **20**	493	435	125	179
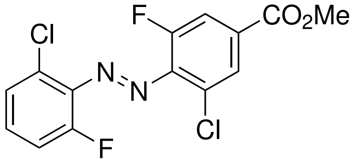 **21**	476	429	115	155
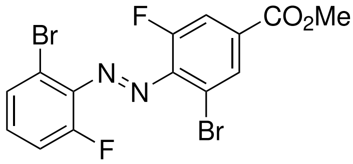 **22**	472	441	129	147
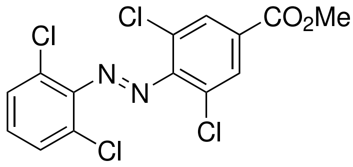 **23**	490	450	90	144
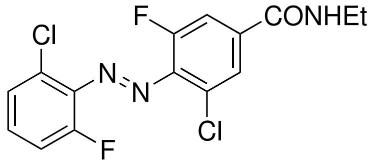 **24**	476	429	113	153
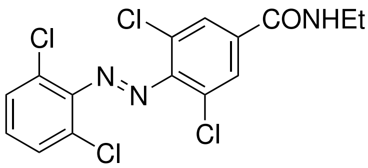 **25**	481	448	103	140
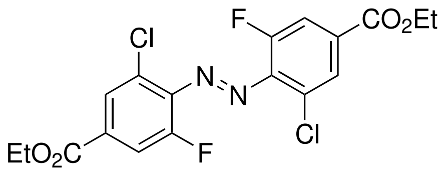 **26**	481	433	152	180

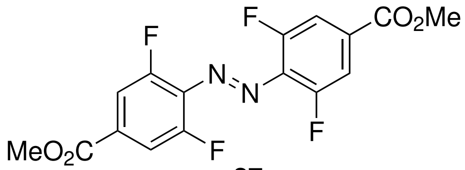 **27**	472	427	173	203
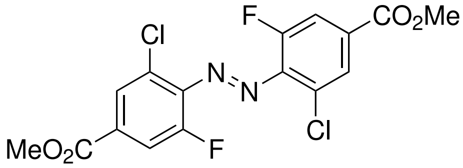 **28**	481	437	168	178
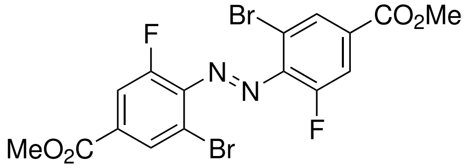 **29**	488	444	115	171
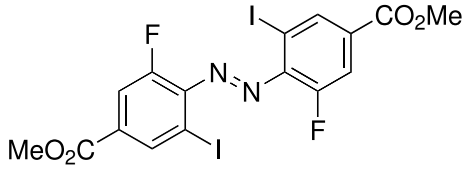 **30**	490	452	149	147
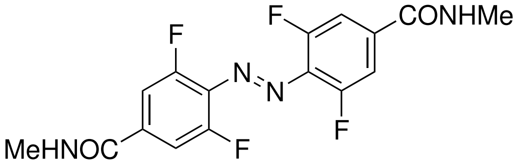 **31**	465	424	152	195
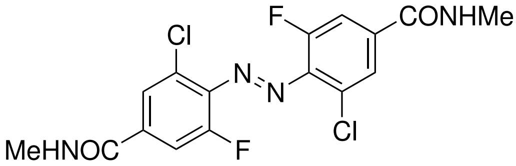 **32**	476	431	122	171
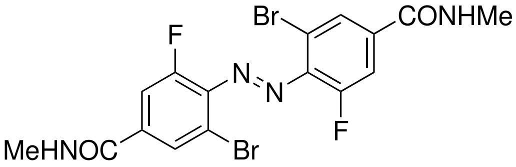 **33**	481	441	126	168

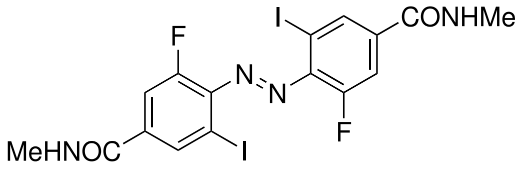 **34**	483	446	133	146
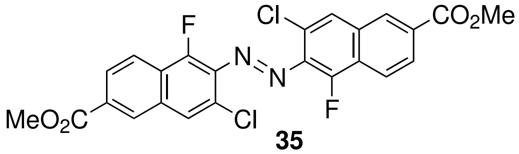 **35**	478	435	290	327
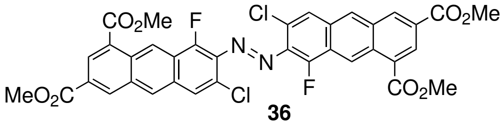 **36**	476	433	779	701
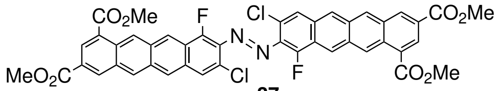 **37**	485	463	1511	1039
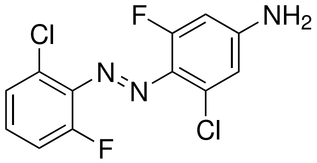 **38**	457	427	236	295
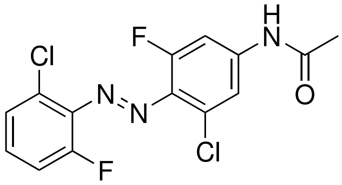 **39**	467	426	187	212
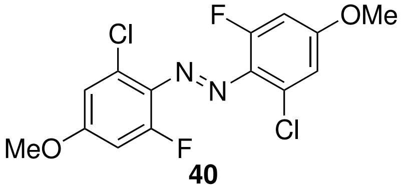 **40**	457	426	159	245
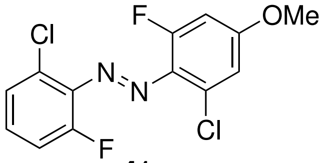 **41**	459	427	144	183
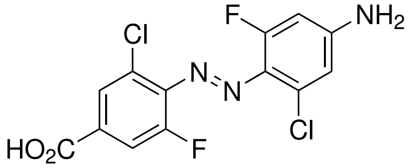 **42**	465	437	318	395

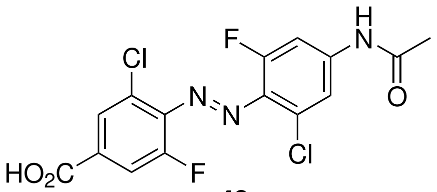 **43**	474	431	195	266
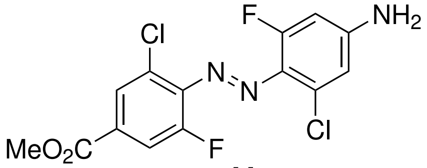 **44**	485	435	296	381
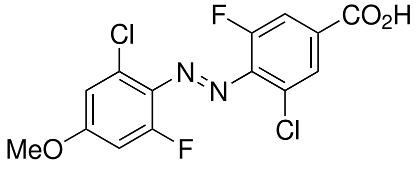 **45**	465	435	177	236
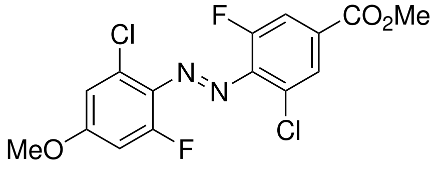 **46**	469	431	167	233
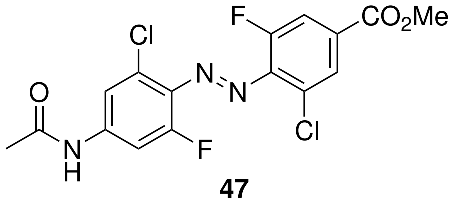 **47**	472	433	211	259
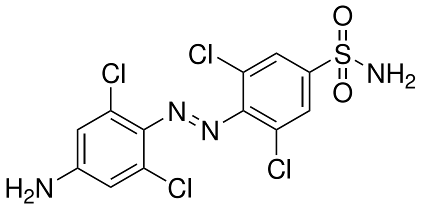 **48**	478	452	253	336
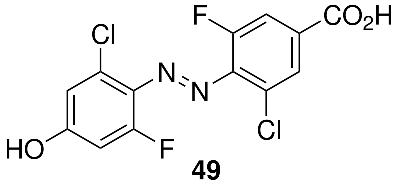 **49**	467	431	162	220

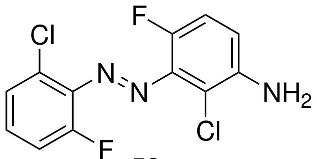 **50**	469	426	88	146
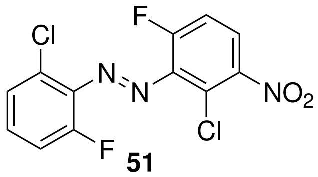 **51**	463	427	105	122
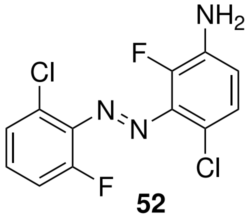 **52**	429	424	126	123
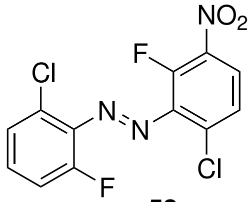 **53**	467	426	97	117
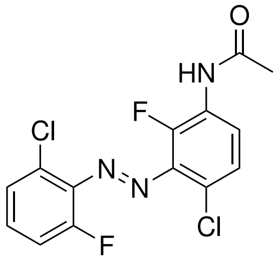 **54**	467	426	137	118
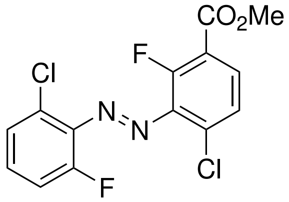 **55**	469	426	110	122

2.1. Molecule 1

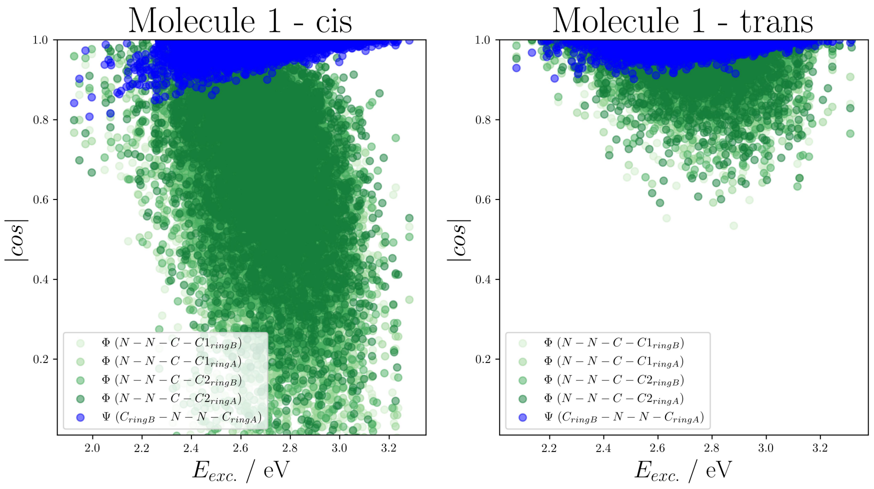

Figure S3.1. Distribution of all 6000 data points for *cis*- (left) and *trans*-conformer (right) of molecule 1.

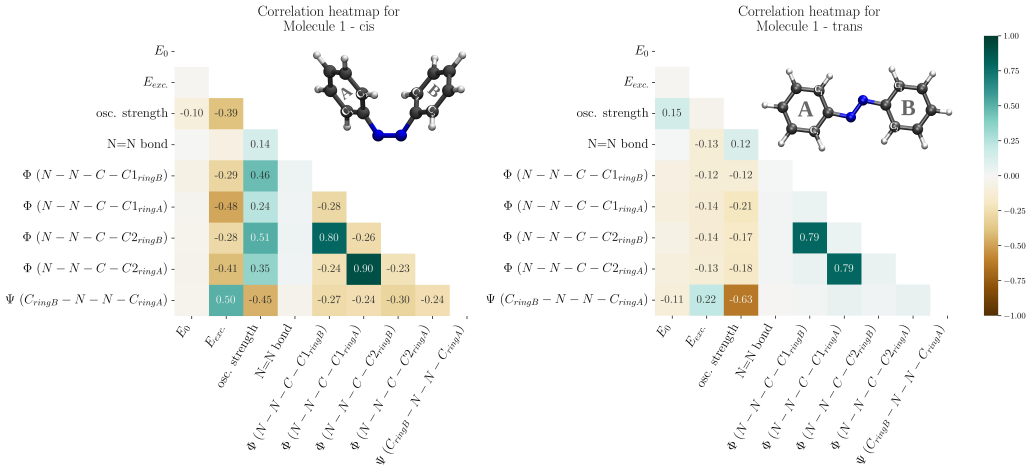

Figure S3.2. Correlation plots for *cis*- (left) and *trans*-conformers (right) of molecule 1.

2.2. Molecule 3

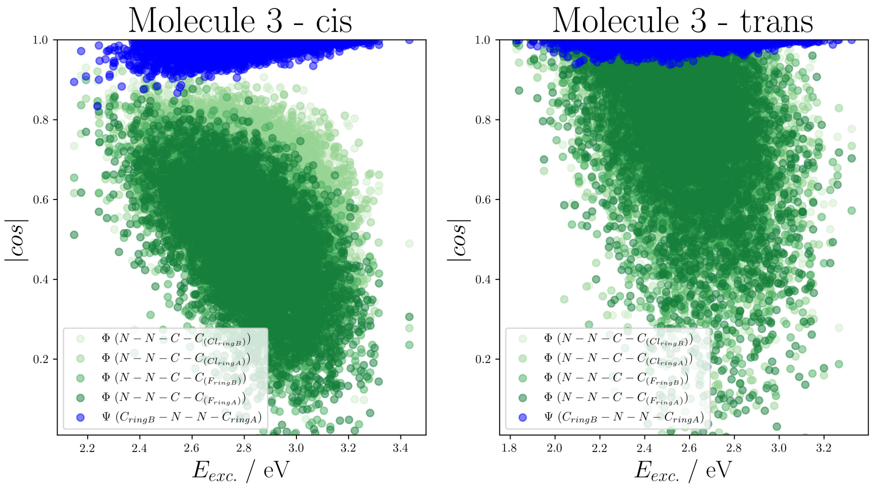

Figure S3.3. Distribution of all 6000 data points for *cis*- (left) and *trans*-conformer (right) of molecule 3.

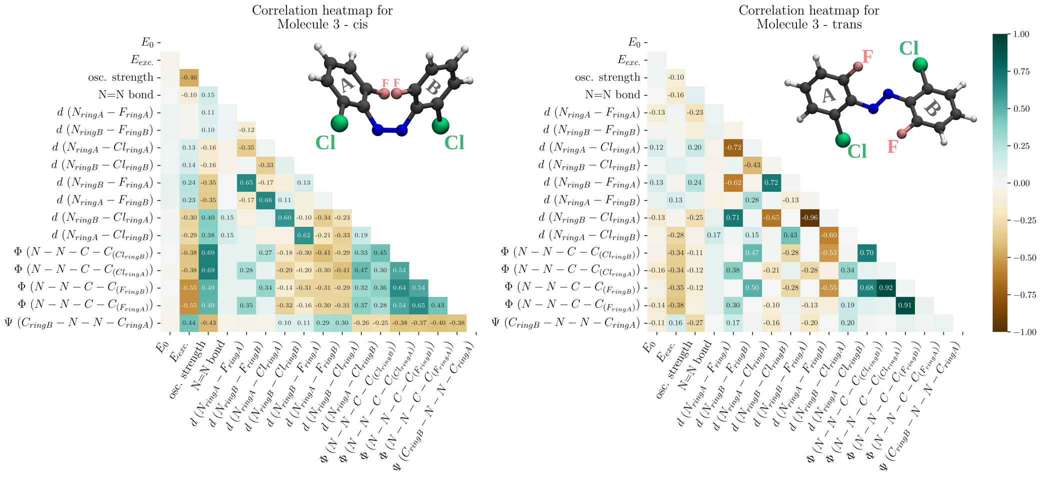

Figure S3.4. Correlation plots for *cis*- (left) and *trans*-conformers (right) of molecule 3.

2.3. Molecule 4

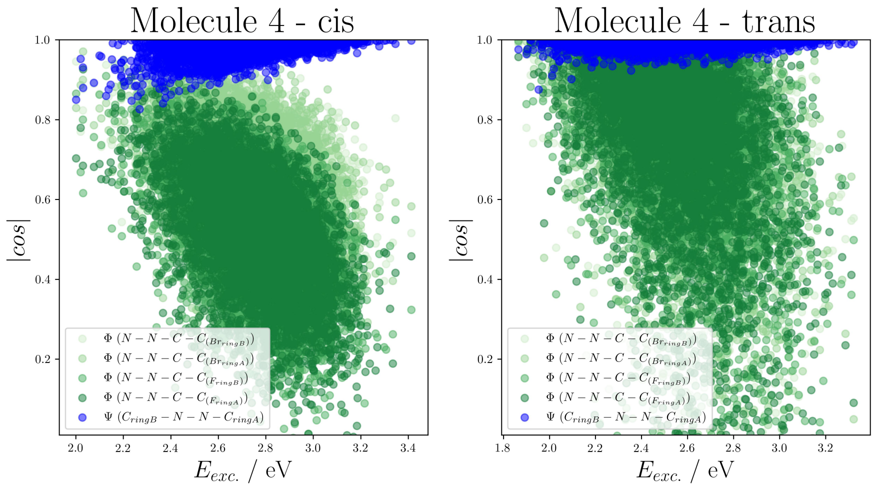

Figure S3.5. Distribution of all 6000 data points for *cis*- (left) and *trans*-conformer (right) of molecule 4.

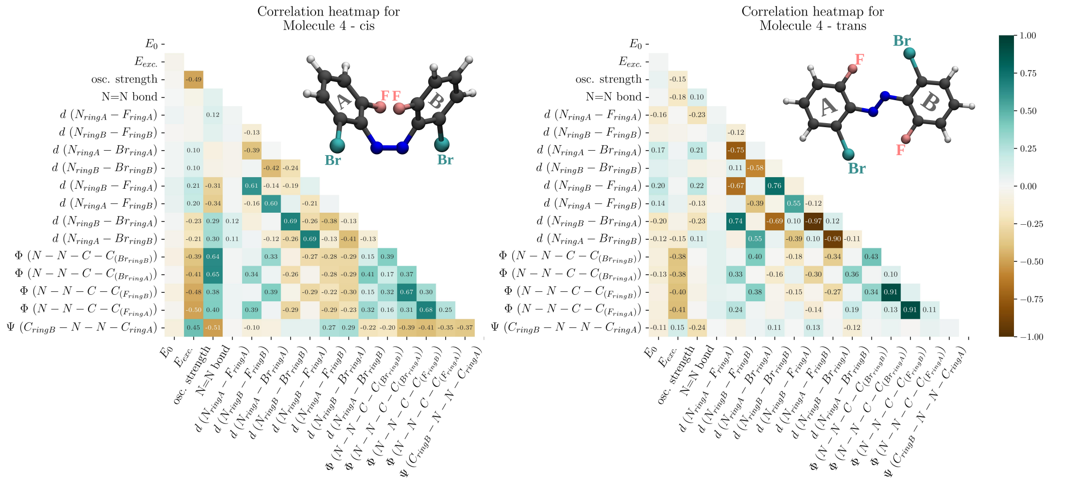

Figure S3.6. Correlation plots for *cis*- (left) and *trans*-conformers (right) of molecule 4.

2.4. Molecule 6

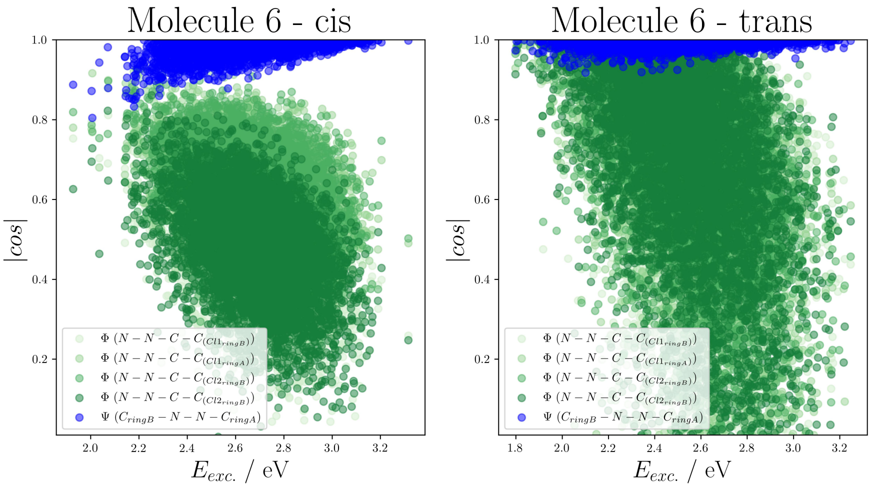

Figure S3.7. Distribution of all 6000 data points for *cis*- (left) and *trans*-conformer (right) of molecule 6.

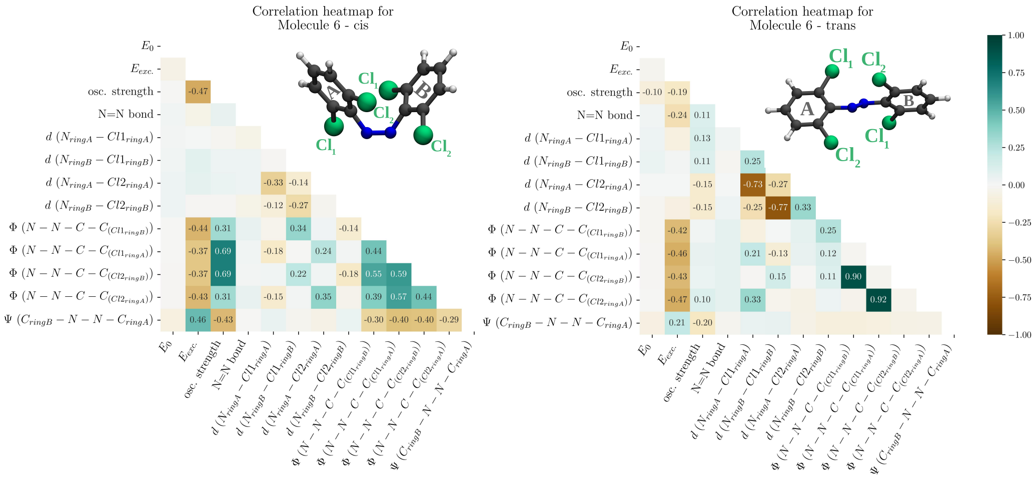

Figure S3.8. Correlation plots for *cis*- (left) and *trans*-conformers (right) of molecule 6.

2.5. Molecule 21

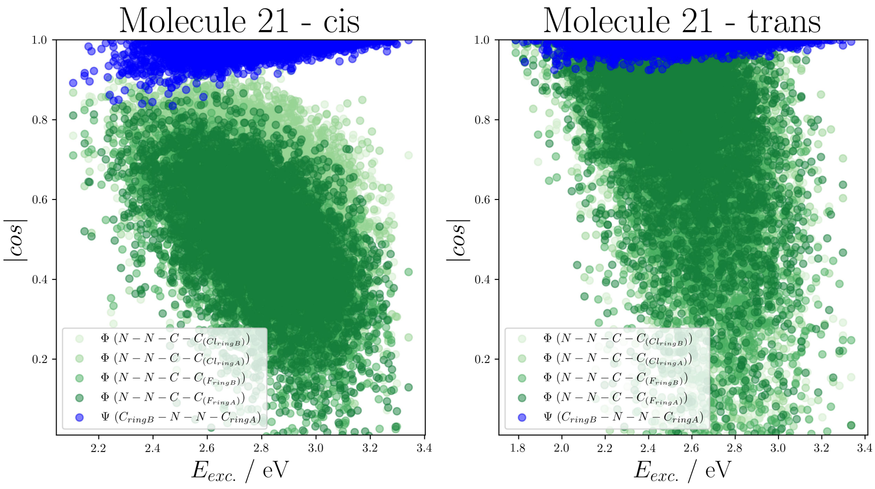

Figure S3.9. Distribution of all 6000 data points for *cis*- (left) and *trans*-conformer (right) of molecule 21.

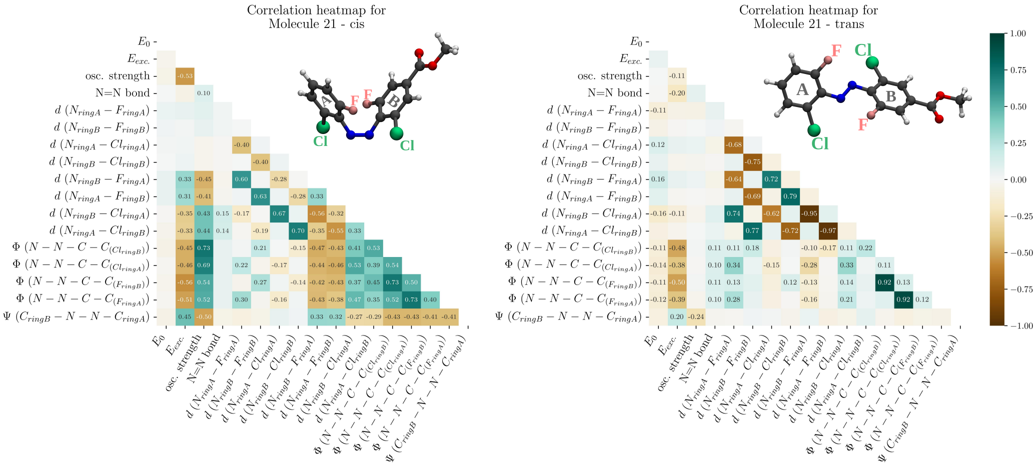

Figure S3.10. Correlation plots for *cis*- (left) and *trans*-conformers (right) of molecule 21.

2.6. Molecule 28

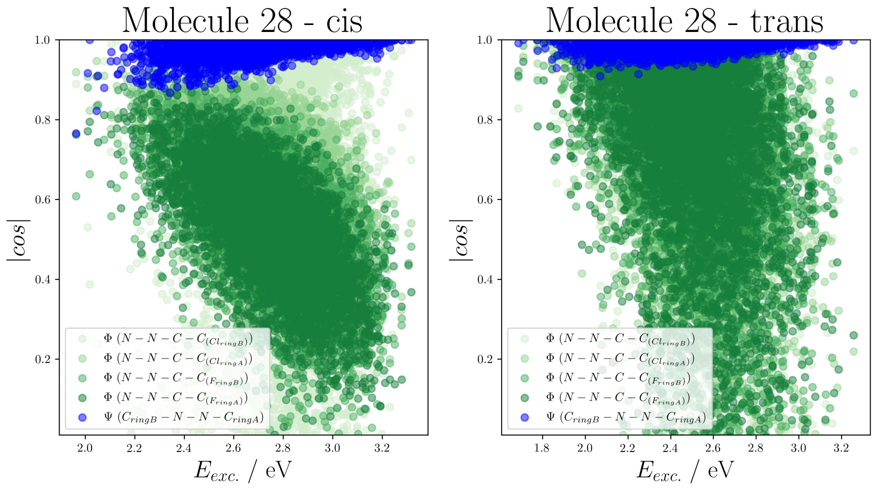

Figure S3.11. Distribution of all 6000 data points for *cis*- (left) and *trans*-conformer (right) of molecule 28.

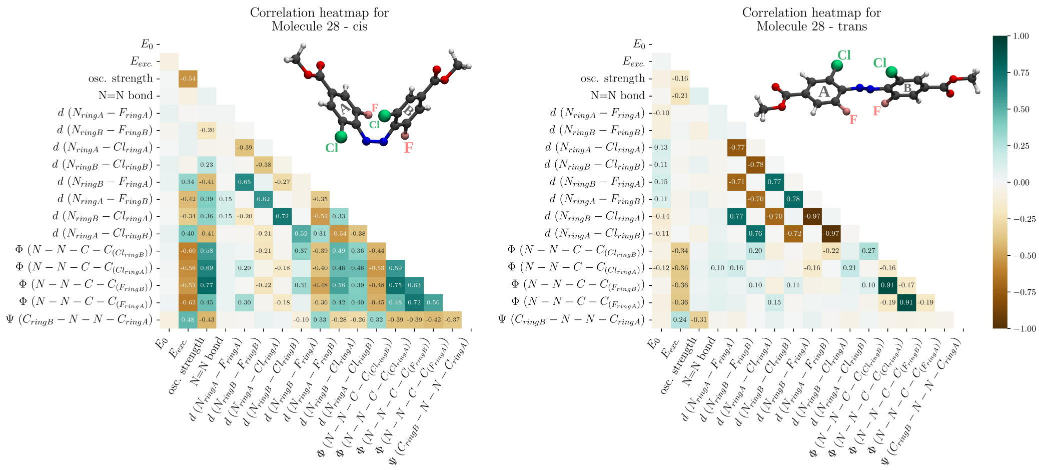

Figure S3.12. Correlation plots for *cis*- (left) and *trans*-conformers (right) of molecule 28.

2.7. Molecule 39

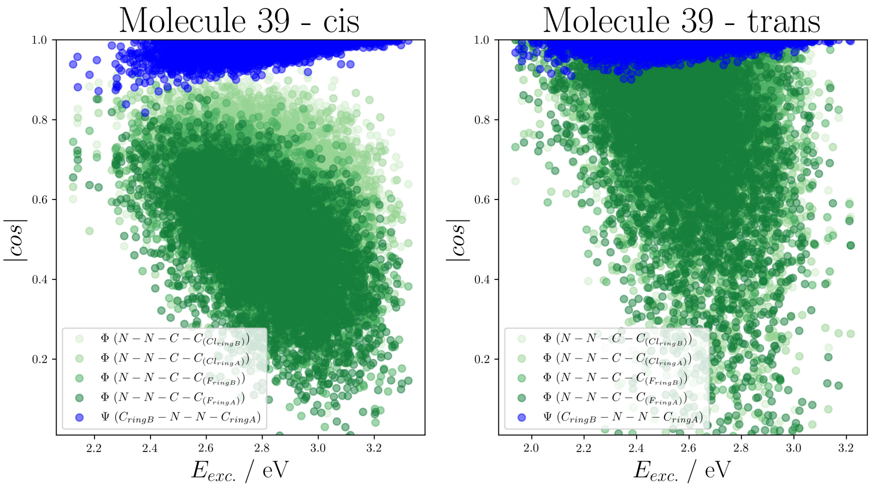

Figure S3.13. Distribution of all 6000 data points for *cis*- (left) and *trans*-conformer (right) of molecule 39.

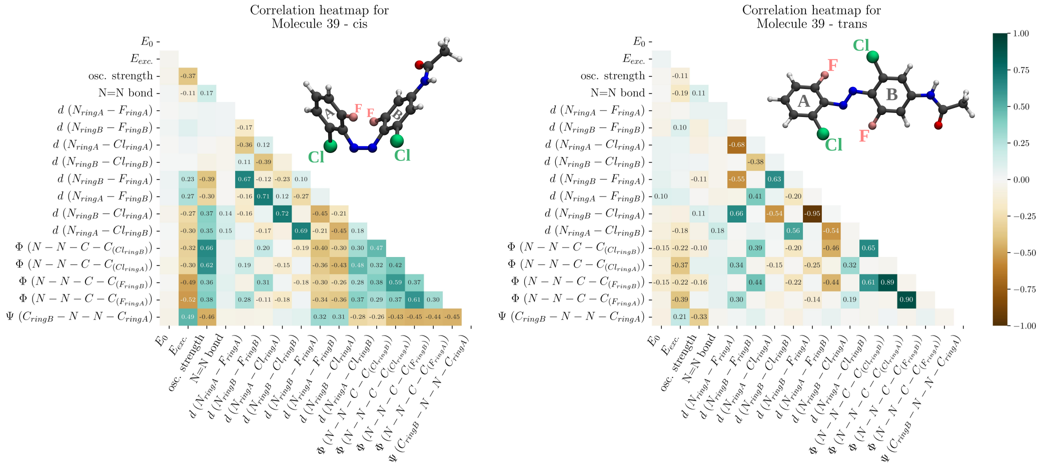

Figure S3.14. Correlation plots for *cis*- (left) and *trans*-conformers (right) of molecule 39.

2.8. Molecule 46

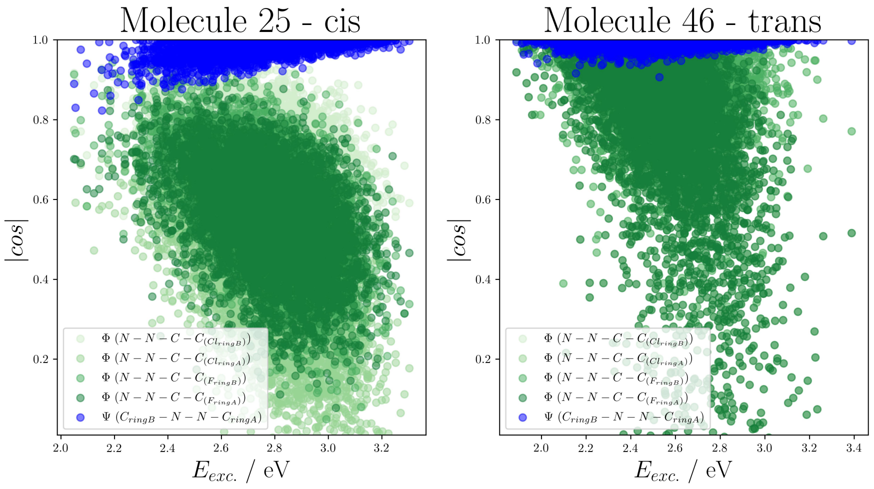

Figure S3.15. Distribution of all 6000 data points for *cis*- (left) and *trans*-conformer (right) of molecule 46.

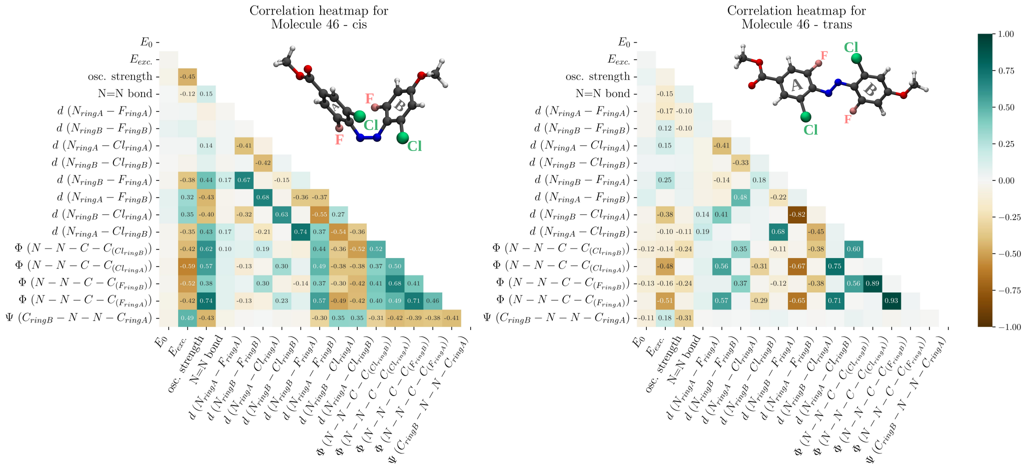

Figure S3.16. Correlation plots for *cis*- (left) and *trans*-conformers (right) of molecule 46.

3. Computed properties for each molecule in detail

This section provides a detailed summary of the computed properties and spectra for each individual molecule. Analogously to Table S3.3, the computed spectra were blue-shifted by $900\,cm^{-1}$ to better match the experimental spectra.

3.1. Molecule 1

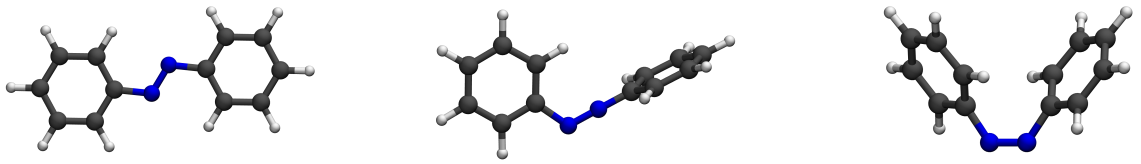

-11.5 27.2 0

Figure S3.17. 3D-Structures and relative energies in kcal/mol (in implicit solvent; DMSO; corrected for ZPVE; referenced to *cis*) of the energetically lowest *trans*- (left), transition state- (middle) *cis*- (right) conformer.

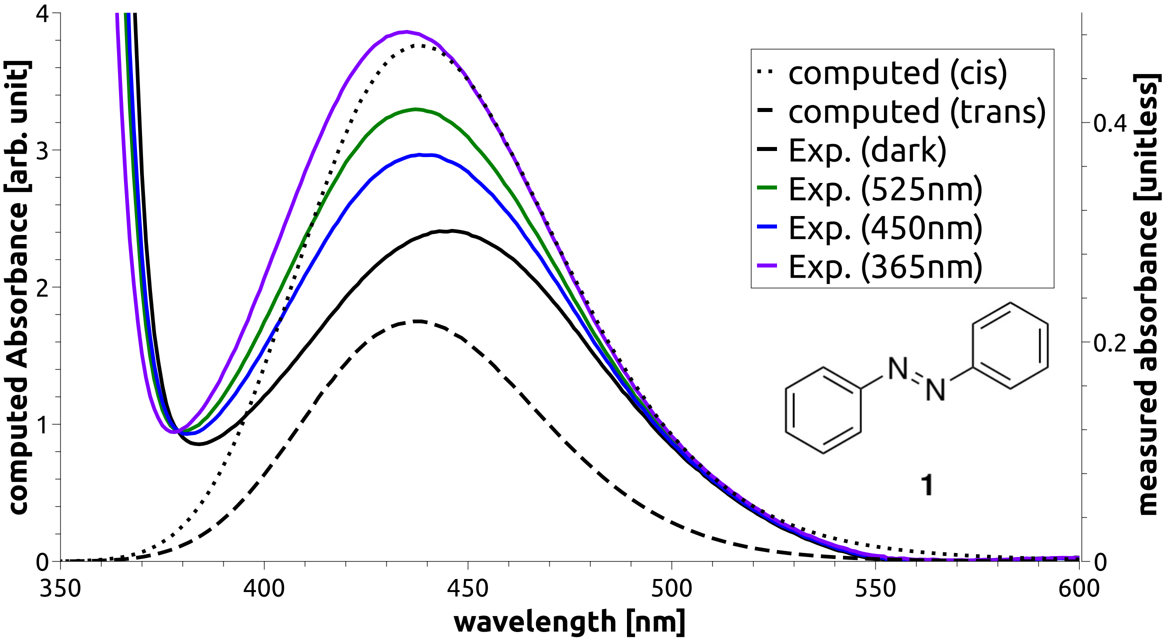

Figure S3.18. Computed absorption spectra (implicit solvent; DMSO; blue-shifted by 900 cm^{-1}) only including lowest energy ($n \rightarrow \pi^*$) excitation for *trans* and *cis* conformer and experimental spectra (500 µM irradiation times: 525 nm - 60 min; 450 nm - 15 min; 365 nm - 15 min).

3.2. Molecule 2

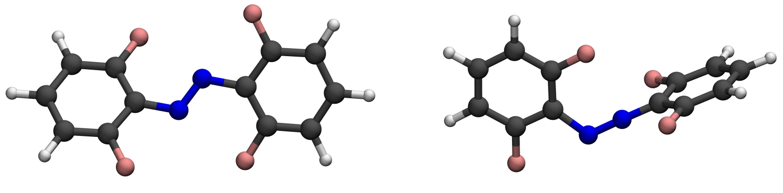

-6.3 32.2 0

Figure S3.19. 3D-Structures and relative energies in kcal/mol (in implicit solvent; DMSO; corrected for ZPVE; referenced to *cis*) of the energetically lowest *trans*- (left), transition state- (middle) *cis*- (right) conformer.

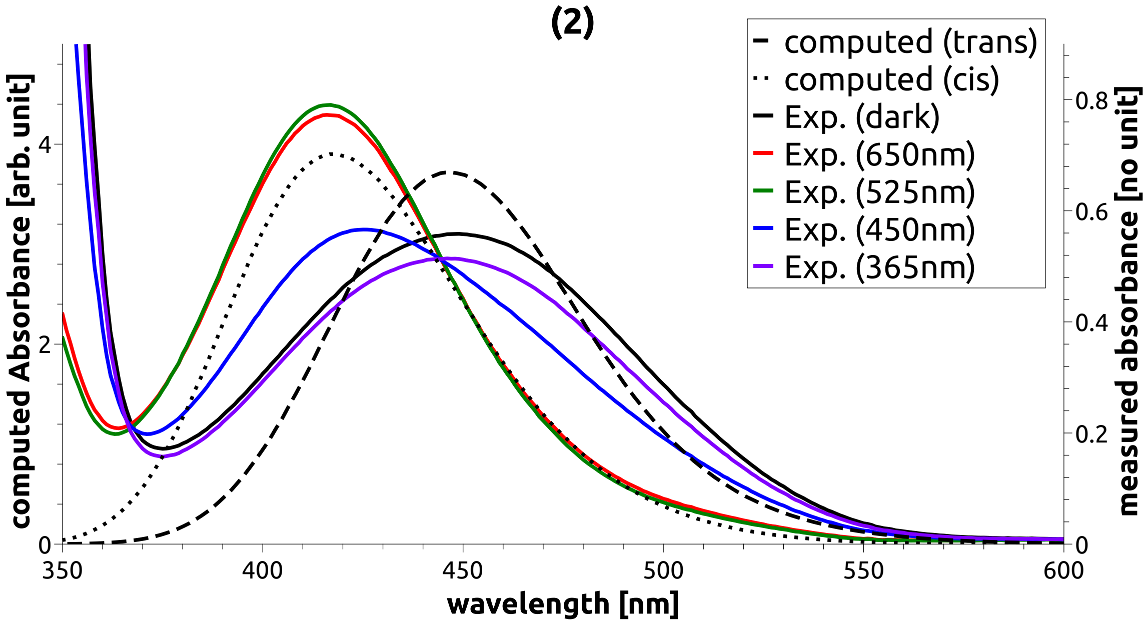

Figure S3.20. Computed absorption spectra (implicit solvent; DMSO; blue-shifted by 900 cm^{-1}) only including lowest energy ($n \rightarrow \pi^*$) excitation for *trans* and *cis* conformer and experimental spectra (500 µM irradiation times: 650 nm - 585 min; 525 nm - 15 min; 450 nm - 15 min; 365 nm - 15 min).

3.3. Molecule 3

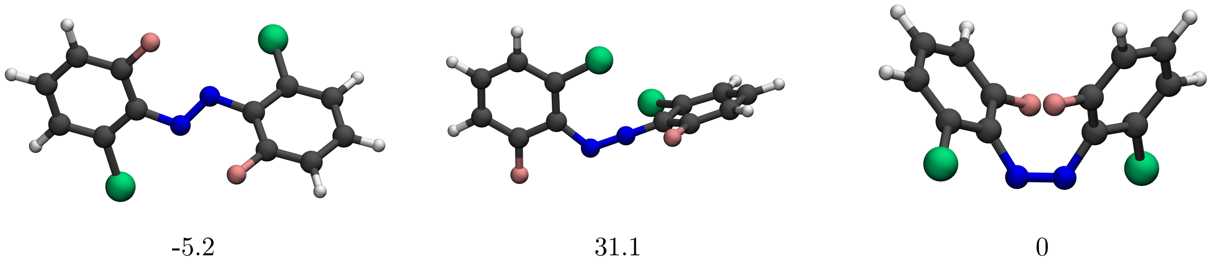

Figure S3.21. 3D-Structures and relative energies in kcal/mol (in implicit solvent; DMSO; corrected for ZPVE; referenced to *cis*) of the energetically lowest *trans*- (left), transition state- (middle) *cis*- (right) conformer.

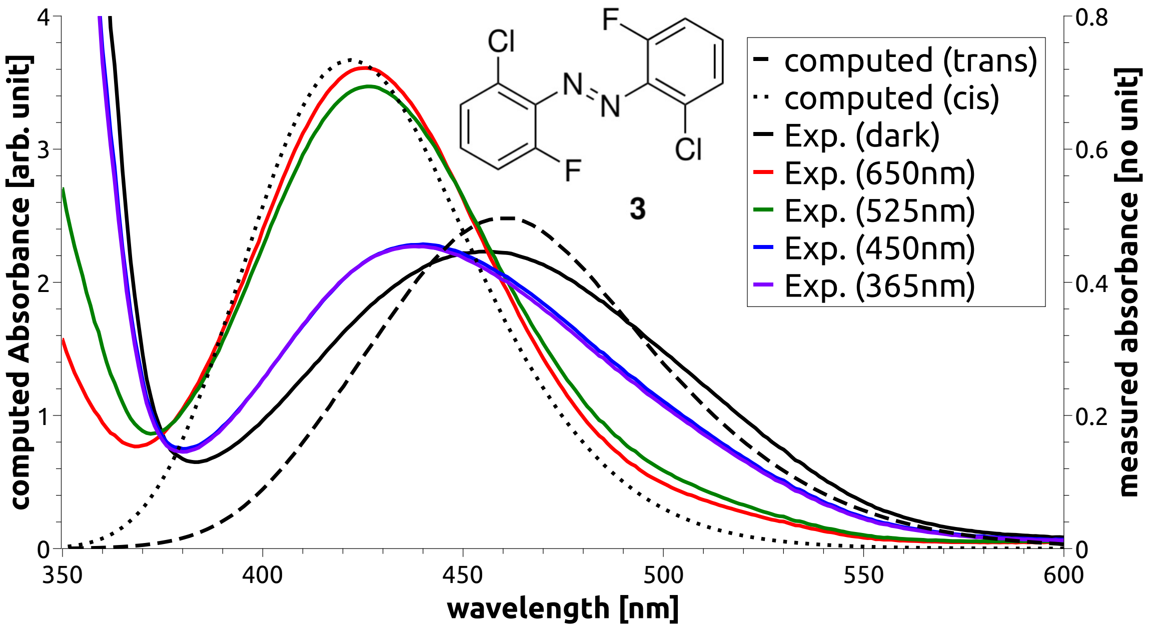

Figure S3.22. Computed absorption spectra (implicit solvent; DMSO; blue-shifted by 900 cm^{-1}) only including lowest energy ($n \rightarrow \pi^*$) excitation for *trans* and *cis* conformer and experimental spectra (500 µM irradiation times: 650 nm - 585 min; 525 nm - 15 min; 450 nm - 15 min; 365 nm - 15 min).

3.4. Molecule 4

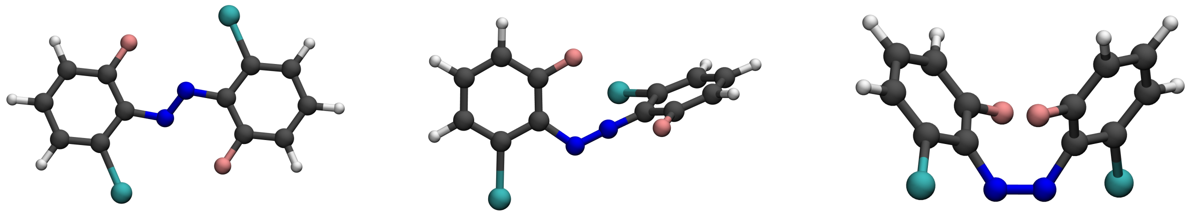

-5.2 30.6 0

Figure S3.23. 3D-Structures and relative energies in kcal/mol (in implicit solvent; DMSO; corrected for ZPVE; referenced to *cis*) of the energetically lowest *trans*- (left), transition state- (middle) *cis*- (right) conformer.

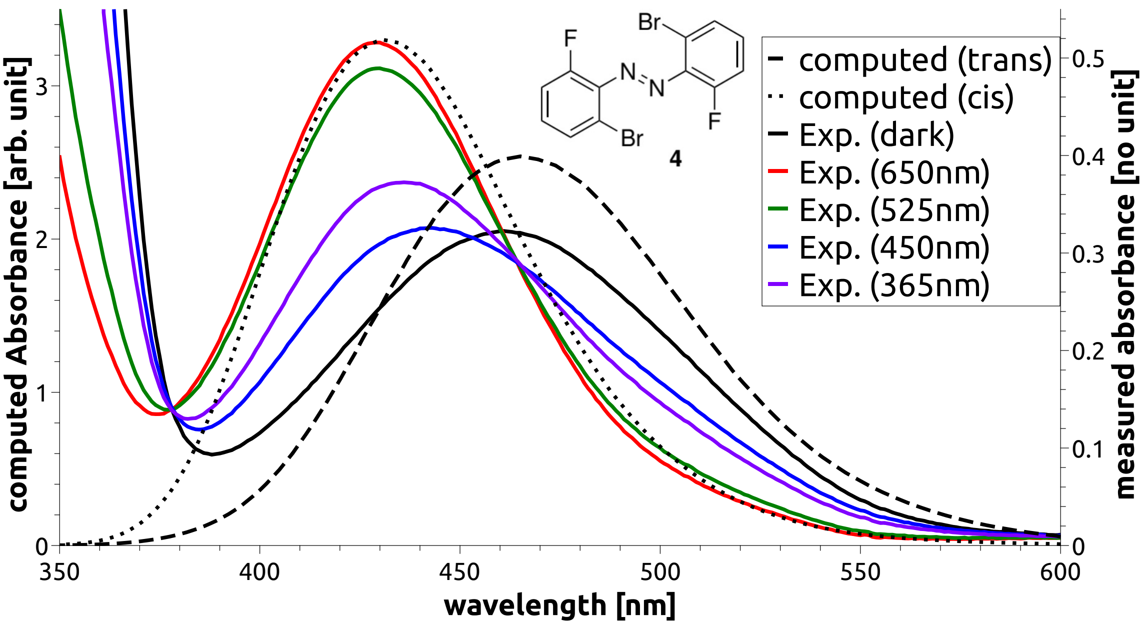

Figure S3.24. Computed absorption spectra (implicit solvent; DMSO; blue-shifted by 900 cm^{-1}) only including lowest energy ($n \rightarrow \pi^*$) excitation for *trans* and *cis* conformer and experimental spectra (500 µM irradiation times: 650 nm - 585 min; 525 nm - 15 min; 450 nm - 15 min; 365 nm - 15 min).

3.5. Molecule 5

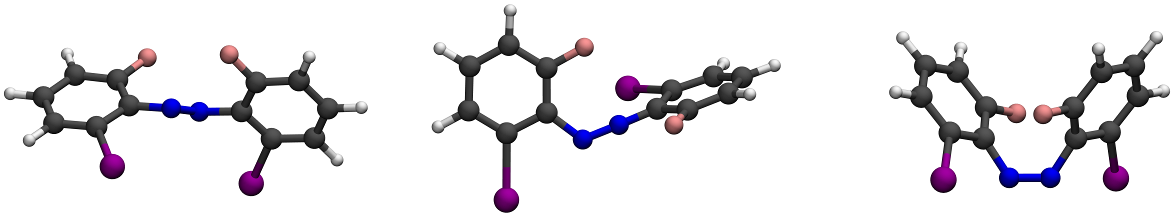

-5.2 30.5 0

Figure S3.25. 3D-Structures and relative energies in kcal/mol (in implicit solvent; DMSO; corrected for ZPVE; referenced to *cis*) of the energetically lowest *trans*- (left), transition state- (middle) *cis*- (right) conformer.

3.6. Molecule 6

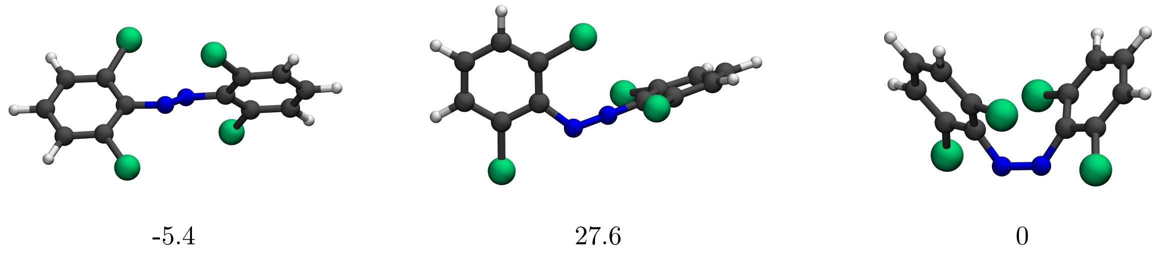

Figure S3.26. 3D-Structures and relative energies in kcal/mol (in implicit solvent; DMSO; corrected for ZPVE; referenced to *cis*) of the energetically lowest *trans*- (left), transition state- (middle) *cis*- (right) conformer.

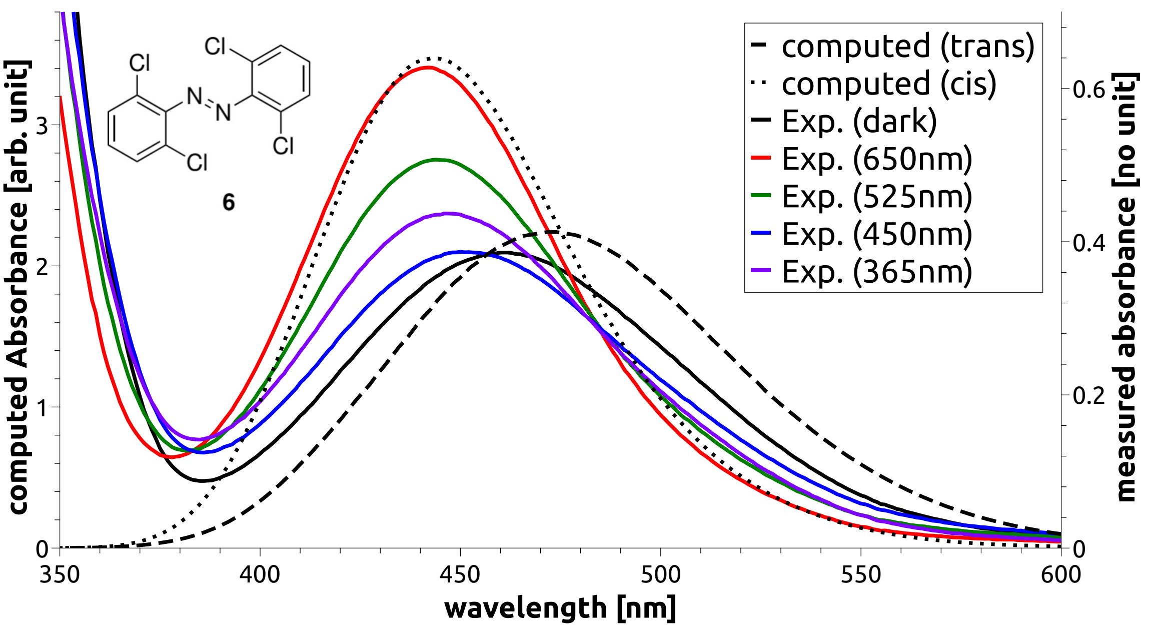

Figure S3.27. Computed absorption spectra (implicit solvent; DMSO; blue-shifted by 900 cm^{-1}) only including lowest energy ($n \rightarrow \pi^*$) excitation for *trans* and *cis* conformer and experimental spectra (500 µM irradiation times: 650 nm - 585 min; 525 nm - 15 min; 450 nm - 15 min; 365 nm - 15 min).

3.7. Molecule 7

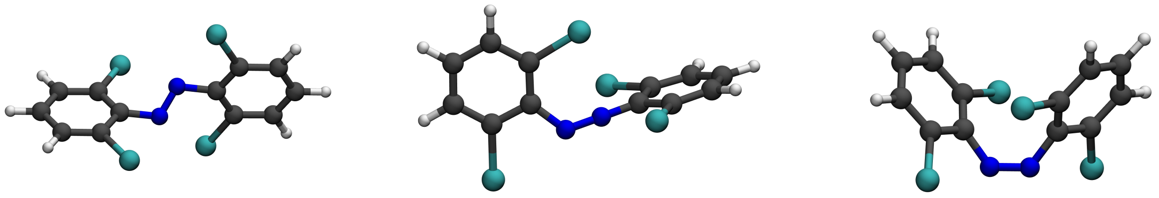

-7.9 25.3 0

Figure S3.28. 3D-Structures and relative energies in kcal/mol (in implicit solvent; DMSO; corrected for ZPVE; referenced to *cis*) of the energetically lowest *trans*- (left), transition state- (middle) *cis*- (right) conformer.

3.8. Molecule 8

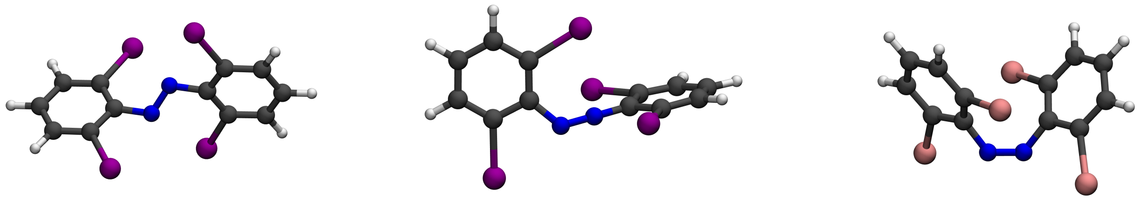

-11.8 | 22.4 | 0

Figure S3.29. 3D-Structures and relative energies in kcal/mol (in implicit solvent; DMSO; corrected for ZPVE; referenced to *cis*) of the energetically lowest *trans*- (left), transition state- (middle) *cis*- (right) conformer.

3.9. Molecule 9

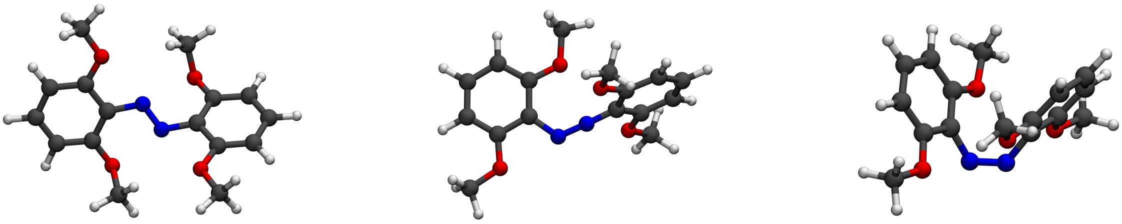

-2.9 34.4 0

Figure S3.30. 3D-Structures and relative energies in kcal/mol (in implicit solvent; DMSO; corrected for ZPVE; referenced to *cis*) of the energetically lowest *trans*- (left), transition state- (middle) *cis*- (right) conformer.

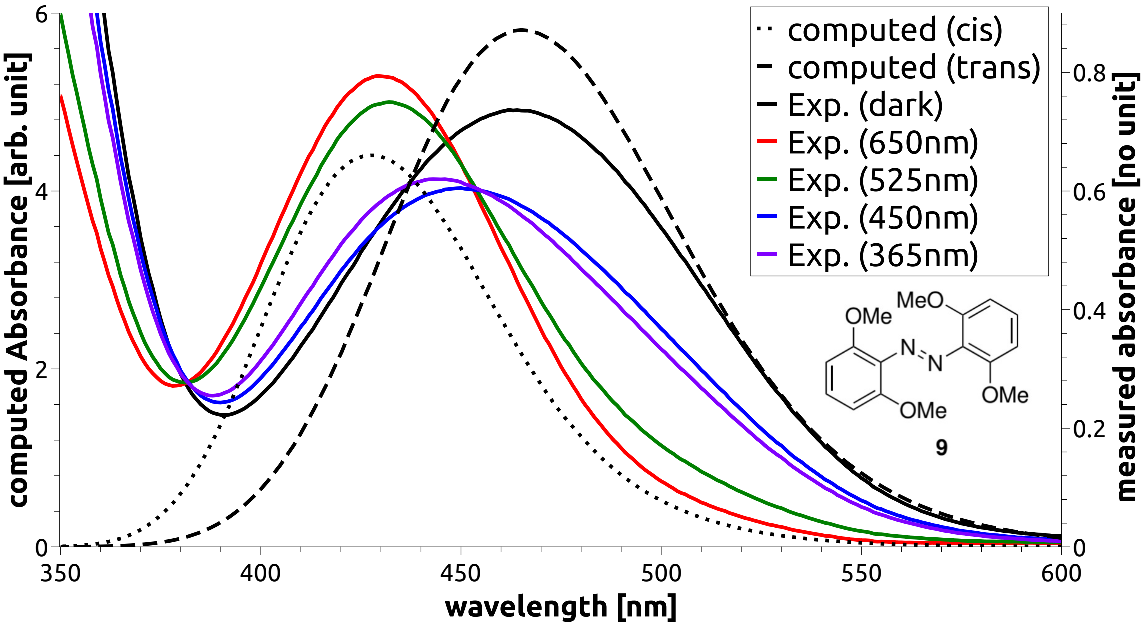

Figure S3.31. Computed absorption spectra (implicit solvent; DMSO; blue-shifted by 900 cm^{-1}) only including lowest energy ($n \rightarrow \pi^*$) excitation for *trans* and *cis* conformer and experimental spectra (500 µM irradiation times: 650 nm - 585 min; 525 nm - 15 min; 450 nm - 15 min; 365 nm - 15 min).

3.10. Molecule 10

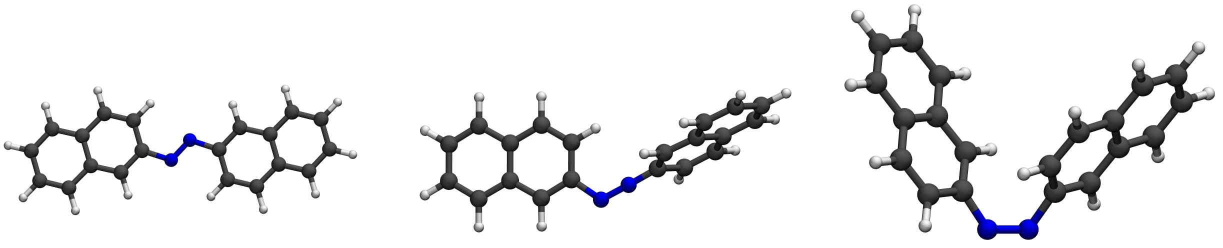

-12.7 27.5 0

Figure S3.32. 3D-Structures and relative energies in kcal/mol (in implicit solvent; DMSO; corrected for ZPVE; referenced to *cis*) of the energetically lowest *trans*- (left), transition state- (middle) *cis*- (right) conformer.

3.11. Molecule 11

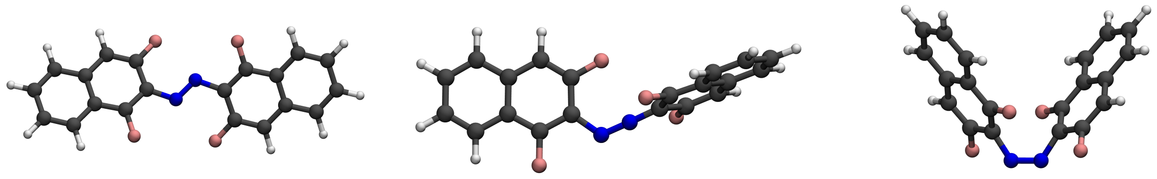

-6.5 31.5 0

Figure S3.33. 3D-Structures and relative energies in kcal/mol (in implicit solvent; DMSO; corrected for ZPVE; referenced to *cis*) of the energetically lowest *trans*- (left), transition state- (middle) *cis*- (right) conformer.

3.12. Molecule 12

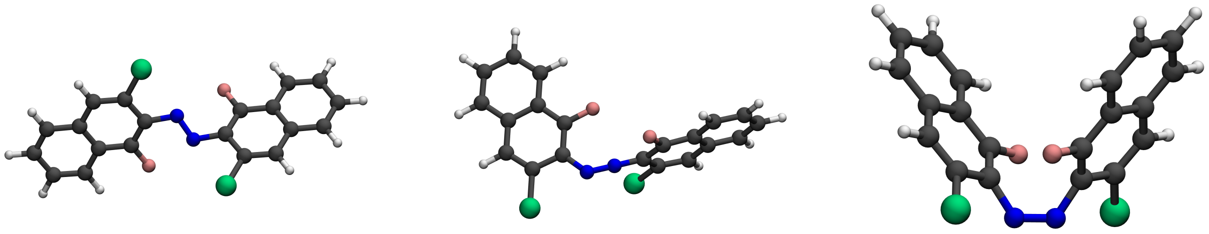

Figure S3.34. 3D-Structures and relative energies in kcal/mol (in implicit solvent; DMSO; corrected for ZPVE; referenced to *cis*) of the energetically lowest *trans*- (left), transition state- (middle) *cis*- (right) conformer.

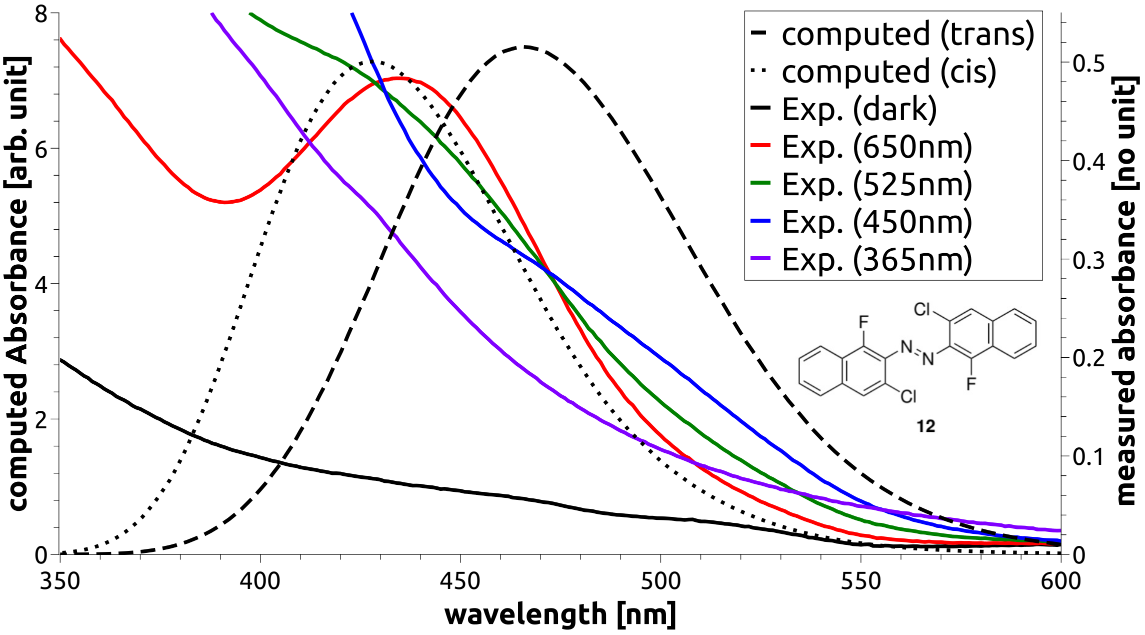

Figure S3.35. Computed absorption spectra (implicit solvent; DMSO; blue-shifted by 900 cm^{-1}) only including lowest energy ($n \to \pi^*$) excitation for *trans* and *cis* conformer and experimental spectra (500 µM irradiation times: 650 nm - 585 min; 525 nm - 15 min; 450 nm - 15 min; 365 nm - 15 min).

3.13. Molecule 13

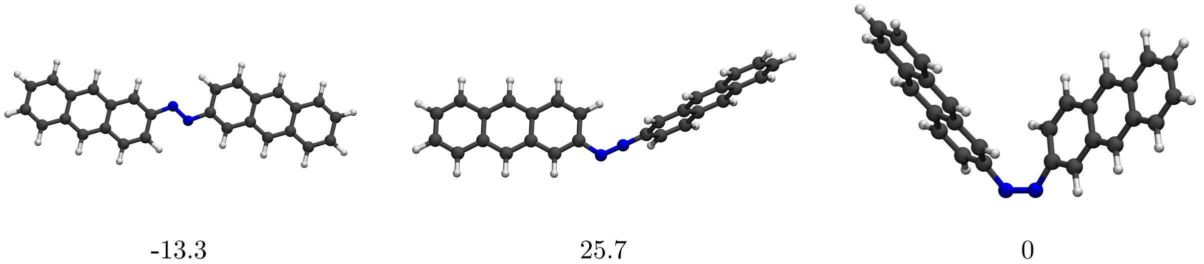

Figure S3.36. 3D-Structures and relative energies in kcal/mol (in implicit solvent; DMSO; corrected for ZPVE; referenced to *cis*) of the energetically lowest *trans*- (left), transition state- (middle) *cis*- (right) conformer.

3.14. Molecule 14

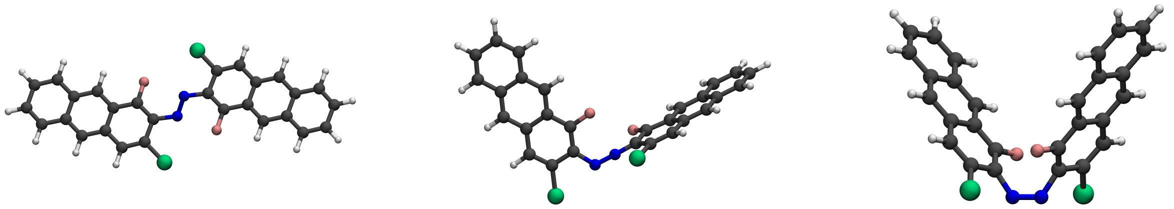

-5.1 30.9 0

Figure S3.37. 3D-Structures and relative energies in kcal/mol (in implicit solvent; DMSO; corrected for ZPVE; referenced to *cis*) of the energetically lowest *trans*- (left), transition state- (middle) *cis*- (right) conformer.

3.15. Molecule 15

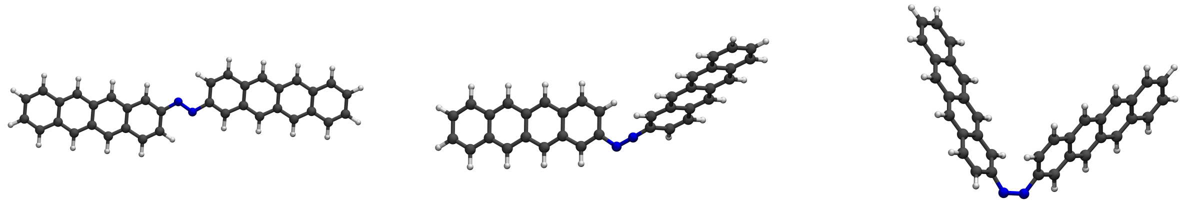

-13.6 25.4 0

Figure S3.38. 3D-Structures and relative energies in kcal/mol (in implicit solvent; DMSO; corrected for ZPVE; referenced to *cis*) of the energetically lowest *trans*- (left), transition state- (middle) *cis*- (right) conformer.

3.16. Molecule 16

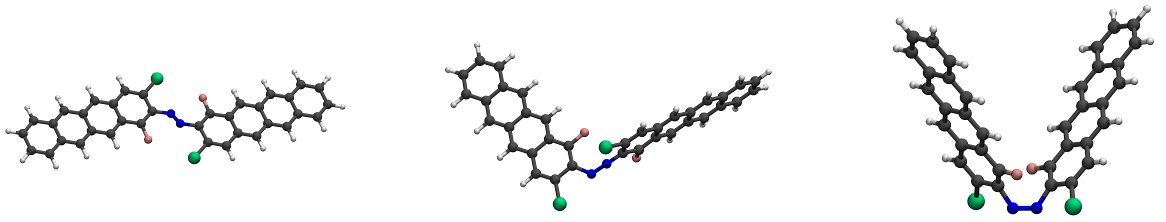

-4.9 30.9 0

Figure S3.39. 3D-Structures and relative energies in kcal/mol (in implicit solvent; DMSO; corrected for ZPVE; referenced to *cis*) of the energetically lowest *trans*- (left), transition state- (middle) *cis*- (right) conformer.

3.17. Molecule 17

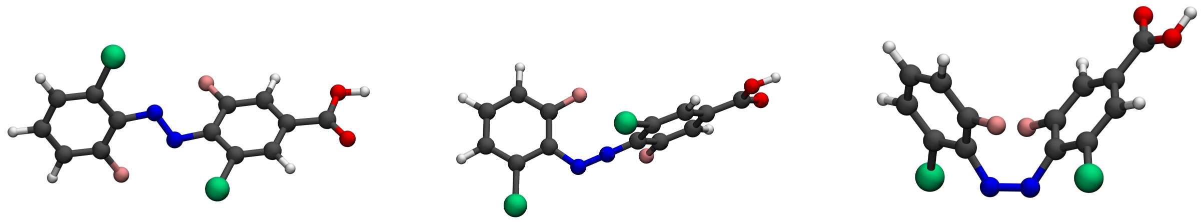

-5.0 26.2 0

Figure S3.40. 3D-Structures and relative energies in kcal/mol (in implicit solvent; DMSO; corrected for ZPVE; referenced to *cis*) of the energetically lowest *trans*- (left), transition state- (middle) *cis*- (right) conformer.

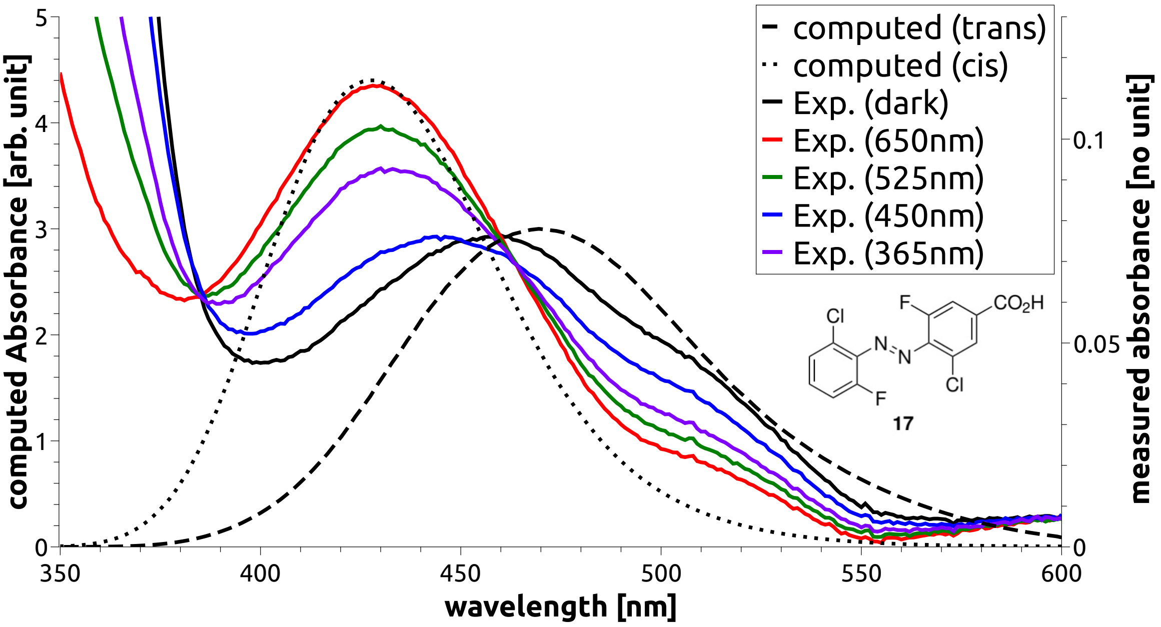

Figure S3.41. Computed absorption spectra (implicit solvent; DMSO; blue-shifted by 900 cm^{-1}) only including lowest energy ($n \rightarrow \pi^*$) excitation for *trans* and *cis* conformer and experimental spectra (500 µM irradiation times: 650 nm - 585 min; 525 nm - 15 min; 450 nm - 15 min; 365 nm - 15 min).

3.18. Molecule 18

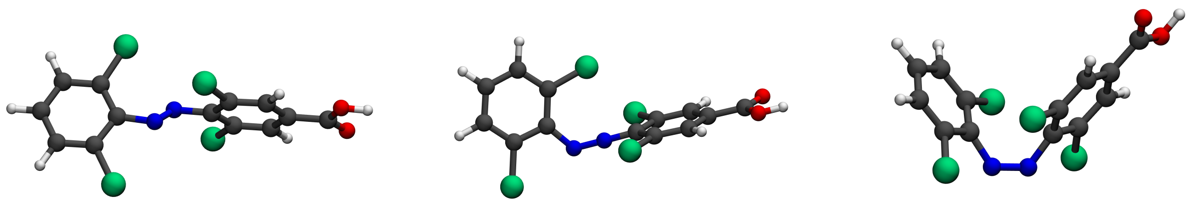

-5.3 23.4 0

Figure S3.42. 3D-Structures and relative energies in kcal/mol (in implicit solvent; DMSO; corrected for ZPVE; referenced to *cis*) of the energetically lowest *trans*- (left), transition state- (middle) *cis*- (right) conformer.

3.19. Molecule 19

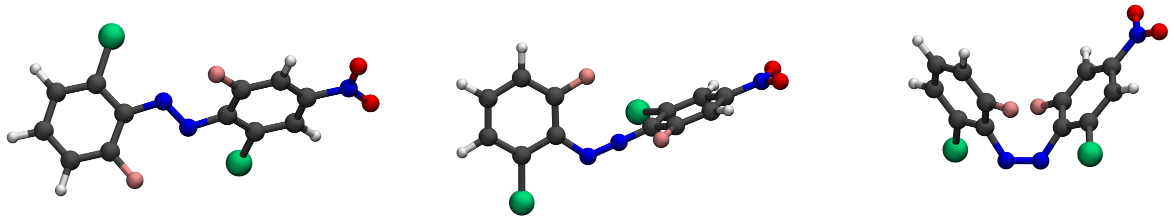

-5.1 23.4 0

Figure S3.43. 3D-Structures and relative energies in kcal/mol (in implicit solvent; DMSO; corrected for ZPVE; referenced to *cis*) of the energetically lowest *trans*- (left), transition state- (middle) *cis*- (right) conformer.

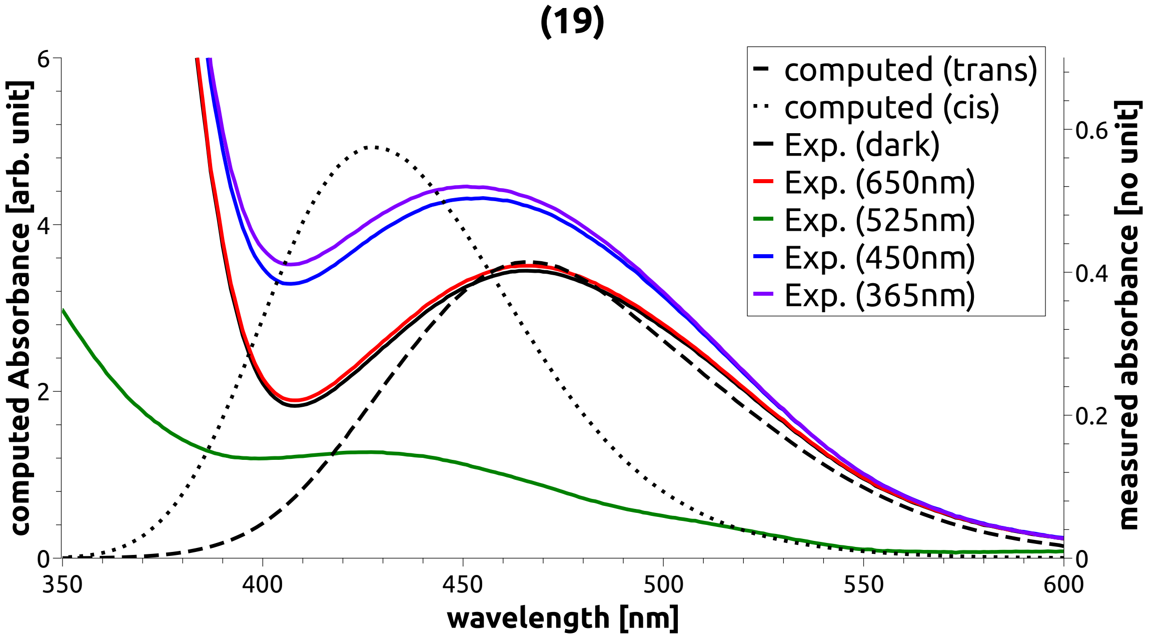

Figure S3.44. Computed absorption spectra (implicit solvent; DMSO; blue-shifted by 900 cm^{-1}) only including lowest energy ($n \to \pi^*$) excitation for *trans* and *cis* conformer and experimental spectra (500 µM irradiation times: 650 nm - 585 min; 525 nm - 15 min; 450 nm - 15 min; 365 nm - 15 min).

3.20. Molecule 20

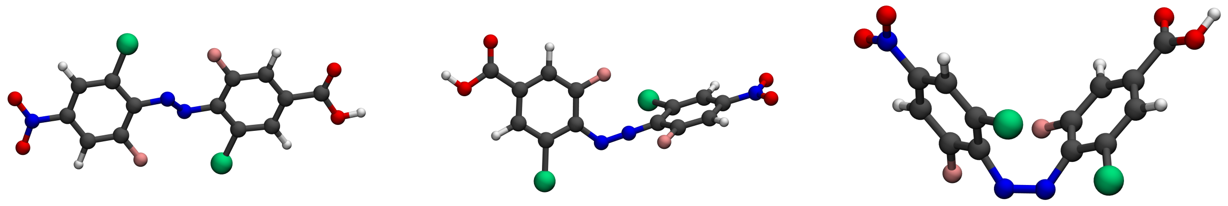

-5.6 24.1 0

Figure S3.45. 3D-Structures and relative energies in kcal/mol (in implicit solvent; DMSO; corrected for ZPVE; referenced to *cis*) of the energetically lowest *trans*- (left), transition state- (middle) *cis*- (right) conformer.

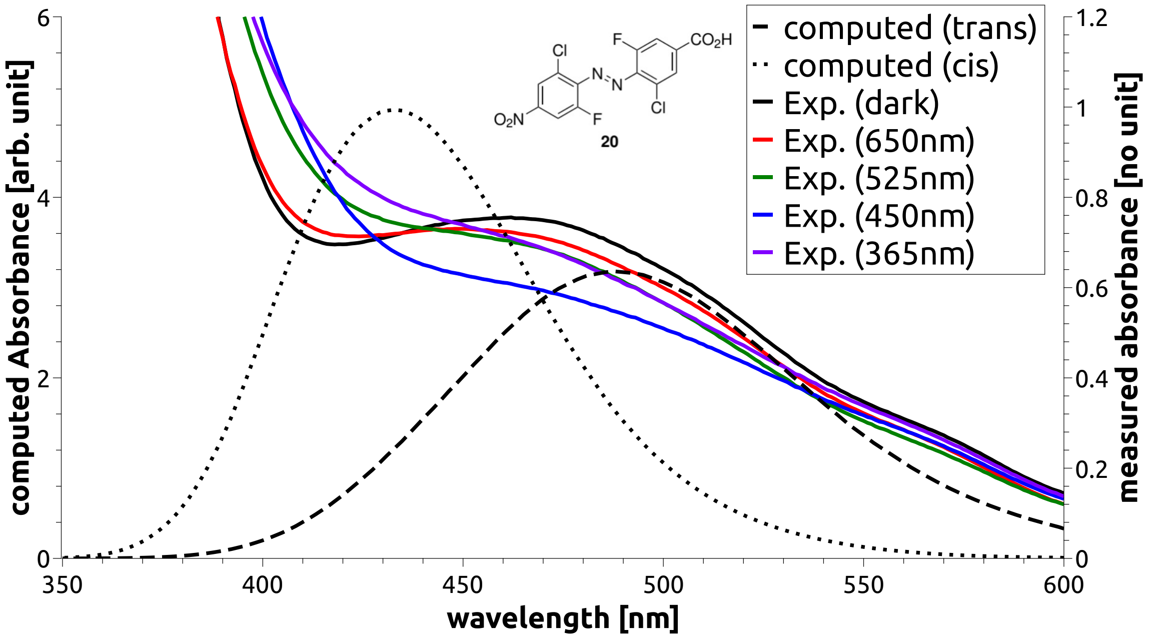

Figure S3.46. Computed absorption spectra (implicit solvent; DMSO; blue-shifted by 900 cm^{-1}) only including lowest energy ($n \rightarrow \pi^*$) excitation for *trans* and *cis* conformer and experimental spectra (500 µM irradiation times: 650 nm - 585 min; 525 nm - 15 min; 450 nm - 15 min; 365 nm - 15 min).

3.21. Molecule 21

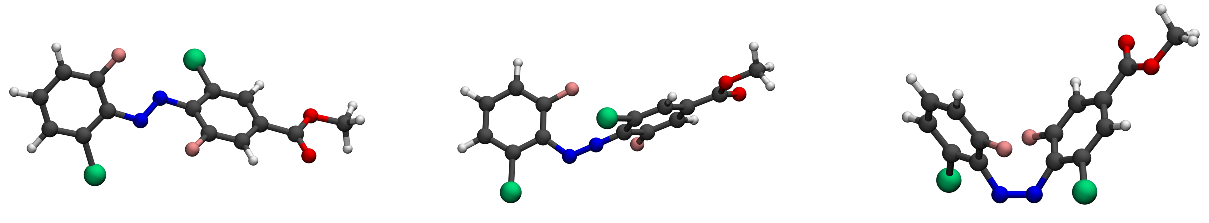

Figure S3.47. 3D-Structures and relative energies in kcal/mol (in implicit solvent; DMSO; corrected for ZPVE; referenced to *cis*) of the energetically lowest *trans*- (left), transition state- (middle) *cis*- (right) conformer.

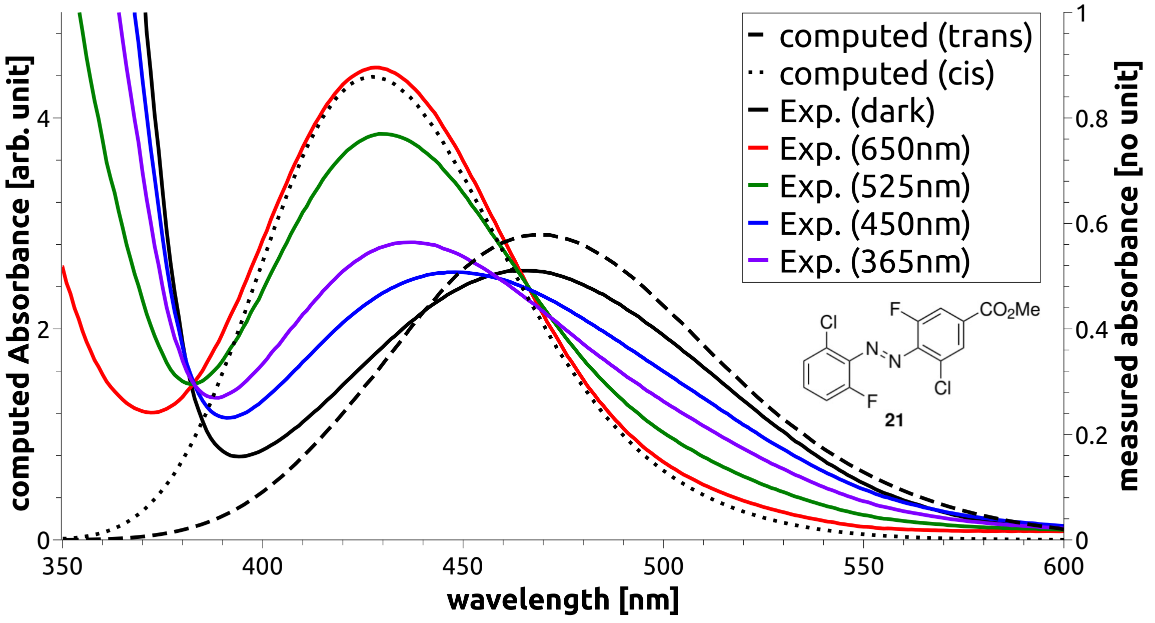

Figure S3.48. Computed absorption spectra (implicit solvent; DMSO; blue-shifted by 900 cm^{-1}) only including lowest energy ($n \rightarrow \pi^*$) excitation for *trans* and *cis* conformer and experimental spectra (500 µM irradiation times: 650 nm - 585 min; 525 nm - 15 min; 450 nm - 15 min; 365 nm - 15 min).

3.22. Molecule 22

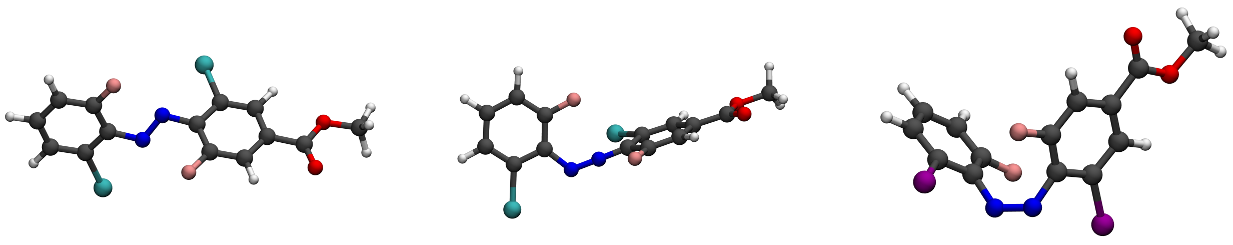

-5.4 26.1 0

Figure S3.49. 3D-Structures and relative energies in kcal/mol (in implicit solvent; DMSO; corrected for ZPVE; referenced to *cis*) of the energetically lowest *trans*- (left), transition state- (middle) *cis*- (right) conformer.

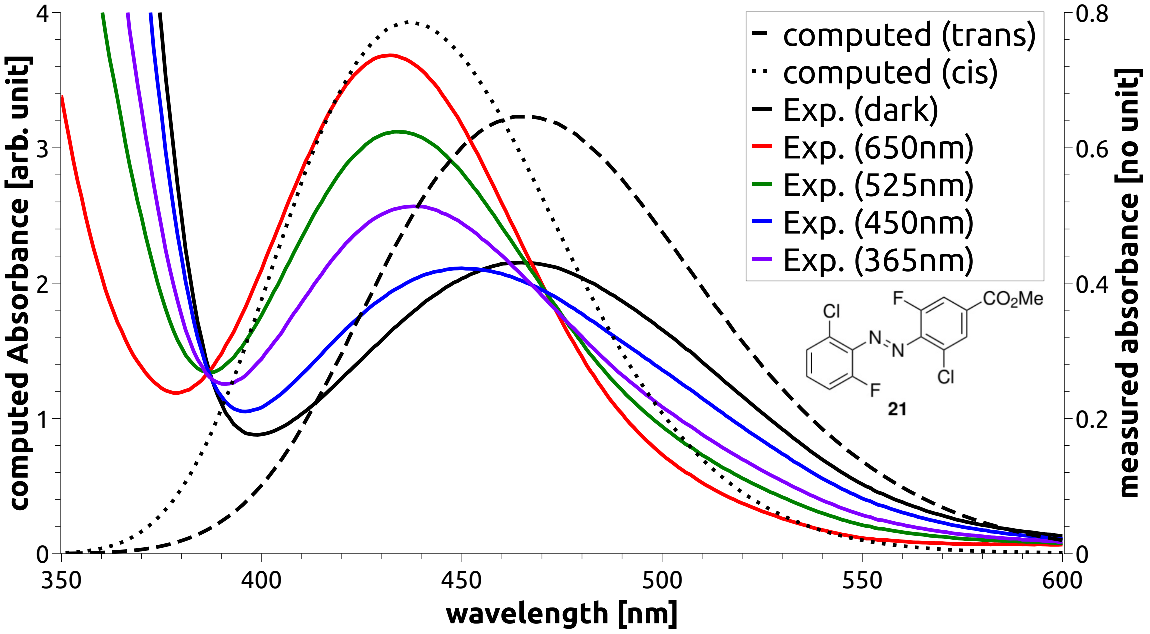

Figure S3.50. Computed absorption spectra (implicit solvent; DMSO; blue-shifted by 900 cm^{-1}) only including lowest energy ($n \rightarrow \pi^*$) excitation for *trans* and *cis* conformer and experimental spectra (500 μM irradiation times: 650 nm - 585 min; 525 nm - 15 min; 450 nm - 15 min; 365 nm - 15 min).

3.23. Molecule 23

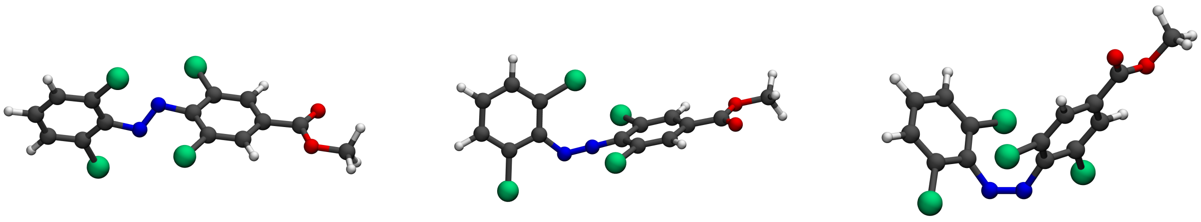

-5.4 23.5 0

Figure S3.51. 3D-Structures and relative energies in kcal/mol (in implicit solvent; DMSO; corrected for ZPVE; referenced to *cis*) of the energetically lowest *trans*- (left), transition state- (middle) *cis*- (right) conformer.

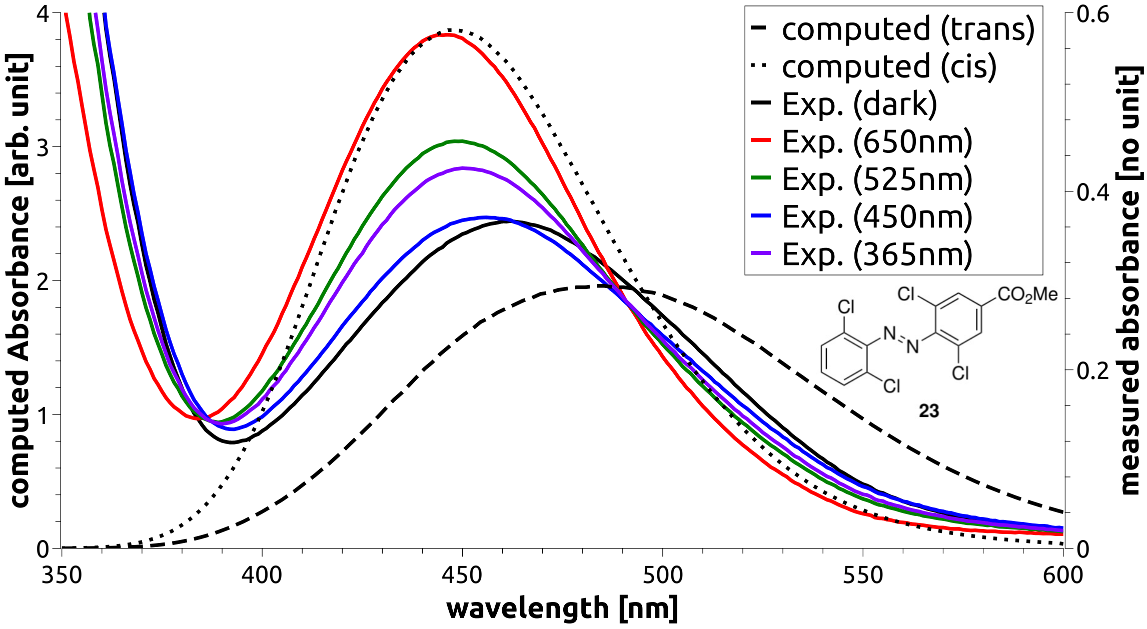

Figure S3.52. Computed absorption spectra (implicit solvent; DMSO; blue-shifted by 900 cm^{-1}) only including lowest energy ($n \rightarrow \pi^*$) excitation for *trans* and *cis* conformer and experimental spectra (500 µM irradiation times: 650 nm - 585 min; 525 nm - 15 min; 450 nm - 15 min; 365 nm - 15 min).

3.24. Molecule 24

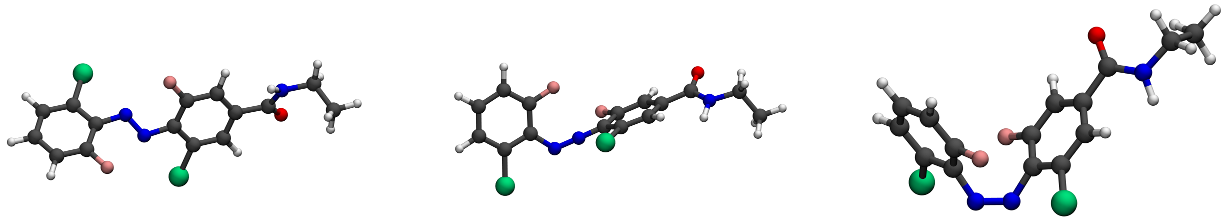

-5.0 28.7 0

Figure S3.53. 3D-Structures and relative energies in kcal/mol (in implicit solvent; DMSO; corrected for ZPVE; referenced to *cis*) of the energetically lowest *trans*- (left), transition state- (middle) *cis*- (right) conformer.

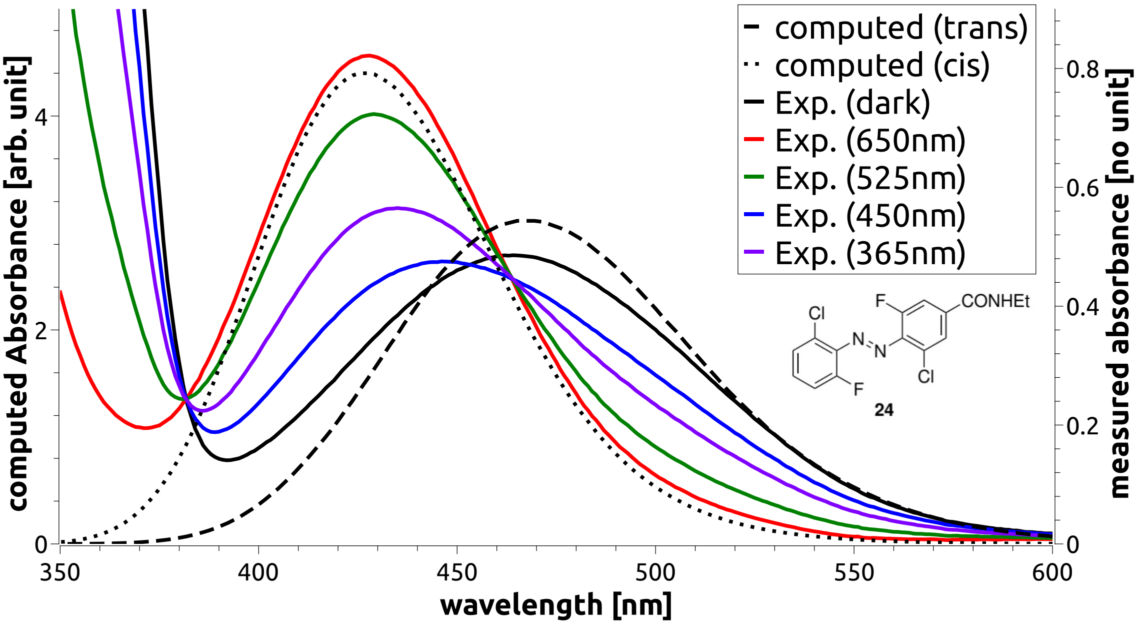

Figure S3.54. Computed absorption spectra (implicit solvent; DMSO; blue-shifted by 900 cm^{-1}) only including lowest energy ($n \rightarrow \pi^*$) excitation for *trans* and *cis* conformer and experimental spectra (500 µM irradiation times: 650 nm - 585 min; 525 nm - 15 min; 450 nm - 15 min; 365 nm - 15 min).

3.25. Molecule 25

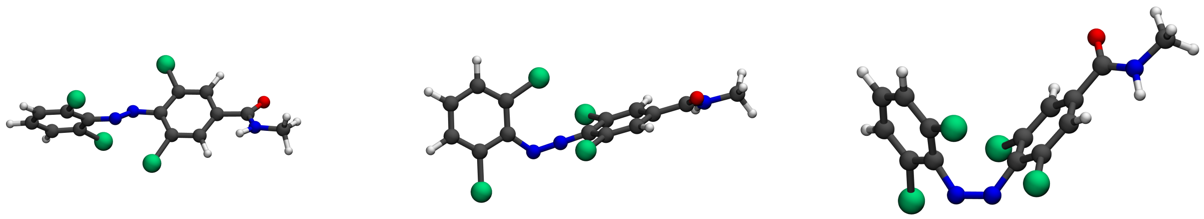

-5.5 26.2 0

Figure S3.55. 3D-Structures and relative energies in kcal/mol (in implicit solvent; DMSO; corrected for ZPVE; referenced to *cis*) of the energetically lowest *trans*- (left), transition state- (middle) *cis*- (right) conformer.

3.26. Molecule 26

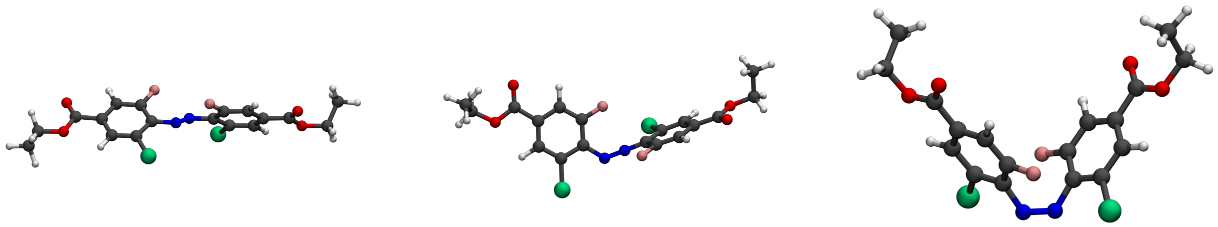

-4.6 27.9 0

Figure S3.56. 3D-Structures and relative energies in kcal/mol (in implicit solvent; DMSO; corrected for ZPVE; referenced to *cis*) of the energetically lowest *trans*- (left), transition state- (middle) *cis*- (right) conformer.

3.27. Molecule 27

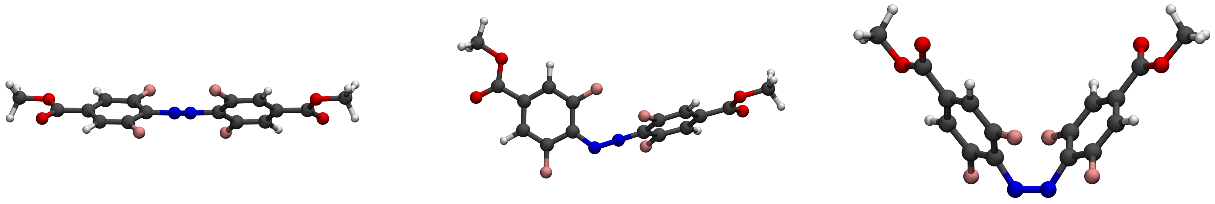

-5.9 28.7 0

Figure S3.57. 3D-Structures and relative energies in kcal/mol (in implicit solvent; DMSO; corrected for ZPVE; referenced to *cis*) of the energetically lowest *trans*- (left), transition state- (middle) *cis*- (right) conformer.

3.28. Molecule 28

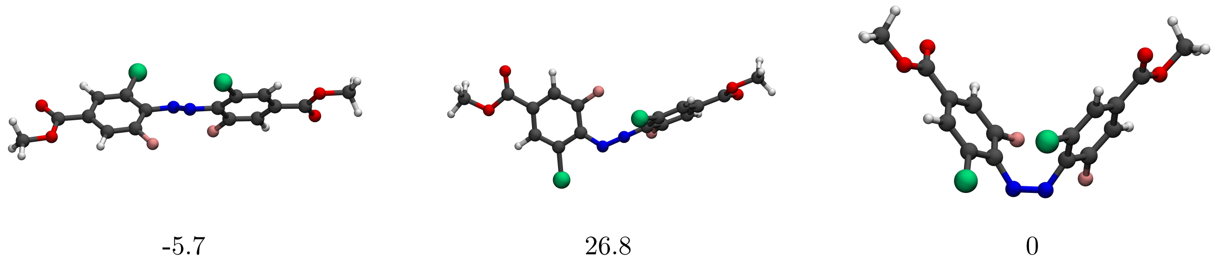

Figure S3.58. 3D-Structures and relative energies in kcal/mol (in implicit solvent; DMSO; corrected for ZPVE; referenced to *cis*) of the energetically lowest *trans*- (left), transition state- (middle) *cis*- (right) conformer.

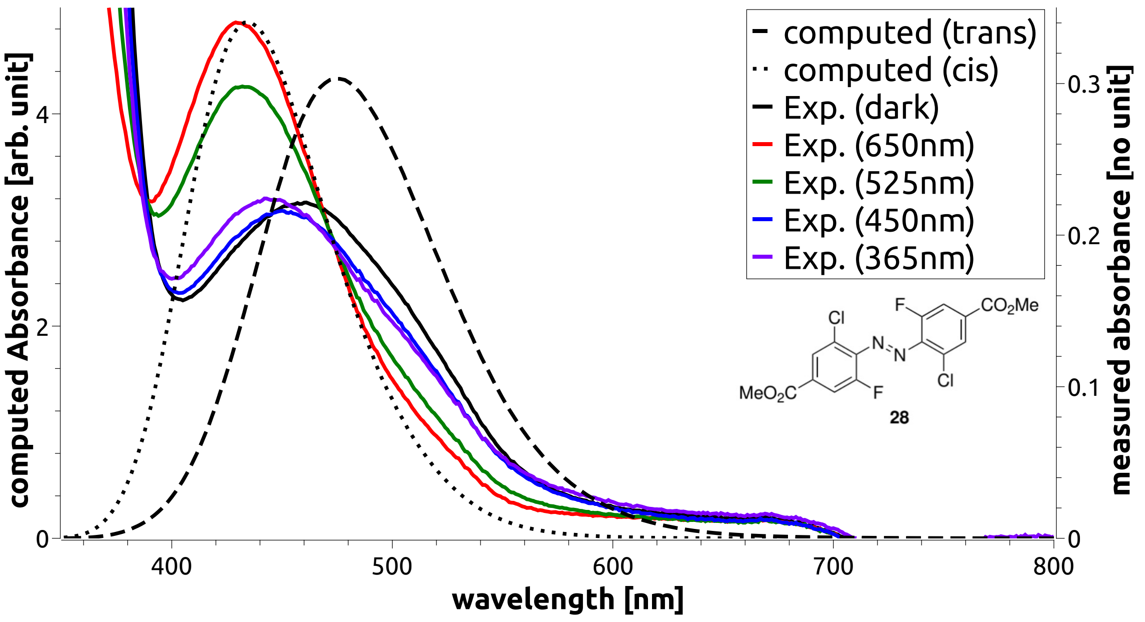

Figure S3.59. Computed absorption spectra (implicit solvent; DMSO; blue-shifted by 900 cm^{-1}) only including lowest energy ($n \to \pi^*$) excitation for *trans* and *cis* conformer and experimental spectra (500 µM irradiation times: 650 nm - 585 min; 525 nm - 15 min; 450 nm - 15 min; 365 nm - 15 min).

3.29. Molecule 29

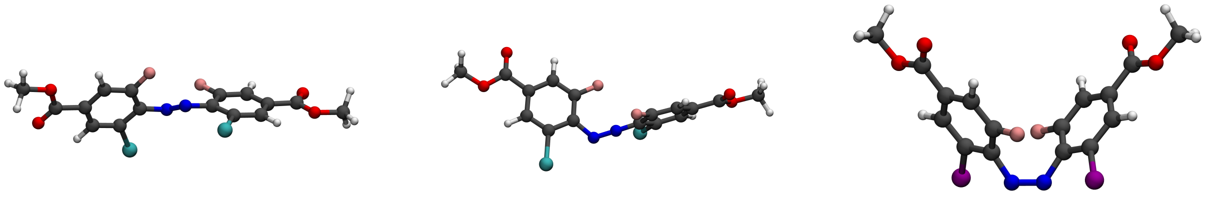

-4.8 27.4 0

Figure S3.60. 3D-Structures and relative energies in kcal/mol (in implicit solvent; DMSO; corrected for ZPVE; referenced to *cis*) of the energetically lowest *trans*- (left), transition state- (middle) *cis*- (right) conformer.

3.30. Molecule 30

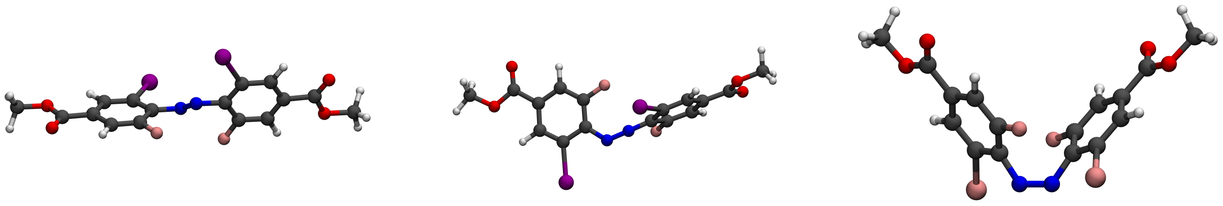

-3.1 29.4 0

Figure S3.61. 3D-Structures and relative energies in kcal/mol (in implicit solvent; DMSO; corrected for ZPVE; referenced to *cis*) of the energetically lowest *trans*- (left), transition state- (middle) *cis*- (right) conformer.

3.31. Molecule 31

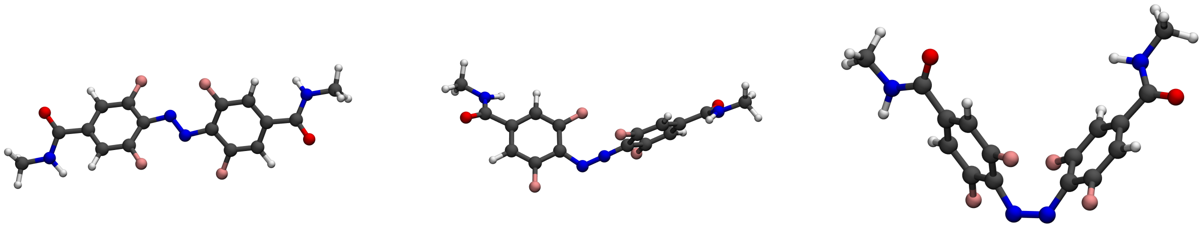

-6.1 28.7 0

Figure S3.62. 3D-Structures and relative energies in kcal/mol (in implicit solvent; DMSO; corrected for ZPVE; referenced to *cis*) of the energetically lowest *trans*- (left), transition state- (middle) *cis*- (right) conformer.

3.32. Molecule 32

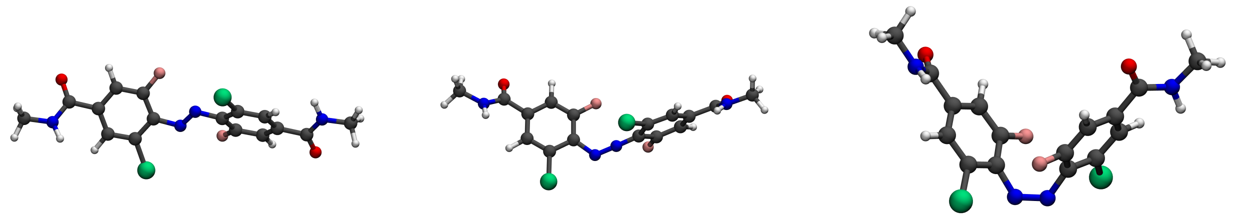

-4.9 29.3 0

Figure S3.63. 3D-Structures and relative energies in kcal/mol (in implicit solvent; DMSO; corrected for ZPVE; referenced to *cis*) of the energetically lowest *trans*- (left), transition state- (middle) *cis*- (right) conformer.

3.33. Molecule 33

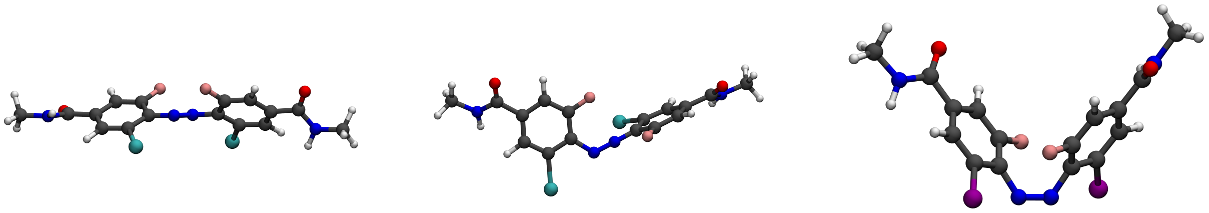

-5.0   28.9   0

Figure S3.64. 3D-Structures and relative energies in kcal/mol (in implicit solvent; DMSO; corrected for ZPVE; referenced to *cis*) of the energetically lowest *trans*- (left), transition state- (middle) *cis*- (right) conformer.

3.34. Molecule 34

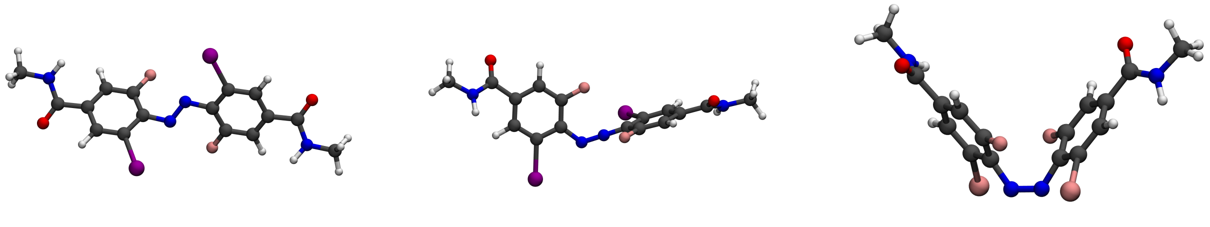

-5.3 28.9 0

Figure S3.65. 3D-Structures and relative energies in kcal/mol (in implicit solvent; DMSO; corrected for ZPVE; referenced to *cis*) of the energetically lowest *trans*- (left), transition state- (middle) *cis*- (right) conformer.

3.35. Molecule 35

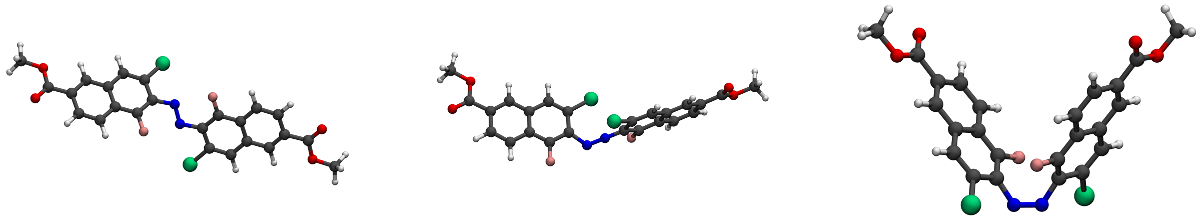

-4.8 | 30.1 | 0

Figure S3.66. 3D-Structures and relative energies in kcal/mol (in implicit solvent; DMSO; corrected for ZPVE; referenced to *cis*) of the energetically lowest *trans*- (left), transition state- (middle) *cis*- (right) conformer.

3.36. Molecule 36

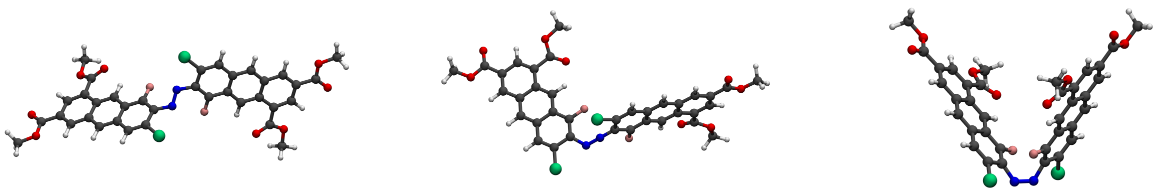

-4.9 29.8 0

Figure S3.67. 3D-Structures and relative energies in kcal/mol (in implicit solvent; DMSO; corrected for ZPVE; referenced to *cis*) of the energetically lowest *trans*- (left), transition state- (middle) *cis*- (right) conformer.

3.37. Molecule 37

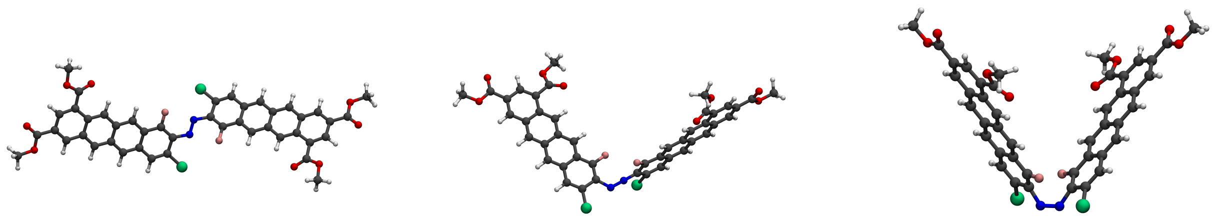

-5.0   30.2   0

Figure S3.68. 3D-Structures and relative energies in kcal/mol (in implicit solvent; DMSO; corrected for ZPVE; referenced to *cis*) of the energetically lowest *trans*- (left), transition state- (middle) *cis*- (right) conformer.

3.38. Molecule 38

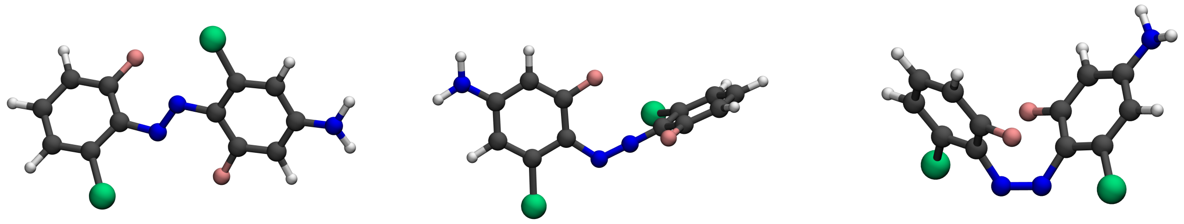

Figure S3.69. 3D-Structures and relative energies in kcal/mol (in implicit solvent; DMSO; corrected for ZPVE; referenced to *cis*) of the energetically lowest *trans*- (left), transition state- (middle) *cis*- (right) conformer.

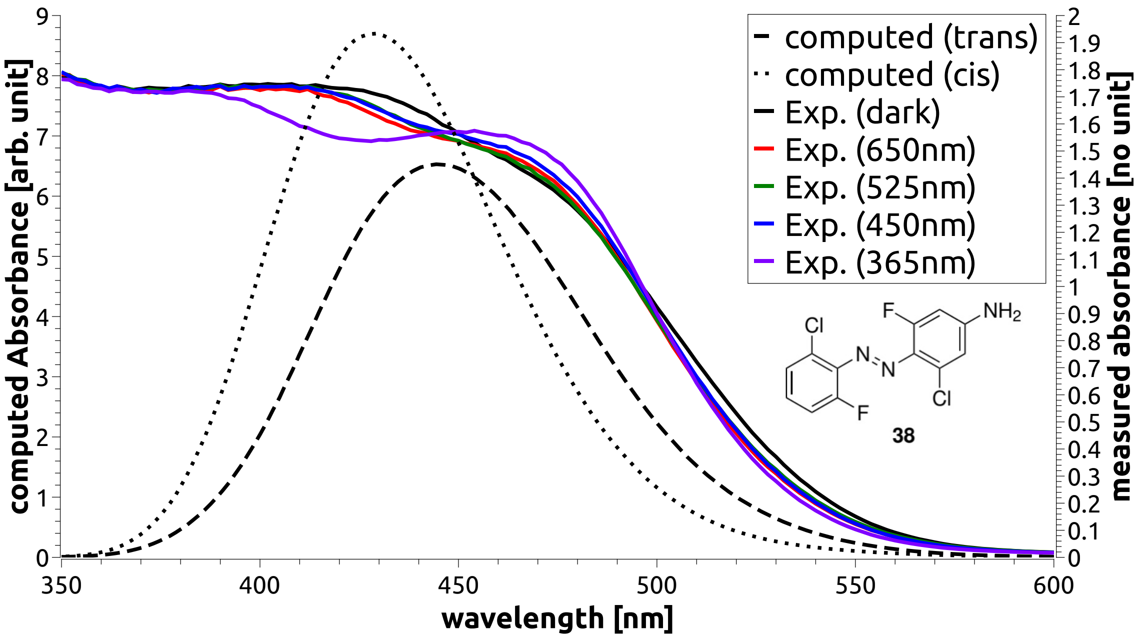

Figure S3.70. Computed absorption spectra (implicit solvent; DMSO; blue-shifted by 900 cm^{-1}) only including lowest energy ($n \rightarrow \pi^*$) excitation for *trans* and *cis* conformer and experimental spectra (500 µM irradiation times: 650 nm - 585 min; 525 nm - 15 min; 450 nm - 15 min; 365 nm - 15 min).

3.39. Molecule 39

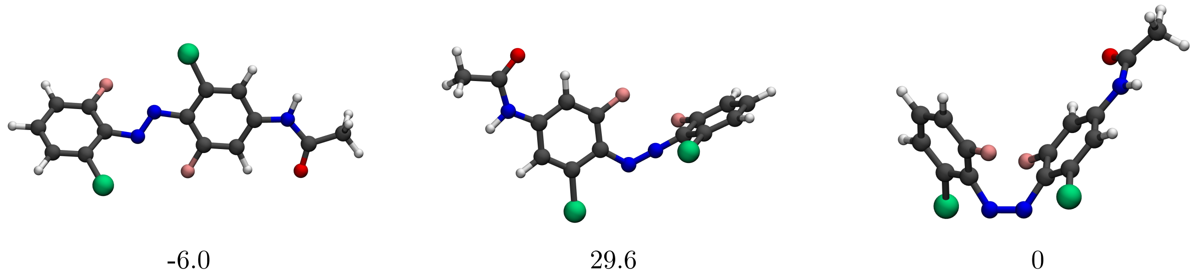

Figure S3.71. 3D-Structures and relative energies in kcal/mol (in implicit solvent; DMSO; corrected for ZPVE; referenced to *cis*) of the energetically lowest *trans*- (left), transition state- (middle) *cis*- (right) conformer.

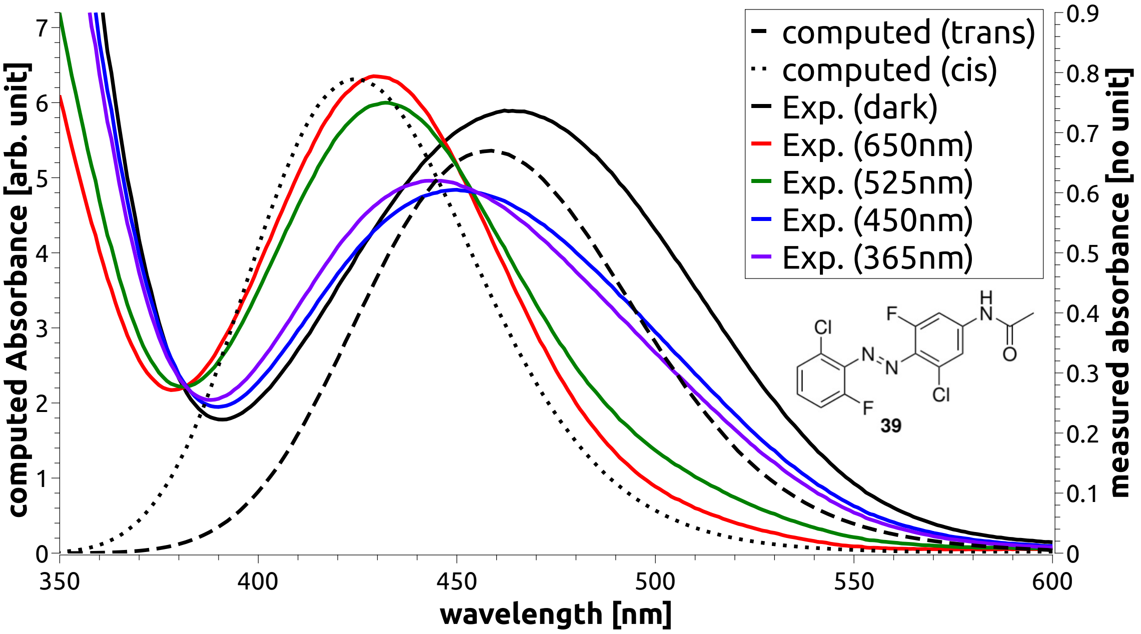

Figure S3.72. Computed absorption spectra (implicit solvent; DMSO; blue-shifted by 900 cm^{-1}) only including lowest energy ($n \rightarrow \pi^*$) excitation for *trans* and *cis* conformer and experimental spectra (500 µM irradiation times: 650 nm - 585 min; 525 nm - 15 min; 450 nm - 15 min; 365 nm - 15 min).

3.40. Molecule 40

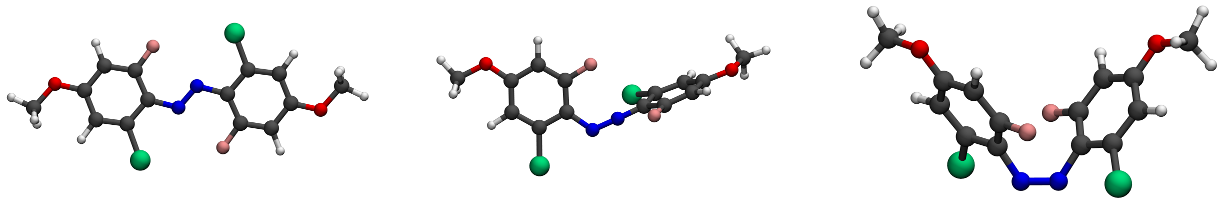

-6.8 30.8 0

Figure S3.73. 3D-Structures and relative energies in kcal/mol (in implicit solvent; DMSO; corrected for ZPVE; referenced to *cis*) of the energetically lowest *trans-* (left), transition state- (middle) *cis-* (right) conformer.

3.41. Molecule 41

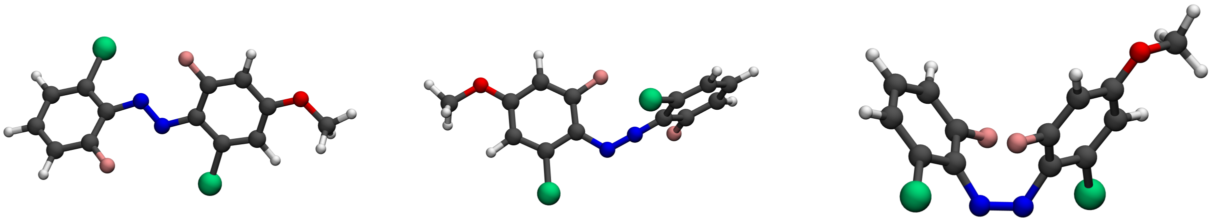

-6.2 29.2 0

Figure S3.74. 3D-Structures and relative energies in kcal/mol (in implicit solvent; DMSO; corrected for ZPVE; referenced to *cis*) of the energetically lowest *trans*- (left), transition state- (middle) *cis*- (right) conformer.

3.42. Molecule 42

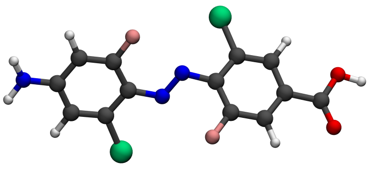

-7.7

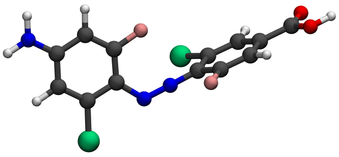

21.7

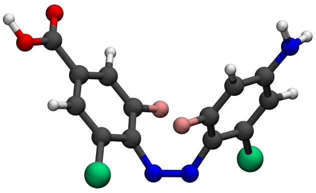

0

Figure S3.75. 3D-Structures and relative energies in kcal/mol (in implicit solvent; DMSO; corrected for ZPVE; referenced to *cis*) of the energetically lowest *trans*- (left), transition state- (middle) *cis*- (right) conformer.

3.43. Molecule 43

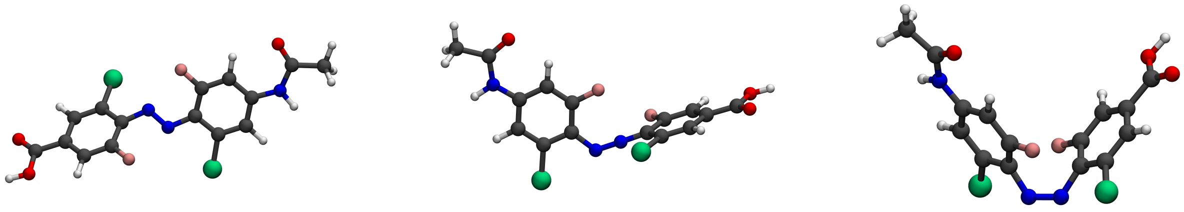

-6.1 24.6 0

Figure S3.76. 3D-Structures and relative energies in kcal/mol (in implicit solvent; DMSO; corrected for ZPVE; referenced to *cis*) of the energetically lowest *trans*- (left), transition state- (middle) *cis*- (right) conformer.

3.44. Molecule 44

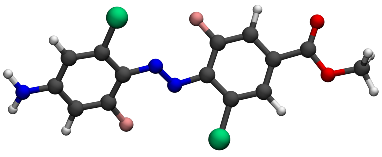
-7.6

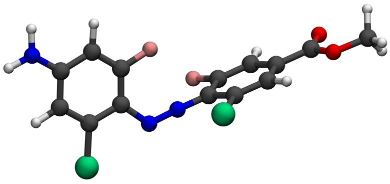
22.1

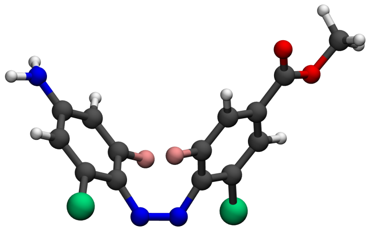
0

Figure S3.77. 3D-Structures and relative energies in kcal/mol (in implicit solvent; DMSO; corrected for ZPVE; referenced to *cis*) of the energetically lowest *trans*- (left), transition state- (middle) *cis*- (right) conformer.

3.45. Molecule 45

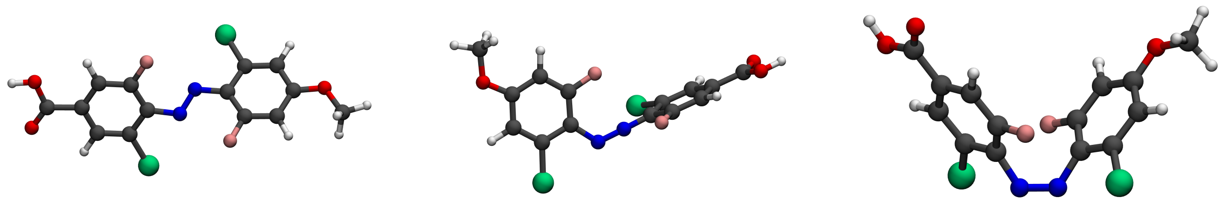

-6.1 24.3 0

Figure S3.78. 3D-Structures and relative energies in kcal/mol (in implicit solvent; DMSO; corrected for ZPVE; referenced to *cis*) of the energetically lowest *trans*- (left), transition state- (middle) *cis*- (right) conformer.

3.46. Molecule 46

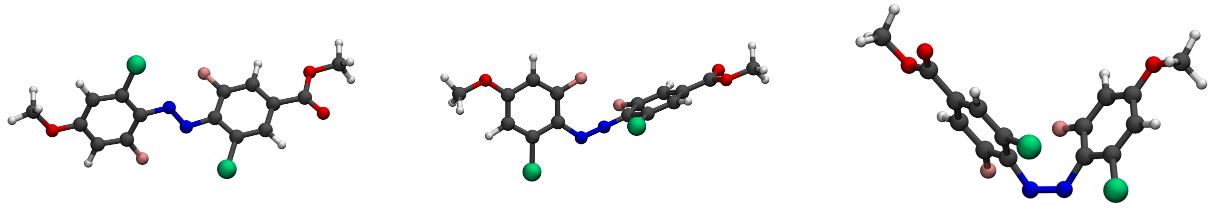

-6.1 24.8 0

Figure S3.79. 3D-Structures and relative energies in kcal/mol (in implicit solvent; DMSO; corrected for ZPVE; referenced to *cis*) of the energetically lowest *trans*- (left), transition state- (middle) *cis*- (right) conformer.

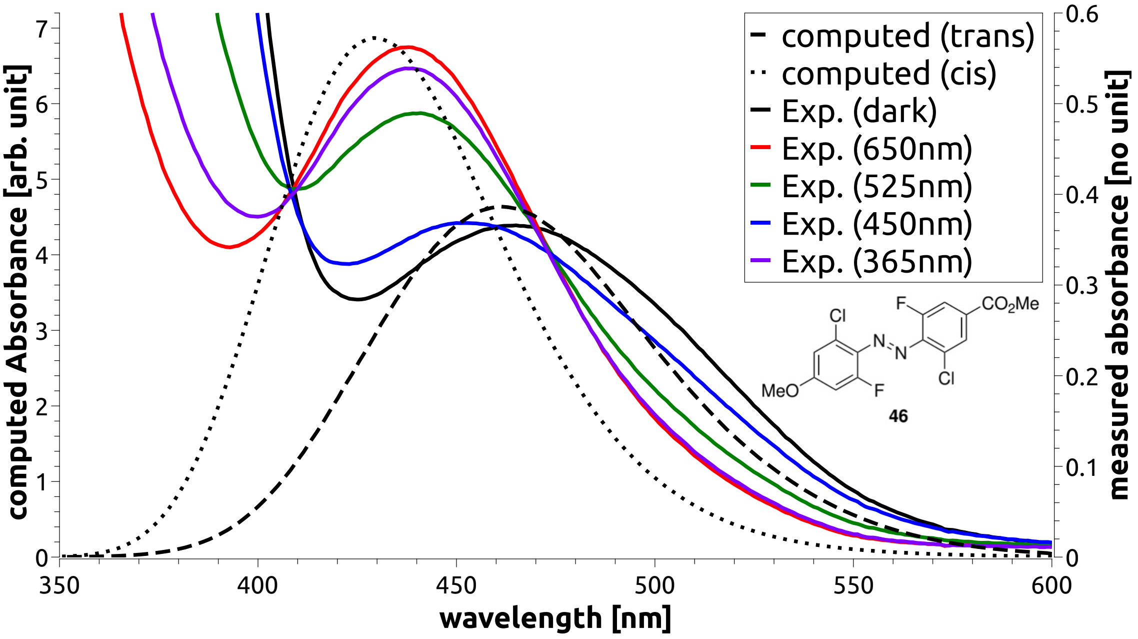

Figure S3.80. Computed absorption spectra (implicit solvent; DMSO; blue-shifted by 900 cm^{-1}) only including lowest energy ($n \rightarrow \pi^*$) excitation for *trans* and *cis* conformer and experimental spectra (500 µM irradiation times: 650 nm - 585 min; 525 nm - 15 min; 450 nm - 15 min; 365 nm - 15 min).

3.47. Molecule 47

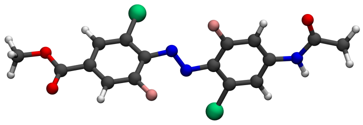

-5.9

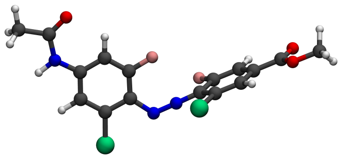

24.9

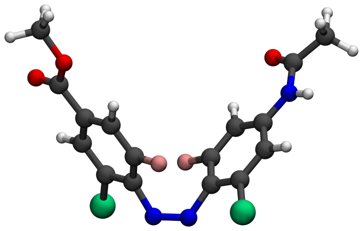

0

Figure S3.81. 3D-Structures and relative energies in kcal/mol (in implicit solvent; DMSO; corrected for ZPVE; referenced to *cis*) of the energetically lowest *trans*- (left), transition state- (middle) *cis*- (right) conformer.

3.48. Molecule 48

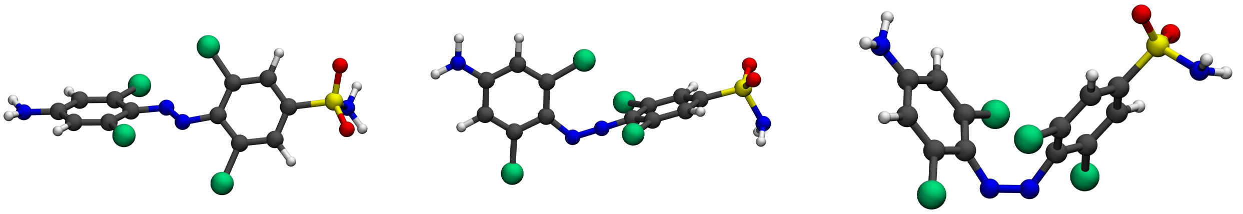

-7.4 18.9 0

Figure S3.82. 3D-Structures and relative energies in kcal/mol (in implicit solvent; DMSO; corrected for ZPVE; referenced to *cis*) of the energetically lowest *trans*- (left), transition state- (middle) *cis*- (right) conformer.

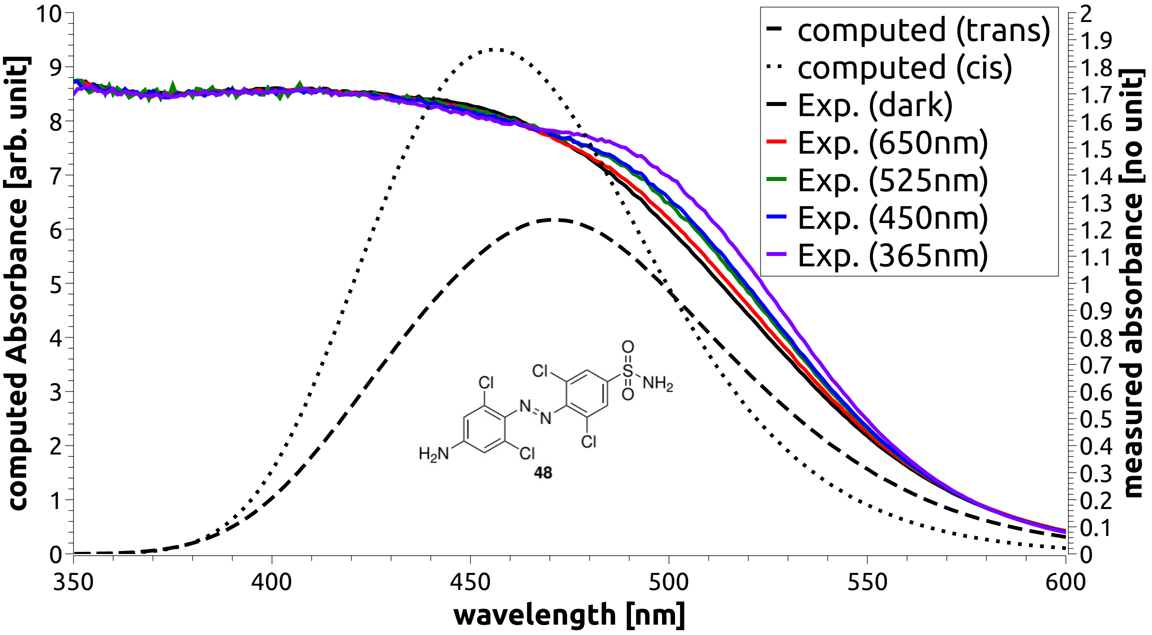

Figure S3.83. Computed absorption spectra (implicit solvent; DMSO; blue-shifted by 900 cm^{-1}) only including lowest energy ($n \rightarrow \pi^*$) excitation for *trans* and *cis* conformer and experimental spectra (500 µM irradiation times: 650 nm - 585 min; 525 nm - 15 min; 450 nm - 15 min; 365 nm - 15 min).

3.49. Molecule 49

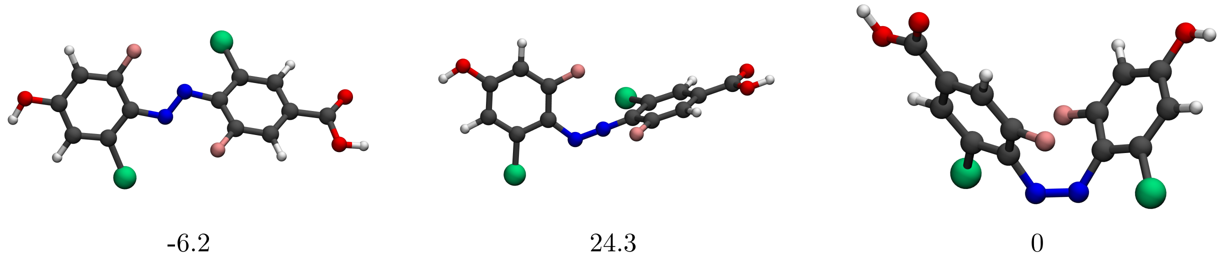

Figure S3.84. 3D-Structures and relative energies in kcal/mol (in implicit solvent; DMSO; corrected for ZPVE; referenced to *cis*) of the energetically lowest *trans*- (left), transition state- (middle) *cis*- (right) conformer.

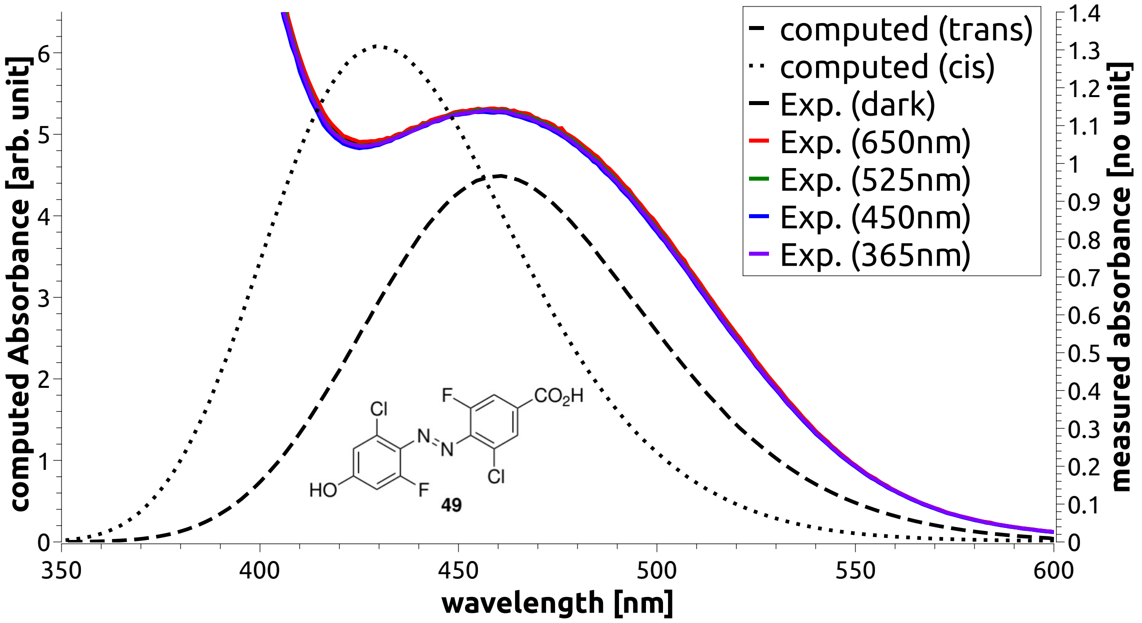

Figure S3.85. Computed absorption spectra (implicit solvent; DMSO; blue-shifted by 900 cm^{-1}) only including lowest energy ($n \rightarrow \pi^*$) excitation for *trans* and *cis* conformer and experimental spectra (500 µM irradiation times: 650 nm - 585 min; 525 nm - 15 min; 450 nm - 15 min; 365 nm - 15 min).

3.50. Molecule 50

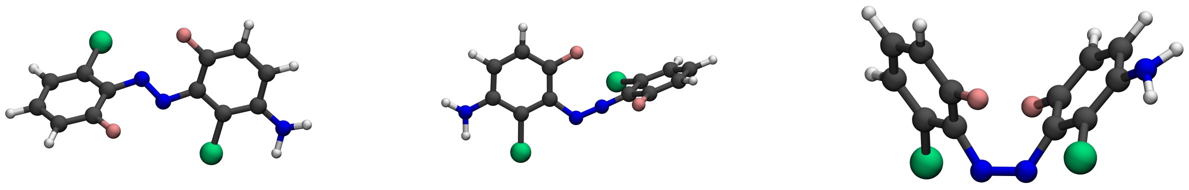

-4.9 31.1 0

Figure S3.86. 3D-Structures and relative energies in kcal/mol (in implicit solvent; DMSO; corrected for ZPVE; referenced to *cis*) of the energetically lowest *trans*- (left), transition state- (middle) *cis*- (right) conformer.

3.51. Molecule 51

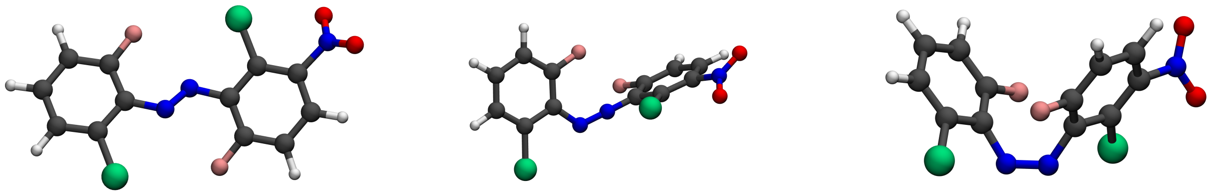

-5.1 28.9 0

Figure S3.87. 3D-Structures and relative energies in kcal/mol (in implicit solvent; DMSO; corrected for ZPVE; referenced to *cis*) of the energetically lowest *trans*- (left), transition state- (middle) *cis*- (right) conformer.

3.52. Molecule 52

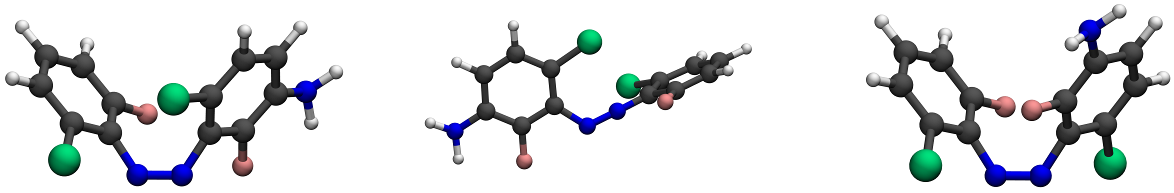

-5.0 31.1 0

Figure S3.88. 3D-Structures and relative energies in kcal/mol (in implicit solvent; DMSO; corrected for ZPVE; referenced to *cis*) of the energetically lowest *trans*- (left), transition state- (middle) *cis*- (right) conformer.

3.53. Molecule 53

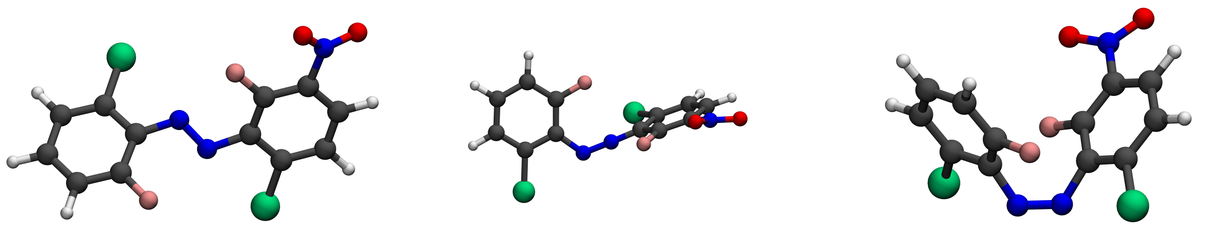

-5.2 29.0 0

Figure S3.89. 3D-Structures and relative energies in kcal/mol (in implicit solvent; DMSO; corrected for ZPVE; referenced to *cis*) of the energetically lowest *trans*- (left), transition state- (middle) *cis*- (right) conformer.

3.54. Molecule 54

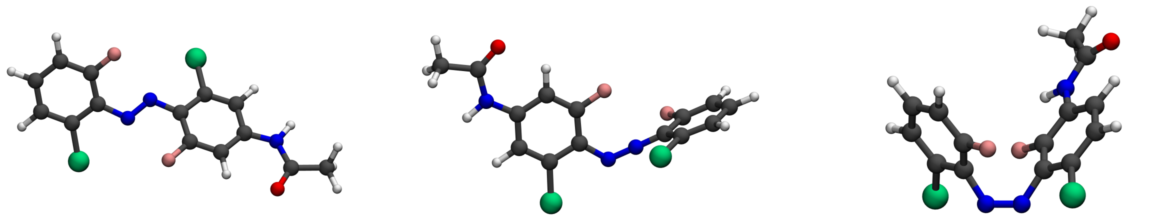

-8.8 26.8 0

Figure S3.90. 3D-Structures and relative energies in kcal/mol (in implicit solvent; DMSO; corrected for ZPVE; referenced to *cis*) of the energetically lowest *trans*- (left), transition state- (middle) *cis*- (right) conformer.

3.55. Molecule 55

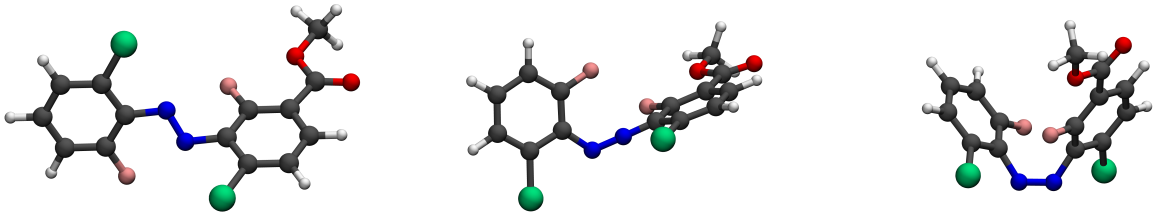

-4.9 30.4 0

Figure S3.91. 3D-Structures and relative energies in kcal/mol (in implicit solvent; DMSO; corrected for ZPVE; referenced to *cis*) of the energetically lowest *trans*- (left), transition state- (middle) *cis*- (right) conformer.

4. Benchmarks

4.1. Barrier Heights

In Table S3.4 we compare the computed *cis*→*trans*-reaction barriers predicted by various density functional approximations (DFAs). All computational results employ PBEh-3c optimized geometries and the zero-point vibrational energies are also obtained from the PBEh-3c vibrational frequencies. While there are significant differences between DFAs regarding signed errors, all tested methods perform quite similar after correcting for this systematic error (mcRMSD), which is irrelevant when analyzing only *differences* in reactivity. In practice, this means we can expect typical ($1\,\sigma$) errors of about one order of magnitude for the computational prediction of differences in thermal half-lives. The largely similar performance of all tested DFAs indicates that the remaining discrepancies of 2-3 kcal/mol with respect to the experimental barriers are most likely not due to the approximation made in the DFA but instead rooted in the simplicity of our reactivity model, in particular the neglect of explicit solvent effects.

Table S3.4. Computed barrier height [kcal/mol] benchmark (in vacuum) for various density functional approximations (DFAs). Experimental free energy barriers (Exp.) were computed from experimental half-lives using the Ayring equation. Mean signed error (MSE), root mean squared deviation (RMSD) and mean-corrected RMSD (mcRMSD) are summarized over all data points where experimental data is available. For PBEh-3c and ωB97-3c the corresponding basis sets were employed, whereas for all other DFAs the def2-TZVP [15] basis set was employed.

Molecule	PBEh-3c [5]	ωB97-3c [20]	HF	PBE0 [13,14]	r^2-SCAN [21]	rc-ωPBEh [22]	wB97M-V [23]	wB97X-V [24]	Exp.
MSE	1.29	2.85	3.71	-2.97	-2.42	-1.28	4.03	3.54	-
RMSD	2.14	3.30	4.36	3.61	3.11	2.41	4.28	3.89	-
mcRMSD	1.85	1.99	2.46	1.95	1.94	1.97	2.02	2.04	-
1	26.08	27.58	29.18	23.16	23.42	24.13	27.81	27.58	25.60
2	31.09	32.35	34.25	27.17	28.02	28.55	33.33	32.95	28.57
3	29.83	31.37	34.40	26.83	27.31	28.14	32.14	31.86	28.52
4	29.03	30.86	32.17	25.50	26.17	26.99	31.77	31.35	28.01
5	28.81	30.58	32.19	25.37	25.79	27.00	31.66	31.24	-
6	26.33	26.43	26.49	21.39	21.96	22.73	27.86	27.28	26.28
7	23.71	24.77	24.45	19.69	20.01	21.03	25.92	25.33	-
8	20.79	22.42	21.89	17.44	17.52	18.76	23.62	22.99	-
9	30.75	33.26	33.54	25.35	26.39	27.18	34.24	33.88	28.43
10	25.46	27.48	28.81	22.45	22.52	23.78	27.94	27.70	-
11	30.67	32.04	33.84	26.27	26.70	28.00	33.02	32.61	-
12	30.00	31.58	33.49	25.72	25.96	27.59	32.78	32.38	26.02
13	25.09	26.77	27.94	21.69	21.70	22.94	27.13	26.82	-
14	29.79	31.93	34.15	25.28	24.90	27.77	33.35	33.03	-
15	24.80	26.59	27.82	21.20	20.74	22.73	26.96	26.65	-
16	29.65	31.84	34.59	25.07	24.01	27.74	33.10	32.77	-
17	25.91	27.45	28.33	21.59	22.16	23.40	28.74	28.22	27.68
18	22.98	23.53	23.23	17.97	18.38	19.65	25.10	24.45	-
19	24.30	26.16	27.22	19.96	20.22	22.16	27.73	27.18	19.17
20	24.78	26.15	26.32	20.03	20.42	22.02	27.83	27.23	22.02
21	26.31	28.12	28.98	22.15	22.71	23.97	29.27	28.78	27.35
22	25.90	27.88	28.54	22.07	22.68	23.78	28.97	28.47	27.02
23	23.34	24.18	23.82	18.52	18.96	20.17	25.60	24.99	24.50
24	27.56	29.45	30.52	23.56	24.10	25.33	30.45	30.01	27.77
25	24.18	24.81	24.23	19.60	20.02	21.13	26.29	25.70	-
26	27.48	29.13	29.92	23.30	23.97	24.95	30.23	29.76	-
27	28.40	29.90	31.34	24.34	24.95	25.96	30.89	30.47	-
28	26.50	27.67	27.52	21.73	22.44	23.34	28.99	28.39	24.94
29	26.96	28.66	29.25	23.15	23.92	24.63	29.71	29.22	-
30	26.64	28.40	28.94	23.04	23.88	24.52	29.56	29.05	-
31	27.97	29.15	30.70	23.78	24.50	25.28	30.08	29.69	-
32	28.39	30.11	31.25	24.25	24.66	26.02	31.10	30.67	-

33	28.08	29.79	30.61	24.15	24.75	25.73	30.71	30.27	-
34	27.49	29.17	29.91	23.95	24.67	25.42	30.28	29.78	-
35	29.17	31.23	34.16	25.79	25.82	27.63	32.12	31.84	-
36	29.01	31.78	34.07	24.70	23.75	27.49	33.13	32.83	-
37	27.43	28.55	28.60	21.90	21.37	24.08	29.42	29.00	-
38	26.83	28.61	30.23	22.92	23.73	24.46	29.79	29.32	25.15
39	28.45	29.91	31.22	24.40	25.21	25.89	30.99	30.55	27.39
40	29.72	32.33	34.21	26.79	26.78	28.92	33.49	33.18	-
41	27.95	29.51	30.80	23.88	24.64	25.43	30.70	30.20	-
42	22.80	24.70	26.23	18.55	18.92	20.61	26.29	25.70	-
43	24.50	26.31	27.21	20.13	20.54	22.12	27.70	27.16	-
44	23.12	25.40	26.54	18.94	19.31	21.01	26.72	26.17	-
45	24.35	25.95	26.97	19.85	20.22	21.82	27.48	26.89	-
46	23.88	25.43	25.23	18.92	19.40	20.91	27.00	26.31	25.00
47	24.79	26.81	27.54	20.57	21.04	22.52	28.06	27.54	-
48	20.03	21.85	21.21	15.18	15.51	17.20	23.38	22.60	19.74
49	24.42	26.19	27.07	19.96	20.37	21.94	27.52	26.96	16.41
50	29.76	31.14	32.84	25.47	26.22	26.98	32.17	31.77	-
51	27.74	29.06	30.95	23.59	24.31	25.13	30.32	29.88	-
52	29.53	31.25	34.46	26.37	26.80	27.82	32.10	31.87	-
53	28.22	29.75	31.78	24.05	24.84	25.56	30.99	30.53	-
54	26.92	29.30	30.07	23.30	24.01	24.90	30.33	29.97	-
55	29.65	31.03	32.82	25.39	26.13	26.87	32.23	31.82	-

4.2. Excitation energies

In Table S3.5 we provide an excited state benchmark for various DFAs regarding the excitation energy of the $n \rightarrow \pi^*$ excitation of the azobenzene derivatives studied in this work. To facilitate this benchmark, we selected 12 structures randomly out of the of the 6000 structures that were used to generate the excitation spectra for each structure (*cis* and *trans* count separately) for a total of 1320 data points. Again, we removed systematic errors in the mean-corrected RMSD (mcRMSD) to analyze the accuracy for differences between structures instead of absolute values. Here, we observe that ADC(2) matches very well (0.02 eV mcRMSD) with the SCS-ADC(2) reference, justifying our choice for SCS-ADC(2) as the reference method. Moreover, most tested DFAs provide a relative accuracy (mcRMSD) on the order of 0.1 eV with the notable exception of r^2-SCAN, the only non-hybrid DFA tested, with an mcRMSD of 0.23 eV. An error of 0.1 eV is equal to about 4 % relative error or about 20 nm error at a wavelength of 500 nm, which pretty much matches our observed discrepancies between computed vs. experimental spectra.

Table S3.5. Error statistics [eV] for the first ($n \rightarrow \pi^*$) excitation of various density functional approximations (DFAs) referenced to SCS-ADC(2)/def2-TZVP [25]. The benchmark contains 12 randomly selected structures for each of the 110 azobenzene derivatives (*cis* and *trans* isomers) investigated in this work resulting in a total of 1320 structures. For PBEh-3c and ωB97-3c the corresponding basis sets were utilized, whereas for all other DFAs the def2-TZVP [15] basis set was employed.

	ADC(2) [26]	PBEh-3c [5]	ωB97-3c [20]	r^2-SCAN [21]	rc-ωPBEh [22]	wB97M-V [23]	wB97X-V [24]
MSE [eV]	-0.20	-0.26	-0.23	-0.55	-0.21	-0.08	-0.10
RMSD [eV]	0.20	0.27	0.24	0.59	0.25	0.17	0.18
mcRMSD [eV]	0.02	0.09	0.09	0.23	0.13	0.13	0.13

References

[1] P. Pracht, S. Grimme, C. Bannwarth, F. Bohle, S. Ehlert, G. Feldmann, J. Gorges, M. Muller, T. Neudecker, C. Plett, S. Spicher, P. Steinbach, P. A. Wesolowski, F. Zeller, *J. Chem. Phys.* **2024**, *160*, 114110.
[2] S. Grimme, C. Bannwarth, P. Shushkov, *J. Chem. Theory Comput.* **2017**, *13*, 1989.
[3] C. Bannwarth, S. Ehlert, S. Grimme, *J. Chem. Theory Comput.* **2019**, *15*, 1652.
[4] S. Grimme, C. Bannwarth, E. Caldeweyher, J. Pisarek, A. Hansen, *J. Chem. Phys.* **2017**, *147*, 161708.
[5] S. Grimme, J. G. Brandenburg, C. Bannwarth, A. Hansen, *J. Chem. Phys.* **2015**, *143*, 054107.
[6] J. Kussmann, M. Beer, C. Ochsenfeld, *Wiley Interdiscip. Rev.: Comput. Mol. Sci.* **2013**, *3*, 614.
[7] J. Kussmann, C. Ochsenfeld, *J. Chem. Theory Comput.* **2015**, *11*, 918.
[8] J. Kastner, J. M. Carr, T. W. Keal, W. Thiel, A. Wander, P. Sherwood, *J. Phys. Chem. A* **2009**, *113*, 11856.
[9] Y. Lu, M. R. Farrow, P. Fayon, A. J. Logsdail, A. A. Sokol, C. R. A. Catlow, P. Sherwood, T. W. Keal, *J. Chem. Theory Comput.* **2019**, *15*, 1317.
[10] G. Bussi, D. Donadio, M. Parrinello, *J. Chem. Phys.* **2007**, *126*, 014101.
[11] M. E. Casida, *Theor. Comput. Chem.* **1996**, *4*, 391.
[12] J. P. Perdew, K. Burke, M. Ernzerhof, *Phys. Rev. Lett.* **1996**, *77*, 3865.
[13] C. Adamo, V. Barone, *J. Chem. Phys.* **1999**, *110*, 6158.
[14] M. Ernzerhof, G. E. Scuseria, *J. Chem. Phys.* **1999**, *110*, 5029.
[15] F. Weigend, R. Ahlrichs, *Phys. Chem. Chem. Phys.* **2005**, *7*, 3297.
[16] L. Goerigk, A. Hansen, C. Bauer, S. Ehrlich, A. Najibi, S. Grimme, *Phys. Chem. Chem. Phys.* **2017**, *19*, 32184.
[17] M. R. Silva-Junior, M. Schreiber, S. P. A. Sauer, W. Thiel, *J. Chem. Phys.* **2008**, *129*, 104103.
[18] T. N. Truong, E. V. Stefanovich, *Chem. Phys. Lett.* **1995**, *240*, 253.
[19] V. Barone, M. Cossi, *J. Phys. Chem. A* **1998**, *102*, 1995.
[20] M. Mueller, A. Hansen, S. Grimme, *J. Chem. Phys.* **2023**, *158*, 014103.
[21] J. W. Furness, A. D. Kaplan, J. Ning, J. P. Perdew, J. Sun, *J. Phys. Chem. Lett.* **2020**, *11*, 8208.
[22] M. A. Rohrdanz, K. M. Martins, J. M. Herbert, *J. Chem. Phys.* **2009**, *130*, 054112.
[23] N. Mardirossian, M. Head-Gordon, *J. Chem. Phys.* **2015**, *142*, 074111.
[24] N. Mardirossian, M. Head-Gordon, *Phys. Chem. Chem. Phys.* **2014**, *16*, 9904.
[25] A. Tajti, L. Tulipán, P. G. Szalay, *J. Chem. Theory Comput.* **2020**, *16*, 468.
[26] J. Schirmer, *Phys. Rev. A* **1982**, *26*, 2395.
