# Supplementary material for: A Platform for the Development of Highly Red‐Shifted Azobenzene‐Based Optical Tools
Source: Angew Chem Int Ed Engl. 2025 Jun 23;64(32):e202501779. doi: 10.1002/anie.202501779 (PMC12322654; doi:10.1002/anie.202501779)
Supplement: Supplementary file 4 — Supporting Information [file ANIE-64-e202501779-s002.pdf]

A platform for the development of Highly Red-Shifted Azobenzene-based Optical Tools

Kyra Lützel#, Henryk Laqua#, Manjima B. Sathian#, Benedikt Nißl#, Judit Katalin Szántó, Christina-Anna Senser, Gökcen Savasci, Lars Allmendinger, Bilal Kicin, Vincent Ruf, Dominik Kammerer, Theobald Lohmüller, Konstantin Karaghiosoff, Ahmed M. Ali, Ursula Storch*, Michael Mederos y Schnitzler*, Christian Ochsenfeld* and David B. Konrad*

[#] these authors contributed equally to this work

[*] corresponding authors

Supporting Information IV: Biological Study

Material and Methods

Materials

Dfdc OptoBI-1 was dissolved in anhydrous DMSO to give a 10 mM stock solution.

Cell culture and transfection

Human embryonic kidney (HEK293T) cells (from Leibniz-Institute DSMZ, Braunschweig, Germany, T293, DSMZ no. ACC 635) were used for electrophysiological measurements. HEK293T cells were kept in Earl's MEM (Sigma-Aldrich) with 100 units/mL penicillin and 100 µg/mL streptomycin supplemented with 10% (vol/vol) FCS (Gibco, Life Technologies, Carlsbad, USA). All cells were held at 37°C in a humified atmosphere with 5% CO_2. HEK293T cells were transfected with 2 µg cDNA coding for mouse TRPC6 (NM_013838) [1] using Genejuice reagent (Sigma-Aldrich) according to the manufacturer's instructions. The cDNA was in pIRES2-EGFP expression vector (Clontech, Palo Alto, CA). Transfected HEK293T cells were seeded onto poly-L-Lysine-coated glass cover slips (diameter 30 mm, thickness 1, Karl Hecht, Sondheim, Germany) 1 hour before patch clamp measurements.

Light stimulation

For light stimulation, three LEDs from Thorlabs (M365LP1 with a peak wavelength of 367 nm, M450LP2 with a peak wavelength of 442 nm, and Solis®-623C; Bergkirchen, Germany) were used. An Olympus IX70 microscope was used with dichroic beamsplitter (H 643 LPXR superflat) from AHF and the 40x oil UV-transmissive apochromatic objective (UApo N 340; Evident, Hamburg, Germany). The LEDs were operated by a self-made control unit with IC-HG30 laser switches mounted on EVAL HG1D evaluation boards from iC-Haus (Bodenheim, Germany) connected to the microcontroller board Arduino Mega 2560 (Arduino SA, Chiasso, Switzerland). A self-written MATLAB app (R2023a; MathWorks Inc.; Natick, Massachusetts, USA) served as user-interface driving the microcontroller through serial communication. The Solis®-623C-LED was controlled via a DC20 driver from Thorlabs.

Patch-clamp recordings

For patch-clamp measurements, **dfdc** OptoBI-1 stock solution was heated to 40 °C for 10 minutes to effect a thermal relaxation and diluted in standard bath solution containing 140 mM NaCl, 5 mM CsCl, 1 mM $MgCl_2$, 2 mM $CaCl_2$, 10 mM glucose, 10 mM HEPES (pH 7.4 with NaOH) resulting in an osmolarity of 295–302 mOsm·kg^{-1} to the final standard concentration of 10 µM just before the measurements. The solution was illuminated with blue light prior to application to the cells. Conventional whole-cell patch-clamp recordings were carried out at room temperature (23 °C) 18 h after transfection. Photoswitching with LEDs had no temperature effects. The standard pipette solution contained 120 mM CsCl, 9.4 mM NaCl, 0.2 mM Na_3-GTP, 1 mM $MgCl_2$, 3.949 mM $CaCl_2$, 10 mM BAPTA (100 nM free Ca^{2+}), and 10 mM HEPES (pH 7.2 with CsOH), resulting in an osmolality of 294 mOsm kg^{-1}. The liquid junction potential of +4.0 mV was calculated by JPCalcWin 1.01 (University of New South Wales, Sydney, Australia) and was corrected before the measurements. Data were collected with an EPC10 patch clamp amplifier (HEKA Elektronik, Lambrecht, Germany) using the Patchmaster software. Transfected cells were selected by application of light of the wavelength 445 nm to detect their green fluorescent protein EGFP.

For determination of current-voltage relationships, current-time course and current kinetics, a stimulation protocol was applied starting with holding potential of -100 mV for 7 ms followed by a voltage-upramp from -100 to +100 mV for 10 ms (slope of 20 V s^{-1} equivalent to 100 µV in 5 µs) and by a holding potential of +100 mV that was applied for 3 ms. This voltage-upramp protocol was applied with a frequency of 50 Hz. The current response at the holding potential of +100 and -100 mV was used

for analysis of current kinetics. Data were acquired at a frequency of 5 kHz after filtering at 2.5 kHz. Patch pipettes were made of borosilicate glass from Science Products (Hofheim, Germany; Cat. No. GB150TF-8P) and had resistances of 2.2 to 3.0 MΩ.

Fit routine

To determine the half-life time constant of activation and deactivation ($\tau_{1/2}$), currents determined at +100 and -100 mV with fast voltage-upramps, were normalized and the time was shifted to zero for fitting with MATLAB R2023a. For $\tau_{1/2}$ of activation, the median of the current before activation was set to zero and ca. 30% of the maximal activated current was set to +1 to exclude the emerging interfering inactivation. For $\tau_{1/2}$ of deactivation, the median of the current before deactivation was set to +1 and the median of fully deactivated current was set to zero. Activation and deactivation were fitted by a mono-exponential function (Formula 1 and Formula 2). Fit optimization was performed with a quadratic error function calculating the summed square of residuals (SSE) (Formula 3 and Formula 4) using the *fminserach* function and the options described in Table S4.1.

$$f_{activation}(t) = a * e^{\frac{\ln 2 * t}{\tau_H}} + c$$

Formula 1: Mono-exponential fit function $f_{activation}$ dependent on time (t), initial quantity (a), time constant (τ_H) and offset (c)

$$f_{deactivation}(t) = a * e^{-\frac{\ln 2 * t}{\tau_H}} + c$$

Formula 2: Mono-exponential fit function $f_{deactivation}$ dependent on time (t), initial quantity (a), time constant (τ_H) and offset (c)

$$SSE = \Sigma_t [y_t - f(a, c, \tau_H, t)]^2$$

Formula 3: Quadratic error function for mono-exponential fits using the summed square of residuals (SSE) with y_t being the current at time t and f(a, c, τ_H, t) the result either of formula 1 or formula 2 at time t

$$SSE = \Sigma_t [y_t - f(a_1, a_2, c, \tau_{H1}, \tau_{H2}, t)]^2$$

Formula 4: Quadratic error function for bi-exponential fits using the summed square of residuals (SSE) with y_t being the current at time t and f(a, c, τ_{H1}, τ_{H2}, t) the result of formula 3 at time t

Table S4.1

Parameter	Value
MaxFunEvals	5000
MaxIter	10000
TolX	$1*10^{-10}$
TolFun	$1*10^{-6}$
Initial values activation	a = 0.01; τ_H = 1; c = 0.01
Initial values deactivation	a = 0.7; τ_H = 7; c = 0.1

Reference

[1] X. Zhu, M. Jiang, M. Peyton, G. Boulay, R. Hurst, E. Stefani, L. Birnbaumer, *Cell* **1996**, *85*, 661-671.
